# Cationic-palladium catalyzed regio- and stereoselective *syn*-1,2-dicarbofunctionalization of unsymmetrical internal alkynes

**DOI:** 10.1038/s41467-022-28949-7

**Published:** 2022-03-16

**Authors:** Shubham Dutta, Shashank Shandilya, Shengwen Yang, Manash Protim Gogoi, Vincent Gandon, Akhila K. Sahoo

**Affiliations:** 1 School of Chemistry, University of Hyderabad, Hyderabad, India; 2 Institut de Chimie Moléculaire et des Matériaux d’Orsay, CNRS UMR 8182, Université Paris-Saclay, Bâtiment 420, Orsay cedex, France; 3 Laboratoire de Chimie Moléculaire (LCM), CNRS UMR 9168, Ecole Polytechnique, Université Paris-Saclay, route de Saclay, Palaiseau cedex, France

**Keywords:** Synthetic chemistry methodology, Homogeneous catalysis

## Abstract

π-Extended tetrasubstituted olefins are widely found motifs in natural products, leading drugs, and agrochemicals. Thus, development of modular strategies for the synthesis of complex all-carbon-substituted olefins always draws attention. The difunctionalization of unsymmetrical alkynes is an attractive approach but it has remained faced with regioselectivity issues. Here we report the discovery of a regio- and stereoselective *syn*-1,2-dicarbofunctionalization of unsymmetrical internal alkynes. A cationic Pd-catalyzed three-component coupling of aryl diazonium salts, aryl boronic acids (or olefins) and yne-acetates enables access to all-carbon substituted unsymmetrical olefins. The transformation features broad scope with labile functional group tolerance, building broad chemical space of structural diversity (94 molecules). The value of this synthetic method is demonstrated by the direct transformation of natural products and drug candidates containing yne-acetates, to enable highly substituted structurally complex allyl acetate analogues of biologically important compounds. Synthetic versatility of the carboxylate bearing highly substituted olefins is also presented. The reaction outcome is attributed to the in situ formation of stabilized cationic aryl-Pd species, which regulates regioselective aryl-palladation of unsymmetrical yne-acetates. Control experiments reveal the synergy between the carboxylate protecting group and the cationic Pd-intermediate in the regioselectivity and reaction productivity; density functional theory (DFT) studies rationalize the selectivity of the reaction.

## Abstract (web summary)

Methods to regioselectively difunctionalize unsymmetrical alkynes with two diverse carbon-based substituents are rare. Here the authors show a method to add two C(sp^2^)-based units in a *syn*, regioselective fashion across a triple bond, via palladium catalysis without an added ligand.

## Introduction

**Fig. 1:**
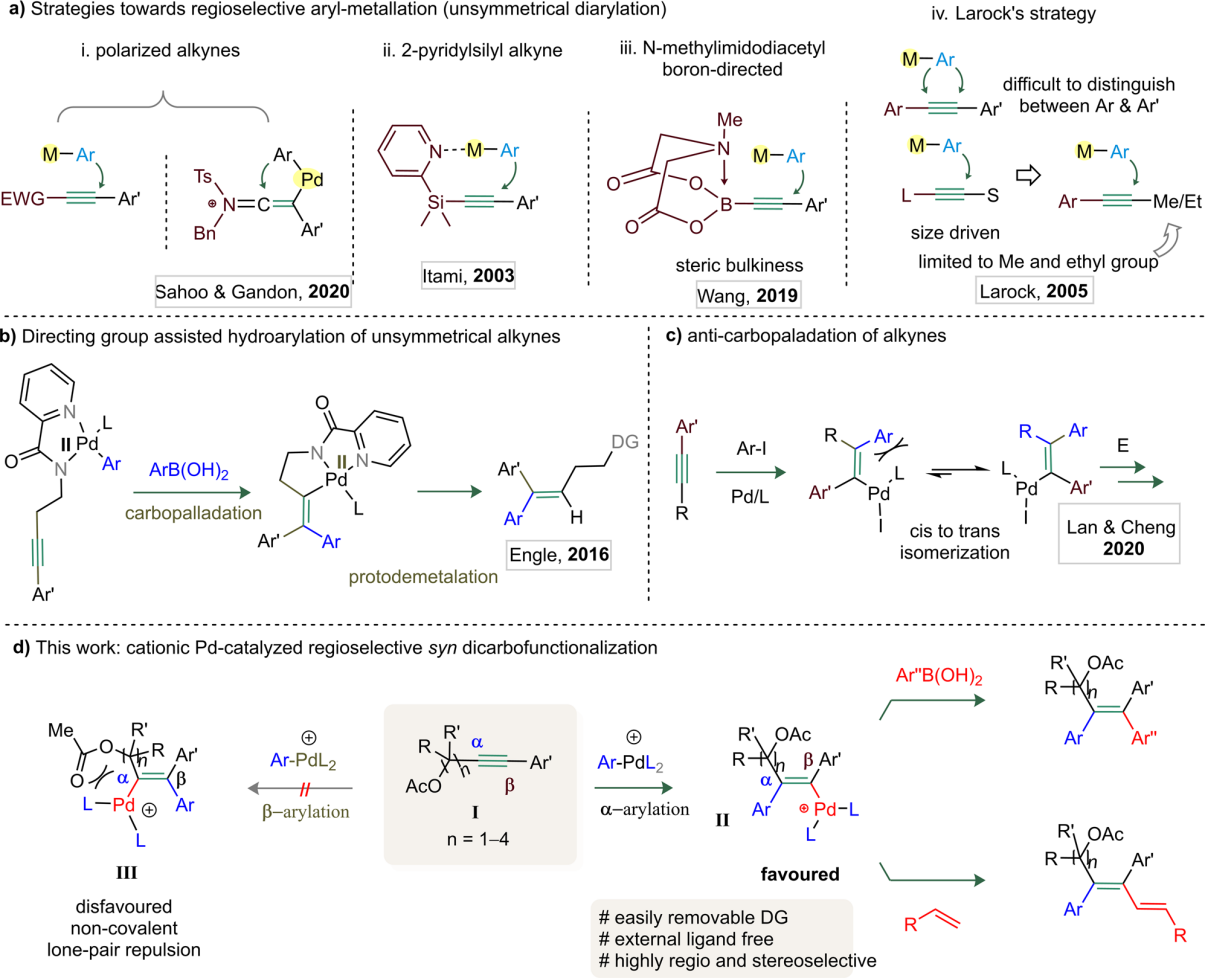
Comparison between hydro- and difunctionalization of alkynes and the conceptual blueprint of a general cationic Pd-catalyzed dicarbofunctionalization of Yne-acetates. **a** (i) Difunctionalization of electronically polarized alkynes: the incoming aryl group forms bond at the electron-deficient carbon center. (ii) Difunctionalization of 2-pyridylsilyl alkynes: the metal coordination to the pyridyl-N makes the C-aryl bond β to silyl group. (iii) Difunctionalization of N-methylimidodiacyl boron alkynes: the aryl group approaches the less-hindered side of borylated-alkyne. (iv) *syn*-1,2-Diarylation of diaryl-substituted alkynes occurs at an elevated temperature, providing a mixture of regioisomers. In case of aryl-alkyl alkynes, only the smaller alkyl groups (i.e., methyl/ethyl) allow the aryl-moiety to approach from the less-hindered side of the alkyne. **b** Pd-catalyzed aminopyridine-directed regioselective hydroarylation of alkynes happens through proximity-driven carbopalladation followed by protodemetalation. **c** *anti*-carbopalladation of alkyne driven by steric repulsion between *ortho*-substituted aryl halide and bulky-ligands. **d** Our strategy: regioselective dicarbofunctionalization of yne-acetates: because of the non-covalent lone pair repulsion between ligated Pd and the carbonyl of acetate group, the ligated cationic Pd species preferably approaches at the β -carbon over α-carbon of the yne-acetate; as a result, intermediate **II** is favored over intermediate **III**.

Tetrasubstituted and π-extended olefins are widespread in numerous natural products, leading drugs, and agrochemicals. They also hold potential applications in electron-transport materials and light-emitting diodes^1–4^. The carbometallation of unsaturated C–C bonds has always been a gateway to biologically valuable feedstocks. In this regard, the carbopalladation has been the most prominent and widely used process^5^. The transition metal-catalyzed alkyne dicarbofunctionalization by interrupting two cross-couplings [for example: Suzuki and Heck] offers a potential entry to highly substituted olefins. However, in contrast with the directed olefin dicarbofunctionalization^5–14^, this approach often suffers from insolvable regioselectivity issues. Mostly, the state-of-the-art regioselective carbometallation of unsymmetrical alkynes is restricted to inherently polarized substrates (Fig. 1a-i)^15–30^, 2-pyridylsilyl alkynes (Itami; Fig. 1a-ii)^31–33^ or sterically hindered borylated alkynes (Wang; Fig. 1a-iii)^34^. The Larock’s Pd-catalyzed *syn*-1,2-diarylation of unsymmetrical alkynes is also a great strategy to access all-carbon-substituted olefins (Fig. 1a-iv); however, it suffers from several limitations^35,36^. For instance, *syn*-1,2-diarylation of diaryl-substituted alkynes occurs at an elevated temperature to deliver a mixture of regioisomers in moderate selectivity (Fig. 1a-iv). In case of aryl-alkyl alkynes, the arylation takes place at the less-hindered site of alkyne, resulting in better regioselectivity (Fig. 1a-iv), but only with Me and Et groups. Thus, the *syn*-1,2-diarylation of unsymmetrical alkynes is substrate specific. An interesting aminopyridine directing group (DG) guided regioselective hydroarylation of alkynes with aryl boronic acids for the construction of trisubstituted olefins has also been described (Engle, Fig. 1b)^37,38^. Overall, DG, ligand and the electron-bias of the alkyne are essential features in regioselective alkyne difunctionalization. The recent reports of Lan and Cheng feature an impressive DG free *anti*-carbopalladation of internal alkynes, the steric clash between the *ortho*-substituted aryl halide and the bulky ligated Pd(II) species making the anti-addition to alkyne possible at an elevated temperature^39^. In addition, Werz and co-workers designed an *anti*-carbopalladation reaction within a cascade process via 14 valence electron Pd species as crucial intermediates^40–42^. In this context, the development of a ligand free regioselective syn-1,2-dicarbofunctionalization of unsymmetrical alkynes to build tetrasubstituted olefins appears to be a worthwhile endeavor.

We herein report a cationic Pd-catalyzed 1,2-dicarbofunctionalization of unsymmetrical alkynes (Fig. 1c). As per DFT studies (vide infra), the reaction relies on a site-selective coordination of a cationic Pd(II) species, generated in situ by the oxidative addition of an aryl diazonium salt^43,44^ to Pd(0), to an yne-acetate (**I**) (Fig. 1c).

This results in a *syn*-α-arylated-Pd-intermediate **II**. The lone pair repulsion between the carboxylate moiety and the Pd-complex possibly excludes *syn*-β-arylated-Pd-intermediate **III**. Further functionalization of vinyl-Pd(II)-cationic species **II** with aryl boronic acids (or olefins) delivers highly substituted olefins (or dienes). This strategy leads to structurally diverse all-carbon-functionalized olefins (94 molecules) in a single step from readily available yne-acetates. The transformation is highly regio- and stereoselective, even in the absence of external ligand and DG. A comparativereactivity profile and mechanistic pathway of the current method over Larock’s strategy for high regioselectivity throughput is established by various control experiments.

## Result and discussion

### Reaction optimization

**Fig. 2:**
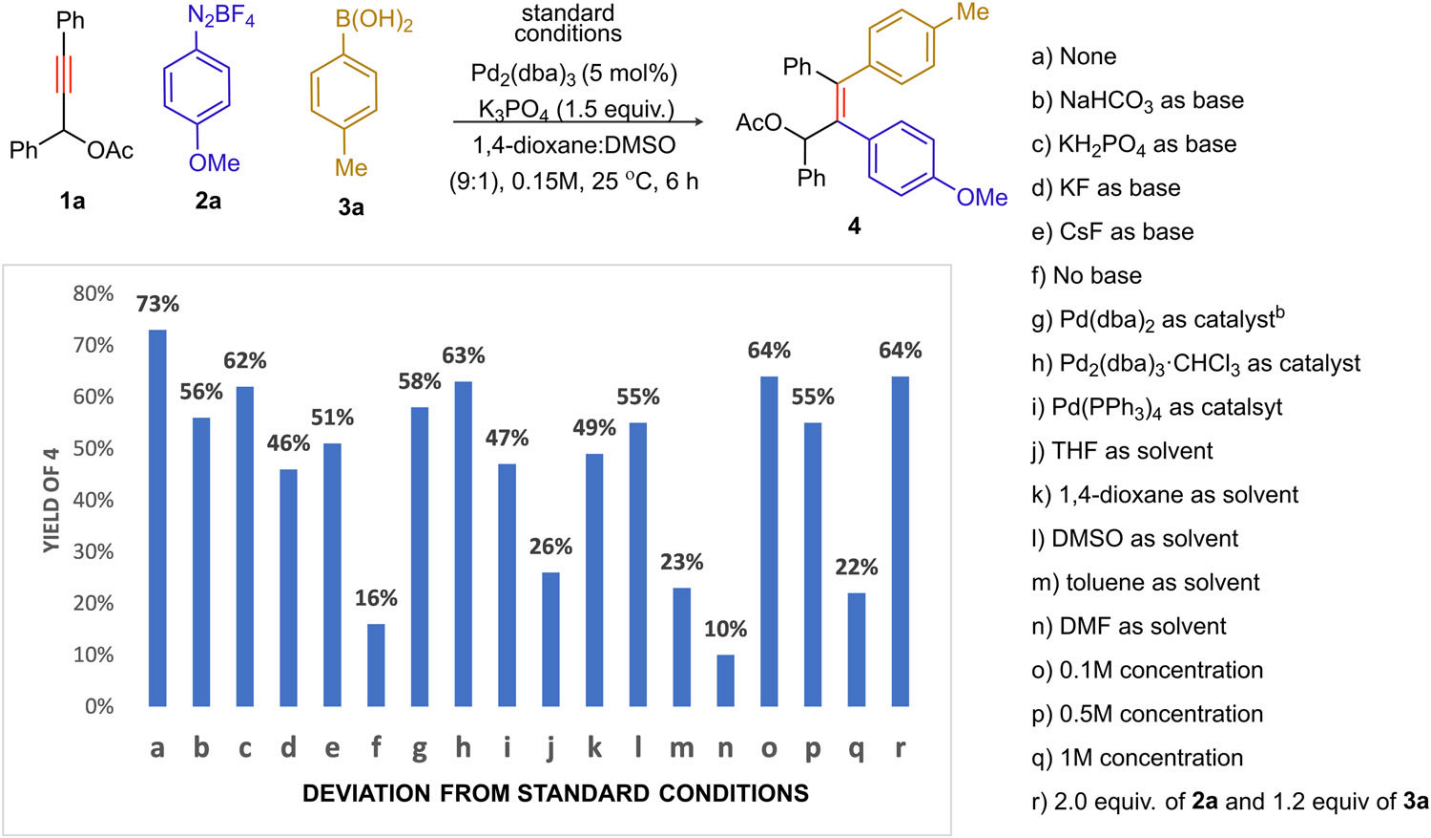
Optimization of the reaction conditions^a^. ^a^Isolated yield. 1a (0.2 mmol), 2a (0.6 mmol), 3a (0.3 mmol), cat. (0.01 mmol) and base (0.45 mmol). ^b^10 mol%.

To investigate the 1,2-diarylation of structurally simple yne-acetates [i.e. propargyl acetates (PAs) to start with], a three-component reaction of 1,3-diphenylprop-2-yn-1-yl acetate (**1a**), *p*–methoxyphenyl diazonium tetrafluoroborate (**2a**), and *p*–tolyl boronic acid (**3a**) in presence of Pd_2_(dba)_3_ as the catalyst and a base was performed (Fig. 2; see also Supplementary Table 3, SI). An extensive screening led to the optimized reaction conditions: [**1a** (1.0 equiv), **2a** (3.0 equiv), **3a** (1.5 equiv), Pd_2_(dba)_3_ (5.0 mol%) and K_3_PO_4_ (1.5 equiv) in 1,4-dioxane/DMSO (9:1) at 25 °C, 6 h]. With these, the unsymmetrical *syn*-diarylation product **4** was isolated in 73% yield (Fig. 2, **a**). NaHCO_3_, KH_2_PO_4_, KF and CsF proved to be less efficient bases (**b**–**e**). Compound **4** was isolated in 16% in the absence of base (**f**). Lower yields were obtained when other Pd(0) catalysts [Pd(dba)_2_, Pd_2_(dba)_3_^.^CHCl_3_, Pd(PPh_3_)_4_,] were used (**g**–**i**). The solvents THF, 1,4-dioxane, DMSO, toluene or DMF did not improve the outcome (**j**–**n**). The reaction concentration did influence the product yield **4** (**o**–**q**). The reaction was less efficient when **2a** (2.0 equiv) and **3a** (1.2 equiv) were used (**r**).

### Scope of unsymmetrical diarylation

**Fig. 3:**
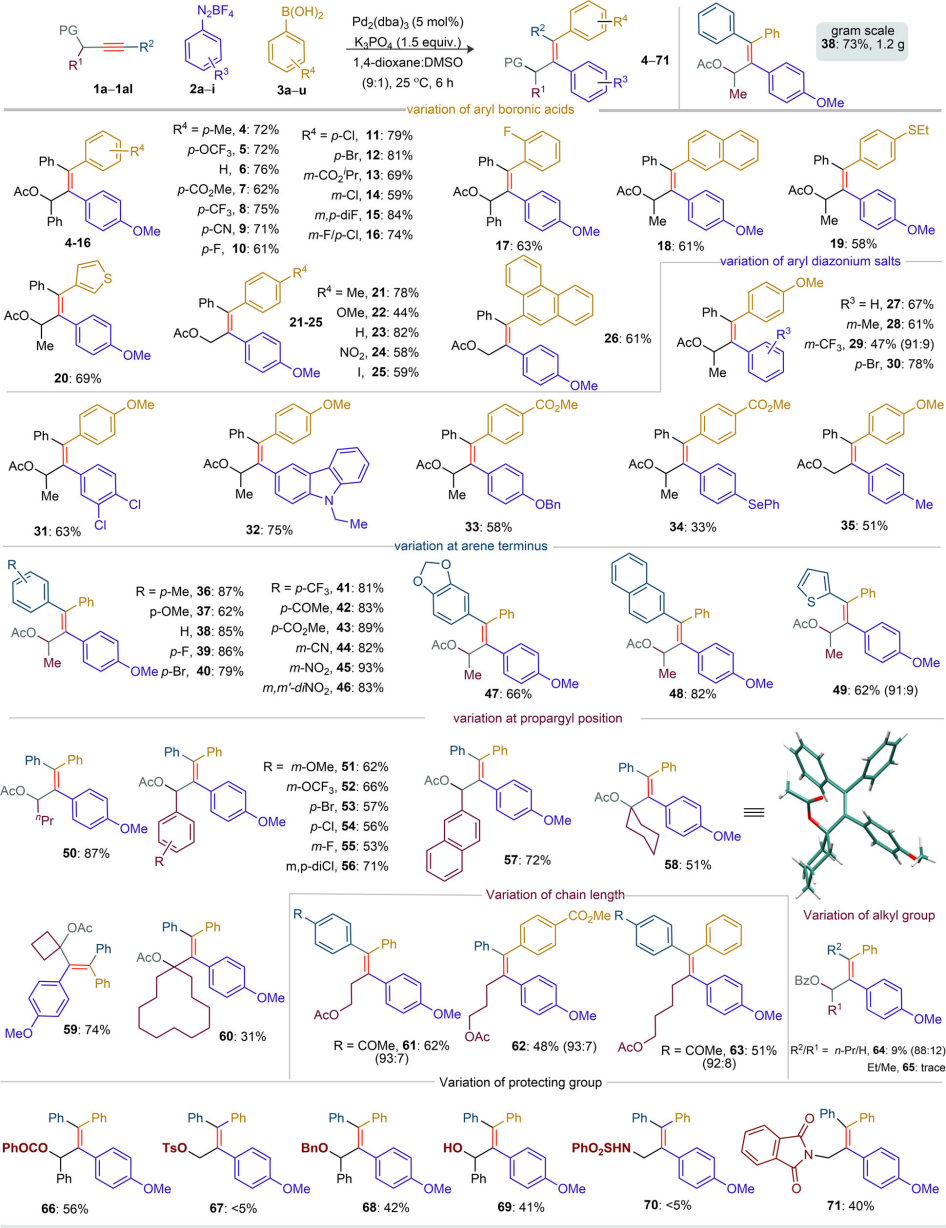
Scope of alkynes, aryl boronic acids, and aryl diazonium salts^a^. All yields correspond to products isolated as a single stereo- and regioisomer, unless otherwise stated. ^a^**1** (0.3 mmol), **2** (0.9 mmol), **3** (0.45 mmol). Ac acetyl and Bz benzyol.

**Fig. 4:**
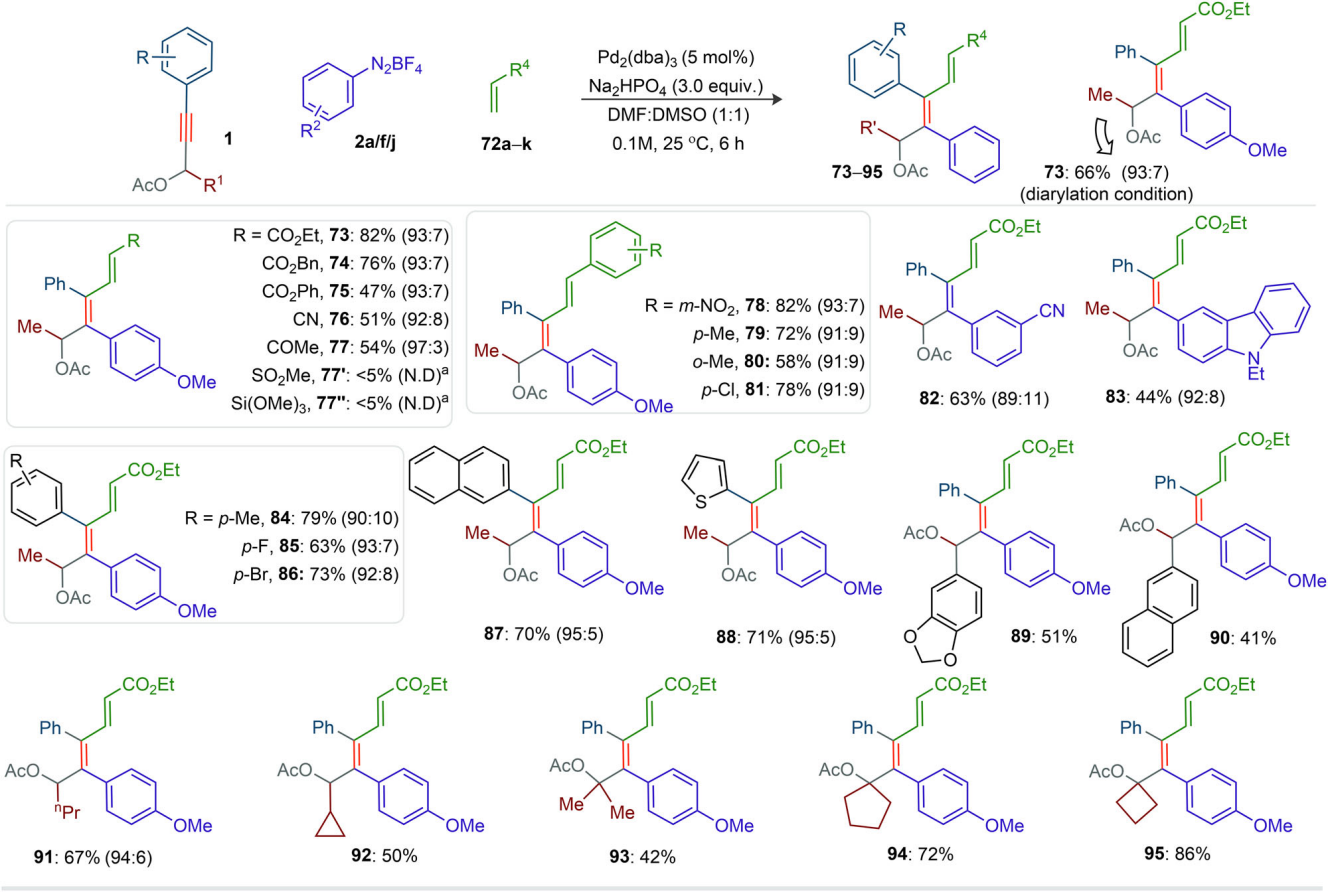
Scope of aryl-olefination of alkynes^a^. All yields correspond to products isolated as a single stereo- and regioisomer, unless otherwise stated. ^a^**1** (0.3 mmol), **2** (0.9 mmol), **72** (0.45 mmol). ^a^Hydroarylation product was observed (see **117**, Fig. 6d). ND not determined.

**Fig. 5:**
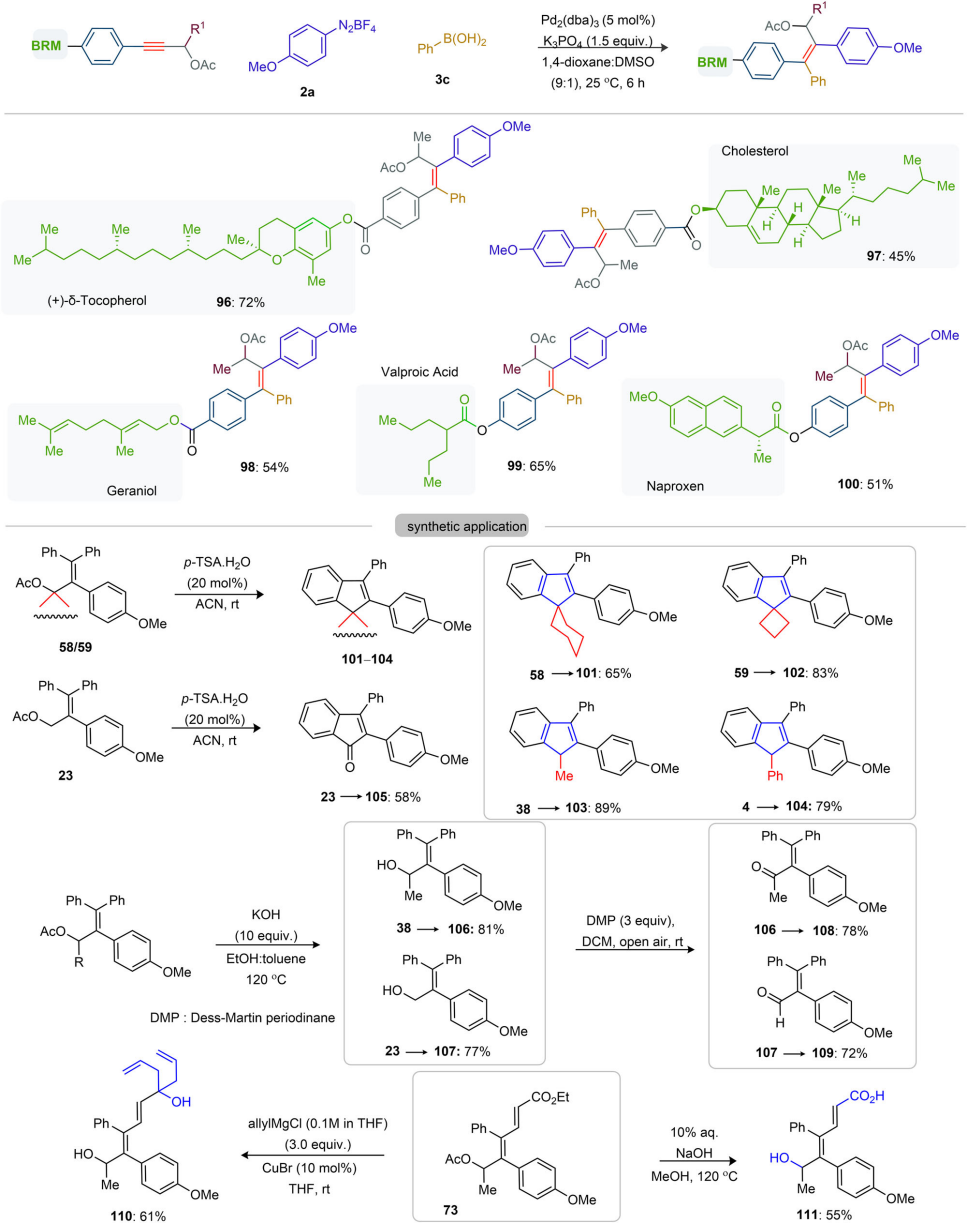
Scope of diarylation of biologically relevant motif coupled alkynes and synthetic applications. All reactions are carried out on a 0.2 mmol scale. All the compounds are isolated as a single isomer, unless otherwise stated. *P*-TSA *p*-tolylsulfonic acid, DMP Dess–Martin periodinane.

This breakthrough encouraged us to investigate the reaction scope (Figs. 3–5). The reactivity of the aryl boronic acid partners was first probed (Fig. 3). The reaction of electron-rich *p*-substituted aryl boronic acids [*p*-Me (**3a**), *p*-OCF_3_ (**3b**)] with **1a** and **2a** provided **4** and **5** in good yields. Likewise, the tetrasubstituted olefins **6**–**9** (62–76 %) were constructed from phenylboronic acid (**3c**) and electron-poor aryl boronic acids [*p*-CO_2_Me (**3d**), *p*-CF_3_ (**3e**), *p*-CN (**3f**)] when exposed to **1a** and **2a**. Although halo groups are usually amenable to cross-couplings under Pd(0)-catalysis, to our delight, the halogen-substituted aryl boronic acids [*p*-F (**3g**), *p*-Cl (**3h**), *p*-Br (**3i**)] proved compatible with the title reaction, leading to **10**–**12** in good yields. The transformation was compatible with *meta*- and *ortho*-substituted aryl boronic acids as well, leading to the densely functionalized tetrasubstituted olefins **13**–**17** (59–84%). The desired 2-naphthyl, 4-ethylthiophenyl, 3-thienyl-bearing allyl acetates **18**–**20** (58–69%) were also assembled from the methyl-substituted yne-acetate **1b**. The unsubstituted PA **1c** also proved to be a relevant substrate; various aryl boronic acids [*p*-Me (**3a**), *p*-OMe (**3r**), *p*-H (**3c**), *p*-NO_2_ (**3s**), and *p*-I (**3t**)] were coupled to provide **21**–**25**. The bulky 9-phenanthrene boronic acid was not an exception, providing the π-extended product **26** in 61% yield. Next, the three-component couplings of aryl diazonium tetrafluoroborates **2** with **1b** and **3r**/**3d** were surveyed. The reaction of **1b**, **3r** and diverse arene diazonium salts [phenyl (**2b**), electron-rich *m*-Me (**2c**), electron-poor *m*-CF_3_ (**2d**), *p*-Br (**2e**), and *m*,*p*-diCl (**2f**)] provided **27**–**31** in good yields. The allyl acetate **29** was isolated as regioisomeric mixture (91:9). A carbazole bearing diarylation product **32** was isolated in 75% yield. The OBn protecting group and the oxidizable SePh group were unaffected under this Pd-catalysis, giving access to **33** and **34**. Likewise, **35** (51%) was made from the reaction of **1c** with **2j** and **3r**. We next scrutinized the reactivity of unsymmetrical alkynes (Fig. 3). The reaction of PAs [having aryl motifs: *p*‐Me, *p*-OMe, *p*‐F, *p*-Br, *p*-CF_3_, *p*-COMe, *p*-CO_2_Me, *m*-CN, *m*‐NO_2_, *m,m*′-diNO_2_, and *m,p*‐methylenedioxy at the alkyne terminus] with **2a** and **3c** independently furnished the desired products **36**–**47** (62–93%). Likewise, the π-extended 2-naphthyl- and heteroaryl 2-thienyl-enabled tetrasubstituted olefins **48** and **49** were constructed. Irrespective of *n*-propyl and various aryl-moieties at the propargyl position of PAs, the diarylation was equally effective in making **50**–**57**. In general, sterically bulky substituents severely affect cross-couplings. Nevertheless, *syn*-diarylation of cyclohexyl and cyclobutyl tethered PAs with **2a** and **3c** provided all-carbon-substituted olefins **58** and **59**. X-ray analysis confirmed the structure of **58** (CCDC 2096145 contains the supplementary crystallographic data for compound 58). A macrocycle dodecane tethered diarylation product **60** was also fabricated. However, the terminal ynamide led to a complex mixture instead of providing the respective diarylation product. The product complexity justifies moderate yields (<50%); in such cases, the reaction was incomplete with recovery of unreacted PAs. To test the potential coordination ability of the carboxylate group, diarylation of yne-acetates with different tether lengths between the alkyne and the acetate functionalities was probed. Irrespective of the acetate position in yne-acetates, *syn*-diarylation of alkyne motifs was highly regioselective, furnishing **61**–**63** in moderate yields (Fig. 3). The diarylation of alkyl-substituted PAs **1ae**/**1af** independently with **2a** and **3c** under the optimized condition has led to **64 (**9%) and **65** (trace). In these cases, reaction was complex forming arylative dimerization of alkyne (confirmed by HRMS analysis) along with unreacted precursors (see the SI Supplementary Figs. 19 and 32). Next, the reaction of O-benzoate and O-benzyl protected propargyl alcohols with **2a** and **3c** afforded **66** (56%) and **68** (42%) respectively. On the other hand, the reaction of O-tosyl protected alkyne led to a complex mixture including a low amount of **67** (<5%); the low yield is possibly due to the facile cleavage of the labile C–OTs bond. The reaction of alkynes having unprotected hydroxy group or phthalimide group at the propargyl position with **2a** and **3c** delivered the desired diarylation products **69** and **71** in moderate yield with good regioselectivity. Unfortunately, the identical transformation of propargyl sulfonamide did not give the desired diarylation product **70**. The reaction is scalable to the gram, as shown by the preparation of **38** (1.2 g, 73 %) from the coupling of **1a** (1.0 g, 2.67 mmol), **2a** (1.77 g, 8.01 mmol) and **3c** (0.5 g, 4.00 mmol) using Pd_2_(dba)_3_ (3.0 mol %) (Fig. 3).

### Substrate scope of aryl-alkenylation

π-Conjugated scaffolds are widely found in molecules of pharmaceutical importance and light-emitting-diode materials^1^. We thus attempted to trap the cationic vinyl palladium species, obtained after aryl-palladation of the alkyne moiety, with olefins for constructing π-conjugated dienes (Fig. 4). As envisaged, the reaction of **1b**, **2a** and ethyl acrylate (**72a**) under the optimized conditions of entry 1, Fig. 2, successfully led to **73** in 66% yield. To enhance the reaction productivity, bases and solvents were further screened (Supplementary Table 4, SI). The Na_2_HPO_4_ base and DMSO:DMF (1:1) solvent combination were found optimum; **73** was isolated in 82% yield. Next, the reaction of wide ranges of acrylates and acrylonitriles with **1b** and **2a** under the adapted catalytic conditions furnished the desired conjugated dienes such as **73** (82%, 93:7), **74** (76%, 93:7), **75** (47%, 93:7) and **76** (51%, 92:8). Methyl-vinyl ketone is usually susceptible to polymerization^45^; despite this potential issue, **77** was isolated in 54% yield and 97:3 regioisomeric ratio. This difunctionalization even worked with styrenes, affording **78** (93:7), **79** (91:9), **80** (91:9), and **81** (91:9) in 58–82% yield. However, identical transformations with methyl-vinyl sulfone or vinyltrimethoxysilane did not provide the desired aryl-alkenylation products **77′** and **77″**. Rather substantial amount of hydroarylation product was observed along with unreacted yne-acetate. The products **82** (63%, 89:11) and **83** (44%, 92:8) were made from the couplings of **1b** and **72a** with **2f** and **2j**, respectively. The PAs [having aryl motifs: *p*-Me, *p*-F, *p*-Br, π-extended 2-naphthyl or 2-thienyl at the alkyne terminus] were independently coupled with **2a** and **72a** to deliver **84**–**88** in good yields along with 5–10% minor isomer. Likewise, **89**–**91** were made, albeit in moderate yield, from the reaction of PAs [with variation of substituents, *m,p*-methylenedioxy-phenyl, 2-naphthyl, *n*-Pr, and cyclopropyl at the propargyl position] with **2a** and **72a**. Even the sterically encumbered di-Me, cyclopentyl, and cyclobutyl tethered PAs were successfully converted into unusual π-conjugated dienes **92**–**95** (42–86%). Thus, the cationic Pd-catalytic system did not virtually affect the reaction outcome and the strained cyclopropyl ring, labile halo groups, and easily modifiable functional groups were well tolerated (Figs. 3 and 4).

### Scope of pharmacophore-coupled alkynes and synthetic applications

Late-stage difunctionalization of unsymmetrical alkynes having biologically relevant motifs (BRMs) is invaluable for the sustainable development of complex molecules with enhanced pharmacokinetic properties. However, BRMs with polar groups and unsaturated moieties often cause problems for cross-coupling reactions, as the TM binding ability could lead to substrate decomposition and affect the difunctionalization efficiency. We were therefore intrigued by the viability of the cationic Pd-catalyzed double arylation of pharmacophore-coupled alkynes **1** (Fig. 5). Once again, the title reaction proved reliable: tetrasubstituted allyl acetates encapsulated fatty alcohol [vitamin-E-tocopherol (**96**)], steroid [cholesterol (**97**)], and terpenoid [geraniol (**98**)] were produced in 45–72% yields from the diarylation of respective BRMs-coupled PAs with **2a** and **3c**. We further probed the efficacy of the regioselective diarylation using marketed drug molecules containing unsymmetrical alkynes. Thus, reaction of the anti-epileptic drug, bipolar disorder, and migraine preventer valproic acid coupled PA with **2a** and **3c** provided highly substituted allyl acetate **99** in 65% yield. Likewise, nonsteroidal anti-inflammatory drug containing allyl acetate **100** was made in good yield.

We next probed the synthetic versatility of the newly constructed tetrasubstituted allyl-acetates. The *p*‐TSA driven intramolecular Friedel-Crafts arene cyclization of **58**/**59** led to unusual cyclohexyl/cyclobutyl spiro-fused indene derivatives **101** (65%) and **102** (83%), respectively, and peripheral-substituted indenes **38**${{\to }}$**103** (89%) and **4**${{\to }}$**104** (79%). Likewise, electrophilic cyclization of **23** provided indanone **105** in 58% yield. Fully substituted propargyl alcohols [**38**$\to$**106** (81%); **23**${{\to }}$1**07** (77%)] were accessed from the KOH mediated hydrolysis of the acetate motif. Dess–Martin periodinane (DMP)-mediated oxidation of **106** and **107** delivered methyl-vinyl ketone **108** (78%) and acrolein **109** (72%), respectively; further functionalization of carbonyl groups is therefore possible. Allylation and hydrolysis of π-extended ester **73** yielded allylic-3°-alcohol **110** and α,β-unsaturated carboxylic acid **111**.

### Mechanistic studies

**Fig. 6:**
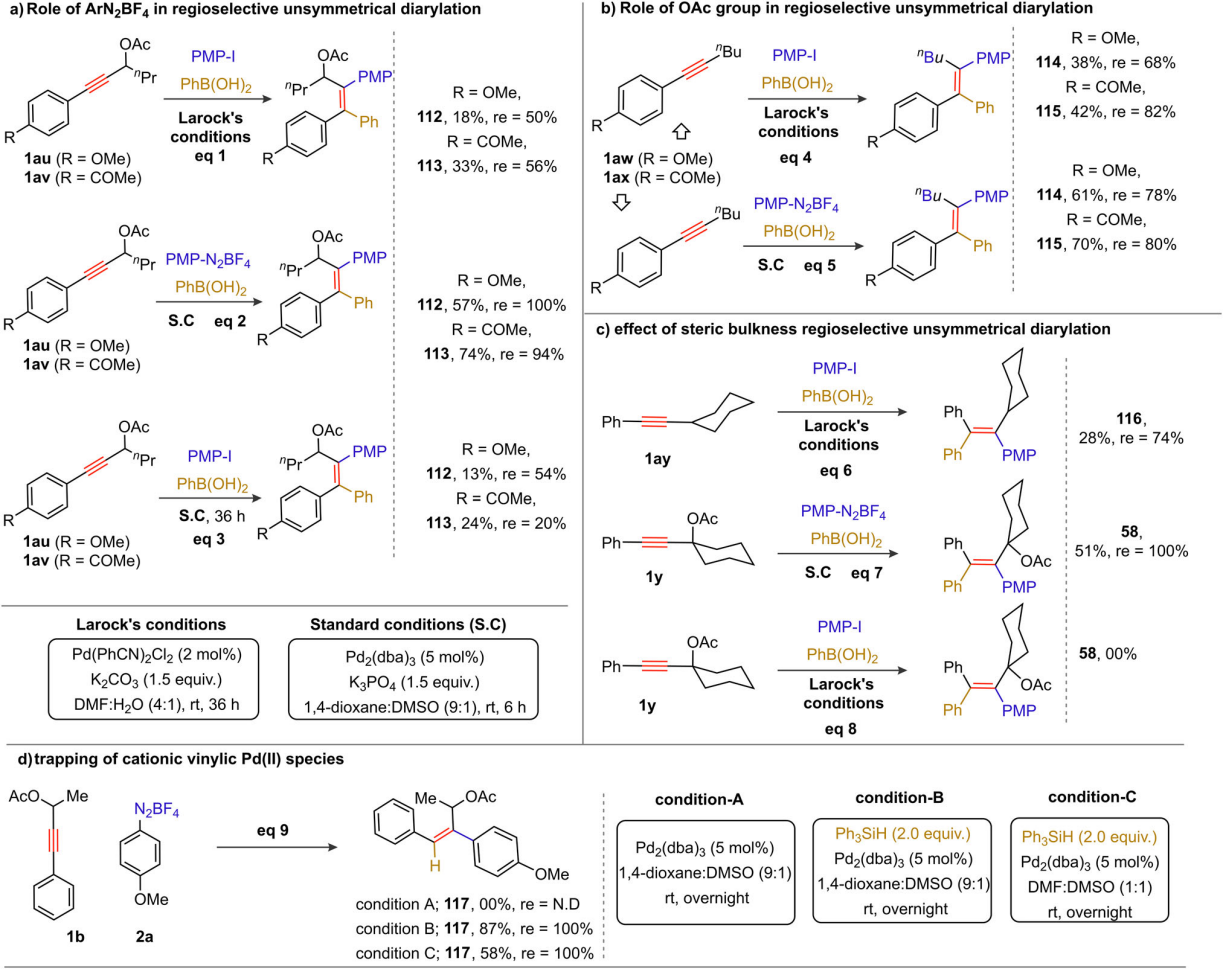
Mechanistic studies. **a** Three comparative reactions were performed with electronically diverse propargyl acetates under the {Larock’s reaction conditions: eq 1 [PMP-I, PhB(OH)_2_, Pd(PhCN)_2_Cl_2_, K_2_CO_3_, DMF:H_2_O (4:1), rt]}, {standard conditions (SC) of current reaction: eq 2 [PMP-N_2_BF_4_, PhB(OH)_2_, Pd_2_(dba)_3_, K_3_PO_4_, 1,4-dioxane:DMSO (9:1), rt]}, and {SC of current reaction: eq 3 [PMP-I instead of PMP-N_2_BF_4_]}. Excellent regioselectivity has been observed in eq 2; thus, PMP-N_2_BF_4_ and cationic palladium species are pivotal. **b** Two comparative reactions were performed with electronically diverse aryl-butyl alkynes using Larock’s reaction conditions (eq 4) and the SC of current reaction (eq 5). **c** Two comparative reactions were independently performed with sterically hindered Ph-cyclohexyl alkyne and cyclohexyl tethered PA under the Larock’s reaction conditions (eqs. 6 and 8) and SC current reaction (eq. 7), respectively. **d** Trapping of cationic vinylic Pd(II) species in the absence of aryl boronic acid. Note: inseparable regioisomers are isolated as mixture and the regioisomeric excess (re) is determined by ^1^H NMR. PMP *p*-methoxyphenyl.

To shed light on the reaction mechanism and importance of ArN_2_BF_4_, cationic palladium species, and acetate (OAc) group for the notable regioselectivity outcome in the 1,2-diarylation of PAs, a series of experiments was planned (Fig. 6a–d). The reaction of electronically diverse PAs (having aryl-moiety *p*-OMe (**1au**)/*p*-COMe (**1av**) at the alkyne terminus) were independently subjected to different reaction conditions: {Larock’s conditions: eq 1 [*p*-methoxyphenyl (PMP)-I, PhB(OH)_2_, Pd(PhCN)_2_Cl_2_, K_2_CO_3_, DMF:H_2_O (4:1), rt]}, {standard conditions (SC) of current reaction: eq 2 [PMP-N_2_BF_4_, PhB(OH)_2_, Pd_2_(dba)_3_, K_3_PO_4_, 1,4-dioxane:DMSO (9:1), rt]}, and {SC of current reaction: eq 3 [PMP-I instead PMP-N_2_BF_4_]}. As anticipated, the diarylation products **112** (57%) and **113** (74%) with regioisomeric excess (*re*) 100% and 94%, respectively, were formed (eq 2, Fig. 6a). On the other hand, the identical reaction under Larock’s conditions provided poor productivity as well as low *re* **112** (11%, 50% *re*) and **113** (21%, 56% *re*) (eq 1, Fig. 6a). In comparison, **112** (13%, 54% *re*) and **113** (24%, 20% *re*) were observed when the reaction was performed with PMP-I instead PMP-N_2_BF_4_ under the SC (eq 3, Fig. 6a). It appears that cationic palladium species [obtained in situ by the oxidative addition of PMP-N_2_BF_4_ with Pd(0) catalyst] play a crucial role in the control of the regioselectivity as well as productivity. Likewise, 1,2-diarylation of *p*-MeO-C_6_H_4_ (**1aw**)/*p*-MeOC-C_6_H_4_ (**1ax**)-^*n*^butyl alkynes with PMP-I/ PMP-N_2_BF_4_ along with PhB(OH)_2_ independently led to [**114** (38%, 68% *re*) and **115** (42%, 82% *re*) under Larock’s conditions, shown in eq 4, Fig. 6b] and [**114** (61%, 78% *re*) and **115** (70%, 80% *re*) under SC, shown in eq 5, Fig. 6b]. Thus, **114** (~70% *re*) and **115** (~80% *re*) with regioisomeric products mixture obtained in the diarylation of **1ar**/**1as** (with no carboxylate moiety) irrespective of Larock/SC suggests that alkynes with electronic variation marginally affect both selectivity and productivity. On the other hand, diarylation of yne-acetates occurs with excellent regioselectivity (eq 2, Fig. 6a); notably, the electronic properties of functional groups did virtually not show any impact.

To further validate the importance of the acetate group, sterically hindered phenyl cyclohexyl alkyne **1ay** was subjected to PMP-I, and PhB(OH)_2_ under Larock’s reaction conditions (eq. 6, Fig. 6c); the respective product **116** (28%, 74% *re*) was isolated in low yield and moderate regioselectivity (eq 6, Fig. 6c). By contrast, reaction of **1y**, exhibiting an acetate group at the propargyl position of **1ay**, with PMP-N_2_BF_4_ and PhB(OH)_2_ under the SC furnished **58** (51%) in 100% *re* (eq 7, Fig. 6c). Moreover, **1y** failed to give the desired diarylation product **58** under Larock condition, thus, cationic Pd species is essential (eq 8, Fig. 6c). From the mechanistic discussions highlighted in eqs 1–8, Fig. 6, we conclude that the outcome and regioselectivity of *syn*-1,2-diarylation of unsymmetrical alkynes are strongly influenced by the carboxylate protecting group and the in situ formation of a stabilized cationic Pd intermediate.

### DFT calculations

**Fig. 7:**
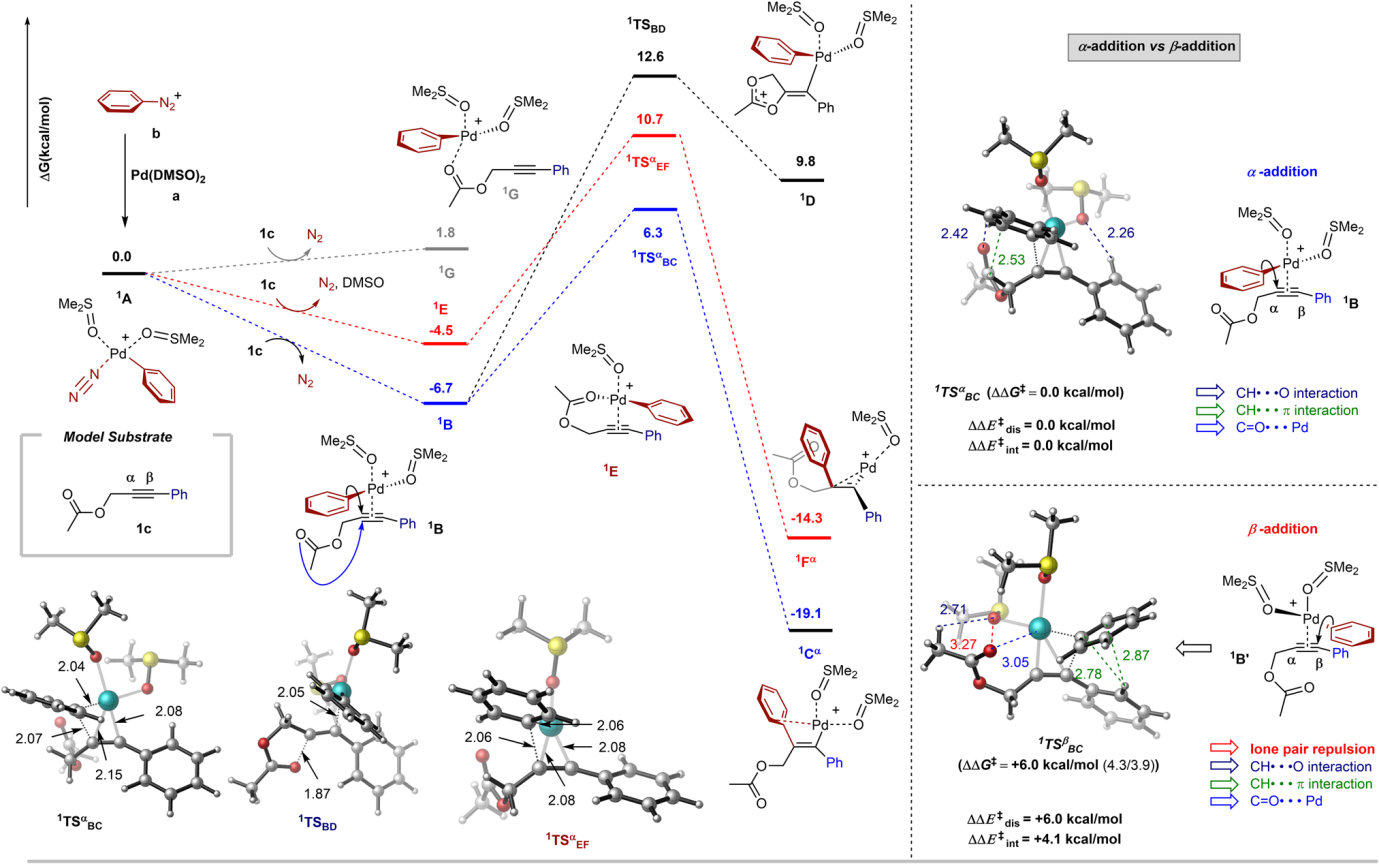
DFT calculations (atom colors: blue = Pd; yellow = S; red = O; gray = C; white = H).

To gain insight into the reaction mechanism and notably the stereo/regioselectivity of the 1,2-diarylation of PAs, DFT calculations were performed at the M06L/def2-TZVPP(SMD)//BP86/LANL2DZ(Pd)_6-31G(d,p) level of theory (Fig. 7, see also the SI for the cross-coupling part, the case of substrates with longer tethers and the case of an unreactive methylalkyne). The transformation begins with the barrierless oxidative addition of Pd(DMSO)_2_ (**a**) to the phenyl diazonium salt **b** to provide the cationic Pd-complex ^**1**^**A**. ^6a^ Next, coordination of ^**1**^**A** to PA **1c** is possible by substitution of N_2_. However, this process could happen in three different ways, via (i) the coordination of the C≡C bond of **1c** to form complex ^**1**^**B** by releasing 6.7 kcal/mol of free energy (Fig. 7; blue) (ii) the coordination of both the C≡C bond and the ester group in **1c** to provide ^**1**^**E** with the release of 4.5 kcal/mol of free energy (Fig. 7; red), and (iii) the coordination of the ester group in **1c** to generate ^**1**^**G**, this step being endergonic by 1.8 kcal/mol (Fig. 7; gray). Thus, the ester group participation for the replacement of N_2_ in ^**1**^**A** is not necessary. Next, a suprafacial α-aryl migration from Pd to the C≡C bond of ^**1**^**B** (*syn*-insertion) proceeds through transition state ^**1**^**TS**^**α**^_**BC**_, found at 6.3 kcal/mol on the free energy surface, and results in the Pd- alkenyl ester complex ^**1**^**C**^**α**^. This complex lies at –19.1 kcal/mol on the free energy surface with *trans*-relationship of two phenyl groups. Intramolecular neighboring group participation of the ester group at C^α^ of ^**1**^**B** can provide the Pd-alkenyl heterocyclic complex ^**1**^**D** through ^**1**^**TS**_**BD**_ (12.6 kcal/mol); however, this process needs an additional 6.3 kcal/mol activation energy and is thus ruled out.

Alternatively, α-aryl migration of ester chelate ^**1**^**E** forms intermediate ^**1**^**F**^**α**^ (–14.3 kcal/mol) through ^**1**^**TS**^**α**^_**EF**_ (10.7 kcal/mol). The energy barrier is 4.4 kcal/mol higher than ^**1**^**TS**^**α**^_**BC**_; this pathway is thus not preferred. A detailed comparison of all the options justifies the feasibility of the bottom pathway ^**1**^**B**${{\to }}$^**1**^**C**^**α**^ (marked in blue). Like normal Suzuki reactions, transmetalation of ^**1**^**C** with aryl boronic acid followed by reductive elimination gives the final diarylation product (Supplementary Scheme 9). We have studied various tether lengths of the yne-acetate and found that the C^α^-arylation process is always favored over the C^α^-arylation (Supplementary Schemes 10–15). To rationalize this selectivity, a distortion/interaction and non-covalent interactions analysis of the aryl migration transition states ^**1**^**TS**^**α**^_**BC**_ and ^**1**^**TS**^**β**^_**BC**_ (that includes substrate fragment and aryl-palladium fragment) was performed (see right side in Fig. 7 and Supplementary Figs. 14–16). The large rotation angle for β-aryl migration (see the SI: 36.12° for ^**1**^**TS**^**β**^_**BC**_ and 7.34° for ^**1**^**TS**^**α**^_**BC**_) contributes to excess distortion energy [+6.0 kcal/mol; that includes both aryl - palladium (+3.68 kcal/mol) and substrate (+2.32 kcal/mol) distortion]. In addition, a large level of non-covalent lone pair repulsion of the carboxylate moiety with ligated DMSO for ^**1**^**TS**^**β**^_**BC**_ (+4.1 kcal/mol) relative to ^**1**^**TS**^**α**^_**BC**_ was detected.

In summary, a regio- and stereoselective *syn*-1,2-dicarbofunctionalization of unsymmetrical alkynes involving structurally distinct carbon functionalities has been developed. The cationic Pd^II^‐catalyst plays an essential role in modulating the regioselective insertion of aryl‐diazonium salts and boronic acids/olefins to the unsymmetrical alkynes. The transformation proceeds at room temperature and tolerates oxidizable halo‐species (I/Br), easily transformable functionalities (CO_2_Me, CN) and strained rings, thus opening a broad chemical space [94 examples]. It is even successful on the gram scale. Carboxylate protecting group and the in situ formation of a stabilized cationic Pd intermediate solely responsible for the outcome and regioselectivity of *syn*-1,2-diarylation of unsymmetrical alkynes. DFT studies rationalize the α-arylation preference over β-arylation of PAs and discard direct participation of the acetate as a DG. The highly substituted olefins are subsequently used for the construction of functionalized indene, methyl-vinyl ketone, and acrolein skeletons. The current finding paves the way to the discovery of unknown difunctionalization strategies of unactivated alkynes.

## Methods

### General procedure for the *syn*-1,2-difunctionalization reactions

To a mixture of alkyne **1** (0.3 mmol), aryl diazonium tetrafluoroborate **2** (0.9 mmol), aryl boronic acid **3** or olefin **72** (0.45 mmol), Pd catalyst (0.015 mmol) and base (0.45 mmol) was added the respective solvent. The resulting reaction mixture was stirred at 25 °C for 6 h. The reaction progress was periodically monitored by TLC. The solvent was next removed either by water workup or by evaporation under reduced pressure. The organic layer was extracted in ethyl acetate (3 × 10 mL) and dried over Na_2_SO_4_. The organic layer was evaporated and purified by column chromatography over neutral alumina to afford **4**–**100** and **112**–**113**. The compounds are sensitive to acidic silica gel and thus, final product purification was carried out on neutral alumina.

## Supplementary information

Supplementary Information

## Data Availability

Data relating to the characterization of materials and products, general methods, optimization studies, experimental procedures, mechanistic studies and NMR spectra are available in the Supplementary Information. Crystallographic data for compound **58** is available free of charge from the Cambridge Crystallographic Data Centre under reference number 2096145.

## Code availability

No code was developed, the commercially available Gaussian software has been used.
