# Supplementary material for: Cationic-palladium catalyzed regio- and stereoselective syn-1,2-dicarbofunctionalization of unsymmetrical internal alkynes
Source: Nat Commun. 2022 Mar 16;13:1360. doi: 10.1038/s41467-022-28949-7 (PMC8927424; doi:10.1038/s41467-022-28949-7)
Supplement: Supplementary file 1 — Supplementary Information [file 41467_2022_28949_MOESM1_ESM.pdf]

Cationic-Palladium Catalyzed Regio- and Stereoselective *syn*-1,2- Dicarbofunctionalization of Unsymmetrical Internal Alkynes

Shubham Dutta,[a] Shashank,[a] Shengwen Yang,[b] Manash Protim Gogoi,[a] Vincent Gandon,[bc]* and Akhila K. Sahoo[a]*

[a]School of Chemistry, University of Hyderabad, Hyderabad 500046, India

[b]Institut de Chimie Moléculaire et des Matériaux d'Orsay (ICMMO), CNRS UMR 8182, Université Paris-Saclay, Bâtiment 420, 91405 Orsay cedex, France.

[c]Laboratoire de Chimie Moléculaire (LCM), CNRS UMR 9168, Ecole Polytechnique, Université Paris-Saclay, route de Saclay, 91128 Palaiseau cedex, France

E-mail: vincent.gandon@universite-paris-saclay.fr

E-mail: akhilchemistry12@gmail.com; akssc@uohyd.ac.in

SUPPORTING INFORMATION

**Table of Contents**	**Page**
Supplementary Note	S2
Supplementary methods	S3
Supplementary Discussion (mechanistic studies)	S51
DFT Computations	S62
X-ray crystallographic data	S166
NMR data	S168
Supplementary reference	S286

1. Supplementary Notes

All the reactions were performed in oven-dried sealed tubes. Commercial grade solvents were distilled prior to use. Column chromatography was performed using either 100-200 Mesh or 230-400 Mesh silica gel or neutral alumina. Thin layer chromatography (TLC) was performed on silica gel GF254 plates and alumina plates.

Proton, carbon, and fluorine nuclear magnetic resonance spectra (^{1}H NMR, ^{13}C NMR, and ^{19}F NMR) were recorded based on the resonating frequencies as follows:(^{1}H NMR, 400 MHz; ^{13}C NMR, 101 MHz; ^{19}F NMR, 376 MHz), (^{1}H NMR, 500 MHz; ^{13}C NMR, 126 MHz; ^{19}F NMR, 470 MHz) and (^{1}H NMR, 600 MHz; ^{13}C NMR, 151 MHz) having the solvent resonance as internal standard (^{1}H NMR, $CDCl_3$ at 7.26 ppm; ^{13}C NMR, $CDCl_3$ at 77.0 ppm). Few cases tetramethylsilane (TMS) at 0.00 ppm was used as reference standard. Data for ^{1}H NMR are reported as follows: chemical shift (ppm), multiplicity (s = singlet; br s = broad singlet; d = doublet; br d = broad doublet, t = triplet; br t = broad triplet; q = quartet; m = multiplet; tt = triplet of triplet; dt = doublet of triplet), coupling constant, *J*, in (Hz), and integration. Data for ^{13}C NMR, ^{19}F NMR were reported in terms of chemical shift (ppm). IR spectra were reported in cm^{-1}. High resolution mass spectra were obtained in ESI mode. Melting points were determined by electro-thermal heating and are uncorrected. X-ray data was collected at 293 K using graphite monochromated Mo-Kα radiation (0.71073 Å).

1.1 Materials: Unless otherwise noted, all the reagents and intermediates were obtained commercially and used without purification. Toluene and 1,4-dioxane were distilled over CaH_2. $Pd_2(dba)_3$ and aryl boronic acids were purchased from commercially available source, and used as received. Analytical and spectral data of all the known compounds are exactly matching with the reported values.

Following the known procedure, the yne-acetates **(1a–1av)** [1-2] were prepared (Table S1). Analytical and spectral data of these compounds except **1ak**–**1ao** are reported and exactly matching with the reported values.

2. Supplementary Methods

2.1 General procedure for the synthesis of propargyl alcohols (GP 1):[1-2]

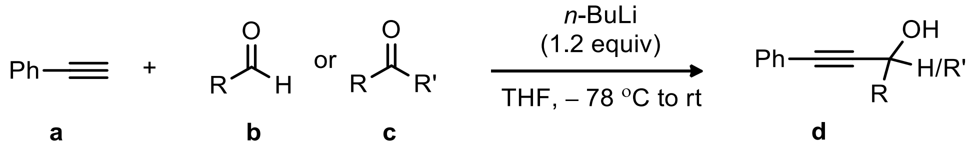

To a solution of alkyne **a** (39 mmol) in anhydrous THF (50 mL) at –78 °C under N_2 atmosphere was added *n*-BuLi (1.6 M solution in hexanes, 20.6 mL, 33 mmol). The reaction was stirred at this temperature for 20 min, and then at room temperature for 1 h. After cooling to –78 °C, aldehyde (30 mmol) was added to the mixture. The reaction mixture was warmed to room temperature gradually, and was stirred for an additional hour before quenching with saturated aqueous NH_4Cl. The mixture was extracted with EtOAc (3 × 20 mL), and the combined organic extracts were washed with water and brine, dried over anhydrous Na_2SO_4. The solvent was then filtered and concentrated under reduced pressure. The residue was purified by column chromatography on silica gel eluting with hexane/EtOAc to give the desired propargyl alcohol **d**.

2.2 General procedure for the synthesis of yne-ols (GP 2):[3]

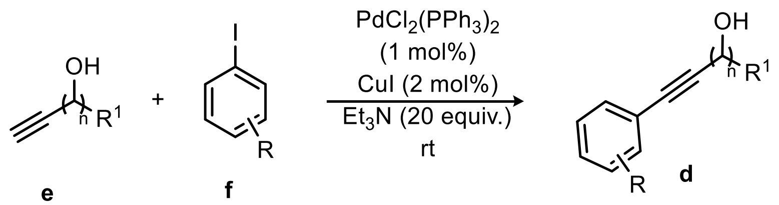

To a solution of $PdCl_2(PPh_3)_2$ (0.02 mmol) and CuI (0.04 mmol) in Et_3N (20 equiv) were added aryl iodide (2.6 mmol) and substrate **e** (2.0 mmol) successively under an argon atmosphere. The resulting mixture was stirred at room temperature overnight. The crude reaction mixture was filtered through a small pad of Celite and concentrated under the reduced pressure. The crude residue was purified using column chromatography on silica gel to afford the respective ynols **d**.

2.3 General procedure for acetyl protection of –OH group (GP 3):[1,2]

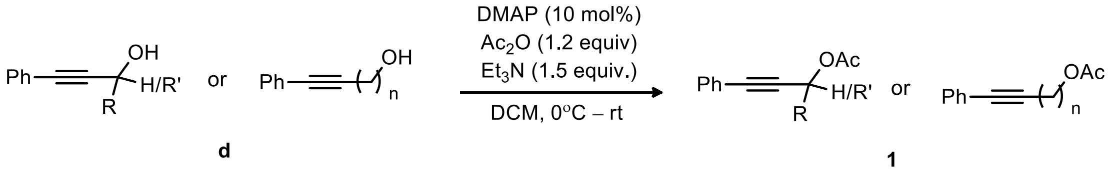

To a solution of an alcohol (**d**, 1.0 equiv), Et_3N (1.5 equiv) and DMAP (0.1 equiv) in anhydrous CH_2Cl_2 (1M) was added acetic anhydride (1.2 equiv) at 0 °C. The mixture was stirred at room temperature for 2–3

h. Upon complete consumption of alcohol (monitored by TLC), the mixture was concerted under reduced pressure and purified by column chromatography on silica gel eluting with hexane/EtOAc to give yne-acetates **1**.

2.4 Procedure-A for benzoyl protection of –OH group:[4]

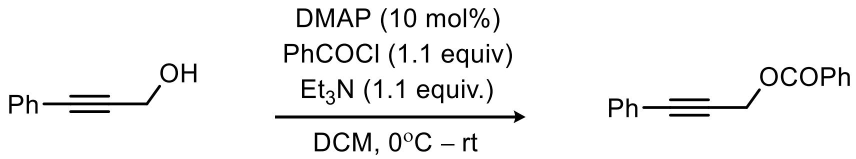

d' **1ag**

To a solution of a propargyl alcohol **d′** (1.0 equiv), Et_3N (1.1 equiv) and DMAP (0.1 equiv) in anhydrous CH_2Cl_2 (0.5M) was added benzoyl chloride (1.1 equiv) at 0 °C. The mixture was stirred at room temperature for 6 h. Upon complete consumption of alcohol (monitored by TLC), the mixture was concerted under reduced pressure and purified by column chromatography on silica gel eluting with hexane/EtOAc to give propargyl benzoate **1ag**.

2.5 Procedure-B for benzoyl protection of –OH group:[4]

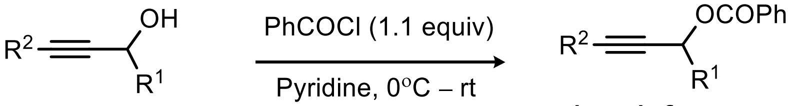

d" **1ae–1af**

To a solution of a propargyl alcohol **d′′** (1.0 equiv) in pyridine (0.3M) was added benzoyl chloride (1.1 equiv) at 0 °C. The mixture was stirred at room temperature for 6 h. Upon complete consumption of alcohol (monitored by TLC), the mixture was quenched with 1N aq. HCl and extracted with CH_2Cl_2 (3 × 10 mL). The combined organic layer was concerted under reduced pressure and purified by column chromatography on silica gel eluting with hexane/EtOAc to give propargyl benzoate **1ae-1af**.

2.6 Procedure for tosyl protection of –OH group:[5]

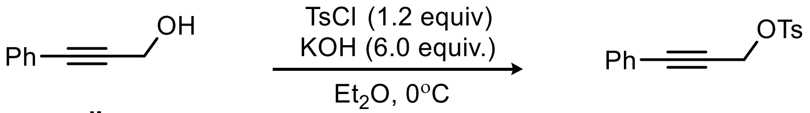

d' **1ah**

To a solution of a propargyl alcohol **d′** (1.0 equiv), crushed KOH (6.0 equiv) in anhydrous Et_2O (0.7M) was added TsCl (1.2 equiv) at 0 °C. The mixture was stirred at same temperature for 1 h. Upon complete consumption of alcohol (monitored by TLC), the mixture was concerted under reduced pressure and

purified by column chromatography on silica gel eluting with hexane/EtOAc to give propargyl tosylate **1ah**.

2.7 Procedure for benzyl protection:

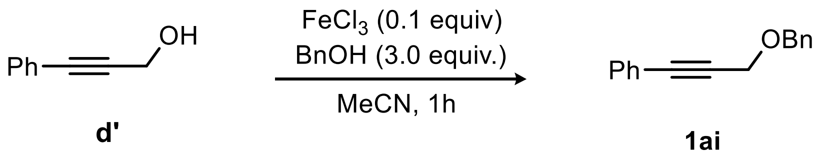

To a solution of a propargyl alcohol (1.0 equiv), $FeCl_3$ (0.1 equiv) in anhydrous MeCN (0.5M) was added BnOH (3.0 equiv). The mixture was stirred at room temperature for 1 h. Upon complete consumption of alcohol (monitored by TLC), the mixture was concerted under reduced pressure and purified by column chromatography on silica gel eluting with hexane/EtOAc to give propargyl benzyl ether **1ai**.

2.8 General procedure for synthesis of biological relavant motif bearing propargyl acetates (1ap, 1aq, and 1ar):

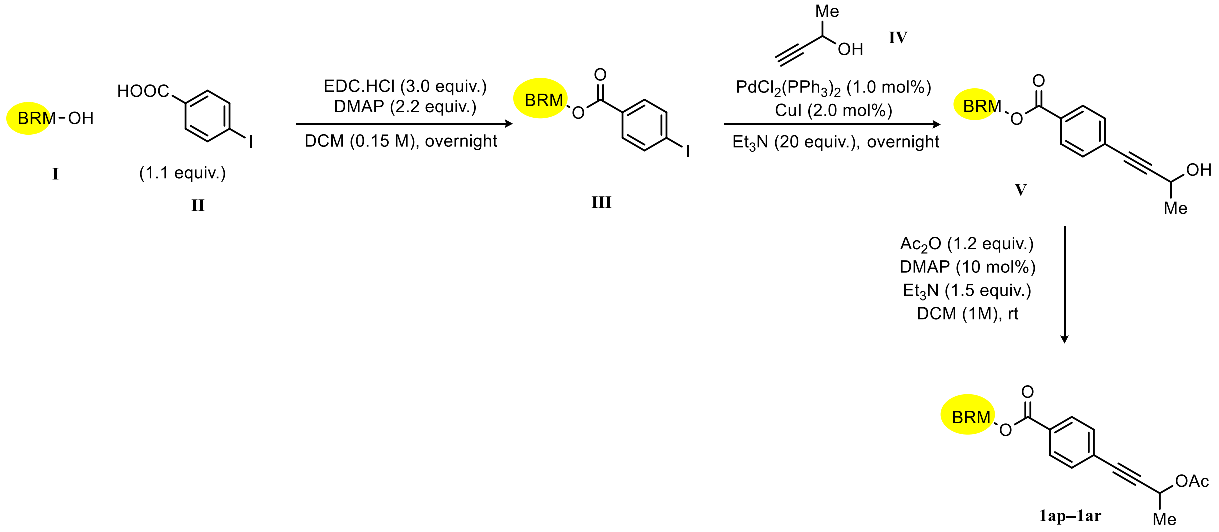

A solution of N'-(3-dimethylaminopropyl)-N-ethylcarbodimide, hydrochloride salt (EDC·HCl) (3.0 equiv), 4-N,N-dimethylaminopyridine (DMAP) (2.2 equiv) and 4-iodobenzoic acids (**II**) (1.1 equiv) in CH_2Cl_2 (0.15M) was stirred under an argon atmosphere. Alcohol (**I**; 1.0 equiv) was introduced dropwise (portion wise if the alcohol is solid) at 0 °C. The resulting reaction mixture was stirred for about 1 h at 0 °C and then 12 h at ambient temperature. Upon complete consumption of alcohol, the solvent was filtered and evaporated under reduced pressure. The crude residue was purified using column chromatography on silica gel using hexane/ethyl acetate to afford **III**.

To a solution of $PdCl_2(PPh_3)_2$ (0.02 mmol) and CuI (0.04 mmol) in Et_3N (20 equiv) were added aryl iodide **III** (2.6 mmol) and substrate **IV** (2.0 mmol) successively under an argon atmosphere. The resulting mixture was stirred at room temperature overnight. The crude reaction mixture was filtered through a small pad of Celite and concentrated under reduced pressure. The crude residue was purified using column chromatography on silica gel to afford the respective ynols **V**.

To a solution of an alcohol (**V**, 1.0 equiv), Et_3N (1.5 equiv) and DMAP (0.1 equiv) in anhydrous CH_2Cl_2 (1M) was added acetic anhydride (1.2 equiv) at 0 °C. The mixture was stirred at room temperature for 2–3 h. Upon complete consumption of alcohol (monitored by TLC), the mixture was concerted under reduced pressure and purified by column chromatography on silica gel eluting with hexane/EtOAc to give yne-acetates **1ap–1ar**.

2.9 General procedure for the synthesis of biological relavant motif bearing propargyl acetates (1as and 1at):

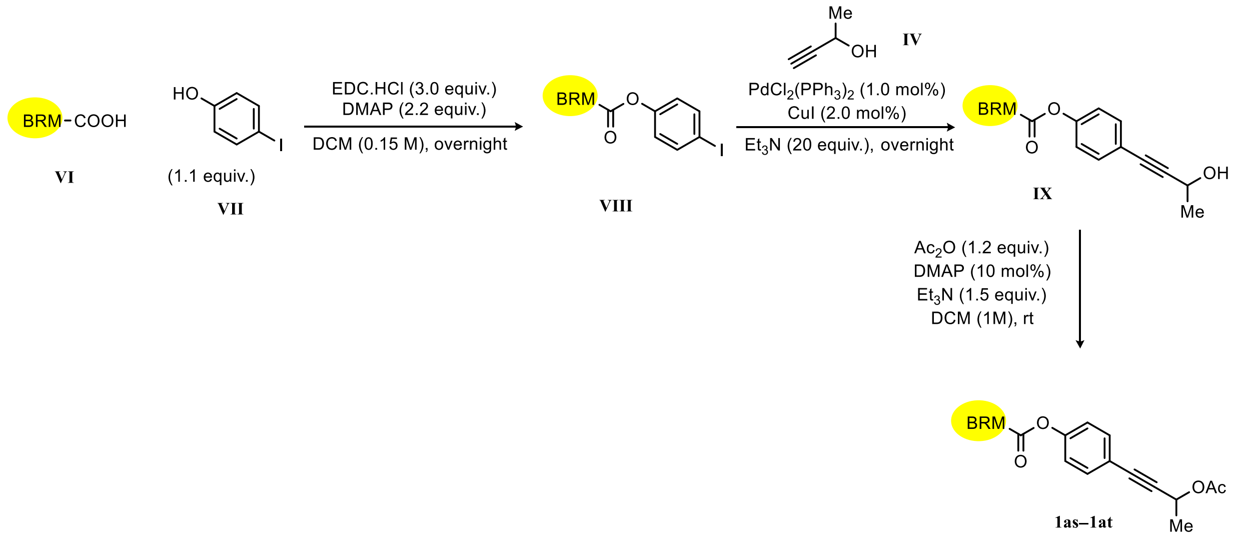

A solution of N'-(3-dimethylaminopropyl)-N-ethylcarbodimide, hydrochloride salt (EDC·HCl) (3.0 equiv), 4-N,N-dimethylaminopyridine (DMAP) (2.2 equiv) and carboxylic acids (**VI**) (1.1 equiv) in CH_2Cl_2 (0.15M) was stirred under an argon atmosphere. Next, 4-iodo phenol (**VII**) (1.0 equiv) was introduced portionwise at 0 °C. The resulting reaction mixture was stirred for about 1 h at 0 °C and then 12 h at ambient temperature. Upon complete consumption of carboxylic acid, the solvent was filtered and evaporated under reduced pressure. The crude residue was purified using column chromatography on silica gel using hexane/ethyl acetate to afford **VIII**.

To a solution of $PdCl_2(PPh_3)_2$ (0.02 mmol) and CuI (0.04 mmol) in Et_3N (20 equiv) were added aryl iodide **VIII** (2.6 mmol) and substrate **IV** (2.0 mmol) successively under an argon atmosphere. The resulting mixture was stirred at room temperature overnight. The crude reaction mixture was filtered through a small pad of Celite and concentrated under reduced pressure. The crude residue was purified using column chromatography on silica gel to afford the respective ynols **IX**.

To a solution of an alcohol (**IX**, 1.0 equiv), Et_3N (1.5 equiv) and DMAP (0.1 equiv) in anhydrous CH_2Cl_2 (1M) was added acetic anhydride (1.2 equiv) at 0 °C. The mixture was stirred at room temperature for 2–3 h. Upon complete consumption of alcohol (monitored by TLC), the mixture was concerted under reduced pressure and purified by column chromatography on silica gel eluting with hexane/EtOAc to give yne-acetates **1as–1at**.

2.10 General procedure for the preparation of aryl diazonium salts (2a–j):[5]

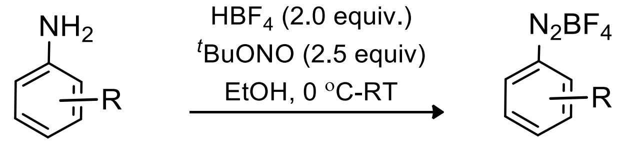

1''' **2**

To the solution of HBF_4 (1.3 mL) and **1'''** (5.0 mmol) in EtOH (5.0 mL) was added ^{t}BuONO (1.4 mL) dropwise at 0 °C. The reaction mixture was stirred for 30 min at room temperature. Upon reaction completion, Et_2O (20 mL) was added to precipitate the diazonium salt. Next, the crude reaction mixture was filtered and the residue was washed with Et_2O (3 × 10 mL).

Supplementary Table 1: List of alkynes

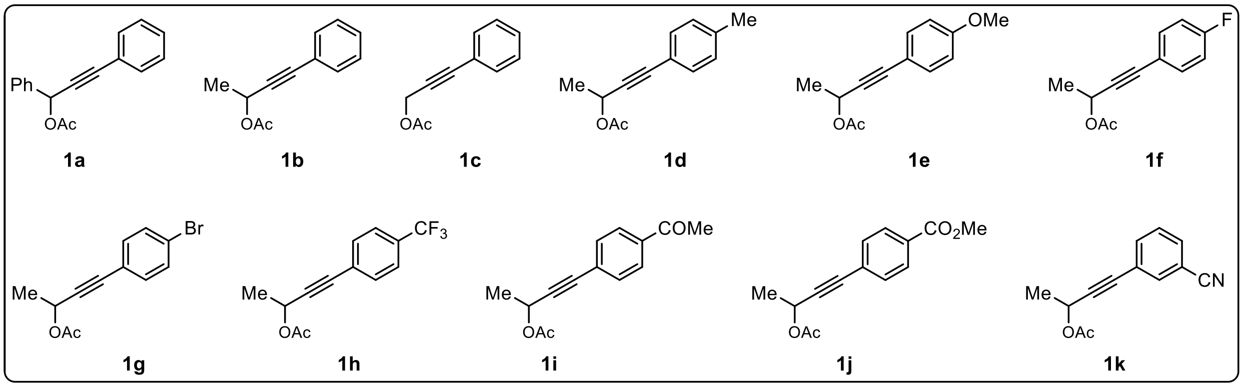

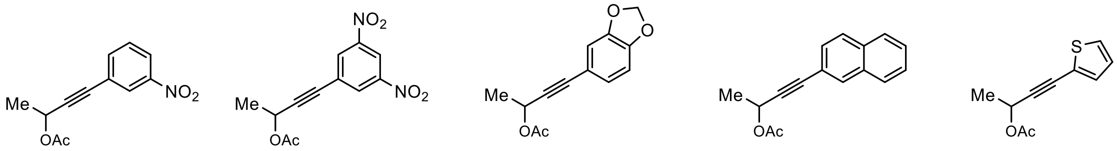

1l **1m** **1n** **1o** **1p**

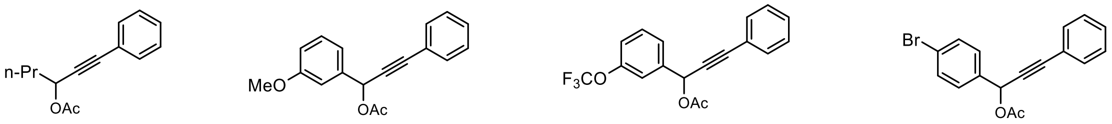

1q **1r** **1s** **1t**

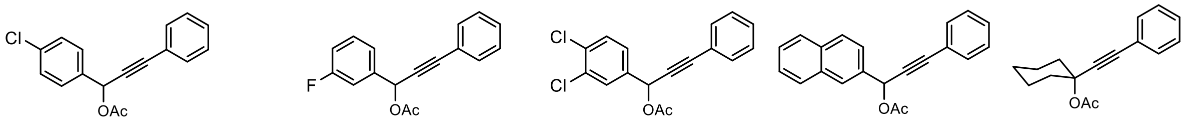

1u **1v** **1w** **1x** **1y**

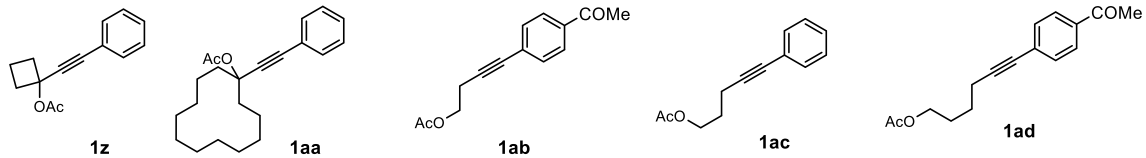

1z **1aa** **1ab** **1ac** **1ad**

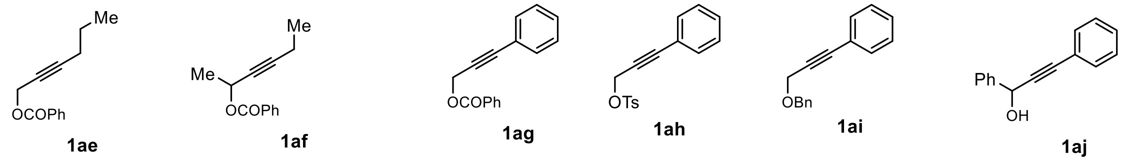

1ae **1af** **1ag** **1ah** **1ai** **1aj**

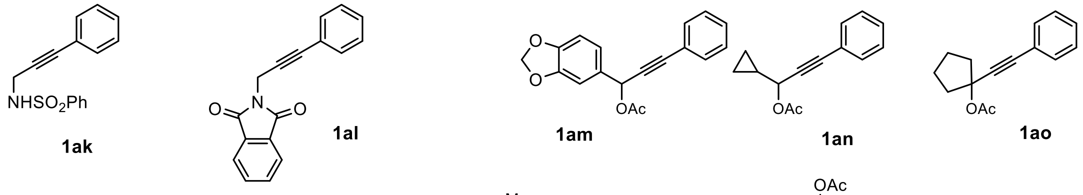

1ak **1al** **1am** **1an** **1ao**

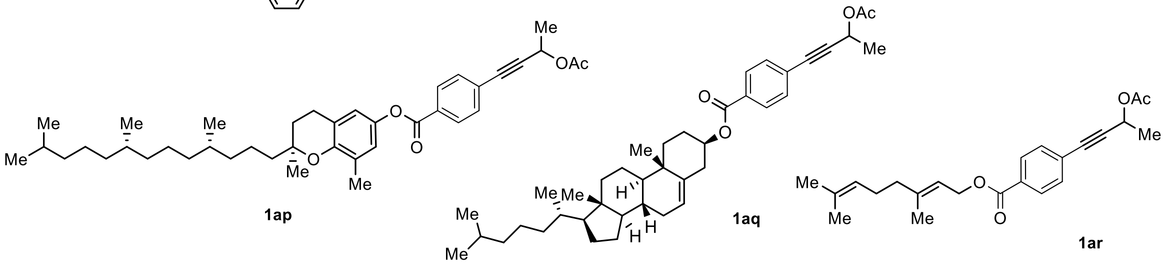

1ap **1aq** **1ar**

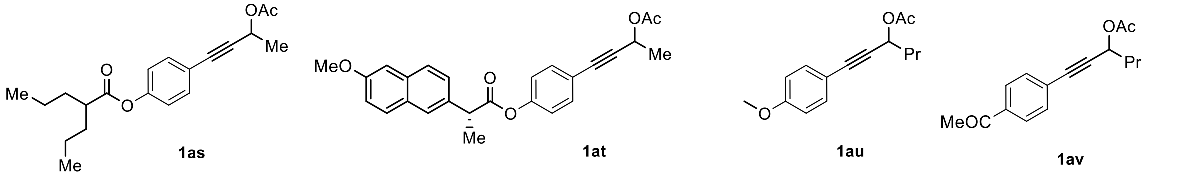

1as **1at** **1au** **1av**

(S)-2,8-Dimethyl-2-((4S,8S)-4,8,12-trimethyltridecyl)chroman-6-yl 4-(3-acetoxybut-1-yn-1-yl)benzoate (1ap):

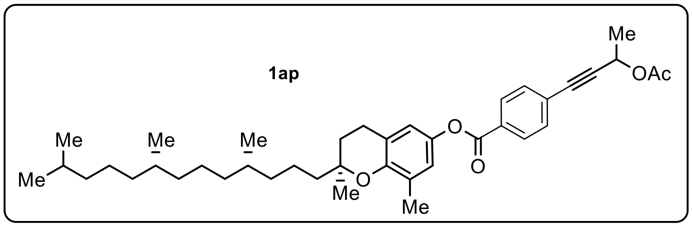

1ap (73%) as pale yellow gummy liquid; R_f = 0.58 (10% EtOAc/Hex); ^{1}H NMR (500 MHz, $CDCl_3$) δ 8.11 (d, J = 9.0 Hz, 2H), 7.55 (d, J = 8.5 Hz, 2H), 6.77 (dd, J = 25.5, 2.5 Hz, 2H), 5.71 (q, J = 6.5 Hz, 1H), 2.81–2.72 (m, 2H), 2.18 (s, 3H), 2.13 (s, 3H), 1.88–1.73 (m, 2H), 1.64–1.50 (m, 7H), 1.46–1.37 (m, 3H), 1.33–1.25 (m, 10H), 1.18–1.03 (m, 6H), 0.91–0.83 (m, 13H); ^{13}C NMR (126 MHz, $CDCl_3$) δ 169.8, 165.1, 149.9, 142.5, 131.8, 129.9, 129.7, 127.4, 127.3, 121.1, 121.0, 119.0, 90.6, 83.7, 76.2, 60.6, 40.1, 39.3, 37.42, 37.39, 37.3, 34.6, 32.8, 32.7, 31.6, 31.0, 27.9, 24.8, 24.4, 24.2, 22.7, 22.6, 22.5, 21.3, 21.0, 20.9, 19.7, 16.1; IR (Neat) ν_{max} 1736, 1471, 1370, 1261, 1221, 1072, 1033, 1016 cm^{-1}; **HRMS (ESI)** for $C_{40}H_{57}O_5$ (M+H)$^+$: calcd. 671.4201, found 671.4204.

(3R,8R,9R,10S,13S,14R,17S)-10,13-Dimethyl-17-((S)-6-methylheptan-2-yl) 2,3,4,7,8,9,10,11,12,13,14,15,16,17-tetradecahydro-1H-cyclopenta[a]phenanthren-3-yl 4-(3-acetoxybut-1-yn-1-yl)benzoate (1aq):

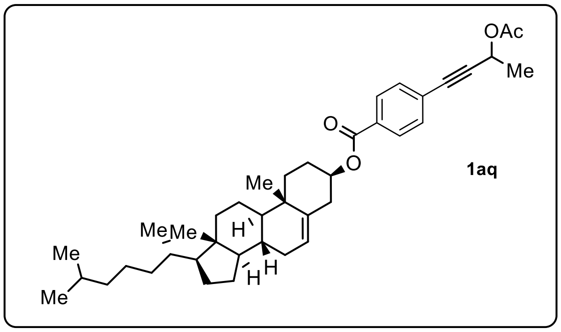

1aq (70%) as pale yellow semi-solid; R_f = 0.64 (10% EtOAc/Hex); ^{1}H NMR (500 MHz, $CDCl_3$) δ 7.97 (d, J = 8.5 Hz, 2H), 7.48 (d, J = 8.0 Hz, 2H), 5.68 (q, J = 6.5 Hz, 1H), 5.41 (d, J = 4.0 Hz, 1H), 4.89–4.79 (m, 1H), 2.51–2.41 (m, 2H), 2.10 (s, 3H), 2.05–1.94 (m, 4H), 1.93–1.80 (m, 3H), 1.78–1.69 (m, 2H), 1.60–1.43 (m, 10H), 1.39–1.31 (m, 3H), 1.28–1.08 (m, 10H), 0.91 (d, J = 6.5 Hz, 3H), 0.86 (dd, J = 6.5, 2.0 Hz, 6H), 0.68 (s, 3H); ^{13}C NMR (126 MHz, $CDCl_3$) δ 169.8, 165.3, 139.5, 131.6, 130.6, 129.3, 126.7, 122.8, 90.1, 83.8, 74.8, 60.6, 56.6, 56.1, 50.0, 42.3, 39.7, 39.5, 38.1, 37.0, 36.6, 36.1, 35.8, 31.9, 31.8, 28.2, 28.0, 27.8, 24.2, 23.8, 22.8, 22.5, 21.3, 19.3, 18.7, 11.8; IR (Neat) ν_{max} 1746, 1714, 1369, 1272, 1225, 1106, 1033, 952, 767 cm^{-1}; **HRMS (ESI)** for $C_{40}H_{56}O_4Na$ (M+Na)$^+$: calcd. 623.4071, found 623.4073.

(E)-3,7-Dimethylocta-2,6-dien-1-yl 4-(3-acetoxybut-1-yn-1-yl)benzoate (1ar):

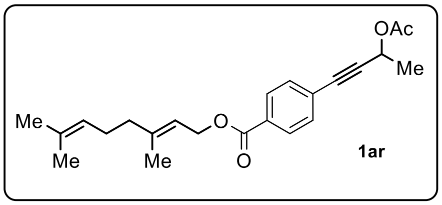

1ar (65%) as pale yellow gummy liquid; R_f = 0.58 (10% EtOAc/Hex); ^{1}H NMR (500 MHz, $CDCl_3$) δ 7.98 (d, J = 8.5 Hz, 2H), 7.48 (d, J = 8.5 Hz, 2H), 5.68 (q, J = 7.0 Hz, 1H), 5.49–5.42 (m, 1H), 5.12–5.05 (m, 1H), 4.83 (d, J = 7.0 Hz, 2H), 2.15–2.04 (m, 7H), 1.75 (m, 3H), 1.66 (m, 3H), 1.61–1.51 (m, 6H); ^{13}C NMR (126 MHz, $CDCl_3$) δ 169.9, 166.0, 142.6, 131.8, 131.7, 130.3, 129.4, 126.7, 123.7, 118.2, 90.2, 83.8, 62.0, 60.6, 39.5, 26.2, 25.6, 21.3, 21.0, 17.7, 16.5; IR (Neat) ν_{max} 1716, 1371, 1266, 1225, 1084, 1032, 1016, 951, 767 cm^{-1}; **HRMS (ESI)** for $C_{23}H_{29}O_4$ $(M+H)^+$: calcd. 369.2060, found 369.2061.

4-(3-Acetoxybut-1-yn-1-yl)phenyl 2-propylpentanoate (1as):

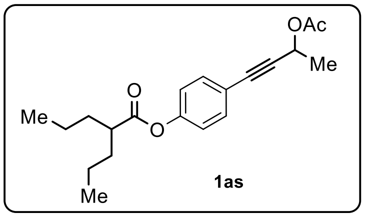

1as (78%) as pale yellow gummy liquid; R_f = 0.61 (10% EtOAc/Hex); ^{1}H NMR (500 MHz, $CDCl_3$) δ 7.44 (d, J = 9.0 Hz, 2H), 7.09 (d, J = 9.0 Hz, 2H), 5.67 (q, J = 6.5 Hz, 1H), 2.63–2.56 (m, 1H), 2.10 (m, 3H), 1.78–1.68 (m, 2H), 1.59–1.50 (m, 5H), 1.46–1.37 (m, 4H), 0.95 (q, J = 7.0 Hz, 6H); ^{13}C NMR (126 MHz, $CDCl_3$) δ 174.6, 169.9, 150.9, 133.0, 121.7, 119.7, 87.4, 83.8, 60.7, 45.3, 34.6, 21.4, 21.1, 20.7, 14.0; IR (Neat) ν_{max} 1742, 1503, 1370, 1227, 1199, 1102, 1032, 752 cm^{-1}; **HRMS (ESI)** for $C_{20}H_{26}O_4$ $(M+Na)^+$: calcd. 353.1723, found 353.1724.

4-(3-Acetoxybut-1-yn-1-yl)phenyl (2R)-2-(6-methoxynaphthalen-2-yl)propanoate (1at):

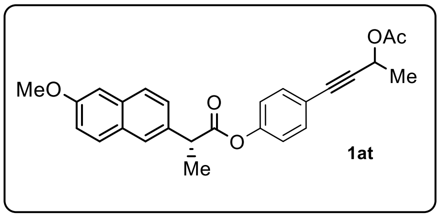

1at (75%) as pale yellow gummy liquid; R_f = 0.5 (10% EtOAc/Hex); ^{1}H NMR (500 MHz, $CDCl_3$) δ 7.78–7.72 (m, 3H), 7.44 (dd, J = 8.5, 2.0 Hz, 1H), 7.40 (d, J = 8.5 Hz, 2H), 7.19–7.12 (m, 2H), 6.95 (d, J = 9.0 Hz, 2H), 5.67 (q, J = 6.5 Hz, 1H), 4.08 (q, J = 7.0 Hz, 1H), 3.92 (s, 3H), 2.09 (s, 3H), 1.69 (d, J = 7.5 Hz, 3H), 1.56 (d, J = 7.0 Hz, 3H); ^{13}C NMR (126 MHz, $CDCl_3$) δ 172.8, 169.9, 157.8, 1509, 134.9, 133.8, 133.0, 132.9, 129.3, 129.0, 127.4, 126.1, 126.0, 121.6, 121.4, 119.8, 119.1, 105.6, 87.4, 83.7, 60.7, 55.3, 45.5, 21.4, 21.1, 18.4; IR (Neat) ν_{max} 1738, 1631, 1502, 1369, 11226, 1196, 1126, 1029, 848 cm^{-1}; **HRMS (ESI)** for $C_{26}H_{24}O_5Na$ $(M+Na)^+$: calcd. 439.1516, found 439.1514.

Supplementary Table 2: List of aryl(hetero) diazonium salts and aryl(hetero) boronic acids

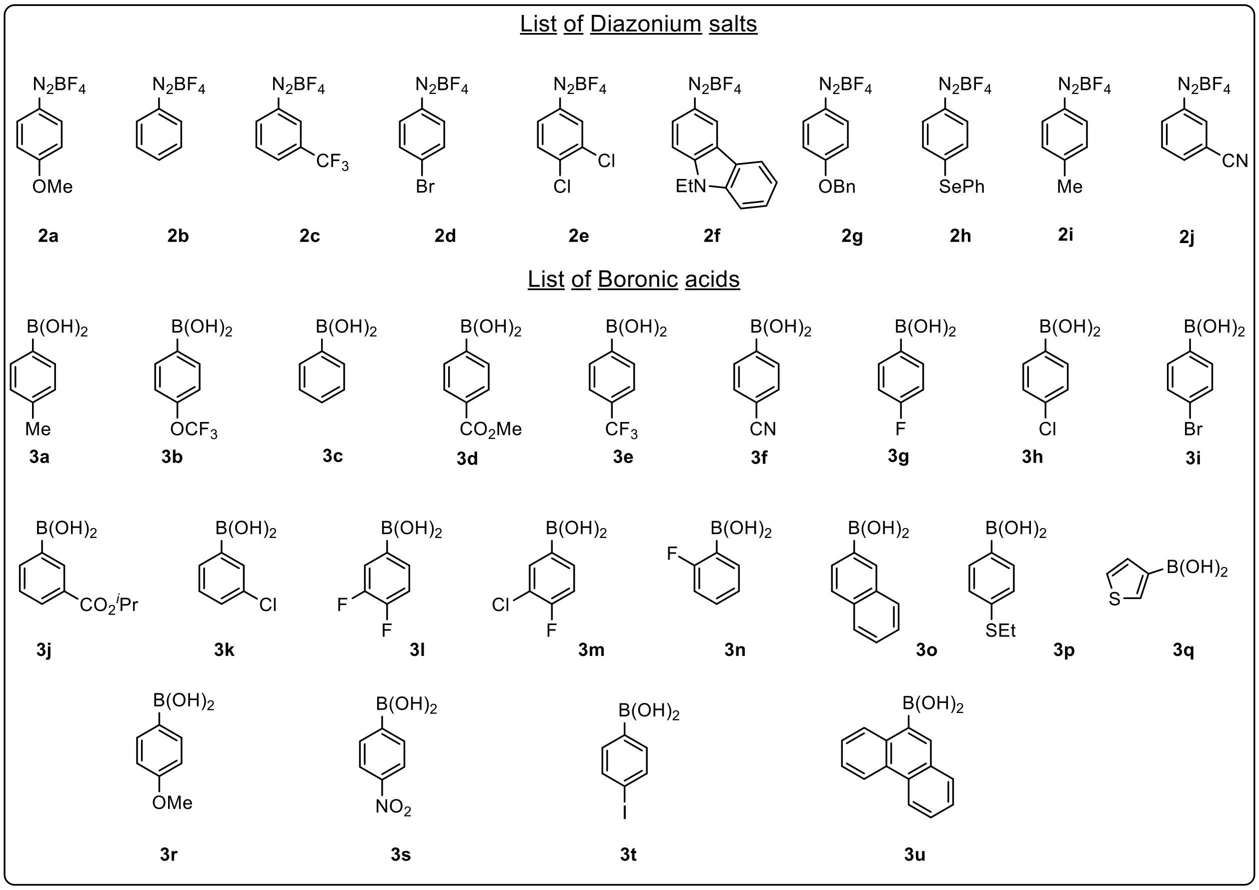

Supplementary Table 3: Reaction optimization

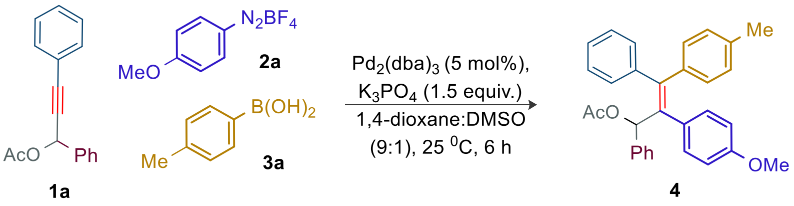

To a mixture of **1a** (0.2 mmol), *p*-methoxy benzenediazonium-tetrafluoroborate **2a** (0.6 mmol), *p*-tolyl boronic acid **3a** (0.3 mmol), Pd catalyst (0.01 mmol), and base (0.3 mmol) was added solvent (1.2 mL). The resulting reaction mixture was stirred at 25 °C for 6 h. The reaction progress was periodically monitored by TLC. The respective solvent was next removed either by water workup or by evaporation under reduced pressure. The organic layer was extracted in ethyl acetate (3 × 10 mL) and dried over Na_2SO_4. The organic layer was evaporated and purified by column chromatography over neutral alumina to afford **4**. The compounds are sensitive to acidic silica gel and thus, final product purification was carried out on neutral alumina.

entry	deviation from the standard condition	yield of **4**
1	None	73%
2	10 mol% $Pd(dba)_2$ as catalyst	58%[b]
3	$Pd_2(dba)_3·CHCl_3$ as catalyst	63%
4	$Pd(PPh_3)_4$ as catalyst	47%
5	$NaHCO_3$ as base	56%
6	KH_2PO_4 as base	62%
7	K_2CO_3 as base	<10%
8	Na_2CO_3 as base	31%
9	Cs_2CO_3 as base	64%
10	$NaHCO_3$ as base	27%
11	NaOH as base	<10%
12	KOH as base	<10%
13	KF as base	46%
14	CsF	51%
15	No base	16%
16	KOAc as base	<10%
17	NaO^tBu as base	52%
18	THF as solvent	26%
19	1,4-dioxane as solvent	49%
20	DMSO as solvent	55%
21	toluene as solvent	23%
22	DMF as solvent	<10%
23	0.1M solvent	64%
24	0.5M solvent	55%
25	0.01M solvent	complex
26	1M solvent	complex
27	2.0 equiv of **2a** and 1.2 equiv of **3a**	64%

2.11 General procedure for the regioselective double arylation of alkynes (1) with aryl(hetero) diazonium salts (2) and aryl(hetero) boronic acids (3) (GP-4):

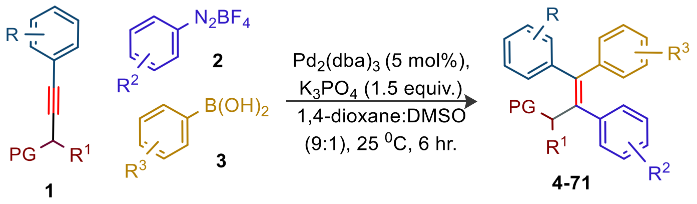

To a mixture of **1** (0.3 mmol), aryl(hetero) diazonium-tetrafluoroborate **2** (0.9 mmol), aryl(hetero) boronic acid **3** (0.45 mmol), $Pd_2(dba)_3$ (0.015 mmol), and K_3PO_4 (0.45 mmol) was added 1,4-dioxane:DMSO (9:1, 2.0 mL). The reaction mixture was stirred at 25 °C for 6 h. The reaction progress was periodically monitored by TLC. The reaction mixture was diluted with EtOAc (20 mL) and washed with water (3 × 10 mL) and brine. The organic layer was dried over Na_2SO_4. The solvent was evaporated and purified by column chromatography over neutral alumina eluting with EtOAc/hexane to afford **4–71**.

Note: 1,4-Dioxane:DMSO (9:1, 3.0 mL) was used for preparation of **21**–**26**.

2-(4-Methoxyphenyl)-1,3-diphenyl-3-(*p*-tolyl)propyl acetate (4):

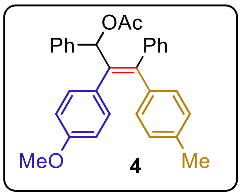

4 (98 mg, 73%) as pale yellow gummy liquid; $R_f = 0.5$ (10% EtOAc/Hex); ^{1}H NMR (600 MHz, DMSO d$_6$) δ 7.39–7.32 (m, 4H), 7.29–7.22 (m, 3H), 7.19 (t, J = 6.6 Hz, 1H), 7.10 (d, J = 7.8 Hz, 2H), 6.83 (d, J = 7.8 Hz, 2H), 6.77 (d, J = 7.2 Hz, 2H), 6.73 (d, J = 8.4 Hz, 2H), 6.63 (s, 1H), 6.58 (d, J = 8.4 Hz, 2H), 3.58 (s, 3H), 2.08 (s, 3H), 1.99 (s, 3H); ^{13}C NMR (151 MHz, DMSO d$_6$) δ 169.7, 158.2, 144.3, 142.3, 139.5, 139.2, 136.5, 136.0, 132.3, 130.3, 130.1, 129.4, 129.01, 128.8, 128.7, 127.8, 127.7, 126.4, 113.3, 75.6, 55.3, 21.4, 21.1; IR (Neat) ν_{max} 1736, 1521, 1235, 1179, 1026, 779 cm^{-1}; **HRMS (ESI)** for $C_{31}H_{28}NaO_3$ $(M+Na)^+$: calcd. 471.1931, found 471.1985.

(E)-2-(4-Methoxyphenyl)-1,3-diphenyl-3-(4-(trifluoromethoxy)phenyl)allyl acetate (5):

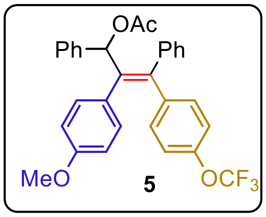

5 (112 mg, 72%) as pale yellow liquid; $R_f = 0.5$ (10% EtOAc/Hex); ^{1}H NMR (400 MHz, DMSO d$_6$) δ 7.48–7.42 (m, 4H), 7.39–7.29 (m, 3H), 7.26 (d, J = 7.2 Hz, 1H), 7.19 (d, J = 7.6 Hz, 2H), 7.11–7.06 (m, 4H), 6.81 (d, J = 8.8 Hz, 2H), 6.70 (s, 1H), 6.65 (d, J = 8.8 Hz, 2H), 3.63 (s, 3H), 2.05 (s, 3H); ^{13}C NMR (101 MHz, DMSO d$_6$) δ 169.7, 158.4, 147.0, 143.0, 141.6, 141.5, 138.8, 137.9, 132.2, 131.9, 129.7, 129.4, 129.1, 128.7, 128.0, 127.9, 126.4, 120.6, 120.4 (q, J = 261 Hz, 1C), 113.3, 75.4, 55.2, 21.3;

^{19}F NMR (376 MHz) δ −56.8; IR (Neat) ν_{max} 1735, 1597, 1447, 1238, 720 cm^{-1}; **HRMS (ESI)** for $C_{31}H_{25}F_3NaO_4$ $(M+Na)^+$: calcd. 541.1603, found 541.1604.

2-(4-Methoxyphenyl)-1,3,3-triphenylallyl acetate (6):

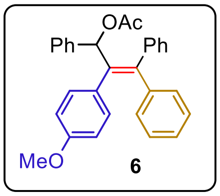

6 (94 mg, 72%) as pale yellow liquid; R_f = 0.5 (10% EtOAc/Hex); ^{1}H NMR (400 MHz, $CDCl_3$) δ 7.54–7.48 (m, 2H), 7.42 (t, J = 7.6 Hz, 2H), 7.36–7.22 (m, 6H), 7.08–6.95 (m, 5H), 6.91 (s, 1H), 6.81 (dt, J = 8.0, 2.8 Hz, 2H), 6.65 (dt, J = 8.8, 2.8 Hz, 2H), 3.71 (s, 3H), 2.02 (s, 3H); ^{13}C NMR (101 MHz, $CDCl_3$) δ 169.7, 158.1, 143.7, 142.2, 141.8, 138.9, 136.9, 132.0, 130.2, 130.1, 129.4, 128.4, 128.0, 127.4, 127.3, 127.2, 126.3, 126.1, 112.7, 76.1, 54.8, 21.0; IR (Neat) ν_{max} 1740, 1605, 1509, 1442, 1230, 751 cm^{-1}; **HRMS (ESI)** for $C_{30}H_{26}NaO_3$+ $(M+Na)^+$: calcd. 457.1774, found 457.1773.

(E)-Methyl 4-(3-acetoxy-2-(4-methoxyphenyl)-1,3-diphenylprop-1-en-1-yl)benzoate (7):

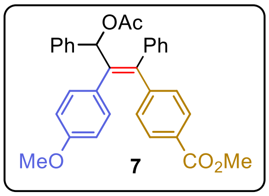

7 (92 mg, 62%) as pale yellow liquid; R_f = 0.3 (10% EtOAc/Hex); ^{1}H NMR (500 MHz, $CDCl_3$) δ 7.74 (d, J = 8.5 Hz, 2H), 7.54–7.48 (m, 2H), 7.44 (t, J = 7.5 Hz, 2H), 7.37–7.23 (m, 6H), 7.08–7.04 (m, 2H), 6.92 (s, 1H), 6.86–6.81 (m, 2H), 6.63 (d, J = 8.5 Hz, 2H), 3.84 (s, 3H), 3.72 (s, 3H), 2.02 (s, 3H); ^{13}C NMR (101 MHz,$CDCl_3$) 169.7, 166.8, 158.3, 147.2, 142.6, 141.0, 138.6, 138.5, 131.9, 130.1, 129.7, 129.5, 128.8, 128.5, 128.1, 127.6, 127.5, 127.4, 126.3, 112.9, 75.9, 54.9, 51.9, 21.0; IR (Neat) ν_{max} 1735, 1603, 1492, 1228, 1029, 695 cm^{-1}; **HRMS (ESI)** for $C_{32}H_{28}NaO_5$+ $(M+Na)^+$: calcd. 515.1829, found 515.1858.

(E)-2-(4-Methoxyphenyl)-1,3-diphenyl-3-(4-(trifluoromethyl)phenyl)allyl acetate (8):

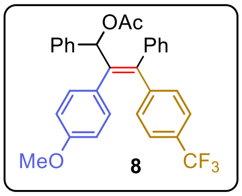

8 (113 mg, 75%) as pale yellow liquid; R_f = 0.5 (10% EtOAc/Hex); ^{1}H NMR (400 MHz, DMSO d_6) δ 7.49–7.42 (m, 6H), 7.39–7.25 (m, 4H), 7.19 (d, J = 8.0 Hz, 4H), 6.82 (d, J = 8.8 Hz, 2H), 6.72 (s, 1H), 6.65 (d, J = 8.8 Hz, 2H), 3.62 (s, 3H), 2.05 (s, 3H); ^{13}C NMR (101 MHz, DMSO d_6) δ 169.7, 158.5, 146.7, 143.0, 141.2, 138.7, 138.5, 132.2, 130.8, 129.50, 129.45, 129.2, 128.7, 128.1, 127.9, 127.2 (q, J = 31 Hz, 1C), 126.4, 125.1 (q, J = 4.0 Hz, 2C), 124.6 (q, J = 273 Hz, 1C), 113.3, 75.3, 55.3, 21.2; ^{19}F NMR (376 MHz) δ −61.0; IR (Neat) ν_{max} 1741, 1509, 1324, 1264, 1123, 1065, 731 cm^{-1}; **HRMS (ESI)** for $C_{31}H_{25}F_3NaO_3$+ $(M+Na)^+$: calcd. 525.1648, found 525.1654.

(E)-3-(4-Cyanophenyl)-2-(4-methoxyphenyl)-1,3-diphenylallyl acetate (9):

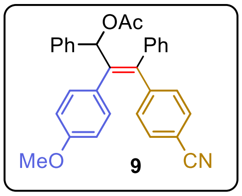

9 (98 mg, 71%) as pale yellow semi-solid; R_f = 0.28 (10% EtOAc/Hex); ^{1}H NMR (500 MHz, DMSO d_6) δ 7.55 (d, J = 8.5 Hz, 2H), 7.44 (d, J = 9.0 Hz, 4H), 7.38–7.33 (m, 1H), 7.31 (d, J = 8.0 Hz, 2H), 7.26 (d, J = 7.0 Hz, 1H), 7.18 (br d, J = 7.5 Hz, 2H), 7.15 (d, J = 8.5 Hz, 2H), 6.80 (d, J = 8.5 Hz, 2H), 6.69 (s, 1H), 6.65 (d, J = 9.0 Hz, 2H), 3.63 (s, 3H), 2.03 (s, 3H); ^{13}C NMR (101 MHz, DMSO d_6) δ 169.7, 158.5, 147.5, 142.9, 140.9, 138.7, 138.6, 132.2, 132.1, 131.0, 129.5, 129.4, 129.2, 128.8, 128.2, 128.0, 126.4, 119.1, 113.4, 109.4, 75.2, 55.3, 21.3; IR (Neat) ν_{max} 2225, 1739, 1603, 1508, 1369, 1287, 1226, 1028, 698 cm^{-1}; **HRMS (ESI)** for $C_{31}H_{25}NNaO_3$+ (M+Na)$^+$: calcd. 482.1727, found 482.1729.

(E)-3-(4-Fluorophenyl)-2-(4-methoxyphenyl)-1,3-diphenylallyl acetate (10):

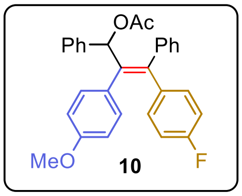

10 (mg, 61%) as pale yellow liquid; R_f = 0.5 (10% EtOAc/Hex); ^{1}H NMR (400 MHz, $CDCl_3$) δ 7.47–7.41 (m, 4H), 7.38–7.24 (m, 4H), 7.17 (d, J = 7.6 Hz, 2H), 7.01–6.88 (m, 4H), 6.79 (d, J = 7.6 Hz, 2H), 6.69 (s, 1H), 6.64 (d, J = 8.0 Hz, 2H), 3.64 (s, 3H), 2.05 (s, 3H); ^{13}C NMR (126 MHz, $CDCl_3$) δ 169.7, 161.0 (d, J = 246 Hz, 1C), 158.2, 142.5, 141.6, 138.8, 138.2, 137.3, 132.0, 131.8 (d, J = 7.6 Hz, 2C), 130.1, 129.4, 128.6, 128.5, 128.1, 127.3 (d, J = 1.3 Hz, 1C), 126.3, 114.4 (d, J = 21.4 Hz, 2C), 112.8, 76.1, 54.9, 21.0; ^{19}F NMR (471 MHz) δ −115.6; IR (Neat) ν_{max} 1663, 1597, 1504, 1244, 1175, 1027, 835, 734 cm^{-1}; **HRMS (ESI)** for $C_{30}H_{25}FNaO_3$+ (M+Na)$^+$: calcd. 475.1680, found 475.1684.

(E)-3-(4-Chlorophenyl)-2-(4-methoxyphenyl)-1,3-diphenylallyl acetate (11):

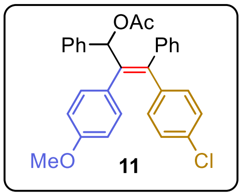

11 (111 mg, 79%) as pale yellow liquid; R_f = 0.5 (10% EtOAc/Hex); ^{1}H NMR (600 MHz, $CDCl_3$) δ 7.48–7.45 (m, 2H), 7.41 (t, J = 7.2 Hz, 2H), 7.35–7.19 (m, 6H), 6.99 (d, J = 9.0 Hz, 2H), 6.88 (d, J = 9.0 Hz, 3H), 6.81 (d, J = 8.4 Hz, 2H), 6.63 (d, J = 9.0 Hz, 2H), 3.72 (s, 3H), 1.99 (s, 3H); ^{13}C NMR (151 MHz, $CDCl_3$) δ 169.7, 158.3, 142.3, 141.4, 140.7, 138.7, 137.7, 132.0, 131.5, 129.9, 129.5, 128.5, 128.1, 127.7, 127.4, 126.4, 112.9, 76.0, 54.9, 21.0; IR (Neat) ν_{max} 1739, 1605, 1508, 1227, 1029, 812, 698 cm^{-1}; **HRMS (ESI)** for $C_{30}H_{25}ClO_3Na$+ (M+Na)$^+$: calcd. 491.1384, found 491.1381.

(E)-3-(4-Bromophenyl)-2-(4-methoxyphenyl)-1,3-diphenylallyl acetate (12):

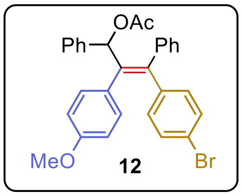

12 (125 mg, 81%) as pale yellow semi-solid; R_f = 0.5 (10% EtOAc/Hex); ^{1}H NMR (400 MHz, DMSO d_6) δ 7.41 (d, J = 4.4 Hz, 4H), 7.38–7.24 (m, 6H), 7.19 (br d, J = 7.6 Hz, 2H), 6.91 (d, J = 8.4 Hz, 2H), 6.81 (d, J = 8.8 Hz, 2H), 6.71 (s, 1H), 6.66 (d, J = 8.8 Hz, 2H), 3.64 (s, 3H), 2.05 (s, 3H); ^{13}C NMR (101 MHz, DMSO d_6)

δ 169.7, 158.4, 143.1, 141.7, 141.5, 138.8, 137.6, 132.2, 129.8, 129.4, 129.1, 128.7, 127.94, 127.88, 126.4, 120.2, 113.4, 75.4, 55.2, 21.3; IR (Neat) ν_{max} 1736, 1604, 1508, 1483, 1225, 1175, 696 cm^{-1}; **HRMS (ESI)** for $C_{30}H_{25}BrO_3Na$ + $(M+Na)^+$: calcd. 535.0879, found 535.0889.

(E)-Isopropyl 3-(3-acetoxy-2-(4-methoxyphenyl)-1,3-diphenylprop-1-en-1-yl)benzoate (13):

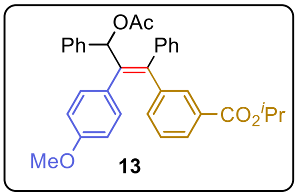

13 (108 mg, 69%) as pale yellow liquid; R_f = 0.35 (10% EtOAc/Hex); ^1H NMR (500 MHz, DMSO d_6) δ 7.63–7.58 (m, 1H), 7.57–7.54 (m, 1H), 7.48–7.41 (m, 4H), 7.38–7.28 (m, 3H), 7.27–7.17 (m, 5H), 6.81 (d, J = 9.0 Hz, 2H), 6.73 (s, 1H), 6.64 (d, J = 9.0 Hz, 2H), 5.07–4.99 (m, 1H), 3.60 (s, 3H), 2.05 (s, 3H), 1.22 (d, J = 6.0 Hz, 6H); ^{13}C NMR (126 MHz, DMSO d_6) δ 169.7, 165.3, 158.4, 143.2, 142.6, 141.5, 138.9, 138.0, 134.8, 132.2, 130.8, 130.0, 129.7, 129.5, 129.1, 128.7, 128.5, 128.0, 127.9, 127.6, 126.4, 113.4, 75.4, 68.5, 55.2, 22.0, 21.3; IR (Neat) ν_{max} 1710, 1579, 1275, 1101, 754 cm^{-1}; **HRMS (ESI)** for $C_{34}H_{32}NaO_5$+ $(M+Na)^+$: calcd. 543.2142, found 543.2152.

(E)-3-(3-Chlorophenyl)-2-(4-methoxyphenyl)-1,3-diphenylallyl acetate (14):

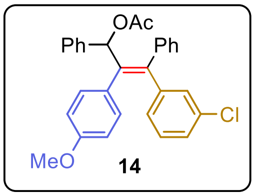

14 (83 mg, 59%) as pale yellow liquid; R_f = 0.5 (10% EtOAc/Hex); ^1H NMR (500 MHz, DMSO d_6) δ 7.48–7.41 (m, 4H), 7.38–7.28 (m, 3H), 7.28–7.23 (m, 1H), 7.17 (d, J = 8.0 Hz, 2H), 7.13–7.02 (m, 2H), 7.00 (s, 1H), 6.91 (br d, J = 6.5 Hz, 1H), 6.81 (d, J = 8.5 Hz, 2H), 6.67 (d, J = 9.0 Hz, 2H), 6.65 (s, 1H), 3.63 (s, 3H), 2.05 (s, 3H); ^{13}C NMR (126 MHz, DMSO d_6) δ 169.9, 158.4, 144.5, 142.9, 141.3, 138.7, 138.0, 132.7, 132.2, 130.0, 129.6, 129.4, 129.1, 128.72, 128.70, 128.0, 127.9, 126.8, 126.4, 113.3, 75.3, 55.3, 21.3; IR (Neat) ν_{max} 1740, 1605, 1508, 1370, 1224, 1164, 1029, 700 cm^{-1}; **HRMS (ESI)** for $C_{30}H_{25}ClO_3Na$+ $(M+Na)^+$: calcd. 491.1384, found 491.1381.

(E)-3-(3,4-Difluorophenyl)-2-(4-methoxyphenyl)-1,3-diphenylallyl acetate (15):

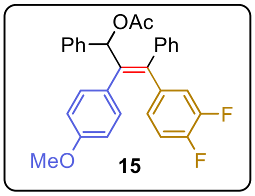

15 (115 mg, 84%) as pale yellow liquid; R_f = 0.5 (10% EtOAc/Hex); ^1H NMR (600 MHz, $CDCl_3$) δ 7.48–7.39 (m, 4H), 7.35–7.22 (m, 4H), 7.18 (d, J = 7.8 Hz, 2H), 6.83 (s, 1H), 6.83–6.72 (m, 4H), 6.68–6.62 (m, 3H), 3.72 (s, 3H), 1.99 (s, 3H); ^{13}C NMR (101 MHz, DMSO d_6) δ 169.7, 158.5, 149.1 (dd, J = 246, 13.1 Hz, 1C), 148.3 (dd, J = 247, 13.1 Hz, 1C), 142.3, 141.2, 140.2–139.7 (m, 1C), 138.7, 138.2, 132.2, 129.6, 129.4, 129.1, 128.7, 128.1, 127.9, 127.0, 126.4, 118.9 (d, J = 17.2 Hz, 1C), 117.3 (d, J = 17.2 Hz, 1C), 113.4, 75.2, 55.3, 21.3; ^{19}F NMR (471 MHz) δ –139.3 (d, J = 22.6 Hz, 1F), –141.1 (d, J = 22.6 Hz, 1F); IR (Neat) ν_{max} 1739, 1603, 1507, 1225, 1027, 698 cm^{-1}; **HRMS (ESI)** for $C_{30}H_{24}F_2NaO_3$ $(M+Na)^+$: calcd. 493.1586, found 493.1587.

(E)-3-(4-Chloro-3-fluorophenyl)-2-(4-methoxyphenyl)-1,3-diphenylallyl acetate (16):

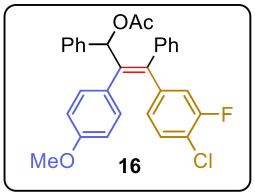

16 (108 mg, 74%) as pale yellow gummy-liquid; R_f = 0.5 (10% EtOAc/Hex); ^{1}H NMR (500 MHz, DMSO d_6) δ 7.49–7.41 (m, 4H), 7.37–7.27 (m, 3H), 7.24 (br d, J = 7.0 Hz, 1H), 7.21–7.09 (m, 4H), 6.98–6.92 (m, 1H), 6.83 (d, J = 8.5 Hz, 2H), 6.67 (d, J = 8.0 Hz, 3H), 3.63 (s, 3H), 2.04 (s, 3H); ^{13}C NMR (101 MHz, DMSO d_6) δ 169.7, 158.5, 156.1 (dd, J = 248 Hz, 1C), 142.1, 141.2, 140.2, 138.7, 138.3, 132.2, 131.9, 130.8, 129.6, 129.4, 129.1, 128.7, 128.1, 127.9, 126.4, 126.4, 119.2 (d, J = 17.2 Hz, 1C), 116.7 (d, J = 21.2 Hz, 1C), 113.4, 75.2, 55.3, 21.3; ^{19}F NMR (471 MHz) δ –118.8; IR (Neat) ν_{max} 1739, 1508, 1228, 1059, 1030, 699 cm^{-1}; **HRMS (ESI)** for $C_{30}H_{24}ClFNaO_3$+ (M+Na)$^+$: calcd. 509.1290, found 509.1292.

(E)-3-(2-Fluorophenyl)-2-(4-methoxyphenyl)-1,3-diphenylallyl acetate (17):

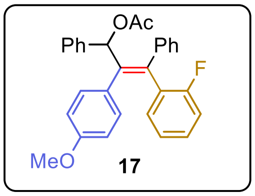

17 (85 mg, 63%) as pale yellow gummy-liquid; R_f = 0.5 (10% EtOAc/Hex); ^{1}H NMR (500 MHz, DMSO d_6) δ 7.48 (d, J = 7.5 Hz, 2H), 7.42 (t, J = 8.0 Hz, 2H), 7.33 (t, J = 7.0 Hz, 2H), 7.28–7.23 (m, 1H), 7.20–7.07 (m, 4H), 6.98–6.91 (m, 2H), 6.78 (s, 3H), 6.77 (d, J = 1.6 Hz, 2H), 6.60 (d, J = 8.5 Hz, 2H), 3.60 (s, 3H), 2.05 (s, 3H); ^{13}C NMR (101 MHz, DMSO d_6) δ 169.7, 158.8 (d, J = 245 Hz, 1C), 158.3, 140.7, 139.7, 138.7, 138.4, 131.7 (d, J = 3.0 Hz, 1C), 131.3, 130.3 (d, J = 17.2 Hz, 1C), 129.7, 129.5 (d, J = 8.1 Hz, 1C), 129.02, 128.98, 128.0, 126.3, 124.5 (d, J = 3.0 Hz, 1C), 115.7 (d, J = 22.2 Hz, 1C), 113.1, 74.8, 55.2, 21.3; ^{19}F NMR (471 MHz) δ –109.1; IR (Neat) ν_{max} 1738, 1605, 1509, 1226, 1026, 697 cm^{-1}; **HRMS (ESI)** for $C_{30}H_{25}FNaO_3$+ (M+Na)$^+$: calcd. 475.1680, found 475.1684.

(E)-3-(4-Methoxyphenyl)-4-(naphthalen-2-yl)-4-phenylbut-3-en-2-yl acetate (18):

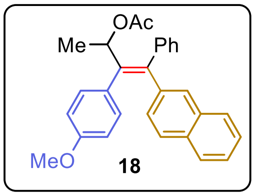

18 (77 mg, 61%) as pale yellow gummy liquid; R_f = 0.45 (10% EtOAc/Hex); ^{1}H NMR (500 MHz, $CDCl_3$) δ 7.73–7.68 (m, 1H), 7.66–7.61 (m, 1H), 7.57 (d, J = 8.5 Hz, 1H), 7.44–7.29 (m, 8H), 7.14 (dt, J = 6.5, 2.0 Hz, 2H), 7.09 (dd, J = 8.5, 1.5 Hz, 1H), 6.75 (d, J = 8.5 Hz, 2H), 5.67 (q, J = 6.5 Hz, 1H), 3.63 (s, 3H), 1.92 (s, 3H), 1.24 (d, J = 6.5 Hz, 3H); ^{13}C NMR (101 MHz, DMSO d_6) δ 169.8, 158.3, 142.4, 141.9, 140.2, 139.1, 132.9, 132.3, 131.8, 130.3, 129.5, 128.9, 128.8, 128.4, 128.2, 127.8, 127.7, 127.3, 126.5, 126.4, 113.5, 71.0, 55.3, 21.4, 19.9; IR (Neat) ν_{max} 1731, 1507, 1235, 1175, 1029, 700 cm^{-1}; **HRMS (ESI)** for $C_{29}H_{26}O_3Na$ (M+Na)$^+$: calcd. 445.1774, found 445.1784.

(E)-4-(4-(Ethylthio)phenyl)-3-(4-methoxyphenyl)-4-phenylbut-3-en-2-yl acetate (19):

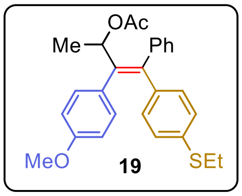

19 (75 mg, 58%) as pale yellow gummy liquid; R_f = 0.42 (10% EtOAc/Hex); ^{1}H NMR (500 MHz, DMSO d_6) δ 7.40 (t, J = 7.0 Hz, 2H), 7.36–7.24 (m, 3H), 7.08 (d, J = 9.0 Hz, 2H), 6.97 (d, J = 8.5 Hz, 2H), 6.81 (t, J = 8.5 Hz, 4H), 5.57 (q, J = 6.5 Hz, 1H), 3.70 (s, 3H), 2.84 (q, J = 7.5 Hz, 2H), 1.89 (s, 3H), 1.19 (d, J = 6.5 Hz, 3H), 1.13 (t, J = 7.0 Hz, 3H); ^{13}C NMR (126 MHz, DMSO d_6) δ 169.6, 158.4, 141.8, 138.6, 134.8, 132.2, 130.6, 130.3, 129.3, 128.8, 127.6, 127.1, 113.6, 71.0, 55.3, 26.2, 21.4, 19.8, 14.4; IR (Neat) ν_{max} 1731, 1507, 1490, 1367, 1235, 1029, 759 cm^{-1}; **HRMS (ESI)** for $C_{27}H_{28}NaO_3S$+ $(M+Na)^+$: calcd. 455.1651, found 455.1658.

(E)-3-(4-Methoxyphenyl)-4-phenyl-4-(thiophen-3-yl)but-3-en-2-yl acetate (20):

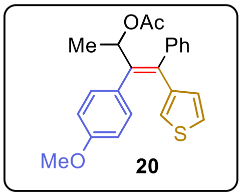

20 (78 mg, 69%) as pale yellow gummy liquid; R_f = 0.4 (10% EtOAc/Hex); ^{1}H NMR (500 MHz, DMSO d_6) δ 7.44 (t, J = 7.5 Hz, 2H), 7.36 (t, J = 7.5 Hz, 1H), 7.28 (d, J = 7.0 Hz, 2H), 7.18–7.10 (m, 3H), 6.92 (d, J = 9.0 Hz, 2H), 6.56 (dd, J = 3.0, 1.5 Hz, 1H), 6.28 (dd, J = 5.0, 1.0 Hz, 1H), 5.44 (q, J = 6.5 Hz, 1H), 3.76 (s, 3H), 1.88 (s, 3H), 1.15 (d, J = 6.5 Hz, 3H); ^{13}C NMR (126 MHz, DMSO d_6) δ 169.7, 158.0, 142.4, 141.7, 137.6, 136.7, 131.8, 130.5, 129.3, 128.9, 128.8, 127.7, 125.7, 125.1, 114.1, 71.2, 55.4, 21.4, 19.6; IR (Neat) ν_{max} 1731, 1507, 1234, 1174, 1029, 699 cm^{-1}; **HRMS (ESI)** for $C_{23}H_{22}NaO_3S$ $(M+Na)^+$: calcd. 401.1182, found 401.1188.

(E)-2-(4-Methoxyphenyl)-3-phenyl-3-(*p*-tolyl)allyl acetate (21):

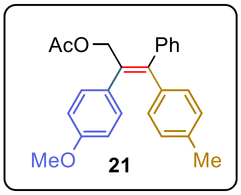

21 (87 mg, 78%) as semi-colorless solid; R_f = 0.39 (10% EtOAc/Hex); ^{1}H NMR (500 MHz, $CDCl_3$) δ 7.36–7.24 (m, 5H), 7.10 (dt, J = 6.5, 2.0 Hz, 2H), 6.88 (d, J = 8.0 Hz, 2H), 6.81 (d, J = 8.0 Hz, 2H), 6.73 (dt, J = 9.0, 2.5 Hz, 2H), 4.88 (s, 2H), 3.77 (s, 3H), 2.22 (s, 3H), 1.95 (s, 3H); ^{13}C NMR (126 MHz, $CDCl_3$) δ 171.0, 158.2, 144.7, 142.3, 139.2, 136.2, 132.9, 132.4, 130.9, 130.5, 129.5, 128.3, 128.2, 127.3, 113.4, 66.7, 55.1, 21.1, 20.9; IR (Neat) ν_{max} 1725, 1508, 1039, 859, 708 cm^{-1}; **HRMS (ESI)** for $C_{25}H_{24}O_3Na$+ $(M+Na)^+$: calcd. 395.1618, found 395.1626.

(E)-2,3-Bis(4-Methoxyphenyl)-3-phenylallyl acetate (22):

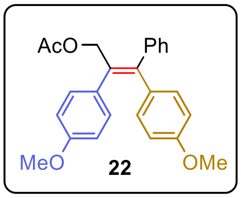

22 (52 mg, 44%) as pale yellow semi-solid solid; R_f = 0.38 (10% EtOAc/Hex); ^{1}H NMR (500 MHz, $CDCl_3$) δ 7.38–7.24 (m, 5H), 7.12 (d, J = 8.5 Hz, 2H), 6.85 (d, J = 8.5 Hz, 2H), 6.74 (d, J = 9.0 Hz, 2H), 6.61 (d, J = 9.0 Hz, 2H), 4.88 (s, 2H), 3.77 (s, 3H), 3.71 (s, 3H), 1.95 (s, 3H); ^{13}C NMR (126 MHz, $CDCl_3$) δ 170.9, 158.3, 158.1, 144.2, 142.4, 134.6, 132.5, 131.9, 130.9, 129.6, 128.2, 127.3, 113.5, 113.0, 66.7, 55.1, 55.0, 20.9; IR (Neat) ν_{max} 1731, 1605, 1508, 1243, 1023, 700 cm^{-1}; **HRMS (ESI)** for $C_{25}H_{24}O_4Na$+ $(M+Na)^+$: calcd. 411.1567, found 411.1575.

2-(4-Methoxyphenyl)-3,3-diphenylallyl acetate (23):

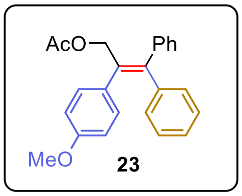

23 (88 mg, 82%) as colorless solid; R_f = 0.39 (10% EtOAc/Hex); M.P (196–198); ^{1}H NMR (500 MHz, $CDCl_3$) δ 7.38–7.25 (m, 5H), 7.14–7.05 (m, 5H), 6.98–6.93 (m, 2H), 6.72 (d, J = 9.0 Hz, 2H), 4.92 (s, 2H), 3.76 (s, 3H), 1.96 (s, 3H); ^{13}C NMR (126 MHz, $CDCl_3$) δ 170.9, 158.3, 144.7, 142.2, 142.1, 133.4, 132.1, 130.9, 130.6, 129.5, 128.2, 127.6, 127.3, 126.5, 113.3, 66.6, 55.0, 20.9; IR (Neat) ν_{max} 1731, 1509, 1245, 1224, 1024, 702 cm^{-1}; **HRMS (ESI)** for $C_{24}H_{22}O_3Na$+ $(M+Na)^+$: calcd. 381.1461, found 381.1469.

(E)-2-(4-Methoxyphenyl)-3-(4-nitrophenyl)-3-phenylallyl acetate (24):

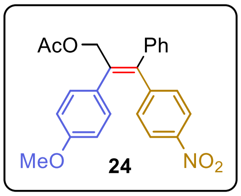

24 (70 mg, 58%) as pale yellow semi-solid; R_f = 0.22 (10% EtOAc/Hex); ^{1}H NMR (500 MHz, $CDCl_3$) δ 7.95 (d, J = 9.0 Hz, 2H), 7.43–7.33 (m, 3H), 7.30–7.24 (m, 2H), 7.13–7.06 (m, 4H), 6.75 (dt, J = 7.0, 2.0 Hz, 2H), 4.94 (s, 2H), 3.78 (s, 3H), 1.96 (s, 3H); ^{13}C NMR (126 MHz, $CDCl_3$) δ 170.7, 158.9, 149.3, 146.0, 142.3, 140.7, 136.7, 131.4, 130.9, 129.5, 128.6, 128.0, 122.9, 113.7, 66.0, 55.1, 20.8; IR (Neat) ν_{max} 1731, 1595, 1506, 1338, 1228, 1081, 700 cm^{-1}; **HRMS (ESI)** for $C_{24}H_{21}NO_5Na$+ $(M+Na)^+$: calcd. 426.1312, found 426.1320.

(E)-3-(4-Iodophenyl)-2-(4-methoxyphenyl)-3-phenylallyl acetate (25):

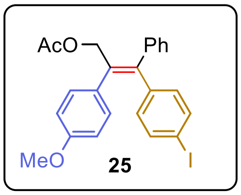

25 (86 mg, 59%) as pale yellow solid; R_f = 0.5 (10% EtOAc/Hex); M.P (210–211 °C); ^{1}H NMR (500 MHz, $CDCl_3$) δ 7.39 (d, J = 8.5 Hz, 2H), 7.37–7.28 (m, 3H), 7.27–7.22 (m, 2H), 7.08 (d, J = 8.5 Hz, 2H), 6.74 (dt, J = 6.5, 2.0 Hz, 2H), 6.67 (dt, J = 6.5, 1.5 Hz, 2H), 4.88 (s, 2H), 3.78 (s, 3H), 1.94 (s, 3H); ^{13}C NMR (126 MHz, $CDCl_3$) δ 170.8, 158.5, 143.4, 141.8, 141.5, 136.8, 134.2, 132.5, 131.6, 130.8, 129.5, 128.4, 127.6, 113.6, 92.4, 66.4, 55.1, 20.9; IR (Neat) ν_{max} 1730, 1605, 1508, 1229, 1023, 719 cm^{-1}; **HRMS (ESI)** for $C_{24}H_{21}IO_3Na$+ $(M+Na)^+$: calcd. 507.0428, found 507.0434.

(E)-2-(4-Methoxyphenyl)-3-(phenanthren-9-yl)-3-phenylallyl acetate (26):

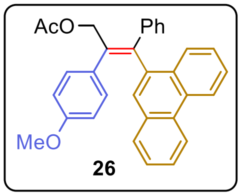

26 (84 mg, 61%) as pale yellow solid; R_f = 0.41 (10% EtOAc/Hex); ^{1}H NMR (500 MHz, $CDCl_3$) δ 8.67–8.58 (m, 2H), 8.19–8.11 (m, 1H), 7.77 (d, J = 7.5 Hz, 1H), 7.65–7.45 (m, 7H), 7.38–7.13 (m, 5H), 6.51 (d, J = 8.5 Hz, 2H), 5.21 (dd, J = 11.5, 11.5 Hz, 2H), 3.56 (s, 3H), 2.07 (s, 3H); ^{13}C NMR (126 MHz, $CDCl_3$) δ 171.0, 158.2, 143.4, 141.0, 138.5, 136.1, 132.2, 131.4, 130.9, 130.5, 129.8, 129.5, 128.9, 128.8, 128.6, 128.2, 127.5, 126.9, 126.5, 126.4, 126.1, 122.8, 122.3, 113.1, 66.1, 54.8, 21.0; IR (Neat) ν_{max} 1734, 1603, 1439, 1223, 1018, 699 cm^{-1}; **HRMS (ESI)** for $C_{32}H_{26}O_3Na+$ $(M+Na)^+$: calcd. 481.1774, found 481.1783.

(E)-4-(4-Methoxyphenyl)-3,4-diphenylbut-3-en-2-yl acetate (27):

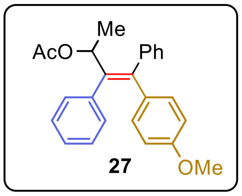

27 (75 mg, 67%) as pale yellow gummy liquid; R_f = 0.45 (10% EtOAc/Hex); ^{1}H NMR (500 MHz, DMSO d_6) δ 7.40 (t, J = 7.5 Hz, 2H), 7.34–7.22 (m, 5H), 7.18 (d, J = 7.0 Hz, 3H), 6.80 (dt, J = 7.0, 2.0 Hz, 2H), 6.59 (d, J = 9.0 Hz, 2H), 5.63 (q, J = 6.5 Hz, 1H), 3.59 (s, 3H), 1.88 (s, 3H), 1.20 (d, J = 6.5 Hz, 3H); ^{13}C NMR (126 MHz, DMSO d_6) δ 169.7, 158.0, 142.1, 138.6, 138.0, 134.3, 131.3, 131.2, 129.4, 128.8, 128.1, 127.6, 127.1, 113.4, 71.0, 55.3, 21.4, 19.8; IR (Neat) ν_{max} 1731, 1658, 1508, 1370, 1243, 1001, 704 cm^{-1}; **HRMS (ESI)** for $C_{25}H_{24}O_3Na+$ $(M+Na)^+$: calcd. 395.1618, found 395.1626.

(E)-4-(4-Methoxyphenyl)-4-phenyl-3-(*m*-tolyl)but-3-en-2-yl acetate (28):

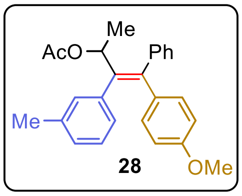

28 (71 mg, 61%) as pale yellow gummy liquid; R_f = 0.45 (10% EtOAc/Hex); ^{1}H NMR (500 MHz, DMSO d_6) δ 7.40 (t, J = 7.5 Hz, 2H), 7.31 (t, J = 7.5 Hz, 1H), 7.25 (d, J = 7.0 Hz, 2H), 7.12 (t, J = 7.5 Hz, 1H), 7.02–6.92 (m, 3H), 6.80 (d, J = 8.5 Hz, 2H), 6.59 (d, J = 8.5 Hz, 2H), 5.57 (q, J = 6.5 Hz, 1H), 3.59 (s, 3H), 2.33 (s, 3H), 1.88 (s, 3H), 1.20 (d, J = 6.5 Hz, 3H); ^{13}C NMR (126 MHz, DMSO d_6) δ 169.7, 157.9, 142.2, 141.8, 138.4, 138.0, 136.9, 134.4, 131.6, 131.2, 129.5, 129.3, 129.0, 128.7, 128.4, 127.9, 127.7, 127.5, 126.2, 113.4, 71.0, 55.3, 21.6, 21.4, 19.8; IR (Neat) ν_{max} 1735, 1650, 1244, 1022, 760 cm^{-1}; **HRMS (ESI)** for $C_{26}H_{26}O_3Na$ $(M+Na)^+$: calcd. 409.1774, found 409.1782.

(E)-4-(4-Methoxyphenyl)-4-phenyl-3-(3-(trifluoromethyl)phenyl)but-3-en-2-yl acetate (29/29′ = 90:10)

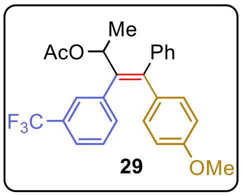

29 (62 mg, 47%) as pale yellow gummy liquid; R_f = 0.45 (10% EtOAc/Hex); ^{1}H NMR (500 MHz, DMSO d_6) δ 7.52–7.48 (m, 2H), 7.46 (br s, 1H), 7.42 (t, J = 7.0 Hz, 2H), 7.37–7.28 (m, 4H), 6.81 (d, J = 9.0 Hz, 2H), 6.62 (d, J = 9.0 Hz, 2H), 5.64 (q, J = 6.5 Hz, 1H), 3.60 (s, 3H), 1.91 (s, 3H), 1.21 (d, J = 6.5 Hz, 3H); ^{13}C NMR (126 MHz, DMSO d_6) δ 169.6, 158.2, 143.4, 141.6, 139.6, 136.7, 135.3, 133.8, 131.2, 129.22, 129.15, 128.8, 127.8, 124.6 (q, J = 272 Hz, 1C), 123.8 (q, J = 3.8 Hz, 1C), 113.5, 70.6, 55.3, 21.3, 19.7; ^{19}F NMR (471 MHz) δ –61.2; IR (Neat) ν_{max} 1730, 1605, 1508, 1232, 1114, 1040, 698 cm^{-1}; **HRMS (ESI)** for $C_{26}H_{23}F_3O_3Na$ $(M+Na)^+$: calcd. 463.1492, found 463.1498.

Representative peaks of the inseparable minor regioisomer **29′**: ^{1}H NMR (500 MHz, DMSO d_6) δ 3.68 (s, 0.32H).

(E)-3-(4-Bromophenyl)-4-(4-methoxyphenyl)-4-phenylbut-3-en-2-yl acetate (30):

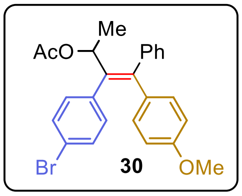

30 (105 mg, 78%) as pale yellow gummy liquid; R_f = 0.46 (10% EtOAc/Hex); ^{1}H NMR (500 MHz, $CDCl_3$) δ 7.44–7.27 (m, 7H), 7.10 (dd, J = 8.0, 1.5 Hz, 2H), 6.83 (d, J = 8.5 Hz, 2H), 6.59 (d, J = 9.0 Hz, 2H), 5.80 (q, J = 6.5 Hz, 1H), 3.70 (s, 3H), 1.94 (s, 3H), 1.29 (d, J = 6.5 Hz, 3H); ^{13}C NMR (126 MHz, $CDCl_3$) δ 169.8, 158.0, 142.9, 141.6, 137.8, 136.7, 133.8, 132.6, 131.4, 130.8, 129.1, 128.3, 127.2, 120.7, 112.9, 71.3, 55.0, 21.1, 19.6; IR (Neat) ν_{max} 1731, 1604, 1507, 1231, 1030, 1009, 701 cm^{-1}; **HRMS (ESI)** for $C_{25}H_{23}BrO_3Na$ $(M+Na)^+$: calcd. 473.0723, found 473.0724.

(E)-3-(3,4-Dichlorophenyl)-4-(4-methoxyphenyl)-4-phenylbut-3-en-2-yl acetate (31):

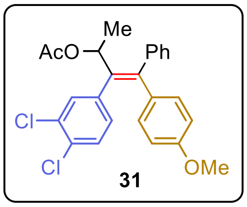

31 (92 mg, 63%) as pale yellow gummy liquid; R_f = 0.46 (10% EtOAc/Hex); ^{1}H NMR (500 MHz, DMSO d_6) δ 7.51 (d, J = 8.5 Hz, 1H), 7.46 (d, J = 1.0 Hz, 1H), 7.40 (d, J = 7.5 Hz, 2H), 7.35–7.29 (m, 1H), 7.26 (dd, J = 8.0, 1.0 Hz, 2H), 7.17 (dd, J = 8.0, 2.0 Hz, 1H), 6.83 (d, J = 9.0 Hz, 2H), 6.66 (d, J = 9.0 Hz, 2H), 5.60 (q, J = 6.5 Hz, 1H), 3.62 (s, 3H), 1.92 (s, 3H), 1.19 (d, J = 6.5 Hz, 3H); ^{13}C NMR (101 MHz, $CDCl_3$) δ 169.6, 158.3, 143.6, 139.5, 135.7, 133.7, 133.0, 131.8, 131.3, 130.7, 130.3, 129.9, 129.5, 129.2, 128.8, 127.8, 113.7, 70.5, 55.4, 21.4, 19.7; IR (Neat) ν_{max} 1734, 1603, 1507, 1369, 1231, 1028, 698 cm^{-1}; **HRMS (ESI)** for $C_{25}H_{22}Cl_2NaO_3$ + $(M+Na)^+$: calcd. 463.0838, found 463.0845.

(E)-3-(9-Ethyl-9H-carbazol-3-yl)-4-(4-methoxyphenyl)-4-phenylbut-3-en-2-yl acetate (32):

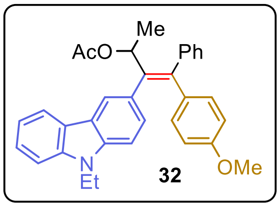

32 (110 mg, 75%) as pale yellow solid; R_f = 0.3 (10% EtOAc/Hex); ^{1}H NMR (500 MHz, DMSO d_6) δ 8.11 (d, J = 7.5 Hz, 1H), 7.98 (d, J = 1.5 Hz, 1H), 7.56 (d, J = 8.5 Hz, 1H), 7.47 (d, J = 8.5 Hz, 1H), 7.43 (t, J = 7.5 Hz, 3H), 7.36–7.28 (m, 3H), 7.22 (dd, J = 8.5, 1.5 Hz, 1H), 7.18 (t, J = 7.5 Hz, 1H), 6.84 (d, J = 9.0 Hz, 2H), 6.53 (d, J = 8.5 Hz, 2H), 5.66 (q, J = 6.0 Hz, 1H), 4.39 (q, J = 7.0 Hz, 2H), 3.52 (s, 3H), 1.94 (s, 3H), 1.30 (t, J = 7.5 Hz, 3H), 1.23 (d, J = 7.0 Hz, 3H); ^{13}C NMR (151 MHz, $CDCl_3$) δ 170.1, 157.5, 142.3, 141.9, 140.0, 138.8, 138.2, 134.5, 131.6, 129.4, 129.0, 128.2, 126.9, 125.5, 123.0, 122.4, 120.3, 118.6, 112.7, 108.4, 107.7, 72.1, 54.8, 37.5, 21.3, 19.8, 13.8; IR (Neat) ν_{max} 1728, 1507, 1231, 1034, 700 cm^{-1}; **HRMS (ESI)** for $C_{33}H_{31}NO_3Na+$ $(M+Na)^+$: calcd. 512.2196, found 512.2207.

(E)-Methyl 4-(3-acetoxy-2-(4-(benzyloxy)phenyl)-1-phenylbut-1-en-1-yl)benzoate (33):

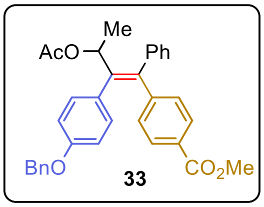

33 (88 mg, 58%) as pale yellow liquid; R_f = 0.23 (10% EtOAc/Hex); ^{1}H NMR (500 MHz, DMSO d_6) δ 7.65 (d, J = 8.5 Hz, 2H), 7.45–7.28 (m, 10H), 7.08 (d, J = 8.5 Hz, 2H), 7.05 (d, J = 8.5 Hz, 2H), 6.87 (d, J = 8.5 Hz, 2H), 5.60 (q, J = 6.5 Hz, 1H), 5.01 (s, 2H), 3.76 (s, 3H), 1.89 (s, 3H), 1.22 (d, J = 6.5 Hz, 3H); ^{13}C NMR (126 MHz, $CDCl_3$) δ 169.7, 166.3, 157.7, 147.5, 141.5, 141.1, 139.9, 137.4, 132.2, 130.3, 130.0, 129.4, 128.9, 128.8, 128.3, 127.8, 127.7, 114.4, 70.8, 69.6, 52.4, 21.3, 19.7; IR (Neat) ν_{max} 1716, 1605, 1506, 1368, 1274, 1232, 1017, 697 cm^{-1}; **HRMS (ESI)** for $C_{33}H_{30}O_5Na+$ $(M+Na)^+$: calcd. 529.1985, found 529.1992.

(E)-Methyl 4-(3-acetoxy-1-phenyl-2-(4-(phenylselanyl)phenyl)but-1-en-1-yl)benzoate (34):

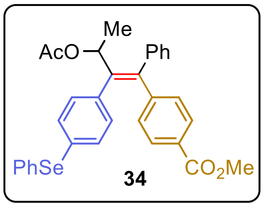

34 (55 mg, 33%) as pale yellow liquid; R_f = 0.31 (10% EtOAc/Hex); ^{1}H NMR (500 MHz, DMSO d_6) δ 7.67 (d, J = 8.5 Hz, 2H), 7.43 (t, J = 7.0 Hz, 2H), 7.38–7.21 (m, 10H), 7.14 (d, J = 8.5 Hz, 2H), 7.05 (d, J = 8.5 Hz, 2H), 5.63 (q, J = 6.5 Hz, 1H), 3.78 (s, 3H), 1.89 (s, 3H), 1.25 (d, J = 7.0 Hz, 3H); ^{13}C NMR (126 MHz, $CDCl_3$) δ 169.8, 166.3, 147.0, 142.1, 140.6, 139.5, 137.7, 133.1, 132.5, 132.1, 131.3, 130.3, 129.3, 129.0, 128.9, 128.7, 128.0, 127.9, 127.7, 70.4, 52.5, 21.3, 19.7; IR (Neat) ν_{max} 1716, 1605, 1476, 1435, 1273, 1231, 1104, 703 cm^{-1}; **HRMS (ESI)** for $C_{32}H_{28}O_4SeNa+$ $(M+Na)^+$: calcd. 579.1045, found 579.1053.

(E)-3-(4-Methoxyphenyl)-3-phenyl-2-(*p*-tolyl)allyl acetate (35):

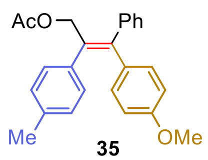

35

35 (57 mg, 51%) as pale red gummy liquid; R_f = 0.3 (10% EtOAc/Hex); ^{1}H NMR (400 MHz, Acetone d_6) δ 7.43–7.29 (m, 5H), 7.15 (d, J = 8.0 Hz, 2H), 7.03 (d, J = 7.6 Hz, 2H), 6.88 (d, J = 8.8 Hz, 2H), 6.66 (d, J = 8.4 Hz, 2H), 4.84 (s, 2H), 3.70 (s, 3H), 2.27 (s, 3H), 1.89 (s, 3H); ^{13}C NMR (101 MHz, Acetone d_6) δ 169.9, 158.4, 144.4, 142.5, 137.6, 136.0, 134.5, 133.4, 131.7, 129.7, 129.5, 128.6, 128.2, 127.3, 113.0, 66.4, 54.5, 20.3, 19.9; IR (Neat) ν_{max} 1734, 1508, 1232, 1018, 704 cm^{-1}; **HRMS (ESI)** for $C_{25}H_{24}NaO_3$+ (M+Na)$^+$: calcd. 395.1618, found 395.1626.

(Z)-3-(4-Methoxyphenyl)-4-phenyl-4-(*p*-tolyl)but-3-en-2-yl acetate (36):

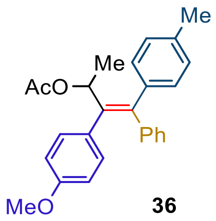

36

36 (101 mg, 87%) as pale yellow gummy liquid; R_f = 0.5 (10% EtOAc/Hex); ^{1}H NMR (500 MHz, DMSO d_6) δ 7.20 (d, J = 8.0 Hz, 2H), 7.15 (d, J = 8.0 Hz, 2H), 7.10–7.00 (m, 4H), 7.00–6.95 (m, 1H), 6.89 (d, J = 8.5 Hz, 2H), 6.77 (d, J = 9.0 Hz, 2H), 5.63 (q, J = 6.5 Hz, 1H), 3.68 (s, 3H), 2.31 (s, 3H), 1.90 (s, 3H), 1.20 (d, J = 7.0 Hz, 3H); ^{13}C NMR (126 MHz, DMSO d_6) δ 169.7, 158.3, 142.5, 142.4, 139.0, 138.3, 136.7, 132.3, 130.4, 130.0, 129..4, 129.2, 127.9, 126.5, 113.4, 71.0, 55.3, 21.4, 21.2, 19.8; IR (Neat) ν_{max} 1734, 1601, 1508, 1227, 1028, 692 cm^{-1}; **HRMS (ESI)** for $C_{26}H_{26}O_3Na$ (M+Na)$^+$: calcd. 409.1774, found 409.1782.

(Z)-3,4-Bis(4-Methoxyphenyl)-4-phenylbut-3-en-2-yl acetate (37):

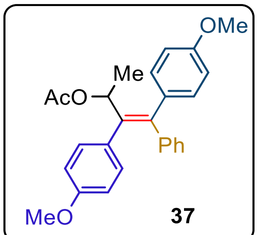

37

37 (75 mg, 62%) as pale yellow liquid; R_f = 0.29 (10% EtOAc/Hex); ^{1}H NMR (500 MHz, DMSO d_6) δ 7.17 (d, J = 9.0 Hz, 2H), 7.06 (d, J = 8.5 Hz, 2H), 7.03 (d, J = 7.5 Hz, 2H), 7.00–6.93 (m, 3H), 6.89 (d, J = 8.5 Hz, 2H), 6.77 (d, J = 9.0 Hz, 2H), 5.68 (q, J = 6.5 Hz, 1H), 3.77 (s, 3H), 3.68 (s, 3H), 1.91 (s, 3H), 1.20 (d, J = 6.5 Hz, 3H); ^{13}C NMR (126 MHz, DMSO d_6) δ 169.7, 158.7, 158.2, 142.8, 142.2, 138.3, 134.1, 132.3, 130.6, 130.5, 130.1, 127.9, 126.5, 114.2, 113.4, 71.0, 55.5, 55.3, 21.4, 19.8; IR (Neat) ν_{max} 1730, 1655, 1243, 10001, 822, 760 cm^{-1}; **HRMS (ESI)** for $C_{26}H_{26}O_4Na$ (M+Na)$^+$: calcd. 425.1723, found 425.1733.

3-(4-Methoxyphenyl)-4,4-diphenylbut-3-en-2-yl acetate (38):

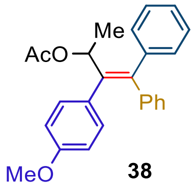

38

38 (95 mg, 85%) as pale yellow solid; R_f = 0.43 (10% EtOAc/Hex); M.P (147–148 ^{o}C); ^{1}H NMR (500 MHz, DMSO d_6) δ 7.40 (d, J = 7.5 Hz, 2H), 7.34–7.25 (m, 3H), 7.10–7.01 (m, 4H), 7.01–6.95 (m, 1H), 6.91 (d, J = 7.0 Hz, 2H), 6.78 (d, J = 8.5 Hz, 2H), 5.60 (q, J = 6.5 Hz, 1H), 3.68 (s, 3H), 1.89 (s, 3H), 1.20 (d, J = 6.5 Hz, 3H); ^{13}C

NMR (126 MHz, DMSO d_6) δ 169.7, 158.3, 142.5, 142.4, 141.9, 138.6, 132.2, 130.3, 129.9, 129.3, 128.8, 128.0, 127.5, 126.6, 113.5, 71.0, 55.3, 21.4, 19.8; IR (Neat) ν_{max} 1725, 1603, 1507, 1441, 1287, 1174, 692 cm^{-1}; **HRMS (ESI)** for $C_{25}H_{24}O_3Na$ $(M+Na)^+$: calcd. 395.1618, found 395.1627.

(Z)-4-(4-Fluorophenyl)-3-(4-methoxyphenyl)-4-phenylbut-3-en-2-yl acetate (39):

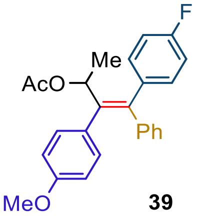

39

39 (101 mg, 86%) as pale yellow liquid; R_f = 0.43 (10% EtOAc/Hex); ^1H NMR (400 MHz, DMSO d_6) δ 7.35–7.29 (m, 2H), 7.28–7.19 (m, 2H), 7.11–6.98 (m, 5H), 6.94–6.88 (m, 2H), 6.78 (d, J = 7.6 Hz, 2H), 5.58 (q, J = 6.4 Hz, 1H), 3.69 (s, 3H), 1.91 (s, 3H), 1.21 (d, J = 6.8 Hz, 3H); ^{13}C NMR (101 MHz, DMSO d_6) δ 169.8, 161.7 (d, J = 244 Hz, 1C), 158.3, 142.2, 141.4, 139.0, 138.2 (d, J = 3.0 Hz, 1C), 132.2, 131.4 (d, J = 8.1 Hz, 2C), 130.1, 130.0, 128.1, 126.7, 115.7 (d, J = 21.2 Hz, 2C), 113.5, 70.9, 55.3, 21.4, 19.7; ^{19}F NMR (376 MHz) δ –115.3; IR (Neat) ν_{max} 1731, 1373, 1242, 1024, 1005, 757 cm^{-1}; **HRMS (ESI)** for $C_{25}H_{23}FO_3Na$ $(M+Na)^+$: calcd. 413.1523, found 413.1532.

(Z)-4-(4-Fluorophenyl)-3-(4-methoxyphenyl)-4-phenylbut-3-en-2-yl acetate (40):

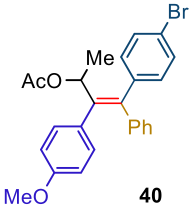

40

40 (107 mg, 79%) as pale yellow liquid; R_f = 0.43 (10% EtOAc/Hex); ^1H NMR (500 MHz, DMSO d_6) δ 7.60 (d, J = 8.5 Hz, 2H), 7.24 (d, J = 8.5 Hz, 2H), 7.09–7.03 (m, 4H), 7.02–6.97 (m, 1H), 6.90 (br d, J = 7.0 Hz, 2H), 6.78 (br d, J = 8.5 Hz, 2H), 5.56 (q, J = 6.5 Hz, 1H), 3.68 (s, 3H), 1.91 (s, 3H), 1.21 (d, J = 6.5 Hz, 3H); ^{13}C NMR (126 MHz, DMSO d_6) δ 169.7, 158.4, 141.9, 141.21, 141.15, 139.1, 132.1, 131.7, 131.6, 130.0, 128.1, 126.8, 120.9, 113.5, 70.9, 55.3, 21.4, 19.7; IR (Neat) ν_{max} 1735, 1569, 1325, 1022, 760 cm^{-1}; **HRMS (ESI)** for $C_{25}H_{23}BrO_3Na$ $(M+Na)^+$: calcd. 473.0723, found 473.0729.

(Z)-3-(4-Methoxyphenyl)-4-phenyl-4-(4-(trifluoromethyl)phenyl)but-3-en-2-yl acetate (41):

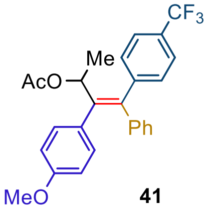

41

41 (107 mg, 81%) as pale yellow liquid; R_f = 0.39 (10% EtOAc/Hex); ^1H NMR (500 MHz, DMSO d_6) δ 7.77 (d, J = 8.0 Hz, 2H), 7.52 (d, J = 8.0 Hz, 2H), 7.11–6.98 (m, 5H), 6.92 (d, J = 7.0 Hz, 2H), 6.79 (d, J = 8.5 Hz, 2H), 5.49 (q, J = 6.5 Hz, 1H), 3.68 (s, 3H), 1.89 (s, 3H), 1.21 (d, J = 6.5 Hz, 3H); ^{13}C NMR (126 MHz, DMSO d_6) δ 169.8, 158.5, 146.2, 141.6, 141.0, 139.5, 132.1, 130.2, 130.0, 129.8, 128.2, 127.8 (q, J = 31.1 Hz, 1C), 126.9, 125.7 (q, J = 2.5 Hz, 1C), 124.7 (d, J = 273 Hz, 1C), 113.5, 70.9, 55.3, 21.3, 19.6; ^{19}F NMR (471 MHz) δ –61.0; IR (Neat) ν_{max} 1739, 1568, 1254, 1158, 1025, 701 cm^{-1}; **HRMS (ESI)** for $C_{26}H_{23}F_3O_3Na$ $(M+Na)^+$: calcd. 463.1492, found 463.1498.

(Z)-4-(4-Acetylphenyl)-3-(4-methoxyphenyl)-4-phenylbut-3-en-2-yl acetate (42):

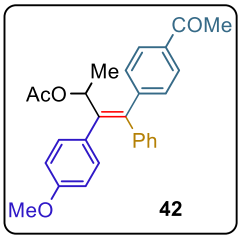

42

42 (104 mg, 83%) as pale yellow liquid; R_f = 0.15 (10% EtOAc/Hex); ^{1}H NMR (500 MHz, DMSO d$_6$) δ 8.00 (d, J = 8.5 Hz, 2H), 7.44 (d, J = 8.5 Hz, 2H), 7.12–6.96 (m, 5H), 6.92 (d, J = 7.0 Hz, 2H), 6.79 (d, J = 8.5 Hz, 2H), 5.55 (q, J = 6.5 Hz, 1H), 3.68 (s, 3H), 2.59 (s, 3H), 1.90 (s, 3H), 1.22 (d, J = 6.5 Hz, 3H); ^{13}C NMR (126 MHz, DMSO d$_6$) δ 197.9, 169.8, 158.4, 146.8, 141.7, 141.5, 139.2, 135.9, 132.2, 130.0, 129.9, 129.7, 128.8, 128.1, 126.9, 113.5, 70.9, 55.3, 27.1, 21.3, 19.7; IR (Neat) ν_{max} 1731, 1680, 1600, 1508, 1235, 1029, 705 cm^{-1}; **HRMS (ESI)** for $C_{27}H_{26}O_4Na$ (M+Na)$^+$: calcd. 437.1723, found 437.1731.

(Z)-Methyl 4-(3-acetoxy-2-(4-methoxyphenyl)-1-phenylbut-1-en-1-yl)benzoate (43):

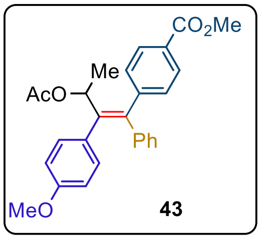

43

43 (115 mg, 89%) as pale yellow liquid; R_f = 0.23 (10% EtOAc/Hex); ^{1}H NMR (500 MHz, CDCl$_3$) δ 8.06 (dt, J = 8.5, 2.0 Hz, 2H), 7.39 (d, J = 8.5 Hz, 2H), 7.12–7.06 (m, 2H), 7.05–6.96 (m, 3H), 6.92–6.87 (m, 2H), 6.74 (dt, J = 9.0, 2.0 Hz, 2H), 5.70 (q, J = 6.5 Hz, 1H), 3.91 (s, 3H), 3.74 (s, 3H), 1.90 (s, 3H), 1.30 (d, J = 7.0 Hz, 3H); ^{13}C NMR (126 MHz, CDCl$_3$) δ 169.9, 166.8, 158.3, 146.7, 141.34, 141.32, 139.2, 131.8, 130.1, 129.6, 129.4, 128.8, 127.5, 126.3, 113.1, 71.4, 55.0, 52.0, 21.1, 19.6; IR (Neat) ν_{max} 1718, 1603, 1508, 1272, 1234, 1100, 707 cm^{-1}; **HRMS (ESI)** for $C_{27}H_{26}O_5Na$ (M+Na)$^+$: calcd. 453.1672, found 453.1681.

The compound **43** was insoluble in DMSO d$_6$. Thus, the spectra have been taken in CDCl$_3$. A little amount of cylization indene product **43′** has been observed in ^{1}H NMR during the recording of spectrum.

(Z)-4-(3-Cyanophenyl)-3-(4-methoxyphenyl)-4-phenylbut-3-en-2-yl acetate (44):

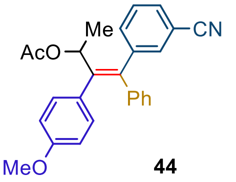

44

44 (98 mg, 82%) as pale yellow liquid; R_f = 0.19 (10% EtOAc/Hex); ^{1}H NMR (500 MHz, DMSO d$_6$) δ 7.83–7.77 (m, 2H), 7.65–7.59 (m, 2H), 7.10–7.05 (m, 4H), 7.04–6.99 (m, 1H), 6.96–6.91 (m, 2H), 6.79 (d, J = 8.5 Hz, 2H), 5.44 (q, J = 6.5 Hz, 1H), 3.69 (s, 3H), 1.91 (s, 3H), 1.20 (d, J = 7.0 Hz, 3H); ^{13}C NMR (126 MHz, DMSO d$_6$) δ 169.8, 158.5, 143.2, 141.5, 140.4, 139.9, 134.3, 132.7, 132.1, 131.4, 130.2, 130.0, 129.7, 128.2, 127.0, 119.1, 113.5, 111.9, 70.8, 55.3, 21.3, 19.5; IR (Neat) ν_{max} 1731, 1373, 1242m 1024, 1005, 611 cm^{-1}; **HRMS (ESI)** for $C_{26}H_{23}NO_3Na$ (M+Na)$^+$: calcd. 420.1570, found 420.1579.

(Z)-3-(4-Methoxyphenyl)-4-(3-nitrophenyl)-4-phenylbut-3-en-2-yl acetate (45):

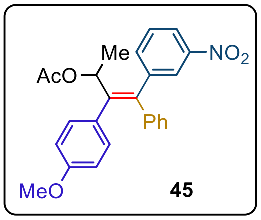

45 (117 mg, 93%) as pale yellow liquid; R_f = 0.18 (10% EtOAc/Hex); ^{1}H NMR (500 MHz, $CDCl_3$) δ 8.23–8.16 (m, 2H), 7.70 (d, J = 7.5 Hz, 1H), 7.58 (t, J = 7.5 Hz, 1H), 7.12 (d, J = 8.5 Hz, 2H), 7.10–7.01 (m, 3H), 6.96–6.91 (m, 2H), 6.78 (d, J = 9.0 Hz, 2H), 5.68 (q, J = 7.0 Hz, 1H), 3.70 (s, 3H), 1.95 (s, 3H), 1.34 (d, J = 7.0 Hz, 3H); ^{13}C NMR (126 MHz, $CDCl_3$) δ 169.9, 158.5, 148.1, 143.4, 140.8, 140.2, 140.1, 135.5, 131.6, 130.0, 129.6, 129.3, 127.7, 126.6, 124.1, 122.1, 113.2, 71.2, 55.0, 21.0, 19.5; IR (Neat) ν_{max} 1732, 1524, 1345, 1029, 701 cm^{-1}; **HRMS (ESI)** for $C_{25}H_{23}NO_5Na$ $(M+Na)^+$: calcd. 440.1468, found 440.1474.

(Z)-4-(3,5-Dinitrophenyl)-3-(4-methoxyphenyl)-4-phenylbut-3-en-2-yl acetate (46):

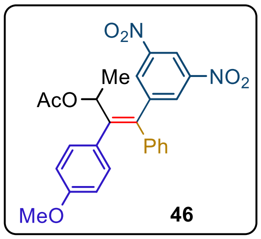

46 (115 mg, 83%) as pale yellow liquid; R_f = 0.19 (10% EtOAc/Hex); ^{1}H NMR (500 MHz, $CDCl_3$) δ 8.98 (t, J = 2.5 Hz, 1H), 8.51 (d, J = 2.0 Hz, 2H), 7.11–7.05 (m, 5H), 6.91–6.87 (m, 2H), 6.76 (d, J = 8.5 Hz, 2H), 5.66 (q, J = 6.5 Hz, 1H), 3.76 (s, 3H), 1.94 (s, 3H), 1.33 (d, J = 6.5 Hz, 3H); ^{13}C NMR (126 MHz, $CDCl_3$) δ 170.0, 158.8, 148.4, 145.5, 142.0, 139.7, 138.3, 131.5, 130.0, 129.6, 128.9, 128.1, 127.3, 117.4, 113.4, 70.9, 55.0, 20.9, 19.4; IR (Neat) ν_{max} 1731, 1535, 1341, 1234, 700 cm^{-1}; **HRMS (ESI)** for $C_{25}H_{22}N_2O_7Na$ $(M+Na)^+$: calcd. 485.1319, found 485.1327.

(Z)-4-(Benzo[d][1,3]dioxol-5-yl)-3-(4-methoxyphenyl)-4-phenylbut-3-en-2-yl acetate (47):

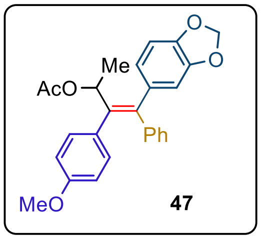

47 (83 mg, 66%) as pale yellow liquid; R_f = 0.21 (10% EtOAc/Hex); ^{1}H NMR (500 MHz, $CDCl_3$) δ 7.07 (dt, J = 6.5, 2.0 Hz, 2H), 7.05–6.96 (m, 3H), 6.94–6.89 (m, 2H), 6.83 (d, J = 8.0 Hz, 1H), 6.78 (dd, J = 7.5, 3.0 Hz, 1H), 6.75–6.70 (m, 3H), 5.96 (q, J = 1.5 Hz, 2H), 5.85 (q, J = 6.5 Hz, 1H), 3.74 (s, 3H), 1.95 (s, 3H), 1.30 (d, J = 6.5 Hz, 3H); ^{13}C NMR (126 MHz, $CDCl_3$) δ 170.0, 158.1, 147.5, 146.6, 142.13, 142.05, 138.4, 135.4, 131.9, 130.5, 130.0, 127.4, 126.1, 122.6, 113.0, 109.8, 108.1, 101.0, 71.5, 55.0, 21.2, 19.6; IR (Neat) ν_{max} 1733, 1604, 1507, 1468, 1240, 1032, 698 cm^{-1}; **HRMS (ESI)** for $C_{26}H_{24}O_5Na$ $(M+Na)^+$: calcd. 439.1516, found 439.1521.

(Z)-3-(4-Methoxyphenyl)-4-(naphthalen-2-yl)-4-phenylbut-3-en-2-yl acetate (48):

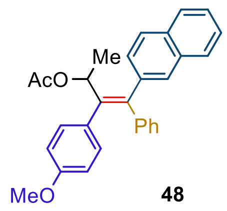

48

48 (104 mg, 82%) as pale yellow liquid; R_f = 0.45 (10% EtOAc/Hex); ^{1}H NMR (500 MHz, DMSO d_6) δ 7.99–7.86 (m, 4H), 7.58–7.51 (m, 2H), 7.37 (dd, J = 8.5, 1.5 Hz, 1H), 7.14 (d, J = 9.0 Hz, 2H), 7.05 (t, J = 7.0 Hz, 2H), 7.02–6.95 (m, 3H), 6.82 (dt, J = 7.5, 2.0 Hz, 2H), 5.68 (q, J = 6.5 Hz, 1H), 3.70 (s, 3H), 1.90 (s, 3H), 1.25 (d, J = 6.5 Hz, 3H); ^{13}C NMR (126 MHz, DMSO d_6) δ 169.8, 158.4, 142.4, 142.2, 139.4, 138.9, 133.3, 132.4, 132.3, 130.3, 130.2, 128.4, 128.3, 128.1, 127.9, 127.8, 126.8, 126.7, 126.6, 113.5, 71.1, 55.3, 21.4, 19.8; IR (Neat) ν_{max} 1739, 1604, 1507, 1368, 1232, 1031, 708 cm^{-1}; **HRMS (ESI)** for $C_{29}H_{26}O_3Na$ $(M+Na)^+$: calcd. 445.1774, found 445.1784.

(Z)-3-(4-Methoxyphenyl)-4-phenyl-4-(thiophen-2-yl)but-3-en-2-yl acetate (49):

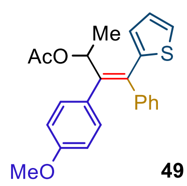

49

49 (71 mg, 62%) as pale yellow liquid; R_f = 0.42 (10% EtOAc/Hex); ^{1}H NMR (500 MHz, DMSO d_6) δ 7.58 (dd, J = 4.5, 1.5 Hz, 1H), 7.12–7.00 (m, 7H), 6.94 (d, J = 7.0 Hz, 2H), 6.75 (d, J = 8.5 Hz, 2H), 5.96 (q, J = 6.5 Hz, 1H), 3.67 (s, 3H), 1.94 (s, 3H), 1.27 (d, J = 6.5 Hz, 3H); ^{13}C NMR (126 MHz, DMSO d_6) δ 169.8, 158.3, 142.9, 142.3, 141.1, 135.2, 132.1, 130.0, 129.9, 128.1, 127.6, 127.4, 127.0, 113.4, 70.6, 55.3, 21.5, 19.9; IR (Neat) ν_{max} 1731, 1604, 1507, 1236, 1028, 695 cm^{-1}; **HRMS (ESI)** for $C_{23}H_{22}NaO_3S$ $(M+Na)^+$: calcd. 401.1182, found 401.1188.

2-(4-Methoxyphenyl)-1,1-diphenylhex-1-en-3-yl acetate (50):

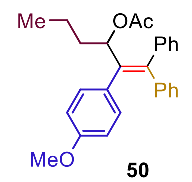

50

50 (105 mg, 87%) as pale yellow liquid; R_f = 0.43 (10% EtOAc/Hex); ^{1}H NMR (500 MHz, DMSO d_6) δ 7.42–7.35 (m, 2H), 7.32–7.26 (m, 3H), 7.07–7.02 (m, 4H), 7.00–6.95 (m, 1H), 6.94–6.89 (m, 2H), 6.76 (d, J = 9.0 Hz, 2H), 5.54 (dd, J = 8.0, 8.0 Hz, 1H), 3.68 (s, 3H), 1.89 (s, 3H), 1.59–1.48 (m, 2H), 1.26–1.11 (m, 2H), 0.69 (t, J = 7.5 Hz, 3H); ^{13}C NMR (126 MHz, DMSO d_6) δ 169.8, 158.3, 142.9, 142.5, 141.9, 138.0, 132.0, 130.5, 129.9, 129.3, 128.6, 128.0, 127.4, 126.5, 113.5, 74.2, 55.3, 35.5, 21.3, 18.8, 13.8; IR (Neat) ν_{max} 1731, 1373, 1242, 1023, 1004, 758 cm^{-1}; **HRMS (ESI)** for $C_{27}H_{28}O_3Na$ $(M+Na)^+$: calcd. 423.1931, found 423.1940.

1-(3-Methoxyphenyl)-2-(4-methoxyphenyl)-3,3-diphenylallyl acetate (51):

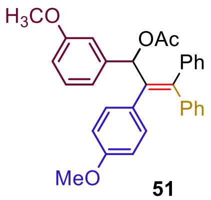

51

51 (86 mg, 62%) as pale yellow liquid; R_f = 0.40 (10% EtOAc/Hex); ^{1}H NMR (400 MHz, DMSO d$_6$) δ 7.43 (d, J = 4.8 Hz, 4H), 7.38–7.29 (m, 1H), 7.24 (t, J = 8.0 Hz, 1H), 7.11–6.94 (m, 5H), 6.85–6.76 (m, 4H), 6.70–6.62 (m, 4H), 3.69 (s, 3H), 3.63 (s, 3H), 2.04 (s, 3H); ^{13}C NMR (101 MHz, DMSO d$_6$) δ 169.7, 159.5, 158.2, 144.3, 142.4, 142.1, 140.7, 137.0, 132.2, 130.1, 130.0, 129.8, 129.4, 129.0, 128.2, 127.8, 126.8, 118.6, 113.2, 112.1, 75.3, 55.4, 55.2, 21.3; IR (Neat) ν_{max} 1739, 1604, 1508, 1227, 1030, 698 cm^{-1}; **HRMS (ESI)** for $C_{31}H_{25}O_4$ (M+H)$^+$: calcd. 465.2060, found 465.2062.

2-(4-Methoxyphenyl)-3,3-diphenyl-1-(3-(trifluoromethoxy)phenyl)allyl acetate (52):

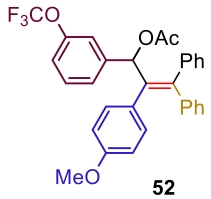

52

52 (103 mg, 66%) as pale yellow liquid; R_f = 0.45 (10% EtOAc/Hex); ^{1}H NMR (400 MHz, DMSO d$_6$) δ 7.43 (d, J = 7.6 Hz, 4H), 7.37–7.27 (m, 5H), 7.12–7.05 (m, 2H), 7.04–6.96 (m, 3H), 6.82 (dt, J = 6.8, 2.0 Hz, 2H), 6.69 (s, 1H), 6.64 (dt, J = 6.8, 2.0 Hz, 2H), 3.62 (s, 3H), 2.06 (s, 3H); ^{13}C NMR (101 MHz, DMSO d$_6$) δ 169.7, 158.3, 147.9, 144.9, 142.2, 141.9, 138.4, 136.5, 132.2, 129.9, 129.8, 129.3, 129.0, 128.4, 128.1, 127.8, 126.9, 121.2, 120.5 (d, J = 257 Hz, 1C), 113.3, 74.9, 55.2, 21.2; ^{19}F NMR (376 MHz) δ –56.8; IR (Neat) ν_{max} 1765, 1542, 1489, 1226, 1098, 1008, 698 cm^{-1}; **HRMS (ESI)** for $C_{31}H_{25}F_3NaO_4$ (M+Na)$^+$: calcd. 541.1603, found 541.1604.

1-(4-Bromophenyl)-2-(4-methoxyphenyl)-3,3-diphenylallyl acetate (53):

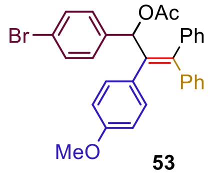

53

53 (88 mg, 57%) as pale yellow liquid; R_f = 0.45 (10% EtOAc/Hex); ^{1}H NMR (500 MHz, ACETONE d$_6$) δ 7.52 (d, J = 8.0 Hz, 2H), 7.47 (d, J = 8.5 Hz, 2H), 7.43 (t, J = 7.5 Hz, 2H), 7.38–7.31 (m, 1H), 7.20 (d, J = 8.5 Hz, 2H), 7.09–6.99 (m, 5H), 6.89 (d, J = 8.0 Hz, 2H), 6.80 (s, 1H), 6.65 (d, J = 8.5 Hz, 2H), 3.67 (s, 3H), 2.04 (s, 3H); ^{13}C NMR (126 MHz, ACETONE d$_6$) δ 169.0, 158.5, 144.6, 142.2, 141.9, 138.7, 136.8, 132.1, 131.1, 129.9, 129.3, 128.5, 128.4, 127.5, 127.3, 126.3, 120.8, 112.7, 75.1, 54.4, 20.1; IR (Neat) ν_{max} 1750, 1510, 1485, 1223, 1067, 1006, 700 cm^{-1}; **HRMS (ESI)** for $C_{30}H_{25}BrO_3Na$ + (M+Na)$^+$: calcd. 535.0879, found 535.0889.

1-(4-Chlorophenyl)-2-(4-methoxyphenyl)-3,3-diphenylallyl acetate (54):

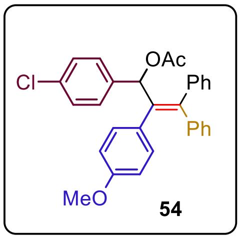

54 (79 mg, 56%) as pale yellow liquid; R_f = 0.45 (10% EtOAc/Hex); ^{1}H NMR (500 MHz, DMSO d_6) δ 7.42 (d, J = 4.5 Hz, 4H), 7.38–7.30 (m, 3H), 7.17 (d, J = 8.0 Hz, 2H), 7.10–7.04 (m, 2H), 7.03–6.95 (m, 3H), 6.81 (d, J = 9.0 Hz, 2H), 6.64 (d, J = 9.0 Hz, 3H), 3.62 (s, 3H), 2.05 (s, 3H); ^{13}C NMR (126 MHz, DMSO d_6) δ 169.6, 158.3, 144.8, 142.2, 141.9, 138.1, 136.5, 132.4, 132.2, 129.9, 129.8, 129.3, 129.0, 128.7, 128.3, 128.1, 127.8, 126.8, 113.3, 75.0, 55.2, 21.2; IR (Neat) ν_{max} 1734, 1605, 1508, 1368, 1226, 1029, 696 cm^{-1}; **HRMS (ESI)** for $C_{30}H_{25}ClO_3Na+$ $(M+Na)^+$: calcd. 491.1384, found 491.1381.

1-(3-Fluorophenyl)-2-(4-methoxyphenyl)-3,3-diphenylallyl acetate (55):

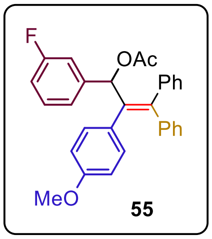

55 (72 mg, 53%) as pale yellow liquid; R_f = 0.45 (10% EtOAc/Hex); ^{1}H NMR (400 MHz, DMSO d_6) δ 7.47–7.41 (m, 4H), 7.39–7.31 (m, 2H), 7.11–6.97 (m, 7H), 6.88 (br d, J = 10.4 Hz, 1H), 6.83 (dt, J = 6.4, 2.0 Hz, 2H), 6.69 (s, 1H), 6.64 (d, J = 8.8 Hz, 2H), 3.62 (s, 3H), 2.07 (s, 3H); ^{13}C NMR (101 MHz, DMSO d_6) δ 169.7, 162.5 (d, J = 244 Hz, 1C), 158.3, 145.0, 142.2, 142.1 (d, J = 7.1 Hz, 1C), 141.9, 136.5, 132.2, 130.8 (d, J = 8.1 Hz, 1C), 130.0, 129.8, 129.3, 129.0, 128.2, 127.8, 126.9, 122.5 (d, J = 3.0 Hz, 1C), 114.7 (d, J = 21.2 Hz, 1C), 113.3, 113.1 (d, J = 23.2 Hz, 1C), 74.8, 55.2, 21.2; ^{19}F NMR (376 MHz) δ –112.8; IR (Neat) ν_{max} 1734, 1677, 1596, 1239, 1064, 703 cm^{-1}; **HRMS (ESI)** $C_{30}H_{25}FNaO_3+$ $(M+Na)^+$: calcd. 475.1680, found 475.1684.

1-(3,4-Dichlorophenyl)-2-(4-methoxyphenyl)-3,3-diphenylallyl acetate (56):

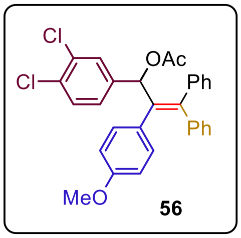

56 (107 mg, 71%) as pale yellow liquid; R_f = 0.42 (10% EtOAc/Hex); ^{1}H NMR (500 MHz, DMSO d_6) δ 7.56 (d, J = 8.5 Hz, 1H), 7.46–7.40 (m, 4H), 7.36–7.31 (m, 1H), 7.25–7.18 (m, 2H), 7.07 (t, J = 7.0 Hz, 2H), 7.03–6.96 (m, 3H), 6.84 (d, J = 8.5 Hz, 2H), 6.66 (d, J = 8.5 Hz, 2H), 6.62 (s, 1H), 3.62 (s, 3H), 2.06 (s, 3H); ^{13}C NMR (126 MHz, DMSO d_6) δ 169.6, 158.4, 145.3, 142.0, 141.7, 140.2, 136.1, 132.2, 131.5, 130.9, 130.6, 129.9, 129.6, 129.2, 129.0, 128.3, 128.1, 127.9, 126.94, 126.91, 113.4, 74.4, 55.3, 21.2, ; IR (Neat) ν_{max} 1690, 1580, 1254, 1005, 968, 705 cm^{-1}; **HRMS (ESI)** for $C_{30}H_{24}Cl_2NaO_3$ $(M+Na)^+$: calcd. 525.0995, found.525.0991.

2-(4-Methoxyphenyl)-1-(naphthalen-2-yl)-3,3-diphenylallyl acetate (57):

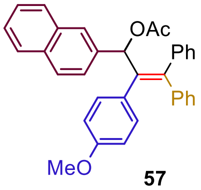

57

57 (105 mg, 72%) as pale yellow liquid; R_f = 0.41 (10% EtOAc/Hex); ^{1}H NMR (500 MHz, ACETONE d$_6$) δ 7.90–7.82 (m, 3H), 7.77 (s, 1H), 7.61 (d, J = 8.5 Hz, 2H), 7.52–7.42 (m, 5H), 7.38–7.32 (m, 1H), 7.11–6.99 (m, 6H), 6.91 (d, J = 8.5 Hz, 2H), 6.60 (d, J = 9.0 Hz, 2H), 3.63 (s, 3H), 2.09 (s, 3H); ^{13}C NMR (126 MHz, ACETONE d$_6$) δ 169.1, 158.4, 144.3, 142.5, 142.2, 137.2, 136.8, 133.2, 132.8, 132.1, 130.2, 130.0, 129.4, 128.5, 128.0, 127.8, 127.6, 127.3, 126.3, 126.2, 126.0, 125.1, 124.5, 112.7, 75.7, 54.4, 20.2; IR (Neat) ν_{max} 1735, 1508, 1224, 1027, 696 cm^{-1}; **HRMS (ESI)** for $C_{34}H_{28}NaO_3$ (M+Na)$^+$: calcd. 507.1931, found. 507.1930.

1-(1-(4-Methoxyphenyl)-2,2-diphenylvinyl)cyclohexyl acetate (58):

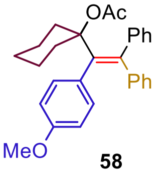

58

58 (65 mg, 51%) as pale yellow solid; R_f = 0.54 (10% EtOAc/Hex); M.P (220–222 °C); ^{1}H NMR (500 MHz, $CDCl_3$) δ 7.36–7.28 (m, 4H), 7.23–7.13 (m, 3H), 7.00–6.94 (m, 4H), 6.89–6.84 (m, 1H), 6.68 (d, J = 9.0 Hz, 2H), 3.72 (s, 3H), 2.57 (d, J = 10 Hz, 2H), 1.59–1.32 (m, 10H), 1.08–0.07 (m, 1H); ^{13}C NMR (126 MHz, $CDCl_3$) δ 169.7, 157.7, 144.8, 143.3, 142.8, 140.3, 132.9, 132.0, 129.0, 128.5, 127.8, 127.3, 125.9, 125.1, 112.4, 83.0, 54.9, 35.9, 24.8, 21.7, 21.6; IR (Neat) ν_{max} 1725, 1606, 1507, 1441, 1235, 1032, 695 cm^{-1}; **HRMS (ESI)** for $C_{29}H_{30}O_3Na$ (M+Na)$^+$: calcd. 449.2087, found 449.2090.

1-(1-(4-Methoxyphenyl)-2,2-diphenylvinyl)cyclobutyl acetate (59):

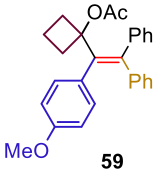

59

59 (89 mg, 74%) as pale yellow liquid; R_f = 0.54 (10% EtOAc/Hex); ^{1}H NMR (500 MHz, $CDCl_3$) δ 7.36–7.20 (m, 7H), 7.02–6.90 (m, 5H), 6.75 (d, J = 9.0 Hz, 2H), 3.76 (s, 3H), 2.58–2.49 (m, 2H), 2.36–2.28 (m, 2H), 1.92–1.81 (m, 1H), 1.66 (s, 3H), 1.49–1.38 (m, 1H); ^{13}C NMR (126 MHz, $CDCl_3$) δ 169.8, 158.0, 143.9, 143.4, 142.3, 139.6, 132.9, 132.2, 130.0, 129.0, 127.6, 127.2, 126.4, 125.4, 112.7, 84.8, 54.9, 36.2, 21.2, 15.6; IR (Neat) ν_{max} 1719, 1606, 1505, 1239, 1028, 698 cm^{-1}; **HRMS (ESI)** for $C_{27}H_{26}O_3Na$ (M+Na)$^+$: calcd. 421.1774, found 421.1780.

1-(1-(4-Methoxyphenyl)-2,2-diphenylvinyl)cyclododecyl acetate (60):

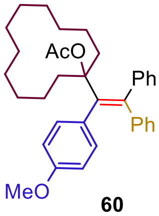

60

60 (48 mg, 31%) as pale yellow liquid; R_f = 0.54 (10% EtOAc/Hex);the compound is partially soluble in DMSO d$_6$ and thus ^{13}C NMR was recorded overnight; ^{1}H NMR (600 MHz, DMSO d$_6$) δ 7.28 (t, J = 8.4 Hz, 2H), 7.23 (d, J = 7.8 Hz, 2H), 7.18–7.00 (m, 3H), 6.95–6.86 (m, 4H), 6.79 (t, J = 6.6 Hz, 1H), 6.62 (d, J = 7.8 Hz, 2H), 3.58 (s, 3H), 1.94–1.82 (m, 2H), 1.79 (s, 3H), 1.37–1.04 (m, 20H); ^{13}C NMR (101 MHz, DMSO d$_6$) δ 169.6, 157.5, 145.2, 143.4, 142.7, 141.3, 132.8, 132.5, 128.8, 128.21, 128.16, 127.9, 126.8, 125.6, 112.6,

85.7, 55.1, 32.7, 26.8, 26.2, 22.3, 21.9, 21.8, 19.6; IR (Neat) ν_{max} 1735, 1650, 1594, 1022, 991, 611 cm^{-1}; **HRMS (ESI)** for $C_{35}H_{42}O_3Na$ $(M+Na)^+$: calcd. 533.3026, found 533.3027.

(*E*)-4-(4-Acetylphenyl)-3-(4-methoxyphenyl)-4-phenylbut-3-en-1-yl acetate (61):

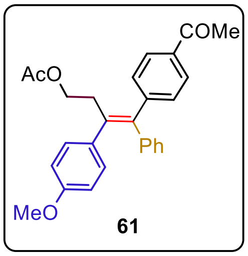

61

61 (77 mg, 62%) as pale yellow liquid; R_f = 0.54 (10% EtOAc/Hex); ^1H NMR (400 MHz, DMSO d_6) δ 8.00 (d, J = 8.4 Hz, 2H), 7.43 (d, J = 8.4 Hz, 2H), 7.12–7.01 (m, 5H), 6.93–6.87 (m, 2H), 6.77 (d, J = 8.8 Hz, 2H), 3.91 (t, J = 6.8 Hz, 2H), 3.69 (s, 3H), 2.72 (t, J = 7.2 Hz, 2H), 2.60 (s, 3H), 1.97 (s, 3H); ^{13}C NMR (101 MHz, DMSO d_6) δ 197.9, 170.6, 158.4, 148.1, 142.5, 140.3, 136.8, 135.7, 132.9, 130.9, 130.5, 129.9, 128.9, 128.3, 113.9, 62.5, 55.4, 34.8, 27.1, 21.1; IR (Neat) ν_{max} 1734, 1679, 1599, 1508, 1359, 1233, 1174, 1028, 699 cm^{-1}; **HRMS (ESI)** for $C_{27}H_{26}NaO_4+$ $(M+Na)^+$: calcd. 437.1723, found 437.1733.

((Z)-Methyl 4-(5-acetoxy-2-(4-methoxyphenyl)-1-phenylpent-1-en-1-yl)benzoate (62/62′ = 93:7)

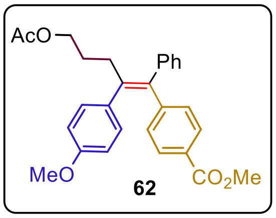

62

62 (mg, 48%) as pale yellow liquid; R_f = 0.54 (10% EtOAc/Hex); ^1H NMR (400 MHz, DMSO d_6) δ 7.66 (d, J = 8.4 Hz, 2H), 7.40 (t, J = 7.2 Hz, 2H), 7.35–7.28 (m, 1H), 7.25–7.20 (m, 2H), 7.05 (d, J = 8.8 Hz, 2H), 7.02 (d, J = 8.4 Hz, 2H), 6.76 (d, J = 8.8 Hz, 2H), 3.87 (t, J = 6.4 Hz, 2H), 3.77 (s, 3H), 3.69 (s, 3H), 2.44–2.40 (m, 2H), 1.90 (s, 3H), 1.61–1.47 (m, 2H); ^{13}C NMR (101 MHz, DMSO d_6) δ 170.7, 166.4, 158.4, 148.5, 142.7, 141.1, 138.5, 133.2, 130.9, 129.5, 129.0, 128.9, 127.5, 127.4, 114.0, 63.8, 55.4, 52.4, 32.1, 27.9, 21.1; IR (Neat) ν_{max} 1717, 1605, 1508, 1276, 1242, 1023, 992, 703 cm^{-1}; **HRMS (ESI)** for $C_{28}H_{28}NaO_5+$ $(M+Na)^+$: calcd. 467.1829, found 467.1836.

Representative peaks of the inseparable minor regio-isomer **62′**: ^1H NMR (400 MHz, DMSO d_6) δ 3.82 (s, 0.23H), 3.62 (s, 0.23H); ^{13}C NMR (101 MHz, DMSO d_6) δ 131.8, 128.8, 127.8.

(E)-6-(4-Acetylphenyl)-5-(4-methoxyphenyl)-6-phenylhex-5-en-1-yl acetate (63/63′ = 92:8):

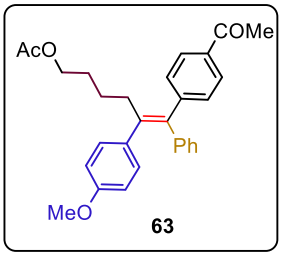

63

63 (67 mg, 51%) as pale yellow liquid; R_f = 0.54 (10% EtOAc/Hex); ^1H NMR (500 MHz, ACETONE d_6) δ 8.02 (d, J = 8.0 Hz, 2H), 7.43 (d, J = 8.5 Hz, 2H), 7.12 (d, J = 9.0 Hz, 2H), 7.10–7.00 (m, 3H), 6.99–6.94 (m, 2H), 6.76 (d, J = 9.0 Hz, 2H), 3.91 (t, J = 65 Hz, 2H), 3.73 (s, 3H), 2.60 (s, 3H), 2.49 (t, J = 7.5 Hz, 2H), 1.93 (s, 3H), 1.59–1.51 (m, 2H), 1.49–1.40 (m, 2H); ^{13}C NMR (126 MHz, ACETONE d_6) δ 196.6, 170.0, 158.4, 148.5, 142.8, 141.0, 138.3, 135.7, 133.7, 130.6, 130.5, 129.6, 128.3, 127.6, 126.0, 113.3, 63.5, 54.5, 35.1, 25.8, 24.9, 19.9; IR (Neat) ν_{max} 1740, 1636, 1569, 1288, 1189, 1056, 956, 699 cm^{-1}; **HRMS (ESI)** for $C_{29}H_{30}O_4Na$ $(M+Na)^+$: calcd. 465.2036, found. 465.2037.

Representative peaks of the inseparable minor regio-isomer **63′**: ^{1}H NMR (500 MHz, DMSO d$_6$) δ 6.85 (d, J = 8.5 Hz, 0.18H), 6.62 (d, J = 9.0 Hz, 0.19H), 3.66 (s, 0.28H); ^{13}C NMR (126 MHz, DMSO d$_6$) δ 158.1, 142.1, 140.6, 134.6, 131.7, 113.0.

(*E*)-2-(4-Methoxyphenyl)-3-phenylhex-2-en-1-yl benzoate (64/64′ = 88:12):

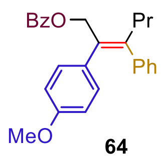

64

64 (10 mg, 9%) as pale yellow liquid; R$_f$ = 0.53 (10% EtOAc/Hex); ^{1}H NMR (500 MHz, DMSO d$_6$) δ 7.85–7.79 (m, 2H), 7.65–7.59 (m, 1H), 7.49 (t, J = 8.0 Hz, 2H), 7.14 (t, J = 7.5 Hz, 2H), 7.10–7.05 (m, 1H), 7.04–7.00 (m, 2H), 7.97 (d, J = 8.5 Hz, 2H), 6.65 (d, J = 9.0 Hz, 2H), 5.27 (s, 2H), 3.62 (s, 3H), 2.66 (t, J = 7.5 Hz, 2H), 1.33–1.26 (m, 2H), 0.86 (t, J = 7.5 Hz, 3H); ^{13}C NMR (101 MHz, DMSO d$_6$) δ 160.0,, 157.8, 144.1, 142.6, 133.8, 133.3, 132.4, 131.0, 130.2, 129.47, 129.45, 129.2, 128.2, 113.5, 65.5, 55.3, 36.6, 21.7, 14.0; IR (Neat) ν_{max} 1718, 159, 1459, 1267, 1246, 711 cm^{-1}; **HRMS (ESI)** for $C_{26}H_{26}O_3Na$ (M+Na)$^+$: calcd. 409.1774, found 409.1777.

Representative peaks of the inseparable minor regio-isomer **64′**: ^{1}H NMR (500 MHz, DMSO d$_6$) δ 3.65 (s, 0.43H); ^{13}C NMR (126 MHz, DMSO d$_6$) δ 158.0, 144.2, 129.4, 128.2, 16.5, 124.6, 115.0, 113.6, 55.3, 21.7.

2-(4-Methoxyphenyl)-1,3,3-triphenylallyl benzoate (66):

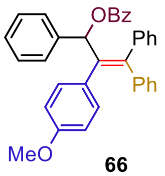

66

66 (83 mg, 56%) as pale yellow liquid; R$_f$ = 0.43 (10% EtOAc/Hex); ^{1}H NMR (500 MHz, DMSO d$_6$) δ 7.89 (d, J = 8.0 Hz, 2H), 7.67 (t, J = 7.5 Hz, 1H), 7.53 (t, J = 8.0 Hz, 2H), 7.50–7.41 (m, 4H), 7.37–7.30 (m, 3H), 7.27 (t, J = 7.0 Hz, 1H), 7.21 (d, J = 7.5 Hz, 2H), 7.08 (t, J = 7.5 Hz, 2H), 7.04–6.96 (m, 4H), 6.83 (dt, J = 7.0, 1.5 Hz, 2H), 6.64 (d, J = 8.5 Hz, 2H), 3.62 (s, 3H); ^{13}C NMR (101 MHz, DMSO d$_6$) δ 165.0, 158.3, 144.7, 142.3, 142.0, 139.0, 136.7, 134.1, 132.2, 130.0, 129.7, 129.4, 129.1, 128.9, 128.2, 128.0, 127.8, 126.9, 126.3, 113.3, 76.2, 55.3; IR (Neat) ν_{max} 1716, 1604, 1508, 1242, 1174, 1025, 748, 695 cm^{-1}; **HRMS (ESI)** for $C_{35}H_{28}NaO_3$ (M+Na)$^+$: calcd. 519.1931, found 519.1934.

(3-(Benzyloxy)-2-(4-methoxyphenyl)prop-1-ene-1,1,3-triyl)tribenzene (68):

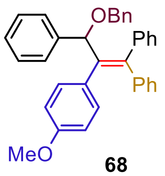

68

68 (61 mg, 42%) as pale yellow liquid; R$_f$ = 0.51 (10% EtOAc/Hex); ^{1}H NMR (500 MHz, DMSO d$_6$) δ 7.44–7.36 (m, 4H), 7.33–7.24 (m, 10H), 7.21–7.16 (m, 1H), 7.08 (d, J = 7.5 Hz, 2H), 7.05–6.99 (m, 3H), 6.86 (d, J = 8.5 Hz, 2H), 6.56 (d, J = 9.0 Hz, 2H), 5.44 (s, 1H), 4.80 (d, J = 8.0 Hz, 1H), 4.45 (d, J = 7.0 Hz, 1H), 3.59 (s, 3H); ^{13}C NMR (101 MHz, DMSO d$_6$) δ 158.0, 145.0, 142.8, 142.2, 140.9, 138.8, 138.0, 132.3, 130.5, 130.2, 129.6, 129.0, 128.7, 128.4, 128.1, 127.93, 127.89, 127.6, 127.3, 126.7, 126.6, 113.0, 79.7, 69.8, 55.2; IR (Neat)

ν_{max} 1604, 1507, 1491, 1441, 1242, 1027, 747 cm^{-1}; **HRMS (ESI)** for $C_{35}H_{30}NaO_2$ $(M+Na)^+$: calcd. 505.2143, found 505.2151.

2-(4-Methoxyphenyl)-1,3,3-triphenylprop-2-en-1-ol (69):

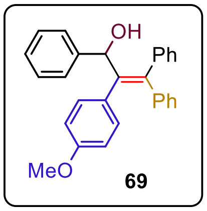

69 (47 mg, 41%) as pale yellow liquid; R_f = 0.51 (10% EtOAc/Hex); ^{1}H NMR (500 MHz, DMSO d_6) δ 7.48 (dd, J = 8.5, 1.5 Hz, 2H), 7.43 (t, J = 7.5 Hz, 2H), 7.34–7.28 (m, 1H), 7.23 (d, J = 4.5 Hz, 4H), 7.17–7.12 (m, 1H), 7.05 (t, J = 7.0 Hz, 2H), 7.00–6.92 (m, 3H), 6.77 (d, J = 9.0 Hz, 2H), 6.52 (d, J = 9.0 Hz, 2H), 5.65 (d, J = 3.5 Hz, 1H), 5.56 (d, J = 3.5 Hz, 1H), 3.58 (s, 3H); ^{13}C NMR (126 MHz, DMSO d_6) δ 157.8, 143.7, 143.0, 142.9, 142.7, 141.6, 132.5, 130.9, 130.2, 129.7, 128.9, 128.1, 128.0, 127.3, 126.7, 126.4, 126.3, 112.6, 72.2, 55.1; IR (Neat) ν_{max} 3413, 2923,. 1604, 1508, 1443, 1244, 699 cm^{-1}; **HRMS (ESI)** for $C_{28}H_{24}O_2Na$ $(M+Na)^+$: calcd. 415.1669, found 415.1670.

2-(2-(4-Methoxyphenyl)-3,3-diphenylallyl)isoindoline-1,3-dione (71):

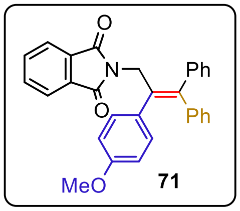

71 (47 mg, 41%) as pale yellow liquid; R_f = 0.51 (10% EtOAc/Hex); ^{1}H NMR (500 MHz, DMSO d_6) δ 7.69–7.64 (m, 2H), 7.62–7.55 (m, 4H), 7.35 (t, J = 8.0 Hz, 2H), 7.26–7.21 (m, 1H), 7.09–6.93 (m, 7H), 6.58 (d, J = 9.0 Hz, 2H), 4.75 (s, 2H), 3.63 (s, 3H); ^{13}C NMR (101 MHz, DMSO d_6) δ 168.0, 158.1, 142.5, 142.4, 141.4, 133.7, 133.6, 131.7, 130.82, 130.76, 130.3, 129.5, 128.4, 127.5, 126.8, 126.0, 122.9, 113.2, 54.9, 42.0; IR (Neat) ν_{max} 1711, 1606, 1509, 1390, 1245, 701 cm^{-1}; **HRMS (ESI)** for $C_{30}H_{24}NO_3$ $(M+Na)^+$: calcd. 446.1751, found 446.1757.

(S)-2,8-Dimethyl-2-((4S,8S)-4,8,12-trimethyltridecyl)chroman-6-yl 4-((Z)-3-acetoxy-2-(4-methoxyphenyl)-1-phenylbut-1-en-1-yl)benzoate (96):

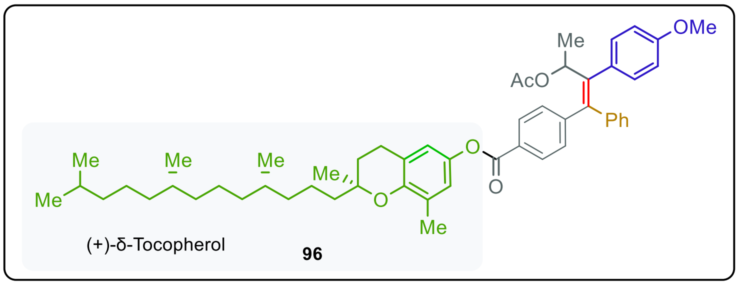

96 (115 mg, 72%, 0.2 mmol scale) as pale yellow liquid; R_f = 0.86 (20% EtOAc/Hex);. ^{1}H NMR (500 MHz, ACETONE D_6) δ 8.19 (d, J = 8.0 Hz, 2H), 7.57 (d, J = 8.0 Hz, 2H), 7.19 (d, J = 8.5 Hz, 2H), 7.12–6.99 (m, 5H), 6.91–6.78 (m, 4H), 5.71 (q, J = 6.5 Hz, 1H), 3.75 (s, 3H), 2.88–2.78 (m, 4H), 2.18 (s, 3H), 1.92 (s, 3H), 1.91–1.80 (m, 2H), 1.70–1.34 (m, 11H), 1.31–1.01 (m, 13H), 0.95–0.84 (m, 13H); ^{13}C NMR (126 MHz, ACETONE d_6) δ 169.1, 164.8, 158.7, ,149.6, 147.4, 143.2, 141.7, 141.6, 139.7, 132.0, 130.0, 129.8, 129.7, 128.7, 127.6, 126.6, 126.4, 121.4, 121.1, 119.5, 113.0, 76.1, 70.9, 54.5, 39.8, 39.2, 37.32, 37.30, 37.2, 37.1, 32.7, 32.5,

30.9, 27.8, 24.7, 24.3, 23.7, 22.2, 22.13, 22.09, 20.8, 20.2, 19.3, 19.2, 19.0, 15.4; IR (Neat) ν_{max} 1732, 1604, 1509, 1470, 1263, 1241, 1176, 1074, 733 cm^{-1}; **HRMS (ESI)** for $C_{53}H_{68}O_6Na$ $(M+Na)^+$: calcd. 823.4908, found 823.4908.

(3R,8R,9R,10S,13S,14R,17S)-10,13-Dimethyl-17-((S)-6-methylheptan-2-yl)-2,3,4,7,8,9,10,11,12,13,14,15,16,17-tetradecahydro-1H-cyclopenta[a]phenanthren-3-yl 4-((Z)-3-acetoxy-2-(4-methoxyphenyl)-1-phenylbut-1-en-1-yl)benzoate (97):

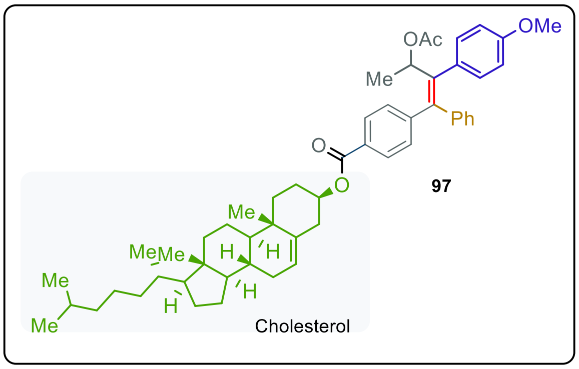

97 (71 mg, 45%, 0.2 mmol scale) as pale yellow liquid; R_f = 0.86 (20% EtOAc/Hex); ^{1}H NMR (500 MHz, ACETONE d_6) δ 8.07 (d, J = 8.5 Hz, 2H), 7.49 (d, J = 8.5 Hz, 2H), 7.17 (dt, J = 7.0, 2.0 Hz, 2H), 7.09–6.97 (m, 5H), 6.79 (d, J = 8.5 Hz, 2H), 5.68 (q, J = 6.5 Hz, 1H), 5.47 (d, J = 4.5 Hz, 1H), 4.88–4.78 (m, 1H), 3.74 (s, 3H), 2.90 (d, J = 16.5 Hz, 4H), 2.57–6.47 (m, 2H), 2.05–1.95 (m, 3H), 1.90 (s, 3H), 1.70–1.50 (m, 7H), 1.48–1.40 (m, 2H), 1.29 (d, J = 6.5 Hz, 3H), 1.28–1.16 (m, 7H), 1.13 (s, 3H), 1.12–0.92 (m, 3H), 0.99 (d, J = 7.0 Hz, 3H), 0.89 (dd, J = 6.5, 1.5 Hz, 6H), 0.76 (s, 3H); ^{13}C NMR (126 MHz, ACETONE d_6) δ 169.1, 165.1, 158.6, 146.8, 141.7, 141.5, 139.8, 139.5, 132.0, 130.0, 129.9, 129.5, 129.4, 129.3, 127.5, 126.3, 122.5, 113.0, 74.4, 70.9, 56.7, 56.2, 54.5, 50.2, 42.3, 39.8, 39.4, 38.1, 37.0, 36.6, 36.1, 35.8, 31.9, 31.8, 27.8, 27.7, 24.1, 23.7, 22.2, 22.0, 20.9, 20.2, 18.93, 18.86, 18.3, 11.4; IR (Neat) ν_{max} 1736, 1714, 1270, 1239, 1176, 1110, 1032, 760 cm^{-1}; **HRMS (ESI)** for $C_{53}H_{68}O_5Na$ $(M+Na)^+$: calcd. 807.4959, found 807.4959.

(E)-3,7-Dimethylocta-2,6-dien-1-yl 4-((Z)-3-acetoxy-2-(4-methoxyphenyl)-1-phenylbut-1-en-1-yl)benzoate (98):

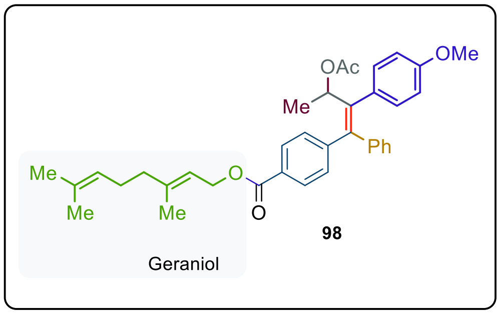

98 (60 mg, 54%, 0.2 mmol scale) as pale yellow liquid; R_f = 0.77 (20% EtOAc/Hex); ^{1}H NMR (500 MHz, DMSO d_6) δ 7.97 (d, J = 8.5 Hz, 2H), 7.42 (d, J = 8.5 Hz, 2H), 7.11–6.98 (m, 5H), 6.92–6.87 (m, 2H), 6.78 (d, J = 9.0 Hz, 2H), 5.51 (q, J = 6.5 Hz, 1H), 5.46–5.41 (m, 1H), 5.09–5.04 (m, 1H), 4.80 (d, J = 7.0 Hz, 2H), 3.68 (s, 3H), 2.12–2.00 (m, 4H), 1.88 (s, 3H), 1.73 (s, 3H), 1.60 (s, 3H), 1.54 (s, 3H), 1.19 (d, J = 7.0 Hz, 3H); ^{13}C NMR (126 MHz, DMSO d_6) δ 169.7, 165.9, 158.4, 146.9, 142.4, 141.6, 141.4, 139.3, 132.1, 131.5, 130.0, 129.8, 129.7, 129.1, 128.1, 126.9, 124.1, 118.9, 113.5, 70.9, 61.8, 55.3, 26.2, 25.9, 21.3, 19.6, 18.0, 16.7; IR (Neat) ν_{max} 1734, 1714,

1604, 1508, 1441, 1265, 1239, 1095, 1033, 706 cm^{-1}; **HRMS (ESI)** for $C_{36}H_{40}O_5Na$ $(M+Na)^+$: calcd. 575.2768, found 575.2768.

(Z)-4-(3-Acetoxy-2-(4-methoxyphenyl)-1-phenylbut-1-en-1-yl)phenyl 2-propylpentanoate (99):

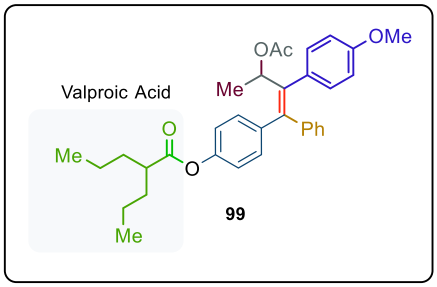

99 (67 mg, 65%, 0.2 mmol scale) as pale yellow liquid; R_f = 0.5 (10% EtOAc/Hex); ^{1}H NMR (500 MHz, DMSO d_6) δ 7.97 (dt, *J* = 8.5, 2.0 Hz, 2H), 7.10 (dt, *J* = 8.5, 2.0 Hz, 2H), 7.09–7.02 (m, 4H), 7.01–6.96 (m, 1H), 6.93–6.88 (m, 2H), 6.77 (dt, *J* = 9.0, 2.0 Hz, 2H), 5.58 (q, *J* = 6.5 Hz, 1H), 3.67 (s, 3H), 2.65–2.56 (m, 1H), 1.89 (s, 3H), 1.70–1.61 (m, 2H), 1.58–1.50 (m, 2H), 1.43–1.34 (m, 4H), 1.21 (d, *J* = 7.0 Hz, 3H), 0.92 (t, *J* = 7.5 Hz, 6H); ^{13}C NMR (126 MHz, DMSO d_6) δ 174.7, 169.8, 158.3, 149.8, 142.2, 141.5, 139.4, 139.0, 132.2, 130.5, 130.2, 130.0, 128.0, 126.7, 122.0, 113.5, 71.0, 55.3, 45.0, 34.6, 21.3, 20.6, 19.7, 14.3; IR (Neat) ν_{max} 1735, 1507, 1263, 1241, 1197, 1107, 731, 701 cm^{-1}; **HRMS (ESI)** for $C_{33}H_{38}O_5Na$ $(M+Na)^+$: calcd. 537.2611, found 537.2611.

4-((Z)-3-acetoxy-2-(4-methoxyphenyl)-1-phenylbut-1-en-1-yl)phenyl (2R)-2-(6-methoxynaphthalen-2-yl)propanoate (100):

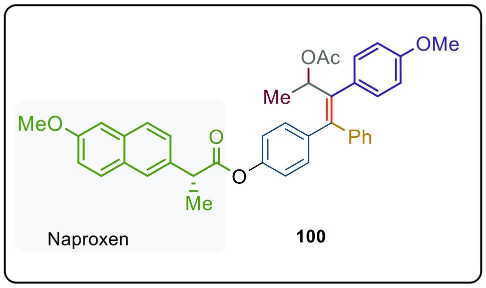

100 (61 mg, 51%, 0.2 mmol scale) as pale yellow liquid; R_f = 0.68 (20% EtOAc/Hex); ^{1}H NMR (500 MHz, DMSO d_6) δ 7.89–7.83 (m, 3H), 7.52 (dd, *J* = 8.5, 1.5 Hz, 1H), 7.32 (d, *J* = 2.5 Hz, 1H), 7.27 (dt, *J* = 8.5, 1.5 Hz, 2H), 7.18 (dd, *J* = 9.0, 2.5 Hz, 1H), 7.09–7.01 (m, 6H), 7.00–6.94 (m, 1H), 6.91–6.85 (m, 2H), 6.76 (d, *J* = 9.0 Hz, 2H), 5.55 (q, *J* = 5.0 Hz, 1H), 4.22 (q, *J* = 7.0 Hz, 1H), 3.87 (s, 3H), 3.67 (s, 3H), 1.87 (s, 3H), 1.60 (d, *J* = 7.0 Hz, 3H), 1.18 (dd, *J* = 7.0, 1.5 Hz, 3H); ^{13}C NMR (126 MHz, DMSO d_6) δ 173.2, 169.7, 158.3, 157.8, 149.9, 142.2, 141.50, 141.48, 139.4, 139.0, 135.7, 134.0, 132.2, 130.4, 130.2, 129.9, 129.7, 129.0, 128.0, 127.7, 126.7, 126.3, 121.8, 119.3, 113.5, 106.3, 71.0, 55.7, 55.3, 45.0, 21.3, 19.7, 18.8; IR (Neat) ν_{max} 1733, 1605, 1507, 1369, 1263, 1240, 1197, 1030, 731, 700cm^{-1}; **HRMS (ESI)** for $C_{39}H_{36}O_6Na$ $(M+Na)^+$: calcd. 623.2404, found 623.2404.

(Z)-1,2-bis(4-methoxyphenyl)-1-phenylhex-1-en-3-yl acetate (112):

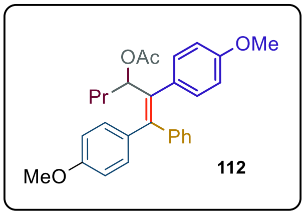

112 (73 mg, 57%) as pale yellow liquid; R_f = 0.77 (20% EtOAc/Hex); ^{1}H NMR (500 MHz, DMSO d_6) δ 7.16 (d, *J* = 8.5 Hz, 2H), 7.06–7.00 (m, 4H), 6.99–6.92 (m, 3H), 6.89–6.85 (m, 2H), 6.75 (d, *J* = 8.5 Hz, 2H), 5.59 (t, *J* = 7.0 Hz, 1H), 3.76 (s, 3H), 3.67 (s, 3H), 1.91 (s, 3H), 1.51 (q, *J* = 7.5 Hz, 2H), 1.25–1.12 (m,

2H), 0.72 (t, J = 7.0 Hz, 3H); ^{13}C NMR (126 MHz, DMSO d$_6$) δ 170.0, 158.6, 158.2, 142.9, 142.8, 137.8, 134.2, 132.1, 130.7, 130.6, 130.0, 127.9, 126.5, 114.0, 113.4, 74.2, 55.5, 55.3, 35.5, 21.3, 18.8, 13.9; IR (Neat) ν_{max} 1733, 1606, 1284, 1240, 1175, 1032, 736 cm^{-1}; **HRMS (ESI)** for $C_{28}H_{30}O_4Na$ (M+Na)$^+$: calcd. 453.2036, found 453.2032.

(Z)-1-(4-acetylphenyl)-2-(4-methoxyphenyl)-1-phenylhex-1-en-3-yl acetate (113):

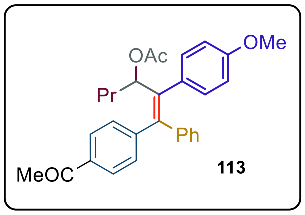

113 (98 mg, 74%) as pale yellow liquid; R$_f$ = 0.64 (20% EtOAc/Hex); ^{1}H NMR (500 MHz, DMSO d$_6$) δ 7.98 (d, J = 8.5 Hz, 2H), 7.43 (d, J = 8.5 Hz, 2H), 7.09–7.03 (m, 4H), 7.02–6.96 (m, 1H), 6.93–6.88 (m, 2H), 6.77 (d, J = 9.0 Hz, 2H), 5.47 (t, J = 7.0 Hz, 1H), 3.67 (s, 3H), 2.58 (s, 3H), 1.90 (s, 3H), 1.54 (q, J = 7.5 Hz, 2H), 1.25–1.12 (m, 2H), 0.70 (t, J = 7.5 Hz, 3H); ^{13}C NMR (126 MHz, DMSO d$_6$) δ 198.1, 170.0, 158.4, 146.9, 142.1, 141.9, 138.6, 135.9, 132.0, 130.2, 130.0, 129.8, 128.7, 128.2, 126.9, 113.6, 74.2, 55.3, 27.2, 21.3, 18.8, 13.9; IR (Neat) ν_{max} 1733, 1680, 1601, 1508, 1239, 1176, 1023, 992, 600 cm^{-1}; **HRMS (ESI)** for $C_{29}H_{30}O_4Na$ (M+Na)$^+$: calcd. 465.2036, found 465.2037.

Supplementary Table 4: Reaction optimization for aryl-alkenylation of unsymmetrical alkyne

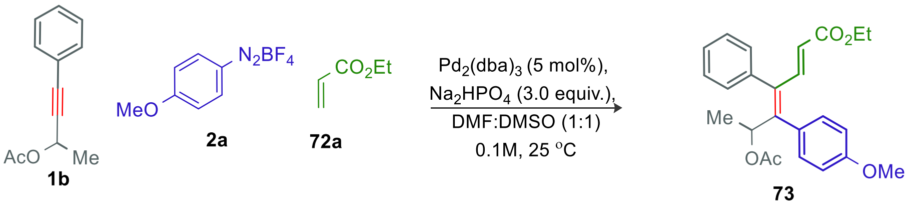

To a mixture of **1b** (0.2 mmol), *p*-methoxy benzenediazonium-tetrafluoroborate **2a** (0.6 mmol), ethyl acrylate **72a** (0.4 mmol), Pd catalyst (0.01 mmol), and base (0.3 mmol) was added solvent (2.0 mL). The resulting reaction mixture was stirred at 25 °C for 6 h. The reaction progress was periodically monitored by TLC. The respective solvent was next removed either by water workup or by evaporation under reduced pressure. The organic layer was extracted in ethyl acetate (3 × 10 mL) and dried over Na_2SO_4. The organic layer was evaporated and purified by column chromatography over neutral alumina to afford **73**. The compounds are sensitive to acidic silica gel and thus, final product purification was carried out on neutral alumina.

entry	deviation from the standard condition	yield (**73:73′**)
1	None	82% (93:7)
2	10 mol% $Pd(dba)_2$ as catalyst	74% (93:7)

3	K_3PO_4 as base	56% (93:7)
4	Diarylation condition	66% (93:7)
5	THF as solvent	76% (92:8)
6	1M solvent	80% (90:10)

2.12 General procedure for the regioselective aryl-alkenylation of alkynes (1) with aryl(hetero) diazonium salts (2) and olefins (72a–f) (GP-5):

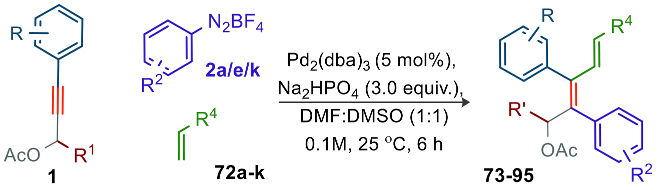

To a mixture of **1** (0.3 mmol), aryl(hetero) diazonium-tetrafluoroborate **2** (0.9 mmol), olefin **72** (0.6 mmol), $Pd_2(dba)_3$ (0.015 mmol), and Na_2HPO_4 (0.9 mmol) was added DMF:DMSO (1:1, 3.0 mL). The reaction mixture was stirred at 25 °C for 6 h. The reaction progress was periodically monitored by TLC. The reaction mixture was diluted with EtOAc (20 mL) and washed with water (3 × 10 mL) and brine. The organic layer was dried over Na_2SO_4. The solvent was evaporated and purified by column chromatography over neutral alumina eluting with EtOAc/hexane to afford **73–95**.

(2E,4Z)-Ethyl 6-acetoxy-5-(4-methoxyphenyl)-4-phenylhepta-2,4-dienoate (73/73′ = 93:7):

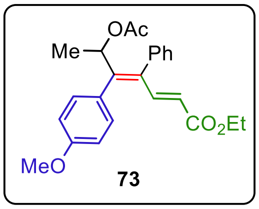

73 (97 mg, 82%) as pale yellow liquid; R_f = 0.23 (10% EtOAc/Hex); ^{1}H NMR (400 MHz, DMSO d_6) δ 7.54–7.41 (m, 3H), 7.31–7.16 (m, 5H), 7.07 (d, J = 8.0 Hz, 2H), 5.30 (q, J = 6.8 Hz, 1H), 5.10 (d, J = 15.6 Hz, 1H), 3.97 (q, J = 7.2 Hz, 2H), 3.83 (s, 3H), 1.85 (s, 3H), 1.16 (d, J = 6.8 Hz, 3H), 1.07 (t, J = 7.2 Hz, 3H); ^{13}C NMR (101 MHz, DMSO d_6) δ 169.8, 166.2, 159.5, 148.1, 145.0, 137.8, 137.1, 131.5, 129.6, 129.1, 128.2, 128.1, 121.9, 114.1, 71.0, 60.4, 55.5, 21.2, 19.3, 14.4; IR (Neat) ν_{max} 1732, 1709, 1508, 1275, 1232, 1168, 1020, 704 cm^{-1}; **HRMS (ESI)** for $C_{24}H_{26}NaO_5$+ $(M+Na)^+$: calcd. 417.1672, found 417.1682.

Representative peaks of the inseparable minor regio-isomer **73′**: ^{1}H NMR (400 MHz, DMSO d_6) δ 6.37 (d, J = 16.4 Hz, 0.08H), 3.78 (s, 0.24H); ^{13}C NMR (101 MHz, DMSO d_6) δ 167.0, 159.8, 151.5, 132.1, 128.8, 55.6, 19.8.

(2E,4Z)-Benzyl 6-acetoxy-5-(4-methoxyphenyl)-4-phenylhepta-2,4-dienoate (74/74′ = 93:7):

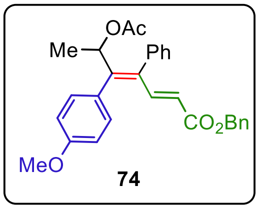

74 (104 mg, 76%) as pale yellow liquid; R_f = 0.20 (10% EtOAc/Hex); ^{1}H NMR (500 MHz, DMSO d$_6$) δ 7.49 (t, J = 7.0 Hz, 2H), 7.44–7.39 (m, 1H), 7.31–7.17 (m, 10H), 7.06 (d, J = 8.5 Hz, 2H), 5.29 (q, J = 6.5 Hz, 1H), 5.12 (d, J = 15.5 Hz, 1H), 5.01 (s, 2H), 3.35 (s, 3H), 1.84 (s, 3H), 1.16 (d, J = 6.5 Hz, 3H); ^{13}C NMR (101 MHz, DMSO d$_6$) δ 169.8, 166.0, 159.5, 148.5, 145.6, 137.8, 137.0, 136.4, 131.5, 129.6, 129.1, 128.8, 128.4, 128.3, 128.1, 121.4, 114.1, 71.0, 65.9, 55.6, 21.2, 19.3; IR (Neat) ν_{max} 1735, 1710, 1606, 1273, 1233, 1158, 1027 cm^{-1}; **HRMS (ESI)** for $C_{29}H_{28}NaO_5$+ (M+Na)$^+$: calcd. 479.1829, found 479.1839.

Representative peaks of the inseparable minor regio-isomer **74′**: ^{1}H NMR (500 MHz, DMSO d$_6$) δ 6.59 (d, J = 16.5 Hz, 0.08H), 3.77 (s, 0.24H); ^{13}C NMR (101 MHz, DMSO d$_6$) δ 132.1, 119.4, 19.9.

(2E,4Z)-Phenyl 6-acetoxy-5-(4-methoxyphenyl)-4-phenylhepta-2,4-dienoate (75/75′ = 93:7):

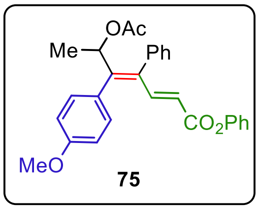

75 (62 mg, 47%) as pale yellow liquid; R_f = 0.18 (10% EtOAc/Hex); ^{1}H NMR (500 MHz, DMSO d$_6$) δ 7.36 (d, J = 7.0 Hz, 2H), 7.31–7.22 (m, 2H), 7.21–7.14 (m, 5H), 7.06–7.01 (m, 3H), 6.90–6.83 (m, 4H), 5.17–5.10 (m, 2H), 3.63 (s, 3H), 1.68 (s, 3H), 1.01 (d, J = 6.5 Hz, 3H); ^{13}C NMR (101 MHz, DMSO d$_6$) δ 169.8, 164.9, 159.6, 150.7, 149.4, 146.8, 137.7, 136.9, 131.5, 129.8, 129.6, 129.2, 128.4, 128.0, 126.3, 122.1, 120.8, 114.2, 71.0, 55.6, 21.2, 19.3; IR (Neat) ν_{max} 1727, 1607, 1507, 1491, 1368, 1272, 1231, 1129, 702 cm^{-1}; **HRMS (ESI)** for $C_{28}H_{26}NaO_5$ (M+Na)$^+$: calcd. 465.1678, found 465.1685.

Representative peaks of the inseparable minor regio-isomer **75′**: ^{1}H NMR (500 MHz, DMSO d$_6$) δ 6.41 (d, J = 16.0 Hz, 0.08H), 3.58 (s, 0.24H); ^{13}C NMR (126 MHz, DMSO d$_6$) δ 143.3, 132.2, 128.8, 19.9.

(3Z,5E)-6-Cyano-3-(4-methoxyphenyl)-4-phenylhexa-3,5-dien-2-yl acetate (76/76′ = 93:7):

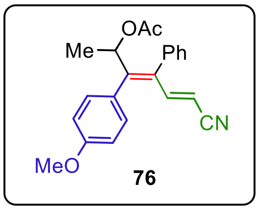

76 (53 mg, 51%) as pale yellow liquid; R_f = 0.18 (10% EtOAc/Hex); ^{1}H NMR (500 MHz, DMSO d$_6$) δ 7.53–7.42 (m, 4H), 7.29–7.25 (m, 3H), 7.19 (d, J = 9.0 Hz, 2H), 7.07 (d, J = 9.0 Hz, 2H), 6.94 (d, J = 16.5 Hz, 1H), 5.25 (q, J = 6.5 Hz, 1H), 4.83 (d, J = 16.5 Hz, 1H), 3.83 (s, 3H), 1.84 (s, 3H), 1.79 (s, 3H), 1.16 (d, J = 6.5 Hz, 3H); ^{13}C NMR (101 MHz, DMSO d$_6$) δ 169.8, 159.6, 150.5, 148.8, 137.5, 135.6, 131.5, 129.7, 129.3, 128.6, 127.6, 118.9, 114.3, 100.2, 70.8, 55.3, 21.2, 19.3; IR (Neat) ν_{max} 2213, 1604, 1507, 1368, 1230, 1176, 1028, 705 cm^{-1}; **HRMS (ESI)** for $C_{22}H_{21}NNaO_3$ (M+Na)$^+$: calcd. 370.1419, found 370.1415.

Representative peaks of the inseparable minor regio-isomer **76′**: ^{1}H NMR (500 MHz, DMSO d$_6$) δ 6.11 (d, J = 17.0 Hz, 0.09H), 3.78 (s, 0.24H); ^{13}C NMR (101 MHz, DMSO d$_6$) δ 157.8, 130.6, 21.0, 19.1.

(3Z,5E)-3-(4-Methoxyphenyl)-7-oxo-4-phenylocta-3,5-dien-2-yl acetate (77):

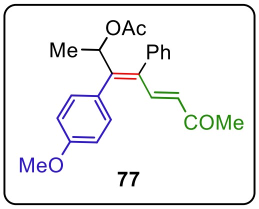

77 (mg, 54%) as pale yellow liquid; R_f = 0.17 (10% EtOAc/Hex); ^{1}H NMR (500 MHz, DMSO d_6) δ 7.49–7.36 (m, 3H), 7.21 (d, J = 7.0 Hz, 2H), 7.15 (d, J = 8.5 Hz, 2H), 7.07–6.99 (m, 3H), 5.36 (d, J = 16.0 Hz, 1H), 5.24 (q, J = 6.5 Hz, 1H), 3.78 (s, 3H), 1.89 (s, 3H), 1.79 (s, 3H), 1.12 (d, J = 6.5 Hz, 3H); ^{13}C NMR (126 MHz, DMSO d_6) δ 198.2, 170.1, 159.4, 148.7, 143.2, 138.1, 137.0, 131.5, 130.3, 129.5, 129.1, 128.3, 128.1, 114.0, 71.1, 55.5, 28.2, 21.1, 19.2; IR (Neat) ν_{max} 1734, 1662, 1586, 1367, 1232, 1175, 1028, 704 cm^{-1}; **HRMS (ESI)** for $C_{23}H_{24}NaO_4$ $(M+Na)^+$: calcd. 387.1572, found 387.1577.

(3Z,5E)-3-(4-Methoxyphenyl)-6-(3-nitrophenyl)-4-phenylhexa-3,5-dien-2-yl acetate (78/78′ = 93:7):

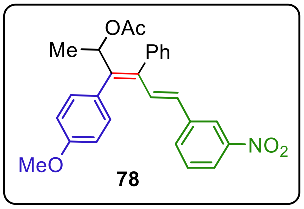

78 (109 mg, 82%) as pale yellow liquid; R_f = 0.28 (10% EtOAc/Hex); ^{1}H NMR (500 MHz, DMSO d_6) δ 7.99–7.95 (m, 1H), 7.82–7.78 (m, 1H), 7.54–7.41 (m, 5H), 7.31 (d, J = 8.0 Hz, 2H), 7.23 (d, J = 9.0 Hz, 2H), 7.06 (d, J = 9.0 Hz, 2H), 6.86 (d, J = 16.0 Hz, 1H), 5.95 (d, J = 16.0 Hz, 1H), 5.32 (q, J = 6.5 Hz, 1H), 3.82 (s, 3H), 1.87 (s, 3H), 1.14 (d, J = 6.5 Hz, 3H); ^{13}C NMR (126 MHz, DMSO d_6) δ 169.7, 159.2, 148.6, 142.6, 139.3, 138.9, 137.9, 132.6, 132.4, 131.8, 130.7, 130.5, 129.8, 129.0, 128.9, 128.0, 122.5, 121.0, 114.1, 71.1, 55.5, 21.3, 19.6; IR (Neat) ν_{max} 1728, 1605, 1526, 1346, 1234, 1029, 828, 699; **HRMS (ESI)** for $C_{27}H_{25}NNaO_5$ $(M+Na)^+$: calcd. 466.1630, found 466.1634.

Representative peaks of the inseparable minor regio-isomer **78′**: ^{1}H NMR (500 MHz, DMSO d_6) δ 6.51 (q, J = 7.0 Hz, 0.08H), 3.75 (s, 0.24H), 1.99 (s, 0.24H); ^{13}C NMR (126 MHz, DMSO d_6) δ 148.7, 145.8, 142.2, 129.4, 20.2.

(3Z,5E)-3-(4-Methoxyphenyl)-4-phenyl-6-(*p*-tolyl)hexa-3,5-dien-2-yl acetate (79/79′ = 91:9):

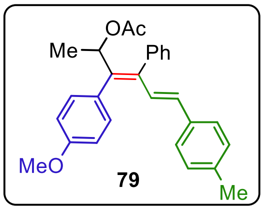

79 (89 mg, 72%) as pale yellow liquid; R_f = 0.35 (10% EtOAc/Hex); ^{1}H NMR (500 MHz, DMSO d_6) δ 7.48 (t, J = 7.0 Hz, 2H), 7.43–7.38 (m, 1H), 7.28 (d, J = 7.0 Hz, 2H), 7.21 (d, J = 8.5 Hz, 2H), 7.04 (d, J = 8.5 Hz, 2H), 6.97 (d, J = 8.0 Hz, 2H), 6.87 (d, J = 8.5 Hz, 2H), 6.71 (d, J = 16.0 Hz, 1H), 5.80 (d, J = 16.0 Hz, 1H), 5.32 (q, J = 6.5 Hz, 1H), 3.80 (s, 3H), 2.17 (s, 3H), 1.86 (s, 3H), 1.11 (d, J = 7.0 Hz, 3H); ^{13}C NMR (126 MHz, DMSO d_6) δ 169.6, 159.0, 140.1, 139.9, 138.4, 137.6, 134.5, 132.8, 131.9, 129.8, 129.4, 129.3, 129.1, 129.0, 128.9, 127.8, 126.4, 114.0, 71.2, 55.5, 21.3, 21.2, 19.6; ; IR (Neat) ν_{max} 1735, 1599, 1505, 1239, 1030, 700; **HRMS (ESI)** for $C_{28}H_{28}NaO_3$ $(M+Na)^+$: calcd. 435.1931, found 435.1932.

Representative peaks of the inseparable minor regio-isomer **79′**: ^{1}H NMR (500 MHz, DMSO d$_6$) δ 3.73 (s, 0.31H), 2.24 (s, 0.3H), 1.96 (s, 0.31H). ^{13}C NMR (126 MHz, DMSO d$_6$) δ 143.2, 137.3, 135.2, 131.0, 126.1, 113.9, 71.4, 20.3.

(3Z,5E)-3-(4-Methoxyphenyl)-4-phenyl-6-(*o*-tolyl)hexa-3,5-dien-2-yl acetate (80/80′ = 91:9):

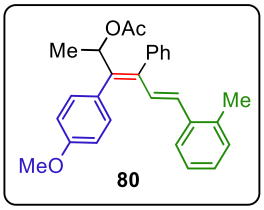

80 (72 mg, 58%) as pale yellow liquid; R$_f$ = 0.35 (10% EtOAc/Hex); ^{1}H NMR (500 MHz, DMSO d$_6$) δ 7.50 (t, J = 7.0 Hz, 2H), 7.47–7.35 (m, 3H), 7.32 (d, J = 7.0 Hz, 2H), 7.23 (d, J = 8.5 Hz, 2H), 7.07–7.02 (m, 4H), 5.62 (d, J = 16.0 Hz, 1H), 6.05 (d, J = 16.0 Hz, 1H), 5.35 (q, J = 6.5 Hz, 1H), 3.80 (s, 3H), 1.91 (s, 3H), 1.87 (s, 3H), 1.13 (d, J = 7.0 Hz, 3H); ^{13}C NMR (126 MHz, DMSO d$_6$) δ 169.7, 159.0, 143.3, 140.4, 140.1, 138.5, 136.1, 135.4, 131.9, 131.3, 130.82, 130.76, 129.8, 129.5, 129.0, 128.8, 128.0, 127.9, 126.7, 125.0, 114.0, 71.2, 55.5, 21.4, 19.7, 19.4; IR (Neat) ν_{max} 1732, 1606, 1508, 1368, 1241, 1177, 1033, 754; **HRMS (ESI)** for $C_{28}H_{28}NaO_3$ (M+Na)$^+$: calcd. 435.1931, found 435.1935.

Representative peaks of the inseparable minor regio-isomer **80′**: ^{1}H NMR (500 MHz, DMSO d$_6$) δ 3.73 (s, 0.31H), 1.99 (s, 0.3H), 1.98 (s, 0.3H). ^{13}C NMR (126 MHz, DMSO d$_6$) δ 169.7, 135.3, 129.3, 126.2, 71.4, 20.4, 19.8.

(3Z,5E)-3-(4-Methoxyphenyl)-4-phenyl-6-(*p*-tolyl)hexa-3,5-dien-2-yl acetate (81/81′ = 91:9):

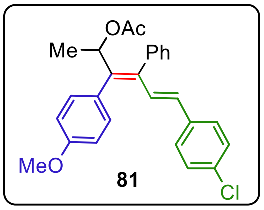

81 (101 mg, 78%) as pale yellow liquid; R$_f$ = 0.35 (10% EtOAc/Hex); ^{1}H NMR (500 MHz, DMSO d$_6$) δ 7.49 (t, J = 7.0 Hz, 2H), 7.45–7.40 (m, 1H), 7.29 (d, J = 7.0 Hz, 2H), 7.24–7.19 (m, 4H), 7.04 (dd, J = 6.5, 2.0 Hz, 2H), 6.99 (d, J = 8.5 Hz, 2H), 6.76 (d, J = 16.00 Hz, 1H), 5.81 (d, J = 16.0 Hz, 1H), 5.32 (q, J = 6.5 Hz, 1H), 3.81 (s, 3H), 1.86 (s, 3H), 1.12 (d, J = 7.0 Hz, 3H); ^{13}C NMR (126 MHz, DMSO d$_6$) δ 169.6, 159.1, 141.3, 139.6, 138.2, 136.00, 132.5, 131.8, 131.4, 130.8, 129.8, 129.4, 129.2, 129.1, 128.9, 128.1, 114.0, 71.1, 55.5, 21.3, 19.6; IR (Neat) ν_{max} 1733, 1606, 1507, 1284, 1239, 1034, 836, 713; **HRMS (ESI)** for $C_{27}H_{25}NaClO_3$ (M+Na)$^+$: calcd. 455.1384, found 455.1382.

Representative peaks of the inseparable minor regio-isomer **81′**: ^{1}H NMR (500 MHz, DMSO d$_6$) δ 3.73 (s, 0.3H), 1.97 (s, 0.31H); ^{13}C NMR (126 MHz, DMSO d$_6$) δ 143.2, 132.5, 126.1, 113.9, 21.3, 20.3.

(2E,4Z)-Ethyl 6-acetoxy-5-(3-cyanophenyl)-4-phenylhepta-2,4-dienoate (82/82′ = 89:11):

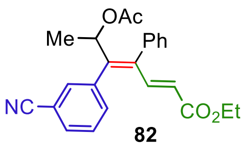

82

82 (73 mg, 63%) as pale yellow liquid; R_f = 0.13 (10% EtOAc/Hex); ^{1}H NMR (500 MHz, DMSO d$_6$) δ 7.98 (d, J = 8.0 Hz, 1H), 7.88–7.12 (m, 2H), 7.67–7.60 (m, 1H), 7.56–7.44 (m, 3H), 7.31 (d, J = 8.0 Hz, 2H), 7.01 (d, J = 15.5 Hz, 2H), 5.34 (q, J = 6.5 Hz, 1H), 5.15 (d, J = 15.5 Hz, 1H), 3.98 (q, J = 7.0 Hz, 2H), 1.88 (s, 3H), 1.17 (d, J = 6.5 Hz, 3H), 1.07 (t, J = 7.0 Hz, 3H); ^{13}C NMR (126 MHz, DMSO d$_6$) δ 169.7, 165.9, 145.6, 143.8, 138.6, 137.5, 136.3, 135.3, 133.7, 132.5, 130.1, 129.5, 129.2, 128.5, 123.2, 119.0, 112.0, 70.3, 60.6, 21.2, 19.2, 14.4; IR (Neat) ν_{max} 1734, 1712, 1620, 1365, 1279, 1168, 1034, 701 cm^{-1}; **HRMS (ESI)** for $C_{24}H_{23}NNaO_4$ (M+Na)$^+$: calcd. 412.1525, found 412.1524.

Representative peaks of the inseparable minor regio-isomer **82′**: ^{1}H NMR (500 MHz, DMSO d$_6$) δ 6.37 (d, J = 16.0 Hz, 0.12H), 1.98 (s, 0.36H); ^{13}C NMR (126 MHz, DMSO d$_6$) δ 165.6, 142.2, 140.2, 139.4, 134.8, 133.3, 129.0, 129.0, 121.7, 19.9, 14.5.

(2E,4Z)-Ethyl 6-acetoxy-5-(9-ethyl-9H-carbazol-3-yl)-4-phenylhepta-2,4-dienoate (83:83′ = 92:8):

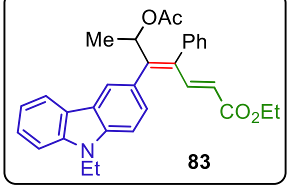

83

83 (64 mg, 44%) as pale yellow liquid; R_f = 0.18 (10% EtOAc/Hex); ^{1}H NMR (400 MHz, DMSO d$_6$) δ 8.25 (d, J = 7.6 Hz, 1H), 8.06 (br s, 1H), 7.74 (d, J = 8.4 Hz, 1H), 7.67 (d, J = 8.4 Hz, 1H), 7.58–7.43 (m, 4H), 7.38–7.31 (m, 3H), 7.29–7.21 (m, 2H), 5.42 (q, J = 6.8 Hz, 1H), 5.12 (d, J = 15.6 Hz, 1H), 4.51 (q, J = 7.2 Hz, 2H), 3.91 (q, J = 6.8 Hz, 2H), 1.87 (s, 3H), 1.39 (t, J = 7.2 Hz, 3H), 1.24 (d, J = 6.8 Hz, 3H), 1.01 (t, J = 7.2 Hz, 3H); ^{13}C NMR (126 MHz, DMSO d$_6$) δ 169.9, 166.2, 149.2, 145.3, 140.4, 139.5, 137.9, 137.3, 129.6, 129.2, 128.3, 128.1, 126.6, 126.4, 122.43, 122.37, 121.9, 121.7, 121.1, 119.5, 109.8, 109.3, 71.2, 60.3, 37.6, 21.3, 19.5, 14.4, 14.3; IR (Neat) ν_{max} 1732, 1707, 1475, 1230, 1171, 700 cm^{-1}; **HRMS (ESI)** for $C_{31}H_{31}NNaO_4$ (M+Na)$^+$: calcd. 504.2151, found 504.2156.

Representative peaks of the inseparable minor regio-isomer **83′**: ^{1}H NMR (500 MHz, DMSO d$_6$) δ 6.40 (d, J = 16.4 Hz, 0.09H), 2.01 (s, 0.27H).

(2E,4Z)-Ethyl 6-acetoxy-5-(4-methoxyphenyl)-4-(*p*-tolyl)hepta-2,4-dienoate (84:84′ = 90:10):

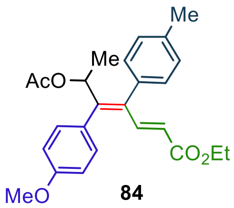

84

84 (97 mg, 79%) as pale yellow liquid; R_f = 0.26 (10% EtOAc/Hex); ^{1}H NMR (500 MHz, DMSO d$_6$) δ 7.30 (d, J = 7.5 Hz, 2H), 7.22–7.12 (m, 5H), 7.05 (d, J = 9.0 Hz, 2H), 5.32 (q, J = 6.5 Hz, 1H), 5.11 (d, J = 15.5 Hz, 1H), 3.96 (q, J = 7.0 Hz, 2H), 3.82 (s, 3H), 2.37 (s, 3H), 1.85 (s, 3H), 1.15 (d, J = 7.0 Hz, 3H), 1.06 (t, J = 7.0 Hz, 3H); ^{13}C NMR (126 MHz, DMSO d$_6$) δ 169.8, 166.2, 159.4, 148.0, 145.1, 137.8, 137.5, 134.1, 131.5, 129.7, 129.4, 128.2, 121.9, 114.1, 71.0, 60.4, 55.5, 21.3, 21.2, 19.3, 14.4; IR

(Neat) ν_{max} 1734, 1708, 1607, 1510, 1233, 1171, 1030 cm^{-1}; **HRMS (ESI)** for $C_{25}H_{28}NaO_5$ $(M+Na)^+$: calcd. 431.1832, found 431.1836.

Representative peaks of the inseparable minor regio-isomer **84′**: ^{1}H NMR (500 MHz, DMSO d_6) δ 6.35 (d, J = 16.5 Hz, 0.11H), 3.77 (s, 0.34H), 2.32 (s, 0.34H); ^{13}C NMR (126 MHz, DMSO d_6) δ 167.0, 159.8, 142.0, 138.6, 133.3, 132.2, 119.5, 19.8, 14.6.

(2E,4Z)-Ethyl 6-acetoxy-4-(4-fluorophenyl)-5-(4-methoxyphenyl)hepta-2,4-dienoate (85:85′ = 93:7):

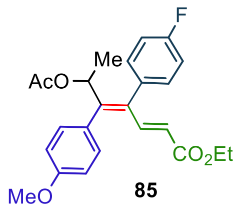

85

85 (78 mg, 63%) as pale yellow liquid; R_f = 0.26 (10% EtOAc/Hex); ^{1}H NMR (400 MHz, DMSO d_6) δ 7.35–7.29 (m, 4H), 7.21–7.15 (m, 3H), 7.05 (d, J = 8.5 Hz, 2H), 5.26 (q, J = 7.0 Hz, 1H), 5.08 (d, J = 15.5 Hz, 1H), 3.97 (q, J = 7.0 Hz, 2H), 3.82 (s, 3H), 1.85 (s, 3H), 1.15 (d, J = 7.0 Hz, 3H), 1.06 (t, J = 7.0 Hz, 3H); ^{13}C NMR (126 MHz, DMSO d_6) δ 169.8, 166.2, 162.1 (d, J = 245 Hz, 1C), 159.5, 148.6, 144.9, 136.8, 133.3, 131.7 (d, J = 7.6 Hz, 2C), 131.5, 121.9, 116.1 (d, J = 21.4 Hz, 2C), 114.1, 70.9, 60.4, 55.6, 55.5, 21.2, 19.2, 14.6, 14.4; ^{19}F NMR (471 MHz) δ –114.3; IR (Neat) ν_{max} 1734, 1709, 1604, 1508, 1367, 1233, 1029 cm^{-1}; **HRMS (ESI)** for $C_{24}H_{25}FNaO_5$ $(M+Na)^+$: calcd. 435.1584, found 435.1589.

Representative peaks of the inseparable minor regio-isomer **85′**: ^{1}H NMR (500 MHz, DMSO d_6) δ 6.35 (d, J = 16.0 Hz, 0.08H), 3.77 (s, 0.24H), 1.96 (s, 0.34H); ^{13}C NMR (126 MHz, DMSO d_6) δ 166.9, 55.6, 14.6; ^{19}F NMR (471 MHz) δ –113.8.

(2E,4Z)-Ethyl 6-acetoxy-4-(4-bromophenyl)-5-(4-methoxyphenyl)hepta-2,4-dienoate (86:86′ =92:8):

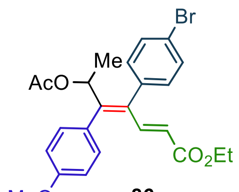

86

86 (103 mg, 73%) as pale yellow liquid; R_f = 0.26 (10% EtOAc/Hex); ^{1}H NMR (400 MHz, DMSO d_6) δ 7.71 (d, J = 8.4 Hz, 2H), 7.27–7.14 (m, 5H), 7.07 (d, J = 9.2 Hz, 2H), 5.25 (q, J = 6.8 Hz, 1H), 5.09 (d, J = 15.6 Hz, 1H), 3.98 (q, J = 7.2 Hz, 2H), 3.84 (s, 3H), 1.87 (s, 3H), 1.16 (d, J = 6.4 Hz, 3H), 1.08 (t, J = 7.2 Hz, 3H); ^{13}C NMR (126 MHz, DMSO d_6) δ 169.9, 166.1, 159.5, 148.5, 144.6, 136.6, 136.4, 132.1, 131.9, 131.5, 127.9, 121.9, 121.7, 114.2, 70.9, 60.5, 55.6, 21.2, 19.2, 14.4; IR (Neat) ν_{max} 1731, 1710, 1610, 1509, 1244, 1167, 1028, 810 cm^{-1}; **HRMS (ESI)** for $C_{24}H_{25}BrNaO_5$ (M+Na)+: calcd. 495.0783, found 495.0789.

Representative peaks of the inseparable minor regio-isomer **86′**: ^{1}H NMR (400 MHz, DMSO d_6) δ 6.35 (d, J = 16.4 Hz, 0.09H), 3.78 (s, 0.29H), 1.97 (s, 0.34H); ^{13}C NMR (101 MHz, DMSO d_6) δ 19.8, 14.6.

(2E,4Z)-Ethyl 6-acetoxy-5-(4-methoxyphenyl)-4-(naphthalen-2-yl)hepta-2,4-dienoate (87:87′ = 95:5):

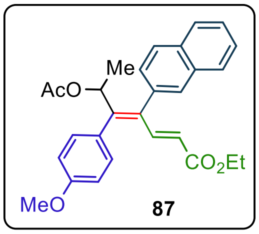

87 (88 mg, 70%) as pale yellow liquid; R_f = 0.22 (10% EtOAc/Hex); ^{1}H NMR (400 MHz, DMSO d_6) δ 8.06–7.94 (m, 3H), 7.84 (s, 1H), 7.59–7.54 (m, 2H), 7.40 (br d, J = 8.0 Hz, 1H), 7.32–7.22 (m, 3H), 7.09 (d, J = 9.0 Hz, 2H), 5.34 (q, J = 6.5 Hz, 1H), 5.11 (d, J = 15.5 Hz, 1H), 3.93 (q, J = 7.0 Hz, 2H), 3.83 (s, 3H), 1.84 (s, 3H), 1.17 (d, J = 6.5 Hz, 3H), 1.01 (t, J = 7.0 Hz, 3H); ^{13}C NMR (126 MHz, DMSO d_6) δ 169.8, 166.2, 159.5, 148.5, 144.9, 137.8, 134.7, 133.4, 132.7, 131.5, 128.7, 128.4, 128.20, 128.15, 127.7, 126.94, 126.87, 122.2, 114.2, 71.1, 60.4, 55.6, 21.2, 19.4, 14.3; IR (Neat) ν_{max} 1733, 1707, 1607, 1507, 1366, 1234, 1171, 1029, 746 cm^{-1}; **HRMS (ESI)** for $C_{28}H_{29}O_5$ $(M+H)^+$: calcd. 445.2010, found 445.1997.

Representative peaks of the inseparable minor regio-isomer **87′**: ^{1}H NMR (500 MHz, DMSO d_6) δ 6.41 (d, J = 16.5 Hz, 0.06H), 3.78 (s, 0.18H), 1.95 (s, 0.19H).

(2E,4Z)-Ethyl 6-acetoxy-5-(4-methoxyphenyl)-4-(thiophen-2-yl)hepta-2,4-dienoate (88):

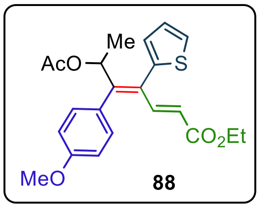

88 (85 mg, 71%) as pale yellow liquid; R_f = 0.16 (10% EtOAc/Hex); ^{1}H NMR (500 MHz, DMSO d_6) δ 7.73 (dd, J = 5.5, 1.5 Hz, 1H), 7.22–7.15 (m, 4H), 7.09–7.04 (m, 3H), 5.50 (q, J = 7.0 Hz, 1H), 5.35 (d, J = 15.5 Hz, 1H), 4.00 (q, J = 7.0 Hz, 2H), 3.83 (s, 3H), 1.87 (s, 3H), 1.22 (d, J = 7.0 Hz, 3H), 1.09 (t, J = 7.5 Hz, 3H); ^{13}C NMR (126 MHz, DMSO d_6) δ 169.8, 166.2, 159.6, 151.6, 144.8, 136.4, 131.3, 130.5, 128.9, 127.9, 122.1, 114.2, 70.9, 60.5, 55.6, 21.2, 19.5, 14.4; IR (Neat) ν_{max} 1733, 1710, 1617, 1506, 1367, 1231, 1163, 1020, 711 cm^{-1}; **HRMS (ESI)** for $C_{22}H_{24}NaO_5S$ $(M+Na)^+$: calcd. 423.1237, found 423.1240.

(2E,4Z)-Ethyl 6-Acetoxy-6-(benzo[d][1,3]dioxol-5-yl)-5-(4-methoxyphenyl)-4-phenylhexa-2,4-dienoate (89):

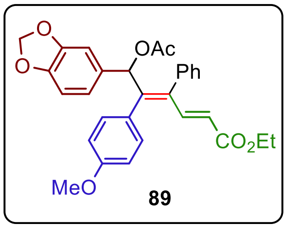

89 (77 mg, 51%) as pale yellow liquid; R_f = 0.13 (10% EtOAc/Hex); ^{1}H NMR (500 MHz, DMSO d_6) δ 7.53 (t, J = 8.0 Hz, 2H), 7.49–7.42 (m, 1H), 7.36 (d, J = 6.8 Hz, 2H), 7.26 (d, J = 16.0 Hz, 1H), 7.03–6.94 (m, 4H), 6.80 (d, J = 8.0 Hz, 1H), 6.53–6.48 (m, 1H), 6.45 (d, J = 1.6 Hz, 1H), 6.33 (s, 1H), 5.98 (dd, J = 5.2, 0.8 Hz, 2H), 5.16 (d, J = 15.6 Hz, 1H), 3.99 (q, J = 7.2 Hz, 2H), 3.80 (s, 3H), 2.01 (s, 3H), 1.08 (t, J = 7.2 Hz, 3H); ^{13}C NMR (101 MHz, DMSO d_6) δ 169.5, 166.2, 159.5, 147.6, 147.1, 145.9, 145.0, 139.4, 137.2, 132.0, 131.9, 129.8, 129.2, 128.5, 128.0, 122.8, 120.1, 113.9, 108.4, 106.9, 101.6, 75.0, 60.5, 55.5, 21.2, 14.4; IR (Neat) ν_{max} 1740, 1708, 1606, 1223, 1172, 1027, 701 cm^{-1}; **HRMS (ESI)** for $C_{30}H_{28}NaO_7$ $(M+Na)^+$: calcd. 523.1733, found 523.1738.

(2E,4Z)-Ethyl 6-acetoxy-5-(4-methoxyphenyl)-6-(naphthalen-2-yl)-4-phenylhexa-2,4-dienoate (90):

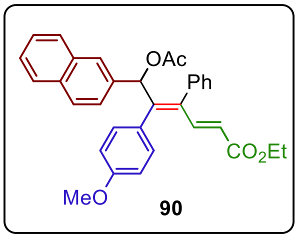

90 (62 mg, 41%) as pale yellow liquid; $R_f = 0.13$ (10% EtOAc/Hex); ^{1}H NMR (500 MHz, DMSO d_6) δ 7.87 (dd, J = 6.4, 6.0 Hz, 2H), 7.83 (d, J = 8.4 Hz, 1H), 7.55 (d, J = 7.6 Hz, 3H), 7.52–7.43 (m, 5H), 7.32 (d, J = 15.6 Hz, 1H), 7.21 (dd, J = 8.8, 1.6 Hz, 1H), 7.00 (br d, J = 7.6 Hz, 2H), 6.90 (d, J = 8.8 Hz, 2H), 6.62 (s, 1H), 5.22 (d, J = 15.6 Hz, 1H), 4.00 (q, J = 7.2 Hz, 2H), 3.74 (s, 3H), 2.11 (s, 3H), 1.09 (t, J = 7.2 Hz, 3H); ^{13}C NMR (101 MHz, DMSO d_6) δ 169.7, 166.2, 159.4, 145.6, 145.1, 139.9, 137.2, 135.5, 133.0, 132.7, 131.9, 129.9, 129.3, 128.5, 128.4, 128.3, 128.1, 127.9, 126.8, 126.7, 125.2, 124.5, 123.0, 113.8, 75.4, 60.5, 55.5, 21.3, 14.4; IR (Neat) ν_{max} 1740, 1707, 1606, 1507, 1366, 1172, 1024, 701 cm^{-1}; **HRMS (ESI)** for $C_{33}H_{30}NaO_5$ $(M+Na)^+$: calcd. 529.1991, found 529.1995.

(2E,4Z)-Ethyl 6-acetoxy-5-(4-methoxyphenyl)-4-phenylnona-2,4-dienoate (91):

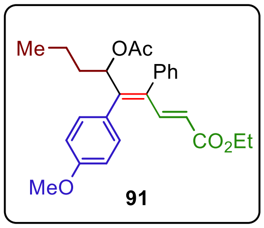

91 (85 mg, 67%) as pale yellow liquid; $R_f = 0.26$ (10% EtOAc/Hex); ^{1}H NMR (500 MHz, DMSO d_6) δ 7.48 (t, J = 7.5 Hz, 2H), 7.41 (t, J = 7.5 Hz, 1H), 7.29–7.14 (m, 5H), 7.05 (d, J = 8.5 Hz, 3H), 5.23 (q, J = 4.5 Hz, 1H), 5.09 (d, J = 15.5 Hz, 1H), 3.96 (q, J = 7.0 Hz, 2H), 3.82 (s, 3H), 1.86 (s, 3H), 1.59–1.38 (m, 2H), 1.20–0.07 (m, 5H), 0.60 (t, J = 7.5 Hz, 3H); ^{13}C NMR (126 MHz, DMSO d_6) δ 170.0, 166.2, 159.5, 147.7, 145.0, 138.3, 137.1, 131.4, 129.7, 129.0, 128.4, 128.2, 121.8, 114.1, 74.1, 60.4, 55.6, 35.1, 21.1, 18.6, 14.4, 13.5; IR (Neat) ν_{max} 1745, 1714, 1605, 1515, 1369, 1174, 1043, 700 cm^{-1}; **HRMS (ESI)** for $C_{26}H_{30}NaO_5$: $(M+Na)^+$: calcd. 445.1985, found 445.1992.

(2E,4Z)-Ethyl 6-acetoxy-6-cyclopropyl-5-(4-methoxyphenyl)-4-phenylhexa-2,4-dienoate (92):

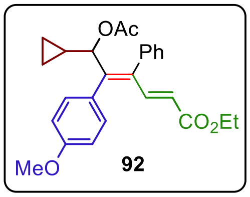

92 (63 mg, 50%) as pale yellow liquid; $R_f = 0.26$ (10% EtOAc/Hex); ^{1}H NMR (500 MHz, DMSO d_6) δ 7.41–7.30 (m, 3H), 7.21–7.13 (m, 5H), 6.99 (d, J = 8.5 Hz, 2H), 5.01 (d, J = 15.5 Hz, 1H), 4.56 (d, J = 9.0 Hz, 1H), 3.89 (q, J = 7.0 Hz, 2H), 3.75 (s, 3H), 1.83 (s, 3H), 0.99 (t, J = 7.0 Hz, 3H), 0.88–0.78 (m, 1H), 0.39–0.27 (m, 2H), 0.05–(-0.19) (m, 2H); ^{13}C NMR (126 MHz, DMSO d_6) δ 169.7, 166.2, 159.4, 147.1, 145.2, 138.1, 137.0, 131.5, 129.9, 128.9, 128.3, 122.3, 114.1, 78.2, 60.4, 55.6, 21.3, 14.40, 14.35, 4.8, 3.3; IR (Neat) ν_{max} 2939, 2183, 1598, 1508, 1329, 1292, 1247, 1171, 1027, 813 cm^{-1}; **HRMS (ESI)** for $C_{26}H_{28}NaO_5$ $(M+Na)^+$: calcd. 443.1834, found 443.1839.

(2E,4Z)-Ethyl 6-acetoxy-5-(4-methoxyphenyl)-6-methyl-4-phenylhepta-2,4-dienoate (93):

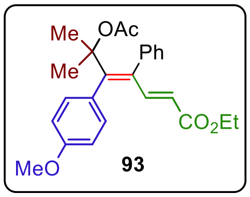

93 (52 mg, 42%) as pale yellow liquid; R_f = 0.26 (10% EtOAc/Hex); ^{1}H NMR (500 MHz, DMSO d_6) δ 7.47 (t, J = 7.5 Hz, 2H), 7.41–7.36 (m, 1H), 7.27 (dt, J = 6.5, 2.0 Hz, 2H), 7.17–7.13 (m, 2H), 7.10–7.03 (m, 3H), 4.75 (d, J = 15.5 Hz, 1H), 3.91 (q, J = 7.0 Hz, 2H), 3.82 (s, 3H), 1.54 (s, 3H), 1.32 (s, 6H), 1.03 (t, J = 7.5 Hz, 3H); ^{13}C NMR (126 MHz, DMSO d_6) δ 169.6, 166.3, 159.0, 152.2, 147.9, 138.4, 135.8, 131.6, 130.8, 129.6, 128.7, 127.6, 120.3, 114.1, 81.1, 60.2, 55.5, 29.2, 22.2, 14.4; IR (Neat) ν_{max} 1722, 1700, 1506, 1240, 1176, 1138, 1029, 733 cm^{-1}; **HRMS (ESI)** for $C_{25}H_{28}NaO_5$ $(M+Na)^+$: calcd. 431.1834, found 431.1839.

(2E,4Z)-Ethyl 5-(1-acetoxycyclopentyl)-5-(4-methoxyphenyl)-4-phenylpenta-2,4-dienoate (94):

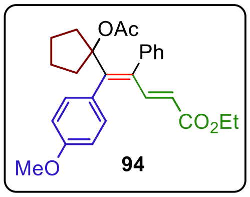

94 (94 mg, 72%) as pale yellow liquid; R_f = 0.26 (10% EtOAc/Hex); ^{1}H NMR (500 MHz, DMSO d_6) δ 7.46 (t, J = 7.5 Hz, 2H), 7.40–7.35 (m, 1H), 7.32 (d, J = 9.0 Hz, 2H), 7.15–7.02 (m, 5H), 4.78 (d, J = 15.5 Hz, 1H), 3.92 (q, J = 7.0 Hz, 2H), 3.82 (s, 3H), 2.10–1.92 (m, 2H), 1.69–1.61 (m, 2H), 1.60 (s, 3H), 1.43–1.34 (m, 4H), 1.04 (t, J = 7.0 Hz, 3H); ^{13}C NMR (101 MHz, DMSO d_6) δ 169.7, 166.3, 159.1, 151.7, 147.7, 138.6, 135.9, 131.7, 130.9, 129.7, 128.5, 127.6, 120.0, 114.1, 90.9, 60.2, 55.6, 23.1, 22.2, 14.4; IR (Neat) ν_{max} 1707, 1606, 1507, 1282, 1242, 1169, 1030, 702 cm^{-1}; **HRMS (ESI)** for $C_{27}H_{30}NaO_5$ $(M+Na)^+$: calcd. 457.1991, found 457.1998.

(2E,4Z)-Ethyl 5-(1-acetoxycyclobutyl)-5-(4-methoxyphenyl)-4-phenylpenta-2,4-dienoate (95):

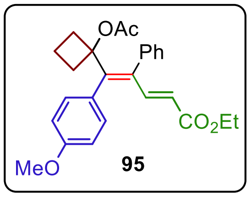

95 (109 mg, 86%) as pale yellow liquid; R_f = 0.26 (10% EtOAc/Hex); ^{1}H NMR (400 MHz, DMSO d_6) δ 7.47–7.34 (m, 5H), 7.18–7.05 (m, 5H), 4.96 (d, J = 15.2 Hz, 1H), 3.94 (q, J = 6.8 Hz, 2H), 3.84 (s, 3H), 2.38–2.27 (m, 2H), 2.08–1.97 (m, 2H), 1.76–1.62 (m, 4H), 1.39–1.24 (m, 1H), 1.04 (t, J = 6.8 Hz, 3H); ^{13}C NMR (101 MHz, DMSO d_6) δ 169.5, 166.3, 159.4, 152.2, 147.2, 138.6, 135.8, 131.3, 129.6, 128.5, 127.7, 120.4, 114.1, 83.6, 60.2, 55.5, 35.4, 21.5, 15.3, 14.4; IR (Neat) ν_{max} 1731, 1708, 1607, 1507, 1258, 1168, 1032, 704 cm^{-1}; **HRMS (ESI)** for $C_{26}H_{28}NaO_5$ $(M+Na)^+$: calcd. 443.1834, found 443.1838.

2.13 General Procedure for synthesis of indene derivatives 101–104:

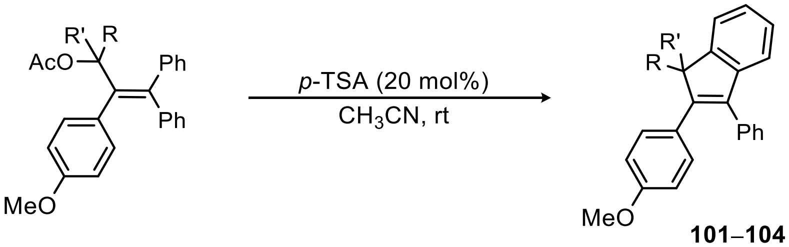

To an independent solution of acetate **58**, **59**, **38**, & **4** (0.2 mmol) in CH_3CN (2.0 mL) was added *p*-TSA (0.04 mmol). The resulting mixture was stirred at rt. The reaction progress was monitored by TLC. Upon completion, the crude mixture was concentrated under reduced pressure. The crude residue was purified using column chromatography on silica gel to afford **101–104**.

2'-(4-methoxyphenyl)-3'-phenylspiro[cyclohexane-1,1'-indene] (101):

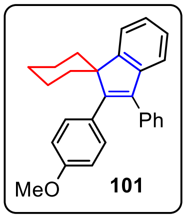

101 (48 mg, 65%) as colorless gummy liquid; R_f = 0.54 (10% EtOAc/Hex); ^{1}H NMR (500 MHz, $CDCl_3$) δ 7.90 (d, J = 7.5 Hz, 1H), 7.36–7.14 (m, 8H), 7.01 (d, J = 9.0 Hz, 2H), 6.81 (d, J = 8.5 Hz, 2H), 3.78 (s, 3H), 2.08–1.96 (m, 2H), 1.85 (td, J = 8.5, 1.0 Hz, 3H), 1.73 (dt, J = 13.5, 3.5 Hz, 2H), 1.46 (d, J = 12.5 Hz, 2H), 1.30–1.17 (m, 1H); ^{13}C NMR (126 MHz, $CDCl_3$) δ 158.4, 154.1, 151.8, 143.8, 138.5, 135.3, 131.7, 129.5, 128.9, 127.9, 126.7, 126.3, 124.8, 124.3, 120.6, 113.3, 55.1, 54.5, 31.5, 25.1, 22.3; IR (Neat) ν_{max} 1610, 1536, 1449, 1239, 1064, 708 cm^{-1}; **HRMS (ESI)** for $C_{27}H_{27}O$ $(M+H)^+$: calcd. 367.2056, found 367.2060.

2'-(4-Methoxyphenyl)-3'-phenylspiro[cyclobutane-1,1'-indene] (102):

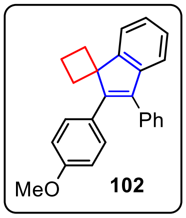

102 (56 mg, 83%) as colorless gummy liquid; R_f = 0.54 (10% EtOAc/Hex); ^{1}H NMR (500 MHz, $CDCl_3$) δ 7.82 (d, J = 7.5 Hz, 1H), 7.38–7.21 (m, 10H), 6.90 (d, J = 9.0 Hz, 2H), 3.84 (s, 3H), 2.69–2.51 (m, 4H), 2.28–2.17 (m, 1H), 1.92–1.81 (m, 1H); ^{13}C NMR (126 MHz, $CDCl_3$) δ 158.6, 152.3, 149.2, 142.8, 137.9, 135.3, 130.9, 129.5, 129.2, 128.0, 126.7, 126.4, , 125.6, 121.3, 120.0, 113.6, 56.5, 55.1, 29.0, 16.7; IR (Neat) ν_{max} 1598, 1555, 1417, 1219, 1068, 709cm^{-1}; **HRMS (ESI)** for $C_{25}H_{23}O$ $(M+H)^+$: calcd. 339.1743, found 339.1747.

2-(4-Methoxyphenyl)-1-methyl-3-phenyl-1H-indene (103):

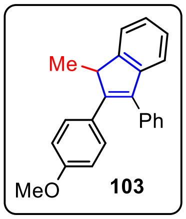

103 (54 mg, 89%) as pale yellow liquid; R$_f$ = 0.58 (10% EtOAc/Hex); ^{1}H NMR (500 MHz, DMSO d$_6$) δ 7.58–7.54 (m, 1H), 7.45–7.39 (m, 2H), 7.38–7.33 (m, 1H), 7.31–7.23 (m, 4H), 7.15–7.10 (m, 3H), 6.83 (dt, J = 9.0, 2.5 Hz, 2H), 4.13 (q, J = 7.5 Hz, 1H), 3.72 (s, 3H), 1.20 (d, J = 7.5 Hz, 3H); ^{13}C NMR (126 MHz, DMSO d$_6$) δ 158.7, 148.5, 147.9, 144.9, 136.6, 135.9, 130.8, 129.6, 129.3, 127.78, 128.75, 127.1, 125.4, 123.4, 119.9, 114.2, 55.5, 45.6, 17.3; IR (Neat) ν_{max} 1637, 1589, 1412, 1214, 1068, 699 cm^{-1}; **HRMS (ESI)** for $C_{23}H_{21}O$ (M+H)$^+$: calcd. 313.1587, found 313.1585.

2-(4-Methoxyphenyl)-1,3-diphenyl-1H-indene (104):

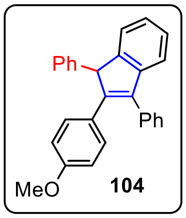

104 (56 mg, 79%) as pale yellow liquid; R$_f$ = 0.54 (10% EtOAc/Hex); ^{1}H NMR (500 MHz, DMSO d$_6$) δ 7.48 (t, J = 7.5 Hz, 2H), 7.40 (t, J = 8.0 Hz, 3H), 7.26–7.16 (m, 6H), 7.15–7.08 (m, 5H), 6.67 (d, J = 8.5 Hz, 2H), 5.38 (s, 1H), 3.62 (s, 3H); ^{13}C NMR (126 MHz, DMSO d$_6$) δ 158.6, 148.4, 145.9, 145.2, 140.7, 139.1, 135.8, 130.8, 129.7, 129.4, 129.1, 128.4, 128.0, 127.8, 127.3, 126.9, 125.8, 124.2, 120.1, 113.9, 57.2, 55.3; IR (Neat) ν_{max} 2939, 1509, 1455, 1246, 1176, 1026, 696 cm^{-1}; **HRMS (ESI)** for $C_{28}H_{23}O$ (M+H)$^+$: calcd. 375.1749, found 375.1756.

2.14 Synthesis of 2-(4-methoxyphenyl)-3-phenyl-1H-inden-1-one (105):[4]

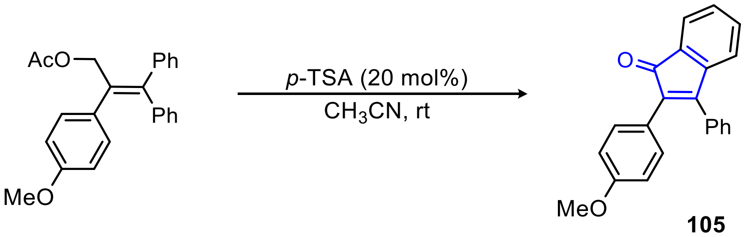

105

To a solution of **23** (0.2 mmol, 72 mg) in CH_3CN (2.0 mL) was added *p*-TSA (0.04 mmol, 7.6 mg). The resulting mixture was stirred at rt. The reaction progress was monitored by TLC. Upon completion, the crude mixture was concentrated under reduced pressure. The crude residue was purified using column chromatography on neutral alumina to afford **105** in 58% yield as red gummy liquid; R$_f$ = 0.4 (10% EtOAc/Hex); ^{1}H NMR (400 MHz, $CDCl_3$) δ 7.59 (br d, J = 7.2 Hz, 1H), 7.48–7.35 (m, 6H), 7.32–7.24 (m, 3H), 7.14 (br d J = 7.2 Hz, 1H), 6.83 (d, J = 9.2 Hz, 2H), 3.82 (s, 3H); ^{13}C NMR (101 MHz, $CDCl_3$) δ 197.0, 159.2, 153.8, 145.5, 133.4, 133.0, 131.9, 131.2, 130.7, 129.1, 128.8, 128.6, 128.5, 123.1, 122.9, 120.9, 113.6, 55.2 cm^{-1}; IR (Neat) ν_{max} 1706, 1604, 1455, 1291, 1247, 1176, 700 cm^{-1}; **HRMS (ESI)** for $C_{22}H_{17}O_2$ (M+H)$^+$: calcd. 313.1223, found 313.1225.

2.15 General Procedure for synthesis of allylic alcohol derivatives 106–107:[3]

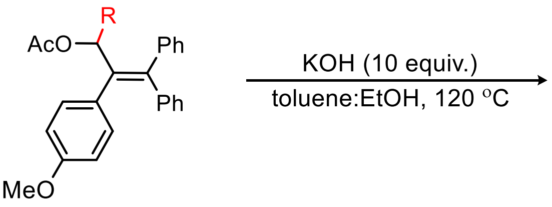

106–107

To a mixture of acetate **38/23** (0.2 mmol) and KOH (2.0 mmol) was added toluene:ethanol (9:1, 2.0 mL). The resulting mixture was stirred at 120 °C overnight. The reaction progress was monitored by TLC. Upon completion, the crude mixture was concentrated under reduced pressure. The crude residue was purified using column chromatography on neutral alumina to afford **106**–**107**.

3-(4-Methoxyphenyl)-4,4-diphenylbut-3-en-2-ol (106):

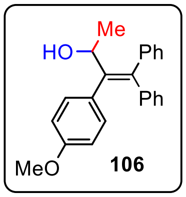

106 (53 mg, 81%) as pale yellow semi-solid; R_f = 0.25 (10% EtOAc/Hex); ^{1}H NMR (500 MHz, DMSO d_6) δ 7.38 (t, J = 7.5 Hz, 2H), 7.32–7.24 (m, 3H), 7.18–7.12 (m, 2H), 7.04 (t, J = 7.0 Hz, 2H), 6.96 (t, J = 7.0 Hz, 1H), 6.94–6.88 (m, 2H), 6.72 (dd, J = 8.5, 1.5 Hz, 2H), 4.73–4.67 (m, 1H), 4.64–4.57 (m, 1H), 3.67 (s, 3H), 1.01 (dd, J = 5.5, 3.0 Hz, 3H); ^{13}C NMR (126 MHz, DMSO d_6) δ 157.9, 143.19, 143.15, 142.5, 139.6, 132.6, 131.1, 130.2, 129.7, 128.6, 127.9, 127.2, 126.2, 112.9, 66.5, 55.2, 22.6; IR (Neat) ν_{max} 1435, 1689, 1587, 1389, 1028, 699 cm^{-1}; **HRMS (ESI)** for $C_{23}H_{22}NaO_2$ (M+Na)$^+$: calcd. 353.1512, found 353.1515.

2-(4-Methoxyphenyl)-3,3-diphenylprop-2-en-1-ol (107):

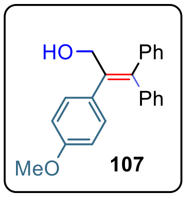

107 (45 mg, 77%) as pale yellow semi-solid; R_f = 0.25 (10% EtOAc/Hex); ^{1}H NMR (500 MHz, DMSO d_6) δ 7.36 (t, J = 8.0 Hz, 2H), 7.32–7.24 (m, 3H), 7.12–7.00 (m, 5H), 6.90–6.85 (m, 2H), 6.72 (dd, J = 8.5, 2.0 Hz, 2H), 4.69 (t, J = 5.0 Hz, 1H), 4.21 (d, J = 5.0 Hz, 2H), 3.68 (s, 3H); ^{13}C NMR (101 MHz, DMSO d_6) δ 158.1, 143.3, 142.9, 140.9, 139.7, 133.6, 131.5, 130.7, 129.9, 128.6, 128.1, 127.4, 126.6, 113.5, 63.5, 55.4; IR (Neat) ν_{max} 3412, 1682, 1586, 1415, 1112, 1025, 700 cm^{-1}; **HRMS (ESI)** for $C_{22}H_{20}NaO_2$ (M+Na)$^+$: calcd. 339.1361, found 339.1356.

2.16 Synthesis of 3-(4-methoxyphenyl)-4,4-diphenylbut-3-en-2-one (108):

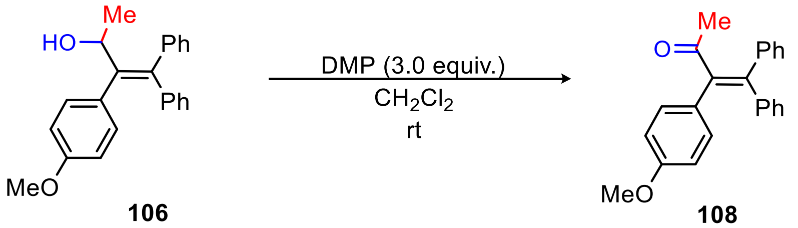

To a solution of **93** (0.2 mmol, 66 mg) in CH_2Cl_2 (2.0 mL) was added Dess-Martin Periodinane (DMP; 0.6 mmol, 255 mg). The resulting mixture was stirred at rt. The reaction progress was monitored by TLC. Upon completion of the reaction, the crude mixture was concentrated under the reduced pressure. The crude residue was purified using column chromatography on neutral alumina to afford **108** (51 mg) in 78% yield as a pale yellow liquid; R_f = 0.23 (10% EtOAc/Hex); ^{1}H NMR (400 MHz, $CDCl_3$) δ 7.37–7.29 (m, 3H), 7.25–7.10 (m, 5H), 7.04–6.93 (m, 4H), 6.72 (d, J = 8.8 Hz, 2H), 3.76 (s, 3H), 2.08 (s, 3H); ^{13}C NMR (101 MHz, $CDCl_3$) δ 206.8, 158.8, 144.0, 142.2, 142.1, 141.1, 131.3, 131.0, 130.8, 129.8, 128.41, 128.36, 127.8, 127.4, 113.8, 55.1, 31.4; IR (Neat) ν_{max} 1689, 1505, 1352, 1242, 1178, 1026, 691 cm^{-1}; **HRMS (ESI)** for $C_{23}H_{21}O_2$ $(M+H)^+$: calcd. 329.1536, found 329.1539.

2.17 Synthesis of 2-(4-methoxyphenyl)-3,3-diphenylacrylaldehyde (109):

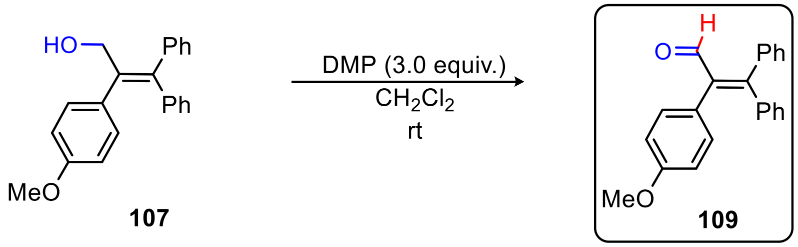

To a solution of **107** (0.2 mmol, 63 mg) in CH_2Cl_2 (2.0 mL) was added Dess-Martin Periodinane (DMP; 0.6 mmol, 255 mg). The resulting mixture was stirred at rt. The reaction progress was monitored by TLC. Upon completion of the reaction, the crude mixture was concentrated under reduced pressure. The crude residue was purified using column chromatography on neutral alumina to afford **109** (45 mg) in 72% yield as a pale yellow liquid; R_f = 0.27 (10% EtOAc/Hex); ^{1}H NMR (400 MHz, $CDCl_3$) δ 9.71 (s, 1H), 7.49–7.38 (m, 3H), 7.31–7.25 (m, 2H), 7.21–7.11 (m, 3H), 7.03–6.97 (m, 4H), 6.79–6.73 (m, 2H), 3.77 (s, 3H); ^{13}C NMR (101 MHz, $CDCl_3$) δ 193.9, 159.8, 158.9, 141.1, 139.5, 139.3, 132.5, 131.6, 131.0, 129.5, 128.5, 128.24, 128.20, 127.8, 113.6, 55.1; IR (Neat) ν_{max} 1665, 1506, 1440, 1243, 1176, 1026, 756 cm^{-1}; **HRMS (ESI)** for $C_{22}H_{19}O_2$ $(M+H)^+$: calcd. 315.1380, found 315.1381.

2.18 Synthesis of (3Z,5E)-7-Allyl-3-(4-methoxyphenyl)-4-phenyldeca-3,5,9-triene-2,7-diol (110):

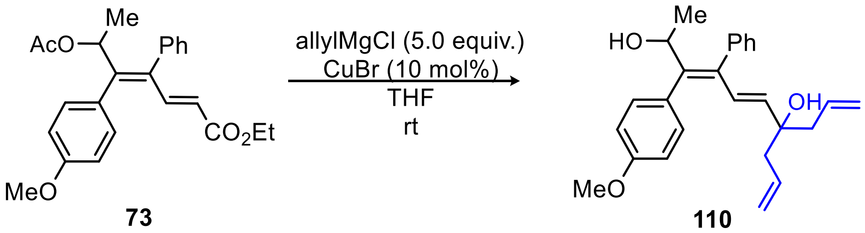

To a solution of **68** (0.2 mmol, 79 mg) and CuBr (0.02 mmol, 2.9 mg) in dry THF (2.0 mL) was added allylMgCl (2M in THF, 1.0 mmol). The resulting mixture was stirred at rt. The reaction progress was monitored by TLC. Upon completion of the reaction, the crude mixture was quenched with saturated ammonium chloride solution and extracted with ethyl acetate (2 × 10 mL). The organic layer was concentrated under reduced pressure. The crude residue was purified using column chromatography on neutral alumina to afford **102** (48 mg) in 61% yield as a pale yellow liquid; R_f = 0.13 (10% EtOAc/Hex); ^{1}H NMR (500 MHz, $CDCl_3$) δ 7.41 (t, J = 7.5 Hz, 2H), 7.35–7.30 (m, 1H), 7.22 (d, J = 8.5 Hz, 2H), 7.17 (d, J = 7.0 Hz, 2H), 6.95 (d, J = 9.0 Hz, 2H), 6.21 (d, J = 16.0 Hz, 1H), 5.64–5.52 (m, 2H), 4.96–4.88 (m, 3H), 4.88–4.80 (m, 2H), 4.46 (d, J = 3.5 Hz, 1H), 4.41 (s, 1H), 4.29–4.22 (m, 1H), 3.79 (s, 3H), 1.98 (d, J = 6.0 Hz, 4H), 0.87 (d, J = 6.5 Hz, 3H); ^{13}C NMR (101 MHz, $CDCl_3$) δ 158.5, 143.2, 140.3, 139.9, 137.0 134.9, 134.8, 132.3, 130.4, 130.0, 129.3, 128.4, 127.1, 117.7, 117.6, 113.3, 73.6, 66.6, 55.4, 45.4, 22.5; IR (Neat) ν_{max} 1606, 1606, 1507, 1440, 1241, 1175, 913 cm^{-1}; **HRMS (ESI)** for $C_{26}H_{30}NaO_3$ $(M+Na)^+$: calcd. 413.2093, found 413.2090.

2.19 Synthesis of (2E,4Z)-6-Hydroxy-5-(4-methoxyphenyl)-4-phenylhepta-2,4-dienoic acid (111):

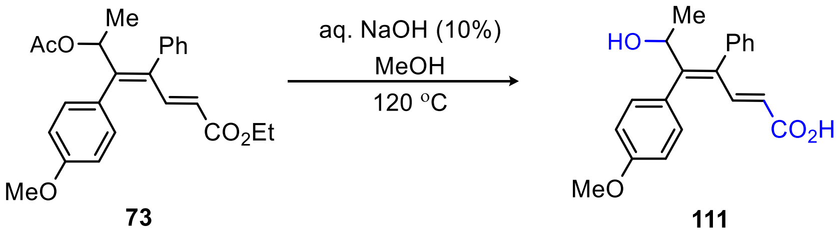

To a solution of **68** (50 mg, 1.0 equiv) in MeOH (2 mL) was added aq. NaOH (2 mL, 10.0%) in a sealed tube. The resulting mixture was stirred at 120 °C for 12 h. The reaction progress was periodically monitored by TLC. Upon completion, MeOH was evaporated under reduced pressure and the reaction mixture was acidified with HCl, extracted with DCM and concentrated under reduced pressure. The product **103** was obtained in 55% yield (36 mg) as a colorless liquid; R_f = 0.32 (10% EtOAc/Hex); ^{1}H NMR (500 MHz, $CDCl_3$) δ 12.13 (bd s, 1H), 7.48 (t, J = 7.5 Hz, 2H), 7.41 (t, J = 7.5 Hz, 1H), 7.28–7.16 (m, 5H), 7.00 (d, J

= 9.0 Hz, 2H), 5.00 (d, J = 15.5 Hz, 1H), 4.76 (br d, J = 3.5 Hz, 1H), 4.33–4.26 (m, 1H), 3.82 (s, 3H), 0.92 (d, J = 6.5 Hz, 3H); ^{13}C NMR (101 MHz, $CDCl_3$) δ 167.8, 159.1, 152.6, 145.5, 138.0 ,135.8, 132.0, 129.8, 129.1, 129.0, 127.9, 122.1, 113.5, 66.6, 55.5, 22.2; IR (Neat) ν_{max} 2920, 1681, 1604, 1507, 1244, 1175, 698 cm^{-1}; **HRMS (ESI)** for $C_{20}H_{20}NaO_4$ $(M+Na)^+$: calcd. 347.1259, found 347.1255.

3. Supplementary Discussion

3.1 Mechanistic Studies

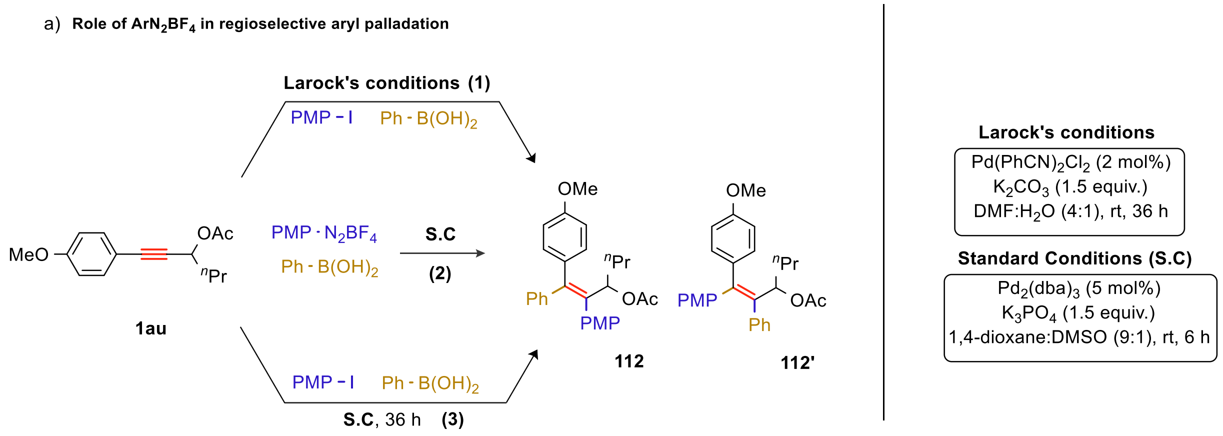

Supplementary Figure 1: Comparision experiments: reaction of **1au** under three different reaction conditions

equations	Yield in % (**112**+**112'**)	Regio-isomeric excess (re) of **112**
1	18	50
2	57	100
3	13	46

3.1.1 Larock's general procedure

A Schlenck tube was charged with the internal alkyne (0.125 mmol), *p*-iodoanisole (0.375 mmol), phenylboronic acid (0.375 mmol), K_2CO_3 (0.75 mmol), and DMF (4.0 mL), H_2O (1.0 mL). The reaction mixture was stirred at room temperature for 10 min. $PdCl_2(PhCN)_2$ catalyst (0.005 mmol in 0.05 mL of DMF) was added. The tube was stirred at room temperature (20–30 °C) for 36 h. The reaction mixture was then quenched with saturated NaCl solution (30 mL), and the aqueous layer was extracted three times with ethyl ether. The combined organic layers were dried over anhydrous Na_2SO_4, and the solvent was evaporated under reduced pressure. The product was isolated by chromatography on a silica gel eluting with hexane.

Equation 1 (Supplementary Figure 1)

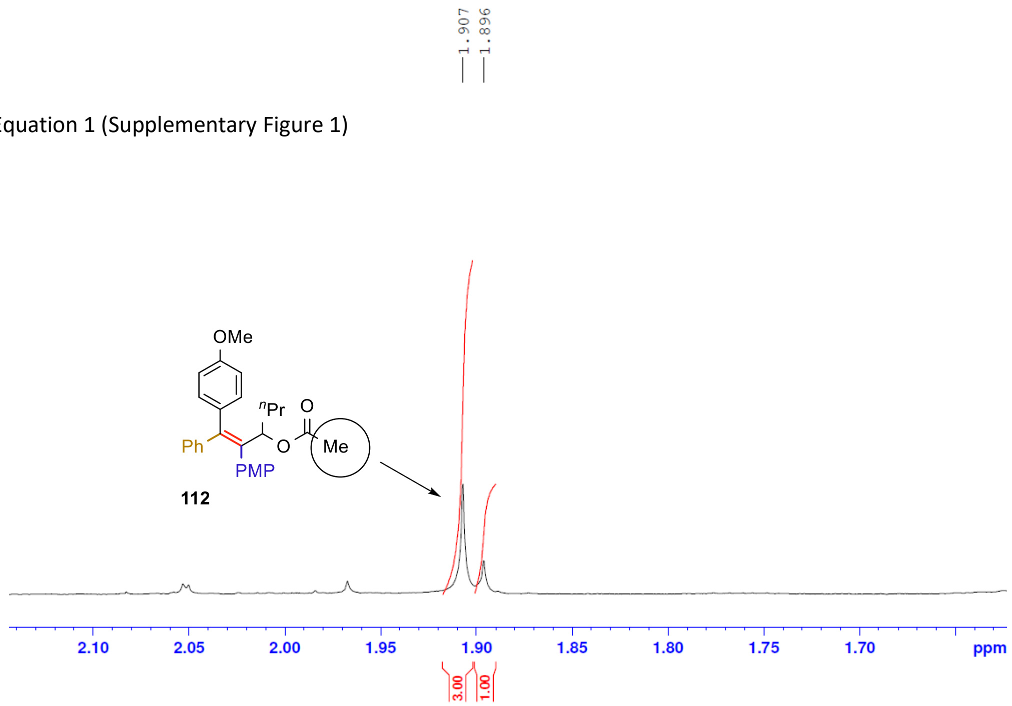

Supplementary Figure 2: Representative NMR spectrum of equation 1 (Supplementary Figure 1)

Equation 2 (Supplementary Figure 1)

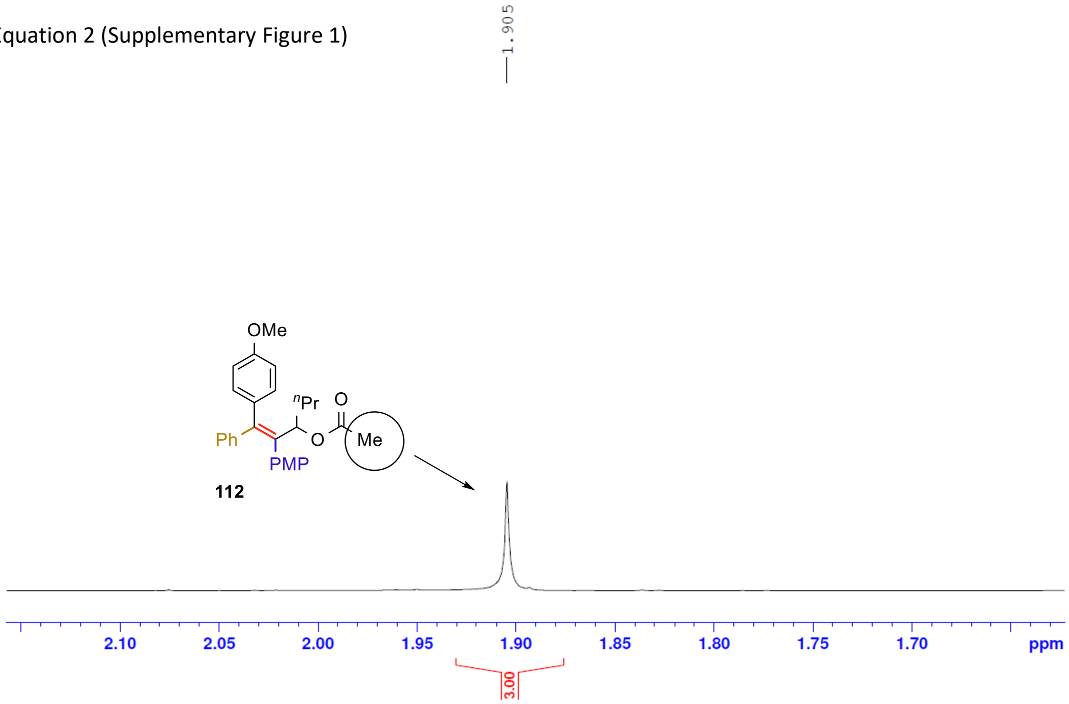

Supplementary Figure 3: Representative NMR spectrum of equation 2 (Supplementary Figure 1)

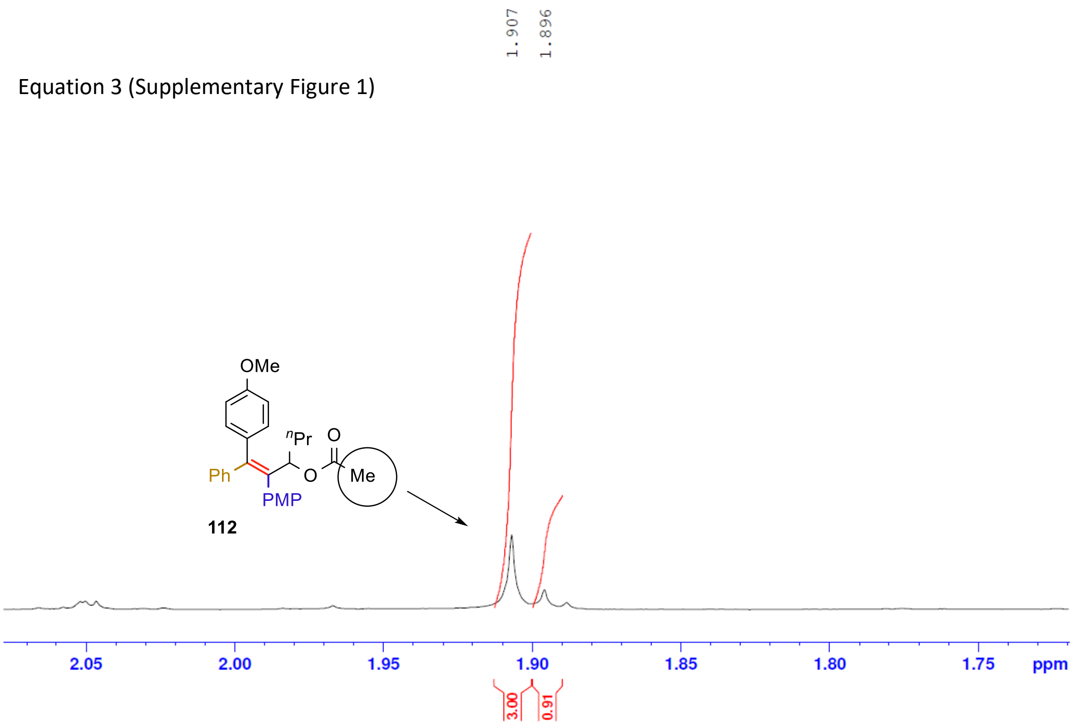

Supplementary Figure 4: Representative NMR spectrum of equation 3 (Supplementary Figure 1)

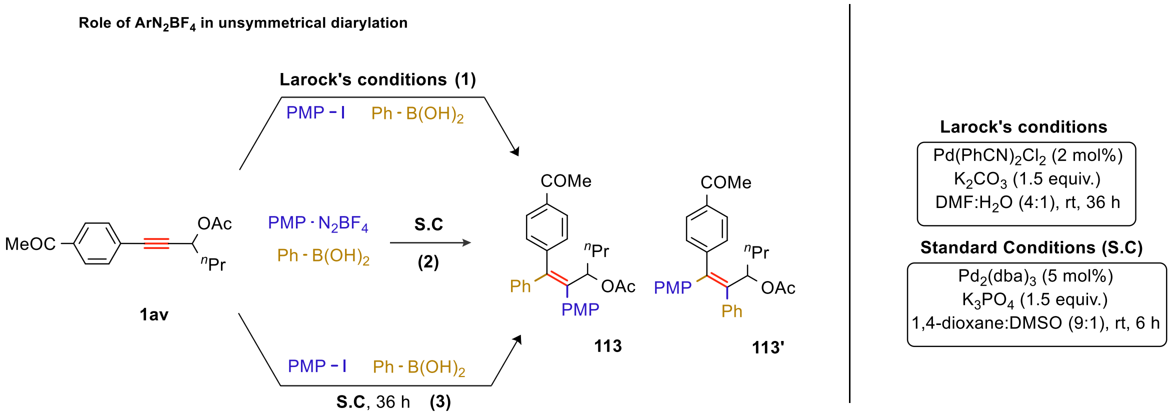

Supplementary Figure 5: Comparision experiments: reaction of **1av** in under three different reaction conditions

equations	Yield in % (**113**+**113'**)	Regio-isomeric excess (re) of **113**
1	33	56
2	74	94
3	24	20

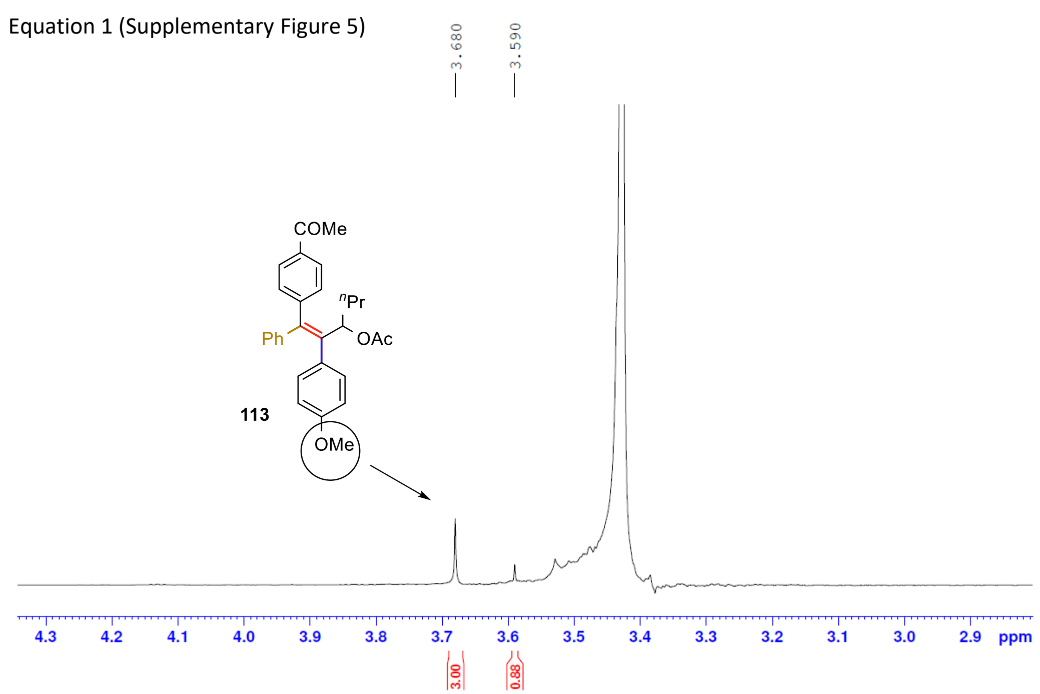

Supplementary Figure 6: Representative NMR spectrum of equation 1 (Supplementary Figure 5)

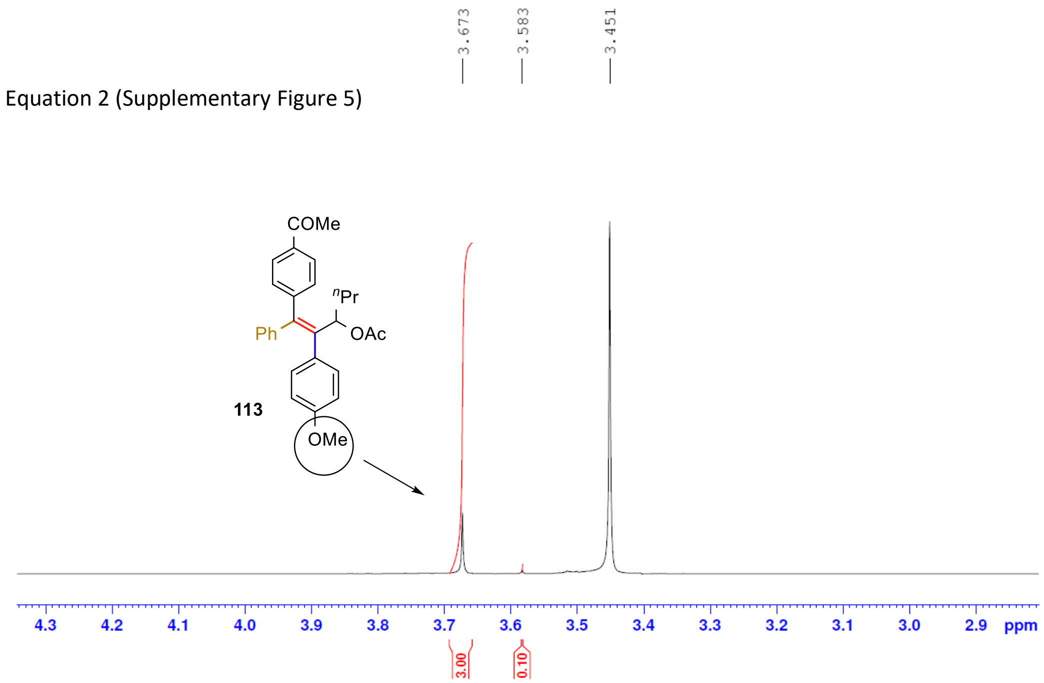

Supplementary Figure 7: Representative NMR spectrum of equation 2 (Supplementary Figure 5)

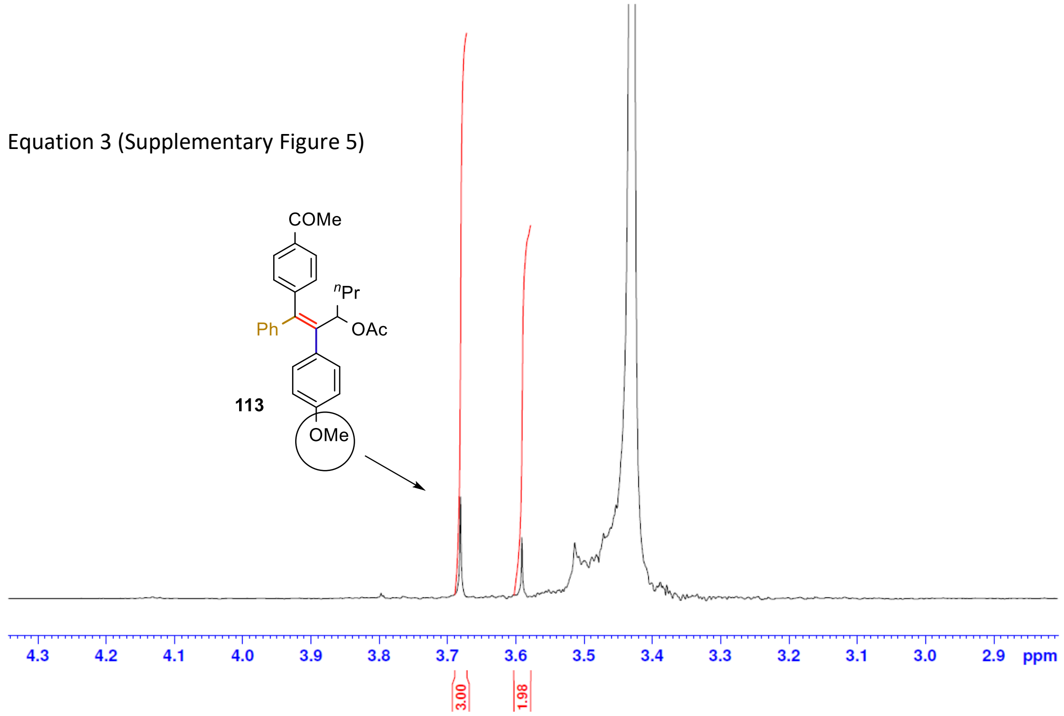

Supplementary Figure 8: Representative NMR spectrum of equation 3 (Supplementary Figure 5)

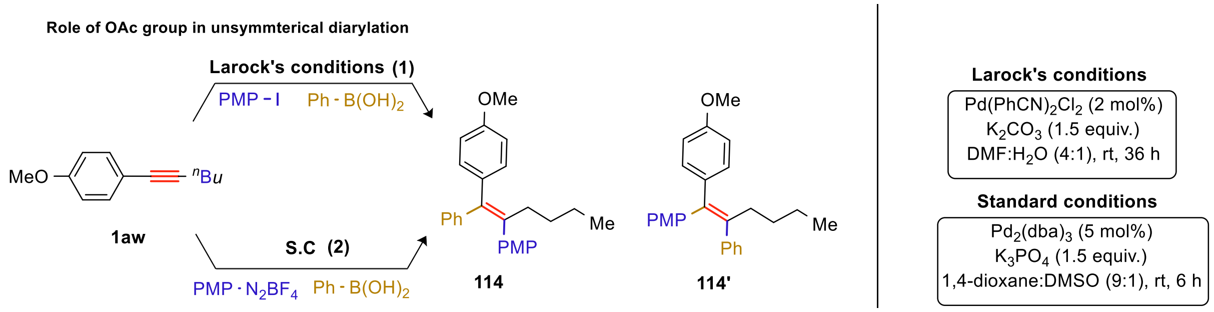

Supplementary Figure 9: Comparision experiments: reaction of **1aw** in under two different reaction conditions

equations	Yield in % (**114**+**114'**)	Regio-isomeric excess (re) of **114**
1	38	68
2	61	78

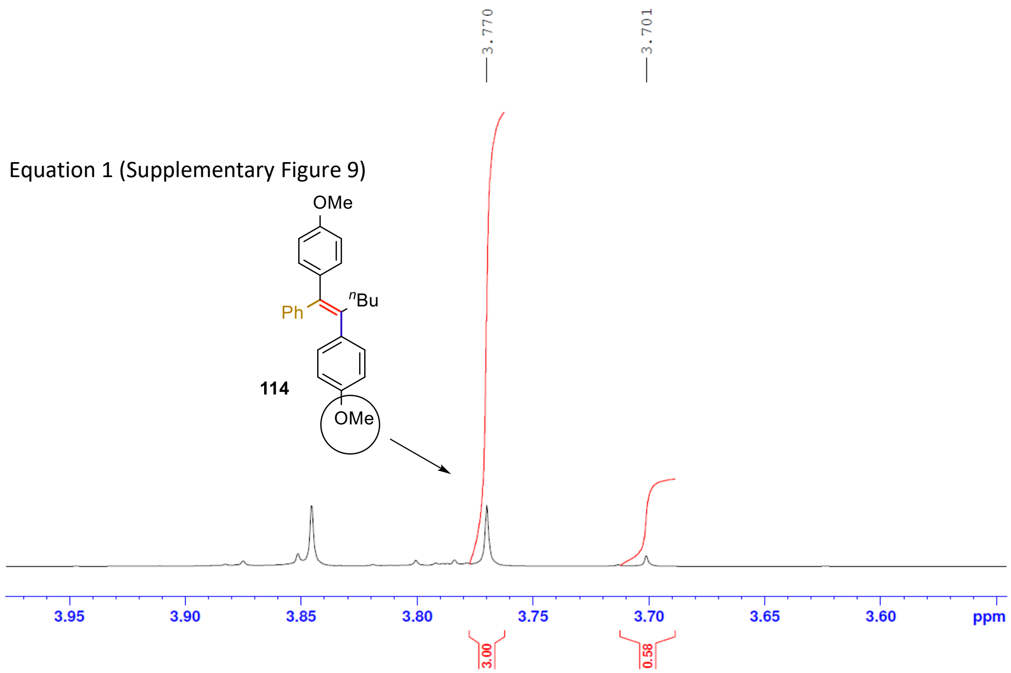

Supplementary Figure 10: Representative NMR spectrum of equation 1 (Supplementary Figure 9)

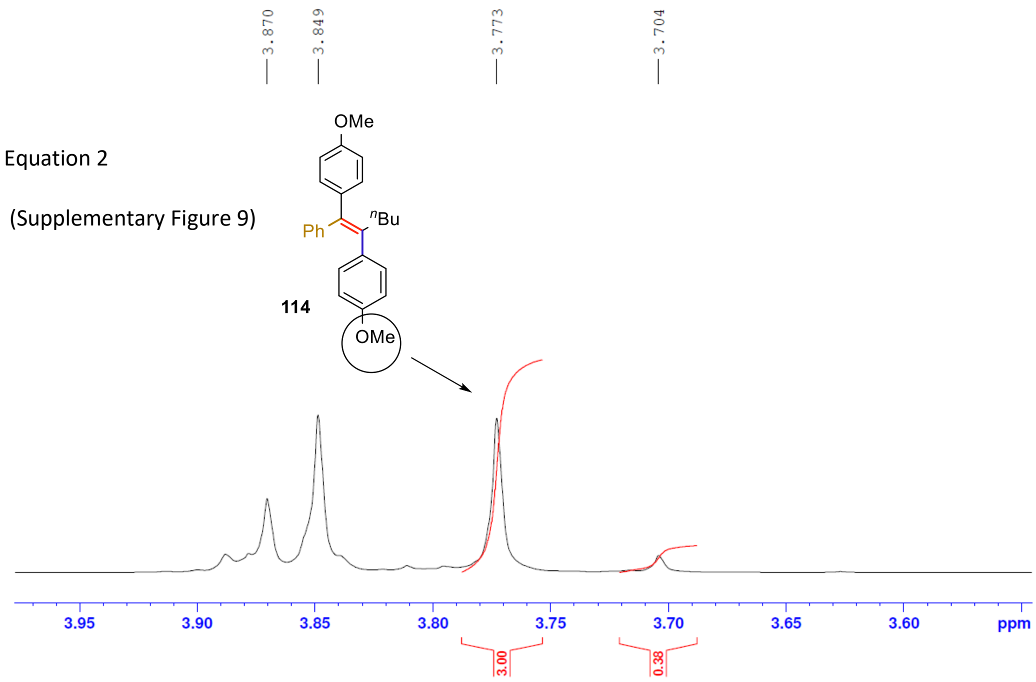

Supplementary Figure 11: Representative NMR spectrum of equation 2 (Supplementary Figure 9)

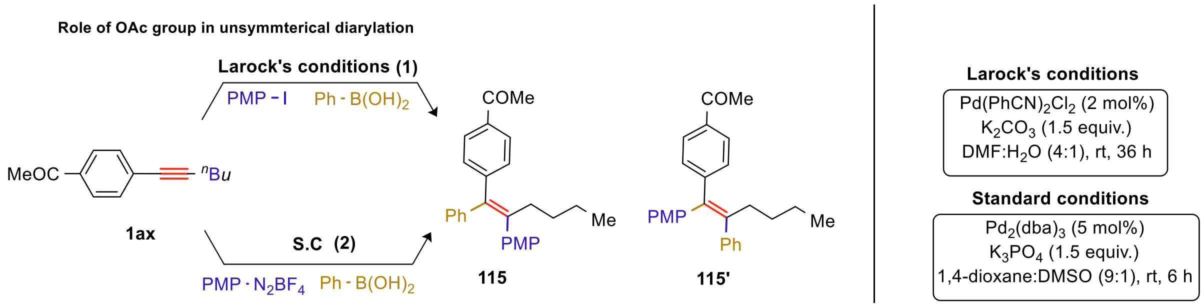

Supplementary Figure 12: Comparision experiments: reaction of **1ax** in under two different reaction conditions

equations	Yield in % (**115**+**115'**)	Regio-isomeric excess (re) of **115**
1	42	82
2	70	80

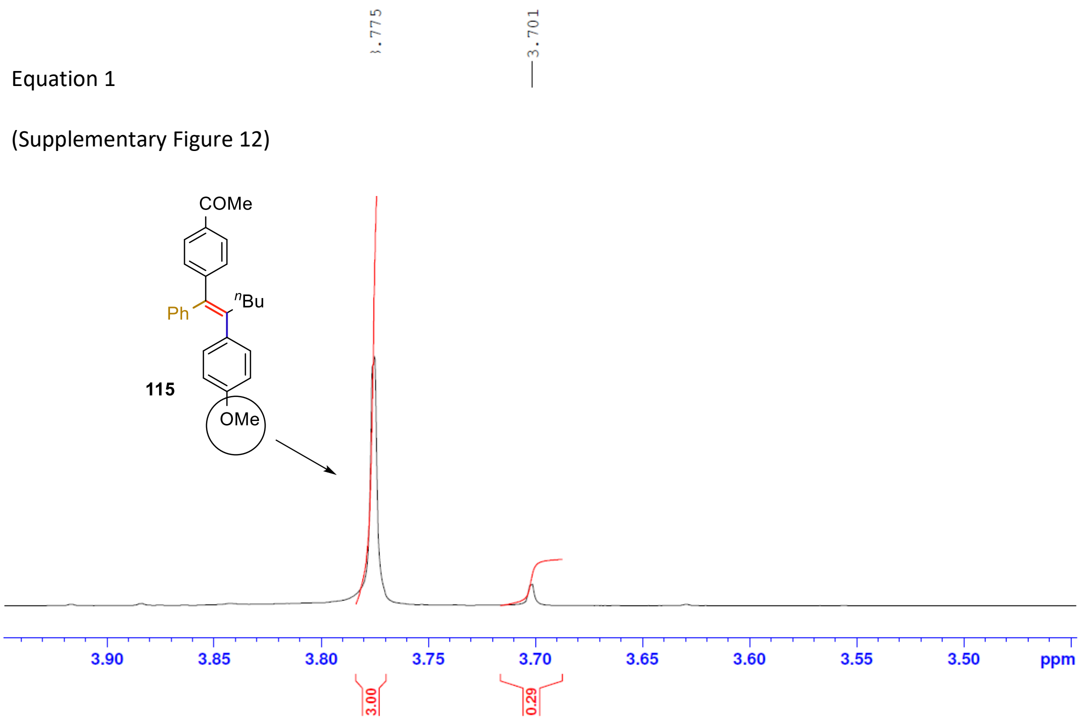

Supplementary Figure 13: Representative NMR spectrum of equation 1 (Supplementary Figure 12)

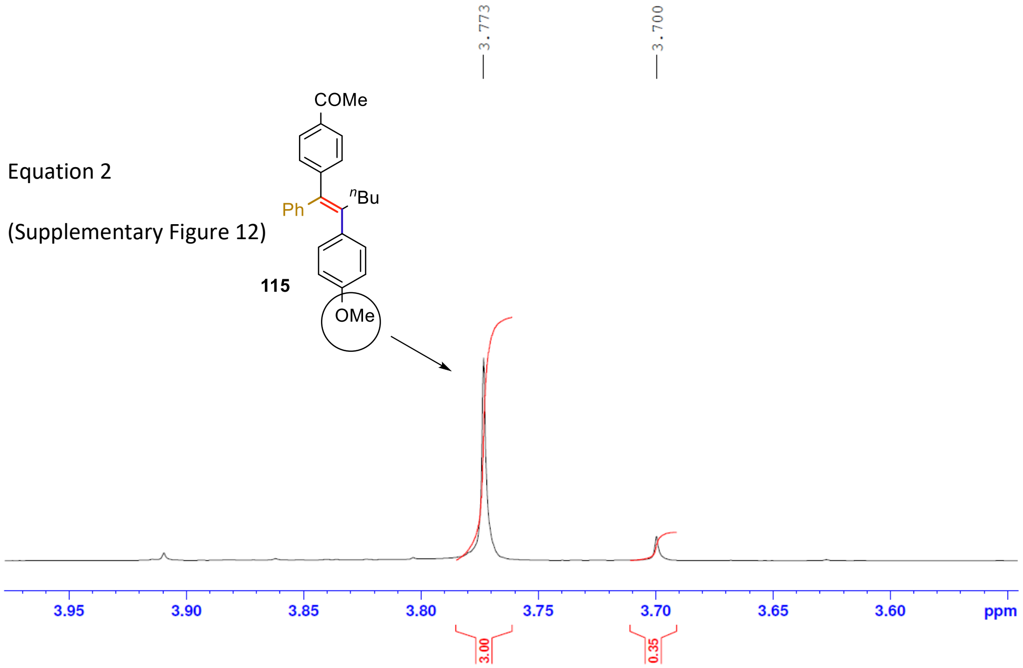

Supplementary Figure 14: Representative NMR spectrum of equation 2 (Supplementary Figure 12)

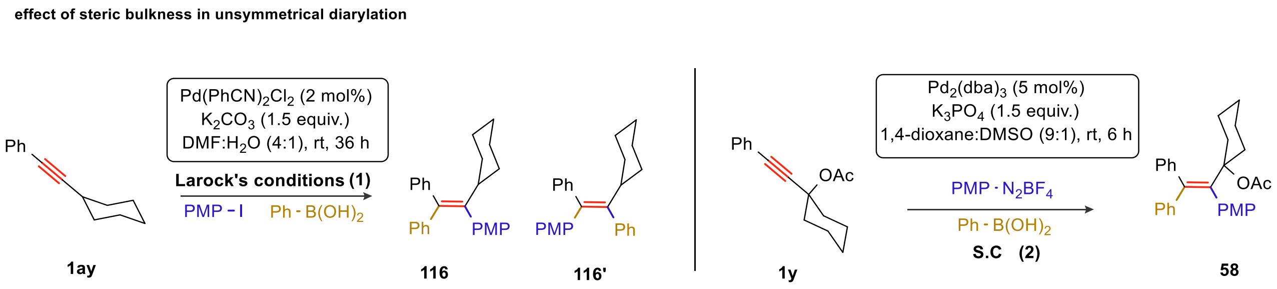

Supplementary Figure 15: Comparision experiments: reaction of **1as** in under two different reaction conditions

equations	Yield in %	Regio-isomeric excess (re)
1	28 (**116**+**116'**)	74 (**116**)
2	51 (**58**)	100

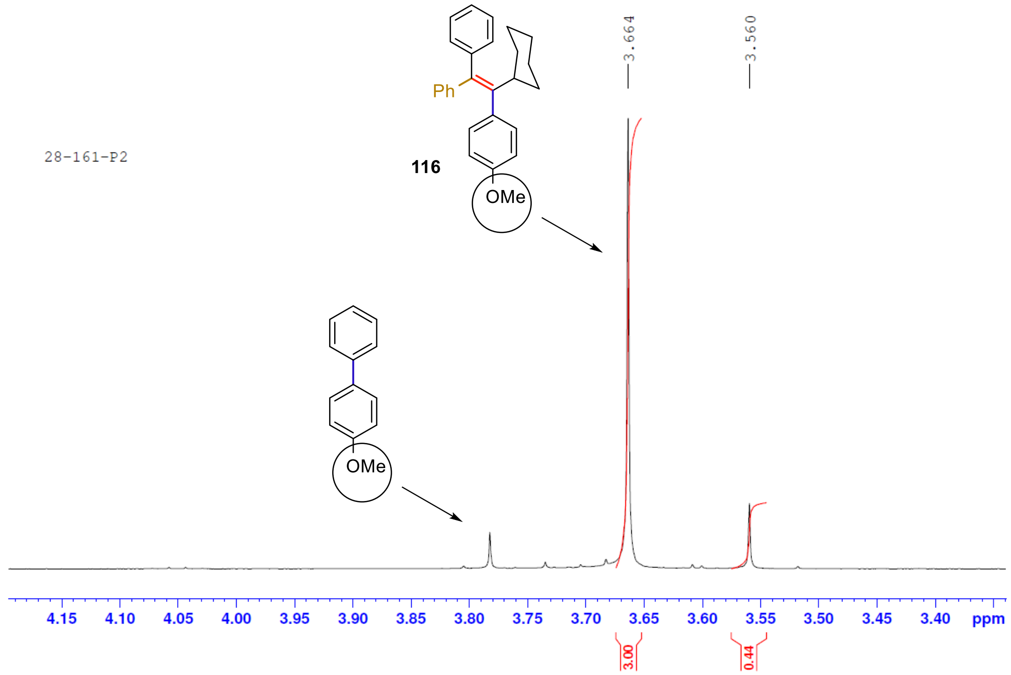

Supplementary Figure 16: Representative NMR spectrum of equation 1 (Supplementary Figure 15)

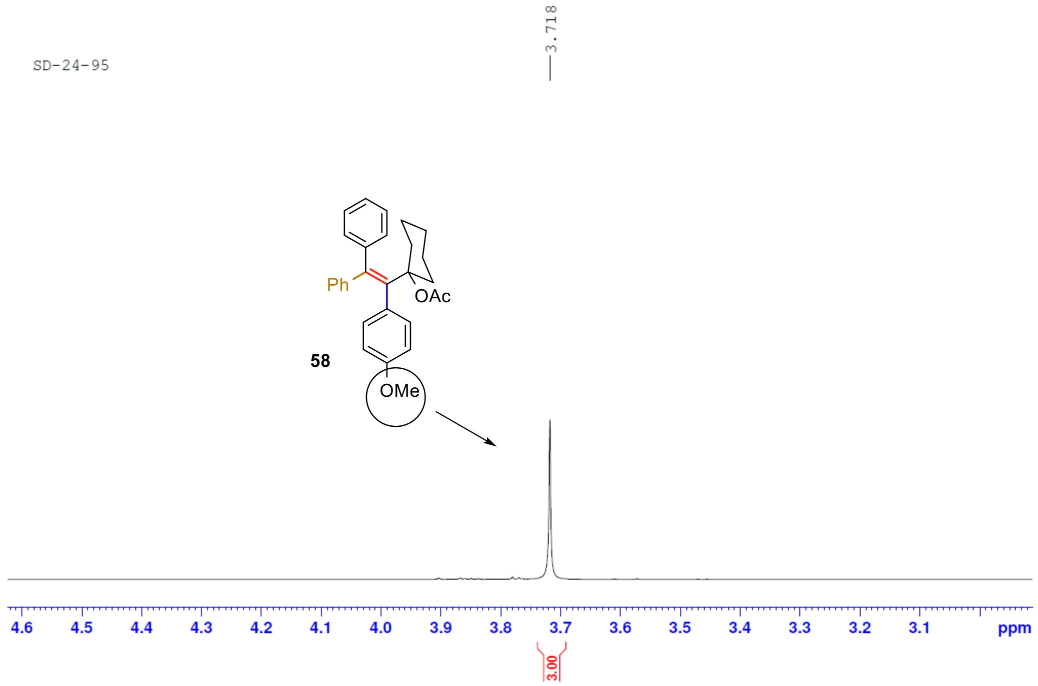

Supplementary Figure 17: Representative NMR spectrum of equation 2 (Supplementary Figure 15)

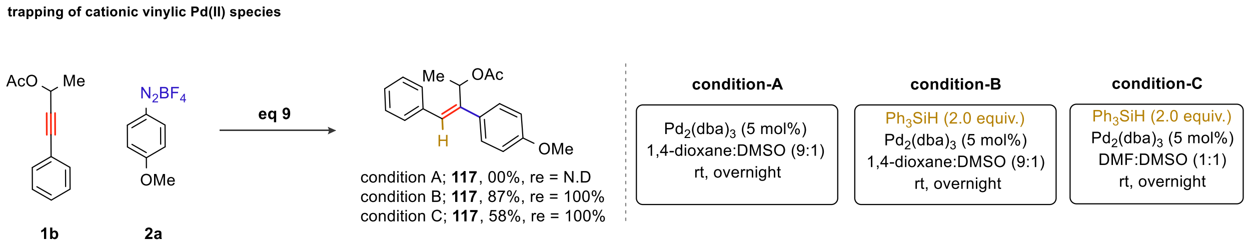

Supplementary Figure 18: Trapping of vinylic Pd(II) species in the absence of aryl boronic acid and olefin

3.12 Condition-B

A Schlenck tube was charged with **1b** (0.3 mmol), **2a** (0.9 mmol), triphenylsilane (0.6 mmol), and 1,4-dioxane:DMSO (9:1, 2.0 mL). The reaction mixture was stirred at room temperature for 6 h. The reaction progress was periodically monitored by TLC. The reaction mixture was diluted with EtOAc (20 mL) and washed with water (3 × 10 mL) and brine. The organic layer was dried over Na_2SO_4. The solvent was evaporated and purified by column chromatography over neutral alumina eluting with EtOAc/hexane to afford **117**.

3.13 Condition-C

A Schlenck tube was charged with **1b** (0.3 mmol), **2a** (0.9 mmol), triphenylsilane (0.6 mmol), DMF:DMSO (1:1, 3.0 mL). The reaction mixture was stirred at room temperature for 6 h. The reaction progress was periodically monitored by TLC. The reaction mixture was diluted with EtOAc (20 mL) and washed with water (3 × 10 mL) and brine. The organic layer was dried over Na_2SO_4. The solvent was evaporated and purified by column chromatography over neutral alumina eluting with EtOAc/hexane to afford **117**.

(*Z*)-3-(4-Methoxyphenyl)-4-phenylbut-3-en-2-yl acetate (117):

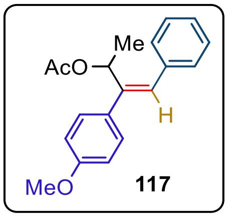

117 (95 mg, 87%) as pale yellow solid; R_f = 0.43 (10% EtOAc/Hex); M.P (147–148 °C); ^{1}H NMR (500 MHz, DMSO d$_6$) δ 7.46 (dt, *J* = 9.0, 2.0 Hz, 2H), 7.44–7.37 (m, 2H), 7.31 (d, *J* = 7.0 Hz, 3H), 6.96 (d, *J* = 9.0 Hz, 2H), 6.69 (s, 1H), 5.98 (q, *J* = 6.5 Hz, 1H), 3.78 (s, 3H), 1.93 (s, 3H), 1.31 (d, *J* = 7.0 Hz, 3H); ^{13}C NMR (126 MHz, DMSO d$_6$) δ 169.8, 159.2, 141.0, 136.9, 132.4, 131.0, 129.9, 129.2, 128.8, 127.6, 114.0, 69.0, 55.5, 21.4, 20.1; IR (Neat) ν_{max} 1736, 1509, 1370, 1241, 1035, 701 cm^{-1}; **HRMS (ESI)** for $C_{19}H_{20}O_3Na$ (M+Na)$^+$: calcd. 319.1305, found 319.1305.

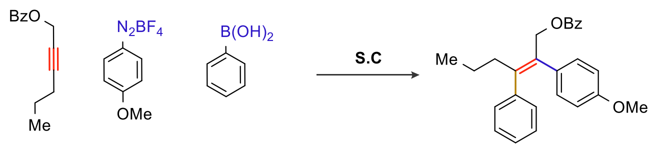

1ae **2a** **3c** **64**, 9%

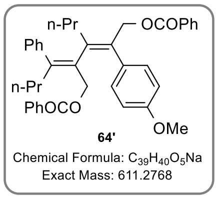

Supplementary Figure 19: Reaction of **1ae** with **2a** and **3c**

The diarylation of alkyl-substituted Pas **1ae** / **1af** independently with **2a** and **3c** under the optimized condition has led to **64** (9%) and **65** (trace). The results are incorporated in figure-2, page 4 in the manuscript. Substantial amount of arylative dimerization of alkyne has been observed (confirmed by HRMS analysis, attached herewith, of crude reaction mixture) along with unreacted precursors. Current effort is directed to address these shortcomings.

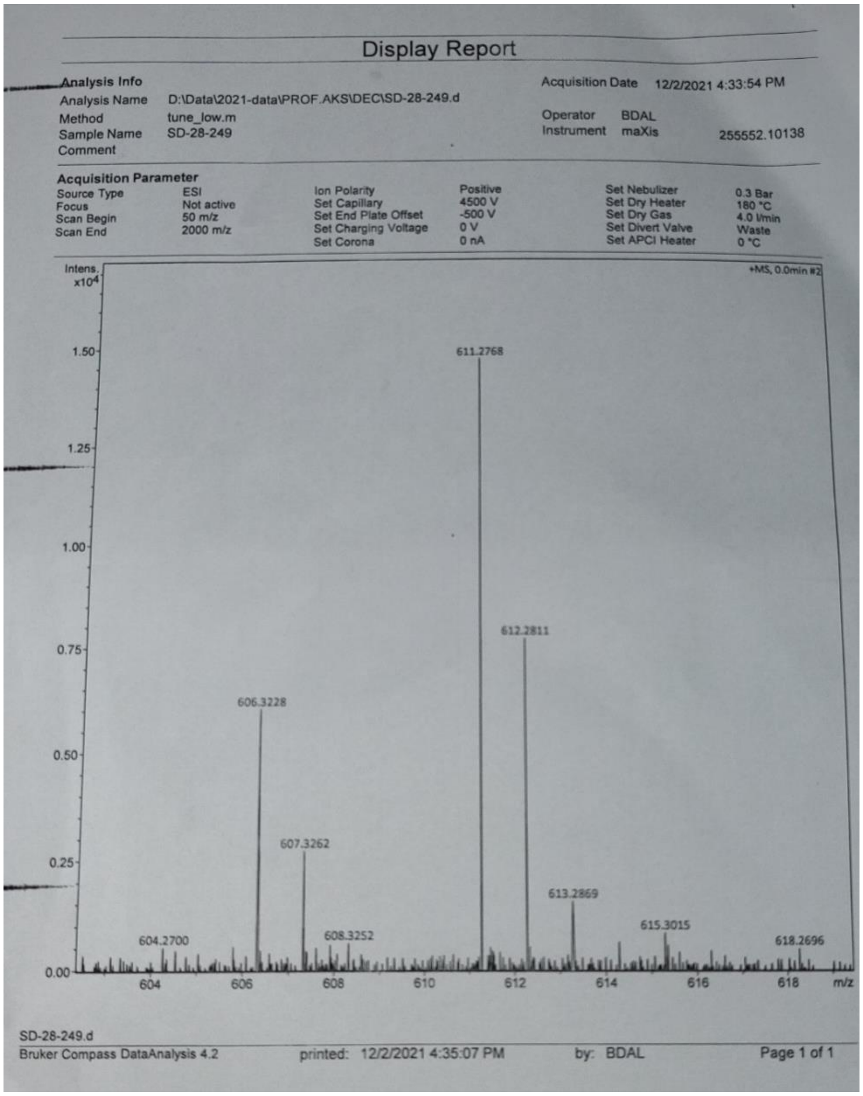

3.2 DFT Computations

3.2.1 Computational details: Following a recent DFT study on Pd-catalyzed reactions,[6] geometries (minima and transition states) were optimized at the BP86 level[7-9] of theory at 298 K and 1 atm using the Gaussian 09 software package.[10] The double-ζ basis set (LANL2DZ ECP) was used for Pd.[11-14] All other atoms were described by the 6-31G(d,p) basis set.[15-16] Frequency calculations were conducted at this level. Single point energy calculations were carried out at the M06L level of theory.[17] This method includes dispersion effects. The triple-ζ def2-TZVPP basis set was used for all atoms.[18-19] Solvent correction for 1,4-dioxane + DMSO (9:1) was obtained with the SMD[20-21]continuum solvation model as implemented in Gaussian. $Pd(DMSO)_2$ was used as starting point of the free energy profiles. The values discussed are Gibbs free energies (ΔG_{298}, kcal/mol). Non-covalent interaction analysis[22] was performed by NCIPLOT and Pymol[23]. The three dimensional images of the optimized structures were prepared using CYLview.[24]

3.2.2 Results and discussion

In agreement with our preceding study,[6] we found that $Pd(DMSO)_2$ is more stable than Pd(DMSO)(1,4-dioxane) and $Pd(1,4\text{-dioxane})_2$ by 4.8 and 4.5 kcal/mol respectively (Supplementary Figure 20). Therefore, $Pd(DMSO)_2$ was used as model for the active species of the title transformation.

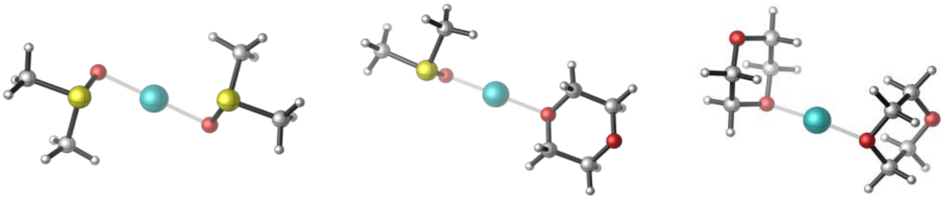

Supplementary Figure 20. Screening of potential active species (ΔG_{298}, kcal/mol). *(atom colors: blue = Pd; yellow = S; red = O; grey = C; white = H)*

3.2.3 *3-Phenyl-2-propynylacetate* as Model Substrate

We explored the reaction mechanism with density functional theory (DFT) calculations, using 3-phenyl-2-propynyl acetate **1c** and phenyl diazonium cation **b** as model substrates (Supplementary Figure 21). The transformation begins with the barrierless oxidative addition of $Pd(DMSO)_2$ (**a**) into the C-N bond of phenyl diazonium cation **b** to provide the cationic Pd-complex ^{1}**A**. This spontaneous character of this process, which is highly exergonic by 64.0 kcal/mol, is consistent with our previous findings. According to different ligand exchange scenarios, there are three putative pathways. We first evaluated the replacement of N_2 in ^{1}**A** by the C≡C bond of **1c** to afford complex ^{1}**B**, which releases 6.7 kcal/mol of free energy. A suprafacial α-aryl migration from Pd to the C≡C bond of ^{1}**B** (*syn* insertion) proceeds through transition state $^1\mathbf{TS}^{\alpha}_{\mathbf{BC}}$, found at 6.3 kcal/mol on the free energy surface, resulting in the Pd-alkenyl ester complex $^1\mathbf{C}^{\alpha}$ (see paragraph 5 for the β selectivity). This complex lies at -19.1 kcal/mol and exhibits two phenyl groups with a *trans* relationship. In contrast, the intramolecular nucleophilic addition of the ester group at C^{α} to give the Pd-alkenyl heterocyclic complex ^{1}**D** through $^1\mathbf{TS}_{\mathbf{BD}}$ (12.6 kcal/mol) needs to overcome a barrier 6.3

kcal/mol higher than **^{1}TS$^\alpha$$_{BC}$**. Furthermore, this process is endergonic by 16.5 kcal/mol, so this pathway will not be discussed further.

The possibility of the ester acting as a Directing Group (DG) was also envisaged. Exchange of N_2 and DMSO in **^{1}A** to give chelate **^{1}E** was found exergonic by 4.5 kcal/mol. α-Aryl migration to form intermediate **^{1}F$^\alpha$** (-14.3 kcal/mol) was achieved through **^{1}TS$^\alpha$$_{EF}$** (10.7 kcal/mol). However, the corresponding barrier is 2.2 kcal/mol higher than that leading to **^{1}TS$^\alpha$$_{BC}$**, which means that it is not a preferred pathway. We have also considered the sole coordination of the ester group **c1**, providing complex **^{1}G** after release N_2. This process is yet endergonic by 1.8 kcal/mol. Thus, comparing all the options, the bottom reaction pathway (blue) appears much more favorable.

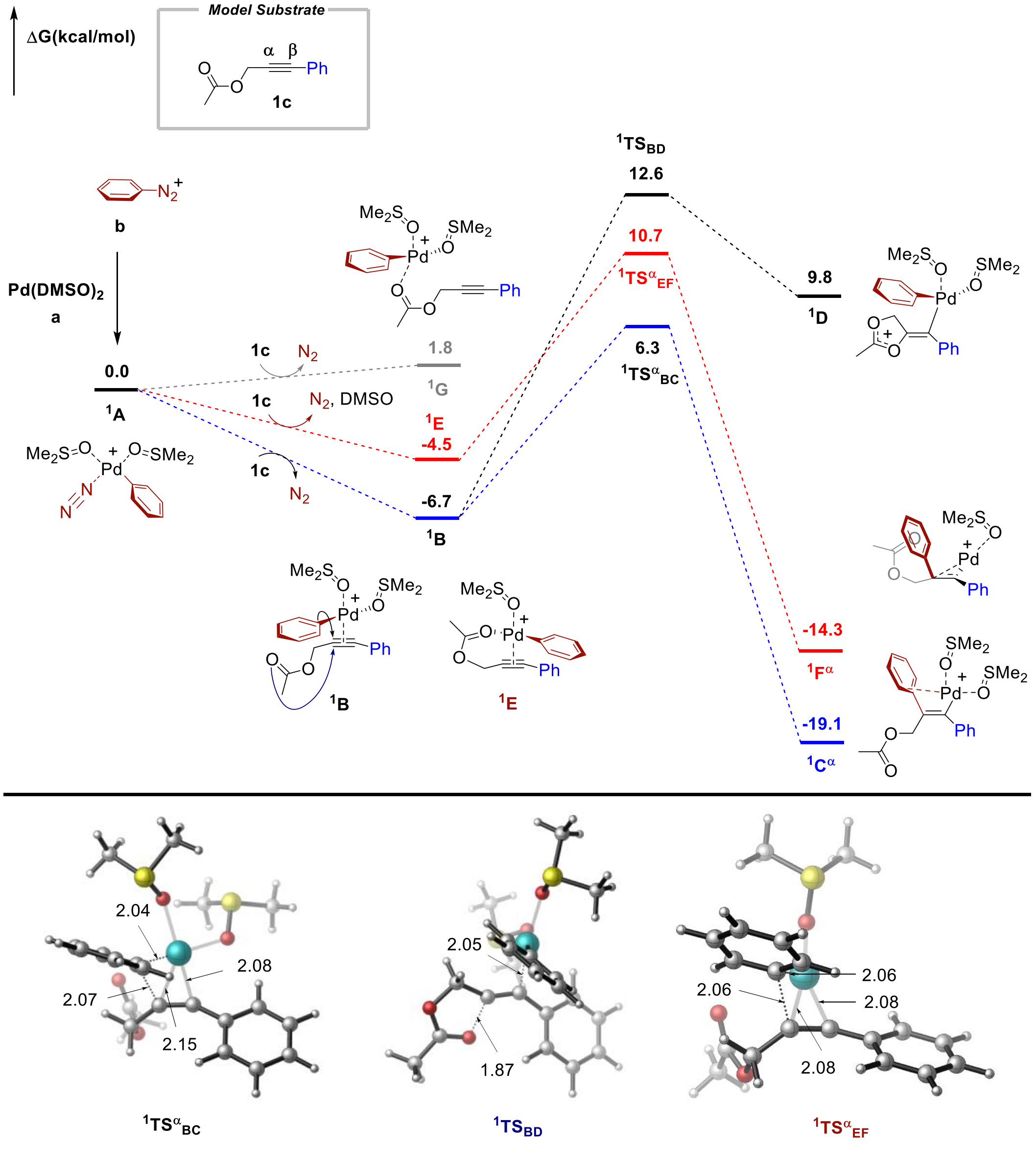

Supplementary Figure 21. Free-energy profile (ΔG_{298}, kcal/mol): *Part 1a (atom colors: blue = Pd; yellow = S; red = O; grey = C; white = H).*

The ligand exchange between $^{1}\mathbf{C}^{\alpha}$ and $PhB(OH)_3^-$ (**d**) is exergonic, leading to complex $^{1}\mathbf{H}$ (-41.0 kcal/mol) with the chelation of one borate oxygen and the *ipso* and *ortho* aryl carbons to Pd (Supplementary Figure 22). Transmetalation through $^{1}\mathbf{TS_{HI}}$ (-36.1 kcal/mol) delivers $^{1}\mathbf{I}$ (-62.6 kcal/mol) in which boric acid serves as L-type ligand. Exchange of boric acid by DMSO then dispenses $^{1}\mathbf{J}$ (-68.9 kcal/mol). Reductive elimination of $^{1}\mathbf{J}$ passes through transition state $^{1}\mathbf{TS_{JK}}$ (-54.0 kcal/mol, requiring 14.9 kcal/mol from $^{1}\mathbf{J}$) to provide $^{1}\mathbf{K}$ (-78.8 kcal/mol). Ligation of the *ipso*-carbon and *ortho*-carbon of phenyl moieties makes the (DMSO)Pd(0) intermediate $^{1}\mathbf{K}$ stable. Finally, the desired product $^{1}\mathbf{L}$ is liberated from $^{1}\mathbf{K}$, which requires 11.4 kcal/mol of free energy, along with the regeneration of the active catalyst species a for further use.

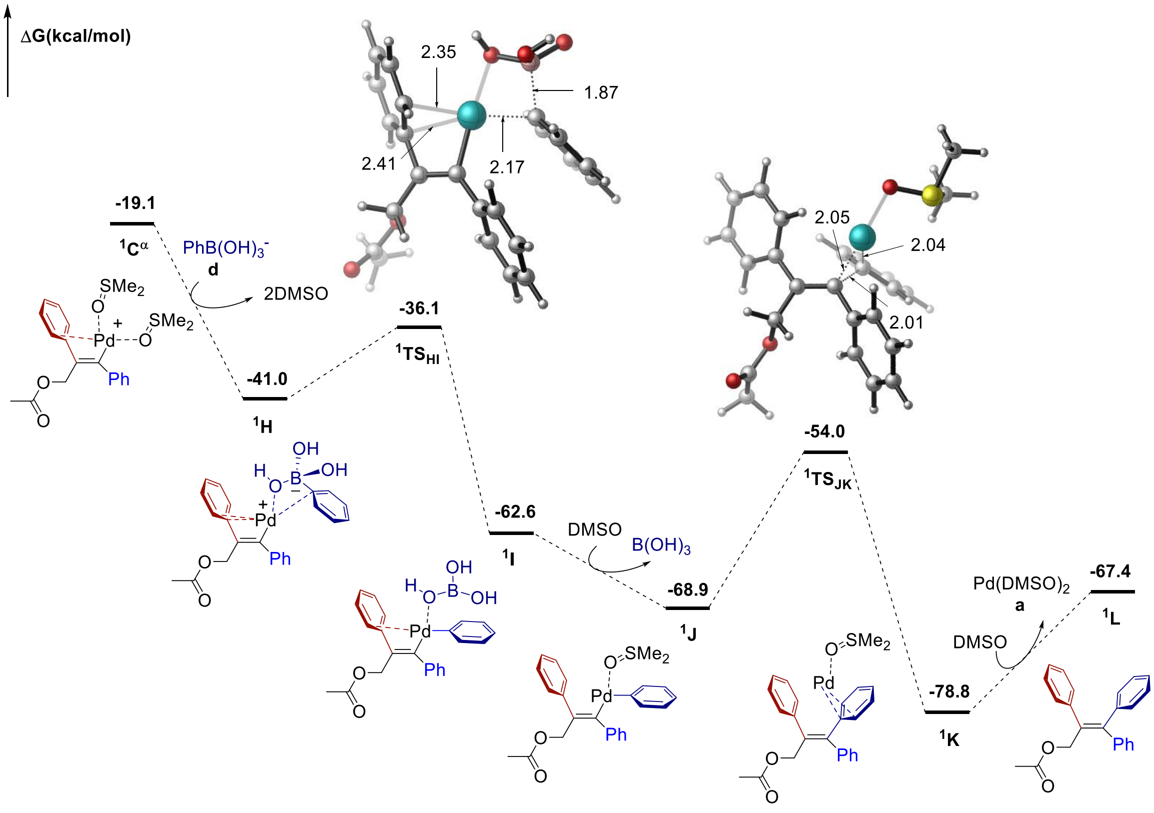

Supplementary Figure 22. Free-energy profile (ΔG_{298}, kcal/mol): *Part 2a*: *(atom colors: blue = Pd; yellow = S; red = O; grey = C; white = H)*

3.2.4 4-Phenyl-3-butynylacetate as Model Substrate

With one more carbon in the tether, the formation of alkyne complex $^{2}\mathbf{B}$ (-5.7 kcal/mol) and its carbopalladation through $^{2}\mathbf{TS^{\alpha}_{BC}}$ (5.2 kcal/mol) remains the most favorable route (Supplementary Figure 23). Forming $^{2}\mathbf{C}^{\alpha}$ (-20.5 kcal/mol) is kinetically and also thermodynamically preferred.

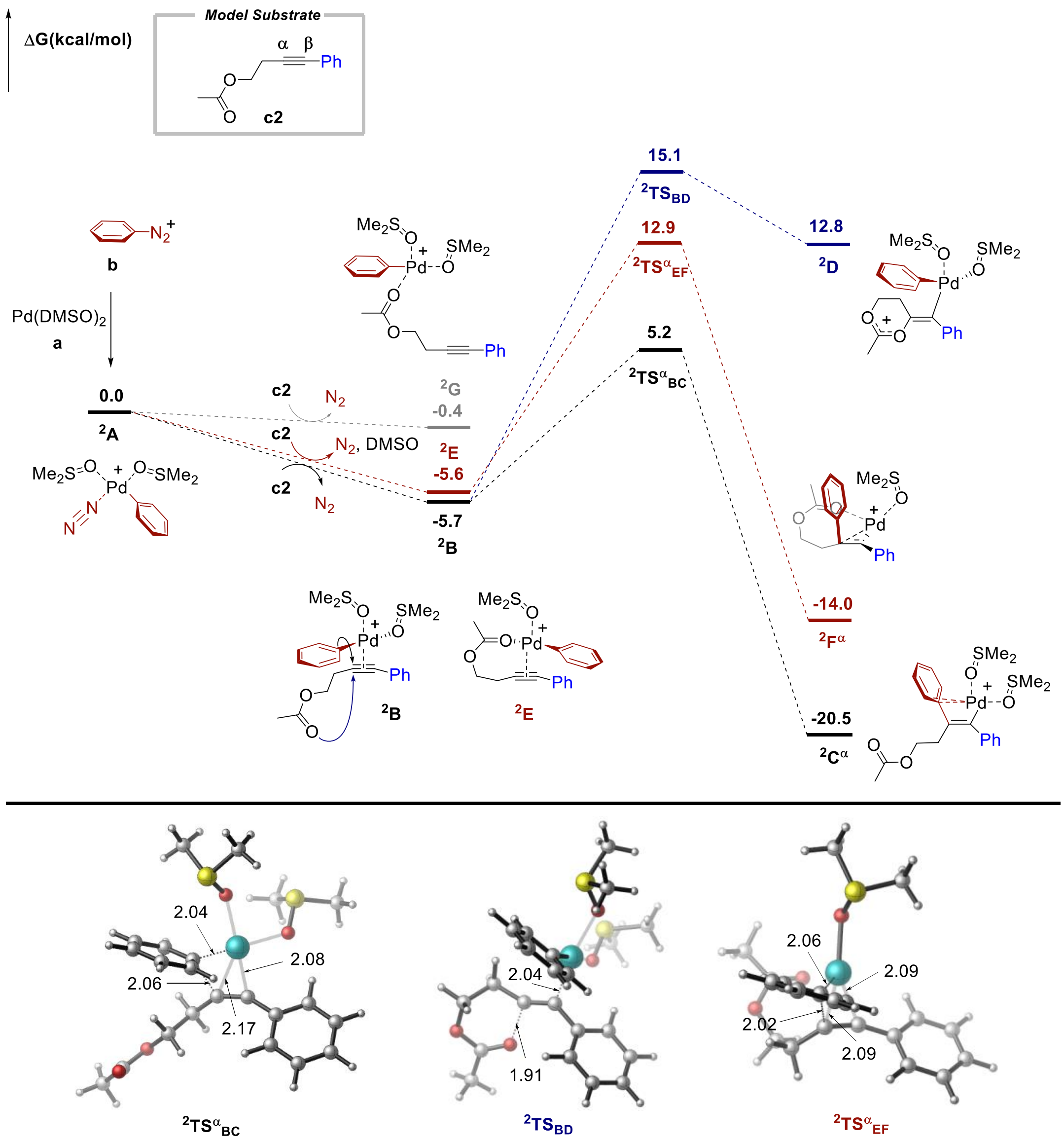

Supplementary Figure 23. Free-energy profile (ΔG_{298}, kcal/mol): *Part 1b (atom colors: blue = Pd; yellow = S; red = O; grey = C; white = H)*

There is not significant changes for the rest of the mechanism, from **^{2}C$^\alpha$** to **^{2}L** (Supplementary Figure 24).

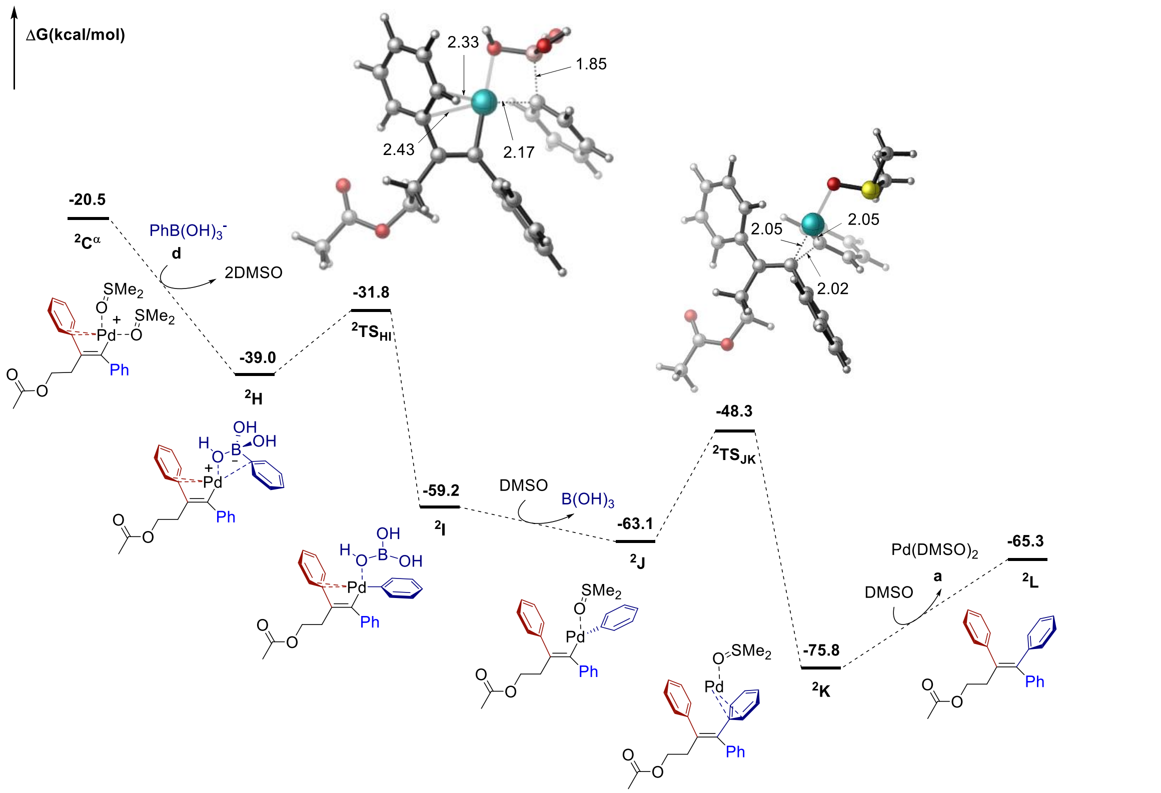

Supplementary Figure 24. Free-energy profile (ΔG_{298}, kcal/mol): *Part 2b (atom colors: blue = Pd; yellow = S; red = O; grey = C; white = H)*

3.2.5 5-Phenyl-4-pentynylacetate as Model Substrate

By increasing the tether length to 3 carbons as in **c3** (Supplementary Figure 25), the preference for the chelate complex is even more pronounced (**^{3}E**, -8.1 kcal/mol vs -7.1 kcal/mol for the alkyne complex **^{3}B**). It even becomes possible to use the internal ester oxygen for chelation, yet the corresponding complex **^{3}G** (-4.5 kcal/mol) is less stable than the carbonyl complex **^{3}E** by 3.6 kcal/mol. Once again, the lowest-lying carbopalladation transition state (**$^3TS^α_{BC}$**, 5.1 kcal/mol) connects the simple alkyne complex **^{3}B** to the **$^3C^α$** species (-20.3 kcal/mol).

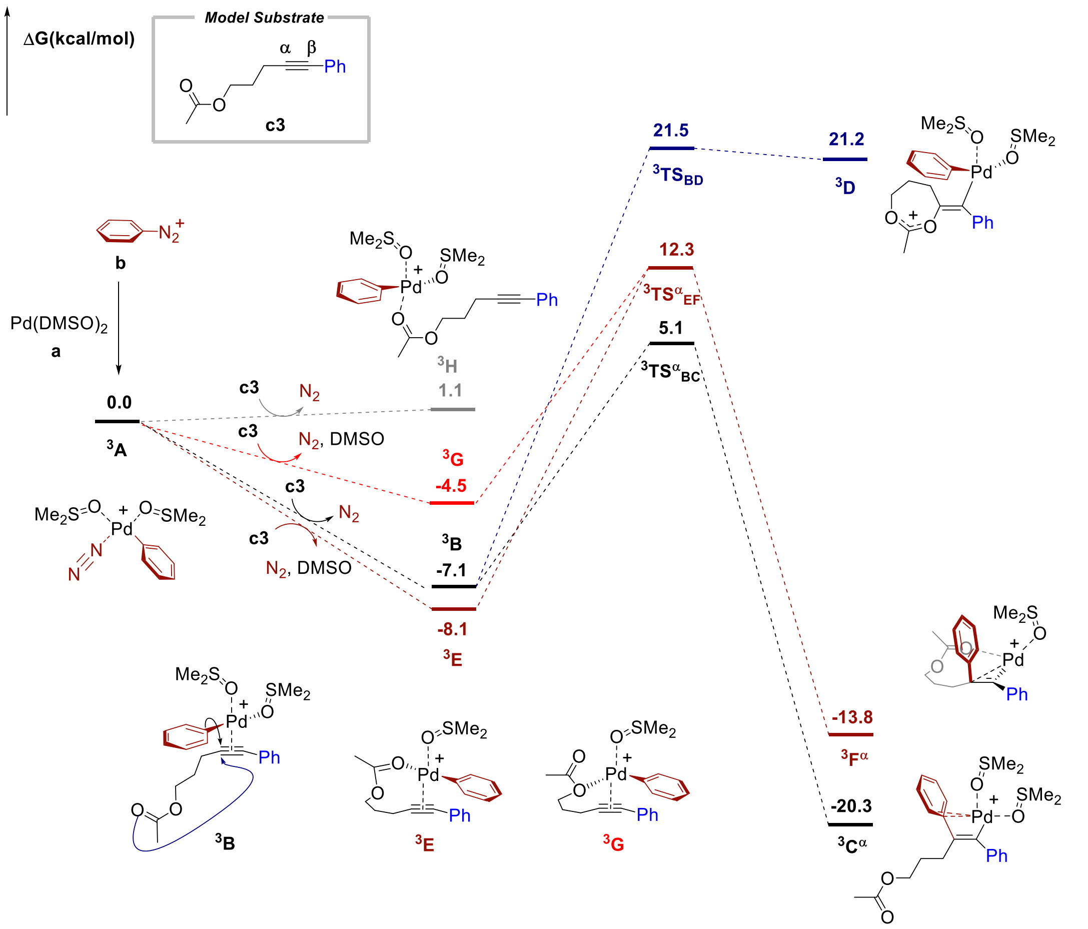

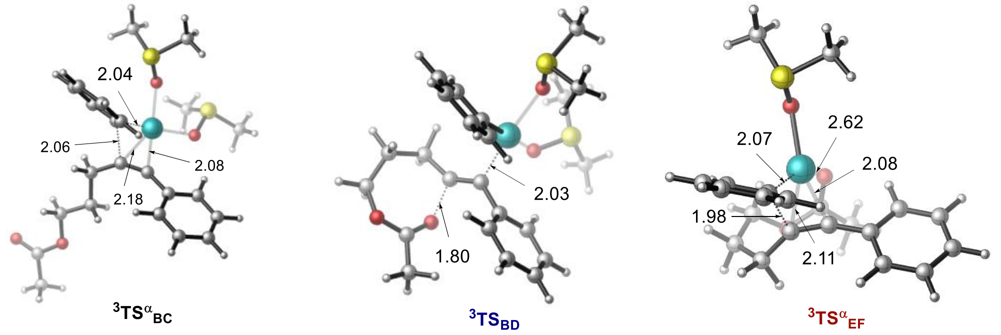

Supplementary Figure 25. Free-energy profile (ΔG_{298}, kcal/mol): *Part 1c (atom colors: blue = Pd; yellow = S; red = O; grey = C; white = H)*

The free energy profile for the subsequent transmetallation, reductive elimination and catalyst regeneration (Supplementary Figure 26) remains similar from those presented above.

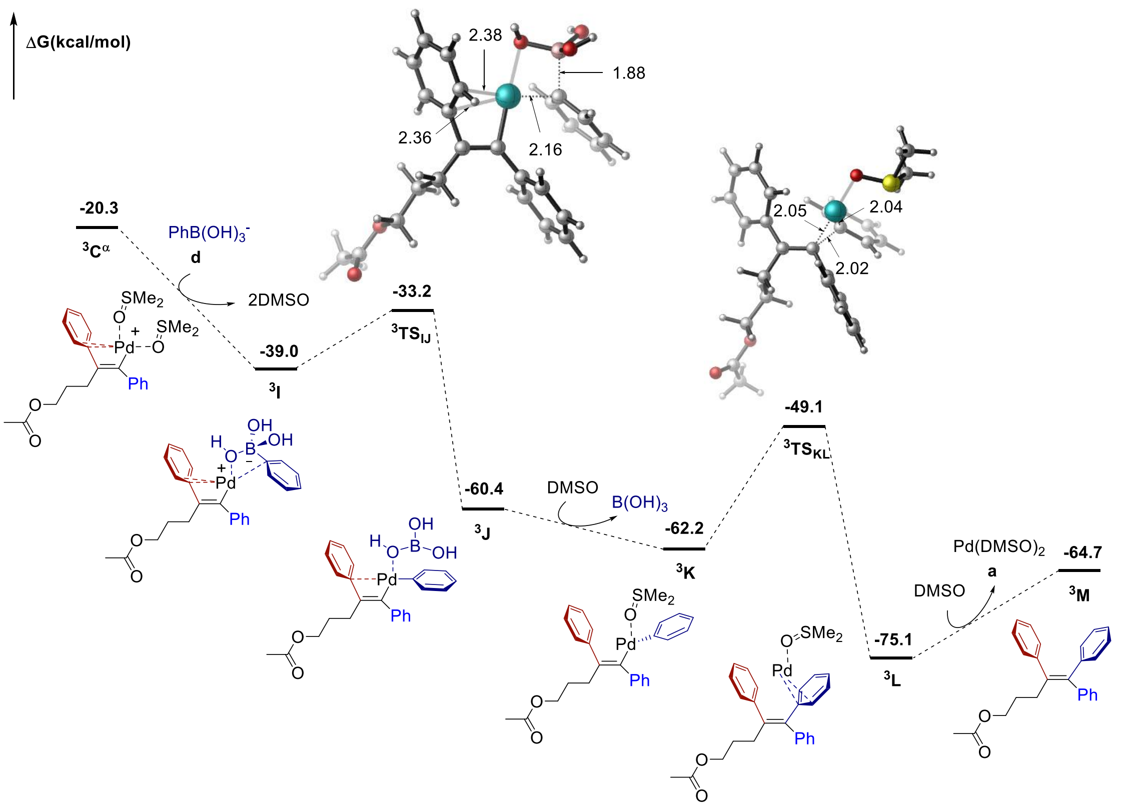

Supplementary Figure 26. Free-energy profile (ΔG_{298}, kcal/mol): *Part 2c (atom colors: blue = Pd; yellow = S; red = O; grey = C; white = H)*

3.2.6 6-Phenyl-5-hexynylacetate as Model Substrate

The same conclusions as above have been reached with the 4-carbon tether substrate **c4** (Supplementary Scheme 27), i.e. a preferred carbopalladation from the alkyne complex **^{4}B** (-7.4 kcal/mol).

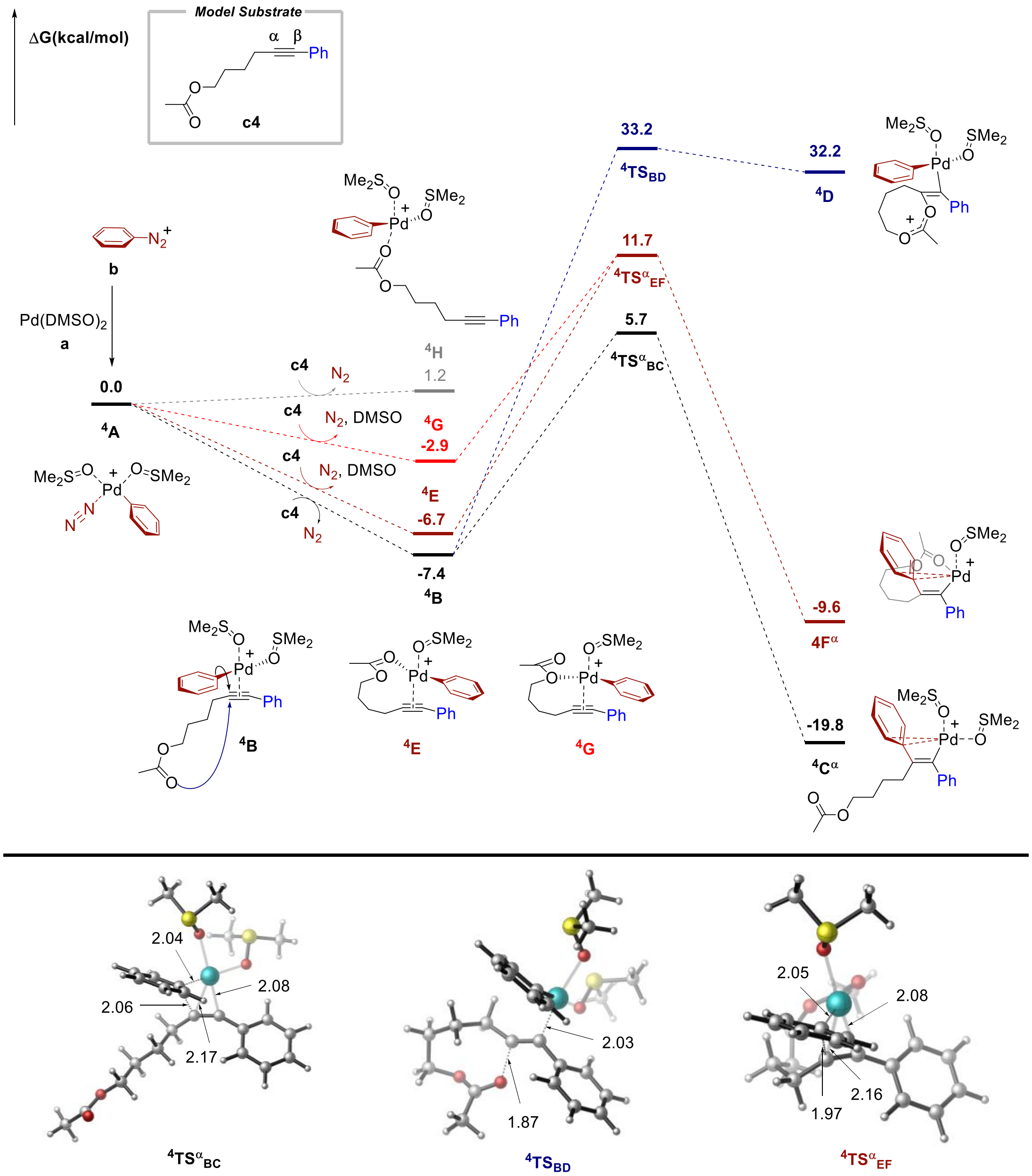

Supplementary Scheme 27. Free-energy profile (ΔG_{298}, kcal/mol): *Part 1d (atom colors: blue = Pd; yellow = S; red = O; grey = C; white = H)*

The rest of the mechanism (Supplementary Figure 28) also remains consistent with the above computations.

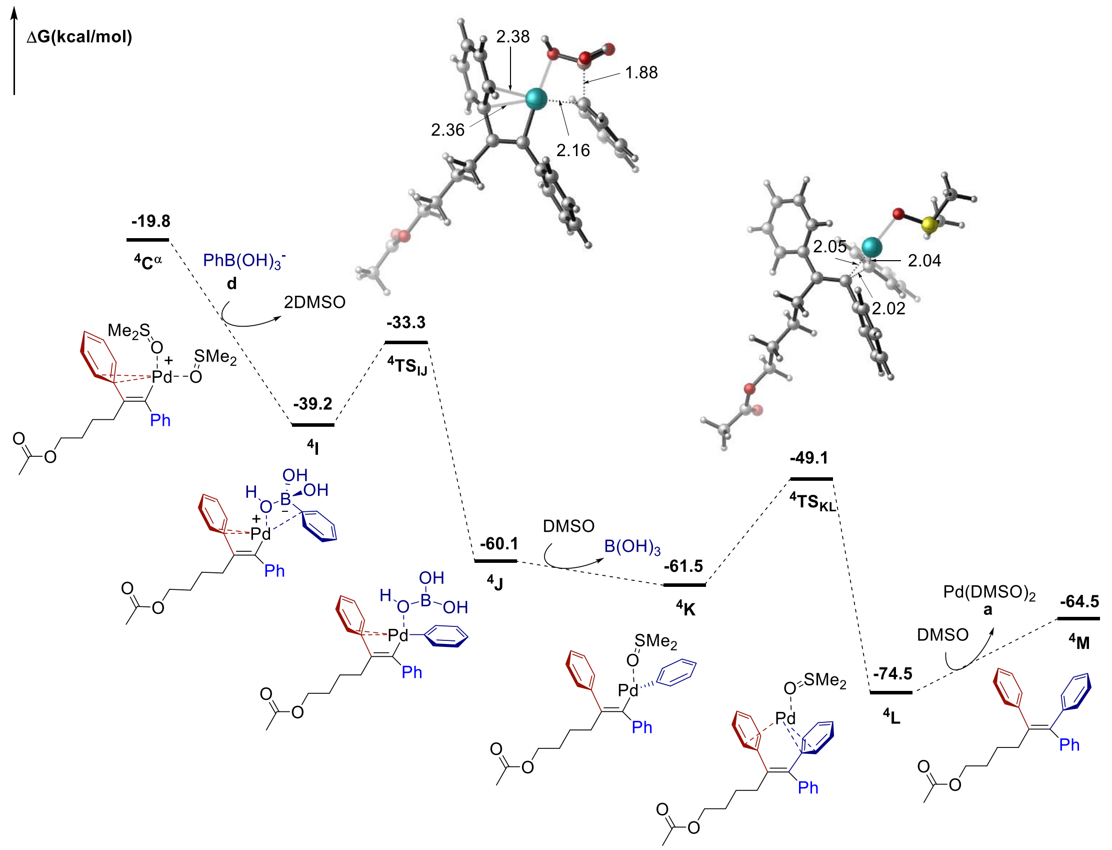

Supplementary Figure 28. Free-energy profile (ΔG_{298}, kcal/mol): *Part 2d (atom colors: blue = Pd; yellow = S; red = O; grey = C; white = H)*

3.2.7 Regioselectivity

According to the calculations presented above, the β-arylation is always disfavored over the α-arylation process. To rationalize this selectivity, we performed a distortion/interaction analysis of the transition states.[25-35] All the aryl migration transition states $^{\mathbf{n}}\mathbf{TS}^{\alpha}_{\mathbf{BC}}$ and $^{\mathbf{n}}\mathbf{TS}^{\beta}_{\mathbf{BC}}$ (n = 1, 2, 3, 4) were divided into two fragments, the substrate fragment and the arylpalladium fragment.

With n = 1 (Supplementary Figure 29), the larger rotation angle for β-aryl migration (see Supplementary Figure 16: 36.12 ° for $^{\mathbf{1}}\mathbf{TS}^{\beta}_{\mathbf{BC}}$, 7.34 ° for $^{\mathbf{1}}\mathbf{TS}^{\alpha}_{\mathbf{BC}}$) leads to a larger distortion energy (+6.0 kcal/mol). Analysis of the contributions from the arylpalladium and substrate fragments for each TS suggests that arylpalladium (+3.68 kcal/mol) and substrate distortion (+2.32 kcal/mol) both contribute to the relative difference in distortion energy between the TSs. Study of the interaction energy reveals a stabilizing effect in $^{\mathbf{1}}\mathbf{TS}^{\beta}_{\mathbf{BC}}$ (-1.9 kcal/mol) which does not compensate the distortion energy (+6.0 kcal/mol). Non-covalent interaction analysis was performed to decipher the specific contribution of different interactions to the differences in total interaction energy. Although $^{\mathbf{1}}\mathbf{TS}^{\beta}_{\mathbf{BC}}$ has a greater level of repulsive interactions from lone pair repulsion relative to $^{\mathbf{1}}\mathbf{TS}^{\alpha}_{\mathbf{BC}}$, it also features more stabling attractive NCIs, ultimately resulting in an overall more negative total interaction energy. On this basis, we propose that distortion (arylpalladium and substrate) is the major contributor to the observed regioselectivity with substrate **1c**.

With n = 2 (Supplementary Figure 29), because of a compensation of the weak distortion and interaction energy differences, there is virtually no energy difference between α- and β-arylation transition states ($\Delta\Delta E^{\ddagger}$ = -0.6 kcal/mol). However, there is a significant difference between the free energies of activation ($\Delta\Delta G^{\ddagger}$ = +6.5 kcal/mol). Without solvent correction, $\Delta\Delta G^{\ddagger}$ drops to 1.7 kcal/mol, still in favor of the α-arylation, while $\Delta\Delta H^{\ddagger}$ is slightly in favor of the β-arylation (-0.4 kcal/mol). It is thus an entropy factor that governs the selectivity in this specific case.

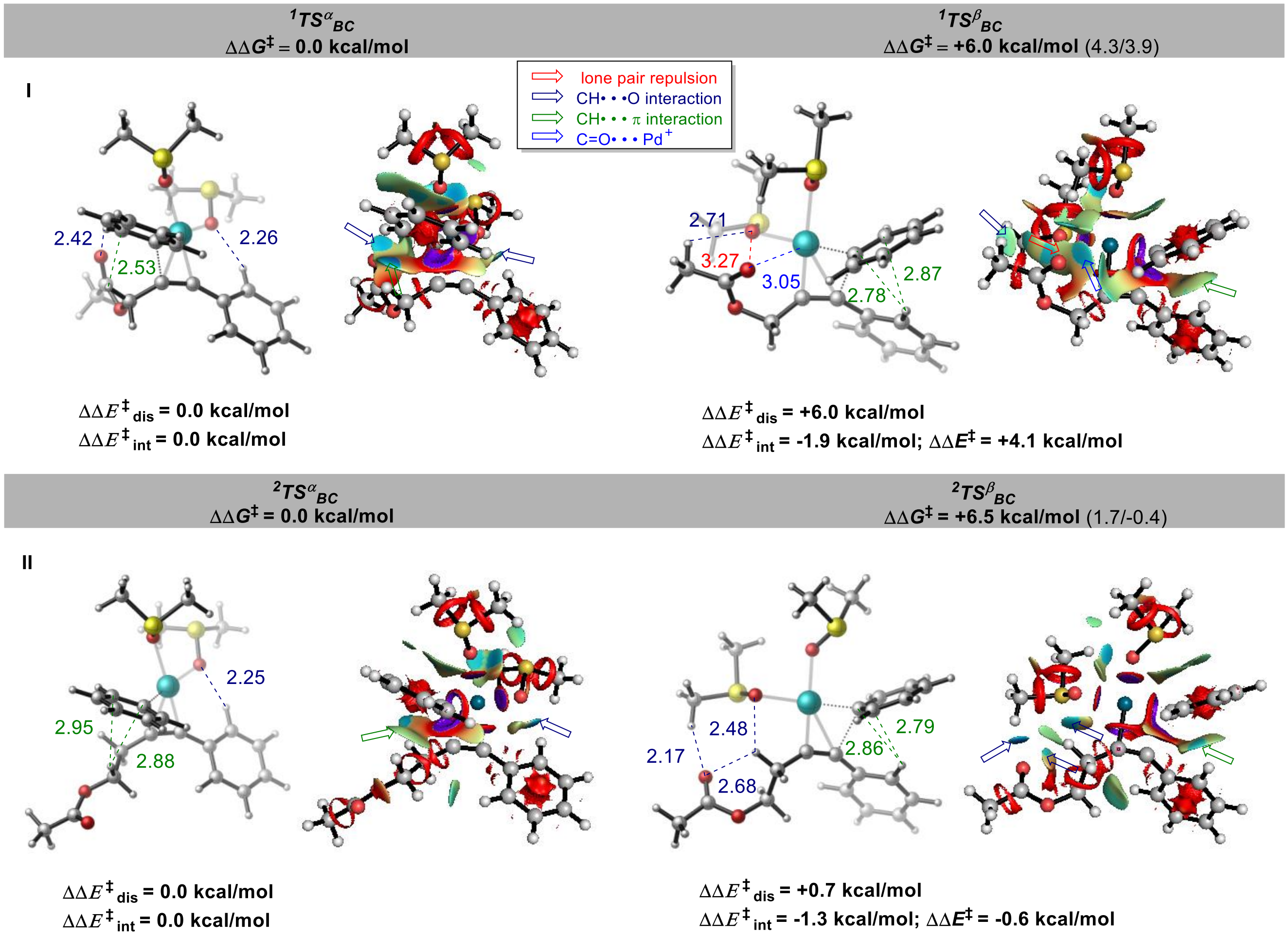

Supplementary Figure 29. Distortion/interaction and non-covalent interactions analysis ((blue, strongly attractive; green, weakly attractive; red, strongly repulsive; NCI index: -3.0 to 3.0) of α- and β-arylation transition states (n = 1 and 2). The bond lengths are in Ångström (Å). The $\Delta\Delta G^{\ddagger}/\Delta\Delta H^{\ddagger}$ without solvent correction are given in parentheses (kcal/mol). *(atom colors: blue = Pd; yellow = S; red = O; grey = C; white = H)*

As shown by the close $\Delta\Delta G^{\ddagger}/\Delta\Delta H^{\ddagger}$ values with n = 3 or 4 (Supplementary Figure 30), the above-mentioned entropy effect is now no longer at the origin of the selectivity. Larger rotation angles for the β-aryl migration TSs (III: 35.99 ° for ^{3}**TS**$^{\beta}_{BC}$, 8.78 ° for ^{3}**TS**$^{\alpha}_{BC}$; IV: 35.75 ° for ^{4}**TS**$^{\beta}_{BC}$, 8.88 ° for ^{4}**TS**$^{\alpha}_{BC}$) lead to larger distortion energies (^{3}**TS**$^{\beta}_{BC}$: +1.2 kcal/mol; ^{4}**TS**$^{\beta}_{BC}$: +0.2 kcal/mol) and larger interaction energies (^{3}**TS**$^{\beta}_{BC}$: +2.0 kcal/mol; ^{4}**TS**$^{\beta}_{BC}$: +3.8 kcal/mol). Although the NCI plots does not clearly attribute the difference in attractive interactions between the areas on the arylpalladium and substrate fragments in the TSs, it can be seen in the 3D structure that the CH···O distance of **TS**$^{\alpha}_{BC}$ (III: 2.25 Å, IV: 2.25 Å) is significantly shorter

than that of $\mathbf{TS}^{\beta}_{\mathbf{BC}}$ (III: 2.60 Å, IV: 2.68 Å). To some extent, it shows that the distance of CH···O interaction determines the attractive interactions for **c3** and **c4**. It is worth noting that the most important contributor to distortion energy is the substrate (+0.97 kcal/mol) followed by the arylpalladium (+0.23 kcal/mol) for **c3**. However, the arylpalladium is the only contributor to the distortion energy with **c4** (substrate: -0.27 kcal/mol; arylpalladium: +0.49 kcal/mol).

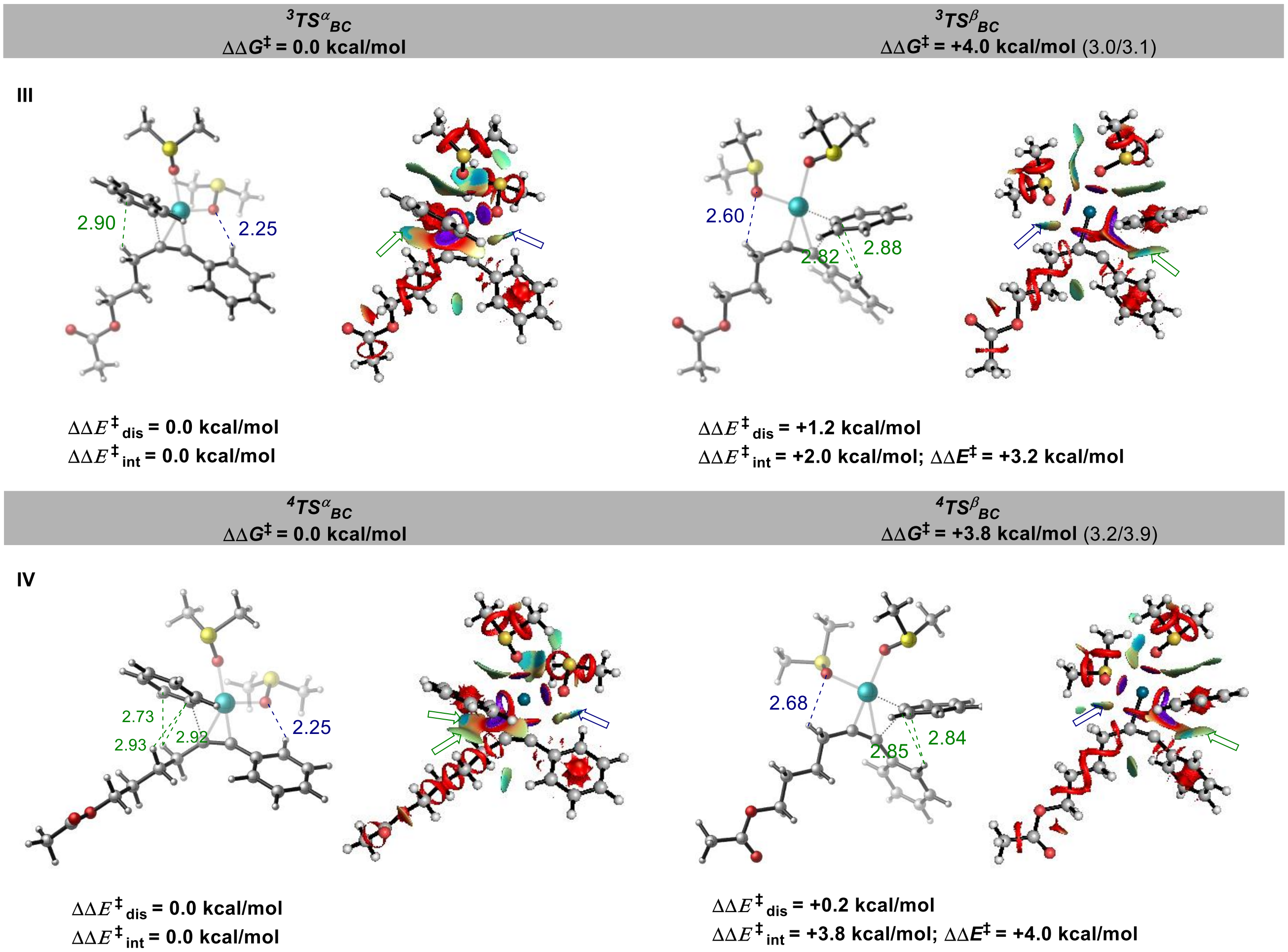

Supplementary Figure 30. Distortion/interaction and non-covalent interactions analysis ((blue, strongly attractive; green, weakly attractive; red, strongly repulsive; NCI index: -3.0 to 3.0) of α- and β-arylation transition states (n = 3 and 4). The bond lengths are in Ångström (Å). The $\Delta\Delta G^{‡}/\Delta\Delta H^{‡}$ without solvent correction are given in parentheses (kcal/mol). *(atom colors: blue = Pd; yellow = S; red = O; grey = C; white = H)*

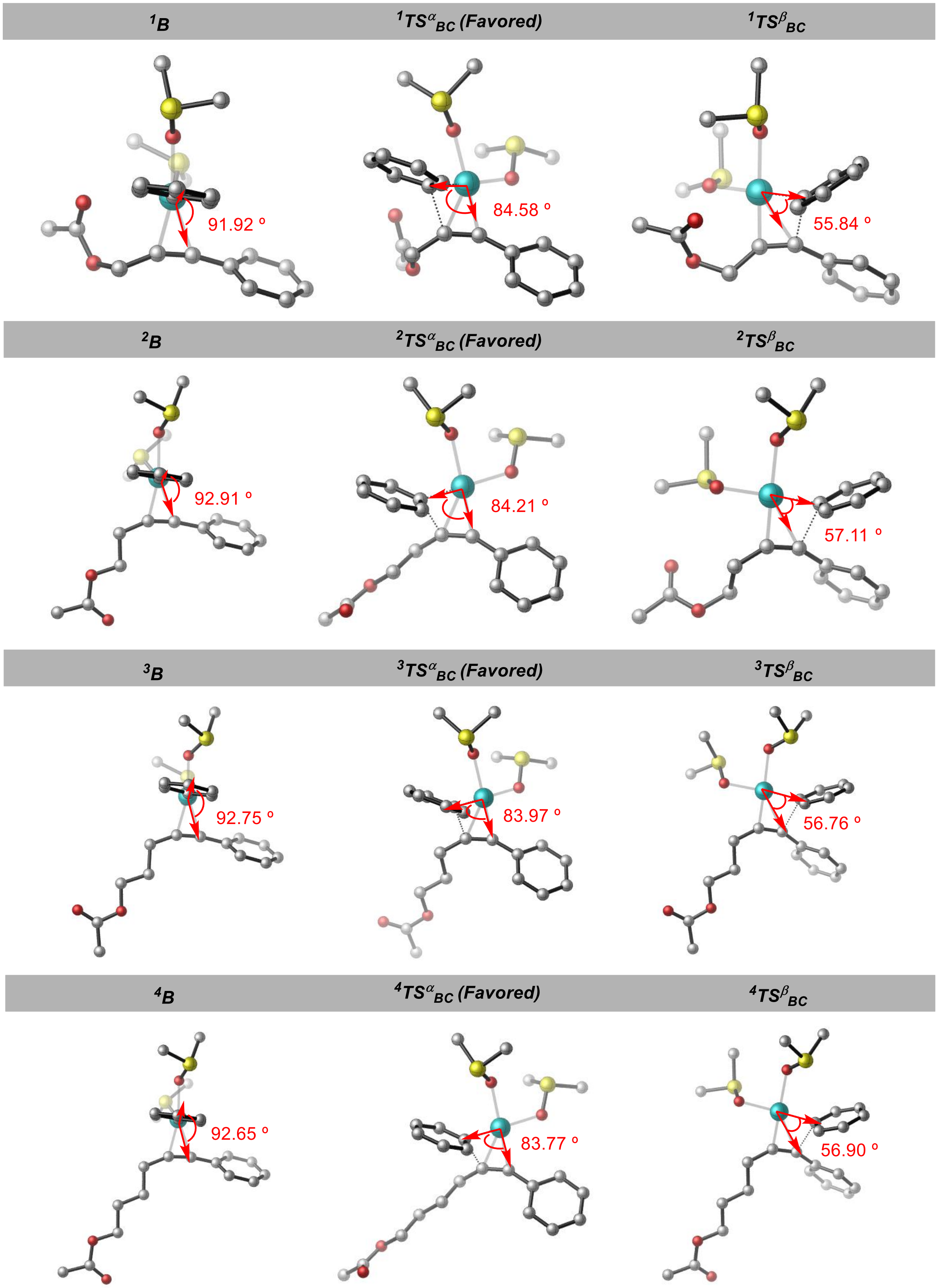

^1B
$^1TS^{\alpha}_{BC}$ (Favored)
$^1TS^{\beta}_{BC}$
91.92 º
84.58 º
55.84 º
^2B
$^2TS^{\alpha}_{BC}$ (Favored)
$^2TS^{\beta}_{BC}$
92.91 º
84.21 º
57.11 º
^3B
$^3TS^{\alpha}_{BC}$ (Favored)
$^3TS^{\beta}_{BC}$
92.75 º
83.97 º
56.76 º
^4B
$^4TS^{\alpha}_{BC}$ (Favored)
$^4TS^{\beta}_{BC}$
92.65 º
83.77 º
56.90 º

Supplementary Figure 31. Rotation angle analysis of α- and β-arylation transition states. *(atom colors: blue = Pd; yellow = S; red = O; grey = C; white = H)*

3.2.8 On the lower reactivity of alkyl-alkynes

As noted in the manuscript, substrates displaying alkyl groups at the alkyne terminus provide the desired product in low amount or only as trace. They follow different reaction pathways and a significant part of them can be recovered. To shed light on this matter, the first part of the mechanism was reinvestigated with the methyl-substituted model compound **c5** (Supplementary Figure 32). This time, chelate ^{5}**E** is more stable than ^{5}**B**, which is in sharp contrast with the previously studied **c1** case (see Supplementary Figure 21). This can be attributed to the fact that there is less steric hindrance and electronic repulsion between the Me/Ph groups in ^{5}**E** than between the Ph/Ph groups in ^{1}**E**. The same effect was observed with **c3** or **c4**, for which the greater number of carbon atoms between the OAc group and the alkyne allowed to pull the Ph/Ph groups apart. While ^{5}**B** faces a barrier of 13.8 kcal/mol to reach $^{5}\mathbf{TS}^{\alpha}_{\mathbf{BC}}$ (20.1 kcal/mol for the β transition state $^{5}\mathbf{TS}^{\alpha}_{\mathbf{BC}}$, not shown), ^{5}**E** needs 20.4 kcal/mol to reach $^{5}\mathbf{TS}^{\alpha}_{\mathbf{EF}}$. If ^{5}**E** is the dominant species in solution, this might slow down the desired process to the point that competing reactions take place. Overall, when looking at the cases for which ^{n}**B** is the most stable species (**c1**, **c2**), and those for which ^{n}**E** is the most stable one (**c3**, **c4**, **c5**), we can see that the best yields were obtained in the propargyl and homopropargyl series, that longer chains give more moderate yields, and that alkylalkynes provide only low amounts of the desired product.

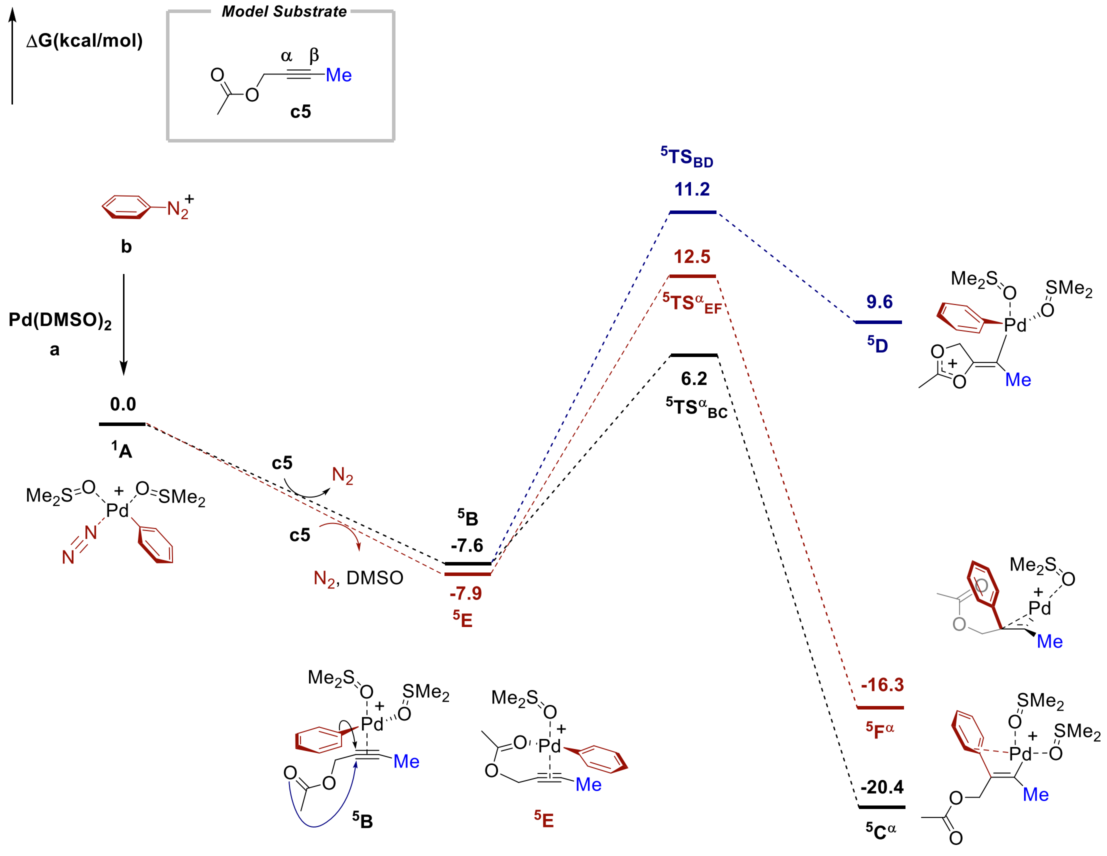

Supplementary Figure 32. Free-energy profile (ΔG_{298}, kcal/mol): *Part 1e (atom colors: blue = Pd; yellow = S; red = O; grey = C; white = H)*

Conclusion: This study on the selectivity of the title reaction leads to the following conclusions:

- Because of the barrierless oxidation addition of Pd into the C-N bond of the aryldiazonium cation, the first aryl group to react with the alkyne comes from the aryldiazonium and not the arylboronic acid.
- The stereoselectivity can be easily rationalized by the fact that the insertion of the alkyne into the Pd-Ar bond is a *syn* process. On the other hand, the regioselectivity of the carbopalladation step is not obvious and appears as an anti-Markovnikov step.
- While it is tempting to attribute the regioselectivity to a directing effect of the ester group, the calculations clearly rule out this possibility.
- The regioselectivity of the α-arylation of **1c** is mainly controlled by the fact that β-arylation requires an unfavorable distortion of the arylpalladium and substrate fragments in the corresponding transition state. In the case of **c3** and **c4**, the preference for α-arylation can be mainly explained by a greater extent of stabilizing non-covalent interactions in the transition state compared to the β-arylation process. The case of **c2** is rather intermediate between these two categories, in the sense that distortion and interaction compensate themselves but the β-arylation TS is destabilized by the entropy factor and weaker solvent interaction.

3.2.9 Energies and Cartesian coordinates for all the intermediates and transition states

a = Pd(DMSO)$_2$

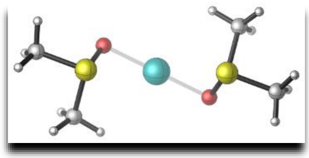

Zero-point correction= 0.155547 (Hartree/Particle)

Thermal correction to Energy= 0.170939

Thermal correction to Enthalpy= 0.171884

Thermal correction to Gibbs Free Energy= 0.110533

Sum of electronic and zero-point Energies= -1233.113497

Sum of electronic and thermal Energies= -1233.098104

Sum of electronic and thermal Enthalpies= -1233.097160

Sum of electronic and thermal Free Energies= -1233.158511

E(RM06L) = -1234.55245310

Atom X Y Z

Pd 0.000035000000 -0.000117000000 -0.537824000000

O 1.888102000000 -0.922311000000 -0.501451000000

S 2.495654000000 -0.053697000000 0.665169000000

C 4.247857000000 -0.707870000000 0.697740000000

H 4.180088000000 -1.758251000000 1.015741000000

H 4.681728000000 -0.646477000000 -0.312252000000

H 4.841589000000 -0.130245000000 1.423869000000

H 3.411260000000 1.459874000000 -1.007270000000

H 3.631785000000 2.100749000000 0.679722000000

C 2.942698000000 1.598459000000 -0.020535000000

H 1.998149000000 2.152449000000 -0.122919000000

O -1.888034000000 0.922071000000 -0.501778000000

S -2.495719000000 0.053926000000 0.665098000000

C -2.942834000000 -1.598414000000 -0.020106000000

H -1.998282000000 -2.152410000000 -0.122428000000

H -3.631822000000 -2.100529000000 0.680373000000

H -3.411507000000 -1.460117000000 -1.006829000000

C -4.247847000000 0.708235000000 0.697428000000

H -4.841640000000 0.130834000000 1.423686000000

H -4.681686000000 0.646603000000 -0.312563000000

H -4.180028000000 1.758693000000 1.015162000000

Pd(1,4-dioxane)$_2$

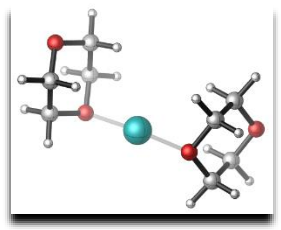

Zero-point correction= 0.239641 (Hartree/Particle)

Thermal correction to Energy= 0.253820

Thermal correction to Enthalpy= 0.254765

Thermal correction to Gibbs Free Energy= 0.196038

Sum of electronic and zero-point Energies= -741.891043

Sum of electronic and thermal Energies= -741.876864

Sum of electronic and thermal Enthalpies= -741.875920

Sum of electronic and thermal Free Energies= -741.934647

E(RM06L) = -743.489608728

Atom	X	Y	Z
Pd	-0.000163000000	-0.528305000000	0.000249000000
C	-3.000404000000	-1.142764000000	-0.028618000000
C	-3.259233000000	-0.179395000000	1.127080000000
C	-2.675082000000	1.643365000000	-0.236436000000
C	-2.414306000000	0.701210000000	-1.408762000000
H	-2.607310000000	-2.110912000000	0.317818000000
H	-3.922930000000	-1.293976000000	-0.624977000000
H	-2.320603000000	-0.086926000000	1.718946000000
H	-4.072227000000	-0.550600000000	1.774705000000
H	-3.053862000000	2.618181000000	-0.589402000000
H	-1.713914000000	1.786529000000	0.308337000000
H	-3.338551000000	0.549397000000	-2.002448000000
H	-1.598989000000	1.058806000000	-2.056395000000
C	2.999672000000	-1.142997000000	0.027593000000
C	3.258519000000	-0.179202000000	-1.127727000000
C	2.676028000000	1.643057000000	0.237068000000
C	2.415565000000	0.700340000000	1.409003000000
H	2.605967000000	-2.110756000000	-0.319230000000
H	3.922410000000	-1.294976000000	0.623445000000
H	2.319926000000	-0.086067000000	-1.719512000000
H	4.071193000000	-0.550733000000	-1.775606000000
H	3.055498000000	2.617480000000	0.590380000000
H	1.714577000000	1.787065000000	-0.306971000000
H	3.340152000000	0.547519000000	2.001905000000
H	1.601081000000	1.058180000000	2.057549000000
O	-1.978883000000	-0.597093000000	-0.912407000000
O	1.978892000000	-0.597310000000	0.912206000000
O	-3.674180000000	1.103680000000	0.638422000000
O	3.674239000000	1.103491000000	-0.638873000000

Pd(DMSO)(1,4-dioxane)

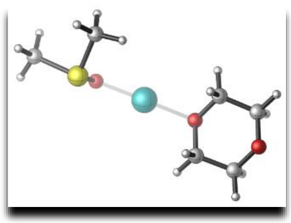

Zero-point correction= 0.197842 (Hartree/Particle)

Thermal correction to Energy= 0.212673

Thermal correction to Enthalpy= 0.213617

Thermal correction to Gibbs Free Energy= 0.153411

Sum of electronic and zero-point Energies= -987.499339

Sum of electronic and thermal Energies= -987.484508

Sum of electronic and thermal Enthalpies= -987.483563

Sum of electronic and thermal Free Energies= -987.543770

E(RM06L) = -989.016976473

Atom	X	Y	Z
Pd	-0.344856000000	0.384737000000	0.443514000000
C	2.400836000000	0.874713000000	-0.882073000000
C	3.912443000000	0.902525000000	-0.660044000000
C	3.820395000000	-1.141282000000	0.476592000000

C 2.305919000000 -1.208785000000 0.280487000000

H 1.955310000000 1.880454000000 -0.856334000000

H 2.154350000000 0.386859000000 -1.846037000000

H 4.140177000000 1.458011000000 0.276016000000

H 4.415012000000 1.407240000000 -1.502787000000

H 4.253044000000 -2.156444000000 0.478355000000

H 4.046584000000 -0.655806000000 1.451328000000

H 1.797367000000 -1.681613000000 1.134207000000

H 2.053428000000 -1.755584000000 -0.649983000000

O 1.767788000000 0.137958000000 0.199659000000

O 4.447266000000 -0.425261000000 -0.593770000000

O -2.387794000000 0.680871000000 0.728068000000

S -2.857505000000 0.000494000000 -0.615837000000

C -2.785759000000 -1.829727000000 -0.390215000000

C -4.717658000000 0.117288000000 -0.414091000000

H -3.401927000000 -2.311051000000 -1.168954000000

H -1.725354000000 -2.105434000000 -0.483056000000

H -3.155552000000 -2.078631000000 0.616609000000

H -5.210899000000 -0.398919000000 -1.253005000000

H -4.962953000000 1.188904000000 -0.432944000000

H -5.010288000000 -0.320746000000 0.552754000000

DMSO

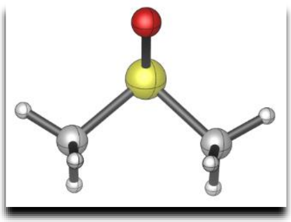

Zero-point correction= 0.077089 (Hartree/Particle)

Thermal correction to Energy= 0.082916

Thermal correction to Enthalpy= 0.083860

Thermal correction to Gibbs Free Energy= 0.048584

Sum of electronic and zero-point Energies= -553.139569

Sum of electronic and thermal Energies= -553.133741

Sum of electronic and thermal Enthalpies= -553.132797

Sum of electronic and thermal Free Energies= -553.168073

E(RM06L) = -553.269669226

Atom X Y Z

C -0.258575000000 -0.815303000000 1.366806000000

S -0.258575000000 0.437279000000 0.000000000000

O 1.097600000000 1.129384000000 0.000000000000

C -0.258575000000 -0.815303000000 -1.366806000000

H -0.213247000000 -0.254224000000 2.311984000000

H -1.186355000000 -1.408801000000 1.327268000000

H 0.629253000000 -1.460928000000 1.274877000000

H -1.186355000000 -1.408801000000 -1.327268000000

H -0.213247000000 -0.254224000000 -2.311984000000

H 0.629253000000 -1.460928000000 -1.274877000000

1,4-dioxane

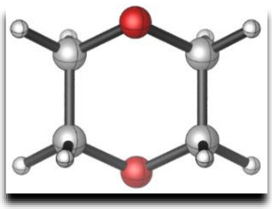

Zero-point correction= 0.118785 (Hartree/Particle)

Thermal correction to Energy= 0.124102

Thermal correction to Enthalpy= 0.125046

Thermal correction to Gibbs Free Energy= 0.090321

Sum of electronic and zero-point Energies= -307.538934

Sum of electronic and thermal Energies= -307.533617

Sum of electronic and thermal Enthalpies= -307.532673

Sum of electronic and thermal Free Energies= -307.567398

E(RM06L) = -307.740827658

Atom X Y Z

C 1.172917000000 0.739140000000 -0.199118000000

C 1.173044000000 -0.738958000000 0.199110000000

C -1.172931000000 -0.739124000000 0.199105000000

C -1.173033000000 0.738965000000 -0.199115000000

H 2.038705000000 1.269521000000 0.234002000000

H 1.220071000000 0.821654000000 -1.307813000000

H 1.220221000000 -0.821350000000 1.307808000000

H 2.038909000000 -1.269264000000 -0.233984000000

H -2.038718000000 -1.269546000000 -0.234011000000

H -1.220121000000 -0.821545000000 1.307802000000

H -1.220184000000 0.821479000000 -1.307807000000

H -2.038880000000 1.269239000000 0.234017000000

O -0.000104000000 1.396851000000 0.297143000000

O 0.000106000000 -1.396892000000 -0.297131000000

N = 1

1c = 3-Phenyl-2-propynylacetate

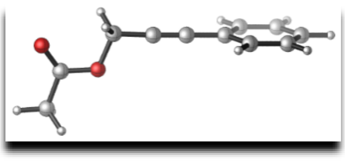

Zero-point correction= 0.175296 (Hartree/Particle)

Thermal correction to Energy= 0.188311

Thermal correction to Enthalpy= 0.189255

Thermal correction to Gibbs Free Energy= 0.131252

Sum of electronic and zero-point Energies= -575.408245

Sum of electronic and thermal Energies= -575.395230

Sum of electronic and thermal Enthalpies= -575.394286

Sum of electronic and thermal Free Energies= -575.452289

E(RM06L) = -575.751419150

Atom X Y Z

H 5.896859000000 0.325326000000 1.274842000000

O 2.747785000000 0.113564000000 0.301491000000

H 4.436390000000 -0.038621000000 2.270567000000

H -2.235842000000 -2.123980000000 0.464170000000

H -4.695465000000 -1.979134000000 0.884470000000

H -5.903797000000 0.182089000000 0.521265000000

C 4.103176000000 0.035947000000 0.133256000000

C 4.818597000000 0.497861000000 1.387569000000

H 4.631700000000 1.572311000000 1.551451000000

H 2.274903000000 0.337389000000 -1.732668000000

C -2.046870000000 -0.046681000000 -0.139389000000

H -4.644413000000 2.197116000000 -0.263843000000

H -2.185085000000 2.050354000000 -0.685623000000

O 4.635734000000 -0.347738000000 -0.898780000000

C -2.770187000000 -1.182419000000 0.306456000000

C -4.147847000000 -1.095518000000 0.540537000000

C -4.826465000000 0.118192000000 0.336783000000

C -4.119142000000 1.249725000000 -0.104188000000

C -2.741502000000 1.173225000000 -0.342141000000

C 0.565104000000 -0.201956000000 -0.585632000000

C -0.641089000000 -0.130557000000 -0.381303000000

C 1.985977000000 -0.303347000000 -0.879108000000

H 2.278086000000 -1.336278000000 -1.143352000000

b = PhN_2^+

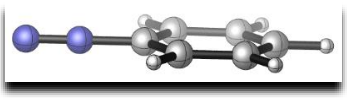

Zero-point correction= 0.096252 (Hartree/Particle)

Thermal correction to Energy= 0.102586

Thermal correction to Enthalpy= 0.103531

Thermal correction to Gibbs Free Energy= 0.065813

Sum of electronic and zero-point Energies= -340.752415

Sum of electronic and thermal Energies= -340.746081

Sum of electronic and thermal Enthalpies= -340.745137

Sum of electronic and thermal Free Energies= -340.782854

E(RM06L) = -341.015861292

**Atom**	**X**	**Y**	**Z**
C	-1.490195000000	1.230329000000	0.000298000000
C	-0.096435000000	1.255648000000	0.000168000000
C	0.571101000000	0.000019000000	-0.000054000000
C	-0.096425000000	-1.255607000000	-0.000150000000
C	-1.490217000000	-1.230345000000	-0.000013000000
C	-2.181193000000	-0.000016000000	0.000208000000
N	1.942877000000	-0.000016000000	-0.000185000000
N	3.074490000000	-0.000005000000	-0.000293000000
H	-2.042995000000	2.173640000000	0.000471000000
H	0.469908000000	2.190394000000	0.000233000000
H	0.469958000000	-2.190331000000	-0.000323000000
H	-2.042891000000	-2.173730000000	-0.000080000000
H	-3.275369000000	0.000012000000	0.000313000000

d = $PhB(OH)_3^-$

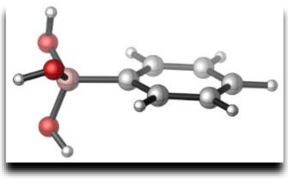

Zero-point correction= 0.132257 (Hartree/Particle)

Thermal correction to Energy= 0.142546

Thermal correction to Enthalpy= 0.143491

Thermal correction to Gibbs Free Energy= 0.096587

Sum of electronic and zero-point Energies= -483.987102

Sum of electronic and thermal Energies= -483.976812

Sum of electronic and thermal Enthalpies= -483.975868

Sum of electronic and thermal Free Energies= -484.022772

E(RM06L) = -484.344958822

**Atom**	**X**	**Y**	**Z**
C	-0.066712000000	-0.004323000000	-0.039415000000
C	-0.823004000000	-1.201908000000	-0.054592000000
C	-0.814123000000	1.198419000000	-0.010757000000
C	-2.230101000000	-1.208326000000	-0.027556000000
C	-2.219085000000	1.215191000000	0.019112000000
C	-2.938925000000	0.005508000000	0.014196000000
H	-0.273846000000	-2.151811000000	-0.108121000000
H	-0.247531000000	2.139202000000	-0.029799000000
H	-2.781025000000	-2.160306000000	-0.045825000000
H	-2.762404000000	2.171320000000	0.041374000000
B	1.593518000000	0.019903000000	0.008466000000
O	2.138308000000	1.269382000000	-0.588984000000
O	2.071885000000	0.025801000000	1.420771000000
O	2.046928000000	-1.204026000000	-0.732317000000
H	2.977742000000	-1.282589000000	-0.449134000000
H	2.088667000000	1.097182000000	-1.545746000000
H	1.562681000000	-0.678951000000	1.858596000000
H	-4.037157000000	0.009805000000	0.034631000000

N_2

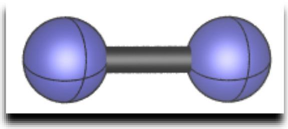

Zero-point correction= 0.005352 (Hartree/Particle)

Thermal correction to Energy= 0.007713

Thermal correction to Enthalpy= 0.008657

Thermal correction to Gibbs Free Energy= -0.013118

Sum of electronic and zero-point Energies= -109.517860

Sum of electronic and thermal Energies= -109.515500

Sum of electronic and thermal Enthalpies= -109.514555

Sum of electronic and thermal Free Energies=   -109.536330

E(RM06L) = -109.552809285

Atom   X   Y   Z

N   0.000000000000   0.000000000000   0.558736000000

N   0.000000000000   0.000000000000 -0.558736000000

$B(OH)_3$

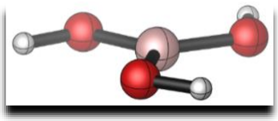

Zero-point correction=   0.047717 (Hartree/Particle)

Thermal correction to Energy=   0.052175

Thermal correction to Enthalpy=   0.053119

Thermal correction to Gibbs Free Energy=   0.021225

Sum of electronic and zero-point Energies=   -252.431550

Sum of electronic and thermal Energies=   -252.427092

Sum of electronic and thermal Enthalpies=   -252.426148

Sum of electronic and thermal Free Energies=   -252.458042

E(RM06L) = -252.573501560

Atom   X   Y   Z

B   0.000037000000   0.000077000000 -0.000025000000

O -0.824073000000   1.109233000000 -0.000121000000

O   1.372671000000   0.158842000000   0.000058000000

O -0.548711000000   -1.267976000000 -0.000058000000

H   0.154806000000   -1.940582000000   0.000178000000

H -1.757976000000   0.835193000000   0.000950000000

H   1.603896000000   1.104220000000 -0.000032000000

^{1}A

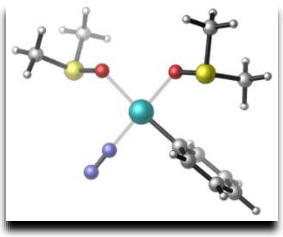

Zero-point correction=   0.253144 (Hartree/Particle)

Thermal correction to Energy=   0.276667

Thermal correction to Enthalpy=   0.277611

Thermal correction to Gibbs Free Energy=   0.195322

Sum of electronic and zero-point Energies=   -1574.008547

Sum of electronic and thermal Energies=   -1573.985024

Sum of electronic and thermal Enthalpies=   -1573.984080

Sum of electronic and thermal Free Energies=   -1574.066369

E(RM06L) = -1575.68931200

Atom   X   Y   Z

Pd 0.277410000000   -0.411336000000 -0.120324000000

C -1.653264000000   -0.939208000000   0.045514000000

C -2.460197000000   -1.006521000000 -1.100677000000

C -2.169020000000   -1.215750000000   1.321884000000

C -3.823830000000   -1.336564000000 -0.957161000000

C -3.533511000000   -1.544525000000   1.449398000000

C -4.358106000000   -1.602038000000   0.312939000000

H -2.048827000000   -0.812432000000 -2.097022000000

H -1.530899000000   -1.178994000000   2.211018000000

H -4.458727000000   -1.394272000000 -1.847392000000

H -3.942849000000   -1.762019000000   2.441507000000

C   4.199587000000   0.911540000000   1.560896000000

S   3.580737000000   -0.319200000000   0.356812000000

O   2.351980000000   0.365239000000 -0.336536000000

C   4.922973000000   -0.188526000000 -0.876727000000

H   3.438252000000   0.997670000000   2.349647000000

H   5.144453000000   0.544168000000   1.992461000000

H	4.340726000000	1.879338000000	1.055930000000
H	5.864691000000	-0.525784000000	-0.415301000000
H	4.657713000000	-0.851350000000	-1.712967000000
H	4.998028000000	0.853632000000	-1.222852000000
C	-2.714703000000	2.447133000000	0.832418000000
S	-1.483085000000	2.216509000000	-0.495601000000
O	-0.212649000000	1.570093000000	0.214373000000
C	-0.922055000000	3.940894000000	-0.681333000000
H	-2.236895000000	2.931045000000	1.697719000000
H	-3.539422000000	3.061115000000	0.435825000000
H	-3.081898000000	1.444766000000	1.097810000000
H	-1.768946000000	4.547613000000	-1.039150000000
H	-0.547392000000	4.309355000000	0.285432000000
H	-0.118332000000	3.937755000000	-1.431437000000
N	0.725247000000	-2.303794000000	-0.406079000000
N	0.971900000000	-3.386501000000	-0.583722000000
H	-5.414890000000	-1.866296000000	0.417313000000

^{1}B

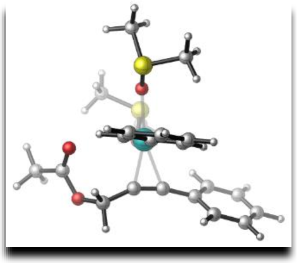

Zero-point correction= 0.422095 (Hartree/Particle)

Thermal correction to Energy= 0.457229

Thermal correction to Enthalpy= 0.458173

Thermal correction to Gibbs Free Energy= 0.348259

Sum of electronic and zero-point Energies= -2039.930630

Sum of electronic and thermal Energies= -2039.895497

Sum of electronic and thermal Enthalpies= -2039.894553

Sum of electronic and thermal Free Energies= -2040.004467

E(RM06L) = -2041.90724838

**Atom**	**X**	**Y**	**Z**
H	2.539202000000	3.144651000000	3.343734000000
O	0.643105000000	1.435726000000	3.461334000000
H	3.877316000000	1.954814000000	3.076812000000
H	-2.017219000000	2.520084000000	-1.105636000000
H	-4.115643000000	3.020835000000	-2.399575000000
H	-6.221493000000	1.777544000000	-1.883394000000
C	1.912816000000	1.090531000000	3.080050000000
C	2.904486000000	2.127781000000	3.555107000000
H	3.023359000000	2.045495000000	4.648899000000
H	-0.006589000000	-0.556612000000	3.369003000000
C	-2.961143000000	1.009486000000	0.126738000000
H	-6.244820000000	0.042024000000	-0.083856000000
H	-4.158449000000	-0.456724000000	1.201731000000
O	2.183119000000	0.066500000000	2.463101000000
C	-2.950661000000	1.989341000000	-0.898420000000
C	-4.122010000000	2.259687000000	-1.613217000000
C	-5.307550000000	1.560380000000	-1.321950000000
C	-5.321855000000	0.584258000000	-0.310899000000
C	-4.157105000000	0.300900000000	0.412826000000
C	-0.907693000000	0.535355000000	1.788472000000
C	-1.778777000000	0.739506000000	0.892272000000
C	-0.390994000000	0.459038000000	3.173026000000
H	-1.204429000000	0.696872000000	3.877454000000
Pd	0.104585000000	-0.042859000000	0.045015000000
C	-0.400504000000	-1.919836000000	0.481256000000
C	0.412950000000	-2.662492000000	1.356397000000
C	-1.526535000000	-2.506220000000	-0.125767000000
C	0.111377000000	-4.018353000000	1.593819000000
C	-1.813681000000	-3.864754000000	0.116838000000
C	-0.994772000000	-4.620053000000	0.972302000000
H	1.269170000000	-2.189105000000	1.849865000000

H -2.188186000000 -1.916335000000 -0.770343000000

H 0.744900000000 -4.599009000000 2.273110000000

H -2.688143000000 -4.324170000000 -0.356966000000

C 3.255167000000 2.373831000000 -1.099661000000

S 1.519266000000 2.440767000000 -1.684174000000

O 0.630354000000 2.116145000000 -0.438230000000

C 1.377731000000 4.258834000000 -1.839452000000

H 3.444681000000 1.322700000000 -0.840906000000

H 3.922721000000 2.695477000000 -1.915198000000

H 3.373295000000 3.016528000000 -0.213987000000

H 2.087256000000 4.610731000000 -2.605136000000

H 0.348268000000 4.479637000000 -2.155973000000

H 1.582541000000 4.722205000000 -0.862305000000

C 0.819929000000 -2.676866000000 -2.993775000000

S 1.964686000000 -2.115300000000 -1.682895000000

O 1.592599000000 -0.600579000000 -1.408897000000

C 3.474240000000 -1.950087000000 -2.697112000000

H 0.779860000000 -1.922465000000 -3.794279000000

H 1.172593000000 -3.648673000000 -3.374978000000

H -0.162516000000 -2.797592000000 -2.514439000000

H 3.762264000000 -2.948778000000 -3.061780000000

H 3.283671000000 -1.262270000000 -3.534808000000

H 4.263488000000 -1.555553000000 -2.041127000000

H -1.227172000000 -5.672179000000 1.164940000000

$^1TS^{\alpha}_{BC}$

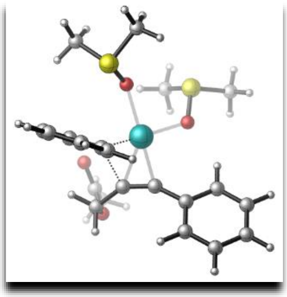

Frequency -242.3426

Zero-point correction= 0.421998 (Hartree/Particle)

Thermal correction to Energy= 0.456123

Thermal correction to Enthalpy= 0.457067

Thermal correction to Gibbs Free Energy= 0.350933

Sum of electronic and zero-point Energies= -2039.916556

Sum of electronic and thermal Energies= -2039.882432

Sum of electronic and thermal Enthalpies= -2039.881487

Sum of electronic and thermal Free Energies= -2039.987621

E(RM06L) = -2041.88918117

Atom	X	Y	Z
C	-1.049843000000	-1.564305000000	0.534244000000
C	0.722068000000	-1.799653000000	-0.507432000000
C	-1.803496000000	-0.726751000000	-0.115186000000
C	-3.031498000000	-0.377995000000	-0.770158000000
C	-3.390740000000	0.971720000000	-1.022143000000
H	-2.702552000000	1.765591000000	-0.718541000000
C	-3.914211000000	-1.421946000000	-1.167076000000
H	-3.631090000000	-2.463357000000	-0.986408000000
C	-4.609093000000	1.263275000000	-1.644152000000
H	-4.885552000000	2.305154000000	-1.834075000000
C	-5.480357000000	0.227062000000	-2.025520000000
H	-6.431761000000	0.462964000000	-2.512341000000
C	-5.129679000000	-1.113791000000	-1.784743000000
H	-5.804986000000	-1.921025000000	-2.084630000000
C	-1.052725000000	-2.484057000000	1.711725000000
O	-1.466076000000	-1.828405000000	2.940514000000
C	-0.486506000000	-1.109918000000	3.577336000000
C	-1.014394000000	-0.527269000000	4.868177000000
H	-1.335975000000	-1.337466000000	5.543051000000
H	-1.899716000000	0.098690000000	4.670473000000
O	0.650951000000	-0.982463000000	3.140436000000
H	-0.228586000000	0.067196000000	5.351457000000

H -0.070172000000 -2.956409000000 1.853747000000

Pd 0.101648000000 0.109826000000 -0.158606000000

H -1.818196000000 -3.259646000000 1.544023000000

O -0.585092000000 2.235810000000 -0.098065000000

O 2.135203000000 1.008294000000 -0.159978000000

C 1.790764000000 -2.281346000000 0.283600000000

C 0.490804000000 -2.328526000000 -1.796520000000

C 2.640571000000 -3.272506000000 -0.237666000000

H 1.929154000000 -1.890924000000 1.297720000000

C 1.360811000000 -3.305419000000 -2.308829000000

H -0.365498000000 -1.988459000000 -2.387124000000

C 2.434003000000 -3.778215000000 -1.533511000000

H 3.462169000000 -3.656646000000 0.376939000000

H 1.185690000000 -3.710763000000 -3.310834000000

H 3.095836000000 -4.554116000000 -1.930793000000

S 0.435579000000 3.418674000000 0.043691000000

C 1.040629000000 3.378531000000 1.771745000000

H 1.667279000000 2.478868000000 1.852216000000

H 1.641761000000 4.282859000000 1.959745000000

H 0.183708000000 3.321029000000 2.460142000000

C -0.666464000000 4.871520000000 0.189555000000

H -0.057396000000 5.762681000000 0.409480000000

H -1.170096000000 4.993798000000 -0.780178000000

H -1.405386000000 4.688260000000 0.984254000000

S 3.207604000000 0.407266000000 -1.142697000000

C 3.659386000000 1.800101000000 -2.242117000000

H 3.936390000000 2.680420000000 -1.642633000000

H 4.492964000000 1.482448000000 -2.888721000000

H 2.774912000000 2.017154000000 -2.858629000000

C 4.740854000000 0.349726000000 -0.148709000000

H 5.583421000000 0.088610000000 -0.808643000000

H 4.902823000000 1.325044000000 0.335026000000

H 4.598917000000 -0.436289000000 0.606258000000

$^{1}C^{\alpha}$

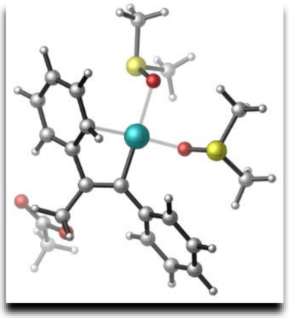

Zero-point correction= 0.424524 (Hartree/Particle)

Thermal correction to Energy= 0.458494

Thermal correction to Enthalpy= 0.459438

Thermal correction to Gibbs Free Energy= 0.353002

Sum of electronic and zero-point Energies= -2039.960589

Sum of electronic and thermal Energies= -2039.926619

Sum of electronic and thermal Enthalpies= -2039.925675

Sum of electronic and thermal Free Energies= -2040.032111

E(RM06L) = -2041.93172062

Atom X Y Z

C -1.696484000000 -1.109336000000 -0.804201000000

C -0.557072000000 -2.055291000000 -0.980501000000

C -1.244565000000 0.172423000000 -0.701013000000

C -1.943456000000 1.450547000000 -0.559446000000

C -2.875292000000 1.681744000000 0.484247000000

H -3.080135000000 0.881348000000 1.199359000000

C -1.692115000000 2.491103000000 -1.492801000000

H -0.983413000000 2.312975000000 -2.308536000000

C -3.541071000000 2.911662000000 0.579484000000

H -4.265172000000 3.073823000000 1.384385000000

C -3.292686000000 3.930246000000 -0.357544000000

H -3.825006000000 4.884059000000 -0.285512000000

C -2.369363000000 3.714535000000 -1.397457000000

H -2.187675000000 4.496528000000 -2.141955000000

C -3.145216000000 -1.550248000000 -0.808324000000

O -3.792488000000 -1.418096000000 0.500226000000

C -3.841195000000 -2.544917000000 1.280045000000

C -4.664456000000 -2.300848000000 2.525358000000

H -5.731708000000 -2.443057000000 2.282359000000

H -4.541366000000 -1.273697000000 2.899236000000

O -3.314548000000 -3.607962000000 0.977776000000

H -4.384202000000 -3.031494000000 3.295936000000

H -3.232916000000 -2.596960000000 -1.140484000000

Pd 0.736585000000 0.055388000000 -0.478067000000

H -3.738670000000 -0.892966000000 -1.463096000000

O 0.881281000000 1.797956000000 0.785809000000

O 2.960586000000 -0.137486000000 -0.141823000000

C -0.429015000000 -3.302285000000 -0.310422000000

C 0.489243000000 -1.645913000000 -1.895396000000

C 0.704424000000 -4.088329000000 -0.516240000000

H -1.238110000000 -3.638203000000 0.346211000000

C 1.645812000000 -2.467109000000 -2.065062000000

H 0.240573000000 -0.924840000000 -2.687106000000

C 1.757083000000 -3.666894000000 -1.371311000000

H 0.782536000000 -5.055087000000 -0.007430000000

H 2.415357000000 -2.158480000000 -2.779330000000

H 2.626108000000 -4.315216000000 -1.522202000000

S 1.273487000000 3.098172000000 -0.020017000000

C 3.058137000000 3.330820000000 0.315807000000

H 3.548782000000 2.426942000000 -0.076449000000

H 3.409481000000 4.226976000000 -0.220276000000

H 3.227774000000 3.424916000000 1.399285000000

C 0.599878000000 4.441820000000 1.014391000000

H 0.988740000000 5.403316000000 0.642964000000

H -0.493882000000 4.406811000000 0.903718000000

H 0.894368000000 4.270097000000 2.061063000000

S 3.252776000000 -1.166900000000 1.004236000000

C 4.986863000000 -1.674941000000 0.719244000000

H 5.612451000000 -0.777959000000 0.593397000000

H 5.326873000000 -2.280862000000 1.574119000000

H 4.998342000000 -2.280355000000 -0.198066000000

C 3.517069000000 -0.171428000000 2.521835000000

H 3.741023000000 -0.850972000000 3.359649000000

H 4.337595000000 0.545622000000 2.365971000000

H 2.571324000000 0.359089000000 2.706306000000

$^{1}TS^{\beta}_{BC}$

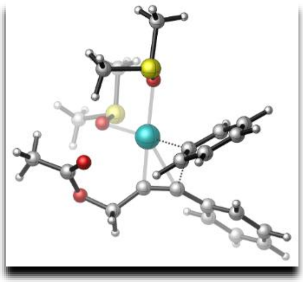

Frequency -276.6824

Zero-point correction= 0.421705 (Hartree/Particle)

Thermal correction to Energy= 0.455759

Thermal correction to Enthalpy= 0.456703

Thermal correction to Gibbs Free Energy= 0.351281

Sum of electronic and zero-point Energies= -2039.909628

Sum of electronic and thermal Energies= -2039.875573

Sum of electronic and thermal Enthalpies= -2039.874629

Sum of electronic and thermal Free Energies= -2039.980052

E(RM06L) = -2041.87992736

Atom X Y Z

C 1.779965000000 -0.828186000000 0.399783000000

C 1.302363000000 1.178834000000 0.271968000000

C 3.100877000000 -1.084138000000 -0.142903000000

C 3.319091000000 -2.298283000000 -0.845065000000

H 2.473240000000 -2.968438000000 -1.026675000000

C 4.187715000000 -0.203081000000 0.078013000000

H 4.021438000000 0.731130000000 0.620920000000

C 4.601362000000 -2.625561000000 -1.300959000000

H 4.762434000000 -3.562710000000 -1.842619000000

C 5.675879000000 -1.749785000000 -1.068642000000

H 6.677028000000 -2.006098000000 -1.429051000000

C 5.466350000000 -0.543033000000 -0.377432000000

H 6.305308000000 0.134726000000 -0.192540000000

C 0.717385000000 -1.350327000000 0.942940000000

C 0.327195000000 -2.208896000000 2.100944000000

O -1.067464000000 -2.572520000000 2.172332000000

C -1.917486000000 -1.558732000000 2.524332000000

C -3.339557000000 -2.050132000000 2.637551000000

H -3.382302000000 -3.125704000000 2.859080000000

H -3.836542000000 -1.866754000000 1.670515000000

O -1.544531000000 -0.402022000000 2.703821000000

H -3.863292000000 -1.474841000000 3.414106000000

H 0.606634000000 -1.682509000000 3.031882000000

Pd -0.345707000000 0.009795000000 -0.069092000000

H 0.866722000000 -3.169809000000 2.055189000000

O -2.191414000000 -1.241448000000 -0.386867000000

O -1.480224000000 1.555801000000 -1.198483000000

C 1.428306000000 1.732666000000 1.567105000000

C 1.831925000000 1.860085000000 -0.849168000000

C 2.045338000000 2.984785000000 1.730337000000

H 1.045746000000 1.185130000000 2.433836000000

C 2.449566000000 3.107051000000 -0.671296000000

H 1.760820000000 1.417051000000 -1.847086000000

C 2.556675000000 3.670334000000 0.614738000000

H 2.138181000000 3.415929000000 2.732523000000

H 2.855808000000 3.637717000000 -1.538867000000

H 3.051317000000 4.637778000000 0.747077000000

S -2.153575000000 -1.854069000000 -1.829861000000

C -3.171573000000 -0.744979000000 -2.874017000000

H -2.680772000000 0.238682000000 -2.818787000000

H -3.170223000000 -1.128523000000 -3.906950000000

H -4.195316000000 -0.685532000000 -2.473148000000

C -3.306599000000 -3.272001000000 -1.739975000000

H -3.442923000000 -3.681657000000 -2.753507000000

H -2.843013000000 -4.026988000000 -1.088973000000

H -4.266062000000 -2.935875000000 -1.317809000000

S -1.675187000000 2.827550000000 -0.291684000000

C -2.584308000000 3.980105000000 -1.380871000000

H -3.477627000000 3.479531000000 -1.784010000000

H -2.860265000000 4.873389000000 -0.798510000000

H -1.900172000000 4.261454000000 -2.194336000000

C -3.033145000000 2.424594000000 0.872013000000

H -3.263689000000 3.324762000000 1.464794000000

H -3.915893000000 2.080837000000 0.311468000000

H -2.658760000000 1.624640000000 1.531630000000

$^{1}C^{\beta}$

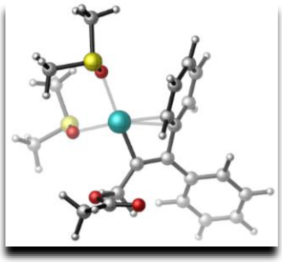

Zero-point correction= 0.424269 (Hartree/Particle)

Thermal correction to Energy= 0.458293

Thermal correction to Enthalpy= 0.459238

Thermal correction to Gibbs Free Energy= 0.352787

Sum of electronic and zero-point Energies= -2039.954097

Sum of electronic and thermal Energies= -2039.920073

Sum of electronic and thermal Enthalpies= -2039.919128

Sum of electronic and thermal Free Energies= -2040.025579

E(RM06L) = -2041.93278602

**Atom**	**X**	**Y**	**Z**
C	2.023262000000	0.320303000000	-0.143730000000
C	1.188391000000	1.570731000000	-0.100510000000
C	3.475218000000	0.347603000000	-0.399257000000
C	4.132154000000	-0.726347000000	-1.046653000000
H	3.546909000000	-1.574784000000	-1.416039000000
C	4.242868000000	1.467209000000	0.004080000000
H	3.752420000000	2.304524000000	0.511737000000
C	5.516355000000	-0.691940000000	-1.260702000000
H	6.008488000000	-1.528399000000	-1.766992000000
C	6.267829000000	0.419441000000	-0.843868000000
H	7.348444000000	0.446665000000	-1.014645000000
C	5.625876000000	1.499819000000	-0.215227000000
H	6.206374000000	2.368223000000	0.111742000000
C	1.194326000000	-0.724085000000	0.118458000000
C	1.453522000000	-2.177290000000	0.347337000000
O	0.642768000000	-2.760892000000	1.404458000000
C	0.950963000000	-2.328179000000	2.673739000000
C	0.140457000000	-3.085156000000	3.704221000000
H	0.599721000000	-4.074698000000	3.871060000000
H	-0.890992000000	-3.253218000000	3.359208000000
O	1.788456000000	-1.470458000000	2.907910000000
H	0.149130000000	-2.531727000000	4.652718000000
H	2.524921000000	-2.297943000000	0.598584000000
Pd	-0.624430000000	0.065622000000	0.008098000000
H	1.214292000000	-2.795005000000	-0.533754000000
O	-1.575273000000	-1.750332000000	-0.636873000000
O	-2.637531000000	1.037060000000	-0.364268000000
C	0.395064000000	1.795135000000	1.086689000000
C	1.178742000000	2.556247000000	-1.133495000000
C	-0.373305000000	2.993424000000	1.201775000000
H	0.613822000000	1.199392000000	1.981492000000
C	0.421225000000	3.713345000000	-0.987900000000
H	1.792788000000	2.395854000000	-2.024879000000
C	-0.362235000000	3.933103000000	0.177918000000
H	-0.928205000000	3.182652000000	2.126353000000
H	0.434875000000	4.472370000000	-1.776864000000
H	-0.926520000000	4.865607000000	0.279017000000
S	-1.564155000000	-1.878955000000	-2.212739000000
C	-3.127251000000	-1.124664000000	-2.795835000000
H	-3.107999000000	-0.084911000000	-2.434092000000
H	-3.142854000000	-1.160224000000	-3.896996000000
H	-3.986894000000	-1.663562000000	-2.369039000000
C	-1.990664000000	-3.637192000000	-2.467711000000
H	-2.166722000000	-3.804341000000	-3.542237000000
H	-1.132583000000	-4.234903000000	-2.128955000000
H	-2.885127000000	-3.880450000000	-1.874088000000
S	-3.487037000000	1.287837000000	0.926384000000
C	-4.750205000000	2.504659000000	0.405649000000
H	-5.244788000000	2.148068000000	-0.510835000000
H	-5.476276000000	2.636930000000	1.223446000000
H	-4.225336000000	3.450951000000	0.212312000000
C	-4.589463000000	-0.166339000000	1.111360000000
H	-5.232557000000	-0.012988000000	1.992816000000
H	-5.194304000000	-0.301156000000	0.201682000000
H	-3.934334000000	-1.035641000000	1.268587000000

^{1}TS$_{BD}$

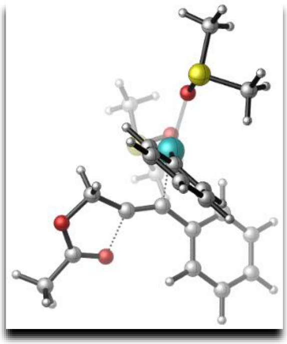

Frequency -215.4236

Zero-point correction= 0.421752 (Hartree/Particle)

Thermal correction to Energy= 0.456034

Thermal correction to Enthalpy= 0.456979

Thermal correction to Gibbs Free Energy= 0.349128

Sum of electronic and zero-point Energies= -2039.911421

Sum of electronic and thermal Energies= -2039.877138

Sum of electronic and thermal Enthalpies= -2039.876194

Sum of electronic and thermal Free Energies= -2039.984044

E(RM06L) = -2041.87728359

**Atom**	**X**	**Y**	**Z**
C	1.679711000000	-0.659208000000	-0.842658000000
O	3.462582000000	-1.202046000000	-0.936029000000
C	3.541217000000	-1.708002000000	-2.082508000000
O	2.482417000000	-1.697655000000	-2.880657000000
C	1.319603000000	-1.054791000000	-2.234101000000
C	4.772515000000	-2.365875000000	-2.621473000000
H	5.661396000000	-1.965331000000	-2.116109000000
H	4.839353000000	-2.227027000000	-3.710021000000
H	4.711898000000	-3.449472000000	-2.417708000000
H	1.056525000000	-0.197493000000	-2.875990000000
C	1.231072000000	-0.235063000000	0.304166000000
C	1.779359000000	-0.094387000000	1.657573000000
C	2.778668000000	-0.981085000000	2.132364000000
H	3.149364000000	-1.773760000000	1.477839000000
C	1.294048000000	0.915330000000	2.525797000000
H	0.517287000000	1.595466000000	2.161564000000
C	3.280871000000	-0.849721000000	3.433177000000
H	4.047362000000	-1.545736000000	3.788810000000
C	2.802700000000	0.163888000000	4.281331000000
H	3.197468000000	0.260671000000	5.297569000000
C	1.810392000000	1.046096000000	3.822360000000
H	1.429791000000	1.834098000000	4.480164000000
Pd	-0.678063000000	0.349165000000	-0.141909000000
H	0.503377000000	-1.796953000000	-2.249934000000
C	-1.281880000000	-1.548474000000	-0.008834000000
O	-0.087221000000	2.515538000000	-0.176934000000
O	-2.666776000000	1.172011000000	-0.319801000000
C	-1.906368000000	-2.162619000000	-1.116835000000
C	-1.143275000000	-2.275817000000	1.192206000000
C	-2.389316000000	-3.483554000000	-1.020981000000
H	-2.039925000000	-1.611075000000	-2.055615000000
C	-1.637566000000	-3.590979000000	1.284603000000
H	-0.640842000000	-1.825491000000	2.054940000000
C	-2.257998000000	-4.197867000000	0.179626000000
H	-2.872954000000	-3.946423000000	-1.888360000000
H	-1.526396000000	-4.143878000000	2.224001000000
H	-2.633615000000	-5.223374000000	0.253753000000
S	1.073514000000	2.978837000000	-1.112057000000
C	2.345827000000	3.698207000000	-0.006442000000
H	2.739662000000	2.871214000000	0.602336000000
H	3.149564000000	4.134585000000	-0.620803000000
H	1.882364000000	4.458945000000	0.640449000000
C	0.464203000000	4.539876000000	-1.849973000000
H	1.276675000000	5.008719000000	-2.427815000000
H	-0.367938000000	4.273853000000	-2.517648000000
H	0.109420000000	5.205443000000	-1.048210000000
S	-3.981239000000	0.308650000000	-0.267048000000

C	-4.291269000000	-0.066554000000	1.499169000000
H	-4.189921000000	0.855462000000	2.092193000000
H	-5.301146000000	-0.496711000000	1.595459000000
H	-3.535726000000	-0.808342000000	1.797680000000
C	-5.267263000000	1.587111000000	-0.493302000000
H	-6.257667000000	1.123896000000	-0.360218000000
H	-5.104269000000	2.399238000000	0.231294000000
H	-5.166140000000	1.966229000000	-1.520333000000

^{1}D

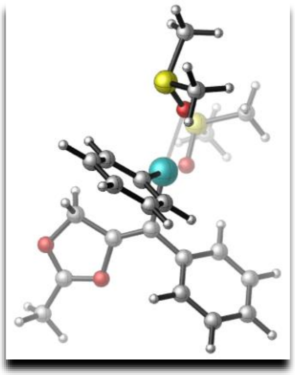

Zero-point correction= 0.423671 (Hartree/Particle)

Thermal correction to Energy= 0.457690

Thermal correction to Enthalpy= 0.458634

Thermal correction to Gibbs Free Energy= 0.352889

Sum of electronic and zero-point Energies= -2039.917189

Sum of electronic and thermal Energies= -2039.883170

Sum of electronic and thermal Enthalpies= -2039.882226

Sum of electronic and thermal Free Energies= -2039.987971

E(RM06L) = -2041.88556662

**Atom**	**X**	**Y**	**Z**
C	2.200026000000	-0.107488000000	-1.033745000000
O	3.679188000000	0.067320000000	-1.093887000000
C	4.095121000000	-0.362440000000	-2.236342000000
O	3.183419000000	-0.846114000000	-3.043002000000
C	1.850812000000	-0.735721000000	-2.358522000000
C	5.522700000000	-0.317451000000	-2.634395000000
H	6.117859000000	0.169140000000	-1.851224000000
H	5.624605000000	0.228514000000	-3.587201000000
H	5.890561000000	-1.344043000000	-2.803920000000
H	1.213270000000	-0.118350000000	-3.011325000000
C	1.479618000000	0.277062000000	0.026261000000
C	2.053523000000	0.801760000000	1.282920000000
C	3.065493000000	0.089803000000	1.973054000000
H	3.419850000000	-0.865039000000	1.571765000000
C	1.577126000000	2.016490000000	1.834639000000
H	0.788822000000	2.559747000000	1.303855000000
C	3.591393000000	0.585073000000	3.175166000000
H	4.365309000000	0.017751000000	3.702451000000
C	3.126092000000	1.800321000000	3.702400000000
H	3.539521000000	2.186905000000	4.639279000000
C	2.120899000000	2.513636000000	3.025869000000
H	1.755002000000	3.461263000000	3.434922000000
Pd	-0.505181000000	0.110999000000	-0.126317000000
H	1.445598000000	-1.757964000000	-2.282368000000
C	-0.270576000000	-1.834938000000	0.270371000000
O	-0.698639000000	2.314528000000	-0.555205000000
O	-2.729748000000	0.077729000000	-0.070035000000
C	-0.586989000000	-2.806301000000	-0.709135000000
C	0.106974000000	-2.277197000000	1.558244000000
C	-0.549060000000	-4.181616000000	-0.400239000000
H	-0.886472000000	-2.498874000000	-1.718753000000
C	0.136253000000	-3.650646000000	1.865561000000
H	0.385464000000	-1.548754000000	2.327687000000
C	-0.191914000000	-4.606818000000	0.888513000000
H	-0.800927000000	-4.917405000000	-1.172560000000
H	0.423287000000	-3.972839000000	2.872923000000
H	-0.161814000000	-5.674458000000	1.128864000000

S -2.076734000000 2.894234000000 -1.014390000000

C -1.669781000000 4.623043000000 -1.456626000000

H -1.039326000000 4.587091000000 -2.356684000000

H -2.602021000000 5.168466000000 -1.673111000000

H -1.119317000000 5.089920000000 -0.625614000000

C -3.026831000000 3.240633000000 0.514876000000

H -3.949912000000 3.781582000000 0.250634000000

H -3.265125000000 2.254287000000 0.938110000000

H -2.407925000000 3.827669000000 1.210792000000

S -3.505890000000 -1.263198000000 -0.335393000000

C -3.697326000000 -2.078412000000 1.292537000000

H -4.079146000000 -1.349508000000 2.023974000000

H -4.388154000000 -2.929355000000 1.178885000000

H -2.698312000000 -2.435492000000 1.583803000000

C -5.233364000000 -0.698630000000 -0.545550000000

H -5.884844000000 -1.581075000000 -0.647114000000

H -5.531987000000 -0.087626000000 0.319881000000

H -5.268448000000 -0.102761000000 -1.468977000000

^{1}E

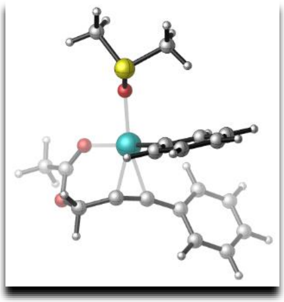

Zero-point correction= 0.343507 (Hartree/Particle)

Thermal correction to Energy= 0.370095

Thermal correction to Enthalpy= 0.371039

Thermal correction to Gibbs Free Energy= 0.282341

Sum of electronic and zero-point Energies= -1486.765038

Sum of electronic and thermal Energies= -1486.738450

Sum of electronic and thermal Enthalpies= -1486.737506

Sum of electronic and thermal Free Energies= -1486.826204

E(RM06L) = -1488.61662861

Atom X Y Z

H -1.025010000000 -5.327513000000 0.998185000000

O -0.735476000000 -3.754992000000 -1.161951000000

H 0.693067000000 -5.106430000000 1.478749000000

H -2.049579000000 -0.091407000000 2.201297000000

H -3.938164000000 1.158517000000 3.266334000000

H -5.768043000000 2.102692000000 1.847818000000

C 0.001162000000 -3.751542000000 -0.027414000000

C -0.007568000000 -5.104671000000 0.634382000000

H 0.258535000000 -5.889038000000 -0.091455000000

H 0.147131000000 -2.467546000000 -2.554286000000

C -2.824883000000 0.149280000000 0.182857000000

H -5.717204000000 1.800152000000 -0.633193000000

H -3.834704000000 0.554421000000 -1.704208000000

O 0.602298000000 -2.771436000000 0.435354000000

C -2.853846000000 0.330171000000 1.590023000000

C -3.912619000000 1.027606000000 2.180392000000

C -4.942444000000 1.556363000000 1.381508000000

C -4.915929000000 1.385864000000 -0.014074000000

C -3.865556000000 0.687874000000 -0.619173000000

C -1.087318000000 -1.326666000000 -1.216710000000

C -1.780271000000 -0.605479000000 -0.436927000000

C -0.797699000000 -2.548407000000 -1.992070000000

H -1.616591000000 -2.758633000000 -2.698755000000

Pd 0.430294000000 -0.519183000000 -0.033445000000

C 0.365741000000 1.394904000000 -0.581727000000

C 0.819740000000 1.752487000000 -1.862513000000

C -0.061803000000 2.372631000000 0.332225000000

C 0.860349000000 3.114411000000 -2.223354000000

C -0.014981000000 3.730880000000 -0.041146000000

C 0.446295000000 4.101175000000 -1.315273000000

H 1.142619000000 0.992493000000 -2.581410000000

H -0.437730000000 2.093779000000 1.321784000000

H 1.211917000000 3.394561000000 -3.221918000000

H -0.349554000000 4.494817000000 0.668873000000

C 4.910596000000 -0.202600000000 0.961416000000

S 3.424494000000 0.648364000000 0.332646000000

O 2.267514000000 -0.237054000000 0.958217000000

C 3.502052000000 2.152548000000 1.367399000000

H 4.958447000000 -1.179649000000 0.459677000000

H 5.797017000000 0.397607000000 0.701878000000

H 4.820850000000 -0.334017000000 2.050272000000

H 4.412661000000 2.712654000000 1.100383000000

H 2.607738000000 2.745824000000 1.126711000000

H 3.506988000000 1.865323000000 2.430085000000

H 0.473394000000 5.156844000000 -1.602609000000

$^{1}TS^{\alpha}_{EF}$

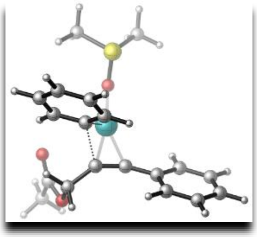

Frequency -288.1766

Zero-point correction= 0.342783 (Hartree/Particle)

Thermal correction to Energy= 0.369773

Thermal correction to Enthalpy= 0.370717

Thermal correction to Gibbs Free Energy= 0.279350

Sum of electronic and zero-point Energies= -1486.742700

Sum of electronic and thermal Energies= -1486.715711

Sum of electronic and thermal Enthalpies= -1486.714767

Sum of electronic and thermal Free Energies= -1486.806134

E(RM06L) = -1488.58949248

Atom X Y Z

C -1.024861000000 1.248992000000 0.365513000000

C 0.528240000000 0.501603000000 1.501742000000

C -1.719157000000 0.222288000000 -0.048221000000

C -2.831775000000 -0.669131000000 -0.058524000000

C -2.994135000000 -1.634297000000 -1.090476000000

H -2.251594000000 -1.687373000000 -1.892853000000

C -3.805582000000 -0.580609000000 0.978800000000

H -3.677027000000 0.159738000000 1.773861000000

C -4.100501000000 -2.486700000000 -1.081537000000

H -4.226119000000 -3.225119000000 -1.878832000000

C -5.059361000000 -2.387902000000 -0.055130000000

H -5.927566000000 -3.054100000000 -0.056216000000

C -4.911326000000 -1.434297000000 0.969568000000

H -5.660611000000 -1.361294000000 1.763438000000

C -1.051085000000 2.748139000000 0.384472000000

O -1.143599000000 3.323332000000 -0.941716000000

C 0.036619000000 3.353682000000 -1.646598000000

C -0.146414000000 4.061571000000 -2.966429000000

H -1.039008000000 3.684595000000 -3.489674000000

H 0.750135000000 3.922266000000 -3.583833000000

O 1.080375000000 2.871572000000 -1.221925000000

H -0.303321000000 5.138957000000 -2.788318000000

H -0.166349000000 3.142715000000 0.906058000000

Pd 0.294859000000 -0.141358000000 -0.439696000000

H -1.968404000000 3.078492000000 0.897859000000

O 1.881288000000 -1.215568000000 -1.372621000000

C 1.603977000000 1.395376000000 1.674397000000

C 0.059445000000 -0.306872000000 2.556281000000

C 2.233651000000 1.453715000000 2.931457000000

H 1.926369000000 2.034647000000 0.845951000000

C 0.707536000000 -0.234487000000 3.800229000000

H -0.797541000000 -0.971627000000 2.414655000000

C 1.791098000000 0.641409000000 3.988581000000

H 3.071076000000 2.143752000000 3.077889000000

H 0.352156000000 -0.858171000000 4.626798000000

H 2.282410000000 0.699366000000 4.964617000000

S 2.804424000000 -2.230382000000 -0.591612000000

C 4.465213000000 -1.473003000000 -0.669516000000

H 4.434587000000 -0.576986000000 -0.032834000000

H 5.201320000000 -2.190054000000 -0.272296000000

H 4.692791000000 -1.198222000000 -1.710763000000

C 3.058614000000 -3.584032000000 -1.791411000000

H 3.808975000000 -4.280789000000 -1.385092000000

H 2.093546000000 -4.098863000000 -1.903889000000

H 3.384216000000 -3.160709000000 -2.753787000000

$^{1}F^{\alpha}$

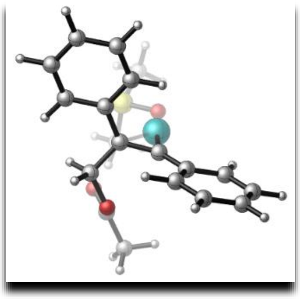

Zero-point correction= 0.345824 (Hartree/Particle)

Thermal correction to Energy= 0.372559

Thermal correction to Enthalpy= 0.373504

Thermal correction to Gibbs Free Energy= 0.283580

Sum of electronic and zero-point Energies= -1486.784254

Sum of electronic and thermal Energies= -1486.757519

Sum of electronic and thermal Enthalpies= -1486.756574

Sum of electronic and thermal Free Energies= -1486.846498

E(RM06L) = -1488.63354473

Atom X Y Z

C -0.772942000000 0.633491000000 -0.747236000000

C -0.965180000000 2.097132000000 -0.536877000000

C -1.254434000000 -0.328111000000 0.174887000000

C -2.471162000000 -0.974801000000 0.511027000000

C -2.510508000000 -1.945828000000 1.559179000000

H -1.583754000000 -2.177387000000 2.093529000000

C -3.675462000000 -0.658483000000 -0.194060000000

H -3.646374000000 0.092047000000 -0.989527000000

C -3.708751000000 -2.578145000000 1.884015000000

H -3.739948000000 -3.322018000000 2.685198000000

C -4.885537000000 -2.253305000000 1.176818000000

H -5.825669000000 -2.750658000000 1.436288000000

C -4.869157000000 -1.295272000000 0.142122000000

H -5.791270000000 -1.053576000000 -0.394277000000

C -0.405886000000 0.183878000000 -2.168731000000

O -0.382933000000 -1.249222000000 -2.317222000000

C 0.857948000000 -1.841285000000 -2.286650000000

C 0.744901000000 -3.334751000000 -2.465661000000

H 0.583145000000 -3.802856000000 -1.478831000000

H 1.682647000000 -3.725208000000 -2.883846000000

O 1.896654000000 -1.211211000000 -2.120137000000

H -0.106547000000 -3.598211000000 -3.109257000000

H 0.572264000000 0.596825000000 -2.464803000000

Pd 0.559845000000 -0.298866000000 0.730059000000

H -1.187184000000 0.540530000000 -2.863912000000

O 2.550322000000 -0.397313000000 1.445440000000

C -0.552609000000 3.031912000000 -1.517596000000

C -1.598212000000 2.582292000000 0.636533000000

C -0.776082000000 4.403777000000 -1.332612000000

H -0.053869000000 2.698999000000 -2.432078000000

C -1.821539000000 3.950159000000 0.813645000000

H -1.904585000000 1.877669000000 1.416825000000

C -1.410481000000 4.868340000000 -0.170389000000

H -0.451337000000 5.110619000000 -2.102221000000

H -2.313349000000 4.304771000000 1.724629000000

H -1.580998000000 5.939711000000 -0.027852000000

S 3.772509000000 0.222645000000 0.662042000000

C 4.553085000000 -1.185386000000 -0.197272000000

H 3.847294000000 -1.487645000000 -0.986724000000

H 5.498040000000 -0.836789000000 -0.644593000000

H 4.729310000000 -2.001434000000 0.520333000000

C 5.009994000000 0.480679000000 1.979747000000

H 5.955161000000 0.806173000000 1.516868000000

H 4.621186000000 1.273245000000 2.635187000000

H 5.142571000000 -0.455083000000 2.543701000000

^{1}G

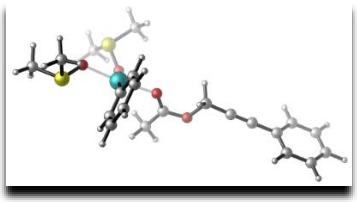

Zero-point correction= 0.422358 (Hartree/Particle)

Thermal correction to Energy= 0.457509

Thermal correction to Enthalpy= 0.458454

Thermal correction to Gibbs Free Energy= 0.346770

Sum of electronic and zero-point Energies= -2039.915473

Sum of electronic and thermal Energies= -2039.880321

Sum of electronic and thermal Enthalpies= -2039.879377

Sum of electronic and thermal Free Energies= -2039.991060

E(RM06L) = -2041.89218070

Atom X Y Z

H 0.215813000000 1.826865000000 2.143016000000

O -2.340519000000 0.526857000000 0.910086000000

H 0.083203000000 0.172120000000 2.782602000000

H -7.068008000000 -2.113416000000 0.604090000000

H -9.563595000000 -2.204145000000 0.718351000000

H -10.920317000000 -0.214461000000 0.043797000000

C -1.017118000000 0.529842000000 0.994197000000

C -0.486298000000 0.995591000000 2.319839000000

H -1.300504000000 1.306725000000 2.987665000000

H -2.575058000000 0.725358000000 -1.173563000000

C -7.014341000000 -0.069970000000 -0.135165000000

H -9.781364000000 1.867476000000 -0.744774000000

H -7.285936000000 1.961420000000 -0.859756000000

O -0.312411000000 0.164452000000 0.019845000000

C -7.668302000000 -1.247103000000 0.311160000000

C -9.065664000000 -1.292645000000 0.373257000000

C -9.827631000000 -0.174002000000 -0.005990000000

C -9.187996000000 0.996524000000 -0.449358000000

C -7.791235000000 1.054265000000 -0.515592000000

C -4.365320000000 0.019463000000 -0.259908000000

C -5.589570000000 -0.019756000000 -0.202974000000

C -2.926599000000 0.040740000000 -0.381939000000

H -2.498194000000 -0.957432000000 -0.577527000000

Pd 1.796920000000 0.134550000000 0.029598000000

C 1.712357000000 -1.831898000000 -0.162422000000

C 1.890323000000 -2.671887000000 0.953304000000

C 1.467015000000 -2.385944000000 -1.433520000000

C 1.830744000000 -4.071252000000 0.791836000000

C 1.413330000000 -3.785300000000 -1.585640000000

C 1.596962000000 -4.627467000000 -0.475201000000

H 2.072027000000 -2.250518000000 1.948460000000

H 1.314961000000 -1.738322000000 -2.303770000000

H 1.963483000000 -4.720944000000 1.663767000000

H 1.222979000000 -4.213524000000 -2.575835000000

C 3.195010000000 4.619399000000 0.517822000000

S 2.689019000000 3.241609000000 -0.576125000000

O 1.762330000000 2.358457000000 0.320869000000

C 1.559100000000 4.189265000000 -1.665026000000

H 3.846581000000 4.190947000000 1.292838000000

H 3.753293000000 5.362097000000 -0.073942000000

H 2.300887000000 5.068739000000 0.976655000000

H 2.144863000000 4.931547000000 -2.230505000000

H 1.106020000000 3.466384000000 -2.359058000000

H 0.781351000000 4.676230000000 -1.056762000000

C 5.181730000000 -2.100968000000 -0.933886000000

S 4.738070000000 -1.010065000000 0.464739000000

O 3.877718000000 0.169088000000 -0.161395000000

C 6.348526000000 -0.179448000000 0.687001000000

H 5.578196000000 -1.493482000000 -1.761744000000

H 5.927662000000 -2.830923000000 -0.579958000000

H 4.257770000000 -2.616442000000 -1.234491000000

H 7.094518000000 -0.931940000000 0.987745000000

H 6.637870000000 0.312414000000 -0.253958000000

H 6.222427000000 0.561853000000 1.488902000000

H 1.550245000000 -5.714164000000 -0.597563000000

[1]H

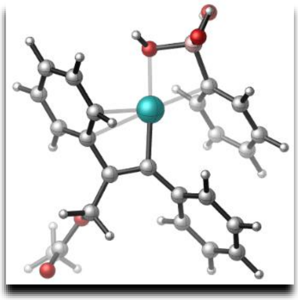

Zero-point correction= 0.401739 (Hartree/Particle)

Thermal correction to Energy= 0.432413

Thermal correction to Enthalpy= 0.433358

Thermal correction to Gibbs Free Energy= 0.336408

Sum of electronic and zero-point Energies= -1417.788184

Sum of electronic and thermal Energies= -1417.757510

Sum of electronic and thermal Enthalpies= -1417.756566

Sum of electronic and thermal Free Energies= -1417.853515

E(RM06L) = -1419.75624628

Atom	X	Y	Z
B	-3.758601000000	-0.493580000000	-0.342850000000
C	-2.720990000000	0.776108000000	-0.621321000000
Pd	-1.033671000000	-0.584626000000	0.111688000000
C	0.687539000000	0.380922000000	0.572955000000
C	1.596907000000	-0.631647000000	0.615714000000
C	0.920977000000	-1.964488000000	0.543424000000
C	1.288018000000	-2.991651000000	-0.372640000000
C	-0.143151000000	-2.224009000000	1.478135000000
C	0.944251000000	1.820420000000	0.674413000000
C	0.322058000000	2.591542000000	1.686215000000
C	1.864979000000	2.458924000000	-0.194488000000
O	-2.763713000000	-1.700255000000	-0.585067000000
C	0.644138000000	3.945078000000	1.851906000000
H	-0.403131000000	2.107707000000	2.347795000000
C	2.172643000000	3.817859000000	-0.033732000000
H	2.342190000000	1.869814000000	-0.983995000000
C	1.568889000000	4.563379000000	0.992575000000
H	0.170043000000	4.521294000000	2.653526000000
H	2.892032000000	4.294540000000	-0.708327000000
H	1.816388000000	5.622280000000	1.121453000000
C	0.617580000000	-4.213013000000	-0.365911000000
H	2.100939000000	-2.799542000000	-1.078983000000
C	-0.816400000000	-3.477935000000	1.458934000000
H	-0.266864000000	-1.559405000000	2.342144000000
C	-0.443915000000	-4.457017000000	0.541892000000
H	0.910211000000	-4.995076000000	-1.074520000000
H	-1.607234000000	-3.670367000000	2.191588000000
C	-2.846846000000	1.994581000000	0.116560000000
C	-1.840464000000	0.802116000000	-1.763110000000

C -2.211593000000 3.164842000000 -0.291701000000

H -3.501082000000 1.981352000000 0.995230000000

C -1.179456000000 1.989238000000 -2.162385000000

H -1.788515000000 -0.077590000000 -2.417636000000

C -1.375039000000 3.163844000000 -1.435394000000

H -2.352707000000 4.095518000000 0.268653000000

H -0.530108000000 1.985272000000 -3.044669000000

O -4.204193000000 -0.524242000000 1.034875000000

O -4.888512000000 -0.558830000000 -1.240298000000

H -5.168411000000 -0.657029000000 1.008933000000

H -3.016716000000 -2.365720000000 0.084602000000

H -4.583158000000 -0.627237000000 -2.160138000000

H -0.875623000000 4.088483000000 -1.742582000000

H -0.953805000000 -5.425584000000 0.532644000000

C 3.087835000000 -0.513008000000 0.752107000000

H 3.373558000000 0.459810000000 1.184853000000

H 3.518917000000 -1.314591000000 1.376197000000

O 3.684041000000 -0.617012000000 -0.592461000000

C 5.051317000000 -0.713763000000 -0.598945000000

C 5.579278000000 -0.759200000000 -2.020562000000

H 4.880960000000 -1.273596000000 -2.697167000000

H 5.709821000000 0.272490000000 -2.390515000000

H 6.560485000000 -1.253595000000 -2.026236000000

O 5.735172000000 -0.731822000000 0.413687000000

$^{1}TS_{HI}$

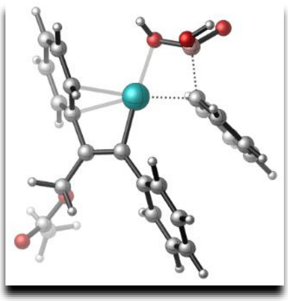

Frequency -146.8295

Zero-point correction= 0.400552 (Hartree/Particle)

Thermal correction to Energy= 0.430953

Thermal correction to Enthalpy= 0.431897

Thermal correction to Gibbs Free Energy= 0.335264

Sum of electronic and zero-point Energies= -1417.785223

Sum of electronic and thermal Energies= -1417.754822

Sum of electronic and thermal Enthalpies= -1417.753878

Sum of electronic and thermal Free Energies= -1417.850512

E(RM06L) = -1419.74725876

Atom	X	Y	Z
B	3.541647000000	-0.873516000000	0.226894000000
C	2.384821000000	0.550378000000	0.601391000000
Pd	0.926166000000	-0.827267000000	-0.216976000000
C	-0.634919000000	0.372863000000	-0.652943000000
C	-1.684457000000	-0.493121000000	-0.611507000000
C	-1.205006000000	-1.908934000000	-0.486693000000
C	-1.638796000000	-2.795758000000	0.542777000000
C	-0.283366000000	-2.404419000000	-1.471830000000
C	-0.684630000000	1.831270000000	-0.829883000000
C	-0.032163000000	2.447072000000	-1.925069000000
C	-1.425830000000	2.645193000000	0.061189000000
O	2.601931000000	-2.033482000000	0.500222000000
C	-0.140037000000	3.827215000000	-2.136683000000
H	0.550135000000	1.825053000000	-2.612577000000
C	-1.518200000000	4.029962000000	-0.146356000000
H	-1.928696000000	2.175723000000	0.912550000000
C	-0.880651000000	4.624963000000	-1.246486000000
H	0.357374000000	4.284587000000	-2.998779000000
H	-2.095413000000	4.644900000000	0.552620000000
H	-0.958543000000	5.704981000000	-1.409930000000
C	-1.160817000000	-4.102980000000	0.597915000000
H	-2.351219000000	-2.425160000000	1.285744000000

C 0.195377000000 -3.740653000000 -1.390546000000

H -0.111876000000 -1.820913000000 -2.383333000000

C -0.232283000000 -4.577441000000 -0.361889000000

H -1.502189000000 -4.772064000000 1.394998000000

H 0.877969000000 -4.111854000000 -2.162569000000

C 2.711387000000 1.728196000000 -0.127530000000

C 1.968694000000 0.719392000000 1.959346000000

C 2.689088000000 2.990757000000 0.473154000000

H 3.035278000000 1.614531000000 -1.167443000000

C 1.925163000000 1.984547000000 2.562050000000

H 1.709112000000 -0.166538000000 2.552061000000

C 2.289517000000 3.119950000000 1.817217000000

H 2.971122000000 3.880861000000 -0.099534000000

H 1.609204000000 2.091068000000 3.605915000000

O 3.923637000000 -0.833927000000 -1.149552000000

O 4.648090000000 -0.772777000000 1.116795000000

H 4.876847000000 -0.638096000000 -1.182597000000

H 2.802747000000 -2.705757000000 -0.180876000000

H 4.372660000000 -0.979406000000 2.025327000000

H 2.262765000000 4.110475000000 2.284945000000

H 0.129132000000 -5.609269000000 -0.305024000000

C -3.145638000000 -0.159478000000 -0.697413000000

H -3.302911000000 0.833720000000 -1.148580000000

H -3.714017000000 -0.903810000000 -1.282029000000

O -3.698832000000 -0.142397000000 0.670304000000

C -5.055035000000 0.035387000000 0.728770000000

C -5.540189000000 -0.009198000000 2.166206000000

H -4.804036000000 0.432767000000 2.854040000000

H -6.502424000000 0.515859000000 2.239383000000

H -5.694149000000 -1.060354000000 2.465945000000

O -5.763944000000 0.183781000000 -0.255886000000

^{1}I

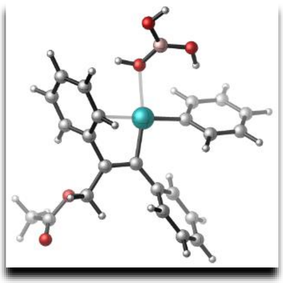

Zero-point correction= 0.402956 (Hartree/Particle)

Thermal correction to Energy= 0.433955

Thermal correction to Enthalpy= 0.434899

Thermal correction to Gibbs Free Energy= 0.334845

Sum of electronic and zero-point Energies= -1417.827894

Sum of electronic and thermal Energies= -1417.796896

Sum of electronic and thermal Enthalpies= -1417.795951

Sum of electronic and thermal Free Energies= -1417.896006

E(RM06L) = -1419.78914164

Atom X Y Z

B 3.758996000000 -2.000079000000 -0.160561000000

C 2.041305000000 1.116221000000 0.430963000000

Pd 0.870950000000 -0.443360000000 -0.026319000000

C -0.812344000000 0.576303000000 -0.395868000000

C -1.766034000000 -0.398130000000 -0.489933000000

C -1.221130000000 -1.797713000000 -0.423418000000

C -1.604738000000 -2.720637000000 0.590306000000

C -0.265977000000 -2.224023000000 -1.395259000000

C -1.051326000000 2.025129000000 -0.507982000000

C -1.555251000000 2.571926000000 -1.714005000000

C -0.804464000000 2.901934000000 0.575526000000

O 2.503196000000 -2.021255000000 0.479528000000

C -1.818900000000 3.945981000000 -1.826223000000

H -1.731078000000 1.906343000000 -2.566157000000

C -1.082678000000 4.269856000000 0.464898000000

H -0.391260000000 2.493632000000 1.502554000000

C -1.588702000000 4.799153000000 -0.735715000000

H -2.206478000000 4.348369000000 -2.768361000000

H -0.895743000000 4.929980000000 1.318636000000

H -1.795680000000 5.871033000000 -0.821285000000

C -1.035788000000 -3.995006000000 0.650146000000

H -2.345308000000 -2.403185000000 1.329265000000

C 0.308082000000 -3.516933000000 -1.321727000000

H -0.063797000000 -1.585938000000 -2.261734000000

C -0.064359000000 -4.394630000000 -0.295985000000

H -1.337890000000 -4.687440000000 1.443048000000

H 1.031098000000 -3.829895000000 -2.082238000000

C 2.273611000000 2.183982000000 -0.462931000000

C 2.774022000000 1.075583000000 1.642647000000

C 3.228029000000 3.172307000000 -0.165370000000

H 1.700541000000 2.251114000000 -1.392694000000

C 3.720556000000 2.078966000000 1.943327000000

H 2.605834000000 0.264527000000 2.360251000000

C 3.952138000000 3.125905000000 1.038077000000

H 3.403232000000 3.985672000000 -0.878899000000

H 4.271372000000 2.035476000000 2.890105000000

O 4.141178000000 -3.150680000000 -0.816610000000

O 4.558081000000 -0.898167000000 -0.108388000000

H 5.023169000000 -3.032744000000 -1.211410000000

H 2.039816000000 -2.867980000000 0.305682000000

H 4.119511000000 -0.145559000000 0.349060000000

H 4.689654000000 3.901637000000 1.269699000000

H 0.372895000000 -5.397286000000 -0.243952000000

C -3.250247000000 -0.170368000000 -0.590705000000

H -3.478030000000 0.882864000000 -0.817577000000

H -3.722304000000 -0.801391000000 -1.364686000000

O -3.867825000000 -0.513593000000 0.702730000000

C -5.235535000000 -0.490197000000 0.699805000000

C -5.778175000000 -0.876275000000 2.063619000000

H -5.237847000000 -0.349019000000 2.865179000000

H -6.850169000000 -0.642208000000 2.106345000000

H -5.639925000000 -1.958884000000 2.226596000000

O -5.908738000000 -0.207577000000 -0.280898000000

[1]J

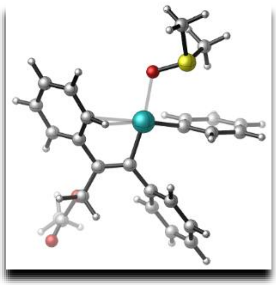

Zero-point correction= 0.432141 (Hartree/Particle)

Thermal correction to Energy= 0.464607

Thermal correction to Enthalpy= 0.465551

Thermal correction to Gibbs Free Energy= 0.362441

Sum of electronic and zero-point Energies= -1718.540298

Sum of electronic and thermal Energies= -1718.507832

Sum of electronic and thermal Enthalpies= -1718.506888

Sum of electronic and thermal Free Energies= -1718.609997

E(RM06L) = -1720.49549079

Atom X Y Z

C 1.472021000000 2.166121000000 -0.317571000000

C 1.911003000000 0.762967000000 -0.502180000000

C 0.952751000000 -0.221500000000 -0.587476000000

C 1.276988000000 -1.647919000000 -0.810741000000

Pd -0.912820000000 0.474992000000 -0.352666000000

C 0.733612000000 -2.339641000000 -1.920405000000

C 2.135407000000 -2.358609000000 0.062966000000

O -2.882579000000 1.515998000000 -0.098938000000

C 1.056992000000 -3.680816000000 -2.162335000000

H 0.059604000000 -1.801705000000 -2.595952000000

C 2.442487000000 -3.708058000000 -0.170701000000

H 2.555308000000 -1.837701000000 0.929093000000

C 1.909970000000 -4.373891000000 -1.285525000000

H 0.637318000000 -4.191369000000 -3.036241000000

H 2.153314000000 -5.425806000000 -1.468994000000

H 3.104371000000 -4.240296000000 0.521505000000

C 2.198773000000 3.101480000000 0.456566000000

C 0.253661000000 2.604202000000 -0.908369000000

C 1.706817000000 4.398298000000 0.660623000000

H 3.133639000000 2.787023000000 0.930300000000

C -0.249921000000 3.897187000000 -0.682902000000

H -0.215478000000 1.973925000000 -1.697535000000

C 0.477106000000 4.800033000000 0.106092000000

H 2.283084000000 5.102450000000 1.271098000000

H -1.195428000000 4.196962000000 -1.145829000000

S -4.181194000000 0.677207000000 -0.308734000000

C -4.766699000000 0.188195000000 1.360320000000

H -4.776856000000 1.074381000000 2.013995000000

H -5.772018000000 -0.253821000000 1.268403000000

H -4.051544000000 -0.559835000000 1.734210000000

C -5.448540000000 1.962600000000 -0.637905000000

H -6.443096000000 1.490802000000 -0.683351000000

H -5.405982000000 2.726116000000 0.154061000000

H -5.201629000000 2.412719000000 -1.610329000000

C -1.576448000000 -1.214143000000 0.485547000000

C -2.304796000000 -2.170598000000 -0.255343000000

C -1.449878000000 -1.394505000000 1.881674000000

C -2.915751000000 -3.264765000000 0.387519000000

C -2.057844000000 -2.492189000000 2.521020000000

C -2.797668000000 -3.427816000000 1.777186000000

H -2.393917000000 -2.071577000000 -1.342327000000

H -0.873671000000 -0.677766000000 2.476264000000

H -3.477740000000 -3.996009000000 -0.205628000000

H -1.946416000000 -2.617038000000 3.604704000000

H -3.266819000000 -4.283108000000 2.275592000000

H 0.100024000000 5.813594000000 0.276765000000

C 3.389868000000 0.499541000000 -0.643584000000

H 3.587820000000 -0.439405000000 -1.183403000000

H 3.908959000000 1.320865000000 -1.166629000000

O 3.988134000000 0.385140000000 0.704118000000

C 5.345175000000 0.219843000000 0.711046000000

O 6.036591000000 0.192640000000 -0.297334000000

C 5.855297000000 0.045607000000 2.130949000000

H 5.736187000000 -1.009532000000 2.432162000000

H 6.923932000000 0.298531000000 2.166296000000

H 5.286414000000 0.662667000000 2.842548000000

$^{1}TS_{JK}$

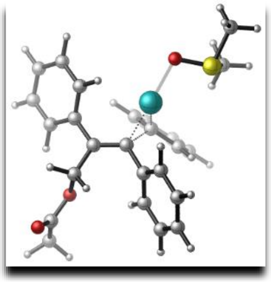

Frequency -275.4806

Zero-point correction= 0.431443 (Hartree/Particle)

Thermal correction to Energy= 0.463399

Thermal correction to Enthalpy= 0.464343

Thermal correction to Gibbs Free Energy= 0.362771

Sum of electronic and zero-point Energies= -1718.522405

Sum of electronic and thermal Energies= -1718.490450

Sum of electronic and thermal Enthalpies= -1718.489506

Sum of electronic and thermal Free Energies= -1718.591077

E(RM06L) = -1720.47208453

Atom	X	Y	Z
C	-0.520747000000	0.029568000000	1.295867000000
C	0.515002000000	-0.358313000000	-0.379047000000
C	1.532728000000	0.517245000000	-0.716591000000
C	1.408755000000	1.997742000000	-0.674654000000
C	2.451172000000	2.783293000000	-0.118314000000
C	0.285371000000	2.678349000000	-1.206103000000
C	0.721299000000	-1.839087000000	-0.484027000000
Pd	-1.444424000000	0.218068000000	-0.517967000000
C	-0.014371000000	-2.597083000000	-1.424799000000
C	1.634558000000	-2.521784000000	0.353656000000
O	-3.485927000000	0.625780000000	-1.206884000000
C	0.168768000000	-3.983076000000	-1.538572000000
H	-0.732935000000	-2.077472000000	-2.069336000000
C	1.811586000000	-3.910289000000	0.244923000000
H	2.206648000000	-1.947252000000	1.088064000000
S	-4.384814000000	-0.519067000000	-0.629380000000
C	1.080970000000	-4.647108000000	-0.700795000000
H	-0.402935000000	-4.546282000000	-2.284346000000
H	2.523799000000	-4.418294000000	0.904560000000
H	1.220024000000	-5.730208000000	-0.784562000000
C	2.351203000000	4.179947000000	-0.056206000000
H	3.329599000000	2.279282000000	0.295680000000
C	0.181847000000	4.076199000000	-1.135675000000
H	-0.499754000000	2.092348000000	-1.703479000000
C	1.212429000000	4.833989000000	-0.557317000000
H	3.164377000000	4.760715000000	0.392860000000
H	-0.699076000000	4.573947000000	-1.555460000000
C	-4.618871000000	-0.152841000000	1.153679000000
C	-6.075480000000	-0.040847000000	-1.187026000000
H	-4.924314000000	0.898366000000	1.272044000000
H	-5.378748000000	-0.839892000000	1.560450000000
H	-3.644058000000	-0.324426000000	1.635928000000
H	-6.813968000000	-0.724259000000	-0.738720000000
H	-6.269037000000	1.003457000000	-0.897548000000
H	-6.087308000000	-0.139124000000	-2.282176000000
C	-0.818215000000	-1.146534000000	2.031672000000
C	-0.246220000000	1.221440000000	2.013960000000
C	-0.866200000000	-1.121816000000	3.436918000000
H	-0.995617000000	-2.086526000000	1.501351000000
C	-0.285784000000	1.232241000000	3.416505000000
H	-0.003878000000	2.139995000000	1.474346000000
C	-0.599037000000	0.065525000000	4.136616000000
H	-1.106534000000	-2.041419000000	3.983213000000
H	-0.072119000000	2.165442000000	3.950313000000
H	-0.626761000000	0.081628000000	5.231392000000
H	1.136895000000	5.925653000000	-0.511005000000
C	2.880263000000	-0.004301000000	-1.174144000000
H	2.807981000000	-1.003836000000	-1.628393000000
H	3.353201000000	0.676106000000	-1.900831000000
O	3.780505000000	-0.103810000000	-0.004465000000
C	5.058765000000	-0.486626000000	-0.303774000000
O	5.457652000000	-0.711590000000	-1.437539000000
C	5.887141000000	-0.622303000000	0.961926000000
H	5.753361000000	-1.637755000000	1.373383000000
H	6.949705000000	-0.487968000000	0.715880000000
H	5.572546000000	0.097511000000	1.732004000000

[1]K

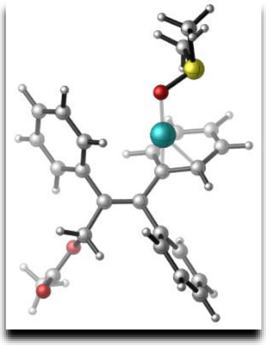

Zero-point correction= 0.432762 (Hartree/Particle)

Thermal correction to Energy= 0.465177

Thermal correction to Enthalpy= 0.466121

Thermal correction to Gibbs Free Energy= 0.361114

Sum of electronic and zero-point Energies= -1718.555476

Sum of electronic and thermal Energies= -1718.523061

Sum of electronic and thermal Enthalpies= -1718.522117

Sum of electronic and thermal Free Energies= -1718.627124

E(RM06L) = -1720.51000283

**Atom**	**X**	**Y**	**Z**
C	-0.090399000000	-0.653376000000	1.001104000000
C	1.136651000000	-0.562747000000	0.131122000000
C	1.730871000000	0.610579000000	-0.273041000000
C	1.096082000000	1.957383000000	-0.139472000000
C	1.733989000000	3.009618000000	0.558676000000
C	-0.127504000000	2.238036000000	-0.794568000000
C	1.755357000000	-1.888744000000	-0.210562000000
Pd	-1.739031000000	-0.294246000000	-0.328786000000
C	1.775143000000	-2.358082000000	-1.542554000000
C	2.297812000000	-2.708722000000	0.803757000000
O	-3.472324000000	0.263574000000	-1.576641000000
C	2.337436000000	-3.606672000000	-1.854300000000
H	1.335272000000	-1.736791000000	-2.330225000000
C	2.866795000000	-3.951512000000	0.490692000000
H	2.274729000000	-2.359834000000	1.841616000000
S	-4.674458000000	-0.316027000000	-0.764952000000
C	2.887906000000	-4.405053000000	-0.839379000000
H	2.342683000000	-3.955289000000	-2.892542000000
H	3.294573000000	-4.569088000000	1.287798000000
H	3.328559000000	-5.377693000000	-1.082226000000
C	1.148275000000	4.283464000000	0.631482000000
H	2.690151000000	2.813508000000	1.052397000000
C	-0.709129000000	3.513155000000	-0.726366000000
H	-0.601966000000	1.444789000000	-1.395045000000
C	-0.076978000000	4.539470000000	-0.005776000000
H	1.653509000000	5.079938000000	1.188845000000
H	-1.651705000000	3.704222000000	-1.250363000000
C	-4.858067000000	0.697855000000	0.758681000000
C	-6.147342000000	0.368766000000	-1.649807000000
H	-4.876290000000	1.766106000000	0.491703000000
H	-5.787269000000	0.398619000000	1.271675000000
H	-3.977869000000	0.472323000000	1.381271000000
H	-7.062811000000	0.091593000000	-1.103175000000
H	-6.047031000000	1.462181000000	-1.730183000000
H	-6.154680000000	-0.085875000000	-2.650967000000
C	-0.922403000000	-1.847331000000	0.963421000000
C	-0.270431000000	0.229880000000	2.132122000000
C	-1.822618000000	-2.128825000000	2.039158000000
H	-0.666383000000	-2.659167000000	0.273699000000
C	-1.143862000000	-0.085153000000	3.168786000000
H	0.341271000000	1.134010000000	2.192104000000
C	-1.920628000000	-1.272922000000	3.134072000000
H	-2.399910000000	-3.059668000000	2.008589000000
H	-1.220058000000	0.590399000000	4.028038000000
H	-2.587269000000	-1.517022000000	3.968348000000
H	-0.529429000000	5.535507000000	0.048321000000
C	3.102118000000	0.617037000000	-0.911237000000
H	3.392185000000	-0.364348000000	-1.312254000000

H 3.176338000000 1.360887000000 -1.722122000000

O 4.082161000000 0.987201000000 0.129286000000

C 5.361135000000 1.135104000000 -0.327070000000

O 5.694367000000 0.969941000000 -1.492131000000

C 6.285764000000 1.547184000000 0.804827000000

H 6.077353000000 0.966718000000 1.716777000000

H 7.328642000000 1.407301000000 0.490202000000

H 6.124697000000 2.612344000000 1.044357000000

^{1}L

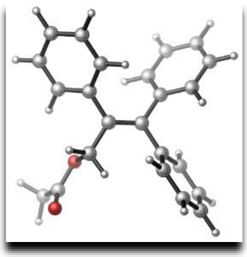

Zero-point correction= 0.355575 (Hartree/Particle)

Thermal correction to Energy= 0.378235

Thermal correction to Enthalpy= 0.379180

Thermal correction to Gibbs Free Energy= 0.299664

Sum of electronic and zero-point Energies= -1038.571898

Sum of electronic and thermal Energies= -1038.549237

Sum of electronic and thermal Enthalpies= -1038.548293

Sum of electronic and thermal Free Energies= -1038.627808

E(RM06L) = -1039.20962593

Atom X Y Z

C -0.920577000000 -1.648794000000 0.296303000000

C 0.130633000000 -0.699111000000 -0.182431000000

C -0.112908000000 0.633072000000 -0.432925000000

C -1.482203000000 1.216813000000 -0.546190000000

C -1.792058000000 2.477406000000 0.019387000000

C -2.482997000000 0.566796000000 -1.308992000000

C 1.492221000000 -1.305622000000 -0.334424000000

C 2.179339000000 -1.258212000000 -1.569675000000

C 2.097297000000 -1.991176000000 0.744126000000

C 3.442009000000 -1.853550000000 -1.714226000000

H 1.705580000000 -0.762453000000 -2.424080000000

C 3.362602000000 -2.577298000000 0.601961000000

H 1.567763000000 -2.052426000000 1.700544000000

C 4.040535000000 -2.509821000000 -0.627168000000

H 3.955470000000 -1.808173000000 -2.680422000000

H 3.820993000000 -3.090989000000 1.453744000000

H 5.026466000000 -2.972916000000 -0.738422000000

C -3.061380000000 3.050378000000 -0.149909000000

H -1.031269000000 2.997355000000 0.608699000000

C -3.747851000000 1.142954000000 -1.481105000000

H -2.253582000000 -0.395008000000 -1.777469000000

C -4.045896000000 2.386551000000 -0.898435000000

H -3.280633000000 4.022164000000 0.305702000000

H -4.501799000000 0.622457000000 -2.081359000000

C -0.993979000000 -2.961508000000 -0.227028000000

C -1.816044000000 -1.288576000000 1.331125000000

C -1.954123000000 -3.869759000000 0.240921000000

H -0.292287000000 -3.263090000000 -1.011588000000

C -2.764558000000 -2.202464000000 1.808819000000

H -1.754613000000 -0.285253000000 1.762732000000

C -2.843135000000 -3.494293000000 1.261357000000

H -2.003125000000 -4.876338000000 -0.188000000000

H -3.442516000000 -1.906241000000 2.616366000000

H -3.586511000000 -4.206593000000 1.634496000000

H -5.034727000000 2.837083000000 -1.034154000000

C 1.029049000000 1.612556000000 -0.614864000000

H 1.972937000000 1.127819000000 -0.896984000000

H 0.793904000000 2.383422000000 -1.367952000000

O 1.241515000000 2.287354000000 0.678447000000

C 2.300230000000 3.154078000000 0.691870000000

O 3.022715000000 3.360850000000 -0.272350000000

C 2.424172000000 3.824417000000 2.047803000000

H 2.155692000000 3.134896000000 2.862012000000

H 3.450802000000 4.193875000000 2.175797000000

H 1.735329000000 4.685746000000 2.093753000000

N = 2

c2 = 4-Phenyl-3-butynylacetate

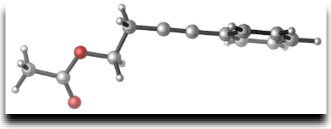

Zero-point correction= 0.203261 (Hartree/Particle)

Thermal correction to Energy= 0.217670

Thermal correction to Enthalpy= 0.218615

Thermal correction to Gibbs Free Energy= 0.157264

Sum of electronic and zero-point Energies= -614.697698

Sum of electronic and thermal Energies= -614.683289

Sum of electronic and thermal Enthalpies= -614.682345

Sum of electronic and thermal Free Energies= -614.743695

E(RM06L) = -615.079609257

Atom X Y Z

H -3.063110000000 -1.976706000000 0.989226000000

H -5.477844000000 -1.435048000000 1.327643000000

H -6.425639000000 0.696036000000 0.418203000000

H 1.592529000000 -1.098894000000 -1.741907000000

C -2.633959000000 -0.156401000000 -0.114310000000

H -4.945443000000 2.281577000000 -0.831558000000

H -2.531541000000 1.737710000000 -1.171403000000

C -3.481892000000 -1.046759000000 0.593049000000

C -4.834864000000 -0.738183000000 0.779755000000

C -5.367354000000 0.458034000000 0.269508000000

C -4.536103000000 1.347747000000 -0.432043000000

C -3.182002000000 1.047901000000 -0.625330000000

C -0.070374000000 -0.746940000000 -0.476382000000

C -1.252203000000 -0.468201000000 -0.308216000000

C 1.347559000000 -1.052155000000 -0.663313000000

H 1.583983000000 -2.049465000000 -0.246349000000

C 2.251785000000 -0.003937000000 0.006136000000

H 2.078270000000 0.046519000000 1.094081000000

H 2.080689000000 1.003465000000 -0.409052000000

O 3.619770000000 -0.415555000000 -0.253797000000

C 4.577172000000 0.406038000000 0.280281000000

O 4.319569000000 1.411382000000 0.924752000000

C 5.963001000000 -0.116070000000 -0.044403000000

H 6.097220000000 -1.127040000000 0.374499000000

H 6.096508000000 -0.195051000000 -1.135749000000

H 6.715081000000 0.563822000000 0.376325000000

^{2}B

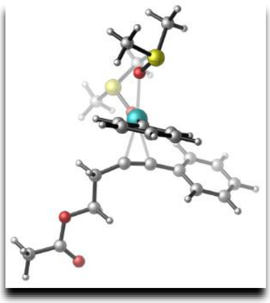

Zero-point correction= 0.449393 (Hartree/Particle)

Thermal correction to Energy= 0.485447

Thermal correction to Enthalpy= 0.486391

Thermal correction to Gibbs Free Energy= 0.372423

Sum of electronic and zero-point Energies= -2079.216628

Sum of electronic and thermal Energies= -2079.180574

Sum of electronic and thermal Enthalpies= -2079.179630

Sum of electronic and thermal Free Energies= -2079.293598

E(RM06L) = -2081.23190699

**Atom**	**X**	**Y**	**Z**
C	-1.424171000000	-0.048073000000	-0.056806000000
C	0.625316000000	1.913570000000	0.210357000000
C	-1.053881000000	-0.494073000000	1.068186000000
C	-0.874581000000	-0.970813000000	2.407457000000
C	-0.265712000000	-2.230104000000	2.649748000000
H	0.086327000000	-2.817075000000	1.796241000000
C	-1.330111000000	-0.186037000000	3.499643000000
H	-1.798257000000	0.783317000000	3.305969000000
C	-0.126065000000	-2.691979000000	3.962466000000
H	0.339050000000	-3.664786000000	4.149751000000
C	-0.587587000000	-1.914670000000	5.040808000000
H	-0.479862000000	-2.284406000000	6.065278000000
C	-1.189225000000	-0.665898000000	4.807102000000
H	-1.550633000000	-0.064602000000	5.646860000000
C	-2.409990000000	0.342697000000	-1.086971000000
H	-2.223662000000	-0.214177000000	-2.022916000000
Pd	0.681839000000	-0.025219000000	-0.238500000000
H	-2.284804000000	1.414691000000	-1.326430000000
O	0.734940000000	-2.238926000000	-0.696787000000
O	2.727519000000	0.037526000000	-0.916076000000
C	0.407248000000	2.848256000000	-0.821141000000
C	0.844825000000	2.350704000000	1.528539000000
C	0.437110000000	4.227557000000	-0.530534000000
H	0.206462000000	2.516104000000	-1.846524000000
C	0.876704000000	3.730848000000	1.807386000000
H	0.986759000000	1.628953000000	2.340446000000
C	0.674788000000	4.668207000000	0.781387000000
H	0.262145000000	4.952291000000	-1.333245000000
H	1.051785000000	4.068761000000	2.834487000000
H	0.690381000000	5.739370000000	1.005491000000
S	1.481238000000	-2.722372000000	-1.982391000000
C	0.465992000000	-4.119573000000	-2.590091000000
H	-0.484172000000	-3.694602000000	-2.944308000000
H	0.991149000000	-4.609469000000	-3.425394000000
H	0.283133000000	-4.824906000000	-1.765091000000
C	2.915861000000	-3.690868000000	-1.383106000000
H	3.408727000000	-4.176620000000	-2.240404000000
H	3.598293000000	-2.968801000000	-0.913591000000
H	2.576721000000	-4.434814000000	-0.646010000000
S	3.703017000000	1.172868000000	-0.400141000000
C	5.334910000000	0.361275000000	-0.521624000000
H	5.465448000000	-0.059590000000	-1.530109000000
H	6.115767000000	1.106116000000	-0.300455000000
H	5.357476000000	-0.433090000000	0.238326000000
C	3.883169000000	2.342538000000	-1.794161000000
H	4.715947000000	3.028251000000	-1.569429000000
H	4.066340000000	1.782235000000	-2.723840000000
H	2.939813000000	2.904717000000	-1.850804000000
C	-3.847890000000	0.083730000000	-0.606171000000
H	-4.014978000000	-0.984773000000	-0.386395000000
H	-4.074187000000	0.654800000000	0.310592000000
O	-4.710935000000	0.510194000000	-1.682643000000
C	-6.056454000000	0.350102000000	-1.418706000000
O	-6.470378000000	-0.113348000000	-0.369404000000
C	-6.888223000000	0.826119000000	-2.588590000000
H	-6.687122000000	1.890517000000	-2.792993000000
H	-6.626425000000	0.262989000000	-3.499349000000
H	-7.951639000000	0.686078000000	-2.356442000000

^{2}TS$^{\alpha}$BC

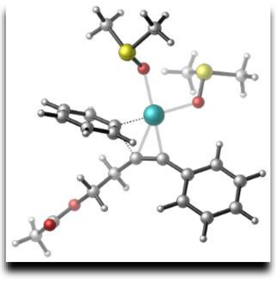

Frequency -260.7968

Zero-point correction= 0.449142 (Hartree/Particle)

Thermal correction to Energy= 0.485086

Thermal correction to Enthalpy= 0.486030

Thermal correction to Gibbs Free Energy= 0.372609

Sum of electronic and zero-point Energies= -2079.199813

Sum of electronic and thermal Energies= -2079.163869

Sum of electronic and thermal Enthalpies= -2079.162925

Sum of electronic and thermal Free Energies= -2079.276346

E(RM06L) = -2081.21479919

**Atom**	**X**	**Y**	**Z**
C	-1.209075000000	0.629882000000	0.472390000000
C	-0.764299000000	-1.093192000000	-0.569972000000
C	-0.468718000000	1.590826000000	0.007582000000
C	-0.321100000000	2.909402000000	-0.544430000000
C	0.887801000000	3.642927000000	-0.426425000000
H	1.738209000000	3.178784000000	0.080226000000
C	-1.431478000000	3.498028000000	-1.212585000000
H	-2.359219000000	2.927320000000	-1.317439000000
C	0.972557000000	4.936237000000	-0.952496000000
H	1.905409000000	5.500663000000	-0.855926000000
C	-0.132576000000	5.516013000000	-1.601016000000
H	-0.058974000000	6.528805000000	-2.009181000000
C	-1.332631000000	4.793836000000	-1.728394000000
H	-2.192794000000	5.241344000000	-2.235830000000
C	-2.362484000000	0.324776000000	1.372139000000
H	-2.490435000000	1.177663000000	2.063184000000
Pd	0.831086000000	-0.024641000000	0.122965000000
H	-2.145806000000	-0.564407000000	1.987327000000
O	2.656748000000	1.180659000000	0.557913000000
O	2.199003000000	-1.772882000000	0.344538000000
C	-1.072965000000	-2.297216000000	0.104384000000
C	-1.120180000000	-0.932481000000	-1.927874000000
C	-1.714247000000	-3.338131000000	-0.588113000000
H	-0.814695000000	-2.419434000000	1.161651000000
C	-1.746005000000	-1.987233000000	-2.613159000000
H	-0.925259000000	0.016133000000	-2.437354000000
C	-2.047228000000	-3.187269000000	-1.946206000000
H	-1.961964000000	-4.266027000000	-0.061464000000
H	-2.019808000000	-1.859316000000	-3.665414000000
H	-2.557767000000	-3.996161000000	-2.477693000000
S	3.971466000000	0.505577000000	1.084992000000
C	3.617227000000	-0.068993000000	2.787881000000
H	2.941612000000	-0.930025000000	2.684506000000
H	4.560911000000	-0.380634000000	3.264010000000
H	3.134432000000	0.742077000000	3.354477000000
C	5.012006000000	1.948216000000	1.509948000000
H	5.939194000000	1.591513000000	1.985983000000
H	5.250140000000	2.462070000000	0.567474000000
H	4.452986000000	2.618422000000	2.180396000000
S	2.183687000000	-2.818783000000	-0.832091000000
C	3.753367000000	-2.549420000000	-1.736662000000
H	4.599651000000	-2.558768000000	-1.033233000000
H	3.860433000000	-3.337821000000	-2.498805000000
H	3.672306000000	-1.568823000000	-2.228215000000
C	2.617636000000	-4.396771000000	-0.017790000000
H	2.775075000000	-5.164308000000	-0.792077000000
H	3.521781000000	-4.259787000000	0.594758000000
H	1.762604000000	-4.675711000000	0.614282000000
C	-3.667285000000	0.090901000000	0.594115000000

H -3.945976000000 0.976658000000 -0.003503000000

H -3.587212000000 -0.766795000000 -0.093651000000

O -4.682890000000 -0.166107000000 1.590324000000

C -5.929067000000 -0.432255000000 1.064019000000

O -6.144938000000 -0.463336000000 -0.136618000000

C -6.941741000000 -0.641709000000 2.167759000000

H -6.518822000000 -1.239491000000 2.989459000000

H -7.235536000000 0.336505000000 2.585751000000

H -7.833368000000 -1.130565000000 1.753668000000

$^{2}C^{\alpha}$

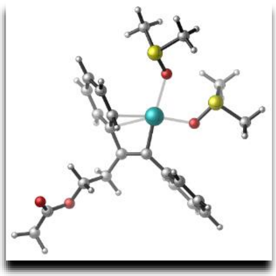

Zero-point correction= 0.451664 (Hartree/Particle)

Thermal correction to Energy= 0.487569

Thermal correction to Enthalpy= 0.488513

Thermal correction to Gibbs Free Energy= 0.375231

Sum of electronic and zero-point Energies= -2079.242700

Sum of electronic and thermal Energies= -2079.206795

Sum of electronic and thermal Enthalpies= -2079.205850

Sum of electronic and thermal Free Energies= -2079.319133

E(RM06L) = -2081.25840392

Atom	X	Y	Z
C	-1.774197000000	-0.041169000000	0.459635000000
C	-1.136649000000	-1.399458000000	0.444625000000
C	-0.869156000000	0.874595000000	0.045017000000
C	-0.899458000000	2.328259000000	-0.089967000000
C	-1.170104000000	3.147589000000	1.035624000000
H	-1.321326000000	2.678403000000	2.012893000000
C	-0.680244000000	2.942988000000	-1.348559000000
H	-0.464360000000	2.314092000000	-2.218301000000
C	-1.251004000000	4.539341000000	0.895468000000
H	-1.474342000000	5.161189000000	1.768277000000
C	-1.049380000000	5.135136000000	-0.361392000000
H	-1.117868000000	6.222332000000	-0.469127000000
C	-0.763204000000	4.333839000000	-1.481765000000
H	-0.613336000000	4.796695000000	-2.462502000000
C	-3.223737000000	0.169106000000	0.837908000000
H	-3.442841000000	1.248222000000	0.885174000000
Pd	0.853177000000	-0.145778000000	-0.062973000000
H	-3.415773000000	-0.245457000000	1.846660000000
O	1.952391000000	1.666987000000	0.281103000000
O	2.902169000000	-1.144566000000	0.117933000000
C	-1.158138000000	-2.288984000000	1.560018000000
C	-0.512120000000	-1.836814000000	-0.780184000000
C	-0.597575000000	-3.558484000000	1.458796000000
H	-1.631897000000	-1.966041000000	2.492361000000
C	0.056201000000	-3.144629000000	-0.852778000000
H	-0.742939000000	-1.305603000000	-1.711221000000
C	0.015736000000	-3.989931000000	0.252117000000
H	-0.640928000000	-4.239271000000	2.315217000000
H	0.470338000000	-3.493017000000	-1.804001000000
H	0.412069000000	-5.008198000000	0.181350000000
S	3.507635000000	1.735764000000	0.036565000000
C	4.280104000000	1.425379000000	1.666793000000
H	4.111763000000	0.359699000000	1.876645000000
H	5.358911000000	1.641084000000	1.601854000000
H	3.793724000000	2.055660000000	2.426909000000
C	3.775406000000	3.539928000000	-0.055802000000
H	4.857595000000	3.738645000000	-0.107887000000
H	3.281078000000	3.888664000000	-0.973587000000

H	3.321151000000	4.018570000000	0.824588000000
S	3.341012000000	-2.382299000000	-0.732961000000
C	4.978637000000	-1.938888000000	-1.423729000000
H	5.654545000000	-1.621215000000	-0.615235000000
H	5.385428000000	-2.813584000000	-1.955899000000
H	4.813973000000	-1.116102000000	-2.134410000000
C	3.906279000000	-3.624468000000	0.487297000000
H	4.355258000000	-4.476063000000	-0.048709000000
H	4.630212000000	-3.162864000000	1.176165000000
H	3.011748000000	-3.953353000000	1.035460000000
C	-4.171713000000	-0.514087000000	-0.156841000000
H	-4.076973000000	-0.077518000000	-1.167098000000
H	-3.967981000000	-1.596344000000	-0.244543000000
O	-5.517836000000	-0.315590000000	0.335519000000
C	-6.488274000000	-0.945457000000	-0.410418000000
O	-6.232251000000	-1.628112000000	-1.389843000000
C	-7.865013000000	-0.646080000000	0.141249000000
H	-7.870261000000	-0.685395000000	1.241233000000
H	-8.165568000000	0.373496000000	-0.155564000000
H	-8.586057000000	-1.362412000000	-0.273973000000

$^{2}TS^{\beta}_{BC}$

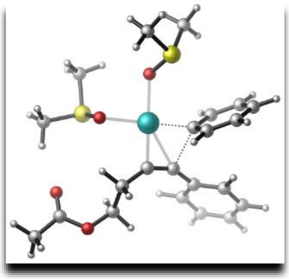

Frequency -245.4533

Zero-point correction= 0.449898 (Hartree/Particle)

Thermal correction to Energy= 0.485453

Thermal correction to Enthalpy= 0.486397

Thermal correction to Gibbs Free Energy= 0.376256

Sum of electronic and zero-point Energies= -2079.204507

Sum of electronic and thermal Energies= -2079.168952

Sum of electronic and thermal Enthalpies= -2079.168008

Sum of electronic and thermal Free Energies= -2079.278149

E(RM06L) = -2081.20808288

**Atom**	**X**	**Y**	**Z**
C	0.006038000000	1.746873000000	-0.222049000000
C	1.859706000000	0.746209000000	-0.494488000000
C	0.308046000000	3.014282000000	0.420171000000
C	-0.477863000000	3.414724000000	1.531338000000
H	-1.237604000000	2.731856000000	1.923549000000
C	1.310169000000	3.884980000000	-0.071603000000
H	1.925792000000	3.569659000000	-0.918945000000
C	-0.276932000000	4.670460000000	2.118356000000
H	-0.888372000000	4.972736000000	2.974221000000
C	0.709393000000	5.534411000000	1.613982000000
H	0.866365000000	6.514009000000	2.076141000000
C	1.499531000000	5.138991000000	0.519593000000
H	2.267305000000	5.812587000000	0.126739000000
C	-0.880835000000	0.921208000000	-0.678224000000
C	-2.234226000000	0.778480000000	-1.286126000000
H	-2.528290000000	-0.282573000000	-1.277045000000
Pd	0.345009000000	-0.588052000000	-0.163673000000
H	-2.202412000000	1.115843000000	-2.338690000000
O	-1.246111000000	-2.092691000000	-0.169755000000
O	1.689465000000	-2.208046000000	0.564017000000
C	2.237019000000	0.949879000000	-1.841066000000
C	2.799483000000	0.951587000000	0.543296000000
C	3.565318000000	1.297572000000	-2.149817000000
H	1.494856000000	0.848398000000	-2.638797000000
C	4.118324000000	1.307083000000	0.224475000000
H	2.496176000000	0.838458000000	1.588922000000

C	4.503852000000	1.476493000000	-1.120190000000
H	3.857870000000	1.446566000000	-3.194462000000
H	4.846237000000	1.466929000000	1.027296000000
H	5.531191000000	1.767701000000	-1.361010000000
S	-1.809246000000	-2.467682000000	1.254606000000
C	-0.849625000000	-3.933833000000	1.788937000000
H	0.199243000000	-3.603761000000	1.833014000000
H	-1.206442000000	-4.246326000000	2.783471000000
H	-0.967522000000	-4.743789000000	1.052553000000
C	-3.392993000000	-3.291648000000	0.883923000000
H	-3.761301000000	-3.761279000000	1.810219000000
H	-4.089415000000	-2.507232000000	0.549564000000
H	-3.224082000000	-4.044076000000	0.098016000000
S	3.001679000000	-2.443613000000	-0.264418000000
C	4.222095000000	-2.993568000000	0.981162000000
H	3.788640000000	-3.802298000000	1.589242000000
H	5.133695000000	-3.331223000000	0.463038000000
H	4.450147000000	-2.120271000000	1.608550000000
C	2.753367000000	-4.048269000000	-1.114225000000
H	3.681873000000	-4.316917000000	-1.643070000000
H	2.475467000000	-4.823908000000	-0.384393000000
H	1.941654000000	-3.897324000000	-1.840816000000
C	-3.284590000000	1.600627000000	-0.507952000000
H	-3.309259000000	1.283983000000	0.547147000000
H	-3.082712000000	2.679767000000	-0.576122000000
O	-4.598811000000	1.422847000000	-1.093622000000
C	-5.278181000000	0.307520000000	-0.686520000000
O	-4.812230000000	-0.512095000000	0.099311000000
C	-6.657787000000	0.260036000000	-1.299348000000
H	-7.330102000000	0.921684000000	-0.726109000000
H	-6.643686000000	0.621970000000	-2.338076000000
H	-7.048987000000	-0.764790000000	-1.249554000000

$^{2}TS_{BD}$

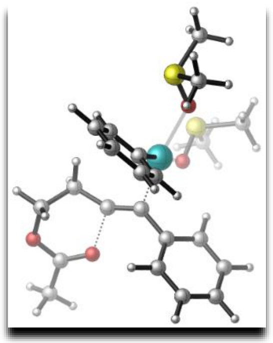

Frequency -249.4597

Zero-point correction= 0.449956 (Hartree/Particle)

Thermal correction to Energy= 0.485306

Thermal correction to Enthalpy= 0.486250

Thermal correction to Gibbs Free Energy= 0.376716

Sum of electronic and zero-point Energies= -2079.198385

Sum of electronic and thermal Energies= -2079.163035

Sum of electronic and thermal Enthalpies= -2079.162091

Sum of electronic and thermal Free Energies= -2079.271625

E(RM06L) = -2081.20301757

**Atom**	**X**	**Y**	**Z**
C	1.943416000000	-0.119781000000	-0.921391000000
O	3.805509000000	0.273295000000	-0.777905000000
C	1.352036000000	0.250284000000	0.176008000000
C	1.838067000000	0.740070000000	1.474872000000
C	2.801006000000	-0.002916000000	2.198341000000
H	3.182890000000	-0.937563000000	1.777689000000
C	1.332342000000	1.939495000000	2.029561000000
H	0.585589000000	2.506587000000	1.465197000000
C	3.253216000000	0.451772000000	3.445182000000
H	3.995436000000	-0.133446000000	3.997775000000
C	2.758438000000	1.650870000000	3.983216000000
H	3.114682000000	2.004386000000	4.955955000000
C	1.801298000000	2.392768000000	3.269630000000

H 1.415043000000 3.329189000000 3.685505000000

C 1.904962000000 -0.752148000000 -2.254585000000

H 1.901304000000 0.001403000000 -3.065151000000

Pd -0.657152000000 0.116693000000 -0.119401000000

H 0.968255000000 -1.330477000000 -2.334651000000

C 3.073812000000 -1.724758000000 -2.464639000000

C -0.547404000000 -1.826670000000 0.296212000000

O -0.732595000000 2.329612000000 -0.536392000000

O -2.863425000000 0.172104000000 -0.126797000000

C -0.830241000000 -2.781791000000 -0.704906000000

C -0.283850000000 -2.270263000000 1.609786000000

C -0.868976000000 -4.156490000000 -0.392691000000

H -1.043890000000 -2.464504000000 -1.733432000000

C -0.329218000000 -3.643404000000 1.918007000000

H -0.034952000000 -1.548452000000 2.395153000000

C -0.622661000000 -4.588837000000 0.919450000000

H -1.094009000000 -4.884852000000 -1.179985000000

H -0.126728000000 -3.973305000000 2.943117000000

H -0.651343000000 -5.655864000000 1.162435000000

S -2.039291000000 2.955519000000 -1.123224000000

C -1.512241000000 4.640796000000 -1.603430000000

H -0.819072000000 4.532285000000 -2.449999000000

H -2.395437000000 5.221617000000 -1.913505000000

H -1.000750000000 5.116484000000 -0.752806000000

C -3.079456000000 3.410015000000 0.316222000000

H -3.954431000000 3.980679000000 -0.034639000000

H -3.395264000000 2.455345000000 0.760816000000

H -2.484456000000 3.996981000000 1.032780000000

S -3.691944000000 -1.118938000000 -0.483402000000

C -4.033905000000 -1.966517000000 1.102042000000

H -4.414494000000 -1.236439000000 1.832788000000

H -4.767802000000 -2.767586000000 0.917998000000

H -3.079240000000 -2.395075000000 1.441291000000

C -5.364978000000 -0.445371000000 -0.786681000000

H -6.058645000000 -1.284609000000 -0.953783000000

H -5.682590000000 0.161223000000 0.075055000000

H -5.304371000000 0.173349000000 -1.693547000000

H 2.983268000000 -2.256694000000 -3.421717000000

H 3.139688000000 -2.453062000000 -1.639803000000

O 4.363232000000 -1.038650000000 -2.564246000000

C 4.622071000000 -0.099499000000 -1.652346000000

C 6.008030000000 0.470633000000 -1.745371000000

H 6.075990000000 1.385016000000 -1.142403000000

H 6.731637000000 -0.271203000000 -1.365735000000

H 6.266098000000 0.674962000000 -2.796162000000

^{2}D

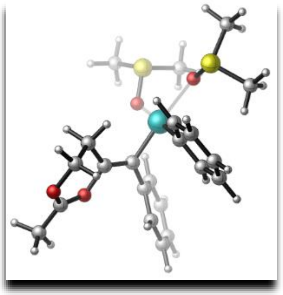

Zero-point correction= 0.452058 (Hartree/Particle)

Thermal correction to Energy= 0.487091

Thermal correction to Enthalpy= 0.488035

Thermal correction to Gibbs Free Energy= 0.380345

Sum of electronic and zero-point Energies= -2079.204795

Sum of electronic and thermal Energies= -2079.169762

Sum of electronic and thermal Enthalpies= -2079.168818

Sum of electronic and thermal Free Energies= -2079.276508

E(RM06L) = -2081.21026224

Atom X Y Z

C -2.142685000000 -0.041140000000 -0.986033000000

O -3.559858000000 -0.399332000000 -0.829371000000

C	-1.321004000000	-0.377581000000	0.032443000000
C	-1.810642000000	-0.955794000000	1.304564000000
C	-2.757431000000	-0.262933000000	2.099186000000
H	-3.125944000000	0.712513000000	1.765313000000
C	-1.310433000000	-2.197122000000	1.770501000000
H	-0.566217000000	-2.723639000000	1.164490000000
C	-3.195532000000	-0.799086000000	3.319195000000
H	-3.917727000000	-0.244286000000	3.927297000000
C	-2.709062000000	-2.039853000000	3.760313000000
H	-3.053951000000	-2.459772000000	4.710495000000
C	-1.769194000000	-2.735907000000	2.979204000000
H	-1.386094000000	-3.703439000000	3.320503000000
C	-1.890179000000	0.595243000000	-2.314711000000
H	-1.893863000000	-0.154312000000	-3.130439000000
Pd	0.646686000000	-0.120138000000	-0.115862000000
H	-0.893445000000	1.062207000000	-2.289902000000
C	-2.956140000000	1.650334000000	-2.593481000000
C	0.333574000000	1.806975000000	0.326454000000
O	0.943037000000	-2.298367000000	-0.618589000000
O	2.874501000000	-0.003993000000	-0.051764000000
C	0.588049000000	2.813533000000	-0.635257000000
C	-0.038847000000	2.207002000000	1.630081000000
C	0.493266000000	4.178974000000	-0.296632000000
H	0.883964000000	2.542983000000	-1.656531000000
C	-0.126959000000	3.570822000000	1.967992000000
H	-0.266744000000	1.450499000000	2.389107000000
C	0.139233000000	4.561408000000	1.006499000000
H	0.700303000000	4.941499000000	-1.056472000000
H	-0.409456000000	3.858264000000	2.987194000000
H	0.065862000000	5.621473000000	1.270291000000
S	2.329061000000	-2.765965000000	-1.169423000000
C	1.995134000000	-4.474030000000	-1.736682000000
H	1.332304000000	-4.400316000000	-2.610850000000
H	2.945598000000	-4.950092000000	-2.025645000000
H	1.498819000000	-5.034514000000	-0.929852000000
C	3.349805000000	-3.179545000000	0.296276000000
H	4.290220000000	-3.646344000000	-0.038738000000
H	3.549873000000	-2.217766000000	0.789915000000
H	2.787129000000	-3.852393000000	0.961632000000
S	3.584909000000	1.372480000000	-0.313978000000
C	3.758738000000	2.181648000000	1.319227000000
H	4.185324000000	1.465725000000	2.038640000000
H	4.405752000000	3.066193000000	1.204400000000
H	2.747581000000	2.486911000000	1.626836000000
C	5.335717000000	0.894608000000	-0.548900000000
H	5.942787000000	1.808886000000	-0.643665000000
H	5.672220000000	0.286833000000	0.304879000000
H	5.390836000000	0.314294000000	-1.481204000000
H	-2.944850000000	2.038151000000	-3.621691000000
H	-2.916086000000	2.484514000000	-1.876239000000
O	-4.308295000000	1.047617000000	-2.460306000000
C	-4.489754000000	0.088253000000	-1.584124000000
C	-5.867275000000	-0.465731000000	-1.456004000000
H	-5.940893000000	-1.075640000000	-0.545847000000
H	-6.603333000000	0.353079000000	-1.437281000000
H	-6.096393000000	-1.097269000000	-2.332990000000

^{2}E

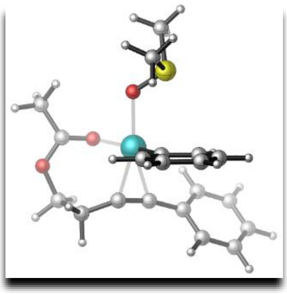

Zero-point correction= 0.371534 (Hartree/Particle)

Thermal correction to Energy= 0.400287

Thermal correction to Enthalpy= 0.401231

Thermal correction to Gibbs Free Energy= 0.306585

Sum of electronic and zero-point Energies= -1526.054110

Sum of electronic and thermal Energies= -1526.025358

Sum of electronic and thermal Enthalpies= -1526.024413

Sum of electronic and thermal Free Energies= -1526.119060

E(RM06L) = -1527.94479112

**Atom**	**X**	**Y**	**Z**
C	0.740460000000	-1.688917000000	1.096788000000
C	0.148726000000	1.288002000000	0.982280000000
C	1.600312000000	-1.207337000000	0.300529000000
C	2.787706000000	-0.790175000000	-0.384235000000
C	2.855134000000	-0.826731000000	-1.801687000000
H	1.980940000000	-1.158069000000	-2.370597000000
C	3.917513000000	-0.365137000000	0.363099000000
H	3.857601000000	-0.329329000000	1.454488000000
C	4.036028000000	-0.454770000000	-2.452861000000
H	4.087946000000	-0.490141000000	-3.545241000000
C	5.155281000000	-0.043906000000	-1.706364000000
H	6.077874000000	0.242926000000	-2.220397000000
C	5.093439000000	-0.000918000000	-0.302273000000
H	5.965241000000	0.319759000000	0.275949000000
C	0.190505000000	-2.744334000000	1.979432000000
H	-0.477980000000	-2.325300000000	2.751729000000
Pd	-0.420303000000	-0.334972000000	-0.013076000000
H	1.040794000000	-3.207090000000	2.516430000000
O	-2.048328000000	0.682657000000	-0.934680000000
C	-0.429761000000	1.531817000000	2.240370000000
C	1.054350000000	2.196832000000	0.412373000000
C	-0.107304000000	2.720740000000	2.925197000000
H	-1.122596000000	0.815057000000	2.693249000000
C	1.365137000000	3.383294000000	1.107732000000
H	1.524063000000	1.994497000000	-0.555769000000
C	0.786274000000	3.645399000000	2.358937000000
H	-0.553197000000	2.913076000000	3.906884000000
H	2.070227000000	4.094838000000	0.665089000000
H	1.038298000000	4.563747000000	2.898234000000
S	-1.954249000000	2.206025000000	-1.350865000000
C	-3.143433000000	2.282506000000	-2.733013000000
H	-4.106432000000	1.857493000000	-2.412156000000
H	-3.255315000000	3.333305000000	-3.043951000000
H	-2.717049000000	1.691064000000	-3.555922000000
C	-2.915535000000	3.130448000000	-0.100348000000
H	-3.027465000000	4.170810000000	-0.445972000000
H	-3.896092000000	2.650475000000	0.040751000000
H	-2.328465000000	3.102566000000	0.829557000000
C	-0.540103000000	-3.874675000000	1.220778000000
H	-0.017049000000	-4.120897000000	0.283154000000
H	-0.598605000000	-4.764660000000	1.864257000000
O	-1.940910000000	-3.586311000000	0.923770000000
C	-2.224052000000	-2.863491000000	-0.180558000000
O	-1.360719000000	-2.307953000000	-0.877623000000
C	-3.698950000000	-2.831520000000	-0.483831000000
H	-3.887090000000	-3.461932000000	-1.370054000000
H	-4.299187000000	-3.208025000000	0.355128000000
H	-3.985256000000	-1.801778000000	-0.745302000000

$^{2}TS^{\alpha}_{EF}$

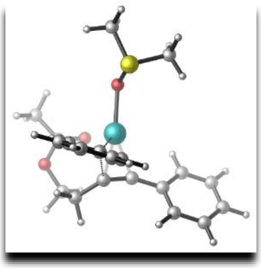

Frequency -312.9327

Zero-point correction= 0.370575 (Hartree/Particle)

Thermal correction to Energy= 0.398790

Thermal correction to Enthalpy= 0.399734

Thermal correction to Gibbs Free Energy= 0.306342

Sum of electronic and zero-point Energies= -1526.030089

Sum of electronic and thermal Energies= -1526.001875

Sum of electronic and thermal Enthalpies= -1526.000930

Sum of electronic and thermal Free Energies= -1526.094323

E(RM06L) = -1527.91508588

**Atom**	**X**	**Y**	**Z**
C	-0.851017000000	1.427578000000	0.590671000000
C	0.374303000000	0.250648000000	1.682869000000
C	-1.679486000000	0.634239000000	-0.036716000000
C	-0.622757000000	2.893408000000	0.802288000000
Pd	0.265242000000	-0.072639000000	-0.349952000000
C	1.571138000000	0.876883000000	2.094687000000
C	-0.339969000000	-0.589665000000	2.564308000000
C	-2.911714000000	-0.043268000000	-0.264498000000
C	-3.152713000000	-0.726196000000	-1.490033000000
C	-3.925087000000	-0.024575000000	0.738503000000
H	-2.380010000000	-0.715659000000	-2.265150000000
C	-4.372190000000	-1.374201000000	-1.700164000000
H	-3.736956000000	0.500358000000	1.679826000000
C	-5.142718000000	-0.670630000000	0.510003000000
H	-4.558472000000	-1.892359000000	-2.645559000000
C	-5.367002000000	-1.347341000000	-0.703939000000
H	-6.323340000000	-1.850638000000	-0.876663000000
H	-5.921178000000	-0.650896000000	1.278610000000
H	0.143815000000	3.059577000000	1.576765000000
H	-1.569516000000	3.317885000000	1.187781000000
C	-0.249371000000	3.675249000000	-0.474117000000
O	1.733837000000	-1.388456000000	-1.188749000000
O	0.697887000000	1.873924000000	-2.311130000000
S	2.182383000000	-2.693678000000	-0.426791000000
C	2.060582000000	0.637997000000	3.390880000000
H	2.109318000000	1.549620000000	1.418735000000
C	0.166773000000	-0.820539000000	3.852346000000
H	-1.284760000000	-1.045214000000	2.252570000000
C	1.363847000000	-0.210027000000	4.267218000000
H	2.988736000000	1.122040000000	3.711553000000
H	-0.385130000000	-1.471682000000	4.537991000000
H	1.746434000000	-0.386309000000	5.277044000000
C	1.283463000000	-4.055123000000	-1.253726000000
C	3.848899000000	-3.020272000000	-1.100632000000
H	1.426515000000	-3.983055000000	-2.342750000000
H	1.657191000000	-5.015307000000	-0.863585000000
H	0.220552000000	-3.938026000000	-0.996444000000
H	4.196658000000	-3.997601000000	-0.730148000000
H	3.807066000000	-2.999872000000	-2.200308000000
H	4.507050000000	-2.223162000000	-0.725971000000
H	-0.862696000000	3.354870000000	-1.330580000000
H	-0.390950000000	4.751306000000	-0.285553000000
O	1.158008000000	3.547249000000	-0.812166000000
C	1.490057000000	2.665202000000	-1.806803000000
C	2.938218000000	2.828603000000	-2.206015000000
H	2.998573000000	3.581457000000	-3.011276000000
H	3.553359000000	3.186374000000	-1.367658000000
H	3.321104000000	1.876978000000	-2.599207000000

$^{2}F^{\alpha}$

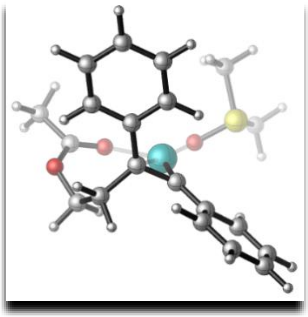

Zero-point correction= 0.373821 (Hartree/Particle)

Thermal correction to Energy= 0.401626

Thermal correction to Enthalpy= 0.402570

Thermal correction to Gibbs Free Energy= 0.310679

Sum of electronic and zero-point Energies= -1526.073828

Sum of electronic and thermal Energies= -1526.046022

Sum of electronic and thermal Enthalpies= -1526.045078

Sum of electronic and thermal Free Energies= -1526.136970

E(RM06L) = -1527.96239453

**Atom**	**X**	**Y**	**Z**
C	0.057075000000	1.482222000000	-0.264400000000
C	0.751823000000	1.888849000000	0.996633000000
C	-1.143870000000	0.721691000000	-0.250240000000
C	0.382433000000	2.225804000000	-1.579827000000
Pd	0.149749000000	-0.659519000000	-0.492364000000
C	2.026525000000	2.502054000000	0.968205000000
C	0.127883000000	1.690470000000	2.254072000000
C	-2.543779000000	0.958295000000	-0.326814000000
C	-3.448785000000	-0.115500000000	-0.583114000000
C	-3.062953000000	2.275726000000	-0.124712000000
H	-3.035536000000	-1.117814000000	-0.732787000000
C	-4.820486000000	0.124131000000	-0.649878000000
H	-2.368060000000	3.094854000000	0.085081000000
C	-4.438313000000	2.501529000000	-0.180224000000
H	-5.514491000000	-0.696016000000	-0.855431000000
C	-5.314423000000	1.429403000000	-0.445686000000
H	-6.392805000000	1.612357000000	-0.491711000000
H	-4.837222000000	3.507569000000	-0.020673000000
H	0.863130000000	3.189744000000	-1.341497000000
H	-0.563435000000	2.468096000000	-2.100642000000
C	1.228156000000	1.462913000000	-2.612558000000
O	0.167815000000	-2.831288000000	-0.283293000000
O	2.208363000000	-0.929410000000	-1.525044000000
S	-0.508831000000	-3.489015000000	0.970821000000
C	2.652041000000	2.904667000000	2.157413000000
H	2.538242000000	2.658368000000	0.013841000000
C	0.753489000000	2.098184000000	3.437237000000
H	-0.862338000000	1.223145000000	2.297062000000
C	2.020544000000	2.706908000000	3.395296000000
H	3.637305000000	3.379589000000	2.113685000000
H	0.247585000000	1.949181000000	4.396546000000
H	2.508063000000	3.028994000000	4.320420000000
C	-0.602796000000	-5.258262000000	0.516676000000
C	0.804100000000	-3.615225000000	2.241579000000
H	0.390043000000	-5.599031000000	0.185625000000
H	-0.954642000000	-5.832769000000	1.388151000000
H	-1.327895000000	-5.340209000000	-0.305715000000
H	0.419151000000	-4.196156000000	3.095056000000
H	1.695425000000	-4.090545000000	1.804107000000
H	1.035644000000	-2.589063000000	2.563085000000
H	0.769169000000	0.491710000000	-2.861375000000
H	1.327983000000	2.069061000000	-3.527703000000
O	2.604728000000	1.242359000000	-2.179152000000
C	2.978585000000	0.017223000000	-1.742548000000
C	4.474384000000	-0.087389000000	-1.589724000000
H	4.894192000000	-0.503672000000	-2.522317000000
H	4.930409000000	0.898080000000	-1.420883000000
H	4.715894000000	-0.778794000000	-0.770694000000

^{2}G

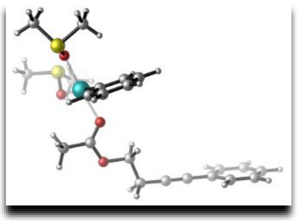

Zero-point correction= 0.450505 (Hartree/Particle)

Thermal correction to Energy= 0.487042

Thermal correction to Enthalpy= 0.487986

Thermal correction to Gibbs Free Energy= 0.370427

Sum of electronic and zero-point Energies= -2079.204430

Sum of electronic and thermal Energies= -2079.167893

Sum of electronic and thermal Enthalpies= -2079.166949

Sum of electronic and thermal Free Energies= -2079.284508

E(RM06L) = -2081.22142390

**Atom**	**X**	**Y**	**Z**
H	-7.107748000000	1.969388000000	-1.316127000000
H	-9.020472000000	1.313072000000	-2.783259000000
H	-10.048373000000	-0.957066000000	-2.562407000000
H	-3.937368000000	0.621705000000	3.125178000000
C	-7.046720000000	0.069381000000	-0.263732000000
H	-9.158480000000	-2.569507000000	-0.868824000000
H	-7.245292000000	-1.915158000000	0.598375000000
C	-7.558588000000	0.976532000000	-1.226067000000
C	-8.631072000000	0.604212000000	-2.045451000000
C	-9.208732000000	-0.670831000000	-1.921131000000
C	-8.708691000000	-1.576618000000	-0.970098000000
C	-7.636386000000	-1.214907000000	-0.145746000000
C	-5.024407000000	0.770596000000	1.308332000000
C	-5.952988000000	0.445155000000	0.577433000000
C	-3.893200000000	1.149314000000	2.153677000000
H	-3.920226000000	2.232375000000	2.379290000000
C	-2.567447000000	0.813131000000	1.457708000000
H	-2.458232000000	1.347408000000	0.500627000000
H	-2.456697000000	-0.267061000000	1.274783000000
O	-1.490217000000	1.250697000000	2.363665000000
C	-0.238789000000	1.022444000000	1.981640000000
O	-0.001703000000	0.462365000000	0.883878000000
C	0.798534000000	1.488939000000	2.961837000000
H	0.336757000000	1.990751000000	3.822574000000
H	1.381819000000	0.618418000000	3.305932000000
H	1.492356000000	2.168033000000	2.439529000000
Pd	1.928963000000	0.108090000000	0.131243000000
C	1.556729000000	-1.830658000000	0.245860000000
C	2.070165000000	-2.604808000000	1.303823000000
C	0.756330000000	-2.429976000000	-0.745990000000
C	1.789313000000	-3.985378000000	1.360605000000
C	0.485571000000	-3.810858000000	-0.683276000000
C	1.002583000000	-4.588581000000	0.367282000000
H	2.684765000000	-2.146554000000	2.086902000000
H	0.338567000000	-1.831263000000	-1.562690000000
H	2.185725000000	-4.582569000000	2.188973000000
H	-0.136863000000	-4.274019000000	-1.456658000000
C	4.120219000000	4.169651000000	-0.467804000000
S	2.909397000000	2.975525000000	-1.146476000000
O	2.241004000000	2.331475000000	0.111234000000
C	1.684684000000	4.184761000000	-1.774261000000
H	4.911356000000	3.580411000000	0.017653000000
H	4.545996000000	4.759547000000	-1.295025000000
H	3.623522000000	4.820131000000	0.268473000000
H	2.144211000000	4.773475000000	-2.584347000000
H	0.838746000000	3.604523000000	-2.170681000000
H	1.350909000000	4.834906000000	-0.950975000000
C	4.322976000000	-2.649025000000	-1.832316000000
S	4.641986000000	-1.469405000000	-0.471889000000

O 3.773279000000 -0.180147000000 -0.799625000000

C 6.299788000000 -0.901583000000 -0.983368000000

H 4.412462000000 -2.127673000000 -2.797553000000

H 5.050974000000 -3.472556000000 -1.753128000000

H 3.301015000000 -3.028344000000 -1.685466000000

H 6.991831000000 -1.757625000000 -0.946112000000

H 6.244554000000 -0.479901000000 -1.998346000000

H 6.616772000000 -0.135187000000 -0.261801000000

H 0.784851000000 -5.660212000000 0.414830000000

^{2}H

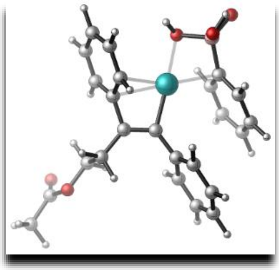

Zero-point correction= 0.429328 (Hartree/Particle)

Thermal correction to Energy= 0.461556

Thermal correction to Enthalpy= 0.462501

Thermal correction to Gibbs Free Energy= 0.361057

Sum of electronic and zero-point Energies= -1457.072081

Sum of electronic and thermal Energies= -1457.039853

Sum of electronic and thermal Enthalpies= -1457.038909

Sum of electronic and thermal Free Energies= -1457.140352

E(RM06L) = -1459.07989035

Atom X Y Z

B 3.961368000000 -0.822887000000 0.310610000000

C 3.064966000000 0.546170000000 0.618309000000

Pd 1.226854000000 -0.639039000000 -0.046900000000

C -0.394733000000 0.494057000000 -0.497751000000

C -1.372648000000 -0.438866000000 -0.612384000000

C -0.761074000000 -1.815150000000 -0.537953000000

C -1.109110000000 -2.812672000000 0.422174000000

C 0.223948000000 -2.147731000000 -1.533970000000

C -0.503670000000 1.952592000000 -0.602163000000

C 0.097732000000 2.639172000000 -1.685327000000

C -1.257242000000 2.701350000000 0.335223000000

O 2.864537000000 -1.926291000000 0.593991000000

C -0.087559000000 4.018162000000 -1.849620000000

H 0.699273000000 2.070495000000 -2.401336000000

C -1.433588000000 4.083641000000 0.172356000000

H -1.706612000000 2.184374000000 1.189293000000

C -0.855136000000 4.745449000000 -0.923035000000

H 0.369032000000 4.528678000000 -2.704256000000

H -2.028065000000 4.644153000000 0.901883000000

H -0.997893000000 5.823403000000 -1.052441000000

C -0.519695000000 -4.074618000000 0.379999000000

H -1.836740000000 -2.572674000000 1.203432000000

C 0.814669000000 -3.441454000000 -1.555371000000

H 0.364678000000 -1.474132000000 -2.386495000000

C 0.445211000000 -4.393126000000 -0.607981000000

H -0.799597000000 -4.827166000000 1.124379000000

H 1.543940000000 -3.682421000000 -2.335668000000

C 3.262610000000 1.735818000000 -0.149806000000

C 2.246051000000 0.669871000000 1.798126000000

C 2.757926000000 2.966469000000 0.265247000000

H 3.866073000000 1.647412000000 -1.059906000000

C 1.719369000000 1.918588000000 2.207159000000

H 2.136985000000 -0.195773000000 2.464265000000

C 1.986598000000 3.060154000000 1.449432000000

H 2.952642000000 3.870958000000 -0.321174000000

H 1.117598000000 1.987910000000 3.120049000000

O 4.356022000000 -0.895858000000 -1.081424000000

O 5.110219000000 -0.994841000000 1.169864000000

H 5.303211000000 -1.121373000000 -1.084714000000

H 3.018436000000 -2.613219000000 -0.084133000000

H 4.827679000000 -1.053223000000 2.097681000000

H 1.592621000000 4.031980000000 1.764127000000

H 0.890584000000 -5.392931000000 -0.628719000000

C -2.840381000000 -0.213491000000 -0.924530000000

H -2.971333000000 0.823932000000 -1.277465000000

H -3.154265000000 -0.885996000000 -1.746073000000

C -3.771086000000 -0.443592000000 0.271070000000

H -3.521452000000 0.227650000000 1.112058000000

H -3.733734000000 -1.479511000000 0.648208000000

O -5.123165000000 -0.160238000000 -0.183788000000

C -6.111641000000 -0.489964000000 0.703333000000

O -5.899842000000 -0.993103000000 1.797280000000

C -7.471712000000 -0.126809000000 0.139893000000

H -7.588337000000 0.970259000000 0.129075000000

H -7.571982000000 -0.477078000000 -0.899434000000

H -8.255875000000 -0.566941000000 0.769769000000

$^{2}TS_{HI}$

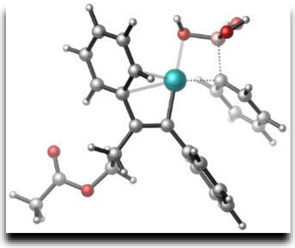

Frequency -126.1347

Zero-point correction= 0.429005 (Hartree/Particle)

Thermal correction to Energy= 0.460527

Thermal correction to Enthalpy= 0.461471

Thermal correction to Gibbs Free Energy= 0.362487

Sum of electronic and zero-point Energies= -1457.070070

Sum of electronic and thermal Energies= -1457.038548

Sum of electronic and thermal Enthalpies= -1457.037604

Sum of electronic and thermal Free Energies= -1457.136589

E(RM06L) = -1459.06978956

Atom	X	Y	Z
B	-3.532236000000	-1.440951000000	-0.363557000000
C	-2.695077000000	0.193228000000	-0.613155000000
Pd	-0.978419000000	-0.911891000000	0.127658000000
C	0.337374000000	0.518081000000	0.675463000000
C	1.501040000000	-0.181297000000	0.761567000000
C	1.267776000000	-1.658633000000	0.671377000000
C	1.995129000000	-2.516649000000	-0.206955000000
C	0.294982000000	-2.246981000000	1.552739000000
C	0.156593000000	1.971788000000	0.820187000000
C	0.002369000000	2.533849000000	2.111912000000
C	0.178083000000	2.844082000000	-0.294792000000
O	-2.379529000000	-2.377764000000	-0.685278000000
C	-0.107104000000	3.920742000000	2.281905000000
H	-0.024513000000	1.866730000000	2.979899000000
C	0.073113000000	4.231944000000	-0.119118000000
H	0.270480000000	2.421187000000	-1.299872000000
C	-0.068140000000	4.776257000000	1.167643000000
H	-0.218343000000	4.336171000000	3.289415000000
H	0.100740000000	4.891282000000	-0.993453000000
H	-0.150248000000	5.859941000000	1.301862000000
C	1.727908000000	-3.883684000000	-0.237300000000
H	2.785867000000	-2.094464000000	-0.836849000000
C	0.037856000000	-3.644099000000	1.497299000000
H	-0.082806000000	-1.661373000000	2.398693000000
C	0.734789000000	-4.450531000000	0.599943000000
H	2.296093000000	-4.529230000000	-0.915328000000
H	-0.686987000000	-4.081072000000	2.192591000000
C	-3.206242000000	1.217415000000	0.236313000000
C	-2.354503000000	0.567830000000	-1.950746000000
C	-3.432375000000	2.514358000000	-0.233920000000

H -3.473886000000 0.942319000000 1.261961000000

C -2.569041000000 1.869227000000 -2.426045000000

H -1.956975000000 -0.193891000000 -2.633234000000

C -3.113450000000 2.840442000000 -1.566496000000

H -3.853118000000 3.278805000000 0.428363000000

H -2.319496000000 2.130666000000 -3.460809000000

O -3.933732000000 -1.571233000000 1.004041000000

O -4.630263000000 -1.513464000000 -1.271056000000

H -4.907109000000 -1.585976000000 1.019605000000

H -2.449045000000 -3.119744000000 -0.052420000000

H -4.307999000000 -1.620036000000 -2.181455000000

H -3.291169000000 3.856663000000 -1.935866000000

H 0.543587000000 -5.528020000000 0.564601000000

C 2.911686000000 0.378319000000 0.823340000000

H 2.975742000000 1.185496000000 1.574082000000

H 3.609296000000 -0.422165000000 1.125381000000

C 3.349025000000 0.926624000000 -0.549760000000

H 2.857079000000 1.888478000000 -0.759079000000

H 3.113517000000 0.209637000000 -1.352205000000

O 4.776318000000 1.213527000000 -0.579779000000

C 5.574179000000 0.157182000000 -0.920441000000

O 5.157296000000 -0.964131000000 -1.185639000000

C 7.029869000000 0.578289000000 -0.942391000000

H 7.200478000000 1.288089000000 -1.769317000000

H 7.296949000000 1.096349000000 -0.007658000000

H 7.664581000000 -0.306295000000 -1.083130000000

^{2}I

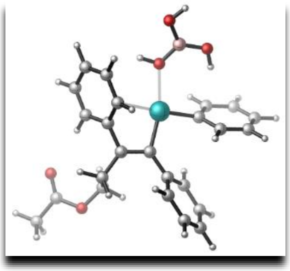

Zero-point correction= 0.431215 (Hartree/Particle)

Thermal correction to Energy= 0.463366

Thermal correction to Enthalpy= 0.464310

Thermal correction to Gibbs Free Energy= 0.362097

Sum of electronic and zero-point Energies= -1457.113714

Sum of electronic and thermal Energies= -1457.081562

Sum of electronic and thermal Enthalpies= -1457.080618

Sum of electronic and thermal Free Energies= -1457.182831

E(RM06L) = -1459.11304743

Atom X Y Z

B -3.863392000000 -2.206559000000 -0.146286000000

C -2.245630000000 1.022503000000 -0.496019000000

Pd -1.059063000000 -0.461870000000 0.133347000000

C 0.535566000000 0.645052000000 0.623880000000

C 1.547245000000 -0.270406000000 0.748289000000

C 1.110884000000 -1.700681000000 0.711335000000

C 1.718390000000 -2.666049000000 -0.142677000000

C 0.053165000000 -2.135167000000 1.570928000000

C 0.703777000000 2.105395000000 0.735309000000

C 1.146020000000 2.665796000000 1.960144000000

C 0.455394000000 2.981258000000 -0.349258000000

O -2.513414000000 -2.147349000000 -0.544500000000

C 1.349463000000 4.047735000000 2.089532000000

H 1.320415000000 2.000631000000 2.813059000000

C 0.671820000000 4.359769000000 -0.220577000000

H 0.084590000000 2.565092000000 -1.290726000000

C 1.118510000000 4.900399000000 0.997896000000

H 1.688214000000 4.458789000000 3.046852000000

H 0.481442000000 5.018221000000 -1.074958000000

H 1.276980000000 5.979356000000 1.097386000000

C 1.233690000000 -3.974238000000 -0.196698000000

H 2.579437000000 -2.377549000000 -0.756444000000

C -0.428035000000 -3.466235000000 1.504067000000

H -0.289875000000 -1.474071000000 2.374480000000

C 0.140720000000 -4.376640000000 0.605833000000

H 1.707151000000 -4.696479000000 -0.870166000000

H -1.228127000000 -3.783123000000 2.181810000000

C -2.670725000000 2.077705000000 0.340896000000

C -2.796255000000 0.930720000000 -1.798487000000

C -3.636200000000 2.999025000000 -0.101388000000

H -2.241899000000 2.188226000000 1.341733000000

C -3.755246000000 1.866736000000 -2.242835000000

H -2.473675000000 0.132395000000 -2.476568000000

C -4.180737000000 2.899286000000 -1.393095000000

H -3.962355000000 3.802247000000 0.569508000000

H -4.162628000000 1.782916000000 -3.257135000000

O -4.283920000000 -3.373819000000 0.454864000000

O -4.711632000000 -1.163225000000 -0.366842000000

H -5.229214000000 -3.311499000000 0.678753000000

H -2.032700000000 -2.959192000000 -0.277629000000

H -4.251856000000 -0.386529000000 -0.758005000000

H -4.927878000000 3.623130000000 -1.735464000000

H -0.226079000000 -5.407273000000 0.558267000000

C 3.037712000000 0.045053000000 0.730267000000

H 3.282835000000 0.842641000000 1.451824000000

H 3.608555000000 -0.854865000000 1.016638000000

C 3.478788000000 0.489418000000 -0.678002000000

H 3.112159000000 1.502948000000 -0.898646000000

H 3.110844000000 -0.209961000000 -1.445281000000

O 4.929960000000 0.577212000000 -0.780506000000

C 5.561959000000 -0.591002000000 -1.096912000000

O 4.988673000000 -1.658420000000 -1.283856000000

C 7.058144000000 -0.373049000000 -1.204526000000

H 7.280407000000 0.259682000000 -2.080317000000

H 7.437299000000 0.155573000000 -0.315617000000

H 7.561936000000 -1.341985000000 -1.316562000000

^{2}J

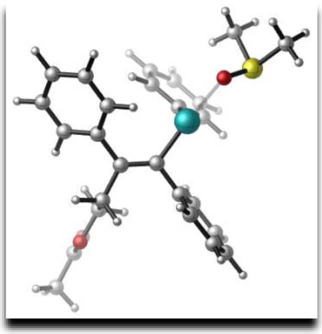

Zero-point correction= 0.459759 (Hartree/Particle)

Thermal correction to Energy= 0.493936

Thermal correction to Enthalpy= 0.494881

Thermal correction to Gibbs Free Energy= 0.385824

Sum of electronic and zero-point Energies= -1757.817723

Sum of electronic and thermal Energies= -1757.783545

Sum of electronic and thermal Enthalpies= -1757.782601

Sum of electronic and thermal Free Energies= -1757.891658

E(RM06L) = -1759.81194468

Atom X Y Z

C -1.339152000000 0.264896000000 1.154871000000

C 0.554372000000 -0.367320000000 -0.835346000000

C 1.443788000000 0.655908000000 -1.026769000000

C 1.038429000000 2.082622000000 -1.152777000000

C 1.863649000000 3.127351000000 -0.665624000000

C -0.162352000000 2.461427000000 -1.805270000000

C 0.940023000000 -1.803272000000 -0.793210000000

Pd -1.428793000000 -0.271068000000 -0.763572000000

C 1.077481000000 -2.537757000000 -2.000142000000

C 1.151449000000 -2.491679000000 0.429446000000

O -3.643704000000 -0.306323000000 -1.036389000000

C 1.406549000000 -3.901453000000 -1.983812000000

H 0.926108000000 -2.019837000000 -2.954169000000

C 1.483828000000 -3.853523000000 0.440775000000

H 1.042854000000 -1.942751000000 1.370555000000

S -4.444365000000 -0.961968000000 0.131823000000

C 1.610671000000 -4.567211000000 -0.763971000000

H 1.508303000000 -4.445034000000 -2.929801000000

H 1.648122000000 -4.361207000000 1.397828000000

H 1.869176000000 -5.631223000000 -0.751401000000

C 1.487999000000 4.473353000000 -0.781635000000

H 2.806195000000 2.885284000000 -0.164676000000

C -0.542370000000 3.806390000000 -1.920181000000

H -0.793731000000 1.677482000000 -2.241554000000

C 0.279104000000 4.822796000000 -1.404898000000

H 2.143962000000 5.252761000000 -0.378628000000

H -1.475414000000 4.061051000000 -2.435133000000

C -4.976701000000 0.414081000000 1.222714000000

C -6.077353000000 -1.317569000000 -0.625255000000

H -5.452326000000 1.198362000000 0.613409000000

H -5.671401000000 0.018198000000 1.981201000000

H -4.065109000000 0.799518000000 1.703203000000

H -6.771429000000 -1.670040000000 0.154281000000

H -6.455384000000 -0.406083000000 -1.113380000000

H -5.917092000000 -2.108344000000 -1.372331000000

C -1.471425000000 -0.727519000000 2.150285000000

C -1.321314000000 1.623797000000 1.530429000000

C -1.618039000000 -0.360678000000 3.502986000000

H -1.455919000000 -1.787799000000 1.876373000000

C -1.470225000000 1.981171000000 2.884750000000

H -1.187518000000 2.403172000000 0.773933000000

C -1.622593000000 0.993646000000 3.873142000000

H -1.720821000000 -1.141084000000 4.266208000000

H -1.456608000000 3.040903000000 3.165217000000

H -1.730150000000 1.278059000000 4.925426000000

H -0.011584000000 5.874237000000 -1.501578000000

C 2.947283000000 0.357520000000 -1.118878000000

H 3.108193000000 -0.578442000000 -1.680508000000

H 3.462950000000 1.161558000000 -1.672417000000

C 3.596775000000 0.200753000000 0.260770000000

H 3.469875000000 1.092981000000 0.897689000000

H 3.174131000000 -0.657672000000 0.808557000000

O 5.021457000000 -0.027975000000 0.042583000000

C 5.768010000000 -0.146971000000 1.178253000000

O 5.312405000000 -0.051744000000 2.309614000000

C 7.218095000000 -0.436470000000 0.834013000000

H 7.324482000000 -1.500589000000 0.561197000000

H 7.549260000000 0.161308000000 -0.028976000000

H 7.848036000000 -0.231537000000 1.710153000000

$^{2}TS_{JK}$

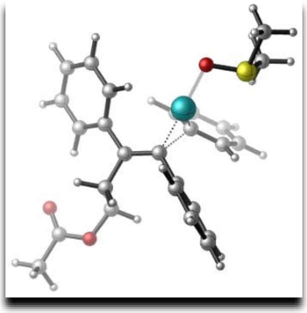

Frequency -273.5638

Zero-point correction= 0.459935 (Hartree/Particle)

Thermal correction to Energy= 0.492908

Thermal correction to Enthalpy= 0.493852

Thermal correction to Gibbs Free Energy= 0.390845

Sum of electronic and zero-point Energies= -1757.805987

Sum of electronic and thermal Energies= -1757.773015

Sum of electronic and thermal Enthalpies= -1757.772071

Sum of electronic and thermal Free Energies= -1757.875078

E(RM06L) = -1759.79335862

**Atom**	**X**	**Y**	**Z**
C	-0.813403000000	0.116727000000	1.311521000000
C	0.284837000000	-0.437071000000	-0.288189000000
C	1.392109000000	0.326411000000	-0.617989000000
C	1.398383000000	1.810988000000	-0.689738000000
C	2.538879000000	2.555373000000	-0.281872000000
C	0.297997000000	2.543078000000	-1.205709000000
C	0.335707000000	-1.930556000000	-0.405822000000
Pd	-1.633297000000	0.226926000000	-0.558874000000
C	0.019048000000	-2.528849000000	-1.652200000000
C	0.704686000000	-2.786567000000	0.659433000000
O	-3.633510000000	0.701229000000	-1.336630000000
C	0.071095000000	-3.918863000000	-1.825091000000
H	-0.268754000000	-1.879257000000	-2.486079000000
C	0.751425000000	-4.180430000000	0.486847000000
H	0.943732000000	-2.351776000000	1.634814000000
S	-4.625442000000	-0.304727000000	-0.663637000000
C	0.435004000000	-4.753698000000	-0.753838000000
H	-0.172475000000	-4.352464000000	-2.801341000000
H	1.040976000000	-4.818728000000	1.329023000000
H	0.473016000000	-5.839989000000	-0.887331000000
C	2.543535000000	3.956327000000	-0.327370000000
H	3.436983000000	2.042151000000	0.077613000000
C	0.301087000000	3.944705000000	-1.242970000000
H	-0.564209000000	1.986556000000	-1.603389000000
C	1.422382000000	4.661642000000	-0.796091000000
H	3.434362000000	4.499095000000	0.006813000000
H	-0.568142000000	4.474837000000	-1.647590000000
C	-4.877998000000	0.284288000000	1.056095000000
C	-6.264253000000	0.219309000000	-1.325892000000
H	-5.128733000000	1.356380000000	1.040524000000
H	-5.682836000000	-0.309055000000	1.519872000000
H	-3.924690000000	0.120431000000	1.582271000000
H	-7.060423000000	-0.352498000000	-0.823271000000
H	-6.387193000000	1.301563000000	-1.166027000000
H	-6.259332000000	-0.006977000000	-2.401954000000
C	-1.315205000000	-0.955975000000	2.094887000000
C	-0.430752000000	1.308289000000	1.979025000000
C	-1.442919000000	-0.833115000000	3.491257000000
H	-1.593547000000	-1.896284000000	1.609668000000
C	-0.550717000000	1.416779000000	3.372276000000
H	-0.036506000000	2.150692000000	1.405724000000
C	-1.059321000000	0.351701000000	4.138151000000
H	-1.842863000000	-1.673603000000	4.070848000000
H	-0.245428000000	2.347827000000	3.863675000000
H	-1.150086000000	0.444792000000	5.225591000000
H	1.432659000000	5.756229000000	-0.835717000000
C	2.738491000000	-0.352156000000	-0.901253000000
H	2.590571000000	-1.273343000000	-1.489201000000
H	3.371275000000	0.325477000000	-1.498424000000
C	3.494820000000	-0.715944000000	0.391261000000
H	3.577269000000	0.146457000000	1.070812000000
H	3.000947000000	-1.549899000000	0.910155000000
O	4.835049000000	-1.210348000000	0.091035000000
C	5.813690000000	-0.263196000000	0.016284000000
O	5.634500000000	0.936285000000	0.192432000000
C	7.147519000000	-0.906808000000	-0.310967000000
H	7.099564000000	-1.389435000000	-1.301226000000
H	7.933279000000	-0.140186000000	-0.309909000000
H	7.384272000000	-1.693610000000	0.423479000000

^{2}K

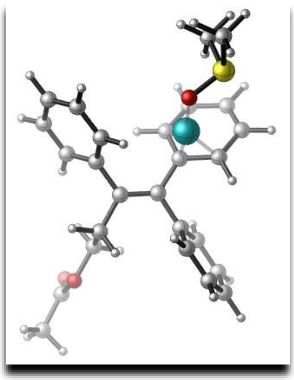

Zero-point correction= 0.460426 (Hartree/Particle)

Thermal correction to Energy= 0.494305

Thermal correction to Enthalpy= 0.495250

Thermal correction to Gibbs Free Energy= 0.386653

Sum of electronic and zero-point Energies= -1757.839714

Sum of electronic and thermal Energies= -1757.805834

Sum of electronic and thermal Enthalpies= -1757.804890

Sum of electronic and thermal Free Energies= -1757.913486

E(RM06L) = -1759.83301285

**Atom**	**X**	**Y**	**Z**
C	-0.353198000000	-0.591007000000	0.998637000000
C	0.868953000000	-0.486820000000	0.120819000000
C	1.449152000000	0.686007000000	-0.299534000000
C	0.792541000000	2.022535000000	-0.169761000000
C	1.411386000000	3.095071000000	0.514457000000
C	-0.437621000000	2.277414000000	-0.823383000000
C	1.508387000000	-1.806704000000	-0.215176000000
Pd	-2.019835000000	-0.283828000000	-0.326963000000
C	1.474634000000	-2.311189000000	-1.534097000000
C	2.118988000000	-2.588938000000	0.790559000000
O	-3.761423000000	0.225192000000	-1.582378000000
C	2.051281000000	-3.553951000000	-1.841985000000
H	0.980564000000	-1.721436000000	-2.314014000000
C	2.702174000000	-3.827128000000	0.481435000000
H	2.130926000000	-2.218571000000	1.821510000000
S	-4.938417000000	-0.394921000000	-0.762858000000
C	2.670673000000	-4.313403000000	-0.836296000000
H	2.012198000000	-3.930644000000	-2.869817000000
H	3.179845000000	-4.414915000000	1.272655000000
H	3.121675000000	-5.281943000000	-1.076642000000
C	0.806117000000	4.359967000000	0.577071000000
H	2.366858000000	2.931790000000	1.024139000000
C	-1.039998000000	3.543576000000	-0.767824000000
H	-0.901998000000	1.468567000000	-1.410243000000
C	-0.424044000000	4.588892000000	-0.060994000000
H	1.299188000000	5.168709000000	1.127258000000
H	-1.987580000000	3.712107000000	-1.290538000000
C	-5.164132000000	0.624126000000	0.751671000000
C	-6.437501000000	0.225426000000	-1.654339000000
H	-5.211424000000	1.689237000000	0.475864000000
H	-6.089020000000	0.301858000000	1.258558000000
H	-4.283742000000	0.429425000000	1.384069000000
H	-7.342111000000	-0.083928000000	-1.106883000000
H	-6.380456000000	1.321350000000	-1.742550000000
H	-6.425169000000	-0.236338000000	-2.652184000000
C	-1.159013000000	-1.802818000000	0.980272000000
C	-0.550586000000	0.301894000000	2.118878000000
C	-2.050568000000	-2.089593000000	2.061722000000
H	-0.888208000000	-2.618327000000	0.300624000000
C	-1.415232000000	-0.017355000000	3.161941000000
H	0.042187000000	1.219263000000	2.167588000000
C	-2.165454000000	-1.222029000000	3.145907000000
H	-2.606540000000	-3.033727000000	2.044795000000
H	-1.503395000000	0.668113000000	4.012165000000
H	-2.824058000000	-1.470062000000	3.985360000000
H	-0.892720000000	5.577777000000	-0.016657000000

C 2.824654000000 0.702050000000 -0.972533000000

H 2.926247000000 -0.147177000000 -1.668351000000

H 2.937589000000 1.626103000000 -1.564677000000

C 3.972527000000 0.620955000000 0.041592000000

H 3.975012000000 1.460698000000 0.756865000000

H 3.925141000000 -0.315758000000 0.623514000000

O 5.216510000000 0.646846000000 -0.716626000000

C 6.348206000000 0.819273000000 0.028008000000

O 6.352028000000 0.957296000000 1.243426000000

C 7.576567000000 0.795278000000 -0.862743000000

H 7.767223000000 -0.237192000000 -1.202109000000

H 7.423067000000 1.413357000000 -1.761090000000

H 8.445485000000 1.152652000000 -0.294372000000

^{2}L

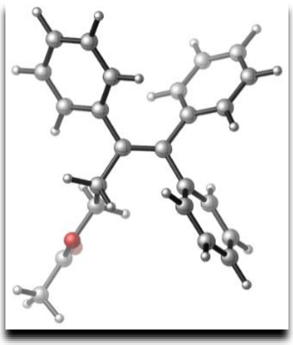

Zero-point correction= 0.383380 (Hartree/Particle)

Thermal correction to Energy= 0.407378

Thermal correction to Enthalpy= 0.408322

Thermal correction to Gibbs Free Energy= 0.325477

Sum of electronic and zero-point Energies= -1077.856641

Sum of electronic and thermal Energies= -1077.832643

Sum of electronic and thermal Enthalpies= -1077.831699

Sum of electronic and thermal Free Energies= -1077.914544

E(RM06L) = -1078.53425587

Atom X Y Z

C 1.938282000000 0.911202000000 0.371357000000

C 0.605371000000 0.646715000000 -0.252879000000

C 0.142408000000 -0.608008000000 -0.577225000000

C 1.014192000000 -1.818671000000 -0.611127000000

C 0.561350000000 -3.067222000000 -0.120125000000

C 2.289592000000 -1.775340000000 -1.225618000000

C -0.235760000000 1.872661000000 -0.463803000000

C -0.647479000000 2.256793000000 -1.760809000000

C -0.584829000000 2.704962000000 0.624463000000

C -1.404570000000 3.421445000000 -1.960422000000

H -0.354415000000 1.638780000000 -2.616842000000

C -1.350422000000 3.863279000000 0.426299000000

H -0.250314000000 2.433470000000 1.631381000000

C -1.764208000000 4.225262000000 -0.866591000000

H -1.708216000000 3.704291000000 -2.974074000000

H -1.621311000000 4.487643000000 1.284353000000

H -2.356715000000 5.132981000000 -1.021330000000

C 1.361850000000 -4.215536000000 -0.208691000000

H -0.424059000000 -3.140015000000 0.351916000000

C 3.084841000000 -2.923978000000 -1.323400000000

H 2.646966000000 -0.827474000000 -1.638538000000

C 2.628339000000 -4.150007000000 -0.810459000000

H 0.991363000000 -5.165126000000 0.192090000000

H 4.063716000000 -2.863219000000 -1.811013000000

C 2.718638000000 2.016072000000 -0.045127000000

C 2.421342000000 0.117972000000 1.439463000000

C 3.953969000000 2.293843000000 0.557210000000

H 2.348927000000 2.655925000000 -0.853212000000

C 3.650218000000 0.403490000000 2.048588000000

H 1.818159000000 -0.723171000000 1.793785000000

C 4.426058000000 1.488411000000 1.606469000000

H 4.546828000000 3.146661000000 0.209376000000

H 4.000058000000 -0.219777000000 2.878492000000

H	5.386373000000	1.710611000000	2.083533000000
H	3.250785000000	-5.047554000000	-0.887307000000
C	-1.332528000000	-0.841152000000	-0.916054000000
H	-1.711096000000	-0.058733000000	-1.592510000000
H	-1.451014000000	-1.805571000000	-1.437207000000
C	-2.206828000000	-0.837264000000	0.344719000000
H	-1.914874000000	-1.615538000000	1.070890000000
H	-2.153165000000	0.135583000000	0.862826000000
O	-3.577153000000	-1.076864000000	-0.083167000000
C	-4.473762000000	-1.265739000000	0.931349000000
O	-4.171736000000	-1.257890000000	2.116271000000
C	-5.872143000000	-1.457784000000	0.375620000000
H	-6.282905000000	-0.480096000000	0.070051000000
H	-5.860011000000	-2.104445000000	-0.515169000000
H	-6.517484000000	-1.885630000000	1.154443000000

N = 3

c3 = 5-Phenyl-4-pentynylacetate

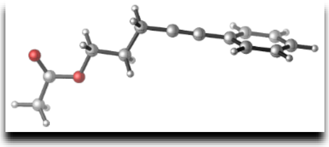

Zero-point correction= 0.230997 (Hartree/Particle)

Thermal correction to Energy= 0.246801

Thermal correction to Enthalpy= 0.247745

Thermal correction to Gibbs Free Energy= 0.182673

Sum of electronic and zero-point Energies= -653.982706

Sum of electronic and thermal Energies= -653.966902

Sum of electronic and thermal Enthalpies= -653.965958

Sum of electronic and thermal Free Energies= -654.031030

E(RM06L) = -654.403378785

**Atom**	**X**	**Y**	**Z**
H	-3.484549000000	-0.749642000000	2.037505000000
H	-5.874241000000	-0.043343000000	2.208668000000
H	-7.030337000000	0.964719000000	0.230308000000
H	0.864648000000	-1.717669000000	-1.324795000000
C	-3.276331000000	-0.145000000000	-0.037580000000
H	-5.782729000000	1.262621000000	-1.919721000000
H	-3.393099000000	0.555644000000	-2.089356000000
C	-3.993616000000	-0.308360000000	1.175342000000
C	-5.333250000000	0.089209000000	1.265745000000
C	-5.982767000000	0.655232000000	0.155530000000
C	-5.281984000000	0.822193000000	-1.051040000000
C	-3.942158000000	0.427715000000	-1.151595000000
C	-0.738592000000	-0.906111000000	-0.220343000000
C	-1.908678000000	-0.550085000000	-0.135259000000
C	0.665549000000	-1.304668000000	-0.315727000000
H	0.863687000000	-2.132332000000	0.394158000000
C	1.648180000000	-0.138529000000	-0.033747000000
H	1.460681000000	0.265833000000	0.975888000000
H	1.462807000000	0.681648000000	-0.748533000000
C	3.098960000000	-0.598485000000	-0.142553000000
H	3.338017000000	-0.982968000000	-1.150898000000
H	3.333588000000	-1.403778000000	0.577258000000
O	3.940616000000	0.551573000000	0.139783000000
C	5.285873000000	0.306350000000	0.080552000000
O	5.762478000000	-0.787554000000	-0.184714000000
C	6.067868000000	1.570523000000	0.379945000000
H	5.864506000000	2.332311000000	-0.391033000000
H	5.757136000000	1.995718000000	1.347965000000
H	7.140803000000	1.339200000000	0.396404000000

^{3}B

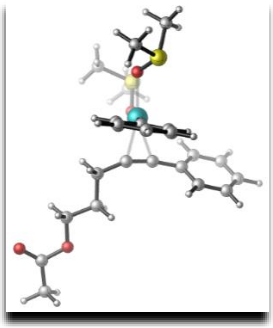

Zero-point correction= 0.477281 (Hartree/Particle)

Thermal correction to Energy= 0.515522

Thermal correction to Enthalpy= 0.516466

Thermal correction to Gibbs Free Energy= 0.396736

Sum of electronic and zero-point Energies= -2118.503068

Sum of electronic and thermal Energies= -2118.464827

Sum of electronic and thermal Enthalpies= -2118.463883

Sum of electronic and thermal Free Energies= -2118.583614

E(RM06L) = -2120.55688162

**Atom**	**X**	**Y**	**Z**
C	-1.188249000000	0.146460000000	-0.152402000000
C	0.818859000000	-1.884869000000	-0.377346000000
C	-0.648298000000	0.669459000000	-1.169408000000
C	-0.281332000000	1.257984000000	-2.424231000000
C	0.379387000000	2.513428000000	-2.463303000000
H	0.622778000000	3.006326000000	-1.517646000000
C	-0.600441000000	0.593279000000	-3.637200000000
H	-1.109440000000	-0.374170000000	-3.600878000000
C	0.702877000000	3.091753000000	-3.694923000000
H	1.206825000000	4.062827000000	-3.724675000000
C	0.377343000000	2.432126000000	-4.894376000000
H	0.630153000000	2.890860000000	-5.855356000000
C	-0.273643000000	1.186679000000	-4.862634000000
H	-0.528868000000	0.676779000000	-5.796622000000
C	-2.296593000000	-0.283195000000	0.725869000000
H	-2.175821000000	0.189051000000	1.719018000000
Pd	0.881470000000	-0.011299000000	0.287037000000
H	-2.213199000000	-1.374745000000	0.889647000000
O	0.866513000000	2.105757000000	1.096330000000
O	2.799045000000	-0.297674000000	1.233144000000
C	0.438968000000	-2.912006000000	0.508669000000
C	1.213346000000	-2.191593000000	-1.691737000000
C	0.477875000000	-4.253885000000	0.078414000000
H	0.114976000000	-2.680605000000	1.529895000000
C	1.254026000000	-3.536510000000	-2.110394000000
H	1.483469000000	-1.395651000000	-2.394571000000
C	0.888389000000	-4.565993000000	-1.228466000000
H	0.178787000000	-5.050675000000	0.768152000000
H	1.564807000000	-3.772769000000	-3.133820000000
H	0.911605000000	-5.608380000000	-1.561342000000
S	2.119382000000	2.670867000000	1.846240000000
C	2.050091000000	1.993470000000	3.548234000000
H	2.263654000000	0.918936000000	3.454419000000
H	2.822618000000	2.483374000000	4.162648000000
H	1.045918000000	2.157856000000	3.968496000000
C	1.630633000000	4.389959000000	2.235539000000
H	2.401115000000	4.845867000000	2.877371000000
H	1.569424000000	4.933452000000	1.281766000000
H	0.648844000000	4.383792000000	2.732858000000
S	3.887874000000	-1.162322000000	0.471676000000
C	5.449750000000	-0.414246000000	1.051053000000
H	5.461385000000	-0.385033000000	2.151136000000
H	6.289592000000	-1.012216000000	0.662957000000
H	5.493722000000	0.602599000000	0.635277000000
C	3.991268000000	-2.745034000000	1.379895000000
H	4.865088000000	-3.302338000000	1.005435000000
H	4.078899000000	-2.545346000000	2.458907000000
H	3.065861000000	-3.296199000000	1.156007000000
C	-3.683620000000	0.065475000000	0.133422000000

H -3.758028000000 1.154985000000 -0.025796000000

H -3.800478000000 -0.418406000000 -0.851533000000

C -4.800616000000 -0.394589000000 1.069277000000

H -4.730346000000 0.085689000000 2.062476000000

H -4.774295000000 -1.487600000000 1.234443000000

O -6.054636000000 -0.031263000000 0.446508000000

C -7.166139000000 -0.348027000000 1.194398000000

O -7.097091000000 -0.881520000000 2.290329000000

C -8.430616000000 0.043809000000 0.461421000000

H -8.376174000000 1.090407000000 0.122057000000

H -8.553735000000 -0.585720000000 -0.435872000000

H -9.293098000000 -0.095955000000 1.125686000000

$^{3}TS^{\alpha}_{BC}$

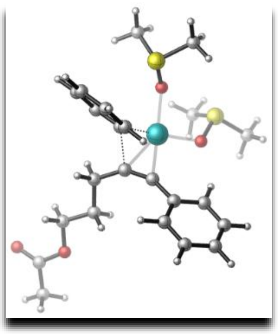

Frequency -249.7638

Zero-point correction= 0.476926 (Hartree/Particle)

Thermal correction to Energy= 0.514299

Thermal correction to Enthalpy= 0.515243

Thermal correction to Gibbs Free Energy= 0.398565

Sum of electronic and zero-point Energies= -2118.486343

Sum of electronic and thermal Energies= -2118.448969

Sum of electronic and thermal Enthalpies= -2118.448025

Sum of electronic and thermal Free Energies= -2118.564703

E(RM06L) = -2120.53926312

Atom X Y Z

C 1.056837000000 -0.332437000000 0.454381000000

C -0.471785000000 -1.584830000000 1.038461000000

C 0.723936000000 0.885343000000 0.759261000000

C 2.170112000000 -1.196040000000 -0.040233000000

Pd -1.021489000000 0.066342000000 -0.027836000000

C -0.497968000000 -2.834502000000 0.377079000000

C -0.635670000000 -1.525986000000 2.440833000000

C 0.982967000000 2.131086000000 1.427103000000

C 0.362760000000 3.342801000000 1.026644000000

C 1.904701000000 2.145174000000 2.511606000000

H -0.350733000000 3.320310000000 0.198475000000

C 0.671258000000 4.535769000000 1.688662000000

H 2.370310000000 1.207953000000 2.831442000000

C 2.208293000000 3.347102000000 3.158582000000

H 0.193677000000 5.469266000000 1.374518000000

C 1.593314000000 4.544292000000 2.750758000000

H 1.830997000000 5.482180000000 3.262272000000

H 2.922843000000 3.350534000000 3.987496000000

H 1.833737000000 -1.738198000000 -0.943170000000

H 2.381711000000 -1.976566000000 0.715741000000

C 3.440381000000 -0.377225000000 -0.355531000000

O -1.773830000000 1.971595000000 -0.912686000000

O -2.848360000000 -0.824653000000 -0.940707000000

S -3.034297000000 2.001108000000 -1.845824000000

S -3.721741000000 -1.742703000000 -0.005742000000

C -0.716216000000 -4.009874000000 1.116111000000

H -0.347191000000 -2.888531000000 -0.706231000000

C -0.871330000000 -2.706008000000 3.165117000000

H -0.567189000000 -0.565626000000 2.961114000000

C -0.909727000000 -3.947707000000 2.507241000000

H -0.725940000000 -4.977398000000 0.602579000000

H -1.005572000000 -2.654435000000 4.250589000000

H -1.071305000000 -4.866597000000 3.079212000000

C -2.516234000000 1.272395000000 -3.444998000000

C -3.096923000000 3.752560000000 -2.368901000000

H -2.388033000000 0.196123000000 -3.261297000000

H -3.311995000000 1.439777000000 -4.188691000000

H -1.568840000000 1.732069000000 -3.765919000000

H -3.895130000000 3.874009000000 -3.118298000000

H -3.331530000000 4.347305000000 -1.474336000000

H -2.118670000000 4.046940000000 -2.778289000000

C -5.297458000000 -0.834420000000 0.209420000000

C -4.329839000000 -3.059569000000 -1.118017000000

H -5.715072000000 -0.563825000000 -0.772142000000

H -5.995841000000 -1.471054000000 0.775892000000

H -5.065493000000 0.069376000000 0.791506000000

H -5.074328000000 -3.665691000000 -0.577613000000

H -4.766529000000 -2.607465000000 -2.021580000000

H -3.461657000000 -3.681684000000 -1.377743000000

H 3.219613000000 0.369816000000 -1.137393000000

H 3.763401000000 0.180506000000 0.540914000000

C 4.572995000000 -1.290854000000 -0.823271000000

H 4.304220000000 -1.848429000000 -1.738830000000

H 4.841363000000 -2.038840000000 -0.054306000000

O 5.717110000000 -0.446771000000 -1.092778000000

C 6.789361000000 -1.110066000000 -1.644935000000

O 6.772100000000 -2.304158000000 -1.898518000000

C 7.951856000000 -0.164177000000 -1.854764000000

H 7.610349000000 0.803804000000 -2.252267000000

H 8.446700000000 0.030024000000 -0.887666000000

H 8.677361000000 -0.626946000000 -2.536610000000

$^{3}C^{\alpha}$

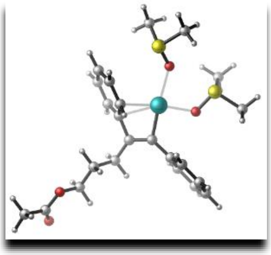

Zero-point correction= 0.479430 (Hartree/Particle)

Thermal correction to Energy= 0.516710

Thermal correction to Enthalpy= 0.517654

Thermal correction to Gibbs Free Energy= 0.400977

Sum of electronic and zero-point Energies= -2118.527927

Sum of electronic and thermal Energies= -2118.490647

Sum of electronic and thermal Enthalpies= -2118.489703

Sum of electronic and thermal Free Energies= -2118.606380

E(RM06L) = -2120.58209967

**Atom**	**X**	**Y**	**Z**
C	-1.460373000000	0.056897000000	0.490124000000
C	-0.870137000000	-1.323471000000	0.466379000000
C	-0.530186000000	0.936407000000	0.055057000000
C	-0.502273000000	2.389212000000	-0.087545000000
C	-0.724775000000	3.224133000000	1.037358000000
H	-0.881180000000	2.765789000000	2.019005000000
C	-0.276345000000	2.989358000000	-1.351960000000
H	-0.096953000000	2.348659000000	-2.221364000000
C	-0.753637000000	4.617310000000	0.891143000000
H	-0.941053000000	5.251416000000	1.763590000000
C	-0.545927000000	5.198915000000	-0.371357000000
H	-0.573444000000	6.287434000000	-0.483788000000
C	-0.306084000000	4.381771000000	-1.491094000000
H	-0.150705000000	4.833966000000	-2.475948000000
C	-2.892879000000	0.318496000000	0.899174000000
H	-3.060317000000	1.408867000000	0.932562000000
Pd	1.144365000000	-0.160694000000	-0.072358000000

H -3.049868000000 -0.055484000000 1.931500000000

O 2.337447000000 1.607854000000 0.201430000000

O 3.136789000000 -1.265808000000 0.116098000000

C -0.891328000000 -2.205327000000 1.589029000000

C -0.303633000000 -1.794271000000 -0.773820000000

C -0.388308000000 -3.497649000000 1.479560000000

H -1.320761000000 -1.856851000000 2.533634000000

C 0.206969000000 -3.125036000000 -0.855238000000

H -0.534384000000 -1.254551000000 -1.699613000000

C 0.165934000000 -3.961702000000 0.256262000000

H -0.431496000000 -4.170617000000 2.342166000000

H 0.578066000000 -3.495255000000 -1.815997000000

H 0.517585000000 -4.995933000000 0.180090000000

S 3.898704000000 1.579791000000 -0.009678000000

C 4.619778000000 1.261055000000 1.642310000000

H 4.386961000000 0.211230000000 1.869931000000

H 5.710288000000 1.413408000000 1.595661000000

H 4.155244000000 1.934163000000 2.378989000000

C 4.273624000000 3.362796000000 -0.135237000000

H 5.366332000000 3.497194000000 -0.170337000000

H 3.817837000000 3.718663000000 -1.070095000000

H 3.832165000000 3.887389000000 0.725303000000

S 3.525202000000 -2.513075000000 -0.744820000000

C 5.162542000000 -2.118515000000 -1.466781000000

H 5.864538000000 -1.824740000000 -0.671561000000

H 5.530943000000 -3.003974000000 -2.009080000000

H 5.009790000000 -1.288832000000 -2.172078000000

C 4.078030000000 -3.771241000000 0.464629000000

H 4.491581000000 -4.635570000000 -0.079401000000

H 4.828506000000 -3.330386000000 1.138520000000

H 3.185649000000 -4.073838000000 1.030995000000

C -3.919794000000 -0.351514000000 -0.042952000000

H -3.791357000000 0.038110000000 -1.068652000000

H -3.741058000000 -1.441434000000 -0.087758000000

C -5.352561000000 -0.096839000000 0.420644000000

H -5.589467000000 0.982162000000 0.451720000000

H -5.535173000000 -0.498478000000 1.434405000000

O -6.233910000000 -0.755109000000 -0.522584000000

C -7.571958000000 -0.605051000000 -0.244555000000

O -7.985810000000 0.028474000000 0.713718000000

C -8.414274000000 -1.333306000000 -1.270616000000

H -8.122832000000 -1.040141000000 -2.291829000000

H -8.255409000000 -2.421355000000 -1.183972000000

H -9.473846000000 -1.104404000000 -1.098530000000

$^{3}TS^{\beta}_{BC}$

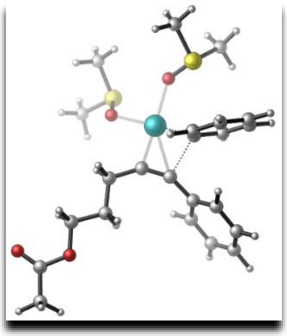

Frequency -247.5957

Zero-point correction= 0.476547 (Hartree/Particle)

Thermal correction to Energy= 0.514070

Thermal correction to Enthalpy= 0.515014

Thermal correction to Gibbs Free Energy= 0.398096

Sum of electronic and zero-point Energies= -2118.484038

Sum of electronic and thermal Energies= -2118.446514

Sum of electronic and thermal Enthalpies= -2118.445570

Sum of electronic and thermal Free Energies= -2118.562489

E(RM06L) = -2120.53232995

Atom X Y Z

C -0.630952000000 1.281182000000 0.120141000000

C 1.394667000000 1.435958000000 -0.522574000000

C -0.969513000000 2.537381000000 0.760199000000

C -1.631238000000 2.501857000000 2.014832000000

H -1.801541000000 1.536697000000 2.501516000000

C -0.726764000000 3.785876000000 0.137386000000

H -0.204742000000 3.813685000000 -0.823384000000

C -2.060317000000 3.691521000000 2.616710000000

H -2.573644000000 3.656202000000 3.582592000000

C -1.828456000000 4.924451000000 1.984184000000

H -2.162573000000 5.852929000000 2.457668000000

C -1.162928000000 4.967710000000 0.745450000000

H -0.984414000000 5.928638000000 0.253195000000

C -0.966147000000 0.068934000000 -0.182182000000

C -2.087613000000 -0.832688000000 -0.564897000000

H -2.030189000000 -1.745138000000 0.055779000000

Pd 0.957898000000 -0.500504000000 -0.037818000000

H -1.919800000000 -1.177186000000 -1.604161000000

O 0.417541000000 -2.614068000000 0.232358000000

O 3.075846000000 -1.202877000000 0.132805000000

C 1.396367000000 1.757857000000 -1.898875000000

C 2.199375000000 2.176728000000 0.376367000000

C 2.247384000000 2.770375000000 -2.378988000000

H 0.730764000000 1.226614000000 -2.586123000000

C 3.035801000000 3.192267000000 -0.111534000000

H 2.156076000000 1.967309000000 1.450303000000

C 3.064686000000 3.487058000000 -1.488446000000

H 2.254690000000 3.010819000000 -3.447348000000

H 3.655057000000 3.767589000000 0.585249000000

H 3.708153000000 4.289859000000 -1.862145000000

S 1.207752000000 -3.396512000000 1.341210000000

C 2.227475000000 -4.612225000000 0.431249000000

H 2.993296000000 -4.021453000000 -0.089735000000

H 2.698556000000 -5.300515000000 1.151077000000

H 1.597887000000 -5.156024000000 -0.289620000000

C -0.040741000000 -4.549290000000 2.017716000000

H 0.456338000000 -5.252456000000 2.704758000000

H -0.771859000000 -3.942452000000 2.571057000000

H -0.532812000000 -5.082354000000 1.189880000000

S 4.196870000000 -0.392498000000 -0.608083000000

C 5.494663000000 -0.127831000000 0.655367000000

H 5.728257000000 -1.082146000000 1.151805000000

H 6.386918000000 0.295117000000 0.166767000000

H 5.089976000000 0.592577000000 1.380136000000

C 5.078670000000 -1.639545000000 -1.619031000000

H 5.945413000000 -1.157858000000 -2.099209000000

H 5.399408000000 -2.481208000000 -0.986109000000

H 4.372488000000 -1.980713000000 -2.390074000000

C -3.474415000000 -0.161572000000 -0.444053000000

H -3.637905000000 0.185820000000 0.590989000000

H -3.519275000000 0.729189000000 -1.094462000000

C -4.580525000000 -1.141503000000 -0.832462000000

H -4.584575000000 -2.038411000000 -0.186925000000

H -4.468086000000 -1.492683000000 -1.875251000000

O -5.841990000000 -0.445574000000 -0.694687000000

C -6.948575000000 -1.245490000000 -0.864817000000

O -6.874180000000 -2.442824000000 -1.092973000000

C -8.219506000000 -0.431238000000 -0.756477000000

H -9.065408000000 -1.103587000000 -0.560958000000

H -8.140219000000 0.332273000000 0.031996000000

H -8.399622000000 0.092308000000 -1.711264000000

$^{3}TS_{BD}$

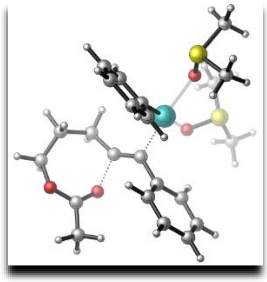

Frequency -240.6210

Zero-point correction= 0.477719 (Hartree/Particle)

Thermal correction to Energy= 0.514205

Thermal correction to Enthalpy= 0.515149

Thermal correction to Gibbs Free Energy= 0.403013

Sum of electronic and zero-point Energies= -2118.476262

Sum of electronic and thermal Energies= -2118.439776

Sum of electronic and thermal Enthalpies= -2118.438832

Sum of electronic and thermal Free Energies= -2118.550968

E(RM06L) = -2120.51747672

**Atom**	**X**	**Y**	**Z**
C	1.839479000000	0.256587000000	-1.173371000000
O	3.482736000000	0.968778000000	-1.024407000000
Pd	-0.798003000000	0.015204000000	-0.266712000000
C	-0.323119000000	-1.839704000000	0.282337000000
O	-1.293984000000	2.080327000000	-1.021338000000
O	-2.969403000000	-0.359919000000	-0.314816000000
C	-0.419002000000	-2.890046000000	-0.656491000000
C	0.028480000000	-2.144208000000	1.614535000000
C	-0.182262000000	-4.221789000000	-0.263754000000
H	-0.697182000000	-2.680532000000	-1.696467000000
C	0.255900000000	-3.478465000000	2.005061000000
H	0.141677000000	-1.339595000000	2.350789000000
C	0.152647000000	-4.518893000000	1.067479000000
H	-0.265677000000	-5.027067000000	-1.002419000000
H	0.522166000000	-3.700569000000	3.044732000000
H	0.335721000000	-5.554665000000	1.371093000000
S	-2.712567000000	2.666449000000	-0.725805000000
C	-3.741797000000	2.253697000000	-2.185745000000
H	-3.878140000000	1.163708000000	-2.151440000000
H	-4.713437000000	2.766793000000	-2.101632000000
H	-3.212705000000	2.552112000000	-3.103977000000
C	-2.508114000000	4.454496000000	-1.063527000000
H	-3.493652000000	4.945064000000	-1.023812000000
H	-1.857723000000	4.860166000000	-0.275368000000
H	-2.037496000000	4.585375000000	-2.049952000000
S	-3.536291000000	-1.637594000000	0.410001000000
C	-3.596775000000	-1.226965000000	2.193447000000
H	-4.060728000000	-0.238132000000	2.329197000000
H	-4.163375000000	-2.012667000000	2.718481000000
H	-2.553932000000	-1.221645000000	2.541882000000
C	-5.327625000000	-1.528497000000	0.058488000000
H	-5.845671000000	-2.327514000000	0.612143000000
H	-5.705040000000	-0.536723000000	0.350681000000
H	-5.454160000000	-1.686238000000	-1.022415000000
C	1.151701000000	0.543858000000	-0.098581000000
C	1.542609000000	1.219811000000	1.153794000000
C	1.161315000000	2.558752000000	1.400132000000
H	0.568407000000	3.082523000000	0.644655000000
C	2.293247000000	0.527877000000	2.134006000000
H	2.568631000000	-0.517391000000	1.959302000000
C	1.543905000000	3.194723000000	2.589189000000
H	1.254115000000	4.236297000000	2.763322000000
C	2.294827000000	2.505390000000	3.557225000000
H	2.585730000000	3.004214000000	4.486916000000
C	2.663782000000	1.169909000000	3.327352000000
H	3.239003000000	0.621401000000	4.080863000000
C	1.833094000000	-0.418029000000	-2.490259000000
H	2.067692000000	0.311173000000	-3.289507000000
H	0.798689000000	-0.759142000000	-2.661320000000

C 2.796023000000 -1.621029000000 -2.572110000000

H 2.611493000000 -2.151710000000 -3.524945000000

H 2.584564000000 -2.339943000000 -1.761891000000

C 4.273378000000 -1.249043000000 -2.585890000000

H 4.897982000000 -2.110419000000 -2.861265000000

H 4.472712000000 -0.419504000000 -3.287305000000

O 4.858797000000 -0.850807000000 -1.285898000000

C 4.508435000000 0.294847000000 -0.721270000000

C 5.468763000000 0.791534000000 0.319433000000

H 5.954973000000 1.706440000000 -0.062476000000

H 4.908145000000 1.070344000000 1.225175000000

H 6.238559000000 0.040891000000 0.541367000000

^{3}D

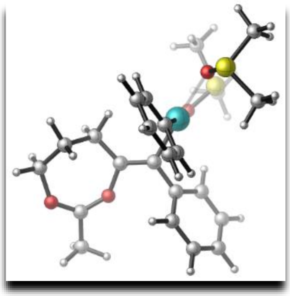

Zero-point correction= 0.479493 (Hartree/Particle)

Thermal correction to Energy= 0.515898

Thermal correction to Enthalpy= 0.516842

Thermal correction to Gibbs Free Energy= 0.405897

Sum of electronic and zero-point Energies= -2118.478294

Sum of electronic and thermal Energies= -2118.441889

Sum of electronic and thermal Enthalpies= -2118.440945

Sum of electronic and thermal Free Energies= -2118.551890

E(RM06L) = -2120.52082555

Atom X Y Z

C -2.089608000000 -0.182293000000 -0.841693000000

O -3.413442000000 -0.697211000000 -0.479727000000

Pd 0.773323000000 -0.008101000000 -0.236988000000

C 0.357940000000 1.888705000000 0.256193000000

O 1.212205000000 -2.110010000000 -0.954017000000

O 2.955733000000 0.342544000000 -0.501091000000

C 0.622025000000 2.903187000000 -0.693650000000

C -0.098598000000 2.269128000000 1.537394000000

C 0.445679000000 4.260878000000 -0.364276000000

H 0.986022000000 2.640306000000 -1.694121000000

C -0.265422000000 3.627834000000 1.866623000000

H -0.335488000000 1.503860000000 2.285844000000

C 0.003766000000 4.627193000000 0.917217000000

H 0.660274000000 5.032082000000 -1.113017000000

H -0.612510000000 3.903050000000 2.869263000000

H -0.131381000000 5.683109000000 1.173356000000

S 2.693249000000 -2.611521000000 -0.972774000000

C 3.400212000000 -2.059556000000 -2.572041000000

H 3.480175000000 -0.965931000000 -2.495941000000

H 4.397004000000 -2.511552000000 -2.700922000000

H 2.723639000000 -2.348874000000 -3.390991000000

C 2.519295000000 -4.388712000000 -1.379850000000

H 3.518570000000 -4.821309000000 -1.546925000000

H 2.042287000000 -4.873232000000 -0.515773000000

H 1.884998000000 -4.496355000000 -2.272986000000

S 3.602393000000 1.487524000000 0.361150000000

C 3.489365000000 0.952488000000 2.110177000000

H 3.844868000000 -0.085162000000 2.203212000000

H 4.088742000000 1.640216000000 2.728417000000

H 2.426663000000 1.026344000000 2.383225000000

C 5.395325000000 1.207034000000 0.126448000000

H 5.950774000000 1.906662000000 0.770827000000

H 5.641470000000 0.162832000000 0.371506000000

H 5.620601000000 1.418785000000 -0.928843000000

C -1.137980000000 -0.453511000000 0.086233000000

C -1.437032000000 -1.097989000000 1.386523000000

C -0.797936000000 -2.309749000000 1.747291000000

H -0.092517000000 -2.756889000000 1.039910000000

C -2.336965000000 -0.508174000000 2.309288000000

H -2.813741000000 0.444900000000 2.057042000000

C -1.072309000000 -2.920266000000 2.977941000000

H -0.581646000000 -3.865049000000 3.235173000000

C -1.965748000000 -2.325194000000 3.886554000000

H -2.167308000000 -2.799270000000 4.852297000000

C -2.590805000000 -1.114044000000 3.550054000000

H -3.277051000000 -0.635168000000 4.256645000000

C -1.972211000000 0.470858000000 -2.180860000000

H -2.088274000000 -0.277683000000 -2.993943000000

H -0.933974000000 0.835642000000 -2.231181000000

C -2.964240000000 1.623261000000 -2.412579000000

H -2.637418000000 2.219309000000 -3.285132000000

H -2.985808000000 2.312508000000 -1.550313000000

C -4.356592000000 1.133574000000 -2.757244000000

H -5.047531000000 1.950399000000 -3.010218000000

H -4.333091000000 0.411005000000 -3.592770000000

O -5.090086000000 0.441766000000 -1.669194000000

C -4.629487000000 -0.421468000000 -0.796662000000

C -5.684706000000 -1.179871000000 -0.050314000000

H -5.988929000000 -2.063062000000 -0.640741000000

H -5.286904000000 -1.522412000000 0.914621000000

H -6.574724000000 -0.549053000000 0.089550000000

^{3}E

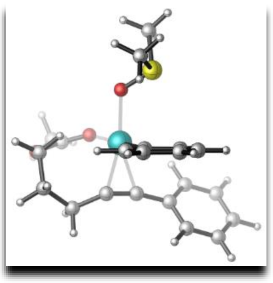

Zero-point correction= 0.399410 (Hartree/Particle)

Thermal correction to Energy= 0.429339

Thermal correction to Enthalpy= 0.430284

Thermal correction to Gibbs Free Energy= 0.333281

Sum of electronic and zero-point Energies= -1565.343380

Sum of electronic and thermal Energies= -1565.313450

Sum of electronic and thermal Enthalpies= -1565.312506

Sum of electronic and thermal Free Energies= -1565.409509

E(RM06L) = -1567.27394275

Atom X Y Z

C 0.392500000000 1.675917000000 1.171528000000

C -0.966989000000 -1.035869000000 0.936784000000

C -0.588918000000 1.832513000000 0.384080000000

C -1.793819000000 2.240056000000 -0.275843000000

C -1.850323000000 2.334202000000 -1.691056000000

H -0.966319000000 2.069644000000 -2.279342000000

C -2.935118000000 2.582512000000 0.496353000000

H -2.891549000000 2.496918000000 1.585721000000

C -3.023315000000 2.772314000000 -2.315229000000

H -3.062297000000 2.848842000000 -3.406089000000

C -4.146405000000 3.121086000000 -1.543372000000

H -5.060484000000 3.466943000000 -2.035875000000

C -4.098879000000 3.026867000000 -0.141222000000

H -4.974300000000 3.297118000000 0.457027000000

C 1.430753000000 2.210635000000 2.095304000000

H 0.909787000000 2.643814000000 2.969112000000

Pd 0.486656000000 -0.073195000000 -0.016916000000

H 1.919044000000 3.060584000000 1.581468000000

O 1.069637000000 -1.847061000000 -1.053465000000

C -0.667500000000 -1.672789000000 2.154661000000

C -2.244010000000 -1.151244000000 0.363141000000

C -1.656124000000 -2.449297000000 2.791195000000

H 0.322738000000 -1.579114000000 2.612257000000

C -3.223610000000 -1.933977000000 1.007422000000

H -2.490348000000 -0.637564000000 -0.571935000000

C -2.931910000000 -2.581803000000 2.217599000000

H -1.423244000000 -2.943168000000 3.740655000000

H -4.218362000000 -2.024697000000 0.558300000000

H -3.698512000000 -3.180985000000 2.718659000000

S 0.012562000000 -2.905165000000 -1.563996000000

C 0.890469000000 -3.638908000000 -2.986329000000

H 1.893431000000 -3.962606000000 -2.669775000000

H 0.299107000000 -4.486009000000 -3.368678000000

H 0.964833000000 -2.855819000000 -3.754432000000

C 0.116365000000 -4.318800000000 -0.409049000000

H -0.472329000000 -5.152615000000 -0.824334000000

H 1.170329000000 -4.605046000000 -0.271366000000

H -0.324033000000 -3.981327000000 0.540864000000

C 2.516848000000 1.240575000000 2.606273000000

H 3.213928000000 1.825752000000 3.230234000000

H 2.065898000000 0.477218000000 3.266339000000

C 3.305251000000 0.488967000000 1.529284000000

H 2.675639000000 -0.254829000000 1.007938000000

H 4.167652000000 -0.029886000000 1.976563000000

O 3.889627000000 1.395748000000 0.545602000000

C 3.335311000000 1.504984000000 -0.680727000000

O 2.282500000000 0.964484000000 -1.049886000000

C 4.181509000000 2.354019000000 -1.595642000000

H 4.718124000000 3.130502000000 -1.031818000000

H 4.935012000000 1.709063000000 -2.080661000000

H 3.552444000000 2.799733000000 -2.377725000000

$^{3}TS^{\alpha}_{EF}$

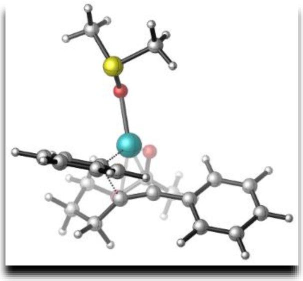

Frequency -337.0518

Zero-point correction= 0.398694 (Hartree/Particle)

Thermal correction to Energy= 0.428008

Thermal correction to Enthalpy= 0.428952

Thermal correction to Gibbs Free Energy= 0.334337

Sum of electronic and zero-point Energies= -1565.316726

Sum of electronic and thermal Energies= -1565.287412

Sum of electronic and thermal Enthalpies= -1565.286468

Sum of electronic and thermal Free Energies= -1565.381083

E(RM06L) = -1567.24238968

Atom X Y Z

C 0.716522000000 0.133862000000 1.529097000000

C -1.101907000000 0.880220000000 1.308008000000

C 1.408280000000 0.519396000000 0.487962000000

C 0.968683000000 -0.604447000000 2.812529000000

Pd -0.369847000000 -0.298625000000 -0.227813000000

C -2.140686000000 0.275530000000 2.056647000000

C -1.044326000000 2.291642000000 1.199558000000

C 2.367407000000 1.273128000000 -0.244749000000

C 2.726852000000 0.895814000000 -1.570745000000

C 2.987488000000 2.408817000000 0.354444000000

H 2.261530000000 0.007145000000 -2.009625000000

C 3.676162000000 1.641875000000 -2.273918000000

H 2.707702000000 2.698031000000 1.371841000000

C 3.944692000000 3.135157000000 -0.358807000000

H 3.951707000000 1.351088000000 -3.292180000000

C 4.286901000000 2.758577000000 -1.671461000000

H 5.034604000000 3.335111000000 -2.224820000000

H 4.424415000000 4.002330000000 0.105068000000

H 0.524778000000 -0.038159000000 3.650395000000

H 2.063432000000 -0.593991000000 2.953702000000

C 0.457572000000 -2.072142000000 2.895173000000

O -1.918467000000 -0.978697000000 -1.552731000000

O 1.241345000000 -2.220311000000 -0.971268000000

S -3.181500000000 -0.081408000000 -1.837783000000

C -3.107454000000 1.079559000000 2.683761000000

H -2.202632000000 -0.813463000000 2.142045000000

C -2.024618000000 3.079011000000 1.818101000000

H -0.223500000000 2.765415000000 0.652401000000

C -3.054975000000 2.477795000000 2.564340000000

H -3.905288000000 0.605650000000 3.264957000000

H -1.973711000000 4.169141000000 1.729968000000

H -3.808329000000 3.098619000000 3.058552000000

C -2.837204000000 0.734890000000 -3.438618000000

C -4.426906000000 -1.287834000000 -2.414433000000

H -2.522961000000 -0.018823000000 -4.176794000000

H -3.744989000000 1.264521000000 -3.769125000000

H -2.028968000000 1.459179000000 -3.260269000000

H -5.313829000000 -0.741018000000 -2.772001000000

H -3.989068000000 -1.907417000000 -3.211772000000

H -4.694706000000 -1.910483000000 -1.548746000000

H 1.020375000000 -2.564896000000 3.706782000000

H -0.601286000000 -2.081718000000 3.210498000000

C 0.524900000000 -2.924547000000 1.625564000000

H -0.140077000000 -2.535254000000 0.835084000000

H 0.226385000000 -3.960450000000 1.860183000000

O 1.879880000000 -3.006774000000 1.097473000000

C 2.092376000000 -2.687344000000 -0.207016000000

C 3.518394000000 -2.973338000000 -0.615053000000

H 3.511715000000 -3.704951000000 -1.439526000000

H 3.975259000000 -2.048789000000 -1.003135000000

H 4.113436000000 -3.365029000000 0.220364000000

$^{3}F^{\alpha}$

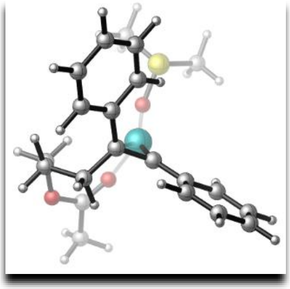

Zero-point correction= 0.401266 (Hartree/Particle)

Thermal correction to Energy= 0.430544

Thermal correction to Enthalpy= 0.431488

Thermal correction to Gibbs Free Energy= 0.335630

Sum of electronic and zero-point Energies= -1565.359843

Sum of electronic and thermal Energies= -1565.330565

Sum of electronic and thermal Enthalpies= -1565.329621

Sum of electronic and thermal Free Energies= -1565.425479

E(RM06L) = -1567.28533566

Atom X Y Z

C 1.158142000000 0.255296000000 0.937101000000

C 1.364002000000 1.729845000000 1.057541000000

C 1.265783000000 -0.381086000000 -0.316977000000

C 1.252008000000 -0.634149000000 2.188144000000

Pd -0.626724000000 -0.406827000000 -0.287194000000

C 1.935797000000 2.292211000000 2.225917000000

C 1.066921000000 2.597928000000 -0.023522000000

C 2.292184000000 -0.914808000000 -1.151910000000

C 1.970698000000 -1.632628000000 -2.341966000000

C 3.664507000000 -0.732339000000 -0.800794000000

H 0.916273000000 -1.763131000000 -2.605813000000

C 2.984757000000 -2.159147000000 -3.141315000000

H 3.912570000000 -0.171947000000 0.105600000000

C 4.671820000000 -1.250798000000 -1.615987000000

H 2.735412000000 -2.716220000000 -4.049173000000

C 4.334311000000 -1.965689000000 -2.781804000000

H 5.127840000000 -2.373173000000 -3.416223000000

H 5.722426000000 -1.104591000000 -1.348154000000

H 2.253540000000 -0.471956000000 2.634639000000

H 1.229347000000 -1.692533000000 1.877881000000

C 0.192641000000 -0.398560000000 3.291335000000

O -2.619666000000 0.166358000000 -1.036273000000

O -1.911532000000 -2.185246000000 0.589542000000

S -2.839005000000 1.527018000000 -1.781965000000

C 2.204207000000 3.666744000000 2.302908000000

H 2.201022000000 1.656505000000 3.075260000000

C 1.342659000000 3.967078000000 0.054437000000

H 0.593674000000 2.180169000000 -0.919173000000

C 1.910260000000 4.510214000000 1.220567000000

H 2.649232000000 4.077224000000 3.214700000000

H 1.105923000000 4.616611000000 -0.794290000000

H 2.116459000000 5.582854000000 1.286158000000

C -3.563374000000 1.058012000000 -3.395854000000

C -4.344392000000 2.230916000000 -1.014539000000

H -4.411500000000 0.376233000000 -3.229777000000

H -3.880476000000 1.970682000000 -3.925060000000

H -2.772367000000 0.550843000000 -3.967087000000

H -4.650656000000 3.125600000000 -1.579651000000

H -5.139073000000 1.469278000000 -1.009568000000

H -4.077500000000 2.512714000000 0.014285000000

H 0.513803000000 -0.941489000000 4.198320000000

H 0.155750000000 0.671964000000 3.560970000000

C -1.237989000000 -0.816895000000 2.954213000000

H -1.611919000000 -0.330426000000 2.036569000000

H -1.910978000000 -0.565769000000 3.790748000000

O -1.337011000000 -2.268109000000 2.816760000000

C -1.731889000000 -2.810847000000 1.642916000000

C -1.970349000000 -4.295023000000 1.773962000000

H -3.021175000000 -4.460694000000 2.069459000000

H -1.806095000000 -4.784105000000 0.804178000000

H -1.328680000000 -4.734584000000 2.551085000000

^{3}G

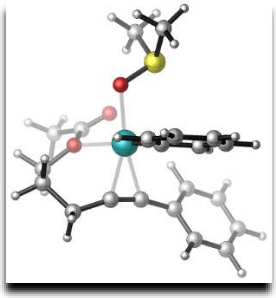

Zero-point correction= 0.399068 (Hartree/Particle)

Thermal correction to Energy= 0.429144

Thermal correction to Enthalpy= 0.430088

Thermal correction to Gibbs Free Energy= 0.333515

Sum of electronic and zero-point Energies= -1565.334920

Sum of electronic and thermal Energies= -1565.304843

Sum of electronic and thermal Enthalpies= -1565.303899

Sum of electronic and thermal Free Energies= -1565.400472

E(RM06L) = -1567.26833795

Atom	X	Y	Z
C	0.356209000000	1.460592000000	-1.681649000000
C	1.049685000000	-1.443064000000	-0.830270000000
C	1.049019000000	1.556154000000	-0.627343000000
C	2.037481000000	1.875558000000	0.361602000000

C 1.691725000000 1.946217000000 1.736192000000

H 0.661202000000 1.734704000000 2.039335000000

C 3.370765000000 2.145972000000 -0.043237000000

H 3.635688000000 2.078575000000 -1.102197000000

C 2.664106000000 2.290410000000 2.682095000000

H 2.394689000000 2.347244000000 3.741150000000

C 3.981417000000 2.566614000000 2.274034000000

H 4.737780000000 2.836831000000 3.017371000000

C 4.330267000000 2.494987000000 0.913843000000

H 5.356260000000 2.707230000000 0.598249000000

C -0.349130000000 1.898488000000 -2.907700000000

H 0.062187000000 1.364909000000 -3.783718000000

Pd -0.394598000000 -0.124568000000 -0.495034000000

H -0.126002000000 2.972694000000 -3.058622000000

O -1.654969000000 -1.705957000000 0.184459000000

C 0.951475000000 -2.266720000000 -1.962640000000

C 2.085408000000 -1.598053000000 0.101901000000

C 1.917221000000 -3.273676000000 -2.156813000000

H 0.137781000000 -2.146403000000 -2.684625000000

C 3.039686000000 -2.615658000000 -0.103850000000

H 2.168756000000 -0.941516000000 0.973938000000

C 2.957687000000 -3.448804000000 -1.229734000000

H 1.847720000000 -3.918989000000 -3.038957000000

H 3.851435000000 -2.742415000000 0.620351000000

H 3.706505000000 -4.230942000000 -1.388924000000

S -1.293507000000 -2.330917000000 1.588893000000

C -2.835115000000 -2.145695000000 2.548164000000

H -3.676272000000 -2.570286000000 1.979188000000

H -2.711777000000 -2.654027000000 3.517790000000

H -2.967622000000 -1.063739000000 2.695441000000

C -1.340326000000 -4.130825000000 1.286356000000

H -1.251707000000 -4.656133000000 2.250774000000

H -2.279835000000 -4.389970000000 0.774832000000

H -0.475856000000 -4.363602000000 0.648066000000

C -1.883568000000 1.676125000000 -2.865036000000

H -2.322875000000 2.127865000000 -3.771949000000

H -2.110490000000 0.595858000000 -2.905689000000

C -2.595790000000 2.279861000000 -1.655648000000

H -3.688873000000 2.249448000000 -1.800839000000

H -2.283806000000 3.325404000000 -1.482096000000

O -2.256391000000 1.479298000000 -0.472604000000

C -2.734131000000 1.785830000000 0.790169000000

O -2.284569000000 1.138630000000 1.722823000000

C -3.770147000000 2.878866000000 0.905969000000

H -3.370620000000 3.845813000000 0.555556000000

H -4.662326000000 2.649661000000 0.298922000000

H -4.060537000000 2.971721000000 1.960055000000

^{3}H

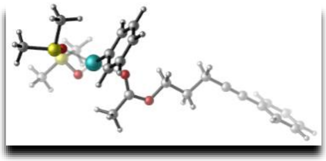

Zero-point correction= 0.478210 (Hartree/Particle)

Thermal correction to Energy= 0.516136

Thermal correction to Enthalpy= 0.517080

Thermal correction to Gibbs Free Energy= 0.398624

Sum of electronic and zero-point Energies= -2118.489566

Sum of electronic and thermal Energies= -2118.451641

Sum of electronic and thermal Enthalpies= -2118.450697

Sum of electronic and thermal Free Energies= -2118.569153

E(RM06L) = -2120.54560370

Atom X Y Z

H -8.593177000000 -1.884660000000 0.995243000000

H -11.048419000000 -1.823901000000 1.449547000000

H -12.461240000000 -0.006884000000 0.467372000000

H	-4.308136000000	0.432033000000	-1.843589000000
C	-8.607880000000	-0.100531000000	-0.244268000000
H	-11.408467000000	1.749178000000	-0.971285000000
H	-8.953049000000	1.690167000000	-1.424941000000
C	-9.214620000000	-1.091293000000	0.569170000000
C	-10.591454000000	-1.053015000000	0.820598000000
C	-11.385033000000	-0.032888000000	0.269040000000
C	-10.793559000000	0.953320000000	-0.538686000000
C	-9.417457000000	0.924844000000	-0.795861000000
C	-5.998192000000	-0.172765000000	-0.731999000000
C	-7.201794000000	-0.136562000000	-0.503320000000
C	-4.557289000000	-0.210987000000	-0.975946000000
H	-4.253653000000	-1.238241000000	-1.261520000000
C	-3.734589000000	0.245050000000	0.258798000000
H	-3.977432000000	-0.399480000000	1.120525000000
H	-4.020179000000	1.275263000000	0.531231000000
C	-2.239663000000	0.181308000000	-0.025082000000
H	-1.936858000000	0.842693000000	-0.855197000000
H	-1.892008000000	-0.840306000000	-0.254188000000
O	-1.546556000000	0.636579000000	1.193720000000
C	-0.219321000000	0.604279000000	1.196310000000
O	0.411471000000	0.219833000000	0.181064000000
C	0.401838000000	1.052712000000	2.487951000000
H	-0.357074000000	1.444402000000	3.178402000000
H	0.914226000000	0.193582000000	2.953397000000
H	1.163970000000	1.816874000000	2.265573000000
Pd	2.515599000000	0.134902000000	0.068462000000
C	2.370252000000	-1.828699000000	-0.115643000000
C	2.584534000000	-2.673419000000	0.990052000000
C	2.041558000000	-2.375502000000	-1.371007000000
C	2.477200000000	-4.070559000000	0.833954000000
C	1.941189000000	-3.772841000000	-1.518214000000
C	2.160408000000	-4.619911000000	-0.418004000000
H	2.832500000000	-2.257211000000	1.973008000000
H	1.859797000000	-1.723516000000	-2.232287000000
H	2.638747000000	-4.723917000000	1.698246000000
H	1.686402000000	-4.195492000000	-2.496247000000
C	4.025247000000	4.594666000000	0.483034000000
S	3.466443000000	3.215198000000	-0.582294000000
O	2.542605000000	2.361274000000	0.345889000000
C	2.331346000000	4.172551000000	-1.657330000000
H	4.686389000000	4.162210000000	1.247668000000
H	4.584204000000	5.318878000000	-0.130625000000
H	3.151319000000	5.067443000000	0.956930000000
H	2.919612000000	4.893697000000	-2.247089000000
H	1.846338000000	3.450270000000	-2.330088000000
H	1.578934000000	4.685199000000	-1.038423000000
C	5.774741000000	-2.171439000000	-1.110975000000
S	5.443375000000	-1.096907000000	0.330884000000
O	4.583012000000	0.118266000000	-0.223202000000
C	7.086304000000	-0.314414000000	0.474973000000
H	6.139868000000	-1.558494000000	-1.949170000000
H	6.519111000000	-2.928134000000	-0.814575000000
H	4.820685000000	-2.655825000000	-1.366266000000
H	7.827226000000	-1.092709000000	0.716648000000
H	7.334144000000	0.189843000000	-0.471234000000
H	7.027568000000	0.412703000000	1.297391000000
H	2.077145000000	-5.704881000000	-0.536199000000

^{3}I

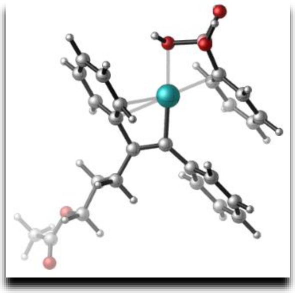

Zero-point correction= 0.456955 (Hartree/Particle)

Thermal correction to Energy= 0.490662

Thermal correction to Enthalpy= 0.491606

Thermal correction to Gibbs Free Energy= 0.385976

Sum of electronic and zero-point Energies= -1496.356867

Sum of electronic and thermal Energies= -1496.323160

Sum of electronic and thermal Enthalpies= -1496.322216

Sum of electronic and thermal Free Energies= -1496.427846

E(RM06L) = -1498.40321874

**Atom**	**X**	**Y**	**Z**
B	4.218179000000	-0.987968000000	0.375797000000
C	3.390103000000	0.427995000000	0.661796000000
Pd	1.501263000000	-0.662474000000	-0.023767000000
C	-0.059819000000	0.542891000000	-0.505793000000
C	-1.069238000000	-0.350105000000	-0.652564000000
C	-0.509179000000	-1.749114000000	-0.567061000000
C	-0.897890000000	-2.725188000000	0.400672000000
C	0.468725000000	-2.126486000000	-1.553199000000
C	-0.110259000000	2.006101000000	-0.590736000000
C	0.537359000000	2.691027000000	-1.648029000000
C	-0.856705000000	2.764490000000	0.345133000000
O	3.060854000000	-2.030815000000	0.644158000000
C	0.405542000000	4.079072000000	-1.788038000000
H	1.132242000000	2.116648000000	-2.365005000000
C	-0.980288000000	4.154898000000	0.206130000000
H	-1.344496000000	2.246412000000	1.177203000000
C	-0.354662000000	4.816281000000	-0.863551000000
H	0.897726000000	4.588728000000	-2.623237000000
H	-1.570460000000	4.722645000000	0.933627000000
H	-0.456087000000	5.900915000000	-0.974821000000
C	-0.358502000000	-4.009759000000	0.369850000000
H	-1.615136000000	-2.447855000000	1.178670000000
C	1.007814000000	-3.442209000000	-1.563911000000
H	0.649781000000	-1.458667000000	-2.402331000000
C	0.595384000000	-4.373064000000	-0.613029000000
H	-0.667788000000	-4.744857000000	1.120082000000
H	1.733436000000	-3.715827000000	-2.336757000000
C	3.659000000000	1.597312000000	-0.115437000000
C	2.559407000000	0.605561000000	1.825925000000
C	3.214043000000	2.857051000000	0.280489000000
H	4.270081000000	1.467499000000	-1.015413000000
C	2.091962000000	1.884182000000	2.214500000000
H	2.396315000000	-0.246451000000	2.498528000000
C	2.430619000000	3.002794000000	1.451299000000
H	3.464607000000	3.744073000000	-0.311459000000
H	1.479663000000	1.993890000000	3.116366000000
O	4.632626000000	-1.086034000000	-1.008988000000
O	5.341617000000	-1.216479000000	1.255713000000
H	5.565838000000	-1.363410000000	-0.994955000000
H	3.186138000000	-2.727248000000	-0.030144000000
H	5.038522000000	-1.263093000000	2.177696000000
H	2.081915000000	3.996688000000	1.750182000000
H	1.001374000000	-5.389679000000	-0.625355000000
C	-2.516170000000	-0.070240000000	-1.010475000000
H	-2.601473000000	1.002124000000	-1.262442000000
H	-2.772145000000	-0.636910000000	-1.929337000000
C	-3.546647000000	-0.419855000000	0.088157000000
H	-3.300629000000	0.121255000000	1.019702000000
H	-3.510793000000	-1.499031000000	0.321126000000

C -4.963788000000   -0.061741000000 -0.351800000000

H -5.252645000000   -0.588705000000 -1.279127000000

H -5.075513000000   1.020787000000 -0.543142000000

O -5.868247000000   -0.451903000000   0.718139000000

C -7.193126000000   -0.227735000000   0.458883000000

O -7.605163000000   0.265379000000 -0.580918000000

C -8.046601000000   -0.683599000000   1.626726000000

H -7.958842000000   -1.775477000000   1.754809000000

H -7.701881000000   -0.216514000000   2.563354000000

H -9.094725000000   -0.418844000000   1.436002000000

$^{3}TS_{IJ}$

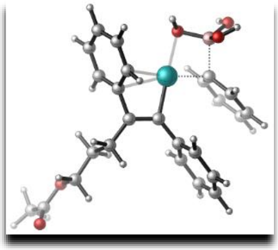

Frequency   -140.7531

Zero-point correction=   0.456066 (Hartree/Particle)

Thermal correction to Energy=   0.489362

Thermal correction to Enthalpy=   0.490306

Thermal correction to Gibbs Free Energy=   0.385878

Sum of electronic and zero-point Energies=   -1496.353924

Sum of electronic and thermal Energies=   -1496.320628

Sum of electronic and thermal Enthalpies=   -1496.319684

Sum of electronic and thermal Free Energies=   -1496.424112

E(RM06L) = -1498.39384385

Atom   X   Y   Z

B   4.120160000000   -0.716296000000   0.419069000000

C   2.882164000000   0.664688000000   0.697327000000

Pd 1.522793000000   -0.809819000000 -0.110449000000

C -0.056956000000   0.311440000000 -0.679140000000

C -1.048159000000   -0.609148000000 -0.767659000000

C -0.474947000000   -1.990272000000 -0.567351000000

C -0.861900000000   -2.877331000000   0.483406000000

C   0.512375000000   -2.448769000000 -1.504889000000

C -0.141169000000   1.767632000000 -0.873243000000

C   0.297552000000   2.357389000000 -2.083703000000

C -0.693186000000   2.606285000000   0.124162000000

O   3.233634000000   -1.915544000000   0.695986000000

C   0.167157000000   3.736022000000 -2.296029000000

H   0.734954000000   1.717863000000 -2.857493000000

C -0.817598000000   3.987318000000 -0.090349000000

H -1.019022000000   2.162190000000   1.070087000000

C -0.391875000000   4.557015000000 -1.300858000000

H   0.500637000000   4.172753000000 -3.243702000000

H -1.251563000000   4.620020000000   0.691550000000

H -0.492472000000   5.634535000000 -1.468310000000

C -0.305277000000   -4.150995000000   0.583788000000

H -1.593571000000   -2.538382000000   1.222971000000

C   1.069843000000   -3.750195000000 -1.383005000000

H   0.697984000000   -1.860607000000 -2.409623000000

C   0.665615000000   -4.591808000000 -0.348253000000

H -0.614649000000   -4.814845000000   1.397900000000

H   1.800845000000   -4.090285000000 -2.124262000000

C   3.157106000000   1.819378000000 -0.088811000000

C   2.428078000000   0.881837000000   2.035732000000

C   3.051432000000   3.108906000000   0.441280000000

H   3.504831000000   1.668270000000 -1.116170000000

C   2.302642000000   2.172705000000   2.568423000000

H   2.205632000000   0.015301000000   2.670969000000

C   2.618688000000   3.285977000000   1.769607000000

H   3.292888000000   3.982118000000 -0.174237000000

H   1.962382000000   2.316709000000   3.600237000000

O   4.557027000000   -0.701630000000 -0.941588000000

O 5.182127000000 -0.524914000000 1.348209000000

H 5.497090000000 -0.448148000000 -0.943159000000

H 3.493028000000 -2.594970000000 0.042430000000

H 4.878451000000 -0.712750000000 2.251787000000

H 2.528282000000 4.296710000000 2.183012000000

H 1.088144000000 -5.597856000000 -0.259045000000

C -2.506784000000 -0.367540000000 -1.108548000000

H -2.585195000000 0.635304000000 -1.566534000000

H -2.824961000000 -1.098714000000 -1.878735000000

C -3.479733000000 -0.447432000000 0.092143000000

H -3.156171000000 0.258810000000 0.877238000000

H -3.463382000000 -1.457888000000 0.538410000000

C -4.909076000000 -0.119422000000 -0.330530000000

H -5.283847000000 -0.816912000000 -1.101246000000

H -4.991459000000 0.901375000000 -0.745625000000

O -5.751726000000 -0.221560000000 0.851307000000

C -7.078802000000 0.026810000000 0.632948000000

O -7.546150000000 0.300213000000 -0.463187000000

C -7.857466000000 -0.062651000000 1.931617000000

H -7.655761000000 0.832095000000 2.545200000000

H -8.931742000000 -0.107732000000 1.708850000000

H -7.548283000000 -0.941378000000 2.518612000000

^{3}J

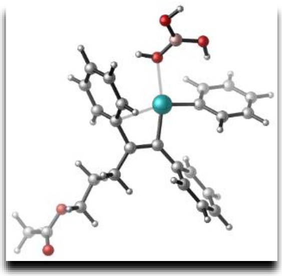

Zero-point correction= 0.458395 (Hartree/Particle)

Thermal correction to Energy= 0.492352

Thermal correction to Enthalpy= 0.493296

Thermal correction to Gibbs Free Energy= 0.384225

Sum of electronic and zero-point Energies= -1496.396107

Sum of electronic and thermal Energies= -1496.362151

Sum of electronic and thermal Enthalpies= -1496.361207

Sum of electronic and thermal Free Energies= -1496.470278

E(RM06L) = -1498.43549297

Atom X Y Z

B 4.224799000000 -2.158508000000 -0.076080000000

C 2.623858000000 1.004202000000 0.598426000000

Pd 1.420321000000 -0.469287000000 -0.033507000000

C -0.185045000000 0.647411000000 -0.491502000000

C -1.169909000000 -0.278992000000 -0.653790000000

C -0.657937000000 -1.695194000000 -0.530842000000

C -0.998529000000 -2.560523000000 0.548995000000

C 0.235592000000 -2.192386000000 -1.525725000000

C -0.325101000000 2.106978000000 -0.637990000000

C -0.623855000000 2.666905000000 -1.904736000000

C -0.177172000000 2.982918000000 0.463871000000

O 2.936735000000 -2.155392000000 0.494498000000

C -0.786002000000 4.052430000000 -2.059945000000

H -0.722874000000 2.000486000000 -2.768563000000

C -0.350064000000 4.364299000000 0.308039000000

H 0.080274000000 2.564465000000 1.441511000000

C -0.653877000000 4.906151000000 -0.953404000000

H -1.015951000000 4.464367000000 -3.048657000000

H -0.239068000000 5.024297000000 1.175122000000

H -0.779931000000 5.987342000000 -1.073130000000

C -0.477234000000 -3.854885000000 0.627584000000

H -1.653898000000 -2.187973000000 1.341610000000

C 0.760267000000 -3.504427000000 -1.438799000000

H 0.442909000000 -1.576799000000 -2.406426000000

C 0.409013000000 -4.332488000000 -0.364705000000

H -0.747218000000 -4.498398000000 1.471428000000

H 1.436814000000 -3.868182000000 -2.218948000000

C 2.977483000000 2.123285000000 -0.186978000000

C 3.252233000000 0.840523000000 1.858435000000

C 3.943847000000 3.039773000000 0.263747000000

H 2.491896000000 2.289563000000 -1.153530000000

C 4.210726000000 1.771192000000 2.313181000000

H 2.993310000000 -0.013373000000 2.494744000000

C 4.561270000000 2.870494000000 1.514671000000

H 4.212779000000 3.893991000000 -0.368377000000

H 4.677549000000 1.630331000000 3.295024000000

O 4.600364000000 -3.293792000000 -0.762789000000

O 5.062636000000 -1.093781000000 0.069837000000

H 5.506997000000 -3.192005000000 -1.102401000000

H 2.448526000000 -2.974736000000 0.263837000000

H 4.628151000000 -0.345867000000 0.538534000000

H 5.308320000000 3.590956000000 1.864435000000

H 0.806010000000 -5.351155000000 -0.301901000000

C -2.634343000000 -0.005279000000 -0.958271000000

H -2.739999000000 1.071150000000 -1.180840000000

H -2.921439000000 -0.550988000000 -1.880647000000

C -3.621658000000 -0.389628000000 0.168726000000

H -3.337977000000 0.125396000000 1.104080000000

H -3.579188000000 -1.475356000000 0.367290000000

C -5.055334000000 -0.019653000000 -0.201776000000

H -5.385787000000 -0.521289000000 -1.129144000000

H -5.174224000000 1.067861000000 -0.357088000000

O -5.914230000000 -0.438322000000 0.896109000000

C -7.251090000000 -0.239551000000 0.689570000000

O -7.715259000000 0.247626000000 -0.331199000000

C -8.047781000000 -0.707694000000 1.892834000000

H -7.873584000000 -1.781614000000 2.071530000000

H -7.723313000000 -0.169999000000 2.798802000000

H -9.115956000000 -0.528614000000 1.714120000000

^{3}K

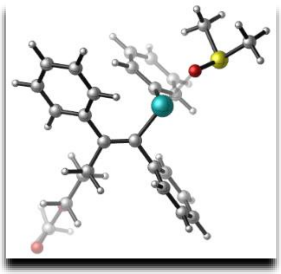

Zero-point correction= 0.487483 (Hartree/Particle)

Thermal correction to Energy= 0.523048

Thermal correction to Enthalpy= 0.523992

Thermal correction to Gibbs Free Energy= 0.411673

Sum of electronic and zero-point Energies= -1797.102249

Sum of electronic and thermal Energies= -1797.066684

Sum of electronic and thermal Enthalpies= -1797.065740

Sum of electronic and thermal Free Energies= -1797.178059

E(RM06L) = -1799.13461479

Atom	X	Y	Z
C	-1.549275000000	0.220049000000	1.199519000000
C	0.233223000000	-0.330269000000	-0.925234000000
C	1.082957000000	0.718655000000	-1.152980000000
C	0.631191000000	2.135105000000	-1.226838000000
C	1.457496000000	3.195804000000	-0.776901000000
C	-0.617944000000	2.490769000000	-1.796621000000
C	0.652905000000	-1.756521000000	-0.940699000000
Pd	-1.742387000000	-0.284778000000	-0.720183000000
C	0.724534000000	-2.461524000000	-2.170823000000
C	0.962164000000	-2.466675000000	0.248296000000
O	-3.970439000000	-0.363084000000	-0.853453000000
C	1.084328000000	-3.816999000000	-2.209133000000
H	0.496391000000	-1.926700000000	-3.099907000000

C 1.324768000000 -3.820110000000 0.205122000000

H 0.904377000000 -1.940790000000 1.206786000000

S -4.687162000000 -1.031281000000 0.360757000000

C 1.385612000000 -4.504229000000 -1.021809000000

H 1.133508000000 -4.337226000000 -3.172264000000

H 1.564002000000 -4.345528000000 1.136609000000

H 1.667957000000 -5.561823000000 -1.051353000000

C 1.039341000000 4.532473000000 -0.846569000000

H 2.436780000000 2.971867000000 -0.342817000000

C -1.040778000000 3.826184000000 -1.864459000000

H -1.254012000000 1.697083000000 -2.207888000000

C -0.215951000000 4.857535000000 -1.385494000000

H 1.699224000000 5.324062000000 -0.474727000000

H -2.010977000000 4.062213000000 -2.315750000000

C -5.187648000000 0.338662000000 1.474565000000

C -6.352705000000 -1.427558000000 -0.299429000000

H -5.713239000000 1.109077000000 0.889031000000

H -5.830444000000 -0.067114000000 2.272562000000

H -4.259196000000 0.746468000000 1.901454000000

H -6.992106000000 -1.793649000000 0.519588000000

H -6.779648000000 -0.526802000000 -0.766679000000

H -6.217278000000 -2.216901000000 -1.052918000000

C -1.602375000000 -0.788887000000 2.185998000000

C -1.533429000000 1.573327000000 1.596416000000

C -1.672379000000 -0.445127000000 3.550949000000

H -1.585668000000 -1.844689000000 1.895240000000

C -1.605128000000 1.907886000000 2.962780000000

H -1.460840000000 2.366604000000 0.846061000000

C -1.678440000000 0.903093000000 3.942847000000

H -1.715312000000 -1.238787000000 4.306293000000

H -1.594287000000 2.963535000000 3.258632000000

H -1.727805000000 1.169665000000 5.004186000000

H -0.540197000000 5.901810000000 -1.446687000000

C 2.584194000000 0.463164000000 -1.344421000000

H 2.719897000000 -0.464177000000 -1.929189000000

H 3.024213000000 1.281265000000 -1.944530000000

C 3.364883000000 0.315285000000 -0.015246000000

H 3.220098000000 1.204307000000 0.624364000000

H 2.964468000000 -0.545500000000 0.547220000000

C 4.855892000000 0.109644000000 -0.262777000000

H 5.311585000000 0.960474000000 -0.801013000000

H 5.052875000000 -0.798892000000 -0.860142000000

O 5.503912000000 -0.028197000000 1.035203000000

C 6.858753000000 -0.187875000000 0.991576000000

O 7.510632000000 -0.209800000000 -0.043224000000

C 7.418702000000 -0.343929000000 2.393884000000

H 7.061654000000 0.467709000000 3.047814000000

H 7.070429000000 -1.294111000000 2.832750000000

H 8.515756000000 -0.342077000000 2.350250000000

$^{3}TS_{KL}$

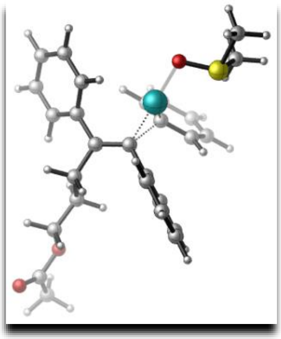

Frequency -266.2591

Zero-point correction= 0.486850 (Hartree/Particle)

Thermal correction to Energy= 0.521637

Thermal correction to Enthalpy= 0.522582

Thermal correction to Gibbs Free Energy= 0.412975

Sum of electronic and zero-point Energies= -1797.088990

Sum of electronic and thermal Energies= -1797.054202

Sum of electronic and thermal Enthalpies= -1797.053258

Sum of electronic and thermal Free Energies= -1797.162864

E(RM06L) = -1799.11509997

Atom	X	Y	Z
C	-1.017957000000	0.133510000000	1.323424000000
C	0.038860000000	-0.160394000000	-0.378319000000
C	0.924722000000	0.827270000000	-0.766285000000
C	0.592280000000	2.276473000000	-0.777161000000
C	1.536191000000	3.247598000000	-0.351913000000
C	-0.655720000000	2.754591000000	-1.251916000000
C	0.395727000000	-1.606332000000	-0.545181000000
Pd	-1.992705000000	0.078228000000	-0.471368000000
C	0.067411000000	-2.258065000000	-1.761031000000
C	1.067675000000	-2.360726000000	0.446070000000
O	-4.086570000000	0.086440000000	-1.139026000000
C	0.399345000000	-3.602647000000	-1.977427000000
H	-0.454468000000	-1.686558000000	-2.536475000000
C	1.394395000000	-3.710355000000	0.230838000000
H	1.323200000000	-1.883751000000	1.397157000000
S	-4.759957000000	-1.159098000000	-0.471271000000
C	1.062834000000	-4.338024000000	-0.979475000000
H	0.139750000000	-4.078015000000	-2.929774000000
H	1.914542000000	-4.271086000000	1.015420000000
H	1.320132000000	-5.389377000000	-1.146074000000
C	1.229089000000	4.614903000000	-0.351405000000
H	2.516492000000	2.925487000000	0.011864000000
C	-0.968273000000	4.122086000000	-1.244909000000
H	-1.378020000000	2.026643000000	-1.648058000000
C	-0.028895000000	5.061154000000	-0.790972000000
H	1.975603000000	5.335699000000	-0.000253000000
H	-1.943420000000	4.453796000000	-1.617945000000
C	-5.046281000000	-0.721723000000	1.288686000000
C	-6.513930000000	-1.029056000000	-1.024118000000
H	-5.545004000000	0.258496000000	1.344476000000
H	-5.659755000000	-1.510629000000	1.753815000000
H	-4.053696000000	-0.673848000000	1.763032000000
H	-7.113624000000	-1.803322000000	-0.519805000000
H	-6.890010000000	-0.019895000000	-0.795587000000
H	-6.517148000000	-1.197952000000	-2.110668000000
C	-1.179800000000	-1.031816000000	2.118026000000
C	-0.876125000000	1.379137000000	1.987329000000
C	-1.210790000000	-0.950332000000	3.522570000000
H	-1.268277000000	-2.009463000000	1.634729000000
C	-0.898281000000	1.448721000000	3.388198000000
H	-0.751047000000	2.295913000000	1.406370000000
C	-1.067154000000	0.288661000000	4.165809000000
H	-1.345751000000	-1.865273000000	4.111557000000
H	-0.786349000000	2.423778000000	3.876439000000
H	-1.082573000000	0.351519000000	5.259082000000
H	-0.266466000000	6.130247000000	-0.794948000000
C	2.338863000000	0.465362000000	-1.240524000000
H	2.291668000000	-0.456359000000	-1.847610000000
H	2.701595000000	1.265087000000	-1.912394000000
C	3.373885000000	0.242762000000	-0.109415000000
H	3.435745000000	1.125985000000	0.550743000000
H	3.052654000000	-0.601856000000	0.522455000000
C	4.757206000000	-0.055120000000	-0.680014000000
H	5.144488000000	0.777313000000	-1.294874000000
H	4.753145000000	-0.958425000000	-1.316561000000
O	5.659823000000	-0.271688000000	0.442899000000
C	6.957916000000	-0.521026000000	0.100484000000
O	7.365365000000	-0.551407000000	-1.052350000000
C	7.794972000000	-0.776604000000	1.340473000000
H	7.603885000000	-1.799569000000	1.708016000000
H	8.859431000000	-0.684952000000	1.086146000000
H	7.529097000000	-0.079363000000	2.149782000000

^{3}L

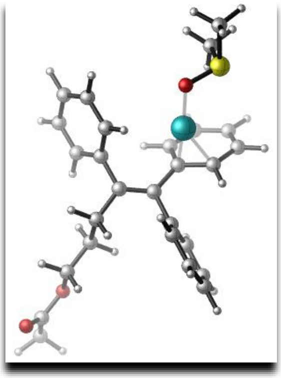

Zero-point correction= 0.488196 (Hartree/Particle)

Thermal correction to Energy= 0.523467

Thermal correction to Enthalpy= 0.524411

Thermal correction to Gibbs Free Energy= 0.412266

Sum of electronic and zero-point Energies= -1797.124145

Sum of electronic and thermal Energies= -1797.088874

Sum of electronic and thermal Enthalpies= -1797.087930

Sum of electronic and thermal Free Energies= -1797.200075

E(RM06L) = -1799.15585846

**Atom**	**X**	**Y**	**Z**
C	-0.664898000000	-0.617754000000	1.002932000000
C	0.563237000000	-0.511666000000	0.133144000000
C	1.156633000000	0.660768000000	-0.267471000000
C	0.509756000000	2.000718000000	-0.119267000000
C	1.125662000000	3.054839000000	0.595587000000
C	-0.708648000000	2.278014000000	-0.785840000000
C	1.194172000000	-1.832398000000	-0.215138000000
Pd	-2.320818000000	-0.278672000000	-0.329432000000
C	1.159313000000	-2.325948000000	-1.538267000000
C	1.800147000000	-2.626511000000	0.784002000000
O	-4.044372000000	0.284980000000	-1.586513000000
C	1.730314000000	-3.568821000000	-1.856448000000
H	0.668686000000	-1.727342000000	-2.313639000000
C	2.377041000000	-3.865202000000	0.465112000000
H	1.812507000000	-2.264759000000	1.817989000000
S	-5.238530000000	-0.306309000000	-0.770483000000
C	2.344795000000	-4.340163000000	-0.856766000000
H	1.690409000000	-3.936510000000	-2.887514000000
H	2.850170000000	-4.462779000000	1.251837000000
H	2.791108000000	-5.308881000000	-1.104997000000
C	0.529912000000	4.323642000000	0.672176000000
H	2.070613000000	2.872768000000	1.118066000000
C	-1.301479000000	3.548031000000	-0.715995000000
H	-1.171329000000	1.483646000000	-1.392777000000
C	-0.687969000000	4.574997000000	0.019211000000
H	1.020369000000	5.118026000000	1.245299000000
H	-2.239849000000	3.733914000000	-1.249349000000
C	-5.431601000000	0.706615000000	0.752811000000
C	-6.719740000000	0.366325000000	-1.654041000000
H	-5.455248000000	1.774665000000	0.485406000000
H	-6.360992000000	0.402363000000	1.262619000000
H	-4.551915000000	0.486036000000	1.377837000000
H	-7.632259000000	0.079998000000	-1.107173000000
H	-6.629913000000	1.460740000000	-1.733447000000
H	-6.723330000000	-0.087413000000	-2.655634000000
C	-1.481108000000	-1.821941000000	0.962800000000
C	-0.864398000000	0.262301000000	2.132862000000
C	-2.383383000000	-2.115337000000	2.033615000000
H	-1.212692000000	-2.630441000000	0.273858000000
C	-1.739153000000	-0.063390000000	3.165680000000
H	-0.265958000000	1.174994000000	2.196648000000
C	-2.498979000000	-1.261512000000	3.128648000000
H	-2.947678000000	-3.054061000000	1.999650000000
H	-1.828884000000	0.612325000000	4.023608000000
H	-3.166450000000	-1.514746000000	3.959517000000

H	-1.149085000000	5.566888000000	0.074677000000
C	2.531545000000	0.676081000000	-0.940576000000
H	2.614196000000	-0.174778000000	-1.639047000000
H	2.622421000000	1.595308000000	-1.547250000000
C	3.716401000000	0.600914000000	0.053709000000
H	3.678912000000	1.442187000000	0.768852000000
H	3.639991000000	-0.328111000000	0.645414000000
C	5.055250000000	0.632660000000	-0.677014000000
H	5.183162000000	1.555198000000	-1.271307000000
H	5.166930000000	-0.221653000000	-1.369060000000
O	6.107910000000	0.568910000000	0.327546000000
C	7.379573000000	0.651574000000	-0.164215000000
O	7.643835000000	0.781892000000	-1.351192000000
C	8.393054000000	0.533210000000	0.959061000000
H	8.073627000000	1.104629000000	1.844096000000
H	8.483749000000	-0.524147000000	1.261991000000
H	9.371159000000	0.885285000000	0.604811000000

^{3}M

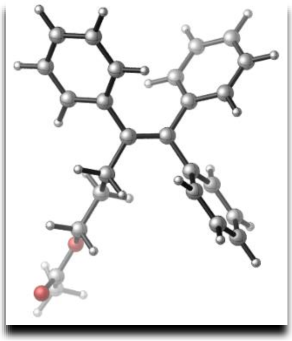

Zero-point correction= 0.410942 (Hartree/Particle)

Thermal correction to Energy= 0.436426

Thermal correction to Enthalpy= 0.437370

Thermal correction to Gibbs Free Energy= 0.350603

Sum of electronic and zero-point Energies= -1117.141329

Sum of electronic and thermal Energies= -1117.115844

Sum of electronic and thermal Enthalpies= -1117.114900

Sum of electronic and thermal Free Energies= -1117.201667

E(RM06L) = -1117.85667970

**Atom**	**X**	**Y**	**Z**
C	2.360923000000	0.725289000000	0.462681000000
C	1.049552000000	0.602844000000	-0.246036000000
C	0.476196000000	-0.594372000000	-0.608447000000
C	1.218052000000	-1.889358000000	-0.603100000000
C	0.604031000000	-3.090487000000	-0.171702000000
C	2.532245000000	-1.973662000000	-1.125116000000
C	0.357661000000	1.911840000000	-0.495875000000
C	0.078209000000	2.346370000000	-1.812101000000
C	0.024727000000	2.769876000000	0.577232000000
C	-0.537016000000	3.585859000000	-2.046404000000
H	0.363902000000	1.706878000000	-2.654743000000
C	-0.599004000000	4.004382000000	0.344123000000
H	0.260539000000	2.458330000000	1.600403000000
C	-0.884234000000	4.416277000000	-0.968469000000
H	-0.738921000000	3.906050000000	-3.074239000000
H	-0.859100000000	4.649203000000	1.190414000000
H	-1.365739000000	5.382712000000	-1.150442000000
C	1.285364000000	-4.315266000000	-0.227686000000
H	-0.415518000000	-3.065908000000	0.226882000000
C	3.209297000000	-3.197787000000	-1.190234000000
H	3.014854000000	-1.063639000000	-1.493084000000
C	2.592284000000	-4.375855000000	-0.736285000000
H	0.789775000000	-5.226240000000	0.124912000000
H	4.221725000000	-3.233294000000	-1.606616000000
C	3.277548000000	1.740952000000	0.099668000000
C	2.691609000000	-0.111853000000	1.554769000000
C	4.496152000000	1.888576000000	0.777099000000
H	3.027433000000	2.414549000000	-0.726653000000
C	3.904142000000	0.044422000000	2.239353000000

H 1.982583000000 -0.885156000000 1.864907000000

C 4.815679000000 1.040652000000 1.850095000000

H 5.195856000000 2.673191000000 0.469510000000

H 4.135413000000 -0.610894000000 3.086008000000

H 5.762990000000 1.161300000000 2.386030000000

H 3.121661000000 -5.332968000000 -0.787552000000

C -0.990848000000 -0.669681000000 -1.038669000000

H -1.230656000000 0.159126000000 -1.725174000000

H -1.157298000000 -1.603965000000 -1.603466000000

C -1.973251000000 -0.601559000000 0.157200000000

H -1.762318000000 -1.409087000000 0.881689000000

H -1.827658000000 0.351807000000 0.694421000000

C -3.422750000000 -0.708053000000 -0.306720000000

H -3.623283000000 -1.658873000000 -0.833004000000

H -3.695096000000 0.111245000000 -0.996426000000

O -4.268394000000 -0.634015000000 0.875428000000

C -5.609334000000 -0.737817000000 0.631058000000

O -6.086650000000 -0.887854000000 -0.484623000000

C -6.393501000000 -0.630844000000 1.925368000000

H -6.022877000000 -1.359799000000 2.663962000000

H -6.262573000000 0.372818000000 2.363434000000

H -7.457747000000 -0.806909000000 1.721578000000

N = 4

---

c4 = 6-Phenyl-5-hexynylacetate

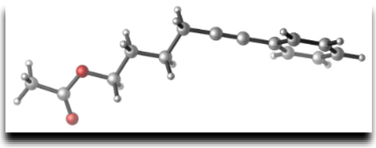

Zero-point correction= 0.258636 (Hartree/Particle)

Thermal correction to Energy= 0.275893

Thermal correction to Enthalpy= 0.276838

Thermal correction to Gibbs Free Energy= 0.207776

Sum of electronic and zero-point Energies= -693.268351

Sum of electronic and thermal Energies= -693.251094

Sum of electronic and thermal Enthalpies= -693.250150

Sum of electronic and thermal Free Energies= -693.319212

E(RM06L) = -693.726634501

Atom	X	Y	Z
H	-3.668296000000	1.602308000000	-1.362898000000
H	-6.023922000000	2.380625000000	-1.075553000000
H	-7.598872000000	1.101353000000	0.391354000000
C	-3.896656000000	-0.119334000000	-0.059742000000
H	-6.802883000000	-0.959072000000	1.569661000000
H	-4.446417000000	-1.735122000000	1.282715000000
C	-4.359602000000	1.046582000000	-0.722185000000
C	-5.681480000000	1.478413000000	-0.557750000000
C	-6.565930000000	0.760848000000	0.265440000000
C	-6.118486000000	-0.395674000000	0.926558000000
C	-4.798258000000	-0.835191000000	0.769326000000
C	-1.394179000000	-0.953295000000	-0.369233000000
C	-2.548018000000	-0.563926000000	-0.225071000000
C	-0.006635000000	-1.386459000000	-0.533609000000
H	0.164752000000	-2.313074000000	0.049271000000
H	0.174411000000	-1.658758000000	-1.592926000000
C	1.026282000000	-0.313408000000	-0.102495000000
H	0.849141000000	-0.052104000000	0.956838000000
H	0.847188000000	0.608726000000	-0.685260000000
C	2.474790000000	-0.792389000000	-0.297601000000
H	2.651364000000	-1.714904000000	0.286824000000
H	2.648080000000	-1.052655000000	-1.358732000000
C	3.487904000000	0.266723000000	0.126371000000
H	3.384413000000	0.535298000000	1.193150000000
H	3.380715000000	1.198929000000	-0.456951000000

O 4.815932000000 -0.283432000000 -0.097930000000

C 5.847069000000 0.553308000000 0.230391000000

O 5.688871000000 1.679932000000 0.677776000000

C 7.180300000000 -0.118632000000 -0.037291000000

H 7.274547000000 -1.032901000000 0.571674000000

H 7.251073000000 -0.422608000000 -1.094464000000

H 7.993670000000 0.576571000000 0.207884000000

^{4}B

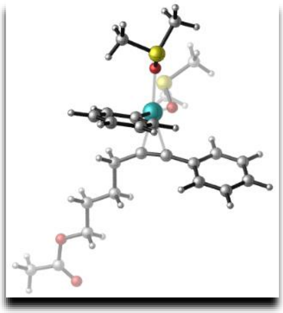

Zero-point correction= 0.504938 (Hartree/Particle)

Thermal correction to Energy= 0.544666

Thermal correction to Enthalpy= 0.545610

Thermal correction to Gibbs Free Energy= 0.421153

Sum of electronic and zero-point Energies= -2157.788599

Sum of electronic and thermal Energies= -2157.748871

Sum of electronic and thermal Enthalpies= -2157.747927

Sum of electronic and thermal Free Energies= -2157.872385

E(RM06L) = -2159.87992270

Atom	X	Y	Z
C	-0.829517000000	-0.141977000000	0.194456000000
C	1.160095000000	1.893463000000	0.386671000000
C	-0.264396000000	-0.672819000000	1.194560000000
C	0.133517000000	-1.276476000000	2.432925000000
C	0.821412000000	-2.518123000000	2.439171000000
H	1.065660000000	-2.986872000000	1.481226000000
C	-0.181900000000	-0.640056000000	3.661899000000
H	-0.710029000000	0.317690000000	3.650161000000
C	1.174126000000	-3.110587000000	3.656132000000
H	1.699892000000	-4.070506000000	3.661269000000
C	0.850097000000	-2.480694000000	4.871829000000
H	1.124847000000	-2.951676000000	5.820811000000
C	0.173176000000	-1.248682000000	4.871765000000
H	-0.079737000000	-0.761201000000	5.818278000000
C	-1.972662000000	0.295662000000	-0.635466000000
H	-1.891537000000	-0.156487000000	-1.641921000000
Pd	1.234279000000	0.018606000000	-0.279253000000
H	-1.903555000000	1.390333000000	-0.783302000000
O	1.303839000000	-2.139007000000	-0.967668000000
O	3.147832000000	0.276850000000	-1.234025000000
C	0.725398000000	2.915091000000	-0.481169000000
C	1.578969000000	2.205276000000	1.692326000000
C	0.736686000000	4.255333000000	-0.043600000000
H	0.372370000000	2.679246000000	-1.492061000000
C	1.589660000000	3.547655000000	2.118788000000
H	1.893038000000	1.414485000000	2.382494000000
C	1.171094000000	4.571585000000	1.253786000000
H	0.394680000000	5.046877000000	-0.719419000000
H	1.919686000000	3.786860000000	3.135493000000
H	1.172136000000	5.612073000000	1.593310000000
S	1.911589000000	-2.463743000000	-2.370783000000
C	0.940545000000	-3.901320000000	-2.954531000000
H	-0.078256000000	-3.540159000000	-3.155691000000
H	1.391140000000	-4.287147000000	-3.882761000000
H	0.923060000000	-4.672105000000	-2.168978000000
C	3.486385000000	-3.337996000000	-2.036205000000
H	3.904959000000	-3.708702000000	-2.985520000000
H	4.159088000000	-2.591527000000	-1.591578000000
H	3.308500000000	-4.164364000000	-1.330820000000
S	4.146338000000	1.391361000000	-0.716504000000

C 5.769489000000 0.712282000000 -1.207350000000
H 5.750657000000 0.441801000000 -2.273968000000
H 6.542783000000 1.470473000000 -1.005774000000
H 5.951506000000 -0.174349000000 -0.583026000000
C 4.048583000000 2.751008000000 -1.935359000000
H 4.870690000000 3.457210000000 -1.736348000000
H 4.112950000000 2.338565000000 -2.953958000000
H 3.080184000000 3.245083000000 -1.769666000000
C -3.334979000000 -0.070413000000 0.003332000000
H -3.378221000000 -1.164366000000 0.155606000000
H -3.395761000000 0.391324000000 1.005659000000
C -4.516767000000 0.391400000000 -0.866523000000
H -4.452132000000 -0.067424000000 -1.870868000000
H -4.475359000000 1.486556000000 -1.014003000000
C -5.858655000000 0.022170000000 -0.236987000000
H -5.990127000000 0.489755000000 0.755850000000
H -5.962722000000 -1.069267000000 -0.098115000000
O -6.897988000000 0.493669000000 -1.130519000000
C -8.175401000000 0.206647000000 -0.712234000000
O -8.418035000000 -0.394555000000 0.322596000000
C -9.191225000000 0.733004000000 -1.704113000000
H -9.065013000000 1.819139000000 -1.842907000000
H -9.043946000000 0.258828000000 -2.688524000000
H -10.202664000000 0.517812000000 -1.336394000000

$^4TS^{\alpha}_{BC}$

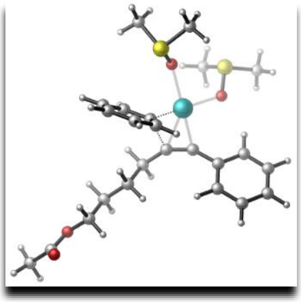

Frequency -265.2422

Zero-point correction= 0.504832 (Hartree/Particle)

Thermal correction to Energy= 0.543511

Thermal correction to Enthalpy= 0.544455

Thermal correction to Gibbs Free Energy= 0.424779

Sum of electronic and zero-point Energies= -2157.771387

Sum of electronic and thermal Energies= -2157.732707

Sum of electronic and thermal Enthalpies= -2157.731763

Sum of electronic and thermal Free Energies= -2157.851440

E(RM06L) = -2159.86261014

Atom X Y Z

C 0.626617000000 0.685661000000 -0.372533000000
C 0.189444000000 -1.024529000000 0.686356000000
C -0.187653000000 1.617935000000 0.023737000000
C -0.432487000000 2.926237000000 0.565840000000
C -1.666774000000 3.601207000000 0.379457000000
H -2.462375000000 3.099921000000 -0.178175000000
C 0.606263000000 3.565002000000 1.300070000000
H 1.552614000000 3.038744000000 1.457673000000
C -1.845731000000 4.885045000000 0.905247000000
H -2.798111000000 5.403768000000 0.756592000000
C -0.810270000000 5.514338000000 1.619311000000
H -0.957645000000 6.519628000000 2.026102000000
C 0.414683000000 4.851210000000 1.813545000000
H 1.220704000000 5.337205000000 2.371982000000
C 1.853845000000 0.416855000000 -1.184767000000
H 1.933099000000 1.223001000000 -1.938365000000
Pd -1.400371000000 -0.060258000000 -0.154849000000
H 1.733174000000 -0.529350000000 -1.739704000000
O -3.238444000000 1.062812000000 -0.742325000000
O -2.691640000000 -1.860250000000 -0.427186000000
C 0.613697000000 -2.229409000000 0.079454000000
C 0.424657000000 -0.808984000000 2.063372000000

C 1.246020000000 -3.216182000000 0.854705000000

H 0.450145000000 -2.396859000000 -0.990648000000

C 1.041607000000 -1.810617000000 2.831194000000

H 0.142432000000 0.142276000000 2.524680000000

C 1.456026000000 -3.011836000000 2.230039000000

H 1.579658000000 -4.145238000000 0.379957000000

H 1.217671000000 -1.640424000000 3.898389000000

H 1.956635000000 -3.779905000000 2.827622000000

S -4.514099000000 0.329989000000 -1.286295000000

C -4.092600000000 -0.281174000000 -2.961181000000

H -3.392677000000 -1.115960000000 -2.813981000000

H -5.011491000000 -0.638034000000 -3.453904000000

H -3.620722000000 0.528753000000 -3.538455000000

C -5.588239000000 1.724486000000 -1.782584000000

H -6.491299000000 1.324885000000 -2.270741000000

H -5.866908000000 2.258129000000 -0.862475000000

H -5.033074000000 2.392486000000 -2.458443000000

S -2.698752000000 -2.901222000000 0.753203000000

C -4.302112000000 -2.656133000000 1.604564000000

H -5.123389000000 -2.677595000000 0.872305000000

H -4.423611000000 -3.446534000000 2.362431000000

H -4.252923000000 -1.674596000000 2.098448000000

C -3.080144000000 -4.488978000000 -0.069092000000

H -3.242678000000 -5.258254000000 0.702431000000

H -3.970700000000 -4.370978000000 -0.705010000000

H -2.204111000000 -4.752655000000 -0.678653000000

C 3.146733000000 0.357413000000 -0.337118000000

H 3.265034000000 1.313697000000 0.205734000000

H 3.042928000000 -0.431484000000 0.429269000000

C 4.389325000000 0.092765000000 -1.205419000000

H 4.493637000000 0.879583000000 -1.975390000000

H 4.276846000000 -0.866225000000 -1.744776000000

C 5.662783000000 0.044665000000 -0.363727000000

H 5.623354000000 -0.755222000000 0.397690000000

H 5.837295000000 0.995374000000 0.172672000000

O 6.769444000000 -0.206183000000 -1.265523000000

C 7.990512000000 -0.314038000000 -0.644852000000

O 8.133589000000 -0.222234000000 0.564728000000

C 9.089962000000 -0.531777000000 -1.662844000000

H 9.305626000000 0.418288000000 -2.181134000000

H 9.999094000000 -0.870269000000 -1.148766000000

H 8.783849000000 -1.262669000000 -2.427041000000

$^{4}C^{\alpha}$

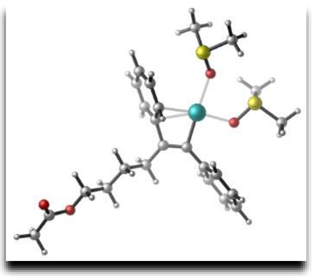

Zero-point correction= 0.507135 (Hartree/Particle)

Thermal correction to Energy= 0.545795

Thermal correction to Enthalpy= 0.546739

Thermal correction to Gibbs Free Energy= 0.426824

Sum of electronic and zero-point Energies= -2157.813828

Sum of electronic and thermal Energies= -2157.775168

Sum of electronic and thermal Enthalpies= -2157.774223

Sum of electronic and thermal Free Energies= -2157.894138

E(RM06L) = -2159.90537755

Atom X Y Z

C -1.124689000000 0.207030000000 0.585159000000

C -0.599259000000 -1.201180000000 0.563898000000

C -0.166081000000 1.032782000000 0.109371000000

C -0.064124000000 2.479989000000 -0.051104000000

C -0.235989000000 3.337353000000 1.066035000000

H	-0.408218000000	2.898216000000	2.053859000000
C	0.184777000000	3.054374000000	-1.323025000000
H	0.328775000000	2.395525000000	-2.185275000000
C	-0.195209000000	4.728443000000	0.904634000000
H	-0.343913000000	5.380580000000	1.771272000000
C	0.032799000000	5.285187000000	-0.365651000000
H	0.059775000000	6.372447000000	-0.489888000000
C	0.224418000000	4.444947000000	-1.477390000000
H	0.397577000000	4.878090000000	-2.467805000000
C	-2.535775000000	0.532909000000	1.021638000000
H	-2.662110000000	1.629052000000	1.041405000000
Pd	1.438117000000	-0.163293000000	-0.058605000000
H	-2.687062000000	0.178189000000	2.061475000000
O	2.749167000000	1.538649000000	0.047725000000
O	3.343714000000	-1.416809000000	0.108392000000
C	-0.612332000000	-2.059063000000	1.706262000000
C	-0.121281000000	-1.727277000000	-0.690688000000
C	-0.186601000000	-3.378880000000	1.601693000000
H	-0.977064000000	-1.668815000000	2.661724000000
C	0.312386000000	-3.084864000000	-0.767121000000
H	-0.359291000000	-1.189698000000	-1.615609000000
C	0.280170000000	-3.896119000000	0.363225000000
H	-0.223604000000	-4.031722000000	2.479876000000
H	0.615754000000	-3.493681000000	-1.735923000000
H	0.570487000000	-4.949663000000	0.292651000000
S	4.312427000000	1.372194000000	-0.072442000000
C	4.919026000000	1.051478000000	1.624262000000
H	4.601710000000	0.026922000000	1.865005000000
H	6.018469000000	1.125882000000	1.634243000000
H	4.464700000000	1.776834000000	2.316310000000
C	4.840349000000	3.113573000000	-0.231056000000
H	5.940982000000	3.156733000000	-0.218579000000
H	4.458856000000	3.474831000000	-1.196633000000

H	4.406192000000	3.700621000000	0.592070000000
S	3.646910000000	-2.660680000000	-0.790774000000
C	5.261678000000	-2.317055000000	-1.587271000000
H	6.012965000000	-2.060697000000	-0.824779000000
H	5.568589000000	-3.208529000000	-2.157353000000
H	5.106904000000	-1.472878000000	-2.274671000000
C	4.211697000000	-3.954876000000	0.373944000000
H	4.567707000000	-4.825040000000	-0.200506000000
H	5.009059000000	-3.549646000000	1.015594000000
H	3.337914000000	-4.234167000000	0.979724000000
C	-3.608771000000	-0.109810000000	0.111913000000
H	-3.467171000000	0.258233000000	-0.921954000000
H	-3.451951000000	-1.205253000000	0.076999000000
C	-5.042788000000	0.191388000000	0.582031000000
H	-5.209878000000	1.283984000000	0.612230000000
H	-5.190418000000	-0.180282000000	1.613242000000
C	-6.082392000000	-0.449770000000	-0.334158000000
H	-5.979941000000	-1.549676000000	-0.368405000000
H	-5.995639000000	-0.083370000000	-1.373530000000
O	-7.393012000000	-0.108593000000	0.184396000000
C	-8.439015000000	-0.650628000000	-0.520586000000
O	-8.285520000000	-1.367792000000	-1.497730000000
C	-9.763263000000	-0.222098000000	0.076355000000
H	-9.780502000000	-0.409006000000	1.161862000000
H	-9.907925000000	0.861374000000	-0.071537000000
H	-10.577687000000	-0.768770000000	-0.416293000000

$^4TS^{\beta}_{BC}$

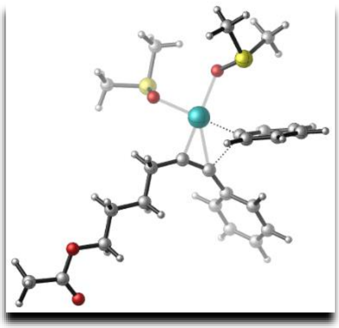

Frequency -246.9971

Zero-point correction= 0.504352 (Hartree/Particle)

Thermal correction to Energy= 0.543224

Thermal correction to Enthalpy= 0.544169

Thermal correction to Gibbs Free Energy= 0.423334

Sum of electronic and zero-point Energies= -2157.769825

Sum of electronic and thermal Energies= -2157.730953

Sum of electronic and thermal Enthalpies= -2157.730009

Sum of electronic and thermal Free Energies= -2157.850843

E(RM06L) = -2159.85502433

**Atom**	**X**	**Y**	**Z**
C	-0.225992000000	1.268568000000	0.195531000000
C	1.752679000000	1.376650000000	-0.559495000000
C	-0.488368000000	2.508494000000	0.902725000000
C	-1.115555000000	2.442386000000	2.173694000000
H	-1.317366000000	1.463378000000	2.619042000000
C	-0.205504000000	3.773234000000	0.332308000000
H	0.286429000000	3.824847000000	-0.643156000000
C	-1.469465000000	3.619553000000	2.844928000000
H	-1.955826000000	3.560719000000	3.823525000000
C	-1.196788000000	4.870002000000	2.265206000000
H	-1.472196000000	5.788584000000	2.792659000000
C	-0.566564000000	4.942872000000	1.009689000000
H	-0.357303000000	5.917408000000	0.557991000000
C	-0.634294000000	0.085614000000	-0.137495000000
C	-1.808993000000	-0.742035000000	-0.527520000000
H	-1.782614000000	-1.687049000000	0.044178000000
Pd	1.276468000000	-0.544592000000	-0.051723000000
H	-1.691441000000	-1.041496000000	-1.587440000000
O	0.711812000000	-2.664724000000	0.202075000000
O	3.404990000000	-1.232194000000	0.115758000000
C	1.668367000000	1.709616000000	-1.930359000000
C	2.652953000000	2.072233000000	0.282651000000
C	2.524809000000	2.689288000000	-2.465729000000
H	0.930847000000	1.214918000000	-2.569668000000
C	3.495677000000	3.053862000000	-0.260120000000
H	2.684223000000	1.850738000000	1.354274000000
C	3.436517000000	3.360442000000	-1.633739000000
H	2.462984000000	2.939865000000	-3.529943000000
H	4.190657000000	3.593357000000	0.392489000000
H	4.086389000000	4.137292000000	-2.048867000000
S	1.055730000000	-3.265935000000	1.612321000000
C	2.652149000000	-4.138462000000	1.411291000000
H	3.375171000000	-3.350340000000	1.151715000000
H	2.918380000000	-4.618656000000	2.366618000000
H	2.574000000000	-4.878093000000	0.599448000000
C	-0.036687000000	-4.727920000000	1.727182000000
H	0.244950000000	-5.312070000000	2.617593000000
H	-1.067278000000	-4.359925000000	1.832444000000
H	0.065347000000	-5.326337000000	0.808896000000
S	4.427318000000	-0.615459000000	-0.903361000000
C	5.973687000000	-0.443573000000	0.057401000000
H	6.196734000000	-1.390731000000	0.572016000000
H	6.789737000000	-0.162713000000	-0.627262000000
H	5.805129000000	0.360919000000	0.787243000000
C	4.937577000000	-2.013028000000	-1.973136000000
H	5.724516000000	-1.663314000000	-2.660431000000
H	5.295905000000	-2.852953000000	-1.358632000000
H	4.048553000000	-2.310750000000	-2.548190000000

C -3.157628000000 -0.012066000000 -0.331950000000

H -3.260328000000 0.287184000000 0.727476000000

H -3.152002000000 0.923103000000 -0.921407000000

C -4.347445000000 -0.894123000000 -0.749221000000

H -4.349702000000 -1.832005000000 -0.162785000000

H -4.249843000000 -1.185041000000 -1.811652000000

C -5.682039000000 -0.179548000000 -0.546132000000

H -5.844889000000 0.101150000000 0.509948000000

H -5.745379000000 0.748835000000 -1.142858000000

O -6.727701000000 -1.091603000000 -0.966256000000

C -8.003272000000 -0.626146000000 -0.754652000000

O -8.241571000000 0.456823000000 -0.242892000000

C -9.026284000000 -1.620685000000 -1.261767000000

H -9.092710000000 -1.550594000000 -2.361153000000

H -8.736405000000 -2.652865000000 -1.012443000000

H -10.008422000000 -1.381603000000 -0.833003000000

$^{4}TS_{BD}$

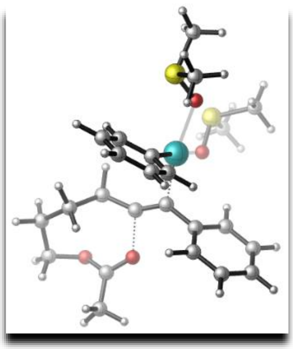

Frequency -286.3983

Zero-point correction= 0.505410 (Hartree/Particle)

Thermal correction to Energy= 0.543113

Thermal correction to Enthalpy= 0.544057

Thermal correction to Gibbs Free Energy= 0.429714

Sum of electronic and zero-point Energies= -2157.741515

Sum of electronic and thermal Energies= -2157.703812

Sum of electronic and thermal Enthalpies= -2157.702868

Sum of electronic and thermal Free Energies= -2157.817211

E(RM06L) = -2159.82374822

Atom X Y Z

C -1.855395000000 -0.159136000000 -0.578591000000

O -3.538824000000 -0.732213000000 -0.010520000000

Pd 0.908543000000 -0.089313000000 -0.089037000000

C 0.599735000000 1.729130000000 0.660736000000

O 1.211323000000 -2.165231000000 -0.925200000000

O 3.090127000000 0.173500000000 -0.307044000000

C 0.581791000000 2.852594000000 -0.194712000000

C 0.493553000000 1.930545000000 2.053646000000

C 0.482129000000 4.155133000000 0.335675000000

H 0.657657000000 2.723500000000 -1.281444000000

C 0.396862000000 3.233459000000 2.579671000000

H 0.477737000000 1.071234000000 2.733201000000

C 0.392936000000 4.348229000000 1.723119000000

H 0.474076000000 5.016944000000 -0.341488000000

H 0.320215000000 3.374234000000 3.663819000000

H 0.314022000000 5.359563000000 2.135002000000

S 2.519887000000 -2.479236000000 -1.717586000000

C 2.166349000000 -4.105905000000 -2.478868000000

H 1.377354000000 -3.945672000000 -3.227689000000

H 3.078627000000 -4.481750000000 -2.968721000000

H 1.816077000000 -4.802326000000 -1.701843000000

C 3.748511000000 -3.039103000000 -0.476643000000

H 4.645951000000 -3.411215000000 -0.996877000000

H 3.989639000000 -2.146473000000 0.118151000000

H 3.302786000000 -3.821440000000 0.156811000000

S 3.677404000000 1.616303000000 -0.532176000000

C 4.124923000000 2.249205000000 1.125989000000

H 4.717960000000 1.490592000000 1.659642000000

H 4.691858000000 3.185903000000 1.002007000000

H 3.178289000000 2.443941000000 1.651750000000

C 5.367215000000 1.271403000000 -1.142138000000

H 5.912506000000 2.224333000000 -1.231052000000

H 5.879389000000 0.588804000000 -0.447031000000

H 5.264967000000 0.807402000000 -2.133685000000

C -1.015574000000 -0.567138000000 0.336764000000

C -1.205591000000 -1.327718000000 1.587376000000

C -1.877930000000 -0.738585000000 2.684003000000

H -2.268002000000 0.279045000000 2.588212000000

C -0.690241000000 -2.638071000000 1.718291000000

H -0.150589000000 -3.078564000000 0.874598000000

C -2.039087000000 -1.452206000000 3.880460000000

H -2.560483000000 -0.985132000000 4.722674000000

C -1.537805000000 -2.758736000000 3.999897000000

H -1.667257000000 -3.314362000000 4.934065000000

C -0.866464000000 -3.347188000000 2.914693000000

H -0.473898000000 -4.365811000000 3.002265000000

C -2.029155000000 0.604062000000 -1.839706000000

H -2.346084000000 -0.080409000000 -2.645034000000

H -1.003288000000 0.920505000000 -2.093154000000

C -2.964965000000 1.863175000000 -1.795538000000

H -2.348331000000 2.762528000000 -1.954328000000

H -3.394792000000 1.987813000000 -0.784308000000

C -4.086178000000 1.836900000000 -2.860290000000

H -4.505262000000 2.849150000000 -3.003490000000

H -3.658895000000 1.533192000000 -3.832597000000

C -5.245753000000 0.898222000000 -2.492099000000

H -5.930899000000 1.357859000000 -1.759434000000

H -5.826993000000 0.567219000000 -3.365394000000

O -4.649271000000 -0.312654000000 -1.908125000000

C -4.615296000000 -0.433374000000 -0.575897000000

C -5.873124000000 -0.274630000000 0.239919000000

H -5.947605000000 0.757804000000 0.625696000000

H -6.773464000000 -0.501837000000 -0.349073000000

H -5.808377000000 -0.940065000000 1.113223000000

^{4}D

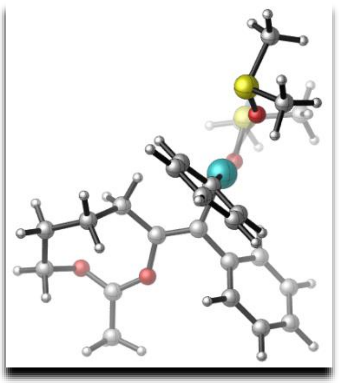

Zero-point correction= 0.507514 (Hartree/Particle)

Thermal correction to Energy= 0.544858

Thermal correction to Enthalpy= 0.545802

Thermal correction to Gibbs Free Energy= 0.433425

Sum of electronic and zero-point Energies= -2157.748689

Sum of electronic and thermal Energies= -2157.711345

Sum of electronic and thermal Enthalpies= -2157.710401

Sum of electronic and thermal Free Energies= -2157.822778

E(RM06L) = -2159.82906611

Atom X Y Z

C -2.050303000000 -0.105727000000 -0.548044000000

O -3.347746000000 -0.530887000000 -0.094488000000

Pd 0.862695000000 -0.127813000000 -0.073277000000

C 0.613458000000 1.729699000000 0.640400000000

O 1.132844000000 -2.234552000000 -0.838494000000

O 3.077373000000 0.054623000000 -0.301768000000

C 0.670730000000 2.846745000000 -0.224841000000

C 0.510044000000 1.961514000000 2.030138000000

C 0.649801000000 4.159253000000 0.288938000000

H 0.744736000000 2.703966000000 -1.309586000000

C 0.491804000000 3.273374000000 2.541572000000

H 0.438949000000 1.114772000000 2.721641000000

C 0.563580000000 4.376368000000 1.673019000000

H 0.699632000000 5.011305000000 -0.398957000000

H 0.417913000000 3.431243000000 3.623639000000

H 0.545417000000 5.395856000000 2.071966000000

S 2.436329000000 -2.595059000000 -1.621097000000

C 2.054030000000 -4.242506000000 -2.322266000000

H 1.270546000000 -4.096136000000 -3.079666000000

H 2.960323000000 -4.653983000000 -2.794312000000

H 1.688579000000 -4.902183000000 -1.520581000000

C 3.656592000000 -3.131085000000 -0.361934000000

H 4.548692000000 -3.532257000000 -0.869651000000

H 3.908965000000 -2.221510000000 0.201769000000

H 3.200294000000 -3.886546000000 0.296134000000

S 3.695788000000 1.468180000000 -0.597217000000

C 4.157025000000 2.172766000000 1.028389000000

H 4.734556000000 1.428799000000 1.598560000000

H 4.743493000000 3.090119000000 0.858854000000

H 3.215201000000 2.412752000000 1.543865000000

C 5.381264000000 1.066787000000 -1.187242000000

H 5.943046000000 2.004926000000 -1.320243000000

H 5.881502000000 0.409506000000 -0.459694000000

H 5.272726000000 0.557972000000 -2.155921000000

C -1.050446000000 -0.456808000000 0.316531000000

C -1.301653000000 -1.148514000000 1.603131000000

C -2.065194000000 -0.528508000000 2.623681000000

H -2.478859000000 0.470341000000 2.450743000000

C -0.741194000000 -2.424930000000 1.855029000000

H -0.133791000000 -2.894807000000 1.075025000000

C -2.264466000000 -1.169710000000 3.855569000000

H -2.844484000000 -0.669719000000 4.638576000000

C -1.721847000000 -2.444086000000 4.086289000000

H -1.881847000000 -2.945011000000 5.046301000000

C -0.964235000000 -3.068117000000 3.079728000000

H -0.537310000000 -4.061412000000 3.254682000000

C -1.948441000000 0.687803000000 -1.822360000000

H -2.160852000000 0.056034000000 -2.703003000000

H -0.876370000000 0.939365000000 -1.872654000000

C -2.805459000000 1.997733000000 -1.882592000000

H -2.141350000000 2.829320000000 -2.169867000000

H -3.177132000000 2.264318000000 -0.874422000000

C -3.973264000000 1.952489000000 -2.889337000000

H -4.387045000000 2.967263000000 -3.039545000000

H -3.599668000000 1.614652000000 -3.872664000000

C -5.131082000000 1.059762000000 -2.456032000000

H -5.701698000000 1.494817000000 -1.617075000000

H -5.821188000000 0.808308000000 -3.274703000000

O -4.556357000000 -0.240167000000 -2.001846000000

C -4.484941000000 -0.496248000000 -0.706181000000

C -5.693103000000 -0.861348000000 0.090940000000

H -6.451883000000 -0.060711000000 0.027992000000

H -6.155576000000 -1.768335000000 -0.337746000000

H -5.428486000000 -1.035911000000 1.141899000000

^{4}E

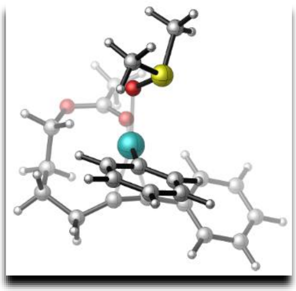

Zero-point correction= 0.427652 (Hartree/Particle)

Thermal correction to Energy= 0.458873

Thermal correction to Enthalpy= 0.459817

Thermal correction to Gibbs Free Energy= 0.360449

Sum of electronic and zero-point Energies= -1604.628967

Sum of electronic and thermal Energies= -1604.597747

Sum of electronic and thermal Enthalpies= -1604.596803

Sum of electronic and thermal Free Energies= -1604.696171

E(RM06L) = -1606.59695087

**Atom**	**X**	**Y**	**Z**
C	1.055319000000	0.359740000000	1.756325000000
C	-1.564303000000	0.942211000000	0.742978000000
C	1.478833000000	1.049477000000	0.782119000000
C	2.105397000000	2.002101000000	-0.089523000000
C	2.731974000000	1.587127000000	-1.293513000000
H	2.710833000000	0.527240000000	-1.563786000000
C	2.113029000000	3.375483000000	0.271564000000
H	1.625270000000	3.690645000000	1.198496000000
C	3.361344000000	2.532389000000	-2.110438000000
H	3.847507000000	2.211008000000	-3.036711000000
C	3.375614000000	3.890283000000	-1.743149000000
H	3.872783000000	4.624245000000	-2.384875000000
C	2.753172000000	4.307592000000	-0.553843000000
H	2.765556000000	5.364000000000	-0.268786000000
C	1.030738000000	-0.123929000000	3.158174000000
H	0.230017000000	0.412646000000	3.700305000000
Pd	-0.130339000000	-0.269302000000	0.094091000000
H	1.988486000000	0.198388000000	3.611181000000
O	-1.455870000000	-1.308465000000	-1.222970000000
C	-2.516323000000	0.442905000000	1.650872000000
C	-1.655097000000	2.256949000000	0.256931000000
C	-3.583701000000	1.269436000000	2.057029000000
H	-2.438418000000	-0.575894000000	2.045230000000
C	-2.729786000000	3.070698000000	0.666613000000
H	-0.901266000000	2.656106000000	-0.429797000000
C	-3.691727000000	2.579791000000	1.563532000000
H	-4.323229000000	0.883751000000	2.767128000000
H	-2.803203000000	4.094864000000	0.285662000000
H	-4.518614000000	3.220096000000	1.885996000000
S	-2.576133000000	-0.519040000000	-2.013508000000
C	-2.628430000000	-1.422457000000	-3.599469000000
H	-2.740541000000	-2.499529000000	-3.403566000000
H	-3.467979000000	-1.036054000000	-4.198927000000
H	-1.677710000000	-1.221472000000	-4.113712000000
C	-4.164413000000	-1.094830000000	-1.315789000000
H	-4.982343000000	-0.719503000000	-1.951650000000
H	-4.168922000000	-2.194645000000	-1.267803000000
H	-4.238692000000	-0.656896000000	-0.309604000000
C	0.853733000000	-1.651229000000	3.351221000000
H	1.051402000000	-1.873245000000	4.412713000000
H	-0.207733000000	-1.912640000000	3.182674000000
C	1.763962000000	-2.508929000000	2.431895000000
H	2.334035000000	-3.252365000000	3.012509000000
H	2.505856000000	-1.859701000000	1.933782000000
C	0.936173000000	-3.223243000000	1.354389000000
H	0.141182000000	-2.560696000000	0.963769000000
H	0.471563000000	-4.145804000000	1.735077000000
O	1.765313000000	-3.668960000000	0.239521000000
C	2.000186000000	-2.799100000000	-0.759044000000
O	1.525106000000	-1.654901000000	-0.839938000000
C	2.950185000000	-3.365365000000	-1.785014000000
H	3.984423000000	-3.113620000000	-1.491573000000
H	2.870803000000	-4.460460000000	-1.836331000000
H	2.750965000000	-2.911910000000	-2.765559000000

$^{4}TS^{\alpha}_{EF}$

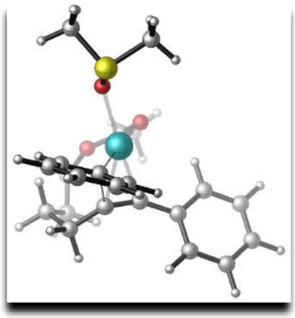

Frequency -317.1005

Zero-point correction= 0.427010 (Hartree/Particle)

Thermal correction to Energy= 0.457123

Thermal correction to Enthalpy= 0.458067

Thermal correction to Gibbs Free Energy= 0.362896

Sum of electronic and zero-point Energies= -1604.605571

Sum of electronic and thermal Energies= -1604.575458

Sum of electronic and thermal Enthalpies= -1604.574513

Sum of electronic and thermal Free Energies= -1604.669684

E(RM06L) = -1606.57006296

**Atom**	**X**	**Y**	**Z**
C	0.686873000000	0.834464000000	1.375383000000
C	-1.140179000000	1.336913000000	0.828281000000
C	1.414042000000	0.780555000000	0.288112000000
C	0.822841000000	0.632574000000	2.855295000000
Pd	-0.330380000000	-0.256613000000	-0.182272000000
C	-2.174830000000	1.024204000000	1.745990000000
C	-1.135188000000	2.601275000000	0.186576000000
C	2.449606000000	1.275372000000	-0.567656000000
C	2.825004000000	0.596097000000	-1.759032000000
C	3.122073000000	2.481958000000	-0.215392000000
H	2.301316000000	-0.324680000000	-2.032466000000
C	3.846535000000	1.107733000000	-2.564918000000
H	2.823090000000	3.016164000000	0.691545000000
C	4.149542000000	2.973662000000	-1.025284000000
H	4.127247000000	0.582613000000	-3.483203000000
C	4.513604000000	2.291843000000	-2.201225000000
H	5.313380000000	2.686460000000	-2.835420000000
H	4.663649000000	3.898356000000	-0.745413000000
H	-0.012735000000	1.130208000000	3.375435000000
H	1.754174000000	1.142960000000	3.166804000000
C	0.875094000000	-0.861141000000	3.281237000000
O	-2.103407000000	-1.342305000000	-0.853537000000
O	0.977123000000	-2.630904000000	0.927467000000
S	-3.348535000000	-0.522069000000	-1.348532000000
C	-3.185742000000	1.962716000000	2.007789000000
H	-2.188691000000	0.051616000000	2.249086000000
C	-2.162673000000	3.520225000000	0.440925000000
H	-0.314340000000	2.866432000000	-0.487109000000
C	-3.186375000000	3.207152000000	1.353902000000
H	-3.977140000000	1.717848000000	2.724145000000
H	-2.152557000000	4.493541000000	-0.060498000000
H	-3.974854000000	3.936126000000	1.564646000000
C	-3.265015000000	-0.590303000000	-3.176720000000
C	-4.742040000000	-1.679879000000	-1.105450000000
H	-3.131460000000	-1.633488000000	-3.501754000000
H	-4.186950000000	-0.154951000000	-3.594355000000
H	-2.398081000000	0.017029000000	-3.475836000000
H	-5.649105000000	-1.248614000000	-1.557768000000
H	-4.491277000000	-2.650542000000	-1.559759000000
H	-4.880893000000	-1.789679000000	-0.020401000000
H	0.815482000000	-0.892105000000	4.382868000000
H	-0.037597000000	-1.354952000000	2.903526000000
C	2.132121000000	-1.632990000000	2.795888000000
H	2.800805000000	-1.862307000000	3.644908000000
H	2.719476000000	-1.011068000000	2.095258000000
C	1.805726000000	-2.952510000000	2.089515000000
H	1.192802000000	-3.618412000000	2.719176000000
H	2.716398000000	-3.492145000000	1.783842000000

C 1.388941000000 -2.746574000000 -0.347262000000

O 0.824419000000 -2.034259000000 -1.194909000000

C 2.474973000000 -3.728504000000 -0.715005000000

H 3.465944000000 -3.326746000000 -0.438835000000

H 2.343139000000 -4.694512000000 -0.202672000000

H 2.458135000000 -3.877151000000 -1.802591000000

$^{4}F^{\alpha}$

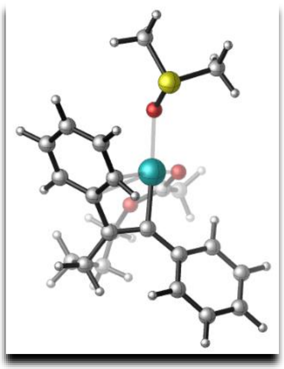

Zero-point correction= 0.429640 (Hartree/Particle)

Thermal correction to Energy= 0.459575

Thermal correction to Enthalpy= 0.460519

Thermal correction to Gibbs Free Energy= 0.365678

Sum of electronic and zero-point Energies= -1604.638733

Sum of electronic and thermal Energies= -1604.608798

Sum of electronic and thermal Enthalpies= -1604.607854

Sum of electronic and thermal Free Energies= -1604.702694

E(RM06L) = -1606.60684456

Atom X Y Z

C 1.157993000000 1.619617000000 -0.670924000000

C -0.297450000000 1.992479000000 -0.713912000000

C 1.245317000000 0.265725000000 -0.788320000000

C 2.260130000000 2.615466000000 -0.384066000000

Pd -0.611950000000 -0.260310000000 -0.234945000000

C -0.936741000000 2.971524000000 0.101554000000

C -1.088252000000 1.341057000000 -1.747505000000

C 2.292964000000 -0.668676000000 -1.176856000000

C 2.409904000000 -1.985968000000 -0.659095000000

C 3.211932000000 -0.261610000000 -2.187322000000

H 1.683590000000 -2.339475000000 0.076605000000

C 3.430440000000 -2.838607000000 -1.099127000000

H 3.098872000000 0.726029000000 -2.644837000000

C 4.235832000000 -1.115584000000 -2.609975000000

H 3.501782000000 -3.851128000000 -0.688805000000

C 4.353310000000 -2.407176000000 -2.066494000000

H 5.147246000000 -3.077876000000 -2.408891000000

H 4.935502000000 -0.778265000000 -3.381341000000

H 1.995914000000 3.591555000000 -0.831810000000

H 3.194433000000 2.285084000000 -0.869459000000

C 2.554538000000 2.835490000000 1.131760000000

O -2.615259000000 -0.783712000000 0.609699000000

O 1.176355000000 0.264780000000 2.186716000000

S -3.756963000000 -1.284298000000 -0.331429000000

C -2.279157000000 3.289060000000 -0.095664000000

H -0.363581000000 3.488145000000 0.875964000000

C -2.465451000000 1.675061000000 -1.906331000000

H -0.564317000000 0.850677000000 -2.578055000000

C -3.054604000000 2.633764000000 -1.087318000000

H -2.743479000000 4.064213000000 0.522906000000

H -3.033432000000 1.212390000000 -2.719866000000

H -4.100313000000 2.920865000000 -1.234979000000

C -3.891979000000 -3.087196000000 -0.027945000000

C -5.293876000000 -0.767704000000 0.516682000000

H -3.976950000000 -3.272974000000 1.053770000000

H -4.767096000000 -3.478728000000 -0.570926000000

H -2.973103000000 -3.545366000000 -0.422146000000

H -6.159542000000 -1.198726000000 -0.010810000000

H -5.257636000000 -1.101586000000 1.564991000000

H -5.329583000000 0.329845000000 0.467099000000

H 3.248909000000 3.691277000000 1.197990000000

H 1.635096000000 3.158109000000 1.652947000000

C 3.180283000000 1.624401000000 1.880479000000

H 4.060285000000 1.966023000000 2.456085000000

H 3.558715000000 0.873400000000 1.163908000000

C 2.281916000000 0.915136000000 2.897687000000

H 1.820017000000 1.627152000000 3.603132000000

H 2.853667000000 0.168129000000 3.470745000000

C 0.844574000000 -1.019353000000 2.333852000000

O 0.076917000000 -1.528090000000 1.489854000000

C 1.374203000000 -1.836497000000 3.486925000000

H 2.436445000000 -2.088683000000 3.320988000000

H 1.297922000000 -1.291692000000 4.441303000000

H 0.802682000000 -2.771637000000 3.545176000000

^{4}G

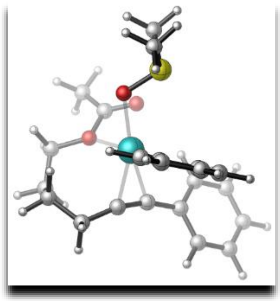

Zero-point correction= 0.427160 (Hartree/Particle)

Thermal correction to Energy= 0.458442

Thermal correction to Enthalpy= 0.459386

Thermal correction to Gibbs Free Energy= 0.359816

Sum of electronic and zero-point Energies= -1604.620118

Sum of electronic and thermal Energies= -1604.588835

Sum of electronic and thermal Enthalpies= -1604.587891

Sum of electronic and thermal Free Energies= -1604.687462

E(RM06L) = -1606.59019942

Atom X Y Z

C 1.345999000000 0.042267000000 1.758061000000

C -0.898056000000 1.687032000000 0.708236000000

C 1.829564000000 0.380398000000 0.637202000000

C 2.684638000000 0.913579000000 -0.387877000000

C 2.596307000000 0.459528000000 -1.728550000000

H 1.851610000000 -0.300130000000 -1.987682000000

C 3.642691000000 1.902789000000 -0.041692000000

H 3.700228000000 2.259252000000 0.990870000000

C 3.459687000000 0.983374000000 -2.698243000000

H 3.390428000000 0.630406000000 -3.731814000000

C 4.413794000000 1.954995000000 -2.348594000000

H 5.087076000000 2.359368000000 -3.110749000000

C 4.503192000000 2.410642000000 -1.021408000000

H 5.243496000000 3.169266000000 -0.749569000000

C 1.240709000000 -0.429066000000 3.152032000000

H 0.661386000000 0.302046000000 3.745330000000

Pd -0.265393000000 -0.165474000000 0.386677000000

H 2.263051000000 -0.444217000000 3.577796000000

O -2.200311000000 -0.731782000000 -0.333753000000

C -1.889941000000 1.885398000000 1.683485000000

C -0.419298000000 2.747562000000 -0.075091000000

C -2.420527000000 3.178663000000 1.863515000000

H -2.256163000000 1.054431000000 2.294639000000

C -0.966357000000 4.032759000000 0.112610000000

H 0.367714000000 2.592391000000 -0.819523000000

C -1.961539000000 4.248853000000 1.078382000000

H -3.190975000000 3.341532000000 2.624871000000

H -0.598712000000 4.864620000000 -0.497444000000

H -2.374035000000 5.251853000000 1.225339000000

S -2.748355000000 0.078172000000 -1.575523000000

C -3.196978000000 -1.235442000000 -2.760390000000

H -3.871263000000 -1.957807000000 -2.275688000000

H -3.673546000000 -0.773045000000 -3.639409000000

H -2.249871000000 -1.715726000000 -3.047221000000

C -4.420030000000 0.605993000000 -1.063394000000

H -4.935795000000 1.044434000000 -1.932698000000

H -4.972639000000 -0.258602000000 -0.664940000000

H -4.278074000000 1.369034000000 -0.284172000000

C 0.589499000000 -1.830882000000 3.283750000000

H 0.570960000000 -2.081958000000 4.357752000000

H -0.467286000000 -1.761235000000 2.965932000000

C 1.318115000000 -2.950200000000 2.491662000000

H 1.546257000000 -3.793269000000 3.168474000000

H 2.293234000000 -2.593029000000 2.113742000000

C 0.544215000000 -3.566699000000 1.327114000000

H -0.467586000000 -3.884826000000 1.637273000000

H 1.091420000000 -4.440542000000 0.940905000000

O 0.390341000000 -2.590144000000 0.237722000000

C 0.218301000000 -2.980199000000 -1.073362000000

O 0.084378000000 -2.090244000000 -1.903209000000

C 0.217038000000 -4.456602000000 -1.399927000000

H 1.210858000000 -4.901351000000 -1.218650000000

H -0.514408000000 -5.011603000000 -0.789658000000

H -0.027038000000 -4.571320000000 -2.463700000000

^{4}H

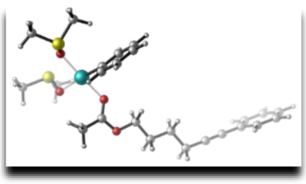

Zero-point correction= 0.506047 (Hartree/Particle)

Thermal correction to Energy= 0.545311

Thermal correction to Enthalpy= 0.546255

Thermal correction to Gibbs Free Energy= 0.424397

Sum of electronic and zero-point Energies= -2157.775449

Sum of electronic and thermal Energies= -2157.736184

Sum of electronic and thermal Enthalpies= -2157.735240

Sum of electronic and thermal Free Energies= -2157.857099

E(RM06L) = -2159.86938265

Atom X Y Z

H -8.981252000000 1.663201000000 -1.037744000000

H -10.937650000000 2.728777000000 0.091351000000

H -11.890781000000 1.714733000000 2.171676000000

C -8.818157000000 0.042979000000 0.399789000000

H -10.879091000000 -0.370095000000 3.116256000000

H -8.922856000000 -1.436260000000 1.987032000000

C -9.399760000000 1.224778000000 -0.126781000000

C -10.496667000000 1.818038000000 0.509614000000

C -11.032330000000 1.248748000000 1.677442000000

C -10.463711000000 0.078143000000 2.207745000000

C -9.366554000000 -0.523211000000 1.578727000000

C -6.747251000000 -1.079149000000 -0.827877000000

C -7.699380000000 -0.563978000000 -0.252330000000

C -5.592040000000 -1.664164000000 -1.507189000000

H -5.644692000000 -2.769971000000 -1.459369000000

H -5.625249000000 -1.405405000000 -2.584281000000

C -4.239634000000 -1.190862000000 -0.913446000000

H -4.188135000000 -1.497625000000 0.147375000000

H -4.214659000000 -0.085989000000 -0.921992000000

C -3.035987000000 -1.750770000000 -1.694483000000

H -3.067784000000 -2.855729000000 -1.708402000000

H -3.081470000000 -1.414243000000 -2.746564000000

C -1.720753000000 -1.289156000000 -1.082604000000

H -1.562310000000 -1.687647000000 -0.066169000000

H -1.637557000000 -0.190221000000 -1.038920000000

O -0.630179000000 -1.790633000000 -1.943509000000

C 0.609357000000 -1.431146000000 -1.634121000000

O 0.834385000000 -0.731689000000 -0.615541000000

C 1.652497000000 -1.923642000000 -2.595301000000

H 1.218312000000 -2.603609000000 -3.340405000000

H 2.099900000000 -1.053997000000 -3.106265000000

H 2.452969000000 -2.420381000000 -2.023551000000

Pd 2.748409000000 -0.058862000000 -0.039935000000

C 2.091925000000 1.790112000000 -0.283748000000

C 2.408599000000 2.516049000000 -1.447755000000

C 1.288904000000 2.370805000000 0.716657000000

C 1.924893000000 3.831109000000 -1.604504000000

C 0.814744000000 3.687127000000 0.553085000000

C 1.132973000000 4.417370000000 -0.605153000000

H 3.024663000000 2.070208000000 -2.236885000000

H 1.025352000000 1.806437000000 1.617753000000

H 2.168014000000 4.390143000000 -2.514669000000

H 0.190900000000 4.136651000000 1.333355000000

C 5.498931000000 -3.739870000000 0.834982000000

S 4.160722000000 -2.637064000000 1.422295000000

O 3.413593000000 -2.199871000000 0.120298000000

C 3.095259000000 -3.916916000000 2.186678000000

H 6.212684000000 -3.110722000000 0.284185000000

H 5.995835000000 -4.197708000000 1.705056000000

H 5.075749000000 -4.508369000000 0.169965000000

H 3.627905000000 -4.362195000000 3.042176000000

H 2.187627000000 -3.406032000000 2.539731000000

H 2.838106000000 -4.681233000000 1.437301000000

C 4.880999000000 3.197091000000 1.430321000000

S 5.248883000000 1.910837000000 0.183825000000

O 4.585317000000 0.574909000000 0.729504000000

C 7.002197000000 1.618765000000 0.599805000000

H 5.130550000000 2.819073000000 2.433524000000

H 5.469328000000 4.095511000000 1.182899000000

H 3.805313000000 3.412274000000 1.349295000000

H 7.568930000000 2.542739000000 0.404190000000

H 7.081678000000 1.320779000000 1.656189000000

H 7.361862000000 0.814851000000 -0.058134000000

H 0.758069000000 5.438096000000 -0.730193000000

^{4}I

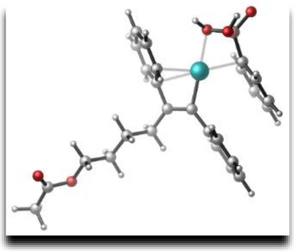

Zero-point correction= 0.484647 (Hartree/Particle)

Thermal correction to Energy= 0.519642

Thermal correction to Enthalpy= 0.520586

Thermal correction to Gibbs Free Energy= 0.412242

Sum of electronic and zero-point Energies= -1535.643762

Sum of electronic and thermal Energies= -1535.608767

Sum of electronic and thermal Enthalpies= -1535.607823

Sum of electronic and thermal Free Energies= -1535.716167

E(RM06L) = -1537.72783458

Atom X Y Z

C 0.777918000000 -0.055944000000 -0.552678000000

C 0.456932000000 -1.523294000000 -0.509380000000

C -0.293944000000 0.669077000000 -0.140444000000

C -0.403817000000 2.122955000000 0.017034000000

C -0.240437000000 2.993458000000 -1.089461000000

H -0.097620000000 2.562696000000 -2.085845000000

C -0.618421000000 2.694093000000 1.295419000000

H -0.766054000000 2.030139000000 2.153032000000

C -0.251741000000 4.385604000000 -0.915046000000

H -0.112938000000 5.042068000000 -1.780945000000

C -0.437657000000 4.936037000000 0.363631000000

H -0.441880000000 6.022552000000 0.500031000000

C -0.624148000000 4.084682000000 1.466823000000

H -0.773386000000 4.507015000000 2.466314000000

C 2.154381000000 0.428302000000 -0.960046000000

H 2.158970000000 1.532186000000 -0.971239000000

Pd -1.754175000000 -0.738081000000 -0.014363000000

H 2.364593000000 0.104022000000 -2.000720000000

C 0.620800000000 -2.404156000000 -1.618779000000

C -0.014645000000 -2.073048000000 0.734541000000

C 0.322783000000 -3.759225000000 -1.497319000000

H 0.977125000000 -1.999585000000 -2.571938000000

C -0.315713000000 -3.460892000000 0.832844000000

H 0.073319000000 -1.472121000000 1.647141000000

C -0.153550000000 -4.291722000000 -0.272335000000

H 0.451934000000 -4.422128000000 -2.359396000000

H -0.650638000000 -3.867261000000 1.792965000000

H -0.375764000000 -5.360809000000 -0.195708000000

C 3.284326000000 -0.095641000000 -0.044667000000

H 3.104331000000 0.260480000000 0.987369000000

H 3.239957000000 -1.201123000000 -0.003927000000

C 4.681023000000 0.346387000000 -0.516615000000

H 4.738568000000 1.450391000000 -0.547484000000

H 4.859288000000 -0.008130000000 -1.549330000000

C 5.785721000000 -0.188567000000 0.389449000000

H 5.792537000000 -1.292697000000 0.426830000000

H 5.679004000000 0.172500000000 1.428483000000

O 7.057788000000 0.274878000000 -0.144387000000

C 8.160599000000 -0.178599000000 0.525019000000

O 8.107662000000 -0.924816000000 1.492158000000

C 9.426471000000 0.393040000000 -0.084535000000

H 9.371266000000 0.398081000000 -1.183914000000

H 9.553965000000 1.438177000000 0.246319000000

H 10.290425000000 -0.193255000000 0.255880000000

B -4.351865000000 -1.406671000000 0.661093000000

C -3.872560000000 0.101736000000 0.145945000000

O -3.277787000000 -2.292983000000 -0.084773000000

C -3.963274000000 1.229781000000 1.020466000000

C -3.596595000000 0.382411000000 -1.240751000000

C -3.870638000000 2.535177000000 0.542535000000

H -4.153579000000 1.024542000000 2.079650000000

C -3.487026000000 1.709458000000 -1.719940000000

H -3.606182000000 -0.442525000000 -1.964853000000

C -3.635660000000 2.777154000000 -0.833025000000

H -3.978976000000 3.384061000000 1.226398000000

H -3.300325000000 1.895199000000 -2.783443000000

O -4.179763000000 -1.557924000000 2.091873000000

O -5.694491000000 -1.775721000000 0.273630000000

H -5.006981000000 -1.947244000000 2.426725000000

H -3.056230000000 -3.003120000000 0.549040000000

H -5.774279000000 -1.773070000000 -0.694731000000

H -3.563096000000 3.806963000000 -1.197577000000

$^{4}TS_{IJ}$

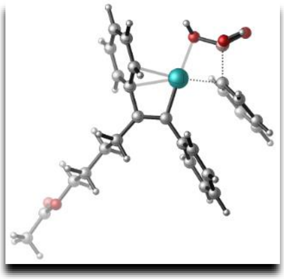

Frequency -143.2568

Zero-point correction= 0.483678 (Hartree/Particle)

Thermal correction to Energy= 0.518410

Thermal correction to Enthalpy= 0.519354

Thermal correction to Gibbs Free Energy= 0.410755

Sum of electronic and zero-point Energies= -1535.639168

Sum of electronic and thermal Energies= -1535.604437

Sum of electronic and thermal Enthalpies= -1535.603492

Sum of electronic and thermal Free Energies= -1535.712091

E(RM06L) = -1537.71691660

**Atom**	**X**	**Y**	**Z**
B	4.416927000000	-0.755387000000	0.488233000000
C	3.189451000000	0.639142000000	0.753070000000
Pd	1.832064000000	-0.811106000000	-0.100046000000
C	0.284055000000	0.334439000000	-0.706092000000
C	-0.715194000000	-0.572120000000	-0.835703000000
C	-0.164169000000	-1.960904000000	-0.619151000000
C	-0.584279000000	-2.838996000000	0.426434000000
C	0.837885000000	-2.434960000000	-1.532537000000
C	0.227327000000	1.792670000000	-0.894907000000
C	0.711334000000	2.382598000000	-2.087833000000
C	-0.342552000000	2.633891000000	0.090165000000
O	3.510827000000	-1.945543000000	0.736821000000
C	0.608247000000	3.764263000000	-2.295628000000
H	1.162424000000	1.740715000000	-2.851777000000
C	-0.439513000000	4.017836000000	-0.119438000000
H	-0.704856000000	2.188907000000	1.022299000000
C	0.031582000000	4.588024000000	-1.312835000000
H	0.977283000000	4.201260000000	-3.229941000000
H	-0.887479000000	4.652584000000	0.652893000000
H	-0.047143000000	5.667999000000	-1.476335000000
C	-0.047719000000	-4.120155000000	0.542027000000
H	-1.325756000000	-2.486892000000	1.150027000000
C	1.373897000000	-3.743789000000	-1.396346000000
H	1.055845000000	-1.848876000000	-2.431379000000
C	0.935419000000	-4.577347000000	-0.368914000000
H	-0.382674000000	-4.776775000000	1.351854000000
H	2.116809000000	-4.095500000000	-2.120165000000
C	3.497416000000	1.799549000000	-0.012149000000
C	2.707061000000	0.846470000000	2.083177000000
C	3.395764000000	3.083857000000	0.531232000000
H	3.866947000000	1.657649000000	-1.033255000000
C	2.585184000000	2.132467000000	2.628527000000
H	2.458633000000	-0.024370000000	2.702724000000
C	2.934138000000	3.250931000000	1.851129000000
H	3.662499000000	3.961092000000	-0.067930000000
H	2.222252000000	2.268388000000	3.653685000000
O	4.882495000000	-0.735230000000	-0.862803000000
O	5.461055000000	-0.583580000000	1.441123000000
H	5.825107000000	-0.492208000000	-0.842359000000
H	3.775345000000	-2.622958000000	0.083272000000
H	5.136340000000	-0.777031000000	2.336184000000
H	2.846940000000	4.257806000000	2.274551000000
H	1.341809000000	-5.588974000000	-0.268434000000
C	-2.153221000000	-0.311937000000	-1.240996000000
H	-2.210148000000	0.714801000000	-1.645762000000
H	-2.426081000000	-0.997949000000	-2.068667000000
C	-3.200794000000	-0.460731000000	-0.112486000000
H	-2.914593000000	0.193184000000	0.733042000000
H	-3.191896000000	-1.497542000000	0.274209000000
C	-4.620857000000	-0.107654000000	-0.591410000000
H	-4.903041000000	-0.753863000000	-1.443751000000
H	-4.642515000000	0.933375000000	-0.964041000000
C	-5.657107000000	-0.263423000000	0.516977000000
H	-5.438730000000	0.387908000000	1.382601000000
H	-5.708646000000	-1.300811000000	0.893949000000
O	-6.952482000000	0.103561000000	-0.036134000000
C	-7.998707000000	0.009036000000	0.838575000000
O	-7.882851000000	-0.365104000000	1.996988000000
C	-9.288685000000	0.451687000000	0.174338000000
H	-9.292430000000	1.550242000000	0.070035000000
H	-9.378914000000	0.024994000000	-0.836697000000
H	-10.141020000000	0.148755000000	0.796800000000

^{4}J

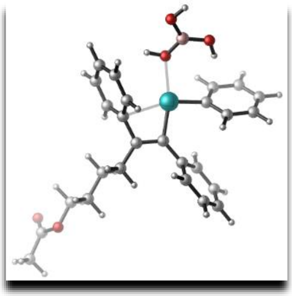

Zero-point correction= 0.486073 (Hartree/Particle)

Thermal correction to Energy= 0.521442

Thermal correction to Enthalpy= 0.522386

Thermal correction to Gibbs Free Energy= 0.409379

Sum of electronic and zero-point Energies= -1535.681344

Sum of electronic and thermal Energies= -1535.645975

Sum of electronic and thermal Enthalpies= -1535.645031

Sum of electronic and thermal Free Energies= -1535.758038

E(RM06L) = -1537.75838412

**Atom**	**X**	**Y**	**Z**
B	4.526798000000	-2.213716000000	0.071848000000
C	2.927133000000	0.985626000000	0.639423000000
Pd	1.730824000000	-0.472095000000	-0.040079000000
C	0.156847000000	0.666590000000	-0.549655000000
C	-0.831554000000	-0.246499000000	-0.759929000000
C	-0.339207000000	-1.669223000000	-0.628849000000
C	-0.727801000000	-2.538024000000	0.431781000000
C	0.584341000000	-2.170118000000	-1.593910000000
C	0.040531000000	2.129374000000	-0.685919000000
C	-0.200407000000	2.707003000000	-1.956891000000
C	0.156687000000	2.991563000000	0.430548000000
O	3.198038000000	-2.182558000000	0.537378000000
C	-0.338257000000	4.096209000000	-2.102635000000
H	-0.273453000000	2.051401000000	-2.831484000000
C	0.007700000000	4.376708000000	0.283952000000
H	0.370876000000	2.559136000000	1.412565000000
C	-0.239194000000	4.936172000000	-0.982223000000
H	-0.522881000000	4.521992000000	-3.095044000000
H	0.093352000000	5.025705000000	1.162164000000
H	-0.345948000000	6.020258000000	-1.094632000000
C	-0.224689000000	-3.839057000000	0.519891000000
H	-1.406400000000	-2.162626000000	1.203298000000
C	1.090424000000	-3.488709000000	-1.497822000000
H	0.831600000000	-1.550548000000	-2.461399000000
C	0.691090000000	-4.320316000000	-0.443339000000
H	-0.532214000000	-4.484845000000	1.349011000000
H	1.790057000000	-3.855065000000	-2.256179000000
C	3.328804000000	2.094433000000	-0.137343000000
C	3.498563000000	0.822650000000	1.926239000000
C	4.288127000000	3.000599000000	0.347705000000
H	2.885710000000	2.260735000000	-1.124127000000
C	4.450589000000	1.742998000000	2.414861000000
H	3.198348000000	-0.021291000000	2.557838000000
C	4.850501000000	2.831205000000	1.624334000000
H	4.595312000000	3.846866000000	-0.277692000000
H	4.873112000000	1.603108000000	3.416711000000
O	4.932675000000	-3.359257000000	-0.579836000000
O	5.372397000000	-1.165509000000	0.279742000000
H	5.865509000000	-3.277055000000	-0.845719000000
H	2.712988000000	-2.994038000000	0.274598000000
H	4.921212000000	-0.407861000000	0.715856000000
H	5.592489000000	3.543537000000	2.000543000000
H	1.073882000000	-5.343957000000	-0.373763000000
C	-2.279570000000	0.044680000000	-1.120252000000
H	-2.368503000000	1.124294000000	-1.333997000000
H	-2.534384000000	-0.487589000000	-2.060053000000
C	-3.323679000000	-0.340465000000	-0.046100000000

H -3.065594000000 0.161660000000 0.905646000000

H -3.280641000000 -1.428881000000 0.147764000000

C -4.755580000000 0.039097000000 -0.467131000000

H -5.009560000000 -0.454952000000 -1.423737000000

H -4.819827000000 1.128320000000 -0.646673000000

C -5.784072000000 -0.358446000000 0.586854000000

H -5.601352000000 0.144694000000 1.553656000000

H -5.780193000000 -1.446542000000 0.779321000000

O -7.097432000000 0.029336000000 0.092999000000

C -8.139574000000 -0.295801000000 0.915449000000

O -8.005879000000 -0.874654000000 1.984312000000

C -9.452995000000 0.173497000000 0.318691000000

H -9.489574000000 1.275981000000 0.314497000000

H -9.547460000000 -0.160448000000 -0.726884000000

H -10.285313000000 -0.216336000000 0.919171000000

^{4}K

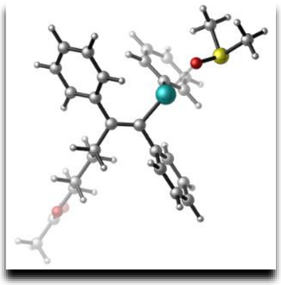

Zero-point correction= 0.515189 (Hartree/Particle)

Thermal correction to Energy= 0.552154

Thermal correction to Enthalpy= 0.553098

Thermal correction to Gibbs Free Energy= 0.437347

Sum of electronic and zero-point Energies= -1836.387350

Sum of electronic and thermal Energies= -1836.350385

Sum of electronic and thermal Enthalpies= -1836.349440

Sum of electronic and thermal Free Energies= -1836.465192

E(RM06L) = -1838.45733621

**Atom**	**X**	**Y**	**Z**
C	-1.786555000000	0.188114000000	1.230224000000
C	-0.098726000000	-0.316177000000	-0.987375000000
C	0.712794000000	0.752087000000	-1.259683000000
C	0.223254000000	2.157821000000	-1.292960000000
C	1.041689000000	3.230984000000	-0.858901000000
C	-1.056322000000	2.491059000000	-1.805330000000
C	0.347305000000	-1.733220000000	-1.030074000000
Pd	-2.061471000000	-0.305778000000	-0.683897000000
C	0.377189000000	-2.433612000000	-2.264412000000
C	0.720933000000	-2.440452000000	0.141825000000
O	-4.293963000000	-0.418876000000	-0.725804000000
C	0.759257000000	-3.782324000000	-2.322622000000
H	0.097390000000	-1.901181000000	-3.180659000000
C	1.105486000000	-3.786991000000	0.078934000000
H	0.693890000000	-1.916540000000	1.102642000000
S	-4.942345000000	-1.083457000000	0.528230000000
C	1.124544000000	-4.466878000000	-1.151809000000
H	0.774523000000	-4.299670000000	-3.288487000000
H	1.393949000000	-4.310492000000	0.997470000000
H	1.423323000000	-5.519424000000	-1.196975000000
C	0.588408000000	4.557693000000	-0.886724000000
H	2.043166000000	3.022704000000	-0.469947000000
C	-1.514170000000	3.816627000000	-1.831679000000
H	-1.688863000000	1.689180000000	-2.205609000000
C	-0.695956000000	4.859917000000	-1.367546000000
H	1.243109000000	5.359360000000	-0.527451000000
H	-2.507705000000	4.035950000000	-2.238452000000
C	-5.402906000000	0.291504000000	1.653001000000
C	-6.634300000000	-1.504234000000	-0.044216000000
H	-5.970431000000	1.047845000000	1.088631000000
H	-5.995752000000	-0.114304000000	2.488704000000
H	-4.458761000000	0.717096000000	2.024777000000

H -7.227272000000 -1.870903000000 0.808846000000

H -7.095084000000 -0.612342000000 -0.496080000000

H -6.527839000000 -2.298307000000 -0.797398000000

C -1.771349000000 -0.826885000000 2.212106000000

C -1.775714000000 1.539330000000 1.634815000000

C -1.778949000000 -0.491577000000 3.580946000000

H -1.748825000000 -1.881102000000 1.915589000000

C -1.784084000000 1.865328000000 3.005225000000

H -1.755657000000 2.337880000000 0.886844000000

C -1.789514000000 0.854092000000 3.981292000000

H -1.769091000000 -1.289910000000 4.332499000000

H -1.777082000000 2.919391000000 3.306818000000

H -1.789638000000 1.113835000000 5.045455000000

H -1.048334000000 5.896488000000 -1.395702000000

C 2.203865000000 0.535527000000 -1.549398000000

H 2.324717000000 -0.379613000000 -2.156437000000

H 2.584697000000 1.372539000000 -2.163923000000

C 3.083953000000 0.386648000000 -0.284663000000

H 2.943998000000 1.258789000000 0.382085000000

H 2.735316000000 -0.492679000000 0.287271000000

C 4.576395000000 0.228653000000 -0.628315000000

H 4.928165000000 1.106592000000 -1.202592000000

H 4.719659000000 -0.652869000000 -1.280906000000

C 5.440139000000 0.073005000000 0.618605000000

H 5.162094000000 -0.819905000000 1.206983000000

H 5.360069000000 0.945697000000 1.291449000000

O 6.825023000000 -0.060992000000 0.183847000000

C 7.738744000000 -0.176094000000 1.191503000000

O 7.448889000000 -0.164562000000 2.379798000000

C 9.137445000000 -0.331940000000 0.622898000000

H 9.227848000000 -1.306203000000 0.113428000000

H 9.342600000000 0.448838000000 -0.126729000000

H 9.870260000000 -0.278253000000 1.438689000000

$^4TS_{KL}$

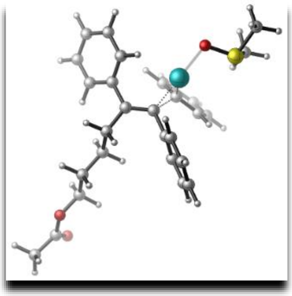

Frequency -266.3438

Zero-point correction= 0.514590 (Hartree/Particle)

Thermal correction to Energy= 0.550787

Thermal correction to Enthalpy= 0.551732

Thermal correction to Gibbs Free Energy= 0.437877

Sum of electronic and zero-point Energies= -1836.374101

Sum of electronic and thermal Energies= -1836.337903

Sum of electronic and thermal Enthalpies= -1836.336959

Sum of electronic and thermal Free Energies= -1836.450813

E(RM06L) = -1838.43805155

Atom	X	Y	Z
C	-1.276771000000	0.106506000000	1.316779000000
C	-0.260995000000	-0.119952000000	-0.418578000000
C	0.572812000000	0.907568000000	-0.819058000000
C	0.180635000000	2.341811000000	-0.799846000000
C	1.098641000000	3.345773000000	-0.394127000000
C	-1.100753000000	2.775199000000	-1.226023000000
C	0.147385000000	-1.548424000000	-0.614577000000
Pd	-2.303019000000	0.040848000000	-0.449214000000
C	-0.185124000000	-2.197792000000	-1.830477000000
C	0.869255000000	-2.288989000000	0.351557000000
O	-4.411507000000	-0.018049000000	-1.065235000000
C	0.190002000000	-3.527005000000	-2.070532000000
H	-0.745950000000	-1.637262000000	-2.586469000000

C 1.239809000000 -3.623201000000 0.112800000000

H 1.128083000000 -1.813216000000 1.302288000000

S -5.017088000000 -1.303378000000 -0.407742000000

C 0.902641000000 -4.249036000000 -1.096985000000

H -0.074998000000 -4.001009000000 -3.022114000000

H 1.797536000000 -4.173564000000 0.878724000000

H 1.192984000000 -5.288734000000 -1.281633000000

C 0.737186000000 4.699426000000 -0.364735000000

H 2.103339000000 3.059393000000 -0.069017000000

C -1.467645000000 4.128701000000 -1.190056000000

H -1.806758000000 2.024128000000 -1.608417000000

C -0.552002000000 5.099778000000 -0.755427000000

H 1.465810000000 5.445601000000 -0.029252000000

H -2.467551000000 4.424719000000 -1.525754000000

C -5.267171000000 -0.918328000000 1.369898000000

C -6.791005000000 -1.227576000000 -0.905494000000

H -5.800617000000 0.040460000000 1.463818000000

H -5.836771000000 -1.740758000000 1.832745000000

H -4.263398000000 -0.843594000000 1.816528000000

H -7.346081000000 -2.035110000000 -0.402268000000

H -7.197124000000 -0.238768000000 -0.642076000000

H -6.820941000000 -1.371844000000 -1.995178000000

C -1.375786000000 -1.076236000000 2.095946000000

C -1.158179000000 1.345546000000 1.997403000000

C -1.368719000000 -1.018360000000 3.502017000000

H -1.444104000000 -2.048448000000 1.598600000000

C -1.141953000000 1.391818000000 3.399331000000

H -1.079988000000 2.275480000000 1.429173000000

C -1.248501000000 0.214421000000 4.161734000000

H -1.455038000000 -1.946644000000 4.079132000000

H -1.048418000000 2.362158000000 3.900673000000

H -1.233846000000 0.259303000000 5.255883000000

H -0.832281000000 6.158335000000 -0.737053000000

C 1.987262000000 0.610064000000 -1.333714000000

H 1.963635000000 -0.304620000000 -1.952791000000

H 2.302932000000 1.432580000000 -2.001745000000

C 3.064082000000 0.412647000000 -0.237251000000

H 3.090353000000 1.288353000000 0.438271000000

H 2.779542000000 -0.449087000000 0.391946000000

C 4.463163000000 0.176525000000 -0.835336000000

H 4.761786000000 1.040538000000 -1.458477000000

H 4.439904000000 -0.703266000000 -1.505162000000

C 5.514772000000 -0.048030000000 0.246290000000

H 5.284690000000 -0.929662000000 0.870592000000

H 5.602750000000 0.819544000000 0.925195000000

O 6.795314000000 -0.259673000000 -0.416087000000

C 7.841529000000 -0.521908000000 0.421328000000

O 7.740255000000 -0.587199000000 1.638528000000

C 9.124571000000 -0.695945000000 -0.370404000000

H 9.456050000000 0.281630000000 -0.760195000000

H 9.904342000000 -1.104837000000 0.285663000000

H 8.965078000000 -1.357412000000 -1.236360000000

^{4}L

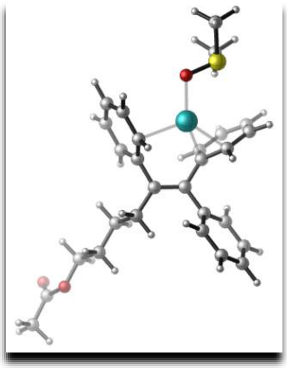

Zero-point correction= 0.515491 (Hartree/Particle)

Thermal correction to Energy= 0.552179

Thermal correction to Enthalpy= 0.553123

Thermal correction to Gibbs Free Energy= 0.438262

Sum of electronic and zero-point Energies= -1836.412427

Sum of electronic and thermal Energies= -1836.375739

Sum of electronic and thermal Enthalpies= -1836.374795

Sum of electronic and thermal Free Energies= -1836.489656

E(RM06L) = -1838.47903249

**Atom**	**X**	**Y**	**Z**
C	1.105807000000	1.059918000000	0.655215000000
C	-0.219394000000	1.170289000000	-0.071463000000
C	-0.971932000000	0.067016000000	-0.379738000000
C	-0.353881000000	-1.293850000000	-0.264124000000
C	-0.691167000000	-2.226967000000	0.736515000000
C	0.629576000000	-1.661250000000	-1.238258000000
C	-0.658267000000	2.575459000000	-0.342337000000
Pd	2.359701000000	-0.393088000000	-0.336004000000
C	-1.034769000000	2.992271000000	-1.640600000000
C	-0.645968000000	3.541972000000	0.691053000000
O	4.211092000000	-1.486295000000	-1.161885000000
C	-1.414270000000	4.320064000000	-1.891260000000
H	-1.008130000000	2.265882000000	-2.459923000000
C	-1.031809000000	4.866207000000	0.442327000000
H	-0.329953000000	3.241431000000	1.695742000000
S	5.391322000000	-0.729027000000	-0.491294000000
C	-1.420325000000	5.261022000000	-0.849407000000
H	-1.697021000000	4.621130000000	-2.905899000000
H	-1.025565000000	5.595374000000	1.259810000000
H	-1.715583000000	6.297485000000	-1.044034000000
C	-0.072965000000	-3.490416000000	0.787351000000
H	-1.418014000000	-1.950336000000	1.507015000000
C	1.241513000000	-2.938631000000	-1.181001000000
H	0.749150000000	-1.036687000000	-2.134497000000
C	0.900162000000	-3.842937000000	-0.160579000000
H	-0.346398000000	-4.194139000000	1.580994000000
H	1.972357000000	-3.215205000000	-1.946900000000
C	5.381174000000	-1.177841000000	1.291135000000
C	6.879028000000	-1.723935000000	-0.951984000000
H	5.308530000000	-2.272231000000	1.391918000000
H	6.300118000000	-0.792693000000	1.763176000000
H	4.493826000000	-0.687786000000	1.722457000000
H	7.765021000000	-1.322390000000	-0.434572000000
H	6.707046000000	-2.778276000000	-0.685598000000
H	7.002816000000	-1.625314000000	-2.040259000000
C	2.286024000000	1.763884000000	0.177522000000
C	1.107652000000	0.678587000000	2.050364000000
C	3.330170000000	2.123094000000	1.086444000000
H	2.245652000000	2.268005000000	-0.795398000000
C	2.142664000000	1.041208000000	2.911222000000
H	0.235313000000	0.142200000000	2.437373000000
C	3.254541000000	1.785443000000	2.437626000000
H	4.174284000000	2.711711000000	0.709018000000
H	2.083809000000	0.760966000000	3.968973000000
H	4.043461000000	2.096189000000	3.131059000000
H	1.383045000000	-4.824924000000	-0.116014000000
C	-2.398615000000	0.132082000000	-0.909424000000
H	-2.681423000000	1.184277000000	-1.077206000000
H	-2.442275000000	-0.371558000000	-1.896650000000
C	-3.462039000000	-0.518665000000	0.007321000000
H	-3.235606000000	-1.592056000000	0.141748000000
H	-3.407541000000	-0.054689000000	1.011079000000
C	-4.885368000000	-0.362897000000	-0.559196000000
H	-4.942225000000	-0.819860000000	-1.565035000000
H	-5.129060000000	0.708597000000	-0.684271000000
C	-5.930354000000	-1.010925000000	0.343310000000
H	-5.941020000000	-0.562909000000	1.353159000000
H	-5.754039000000	-2.094620000000	0.468266000000
O	-7.234355000000	-0.813197000000	-0.274563000000
C	-8.289052000000	-1.315134000000	0.433921000000

O -8.175385000000 -1.890111000000 1.507332000000

C -9.591513000000 -1.058053000000 -0.300974000000

H -9.720573000000 0.020635000000 -0.487353000000

H -9.579052000000 -1.559101000000 -1.283088000000

H -10.428861000000 -1.437861000000 0.298810000000

^{4}M

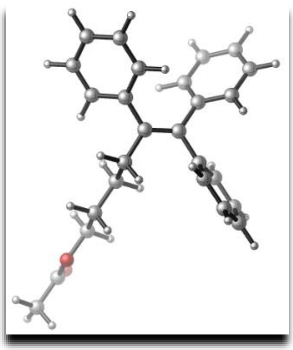

Zero-point correction= 0.438725 (Hartree/Particle)

Thermal correction to Energy= 0.465587

Thermal correction to Enthalpy= 0.466531

Thermal correction to Gibbs Free Energy= 0.375958

Sum of electronic and zero-point Energies= -1156.426475

Sum of electronic and thermal Energies= -1156.399614

Sum of electronic and thermal Enthalpies= -1156.398670

Sum of electronic and thermal Free Energies= -1156.489243

E(RM06L) = -1157.17984716

Atom X Y Z

C 2.726511000000 0.644766000000 0.533503000000

C 1.460598000000 0.586008000000 -0.260706000000

C 0.865243000000 -0.582315000000 -0.679103000000

C 1.556552000000 -1.904646000000 -0.648774000000

C 0.872016000000 -3.086520000000 -0.273969000000

C 2.896759000000 -2.033424000000 -1.089537000000

C 0.836078000000 1.924626000000 -0.529868000000

C 0.645033000000 2.385110000000 -1.853051000000

C 0.480817000000 2.783844000000 0.535322000000

C 0.094483000000 3.651648000000 -2.103103000000

H 0.948874000000 1.743646000000 -2.687781000000

C -0.078862000000 4.045591000000 0.286064000000

H 0.648178000000 2.451494000000 1.565393000000

C -0.275666000000 4.483932000000 -1.034206000000

H -0.038354000000 3.991554000000 -3.135893000000

H -0.357580000000 4.691007000000 1.125969000000

H -0.706244000000 5.471809000000 -1.228456000000

C 1.508512000000 -4.335967000000 -0.305256000000

H -0.168716000000 -3.027061000000 0.061287000000

C 3.529658000000 -3.281972000000 -1.130375000000

H 3.435654000000 -1.138461000000 -1.413717000000

C 2.841440000000 -4.440802000000 -0.732810000000

H 0.957955000000 -5.231310000000 0.003122000000

H 4.564101000000 -3.351499000000 -1.483645000000

C 3.705201000000 1.628121000000 0.252933000000

C 2.949538000000 -0.224132000000 1.628337000000

C 4.880157000000 1.713633000000 1.013375000000

H 3.538353000000 2.325114000000 -0.575031000000

C 4.118628000000 -0.130646000000 2.394753000000

H 2.190914000000 -0.972449000000 1.875961000000

C 5.093042000000 0.834131000000 2.087477000000

H 5.629562000000 2.474316000000 0.769216000000

H 4.266148000000 -0.809625000000 3.241481000000

H 6.006278000000 0.906609000000 2.687430000000

H 3.336589000000 -5.416892000000 -0.765182000000

C -0.576245000000 -0.596922000000 -1.192625000000

H -0.745347000000 0.241551000000 -1.888591000000

H -0.749066000000 -1.522600000000 -1.769411000000

C -1.624940000000 -0.492642000000 -0.057595000000

H -1.457365000000 -1.295417000000 0.686151000000

H -1.467943000000 0.460113000000 0.481034000000

C -3.069732000000 -0.566298000000 -0.582745000000

H -3.232650000000 -1.522514000000 -1.114593000000

H -3.243134000000 0.239203000000 -1.320550000000

C -4.090618000000 -0.441787000000 0.543919000000

H -4.000655000000 0.521934000000 1.076880000000

H -3.977973000000 -1.242912000000 1.296525000000

O -5.417868000000 -0.533631000000 -0.047645000000

C -6.450272000000 -0.484573000000 0.846526000000

O -6.297728000000 -0.376427000000 2.055003000000

C -7.781896000000 -0.570680000000 0.124577000000

H -7.935428000000 0.333083000000 -0.488878000000

H -7.798718000000 -1.435305000000 -0.558065000000

H -8.590882000000 -0.653423000000 0.862108000000

3.3 X-ray crystallography:

X-ray reflections for **58** were collected on Rigaku Oxford diffractometer using Mo-Kα, radiation. OLEX 2 program were used to solve and refine the data. All non-hydrogen atoms were refined anisotropically, and C−H hydrogens were fixed.[36-40]

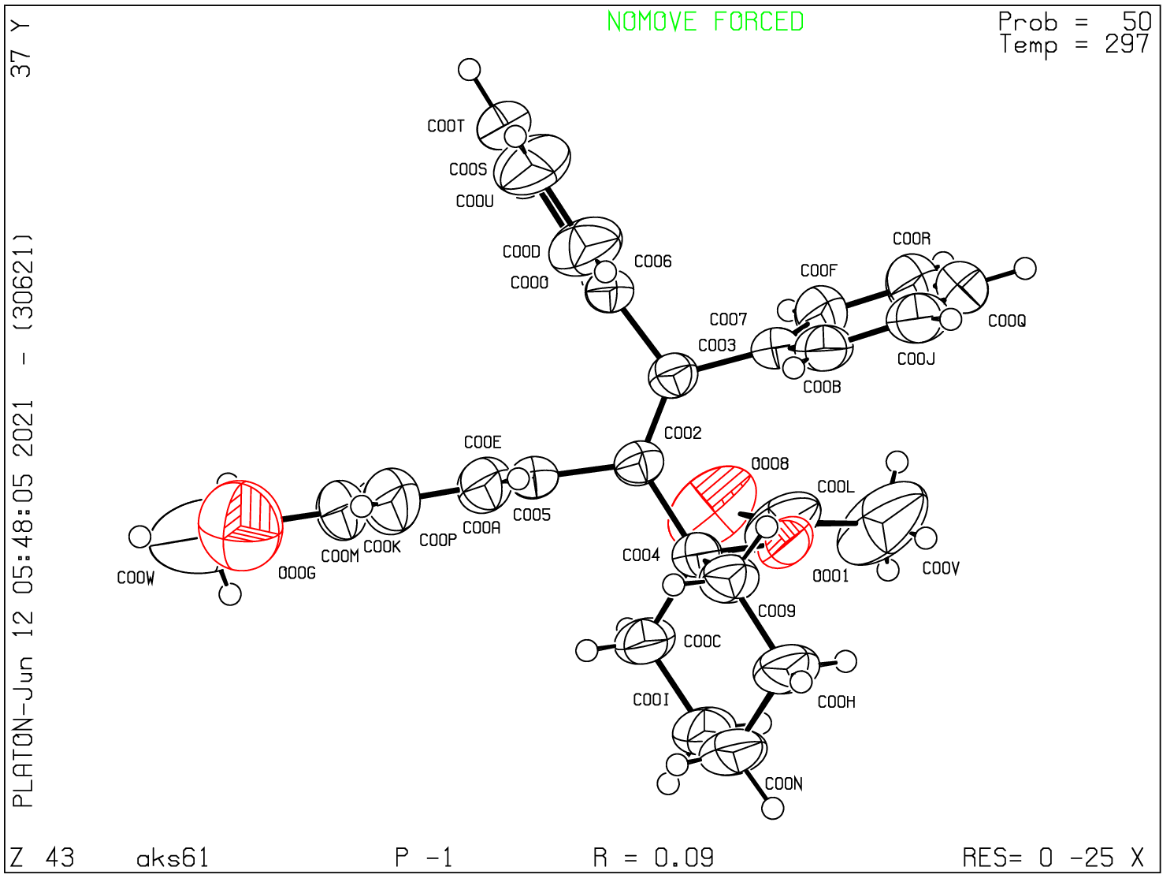

Supplementary Figure 33. Molecular structure of compound **58**, (Oxygen (red))

Supplementary Table 5. Crystallographic Data for Compound **58**

**Compound**	**58**
formula	$C_{29}H_{30}O_3$
Formula weight	426.53
crystal system	triclinic
space group	P -1
T [K]	297 K
a [Å]	9.2741 (7)
b [Å]	9.6365 (5)
c [Å]	15.4637 (7)
α [º]	82.224 (4)
β [º]	89.849 (5)
γ [º]	61.960 (7)
V [$Å^3$]	1205.61(15)
Z	2
ρ_{calcd} [g cm^{-3}]	1.175
μ [mm^{-1}]	0.075
total reflns	10646
unique reflns	4443
observed	3055
R_1[I>2σ(I)]	0.0876 (3055)
wR2 [all]	0.1125 (4443)
GOF	1.085
Diffractometer	Rigaku Oxford
CCDC Number	CCDC 2096145

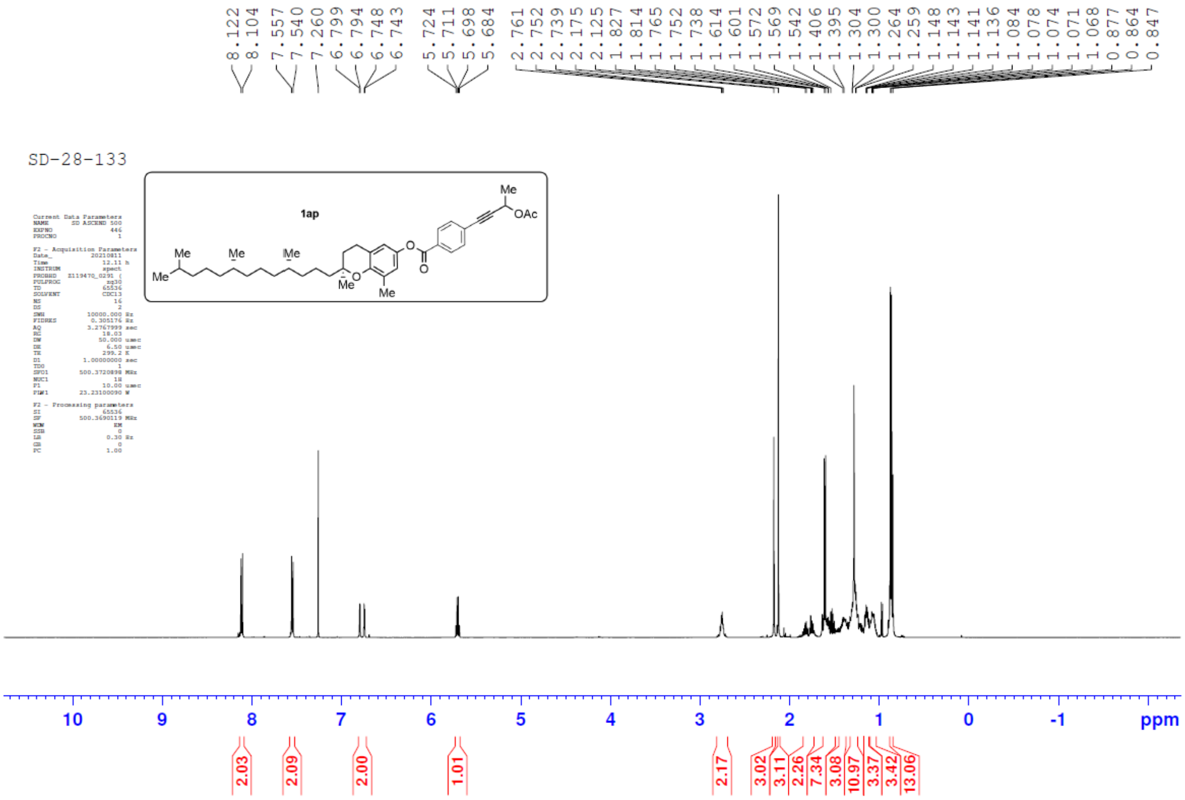

Supplementary Figure 34. ^{1}H NMR of compound **1ap**.

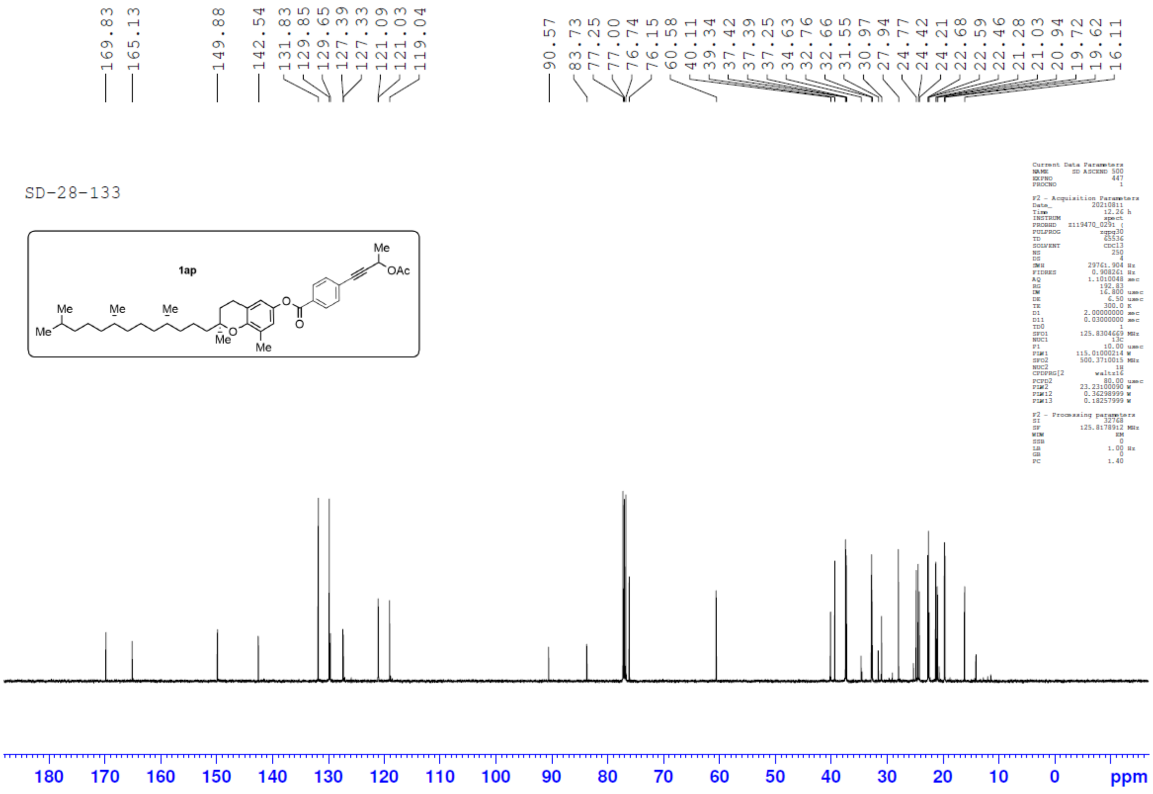

Supplementary Figure 35. ^{13}C NMR of compound **1ap**.

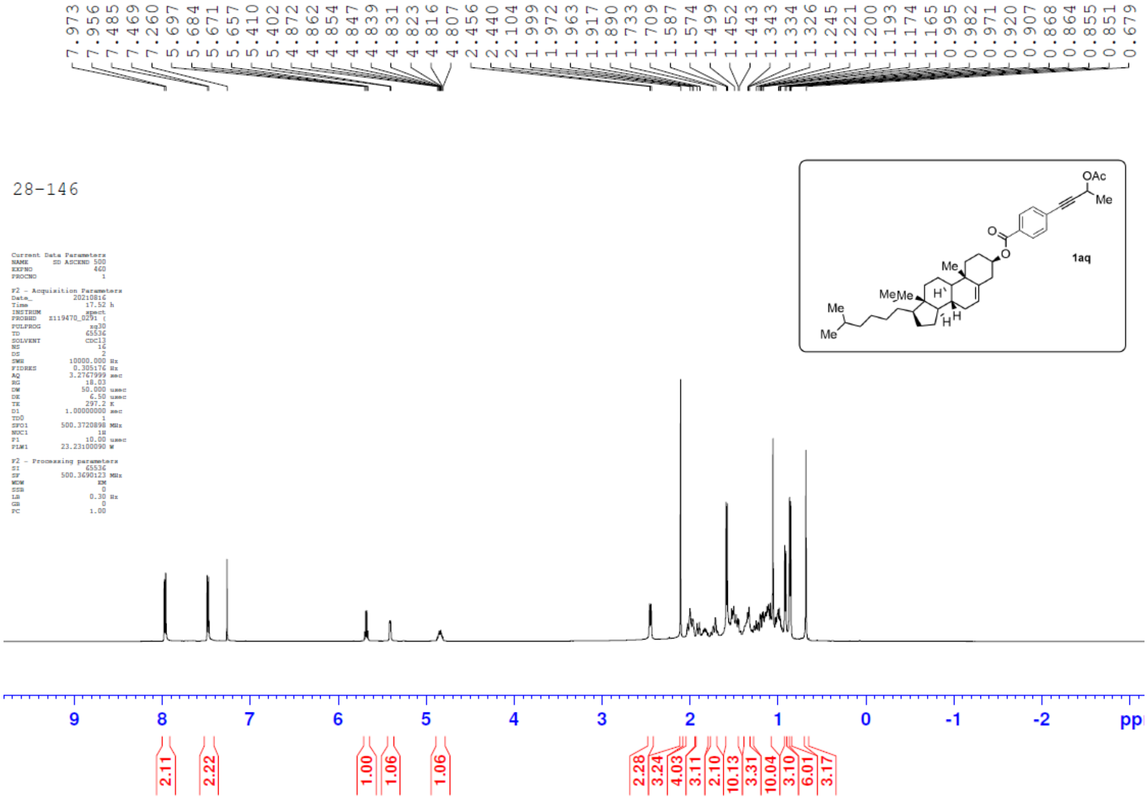

Supplementary Figure 36. ^{1}H NMR of compound **1aq**.

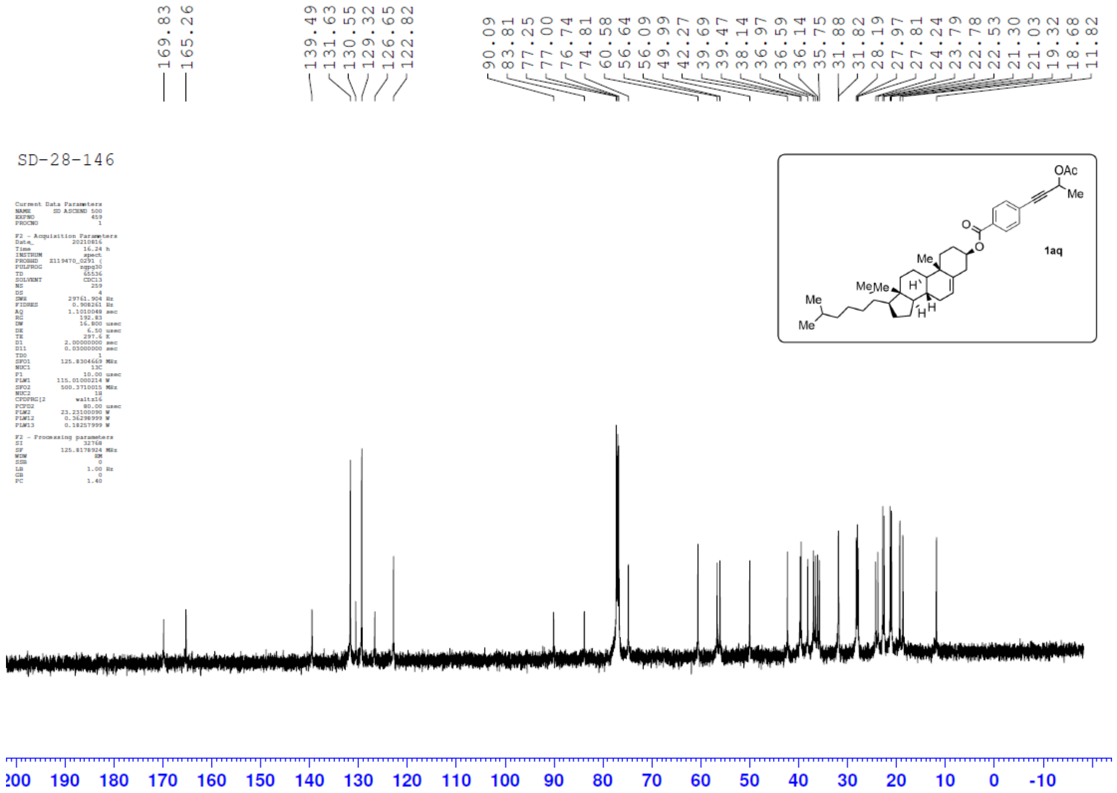

Supplementary Figure 37. ^{13}C NMR of compound **1aq**.

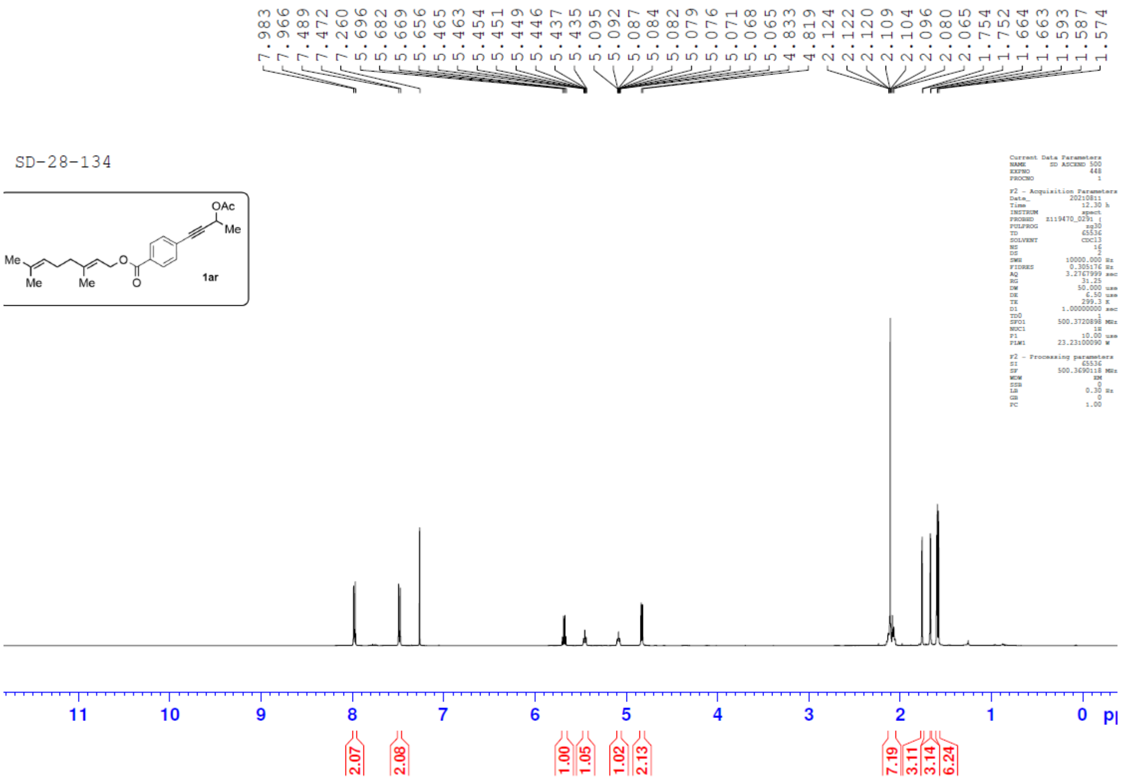

Supplementary Figure 38. ^{1}H NMR of compound **1ar**.

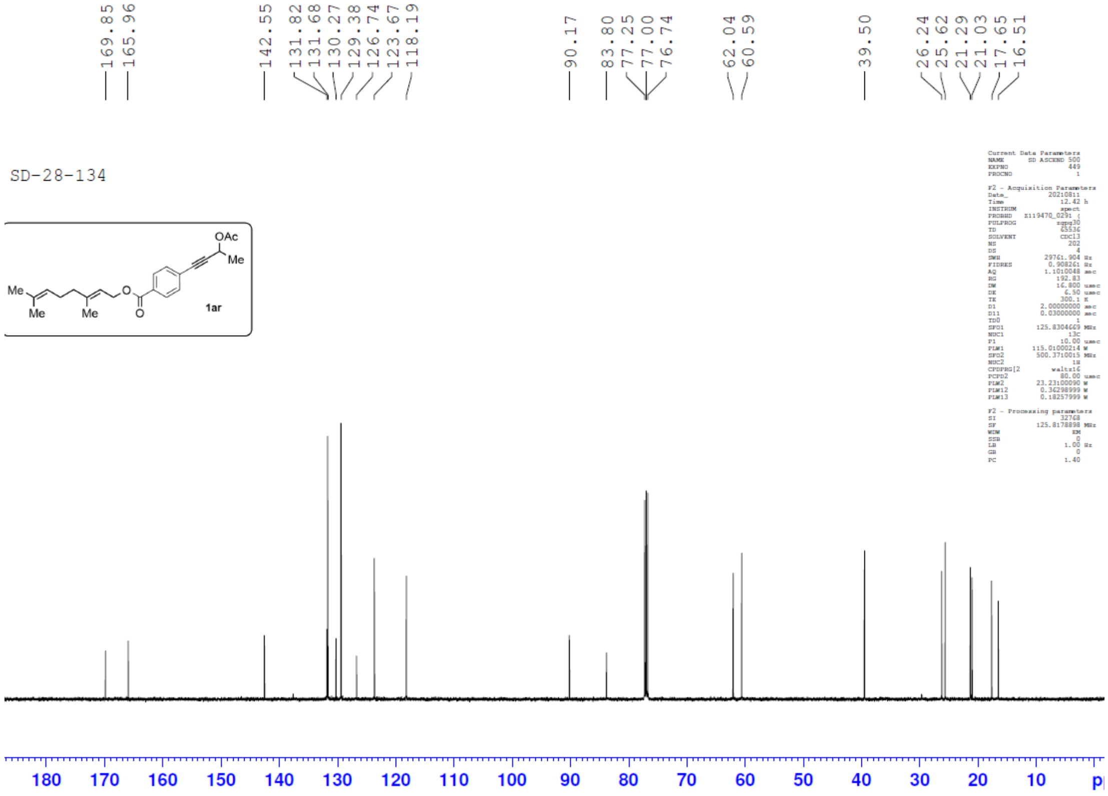

Supplementary Figure 39. ^{13}C NMR of compound **1ar**.

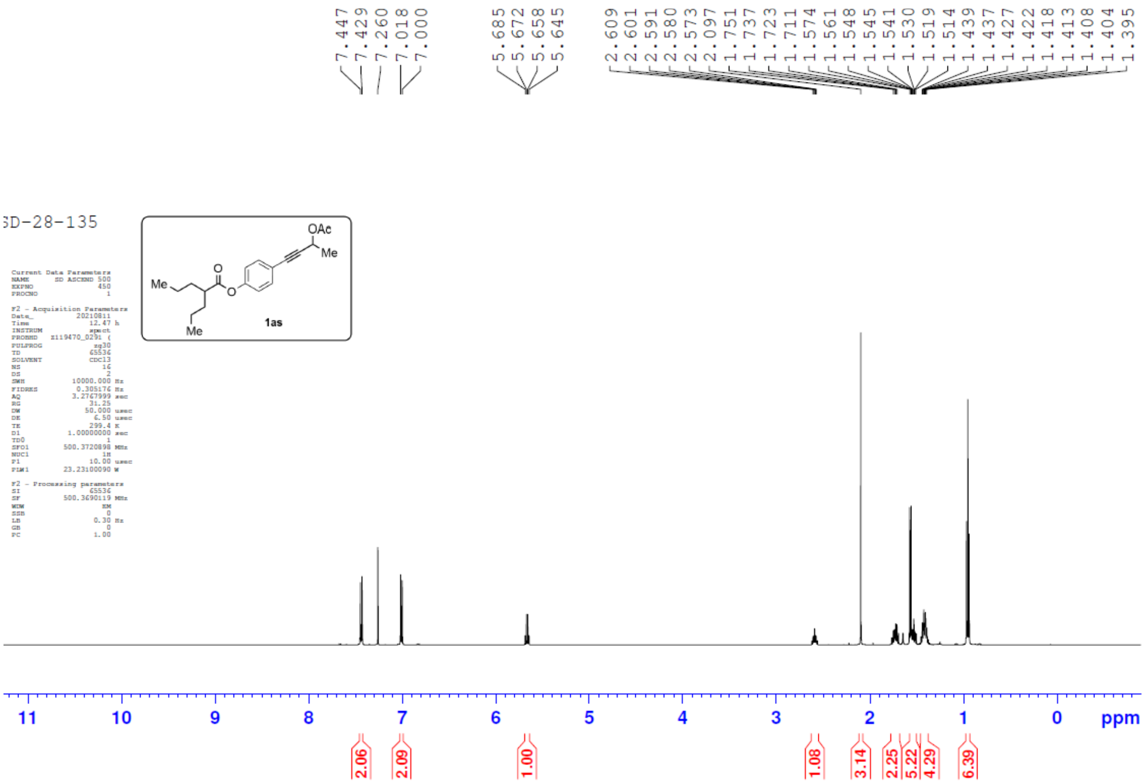

Supplementary Figure 40. ^{1}H NMR of compound **1as**.

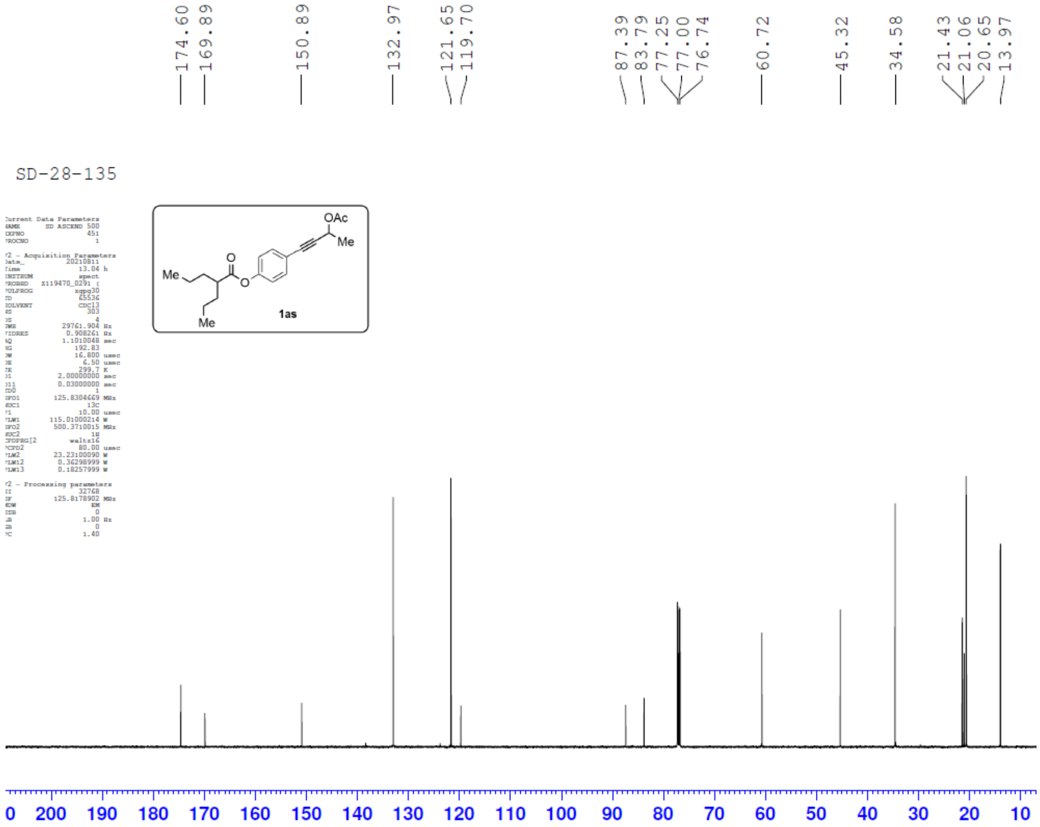

Supplementary Figure 41. ^{13}C NMR of compound **1as**.

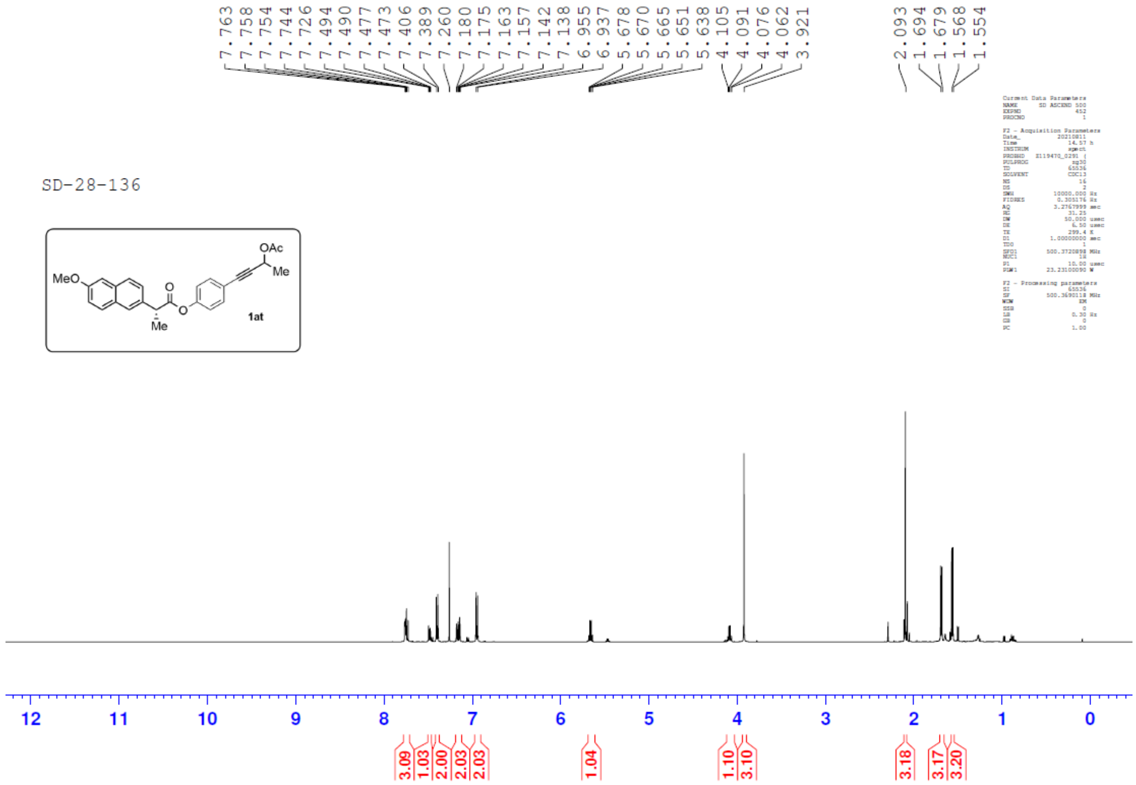

Supplementary Figure 42. ^{1}H NMR of compound **1at**.

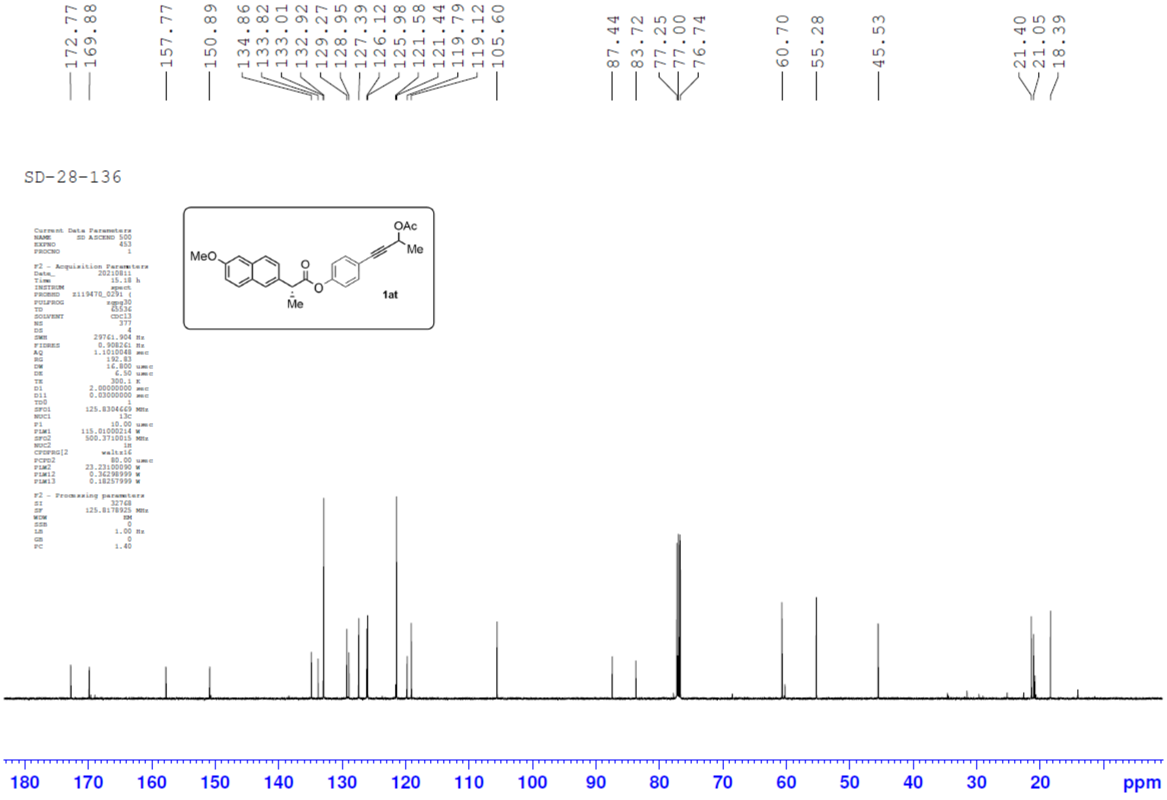

Supplementary Figure 43. ^{13}C NMR of compound **1at**.

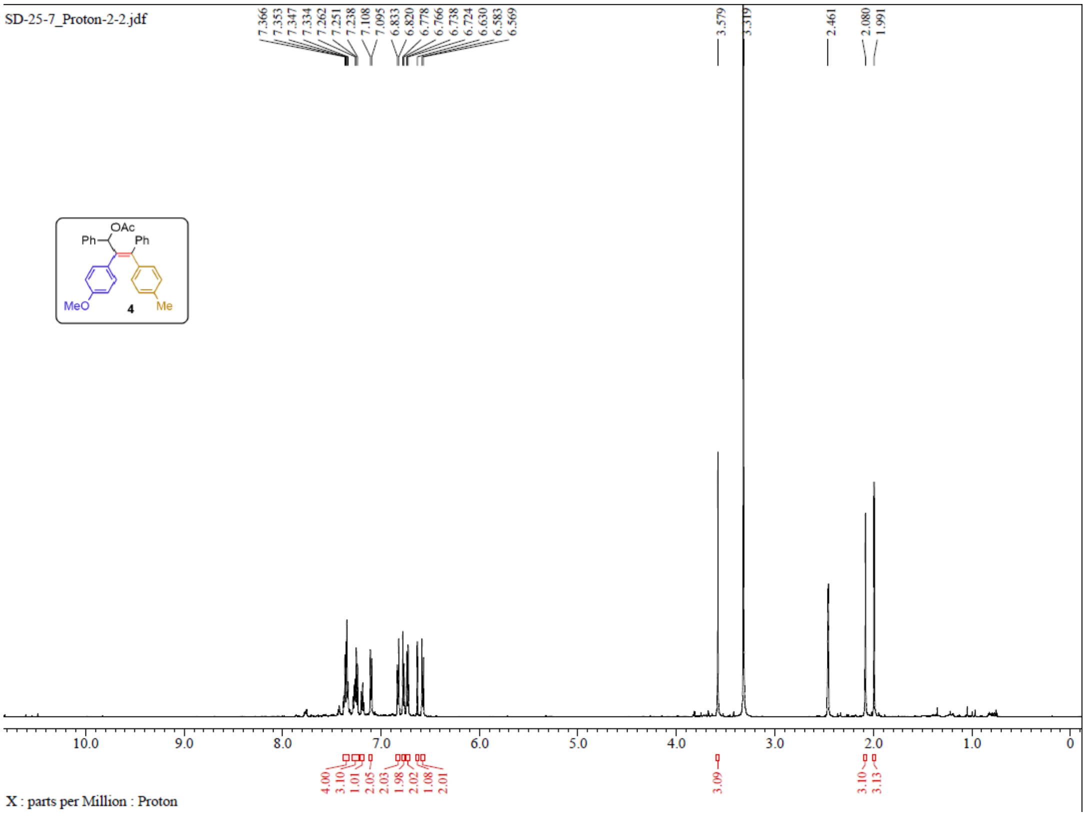

Supplementary Figure 44. ^{1}H NMR of compound **4**.

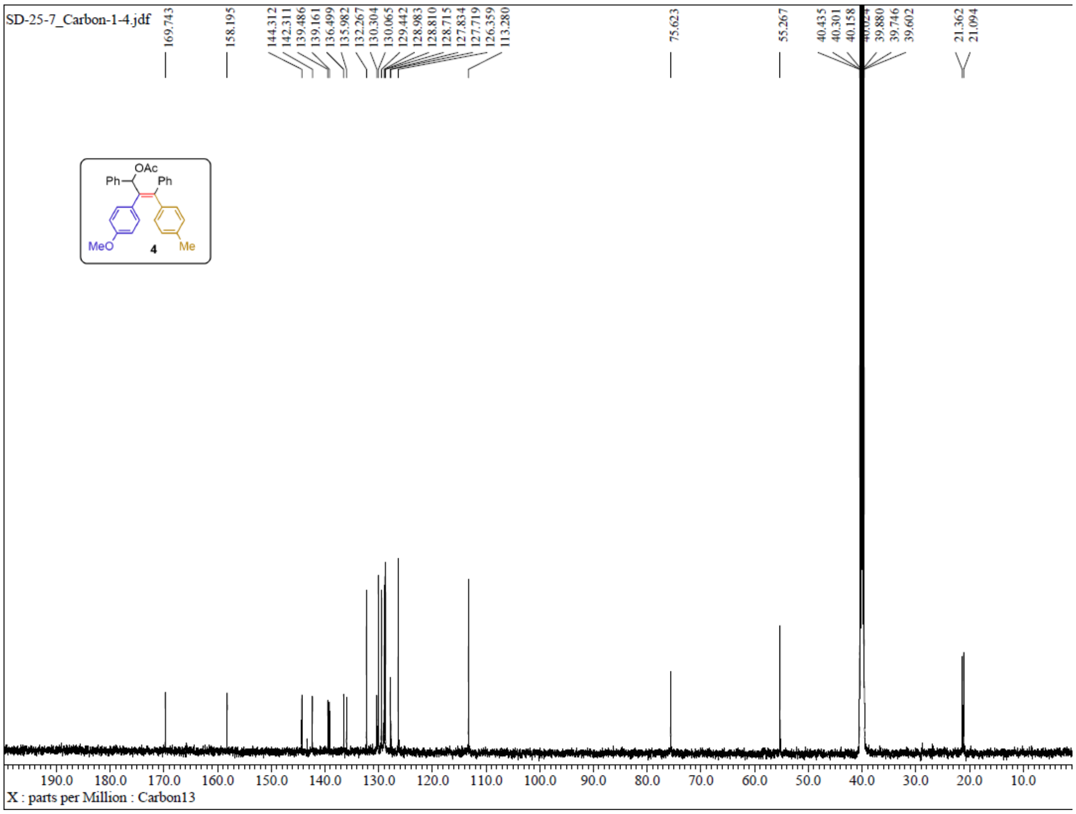

Supplementary Figure 45. ^{13}C NMR of compound **4**.

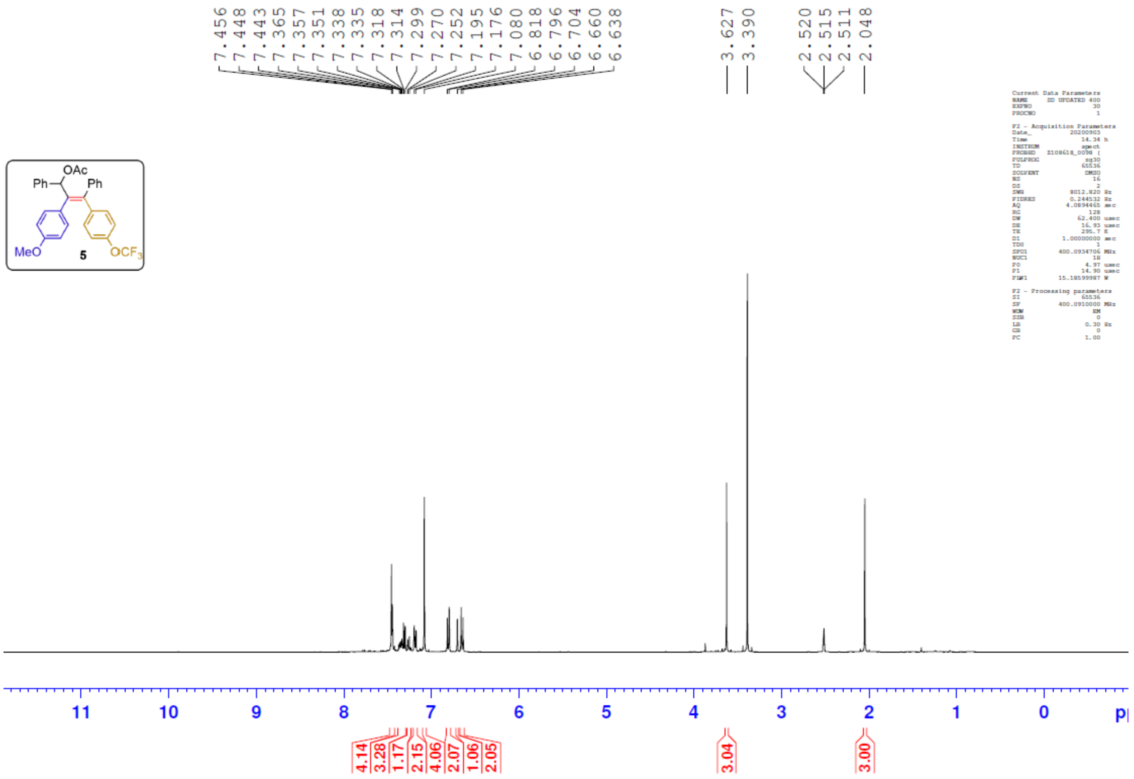

Supplementary Figure 46. ^{1}H NMR of compound **5**.

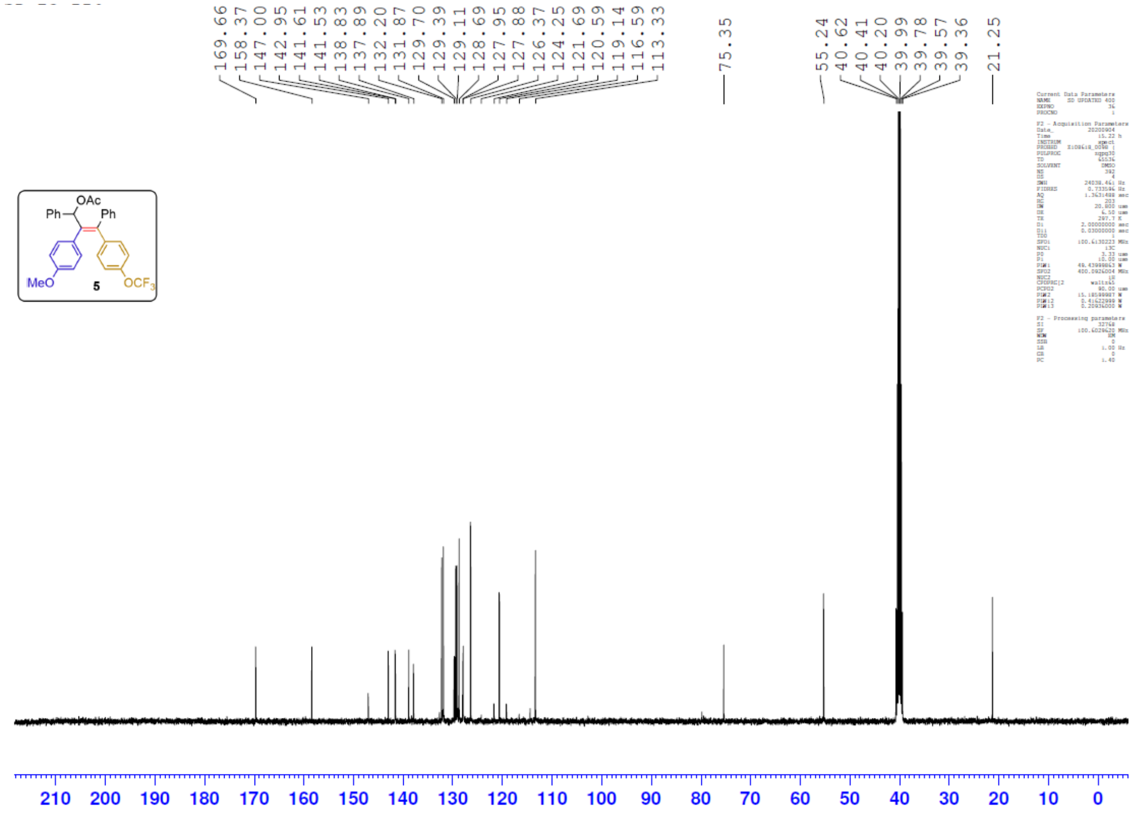

Supplementary Figure 47. ^{13}C NMR of compound **5**.

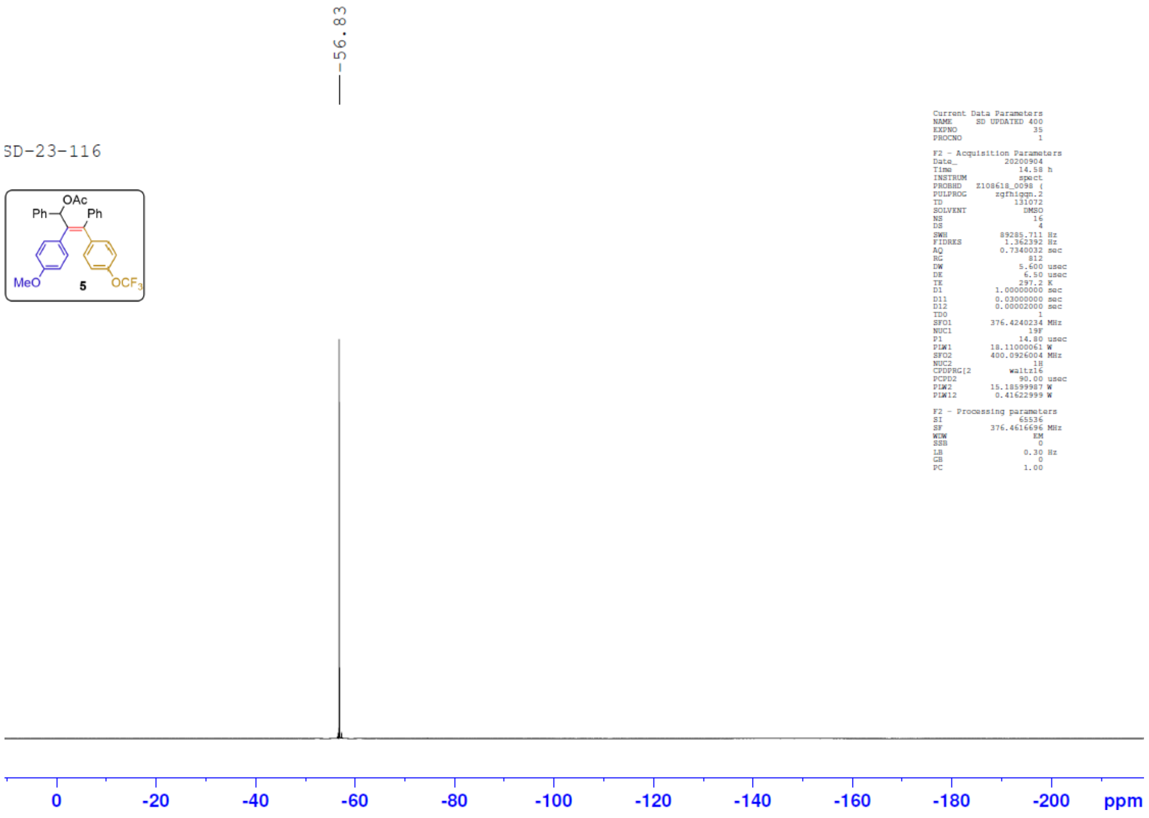

Supplementary Figure 48. ^{19}F NMR of compound **5**.

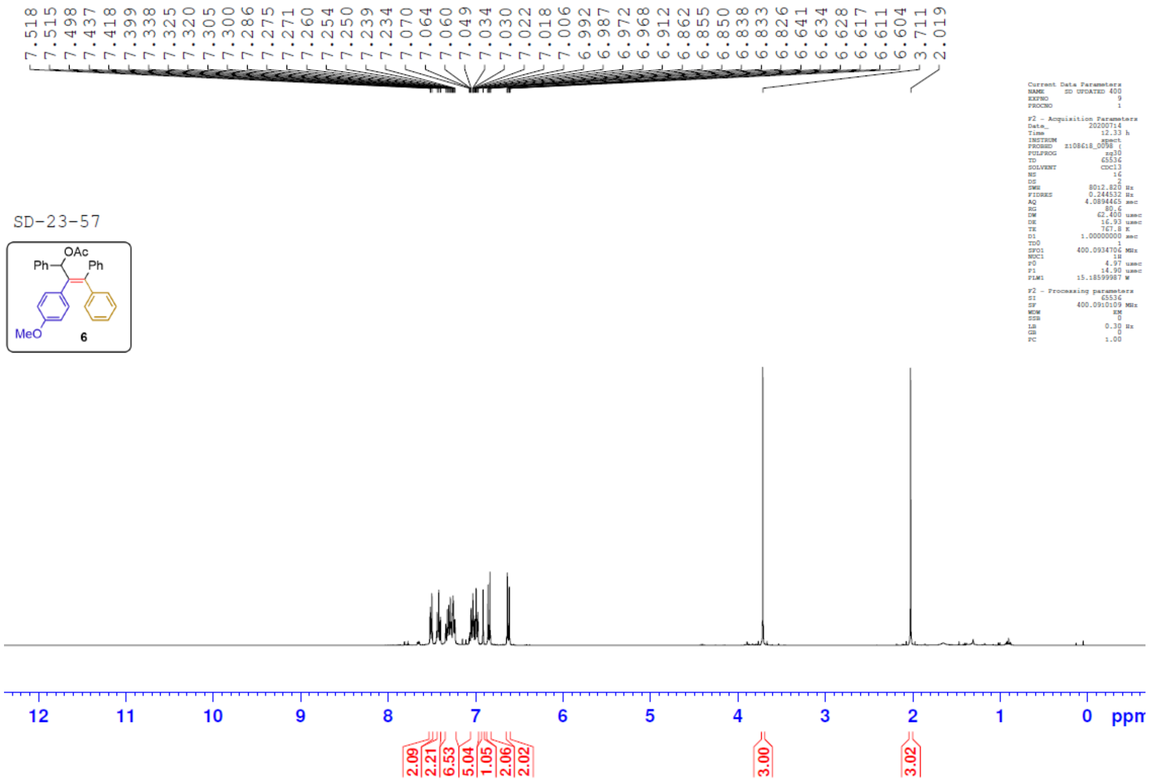

Supplementary Figure 49. ^{1}H NMR of compound **6**.

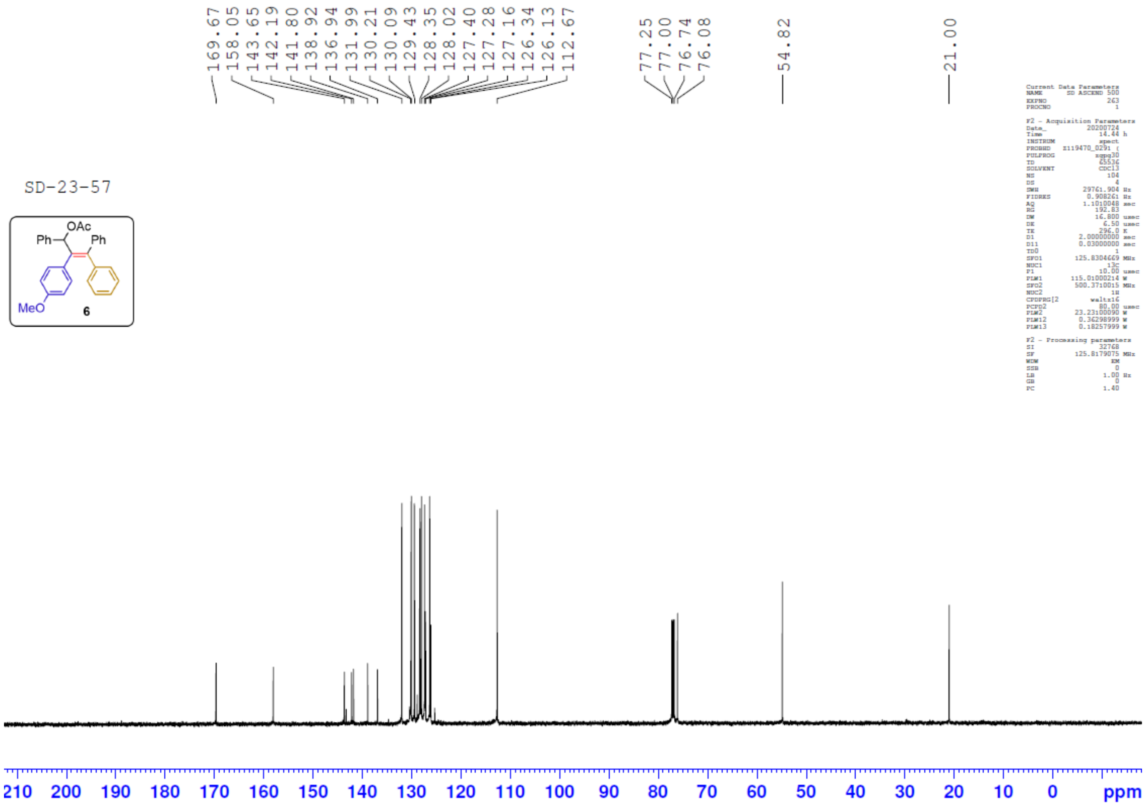

Supplementary Figure 50. ^{13}C NMR of compound **6**.

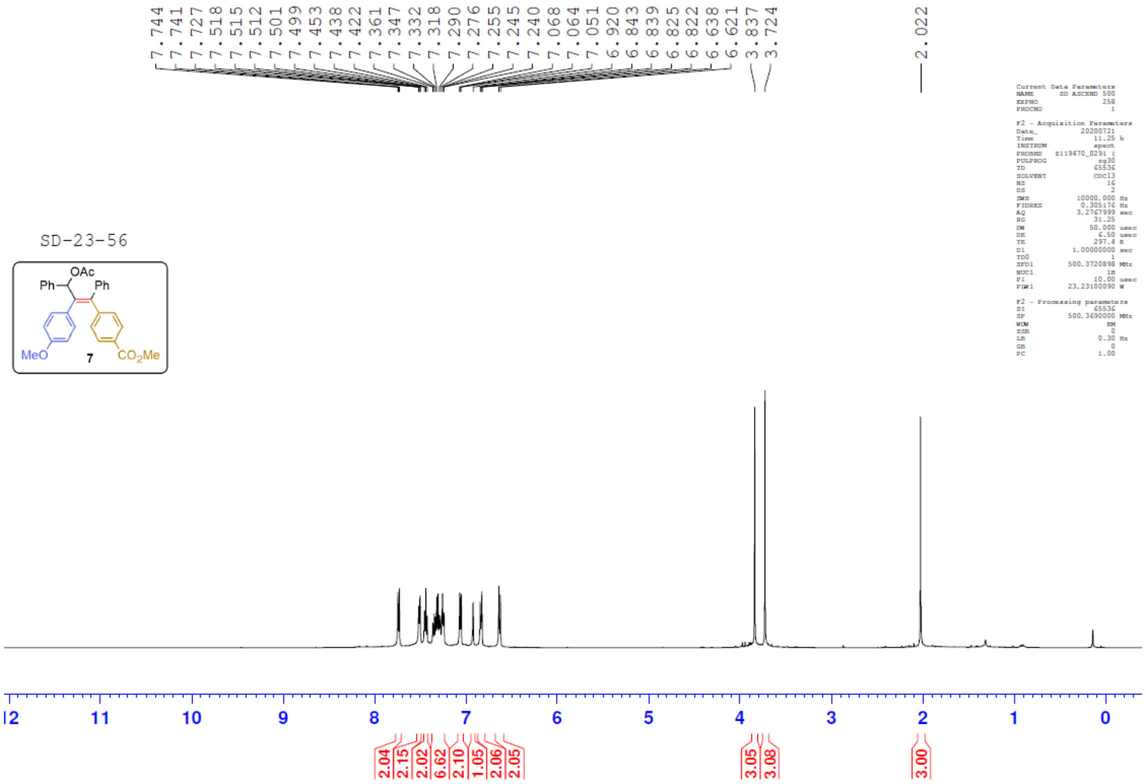

Supplementary Figure 51. ^{1}H NMR of compound **7**.

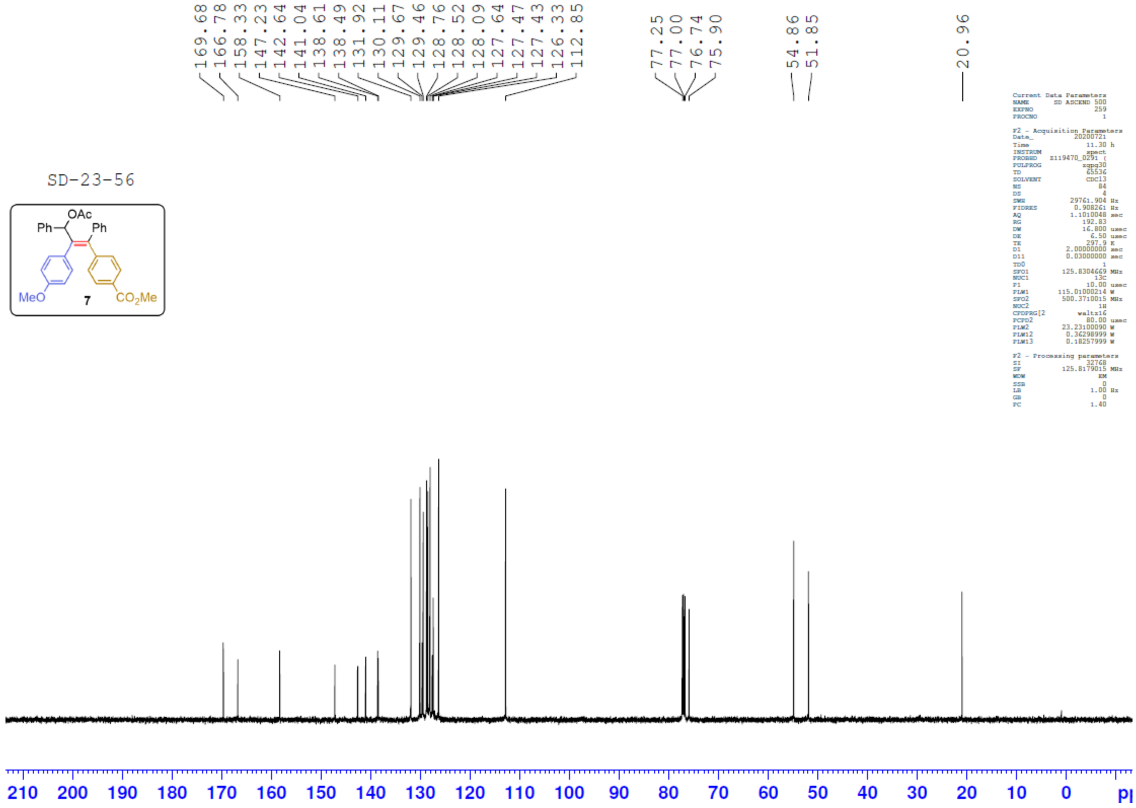

Supplementary Figure 52. ^{13}C NMR of compound **7**.

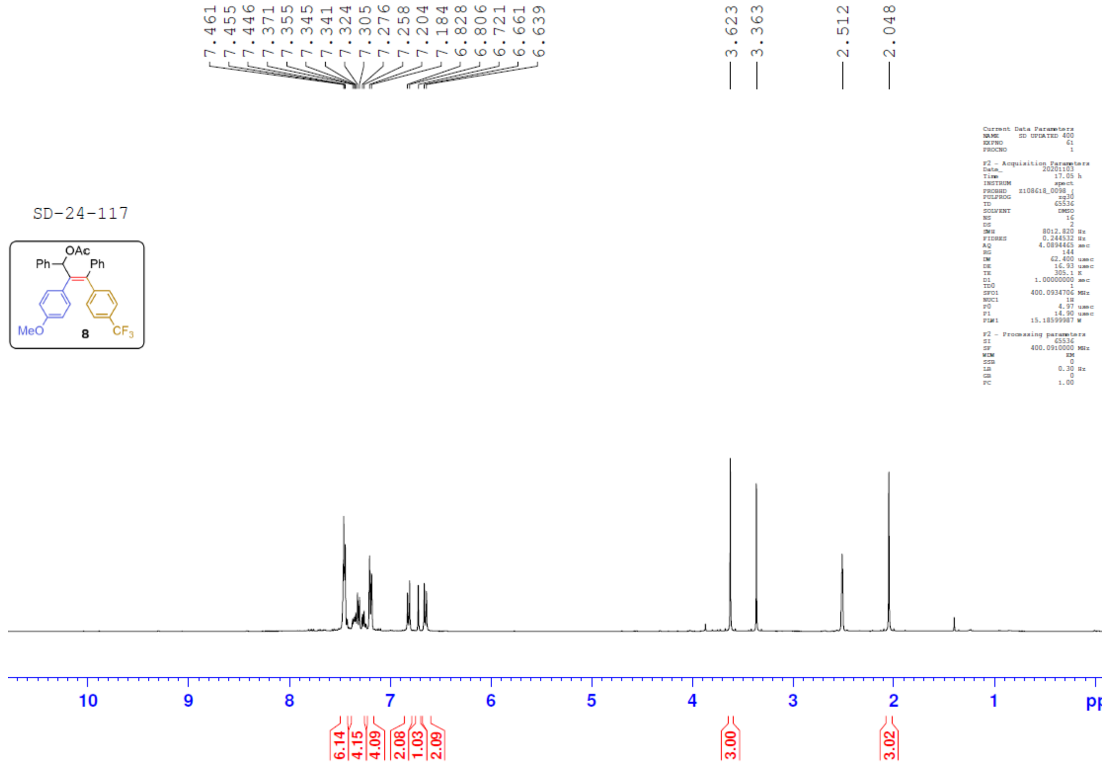

Supplementary Figure 53. ^{1}H NMR of compound **8**.

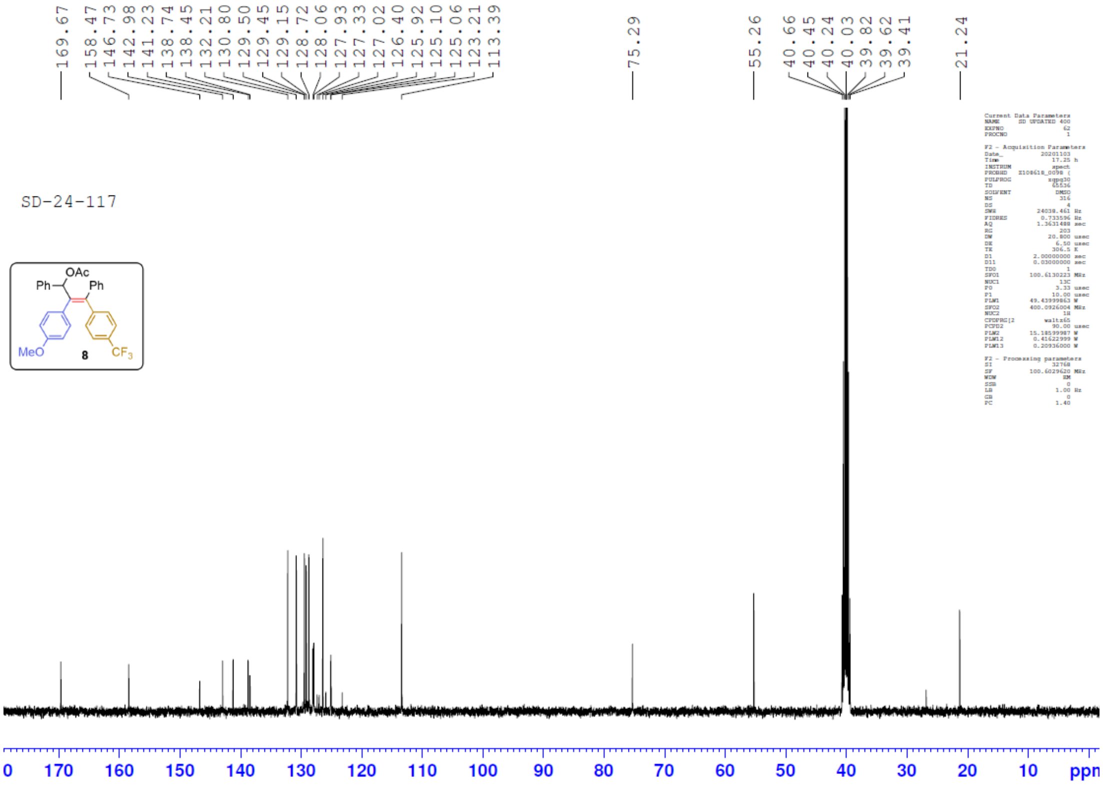

Supplementary Figure 54. ^{13}C NMR of compound **8**.

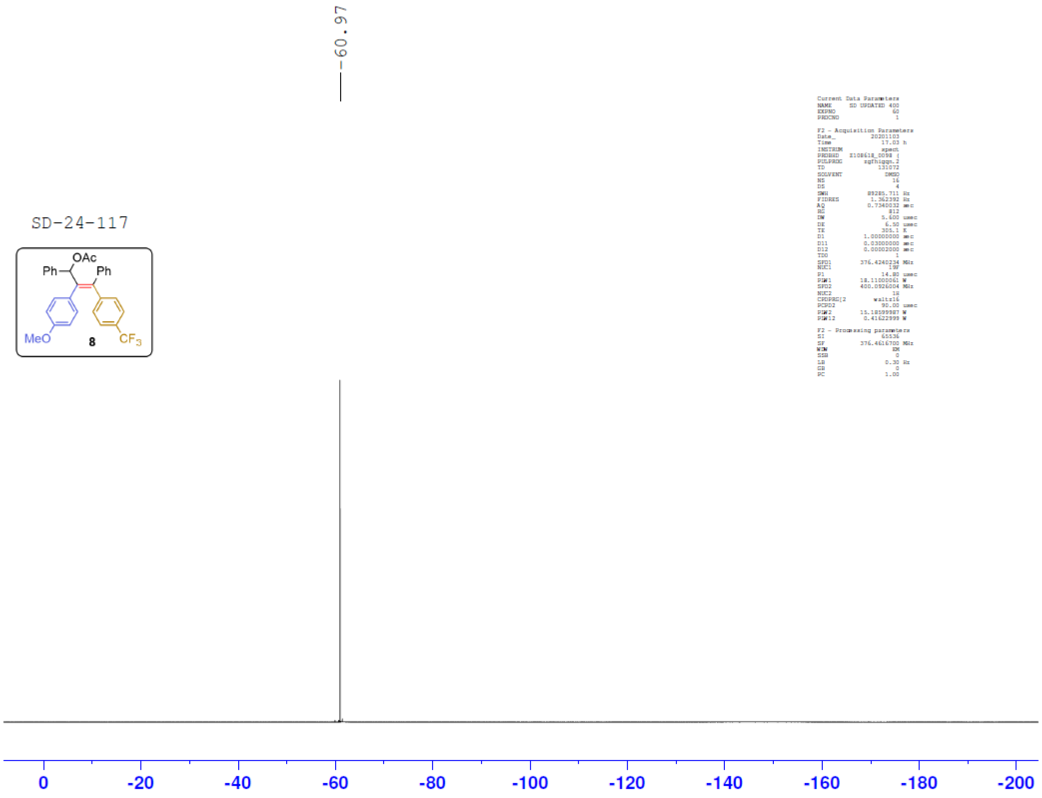

Supplementary Figure 55. ^{19}F NMR of compound **8**.

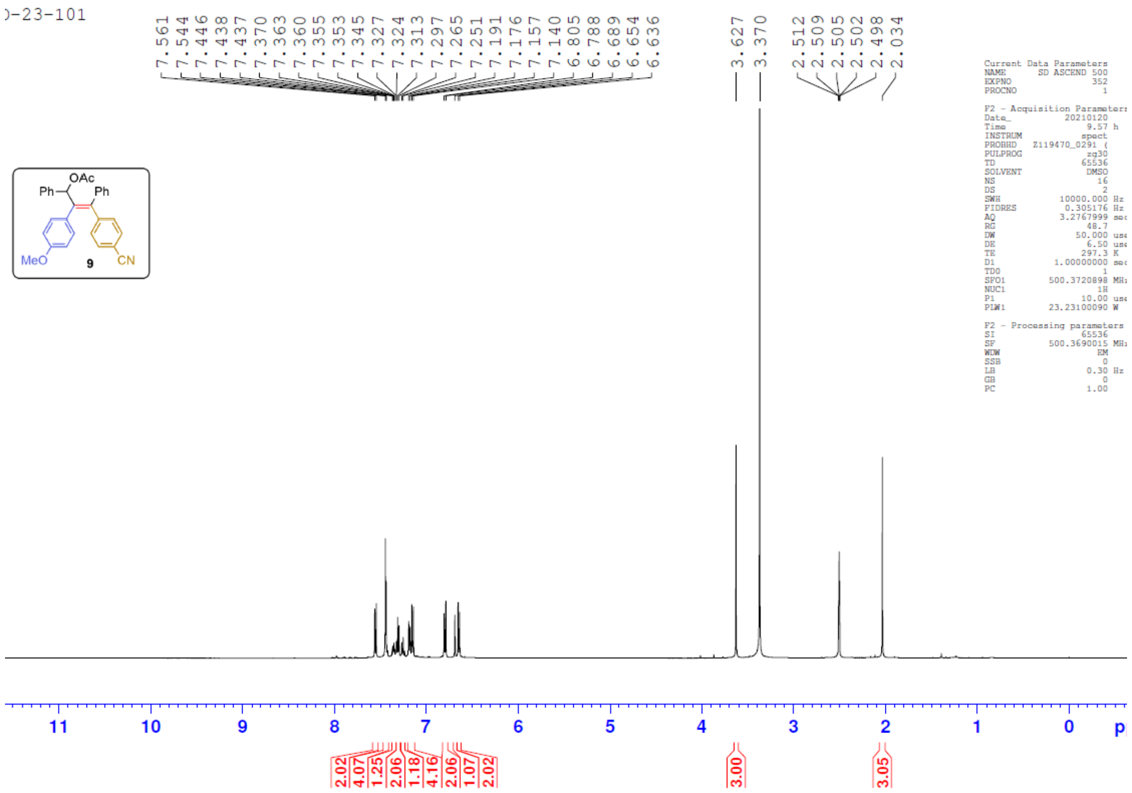

Supplementary Figure 56. ^{1}H NMR of compound **9**.

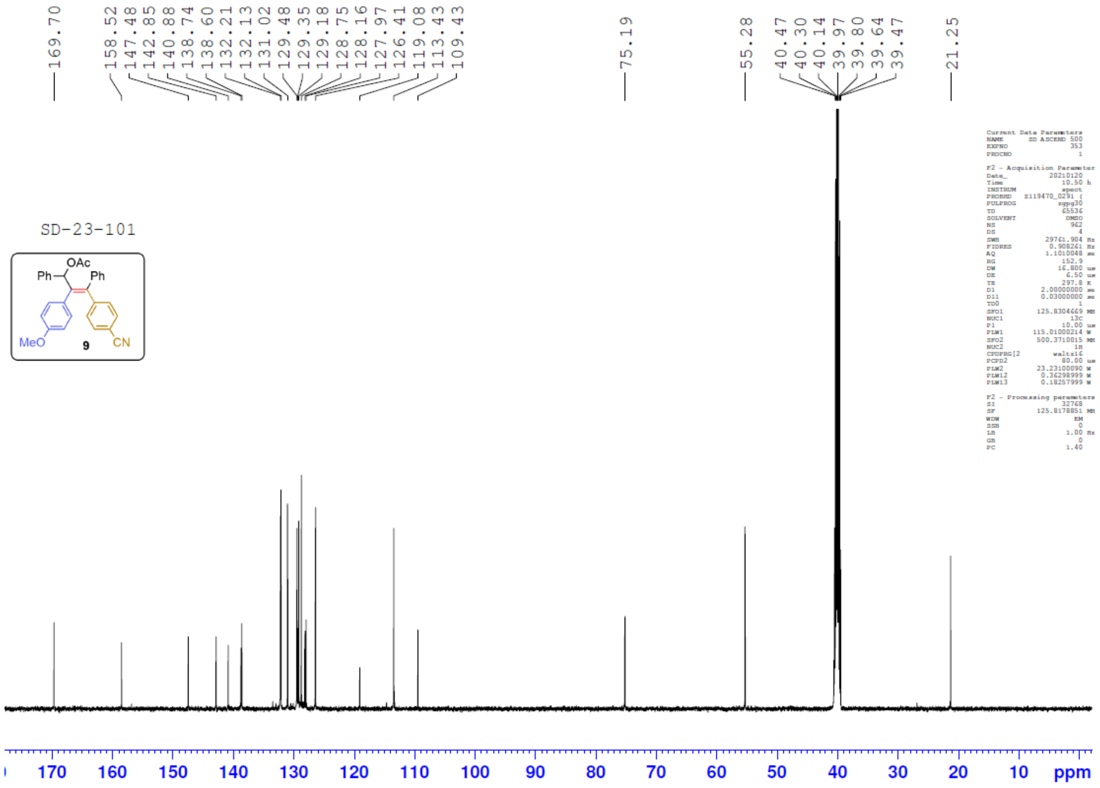

Supplementary Figure 57. ^{13}C NMR of compound **9**.

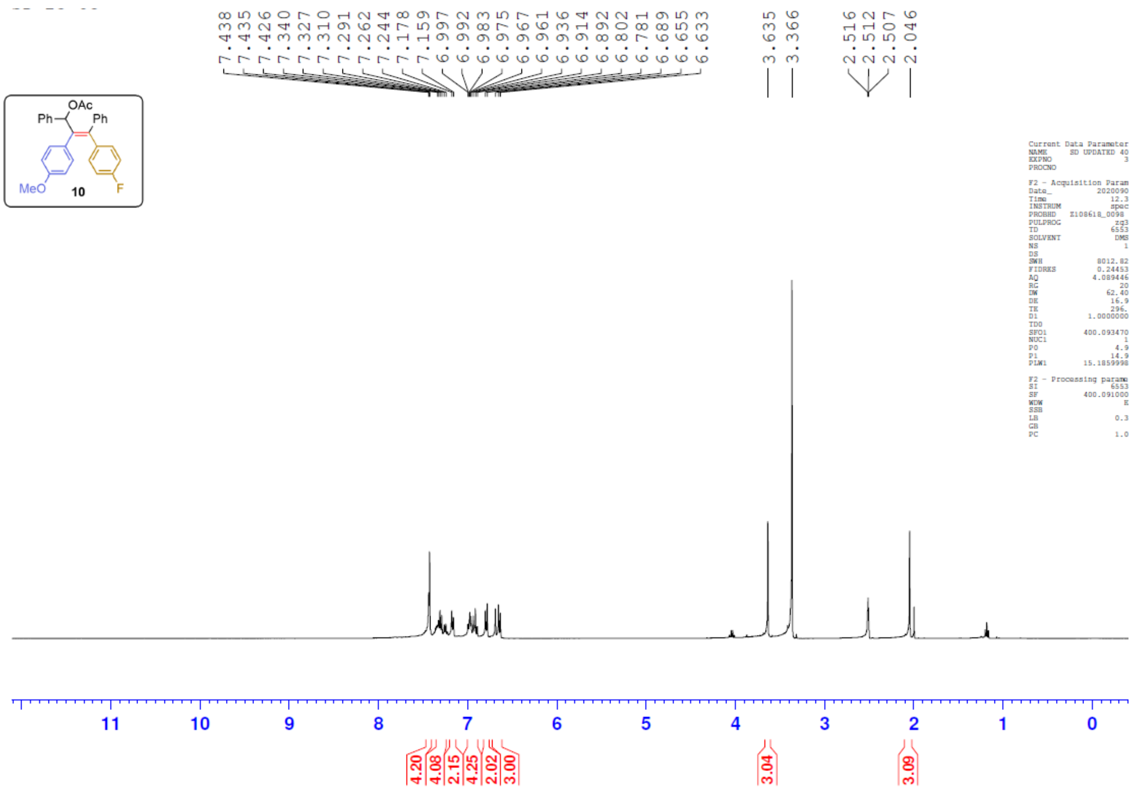

Supplementary Figure 58. ^{1}H NMR of compound **10**.

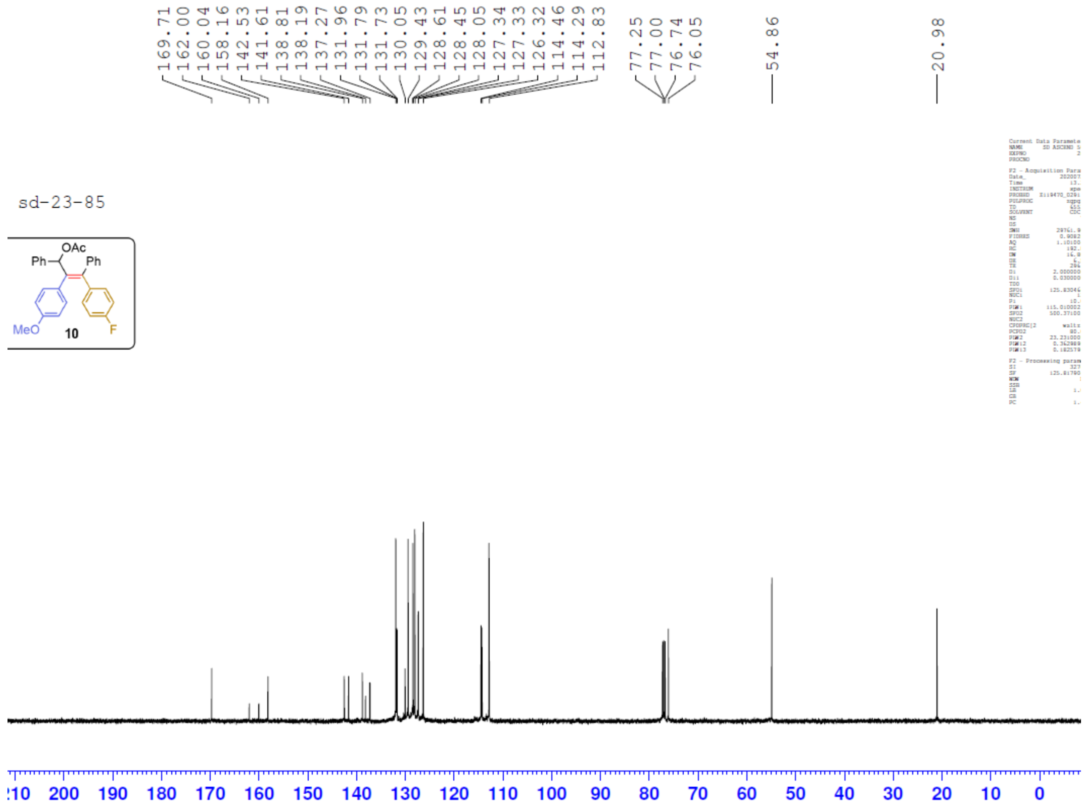

Supplementary Figure 59. ^{13}C NMR of compound **10**.

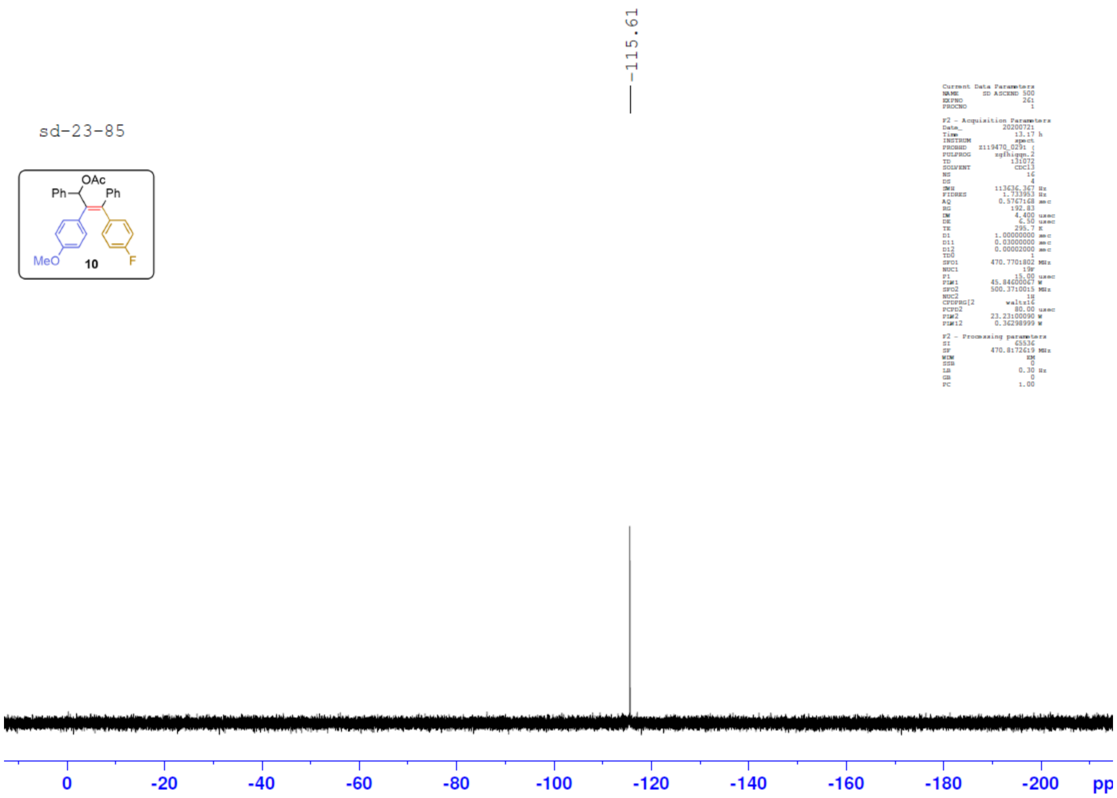

Supplementary Figure 60. ^{13}C NMR of compound **10**.

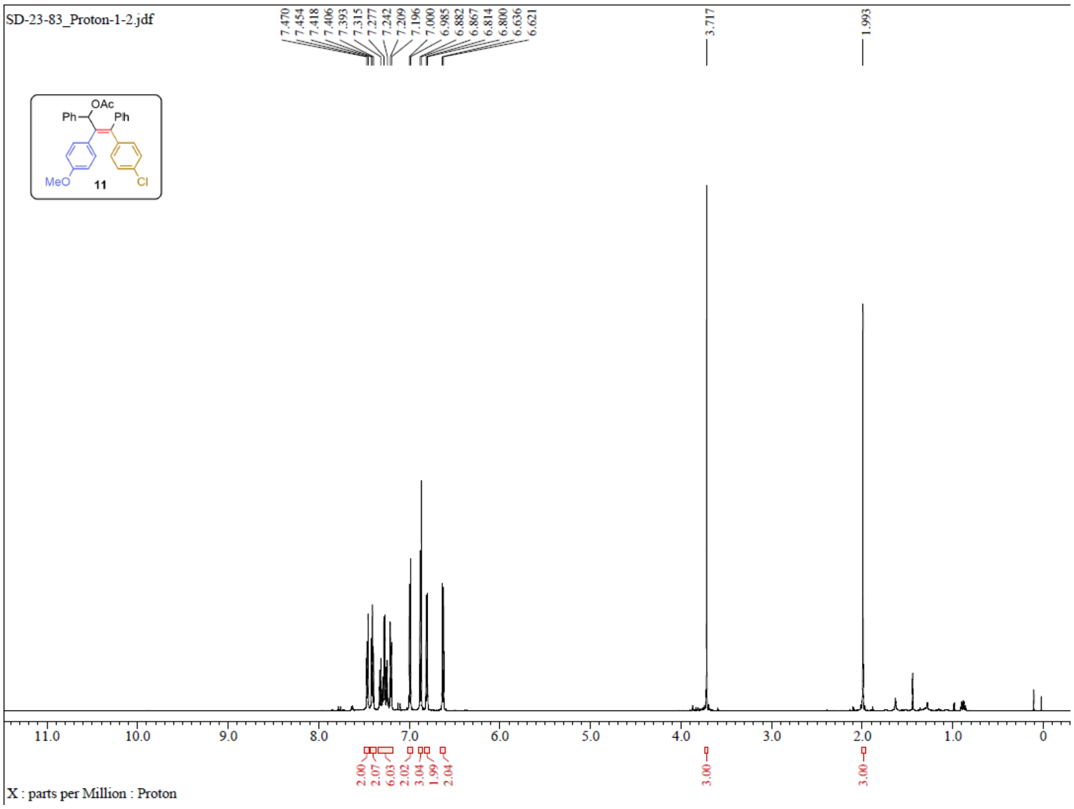

Supplementary Figure 61. ^{1}H NMR of compound **11**.

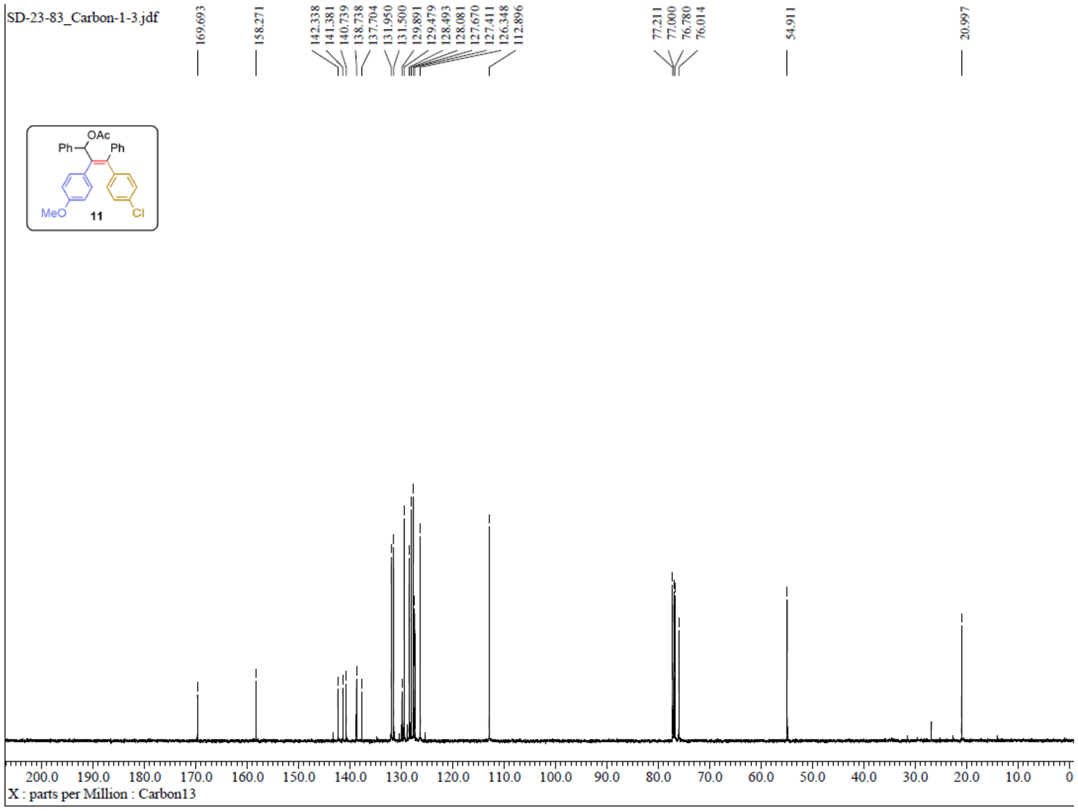

Supplementary Figure 62. ^{13}C NMR of compound **11**.

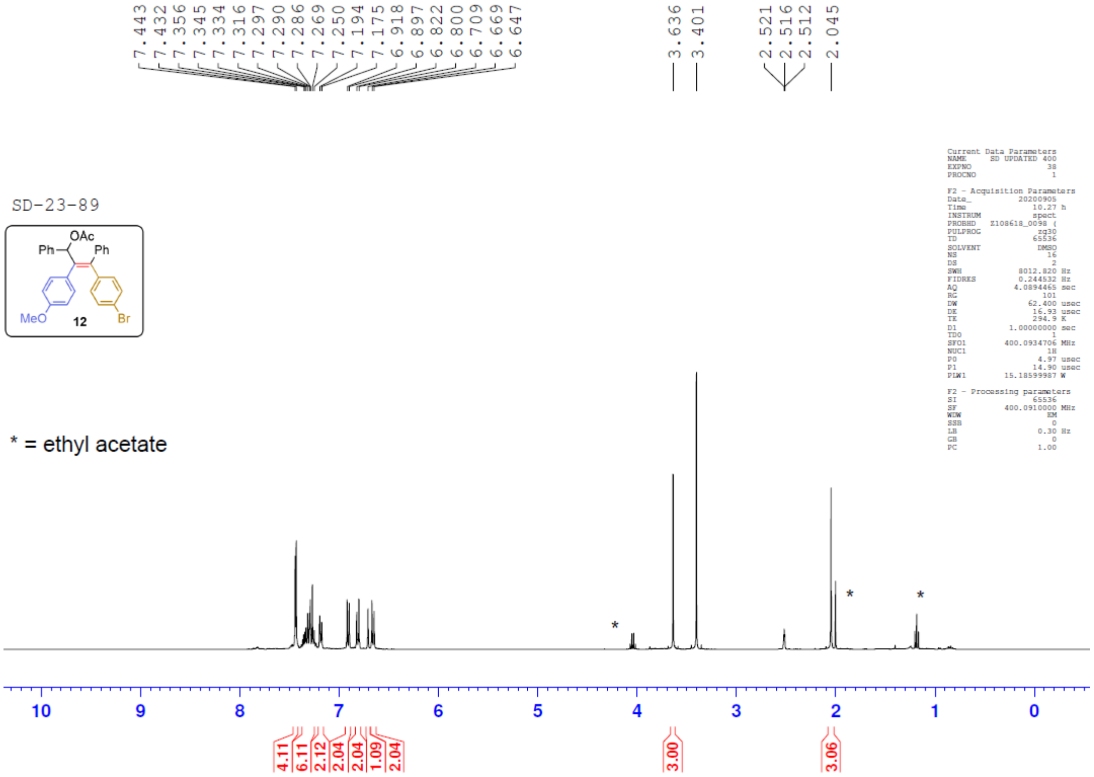

Supplementary Figure 63. ^{1}H NMR of compound **12**.

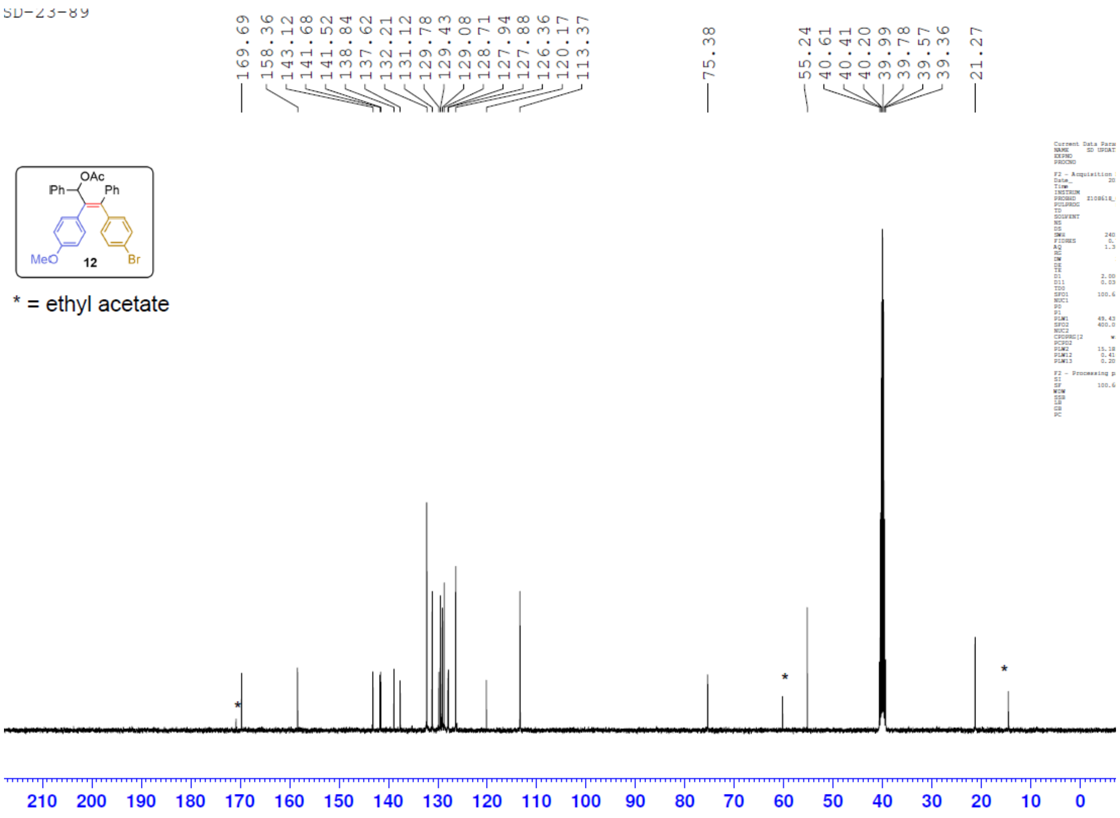

Supplementary Figure 64. ^{13}C NMR of compound **12**.

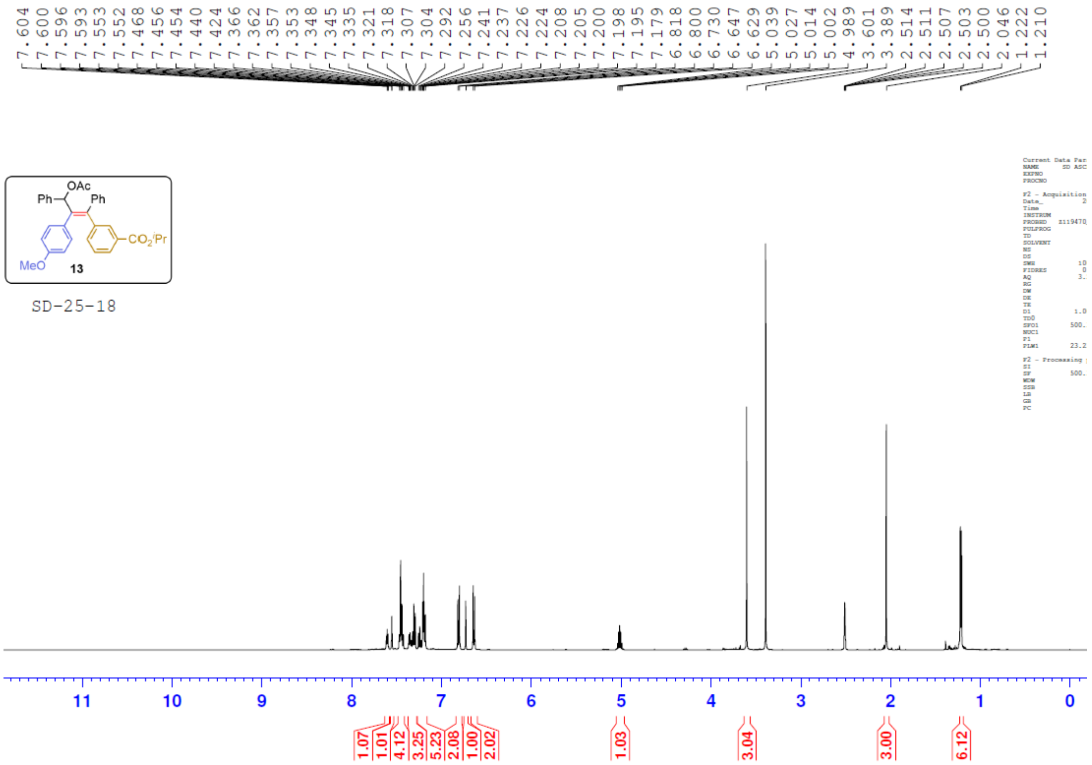

Supplementary Figure 65. ^{1}H NMR of compound **13**.

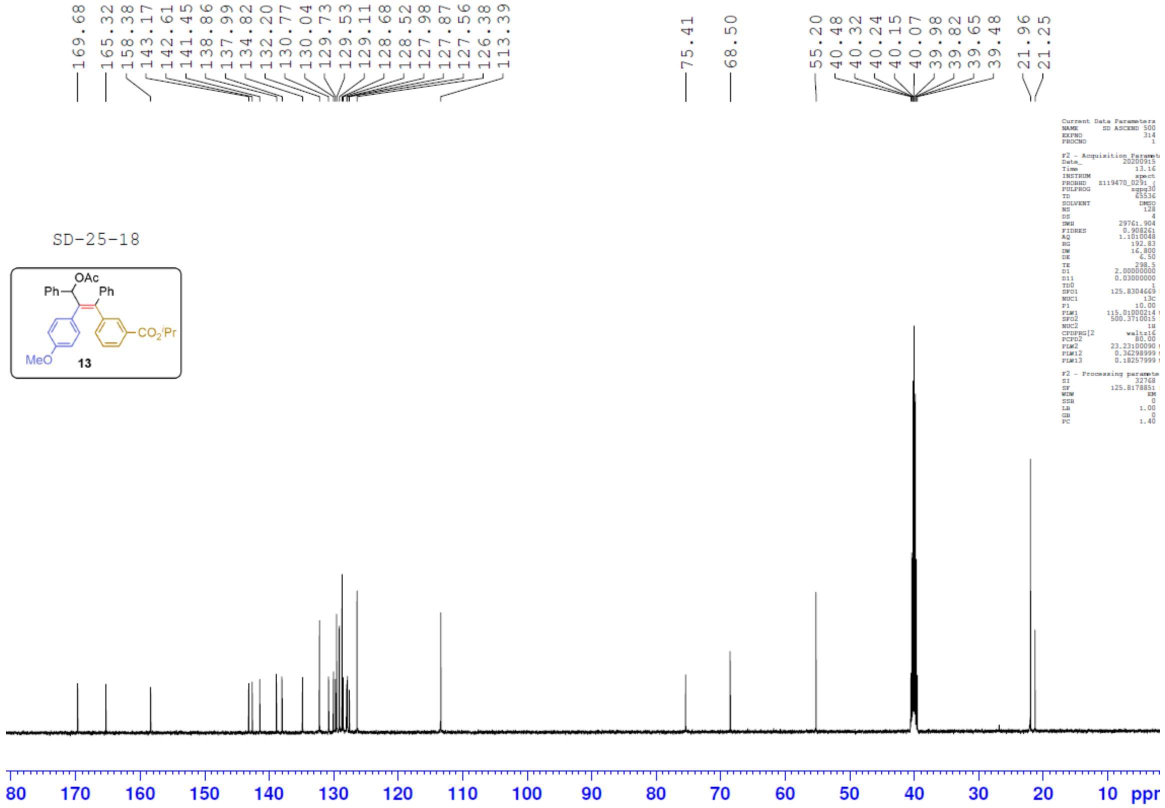

Supplementary Figure 66. ^{13}C NMR of compound **13**

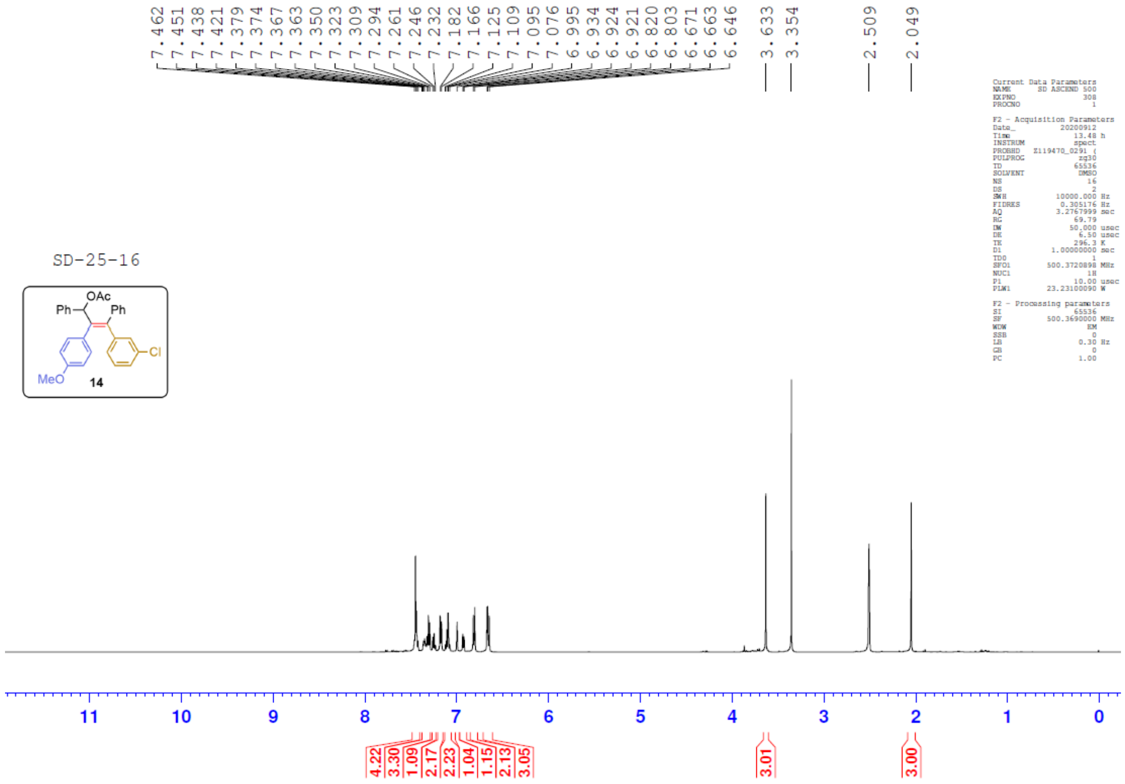

Supplementary Figure 67. ^{1}H NMR of compound **14**

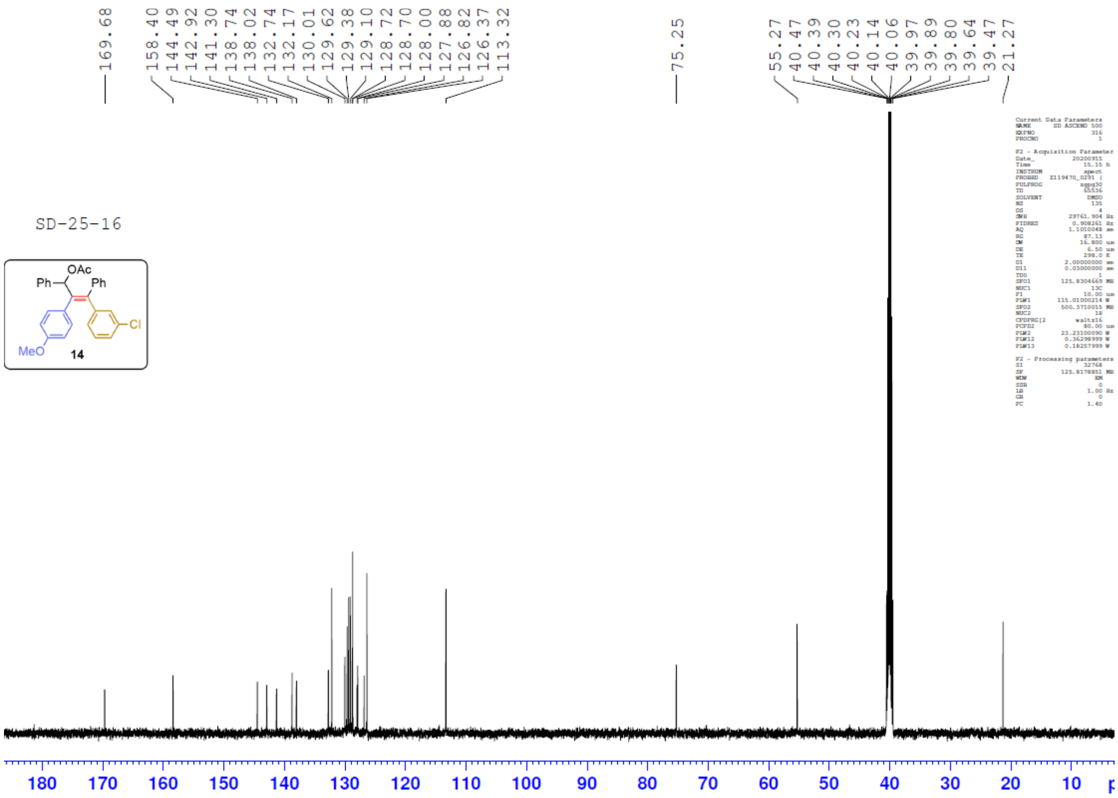

Supplementary Figure 68. ^{13}C NMR of compound **14**

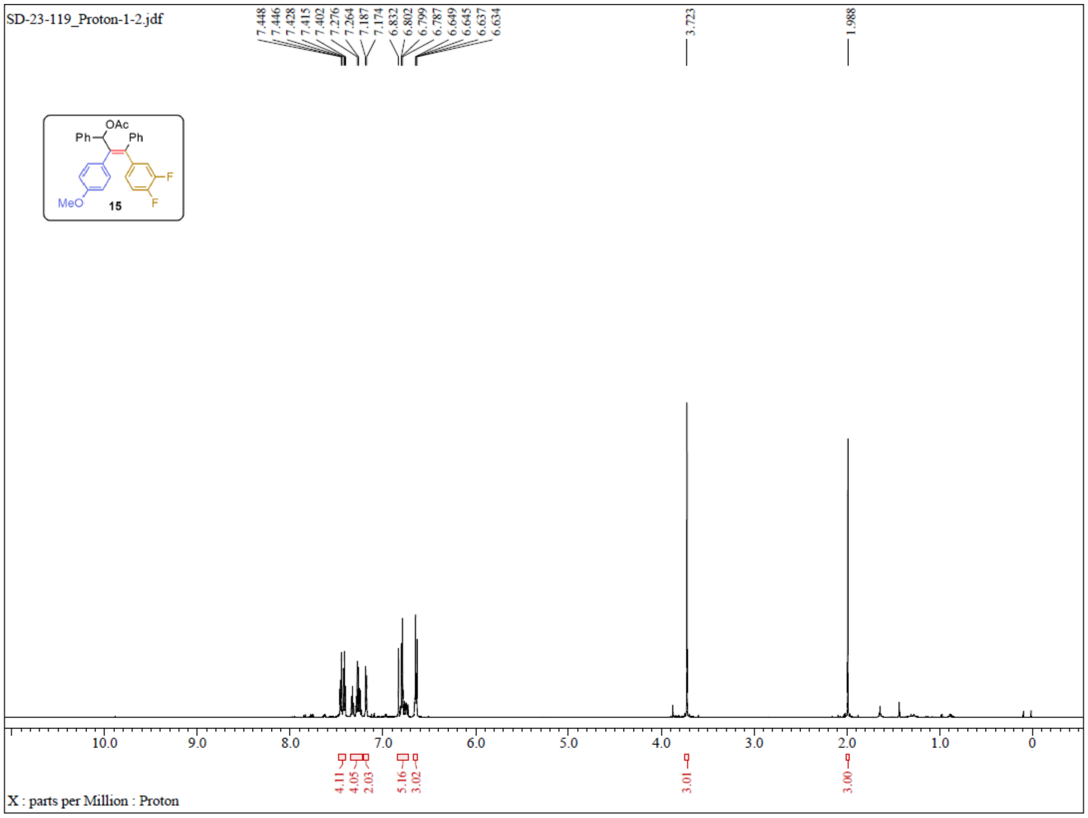

Supplementary Figure 69. ^{1}H NMR of compound **15**

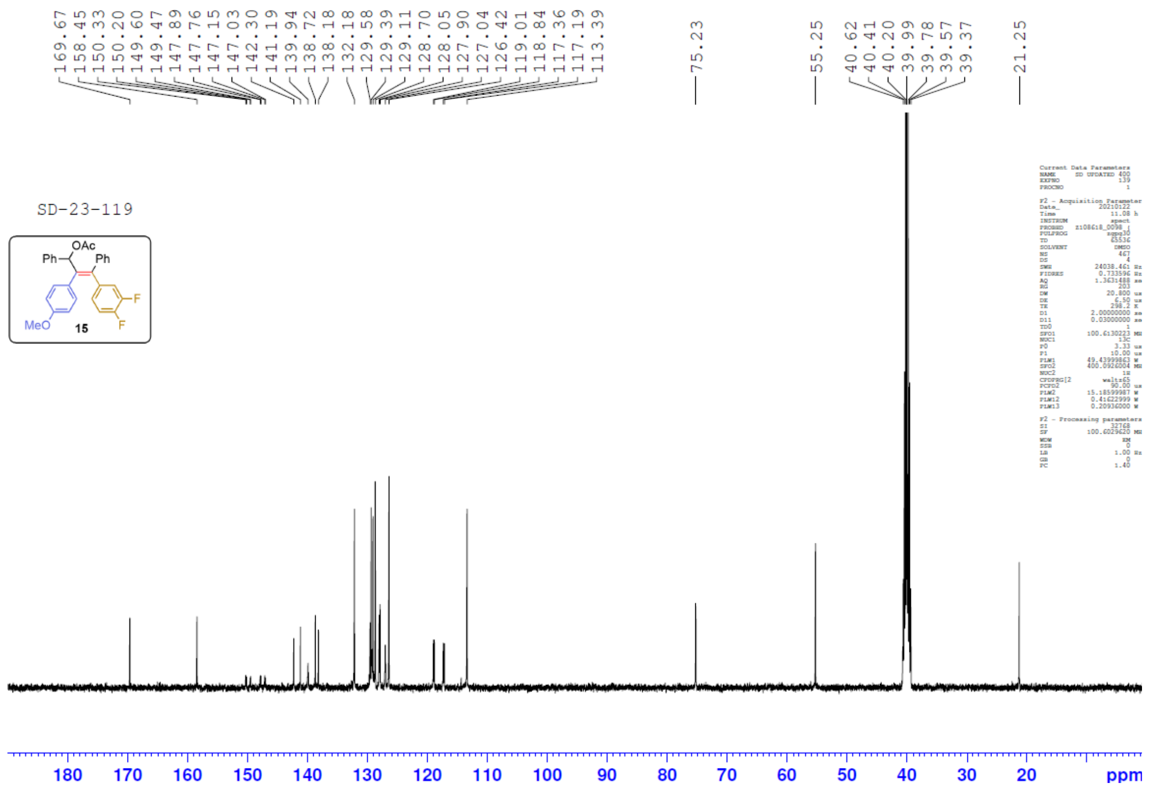

Supplementary Figure 70. ^{13}C NMR of compound **15**

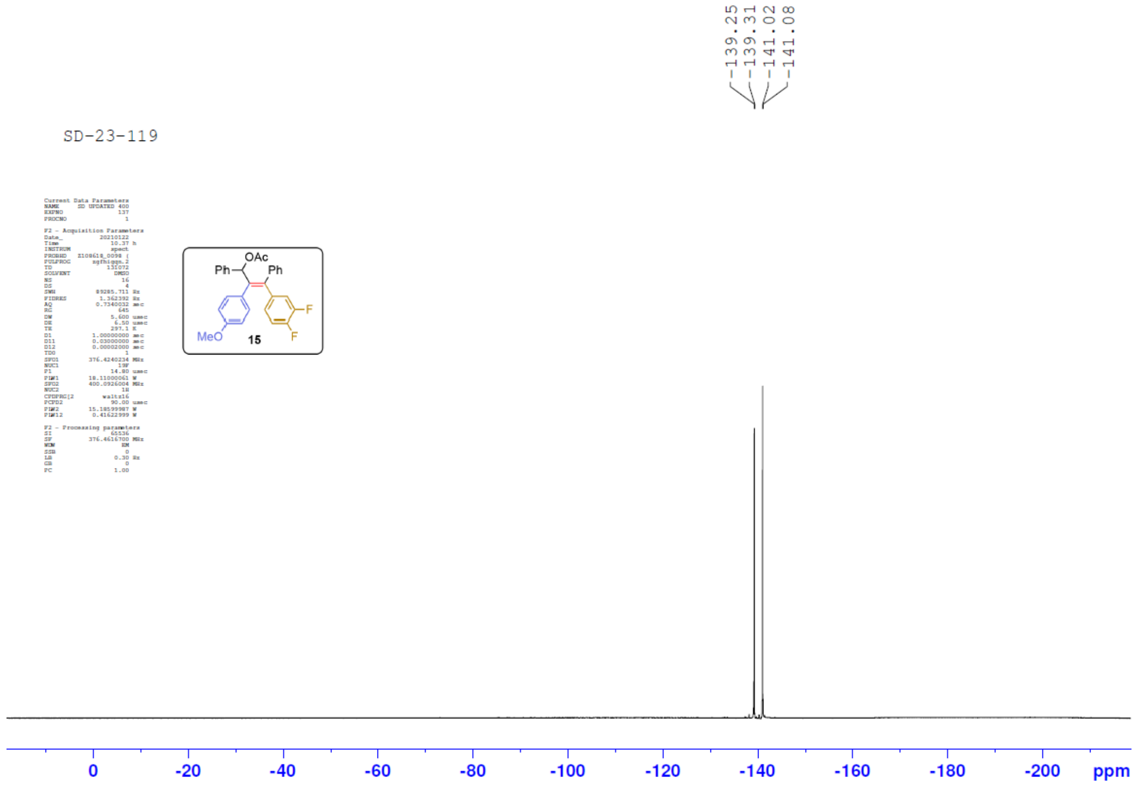

Supplementary Figure 71. ^{19}F NMR of compound **15**

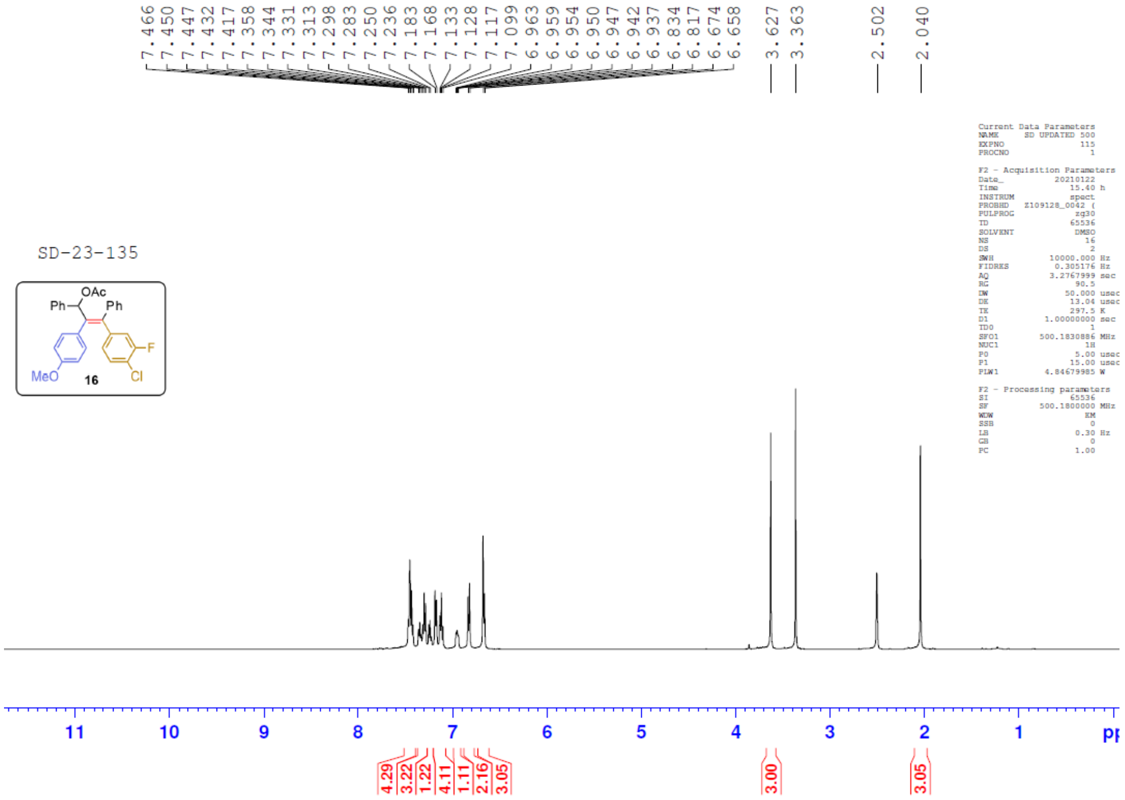

Supplementary Figure 72. ^{1}H NMR of compound **16**

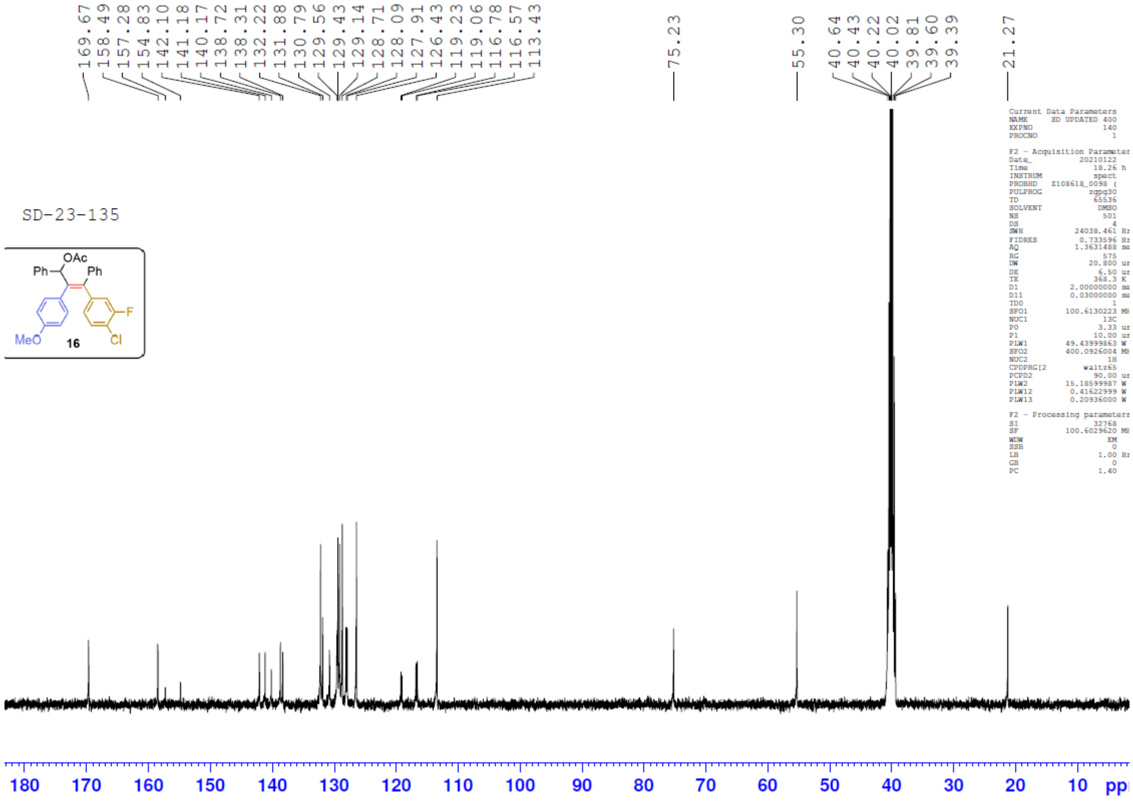

Supplementary Figure 73. ^{13}C NMR of compound **16**

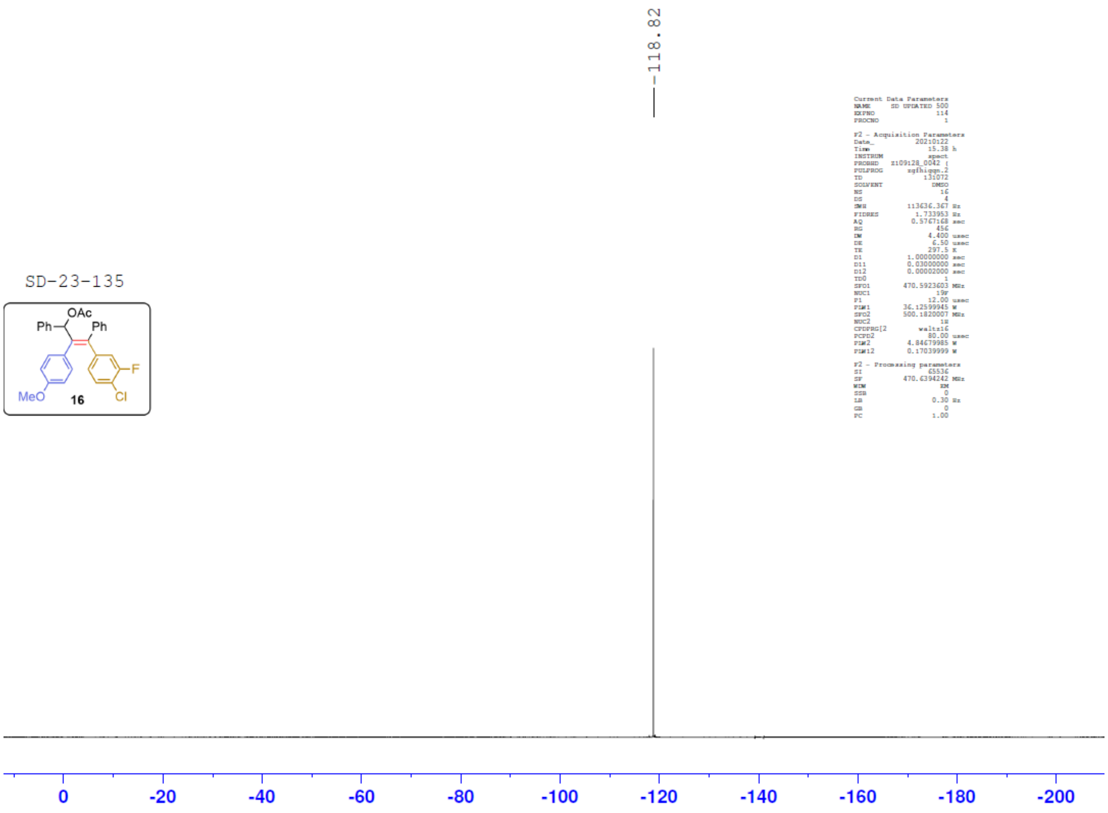

Supplementary Figure 74. ^{19}F NMR of compound **16**

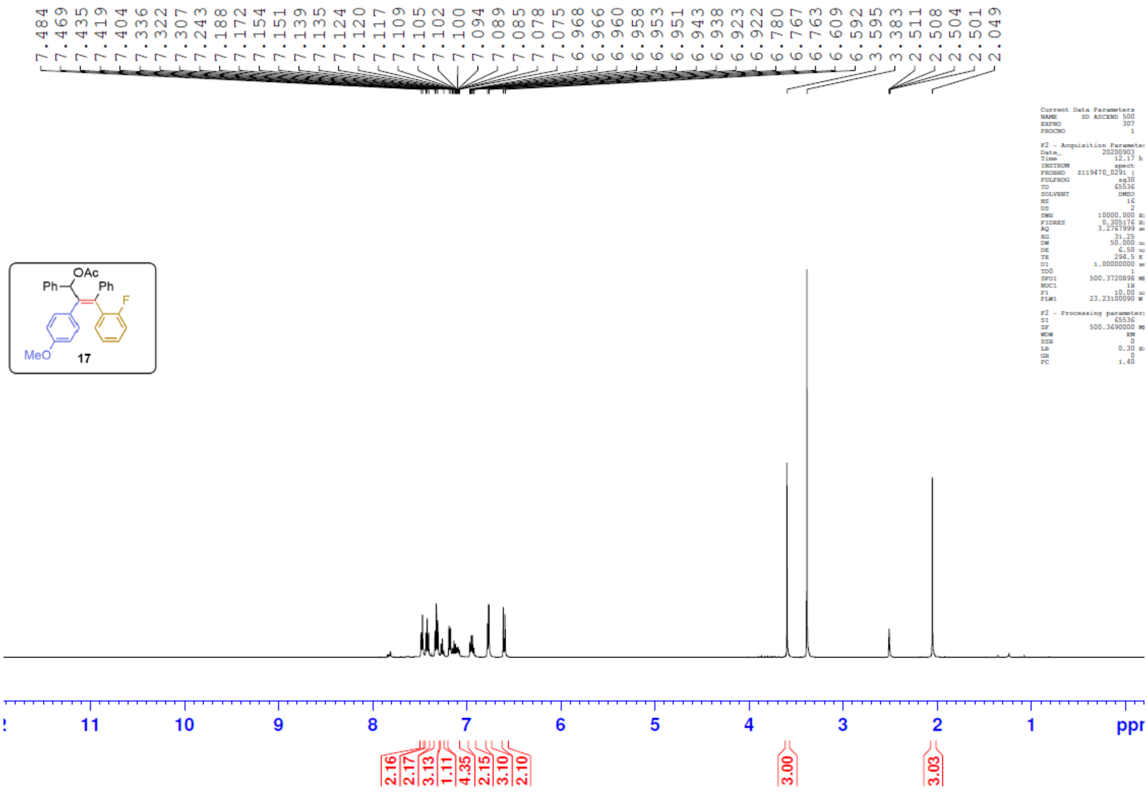

Supplementary Figure 75. ^{1}H NMR of compound **17**

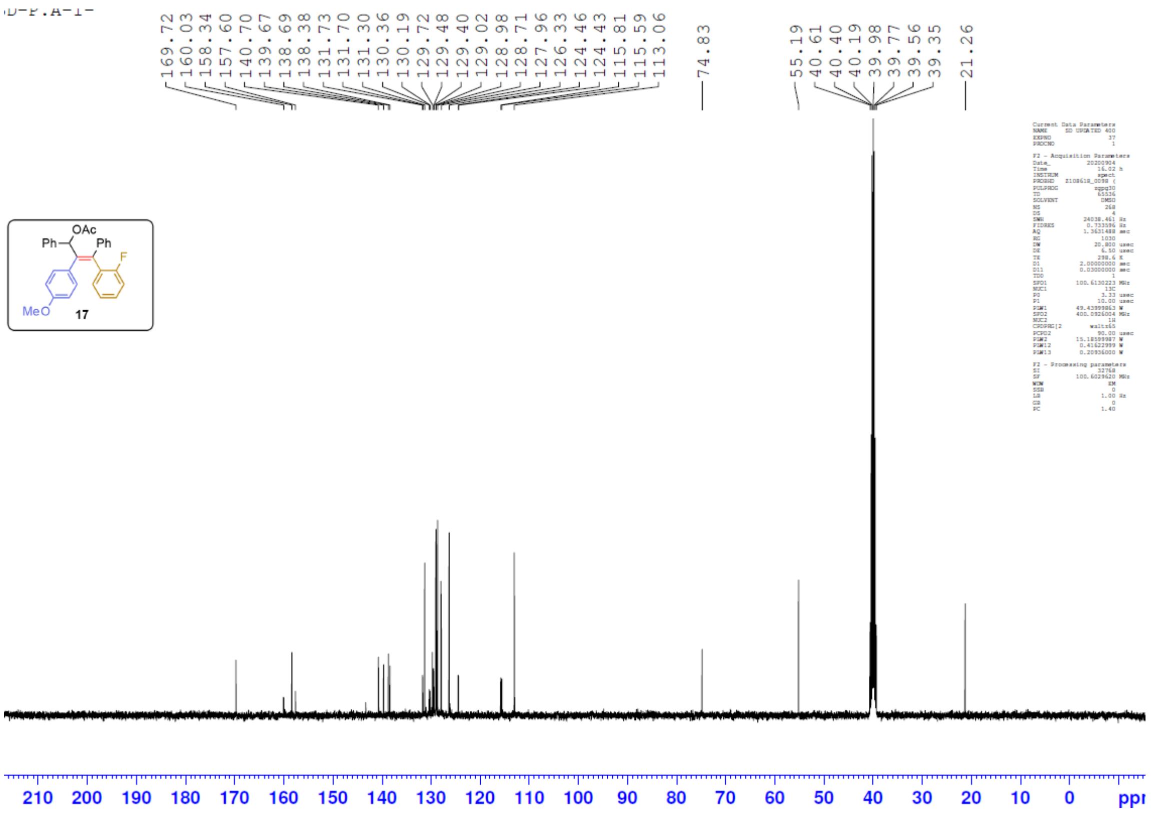

Supplementary Figure 76. ^{13}C NMR of compound **17**

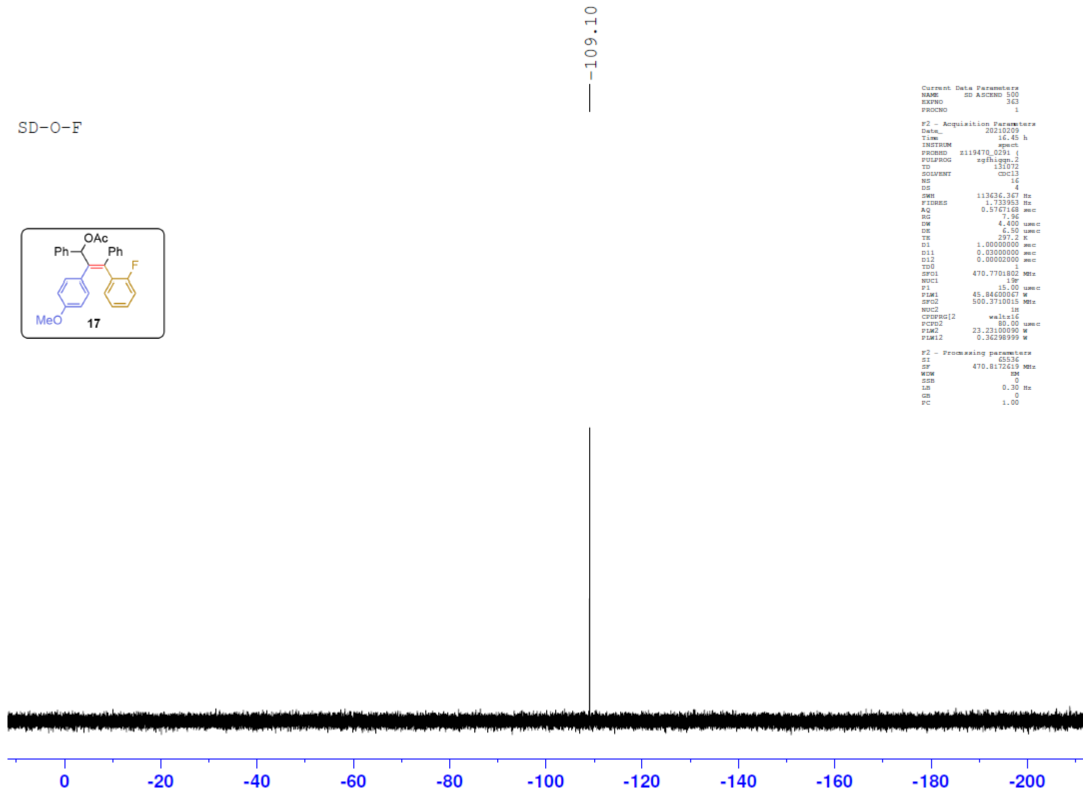

Supplementary Figure 77. ^{19}F NMR of compound **17**

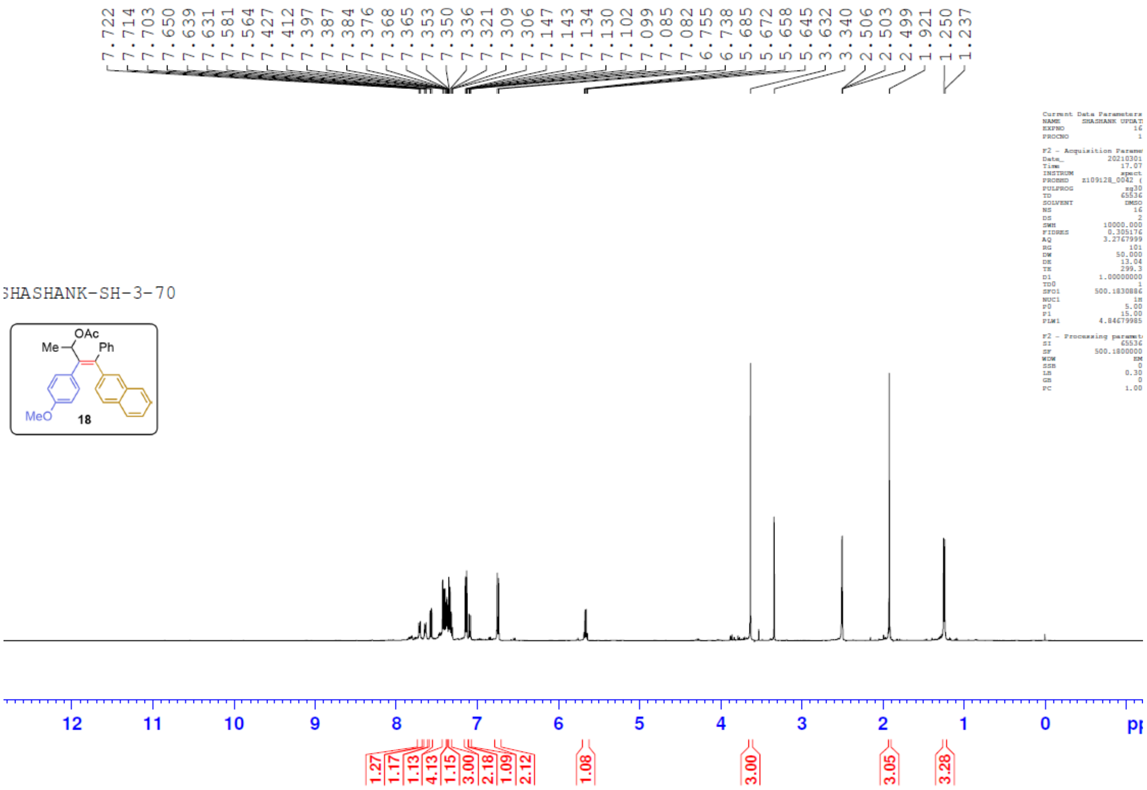

Supplementary Figure 78. ^{1}H NMR of compound **18**

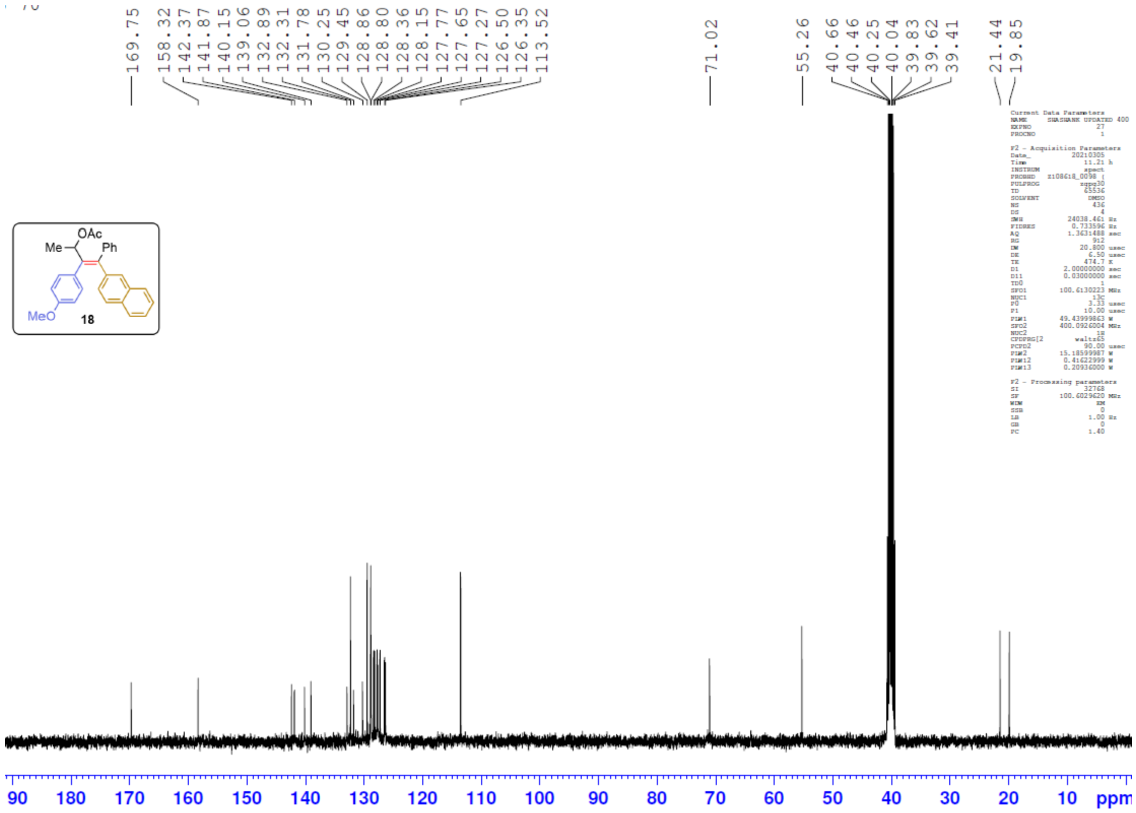

Supplementary Figure 79. ^{13}C NMR of compound **18**

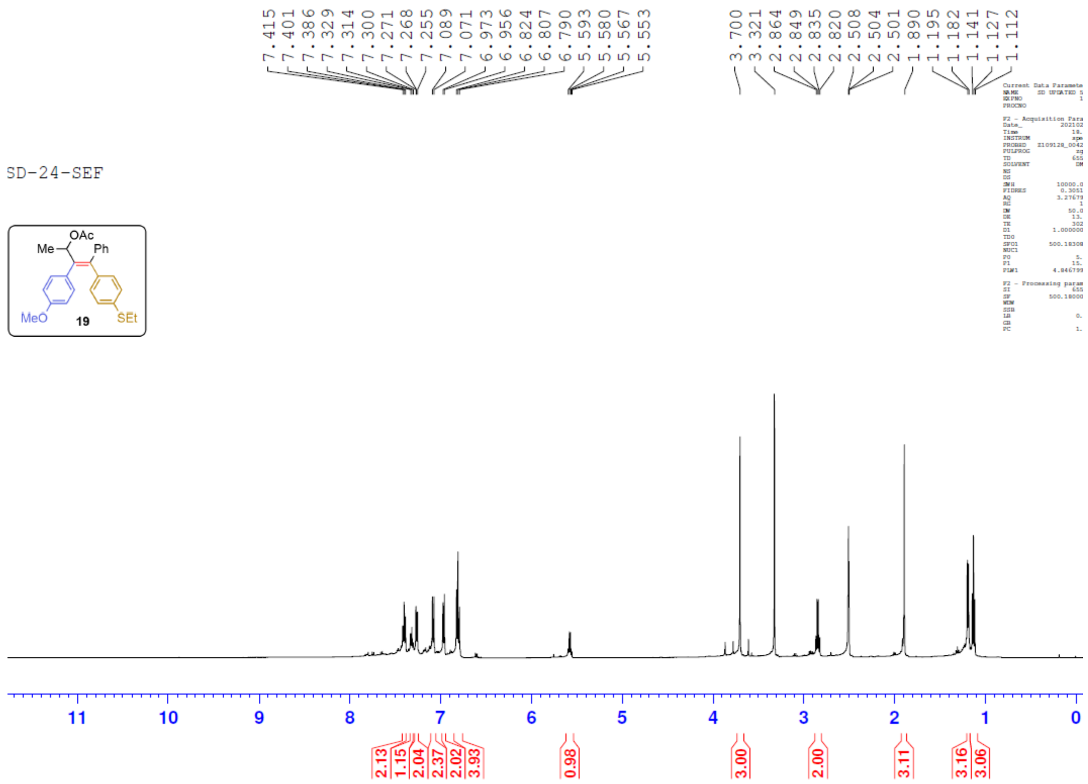

Supplementary Figure 80. ^{1}H NMR of compound **19**

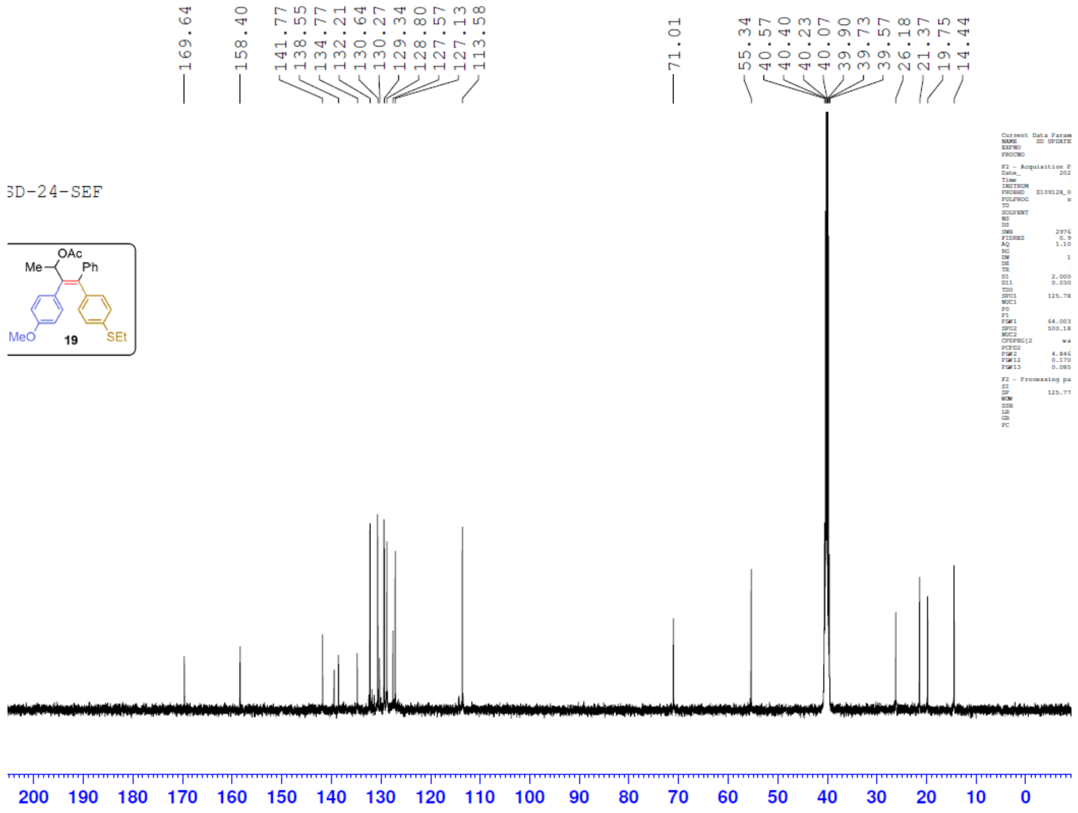

Supplementary Figure 81. ^{13}C NMR of compound **19**

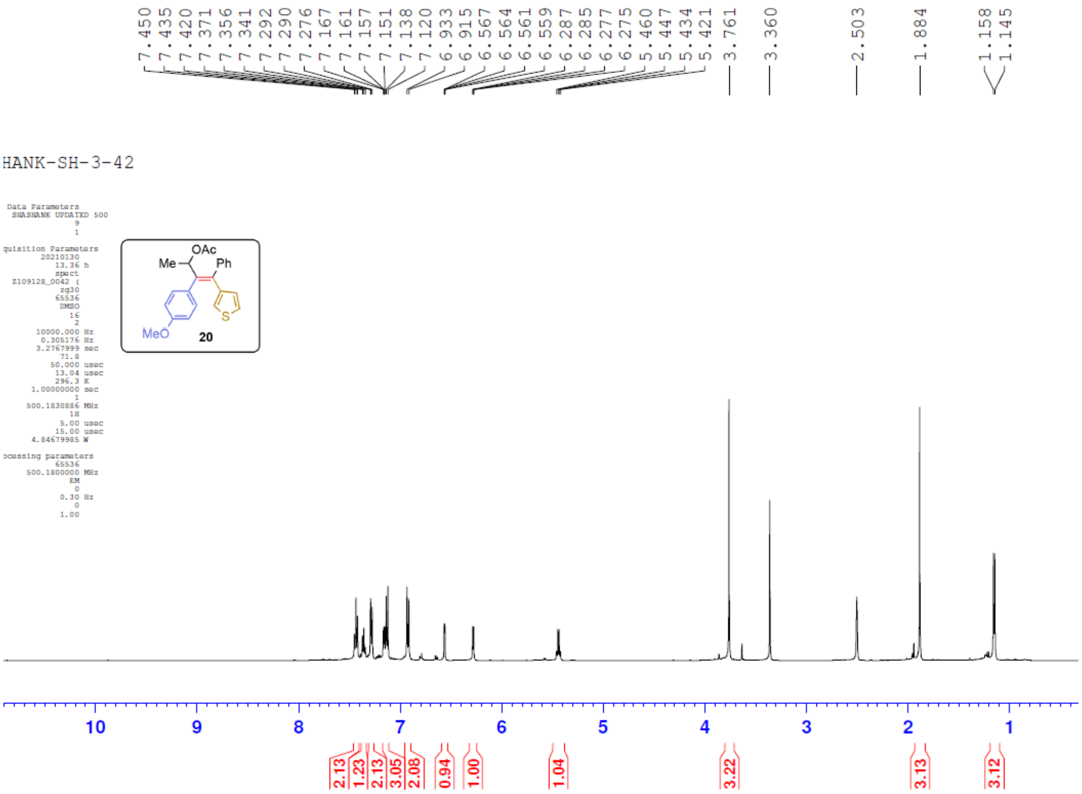

Supplementary Figure 82. ^{1}H NMR of compound **20**

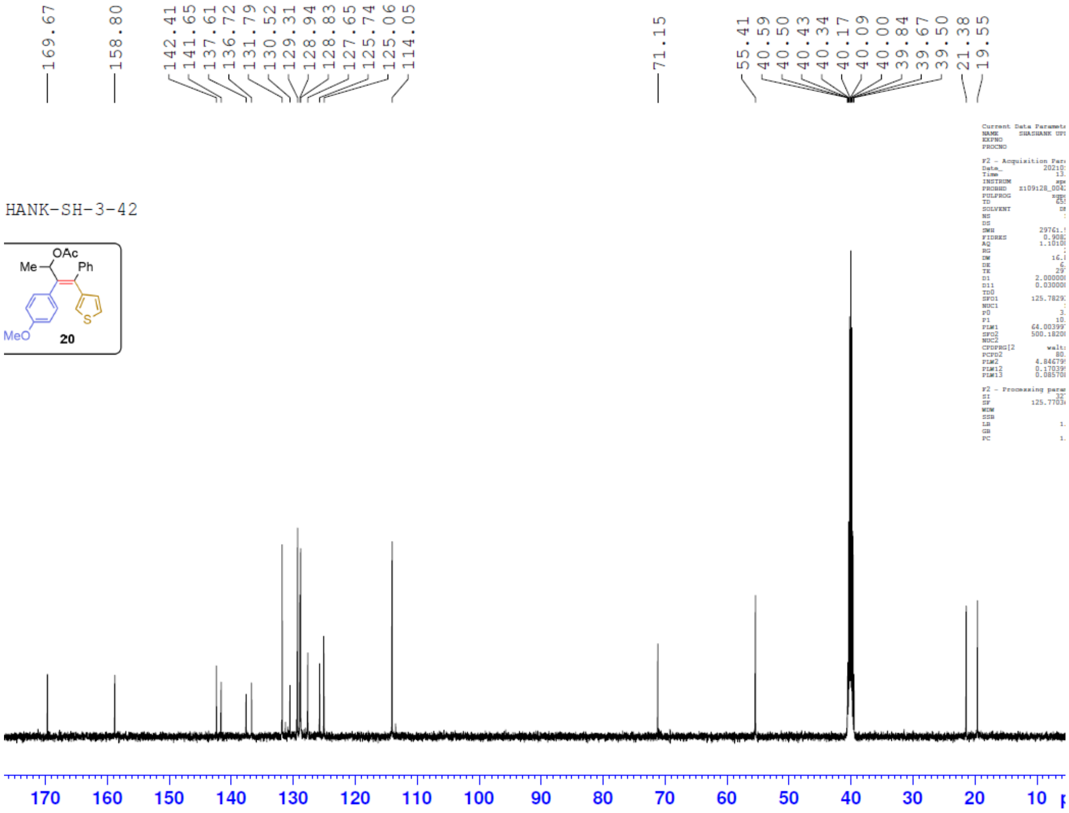

Supplementary Figure 83. ^{13}C NMR of compound **20**

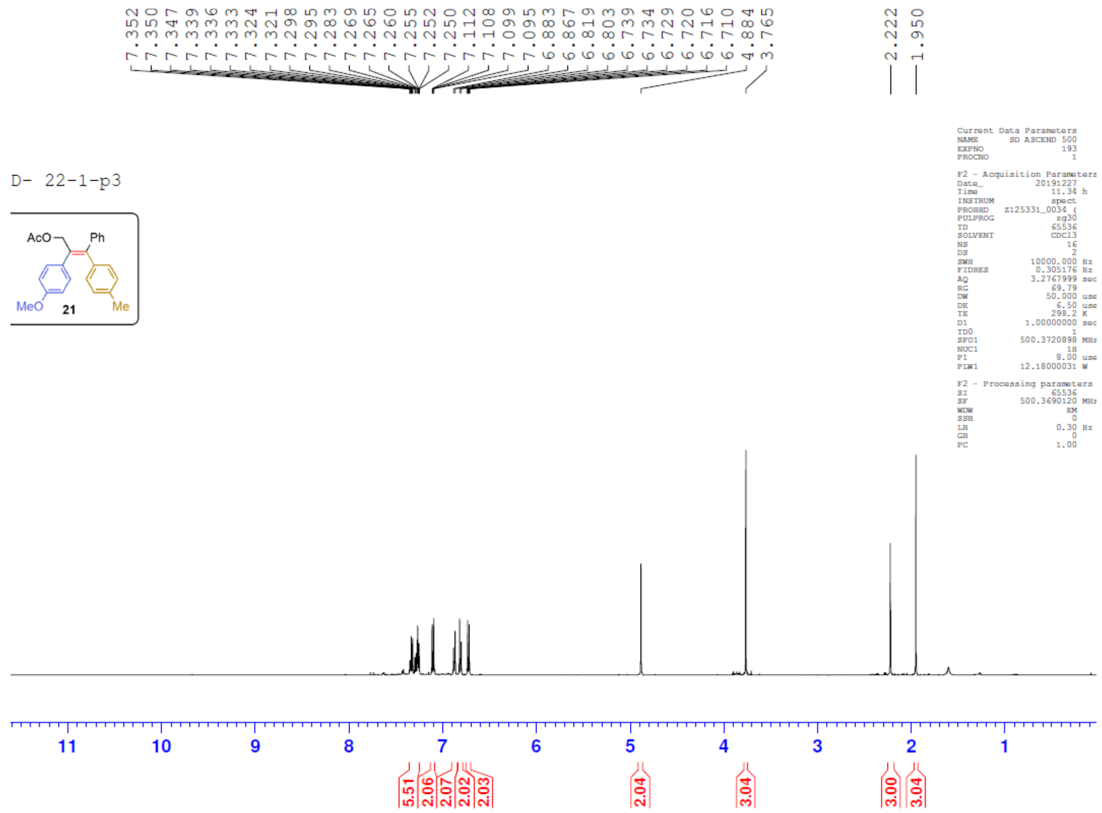

Supplementary Figure 84. ^{1}H NMR of compound **21**

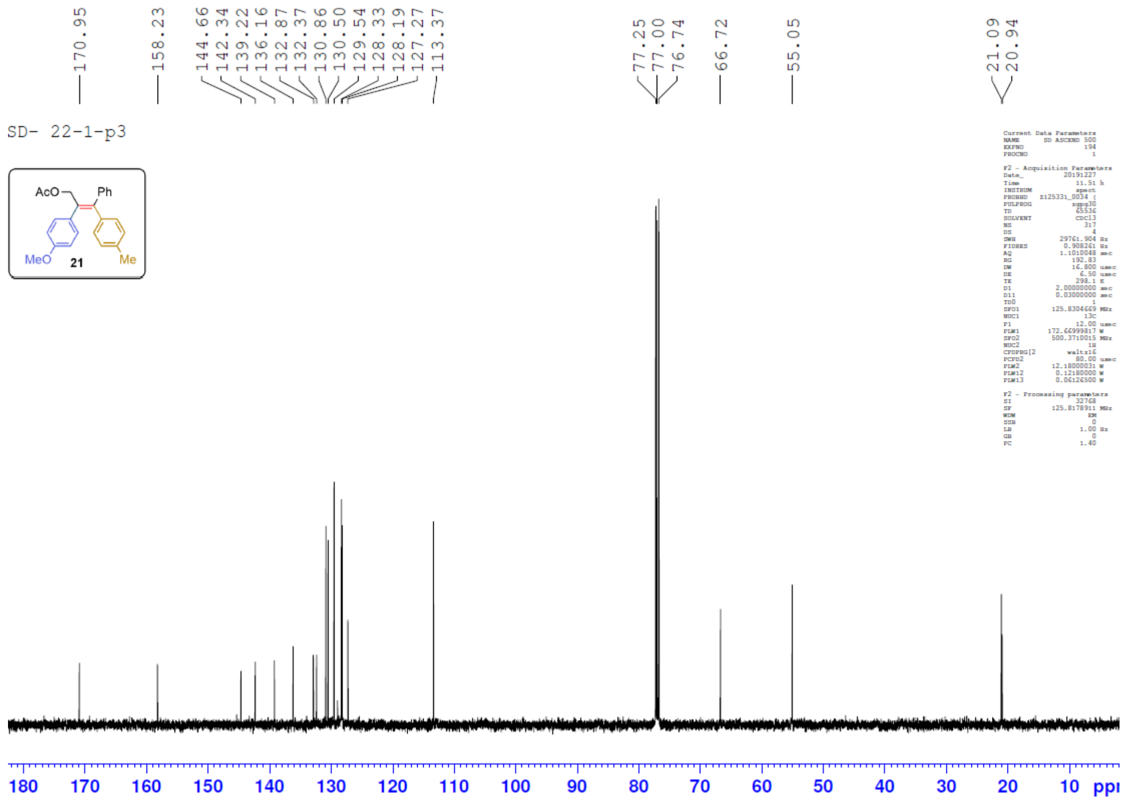

Supplementary Figure 85. ^{1}H NMR of compound **21**

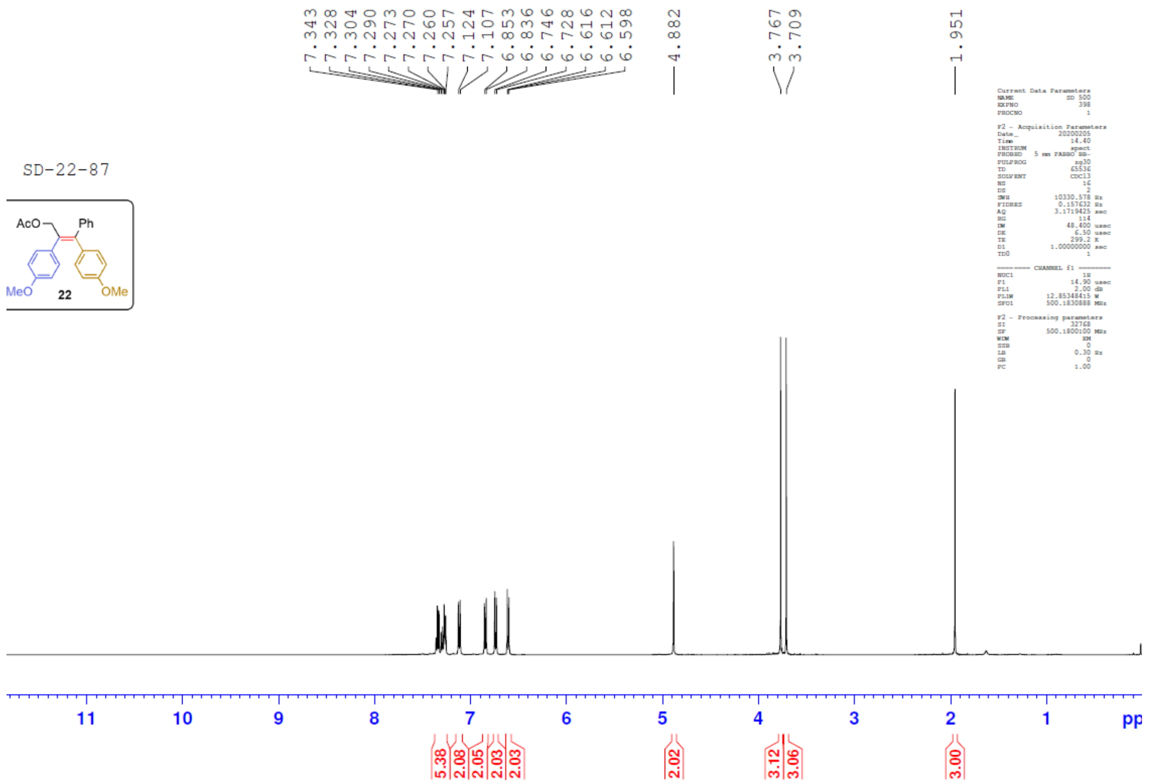

Supplementary Figure 86. ^{1}H NMR of compound **22**

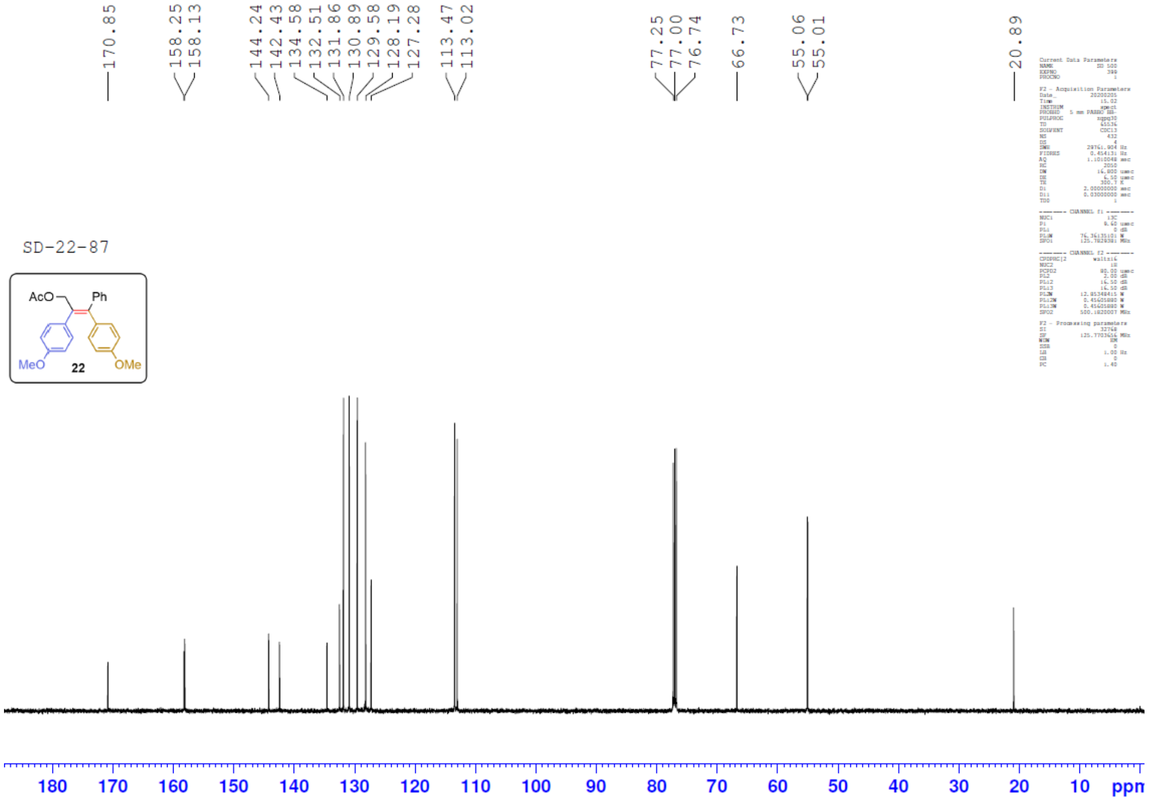

Supplementary Figure 87. ^{13}C NMR of compound **22**

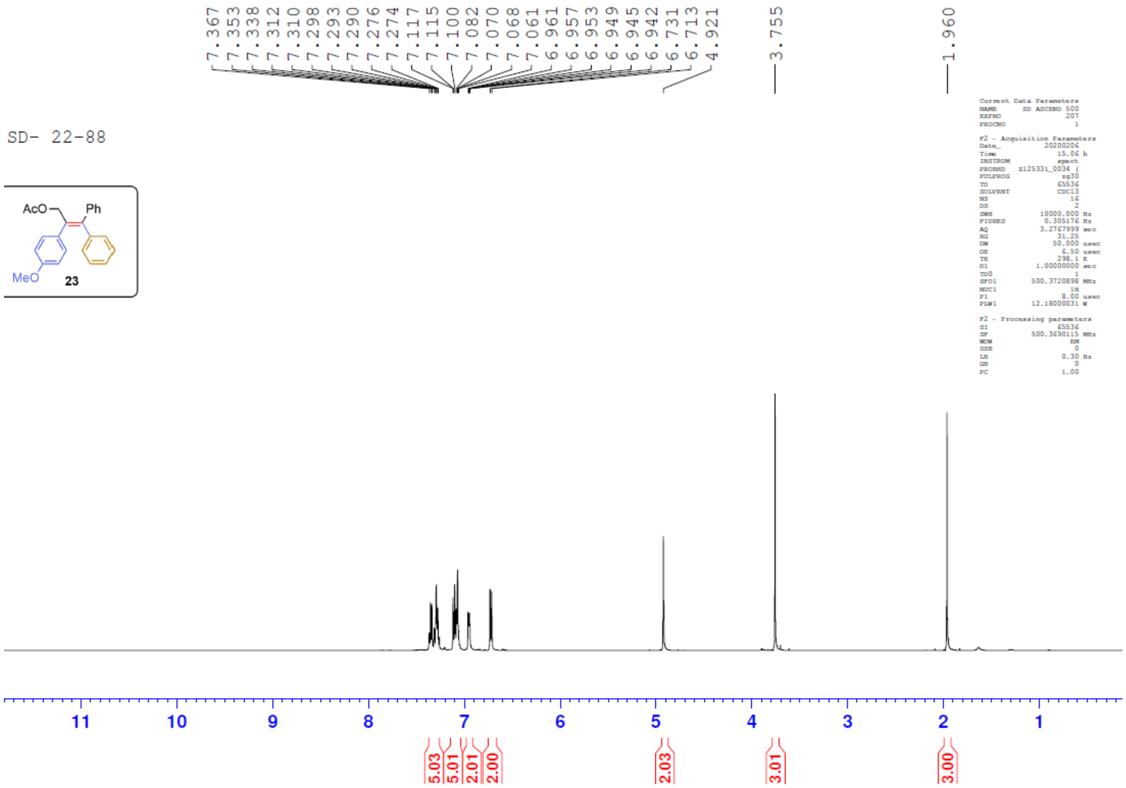

Supplementary Figure 88. ^{1}H NMR of compound **23**

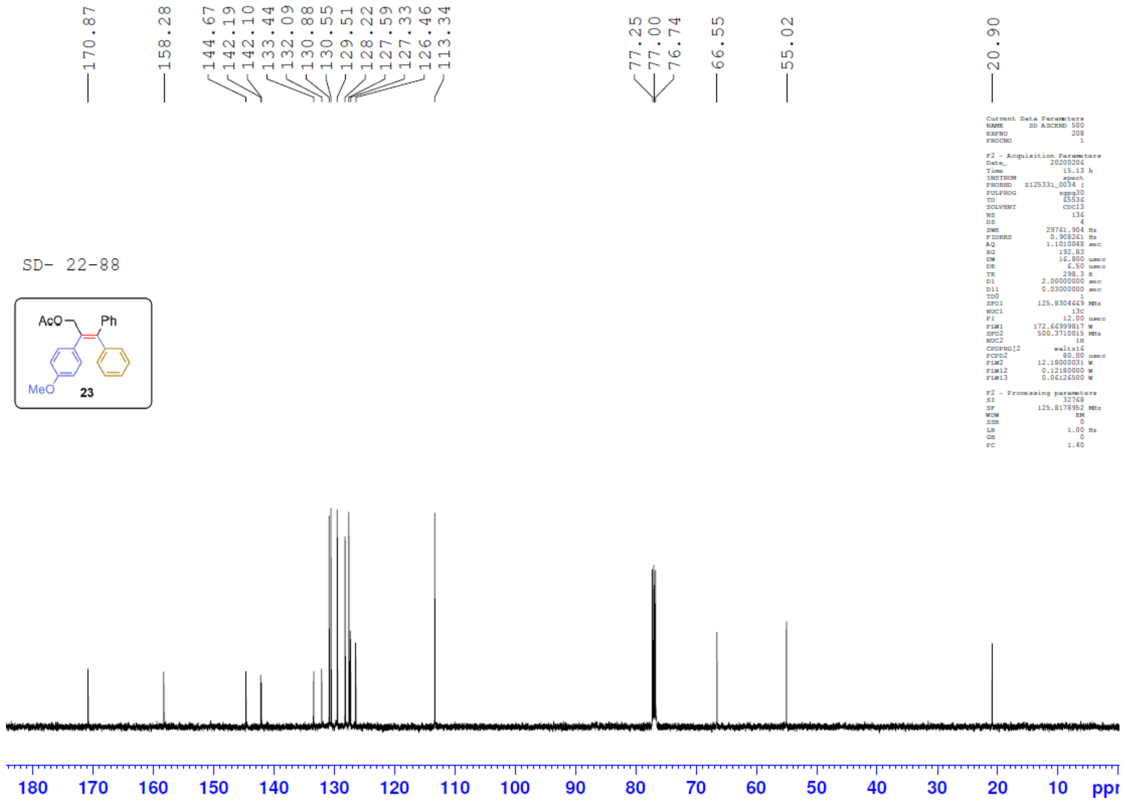

Supplementary Figure 89. ^{13}C NMR of compound **23**

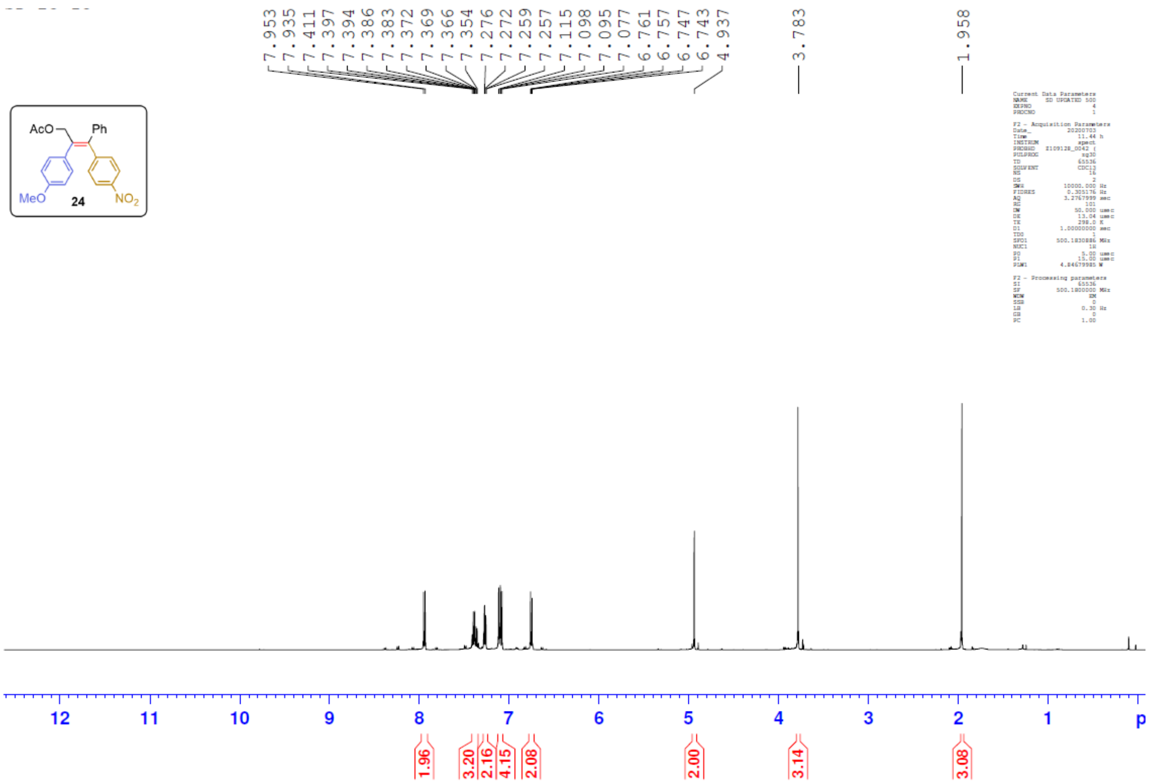

Supplementary Figure 90. ^{1}H NMR of compound **24**

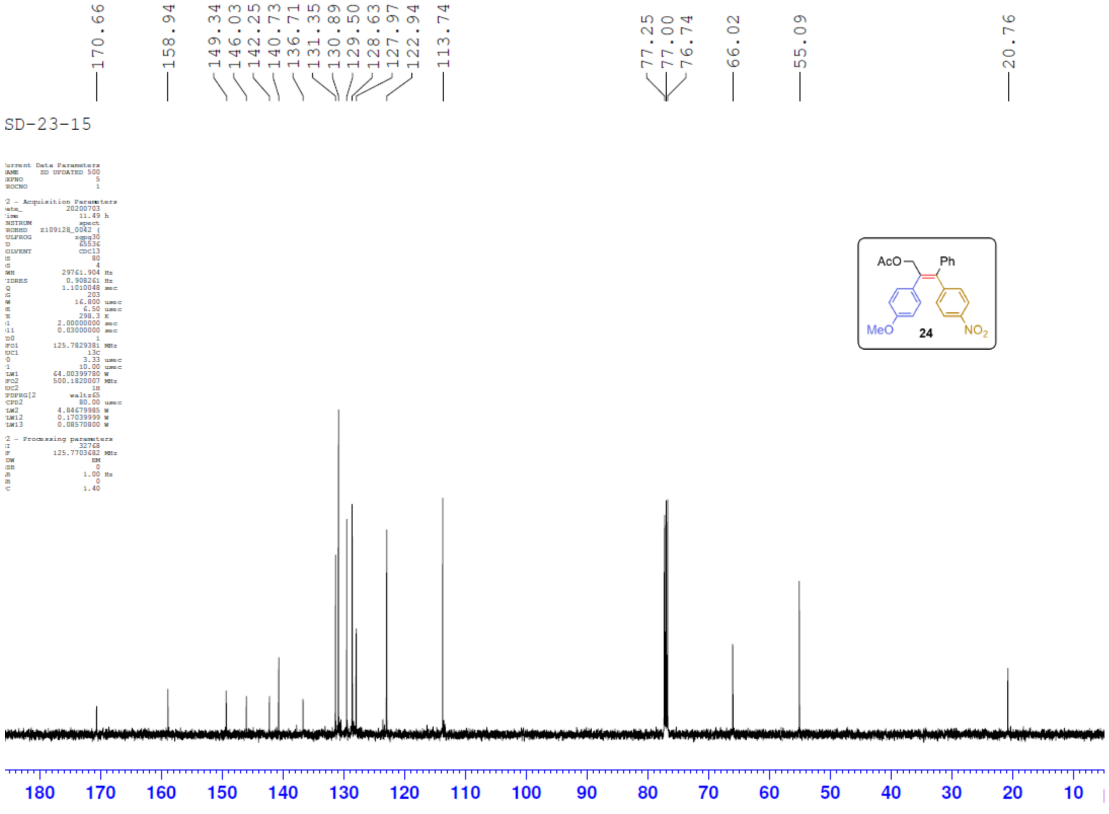

Supplementary Figure 91. ^{13}C NMR of compound **24**

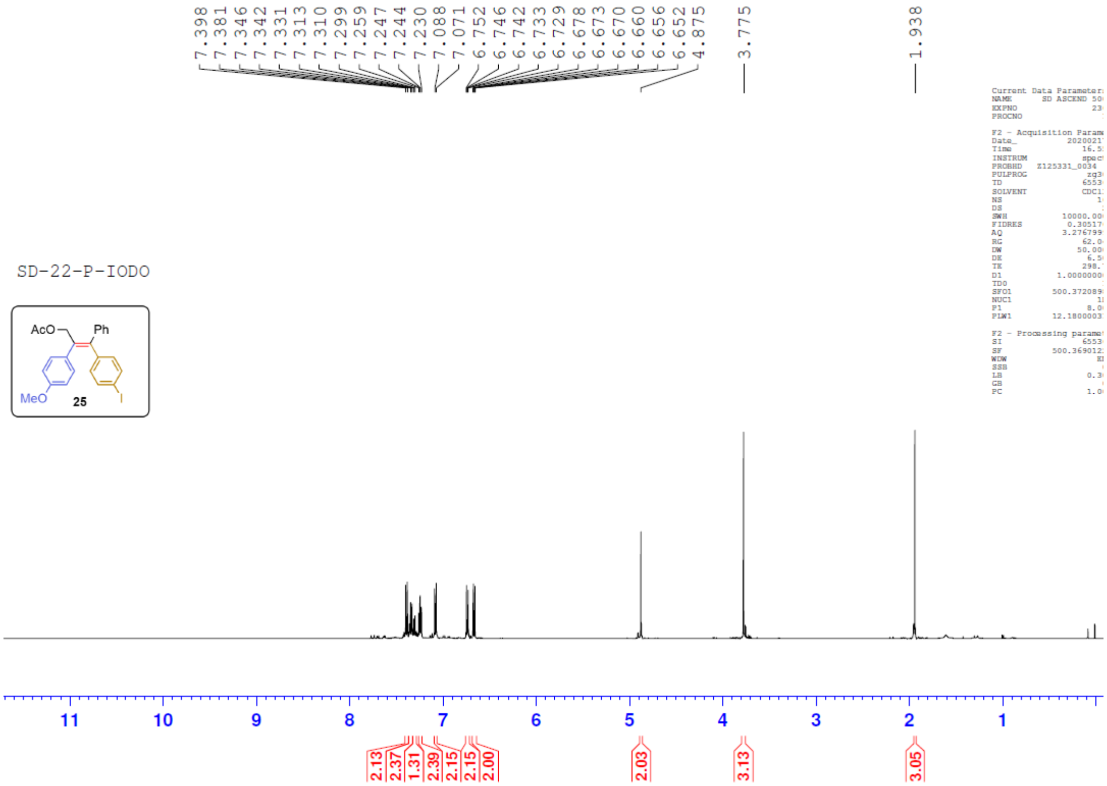

Supplementary Figure 92. ^{1}H NMR of compound **25**

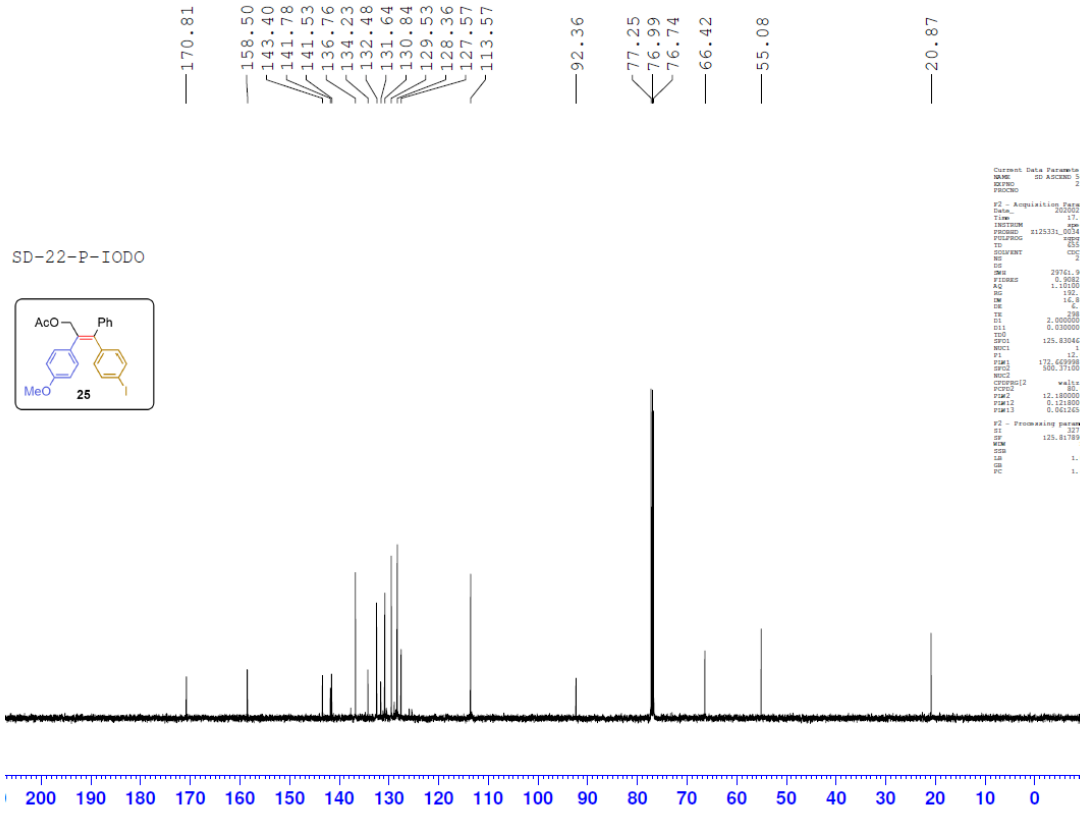

Supplementary Figure 93. ^{13}C NMR of compound **25**

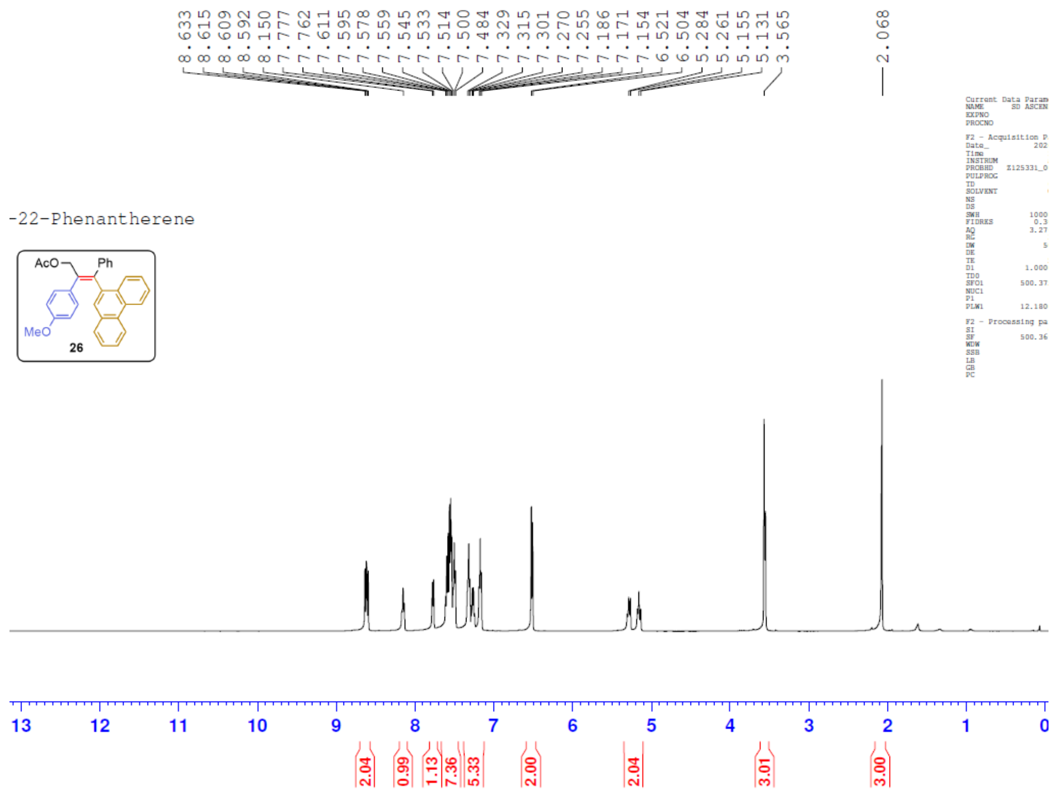

Supplementary Figure 94. ^{1}H NMR of compound **26**

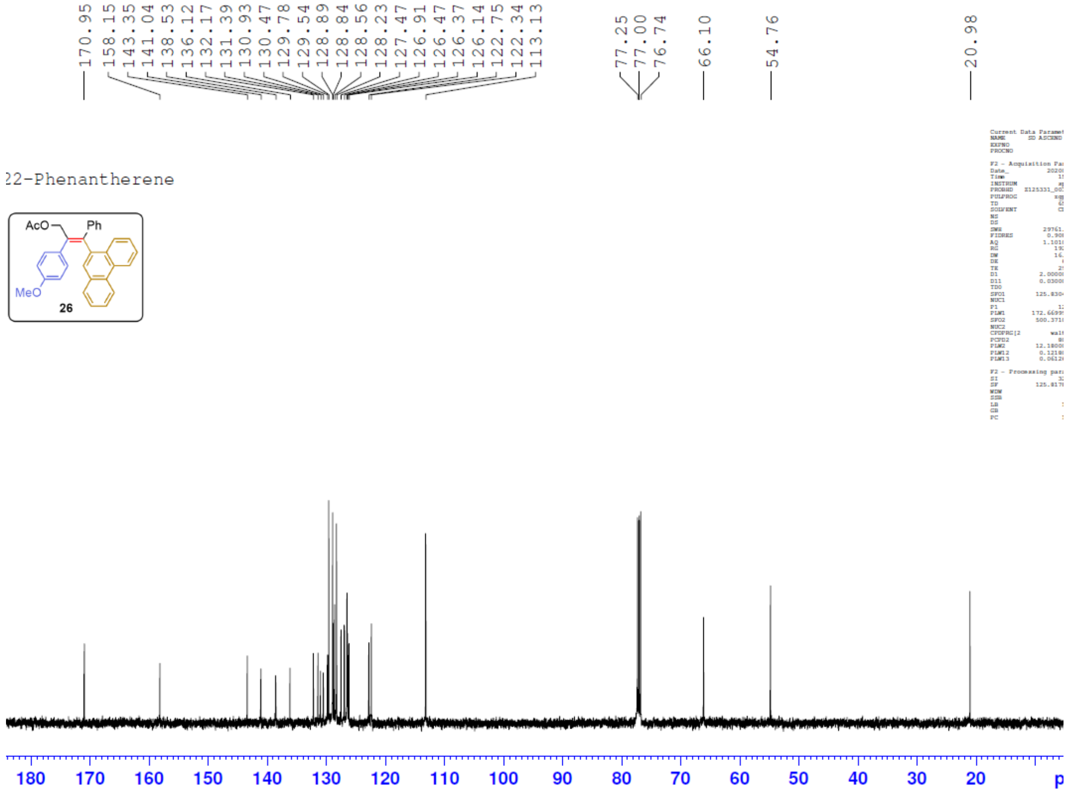

Supplementary Figure 95. ^{13}C NMR of compound **26**

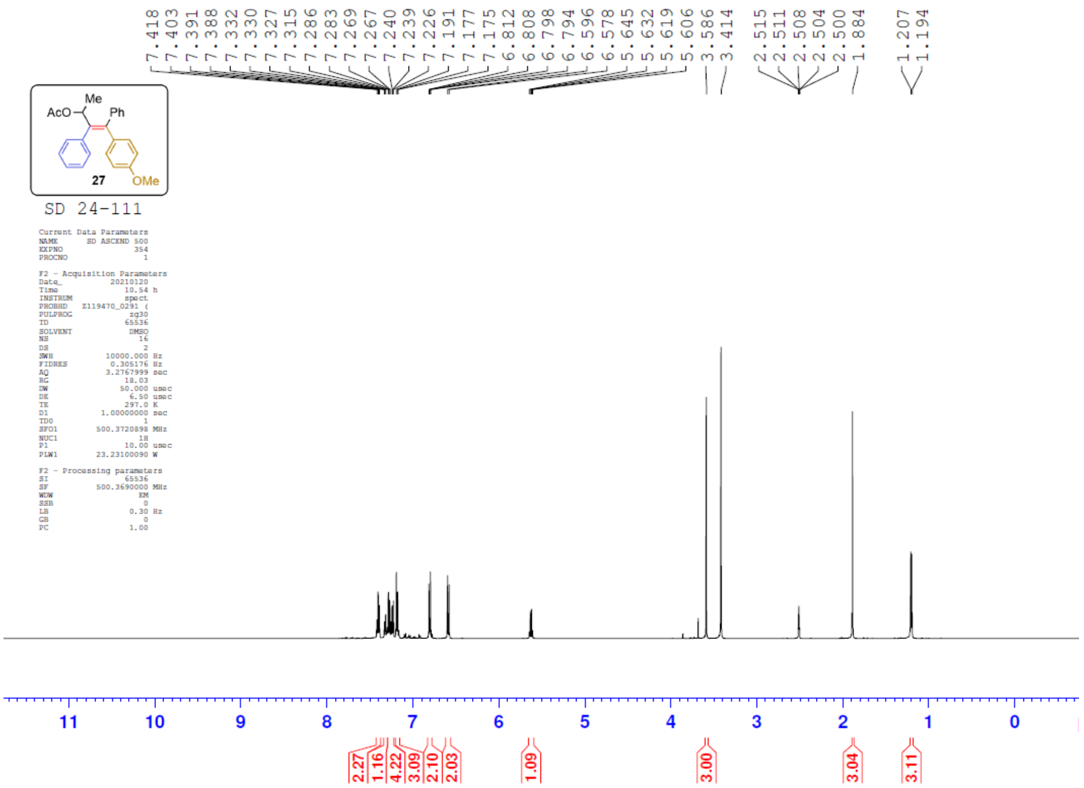

Supplementary Figure 96. ^{1}H NMR of compound **27**

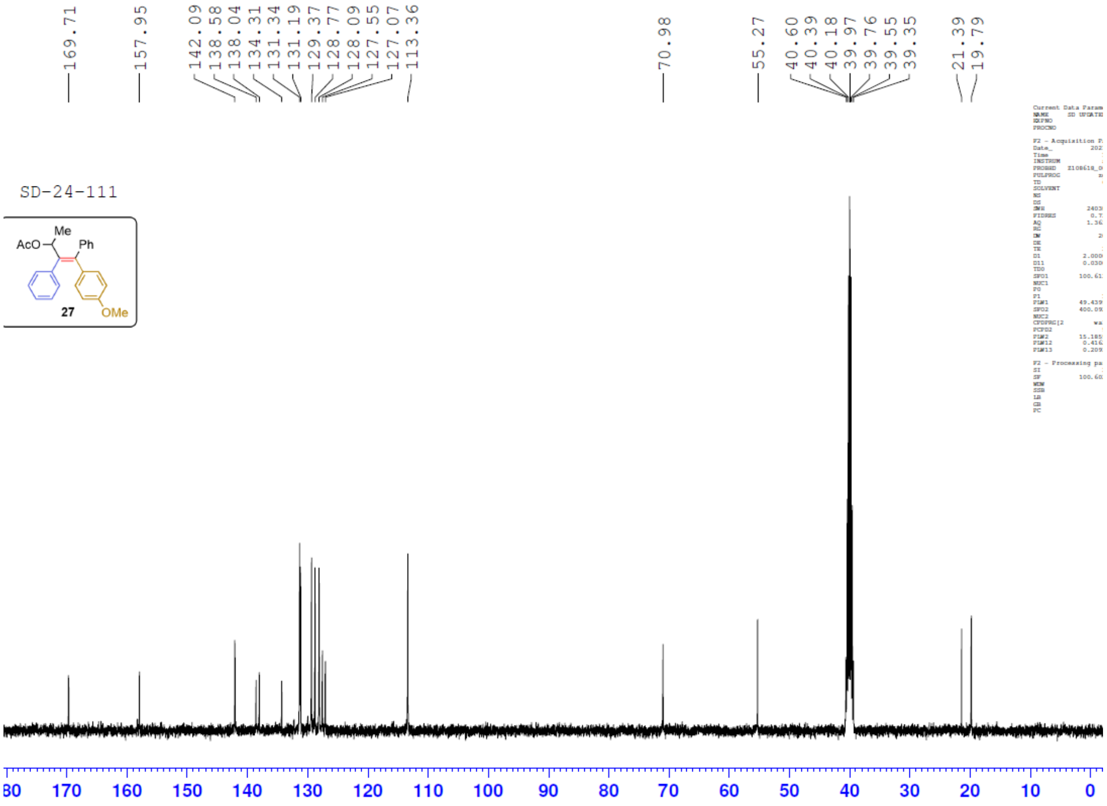

Supplementary Figure 97. ^{13}C NMR of compound **27**

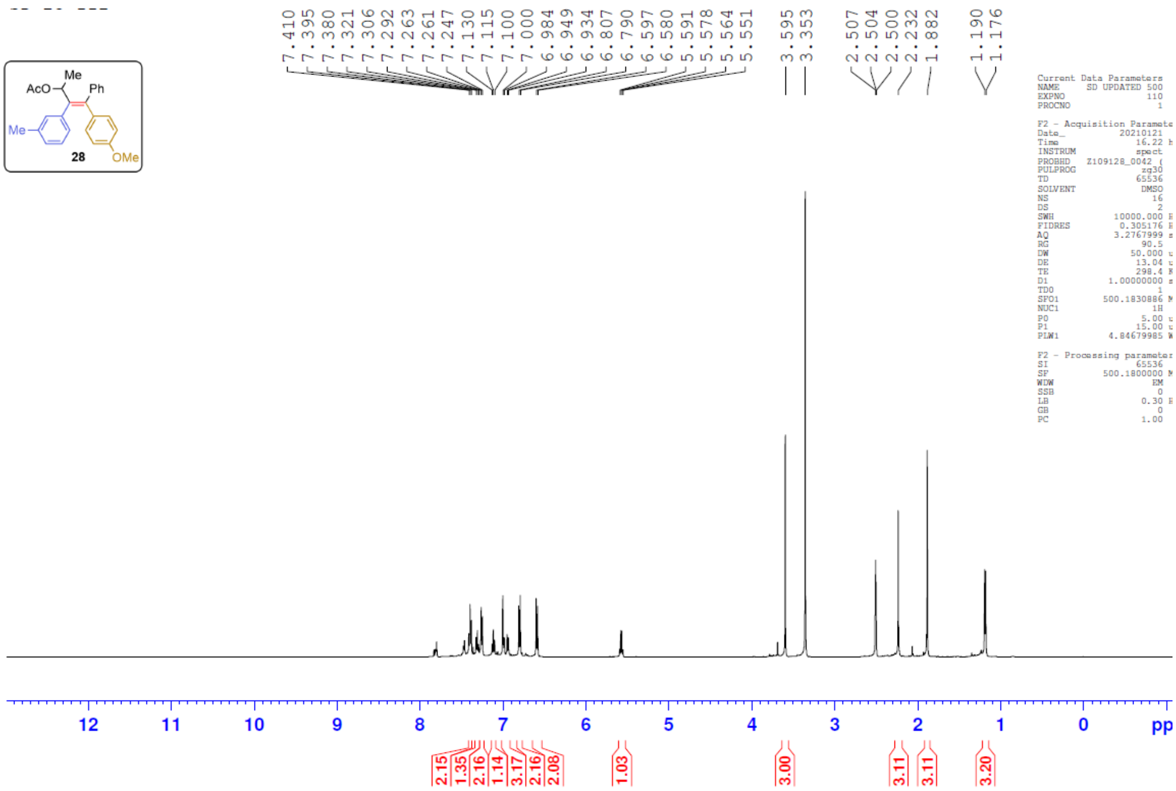

Supplementary Figure 98. ^{1}H NMR of compound **28**

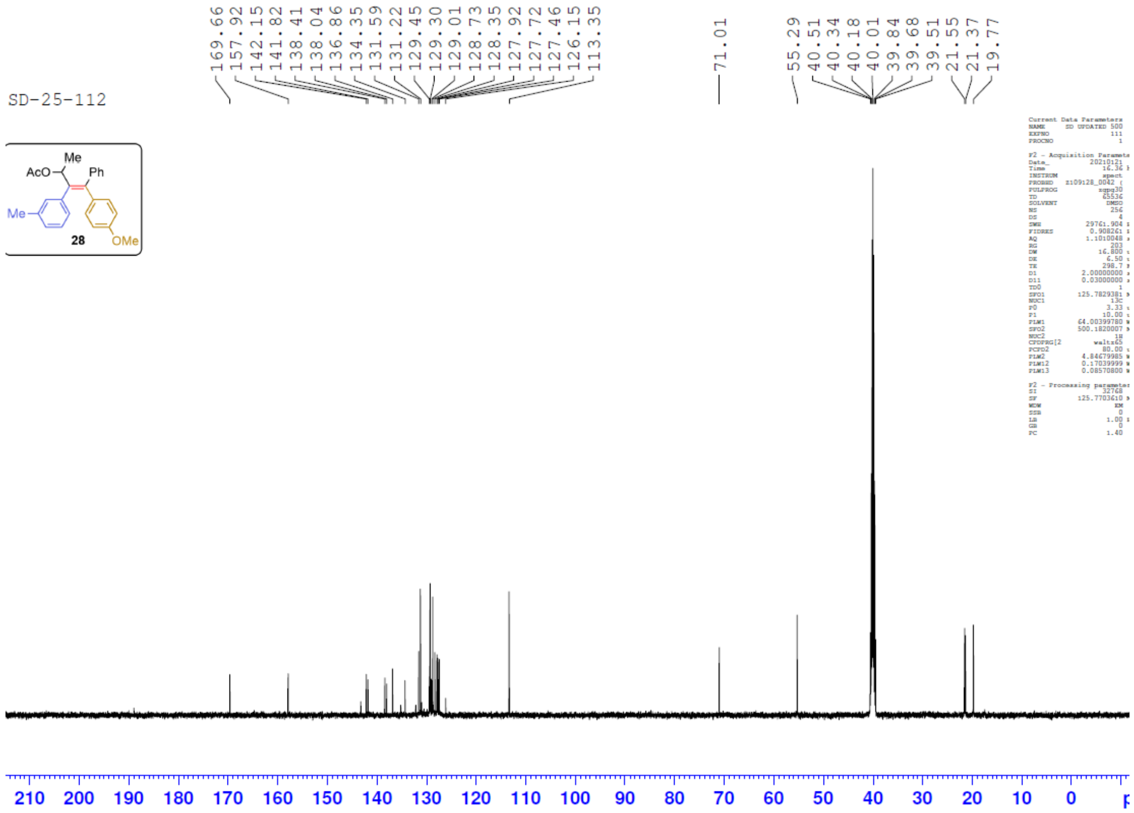

Supplementary Figure 99. ^{13}C NMR of compound **28**

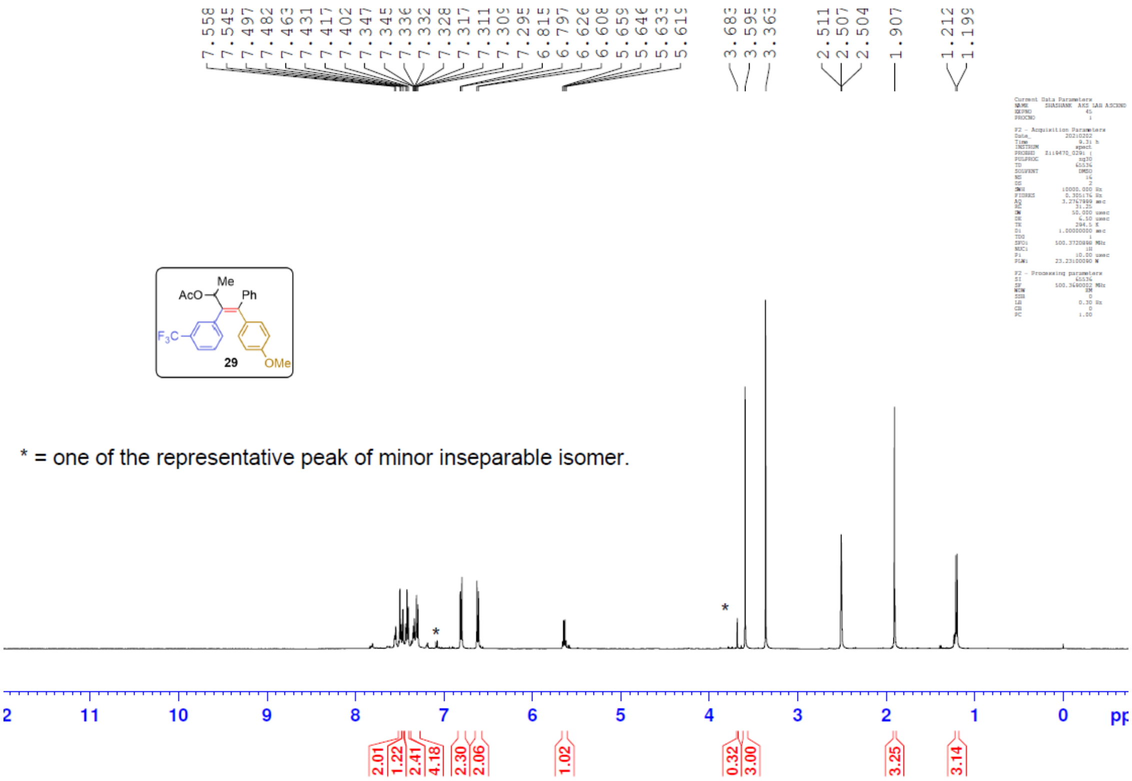

Supplementary Figure 100. ^{1}H NMR of compound **29**

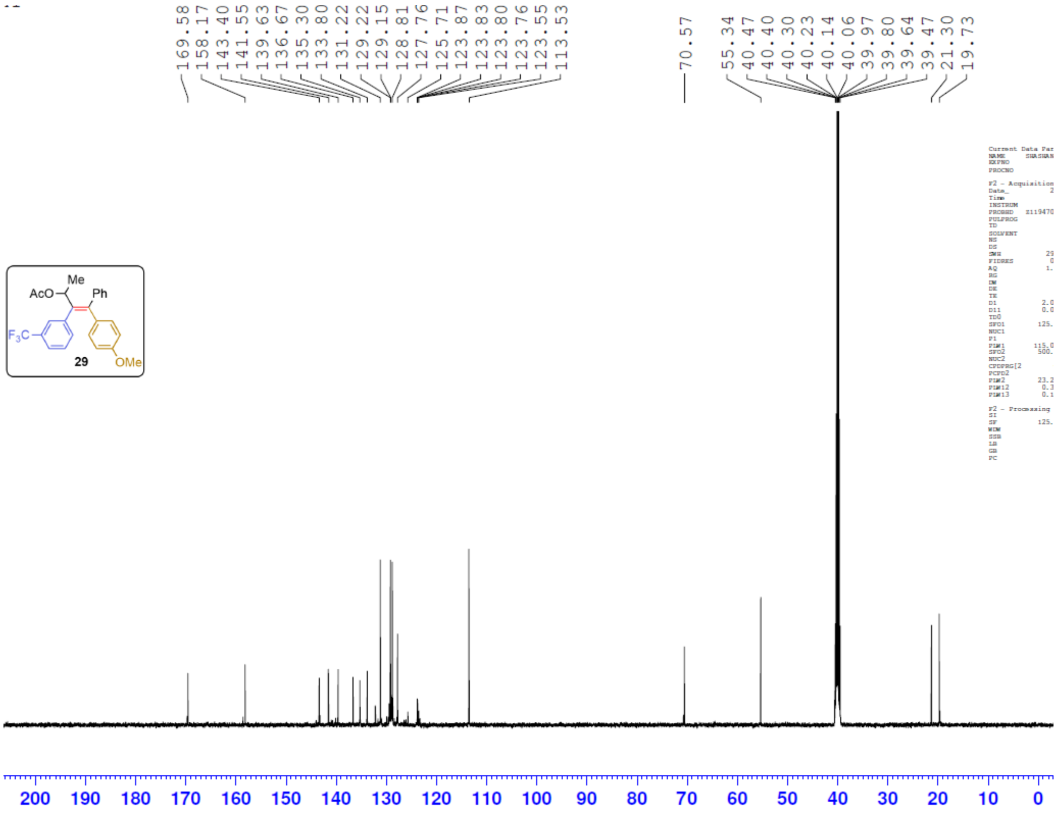

Supplementary Figure 101. ^{13}C NMR of compound **29**

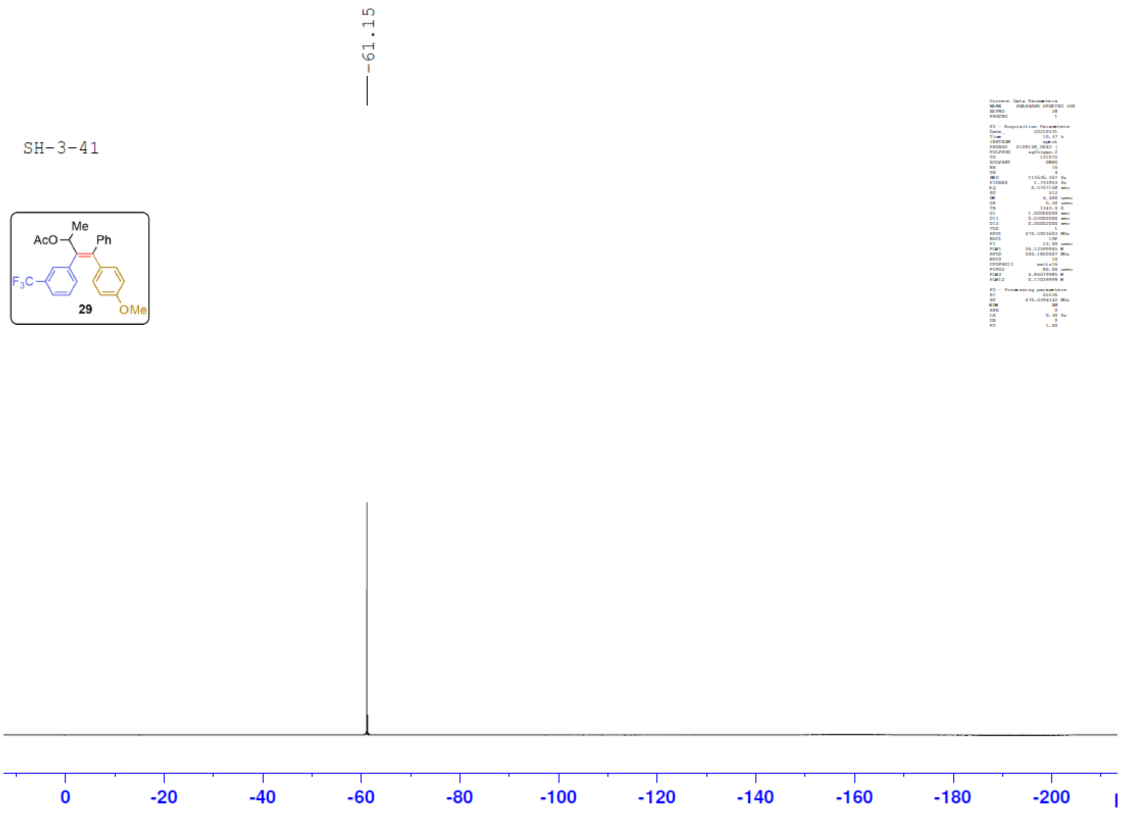

Supplementary Figure 102. ^{19}F NMR of compound **29**

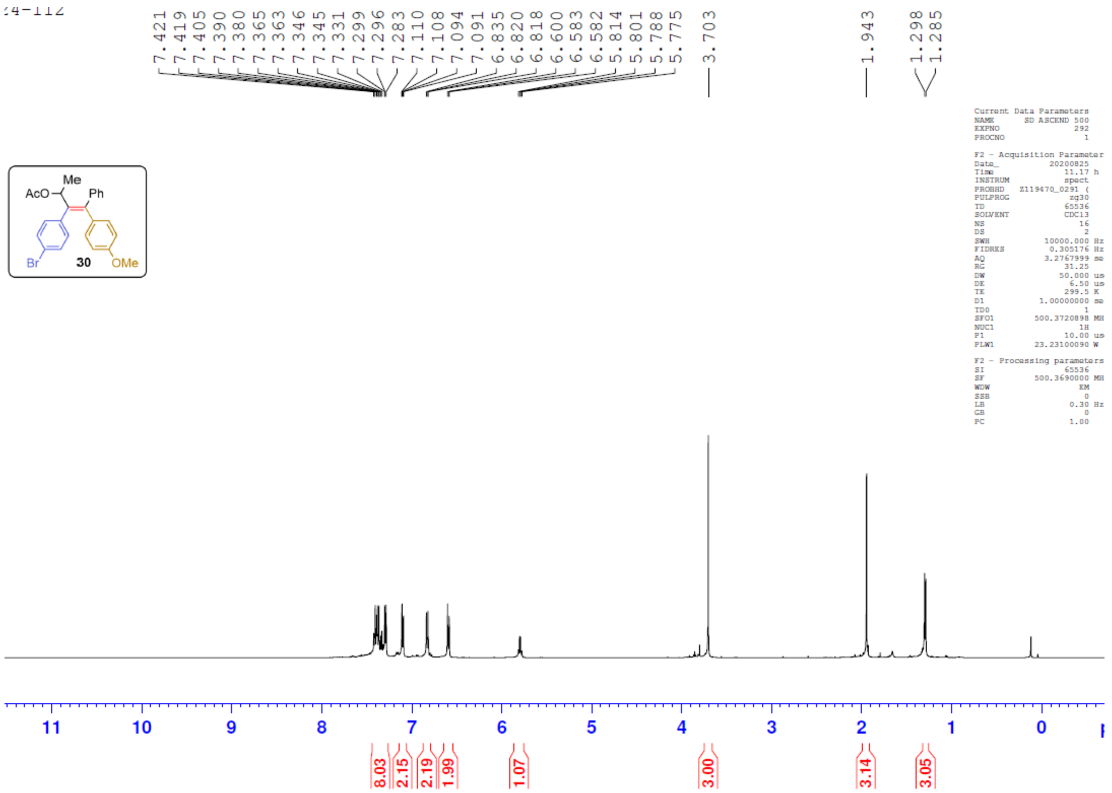

Supplementary Figure 103. ^{1}H NMR of compound **30**

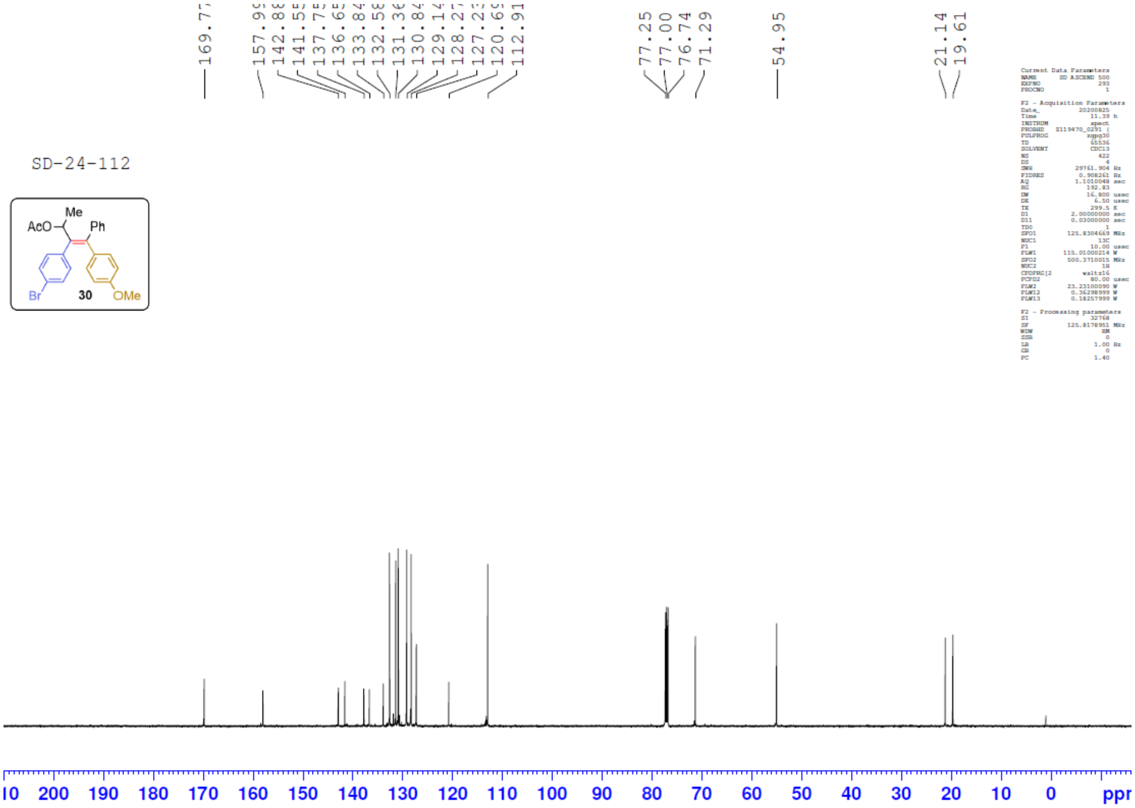

Supplementary Figure 104. ^{13}C NMR of compound **30**

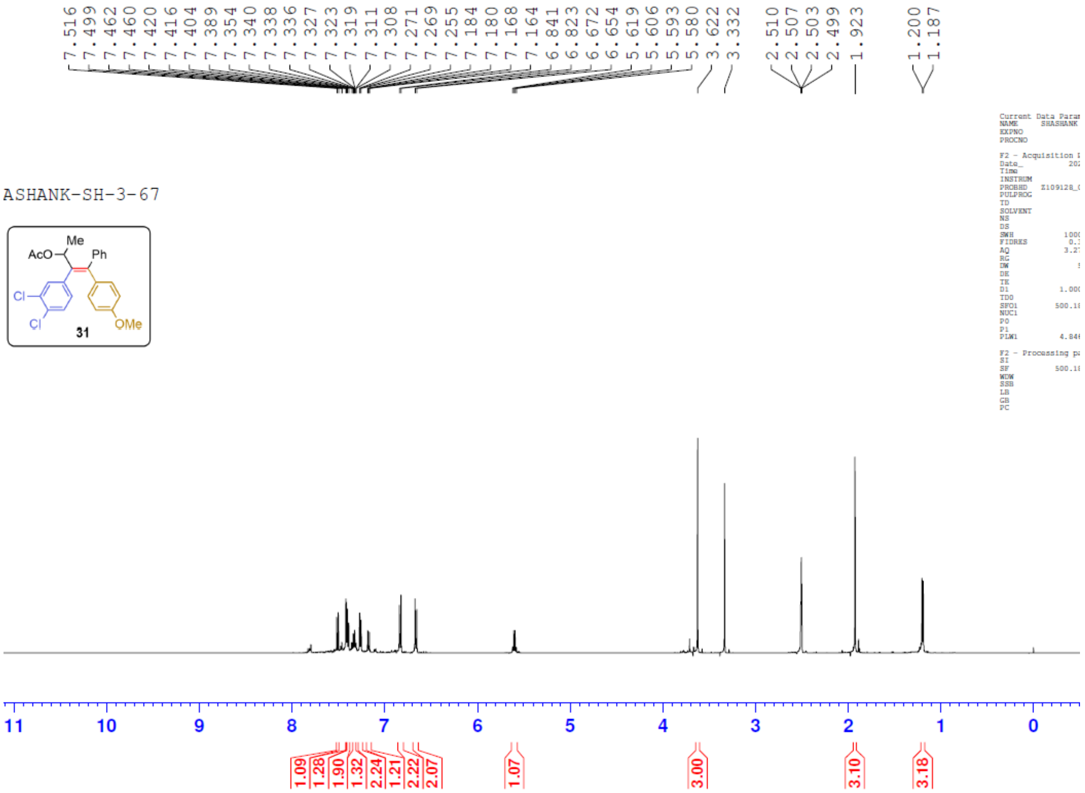

Supplementary Figure 105. ^{1}H NMR of compound **31**

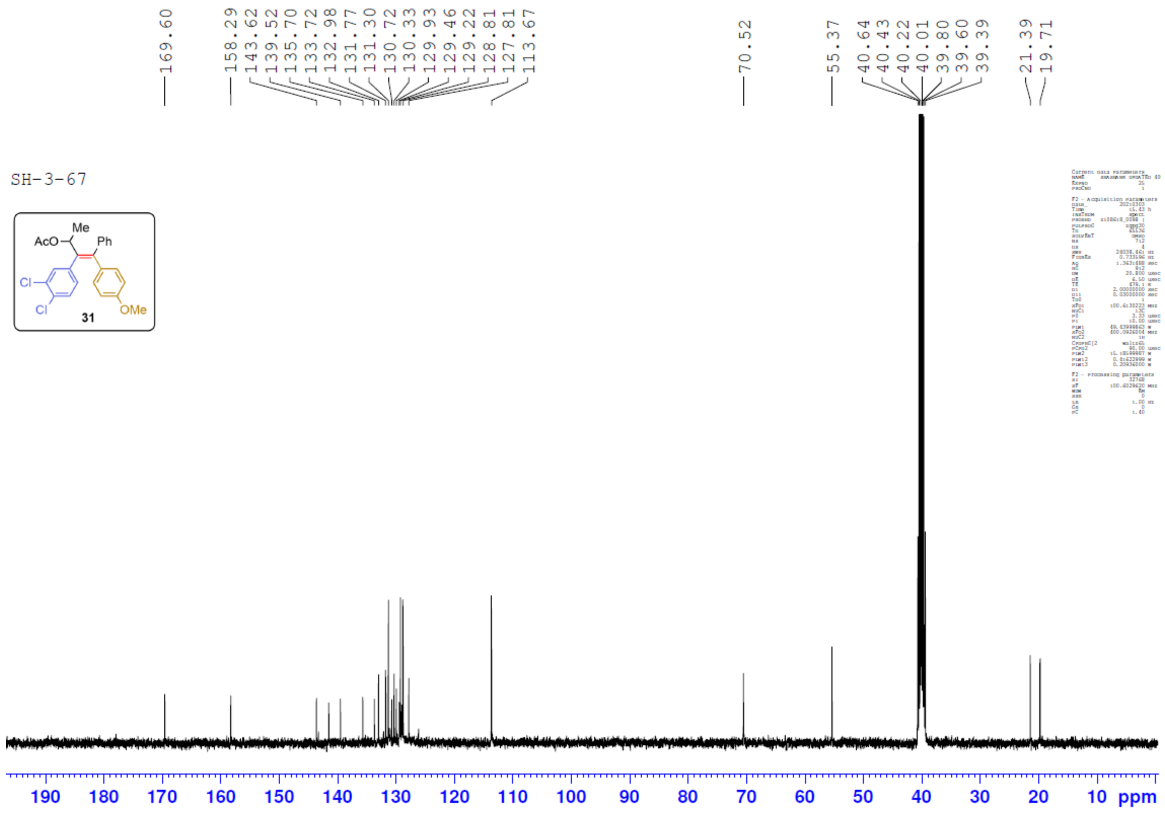

Supplementary Figure 106. ^{13}C NMR of compound **31**

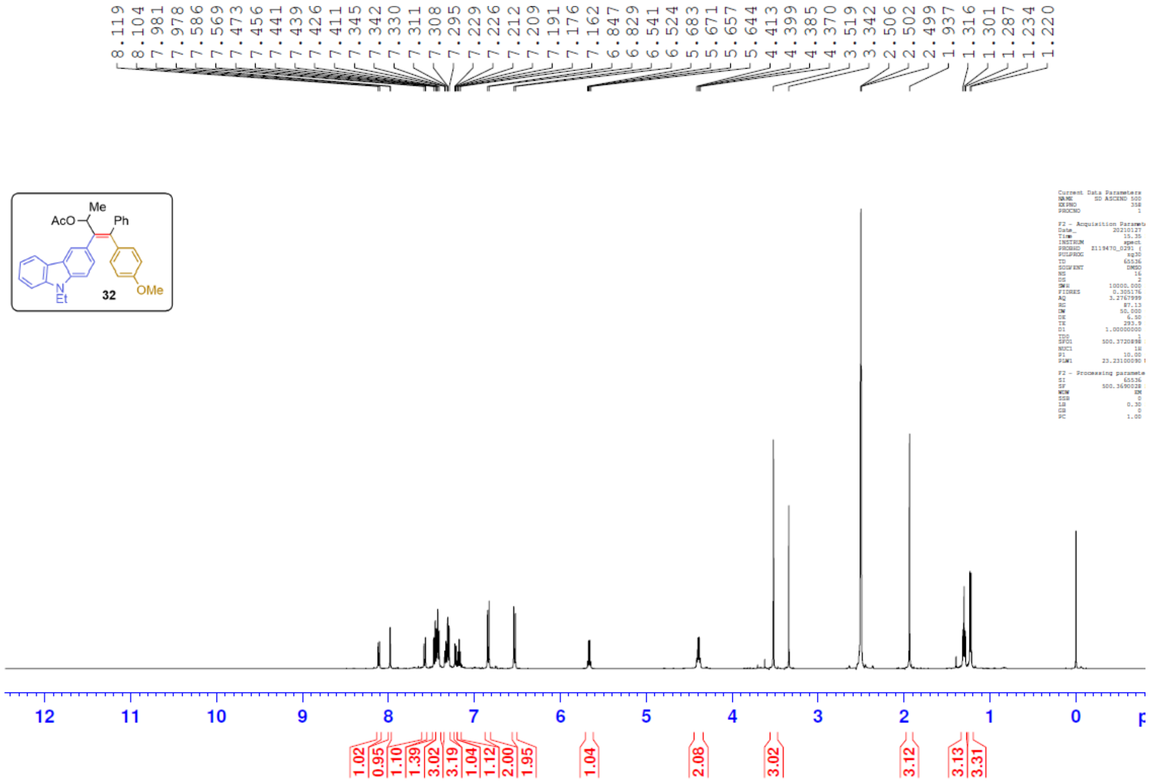

Supplementary Figure 107. ^{1}H NMR of compound **32**

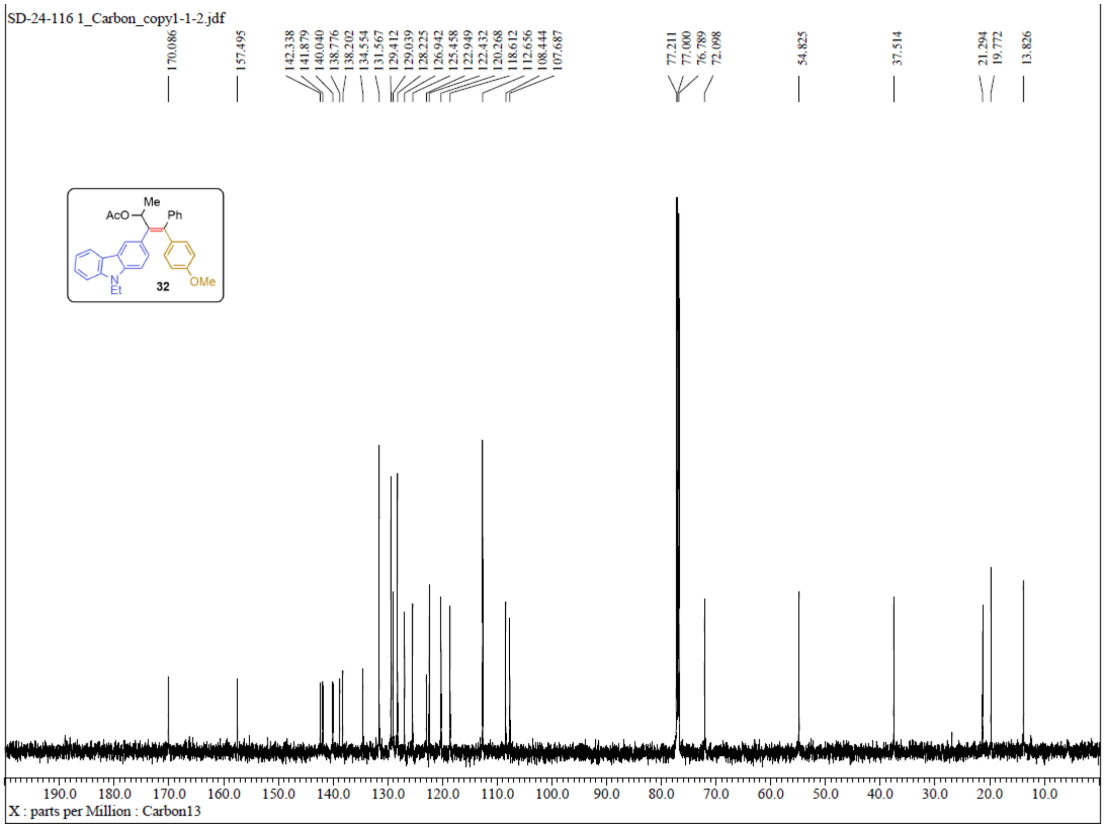

Supplementary Figure 108. ^{13}C NMR of compound **32**

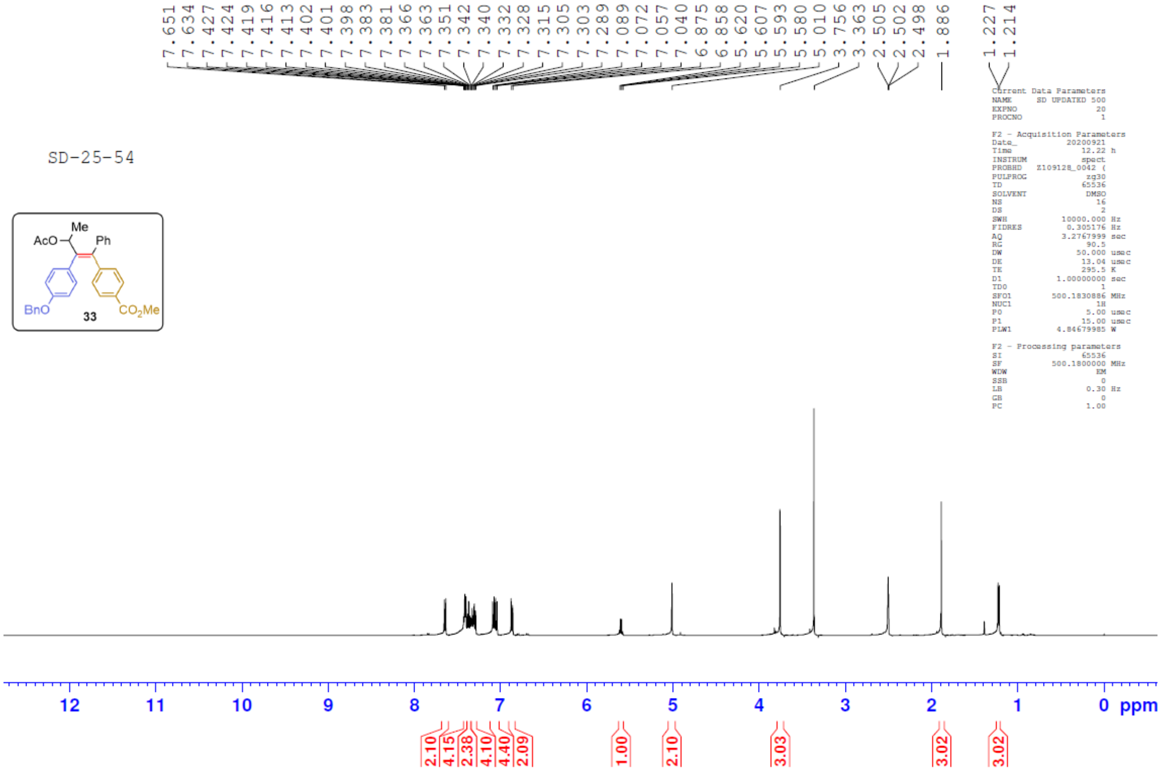

Supplementary Figure 109. ^{1}H NMR of compound **33**

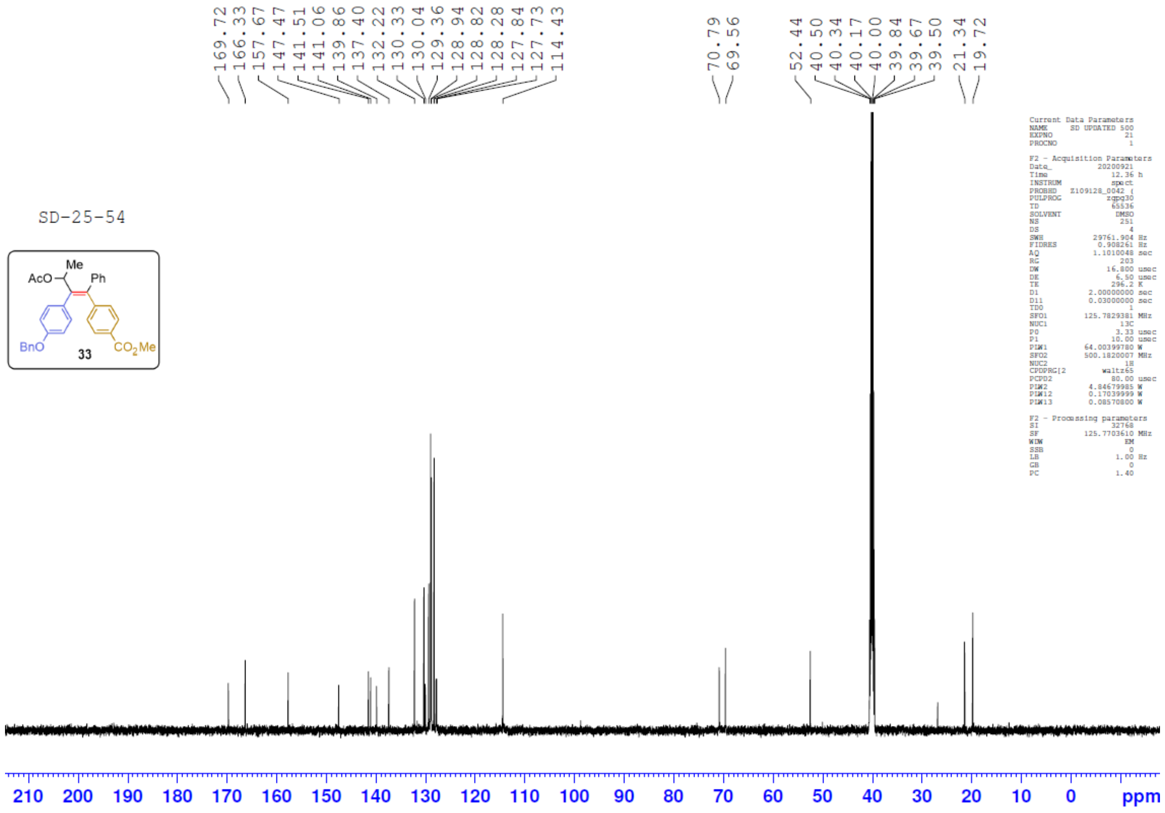

Supplementary Figure 110. ^{13}C NMR of compound **33**

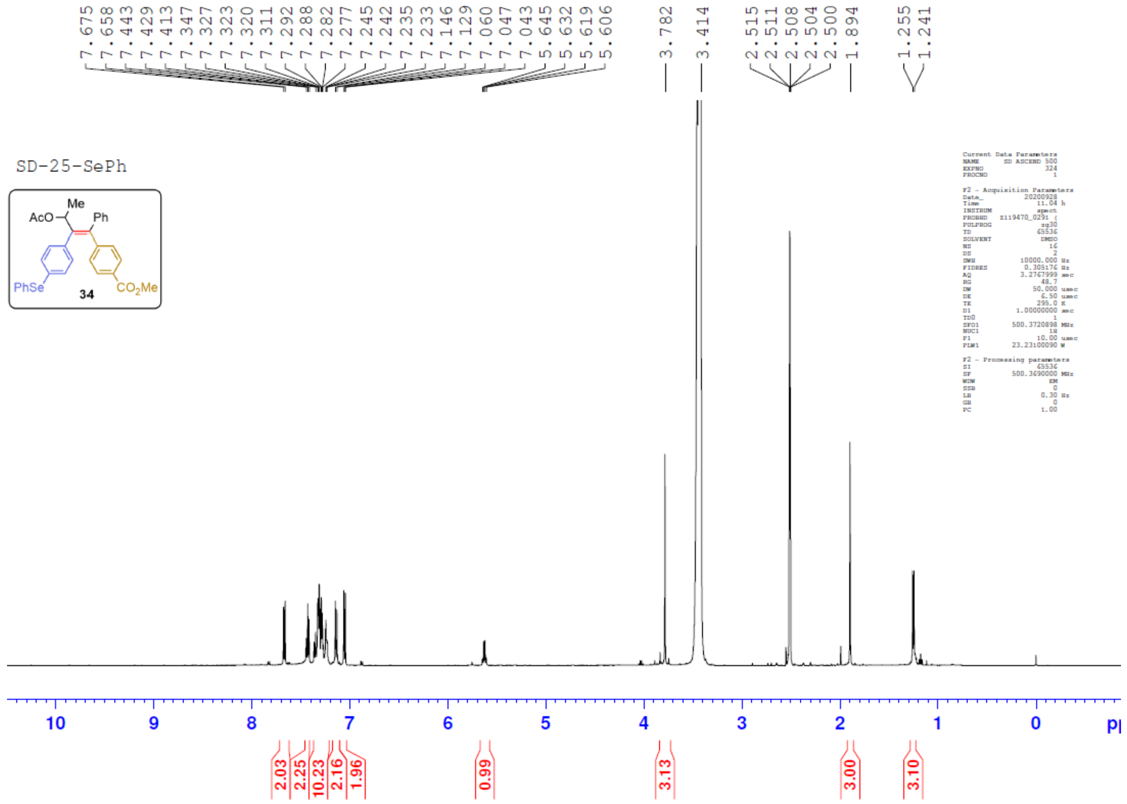

Supplementary Figure 111. ^{1}H NMR of compound **34**

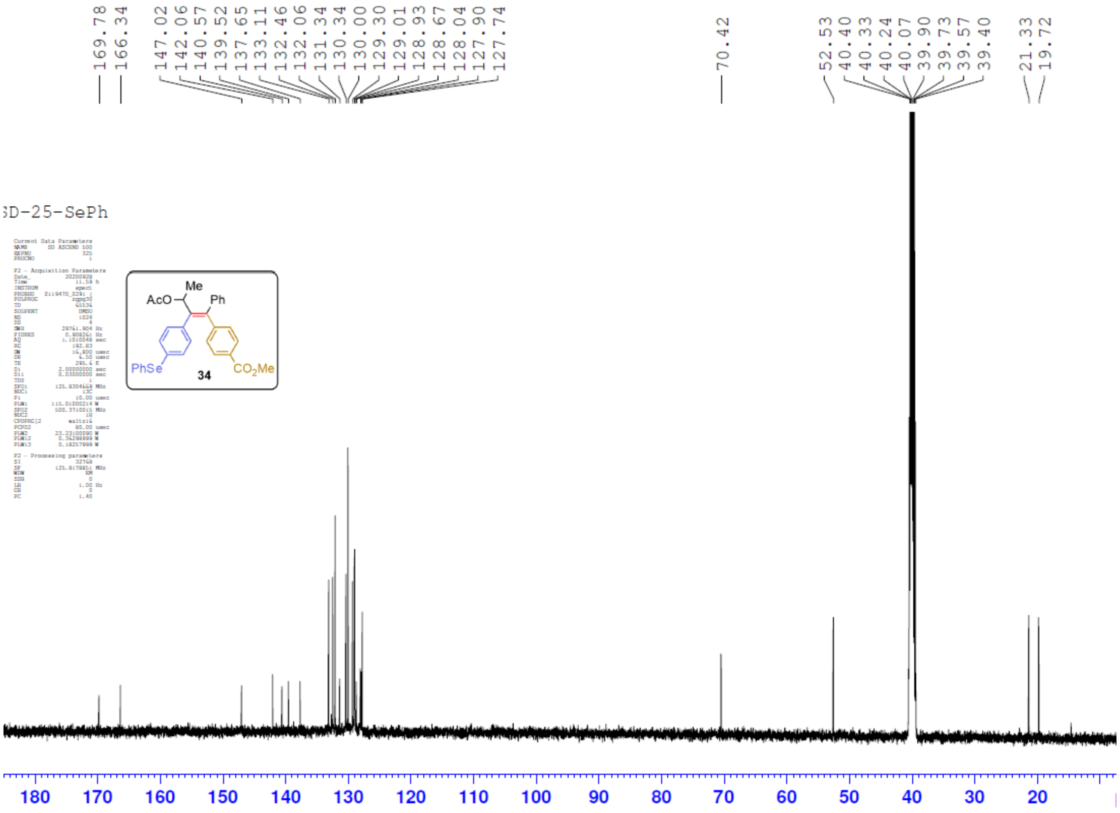

Supplementary Figure 112. ^{13}C NMR of compound **34**

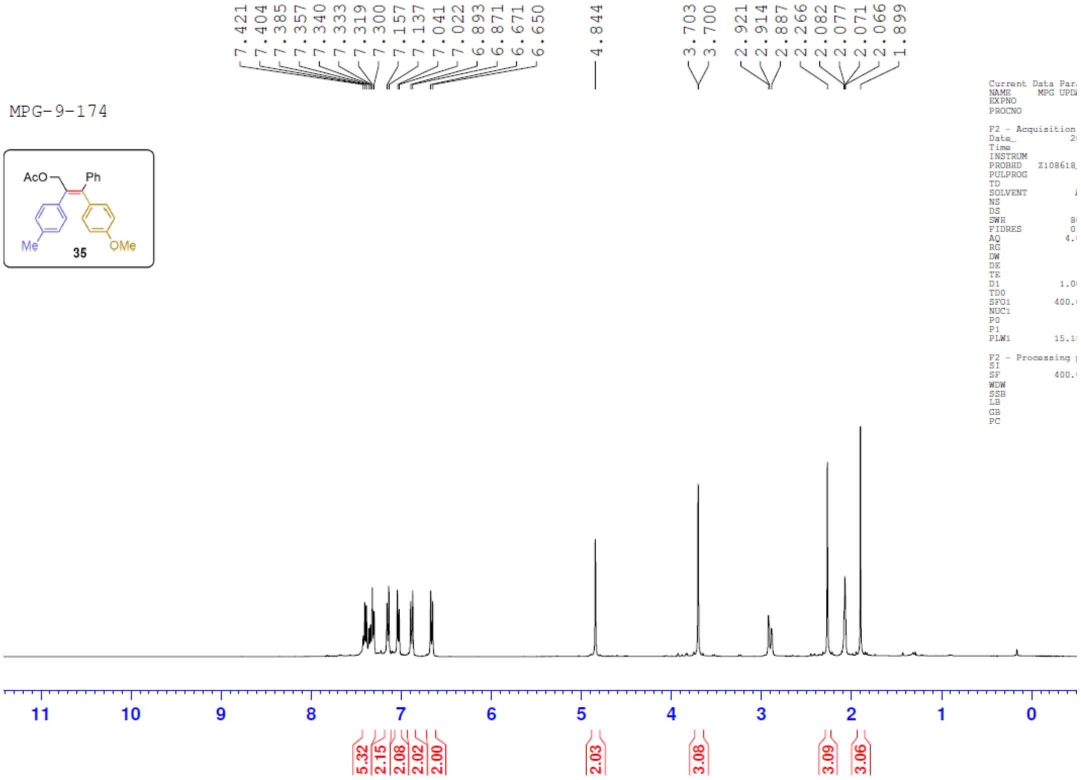

Supplementary Figure 113. ^{1}H NMR of compound **35**

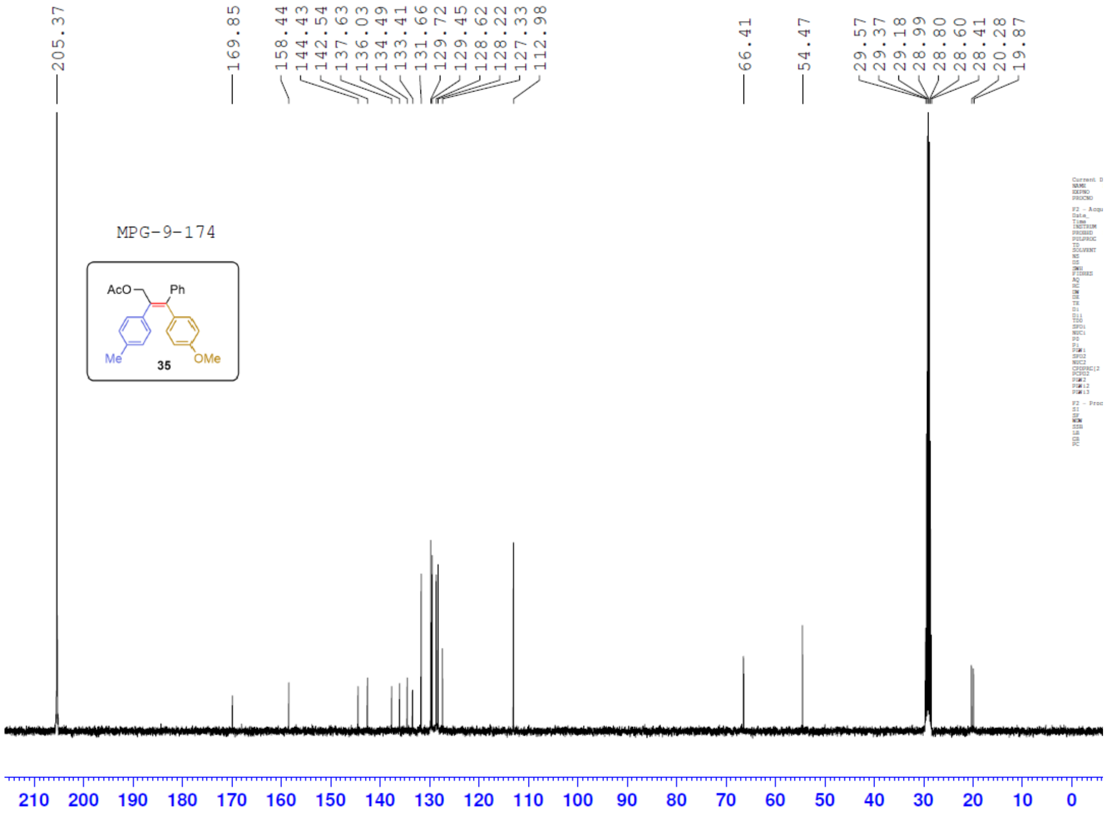

Supplementary Figure 114. ^{13}C NMR of compound **35**

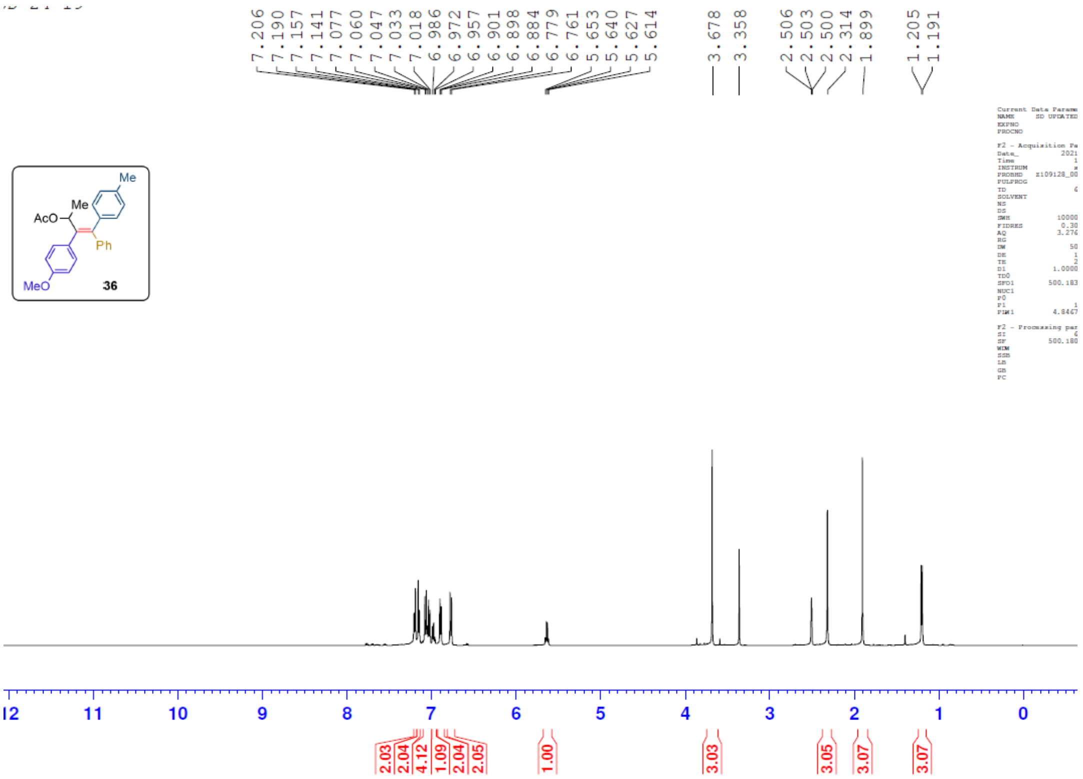

Supplementary Figure 115. ^{1}H NMR of compound **36**

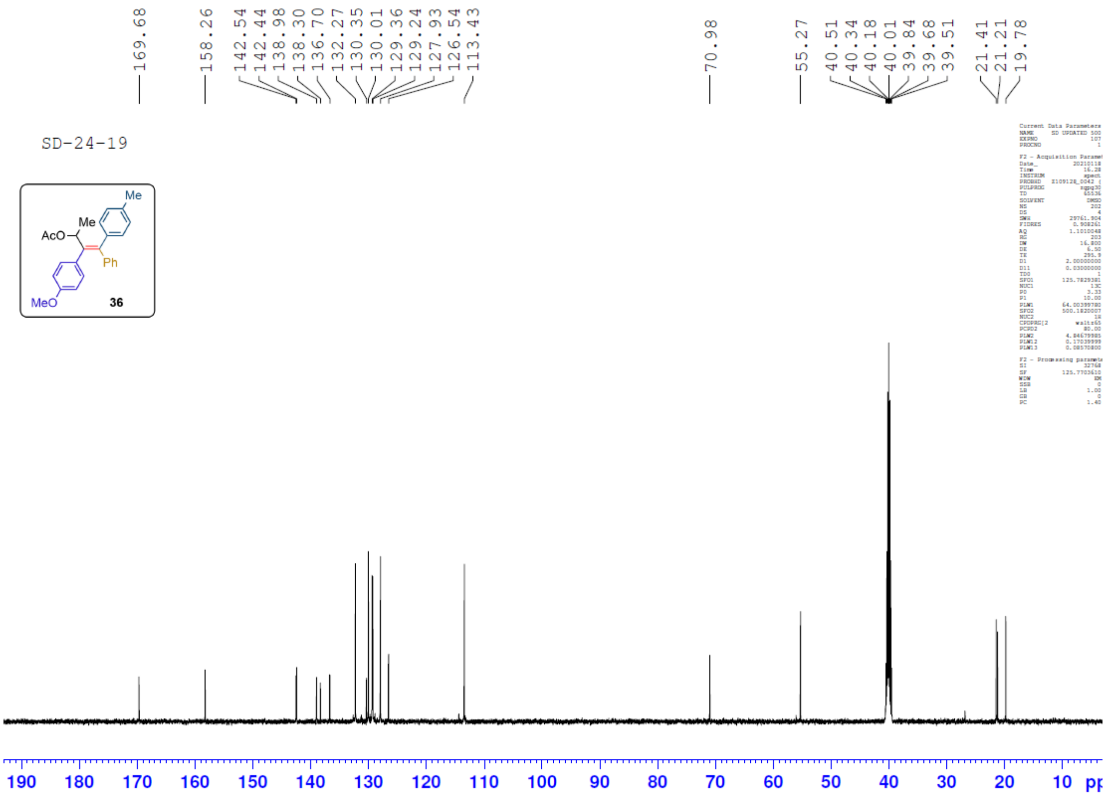

Supplementary Figure 116. ^{13}C NMR of compound **36**

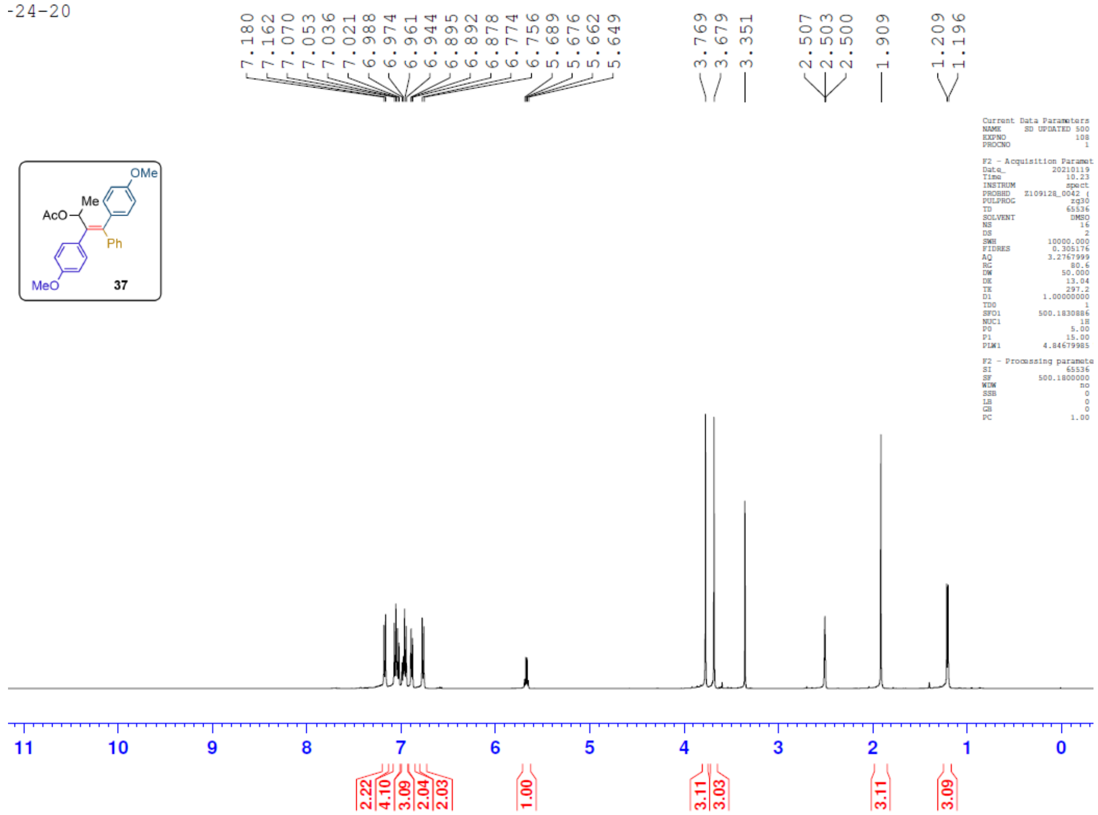

Supplementary Figure 117. ^{1}H NMR of compound **37**

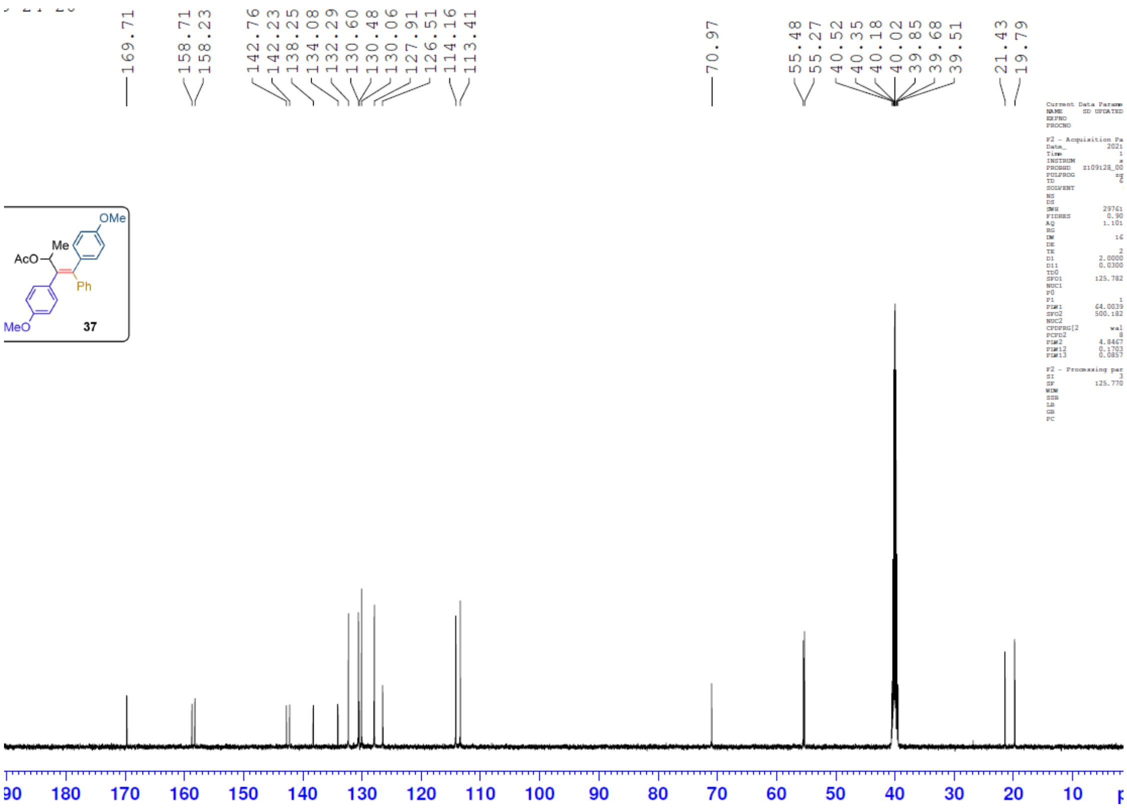

Supplementary Figure 118. ^{13}C NMR of compound **37**

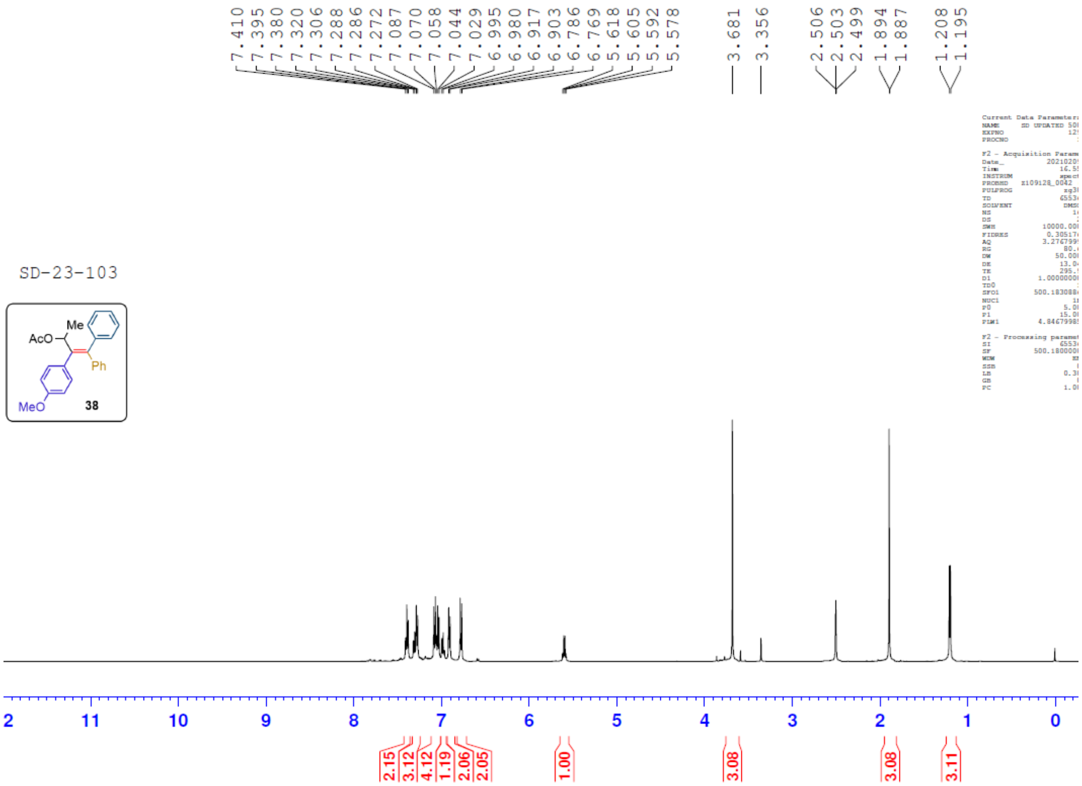

Supplementary Figure 119. ^{1}H NMR of compound **38**

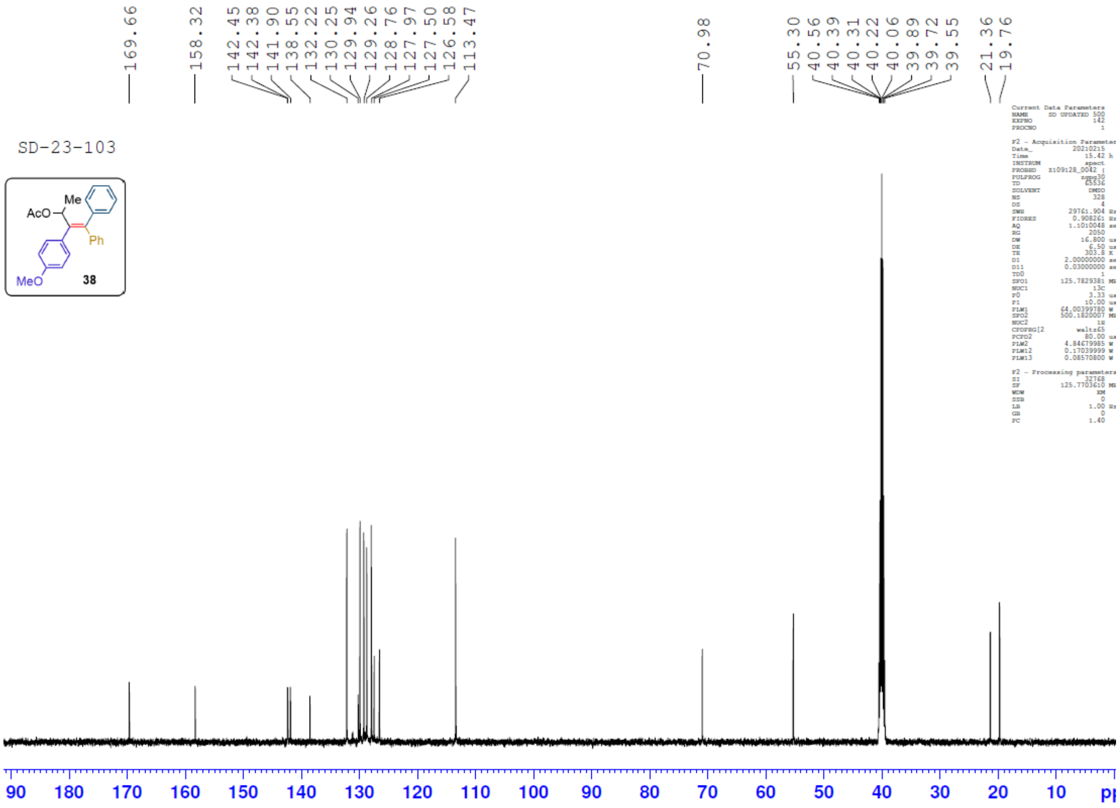

Supplementary Figure 120. ^{13}C NMR of compound **38**

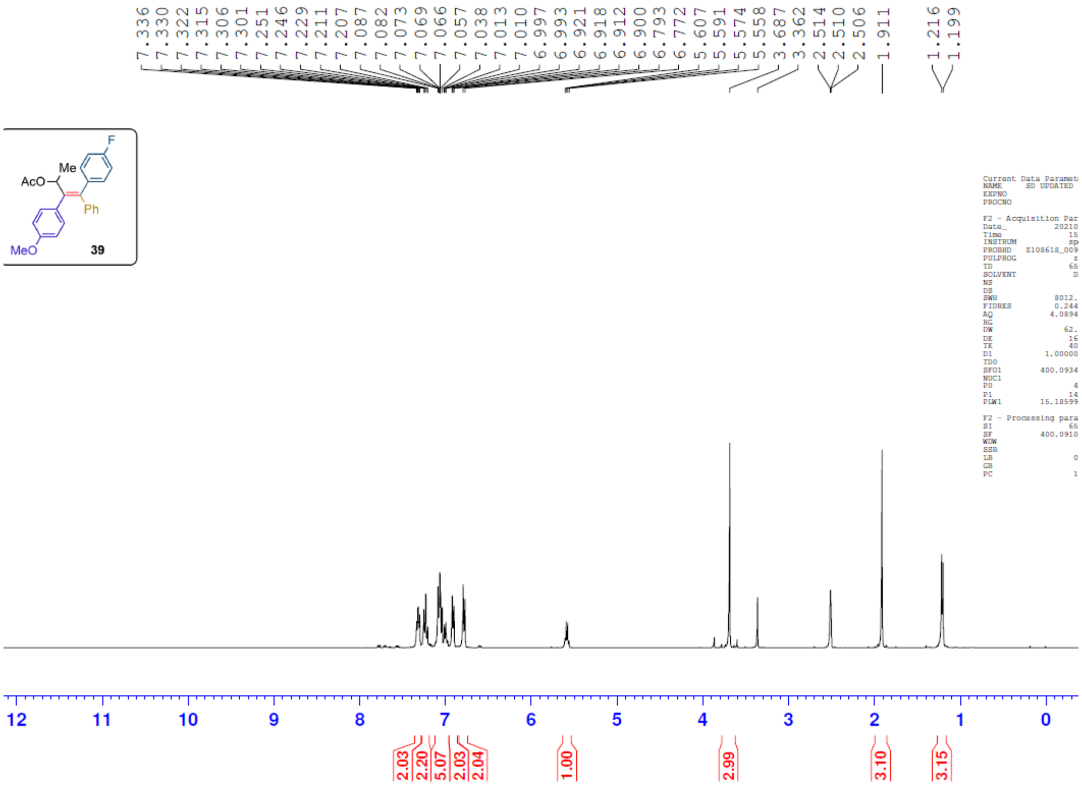

Supplementary Figure 121. ^{1}H NMR of compound **39**

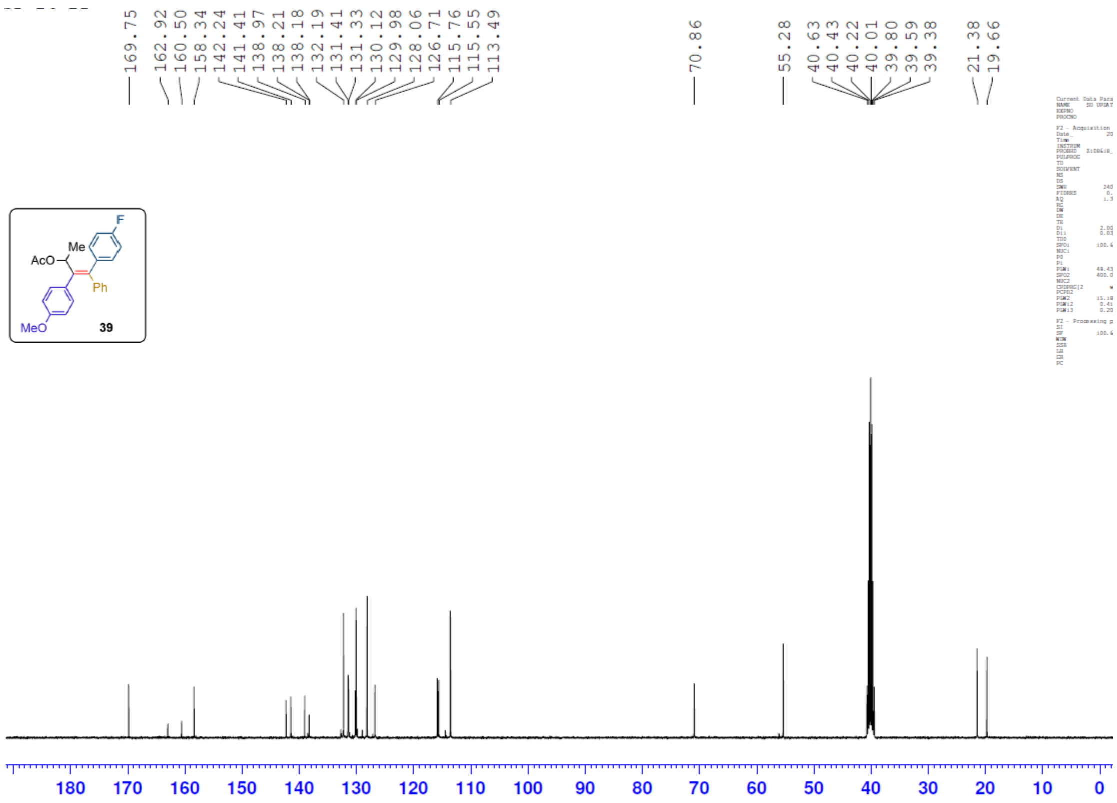

Supplementary Figure 122. ^{13}C NMR of compound **39**

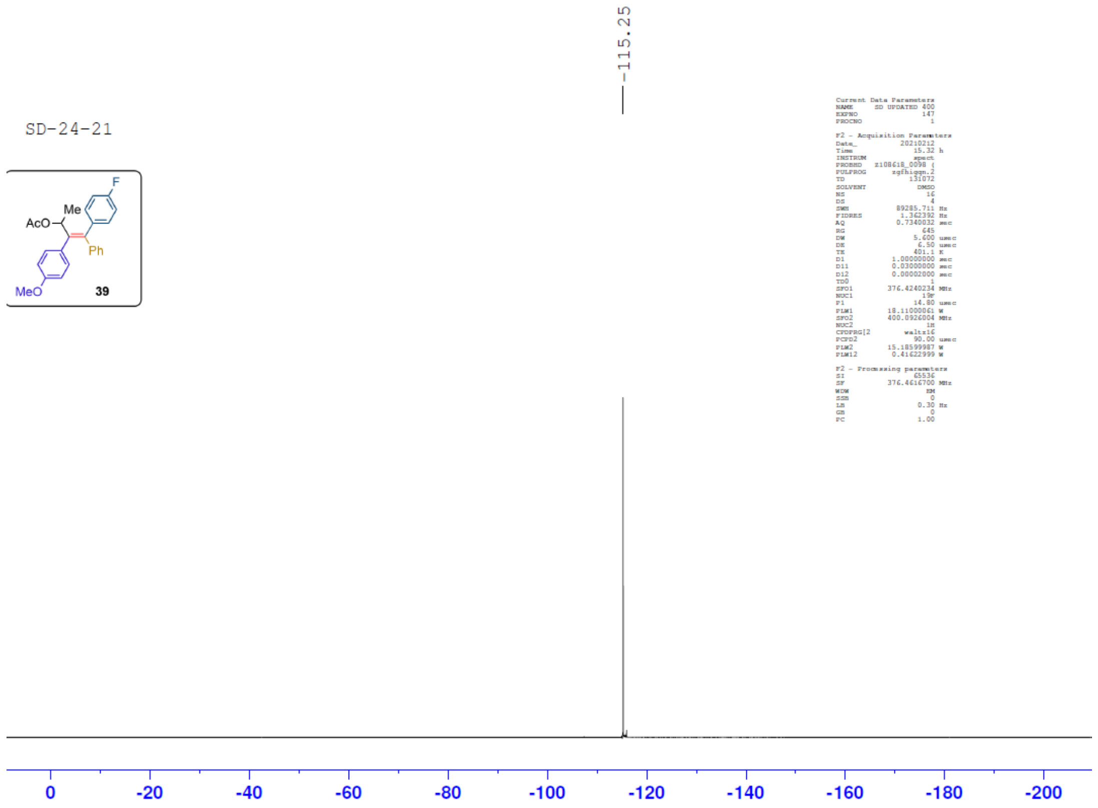

Supplementary Figure 123. ^{19}F NMR of compound **39**

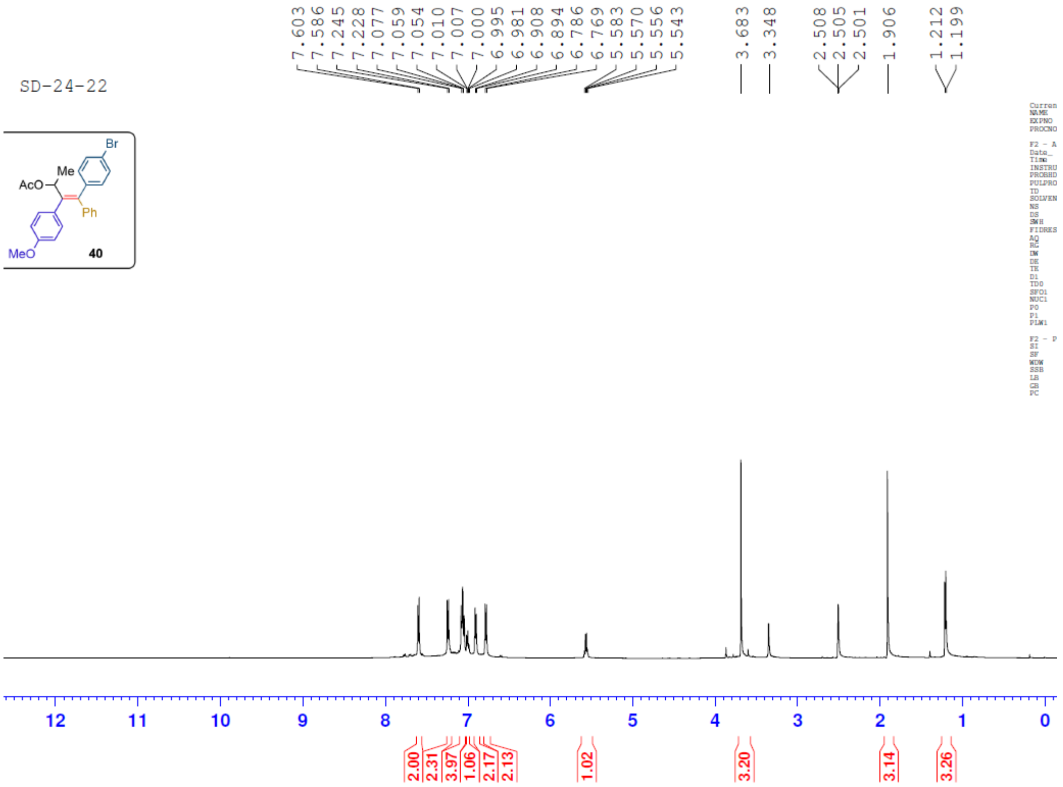

Supplementary Figure 124. ^{1}H NMR of compound **40**

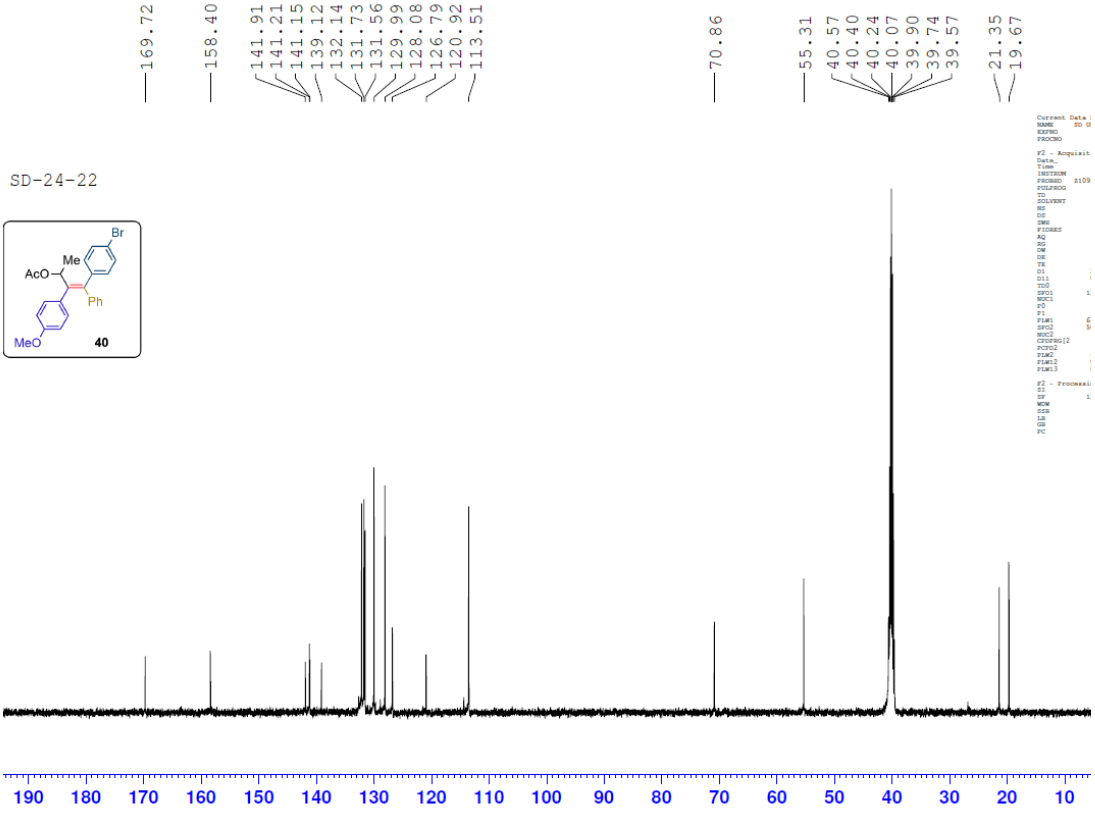

Supplementary Figure 125. ^{13}C NMR of compound **40**

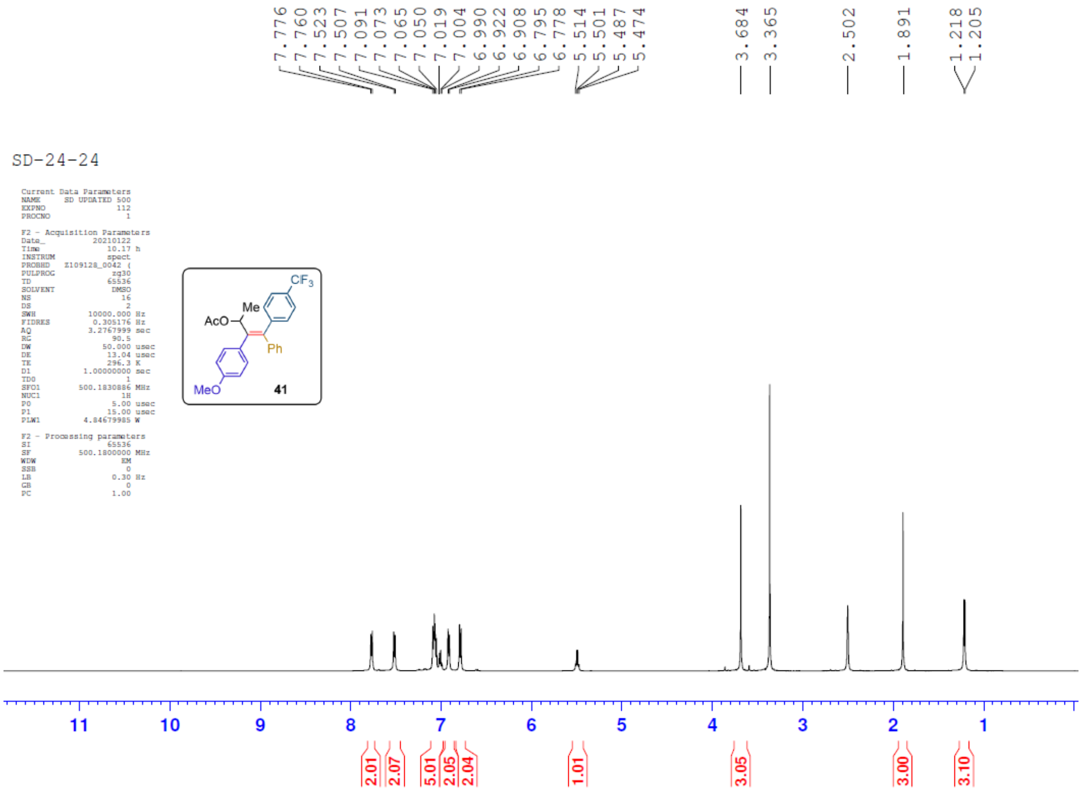

Supplementary Figure 126. ^{1}H NMR of compound **41**

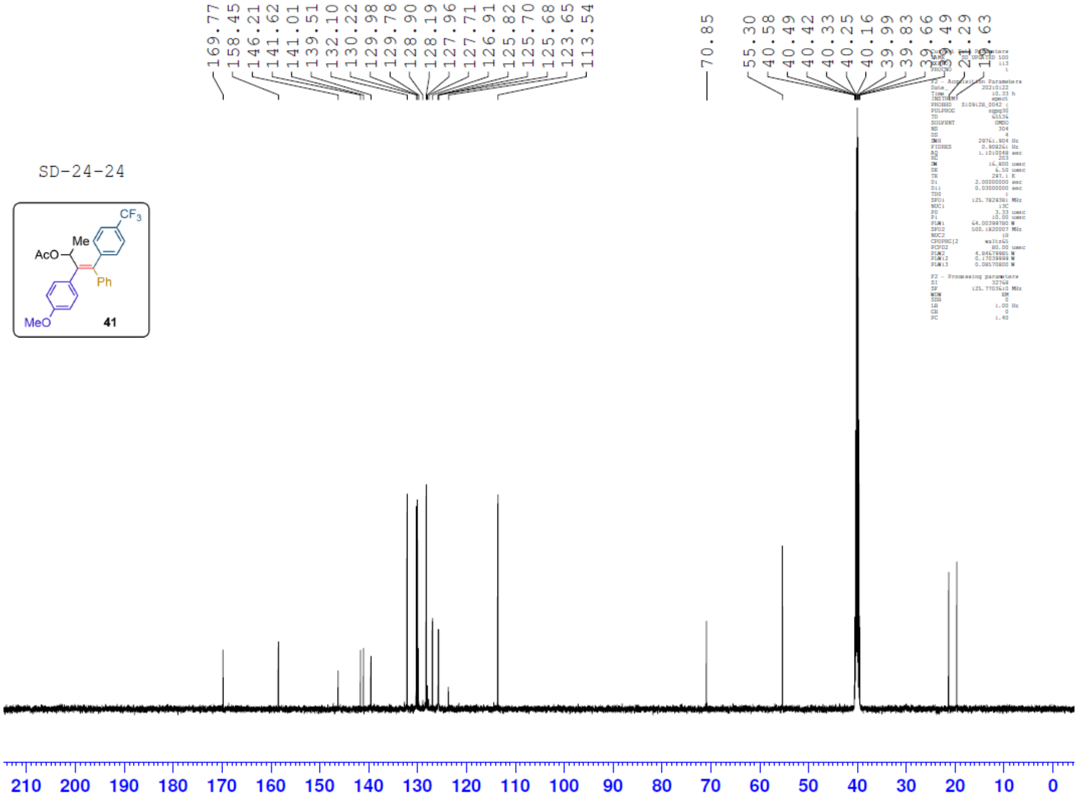

Supplementary Figure 127. ^{13}C NMR of compound **41**

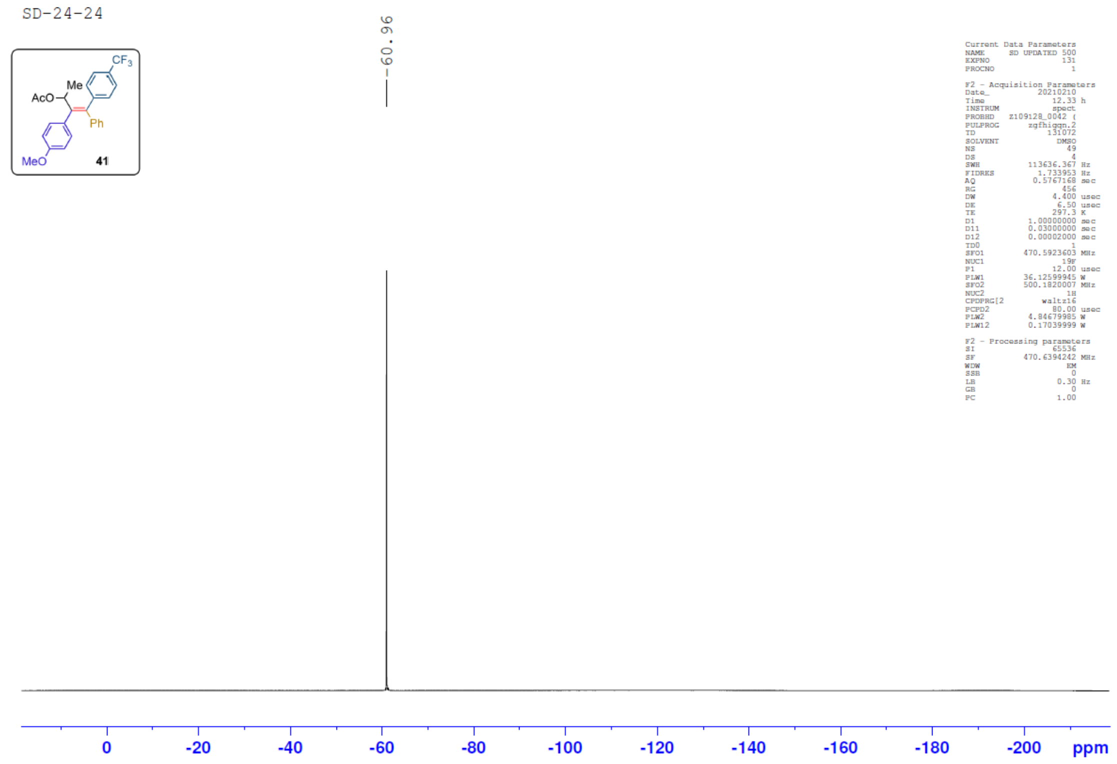

Supplementary Figure 128. ^{19}F NMR of compound **41**

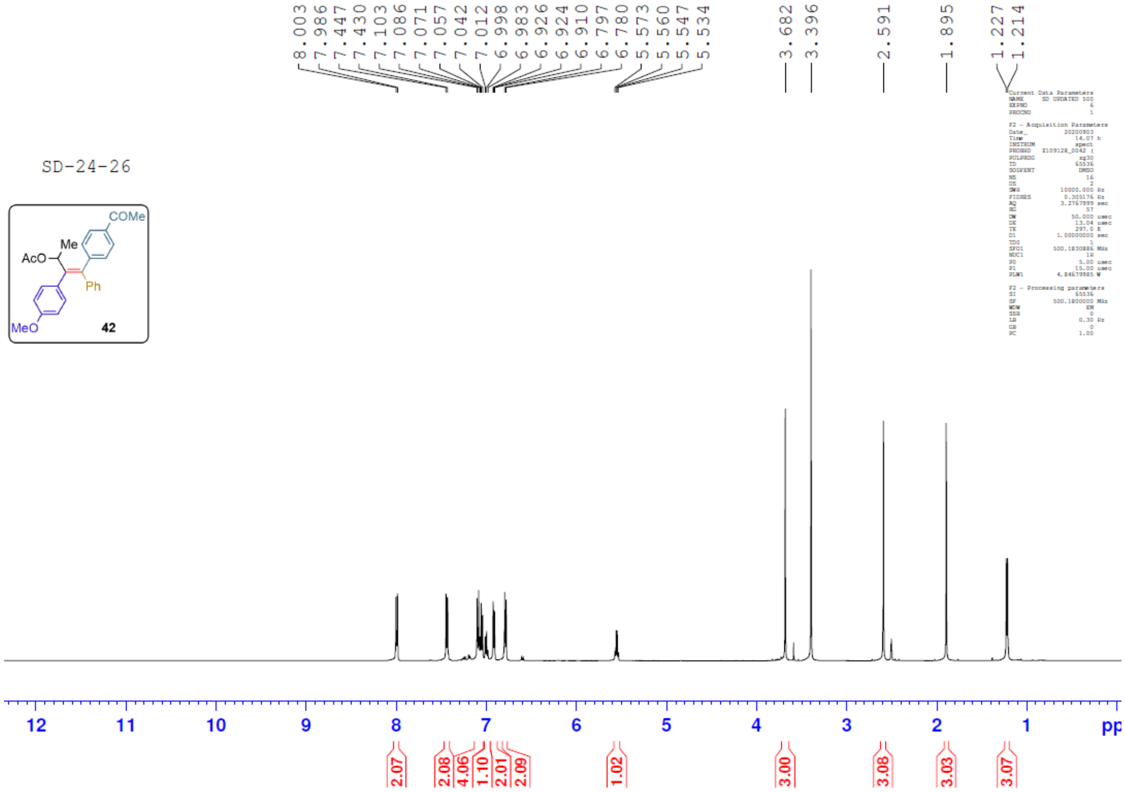

Supplementary Figure 129. ^{1}H NMR of compound **42**

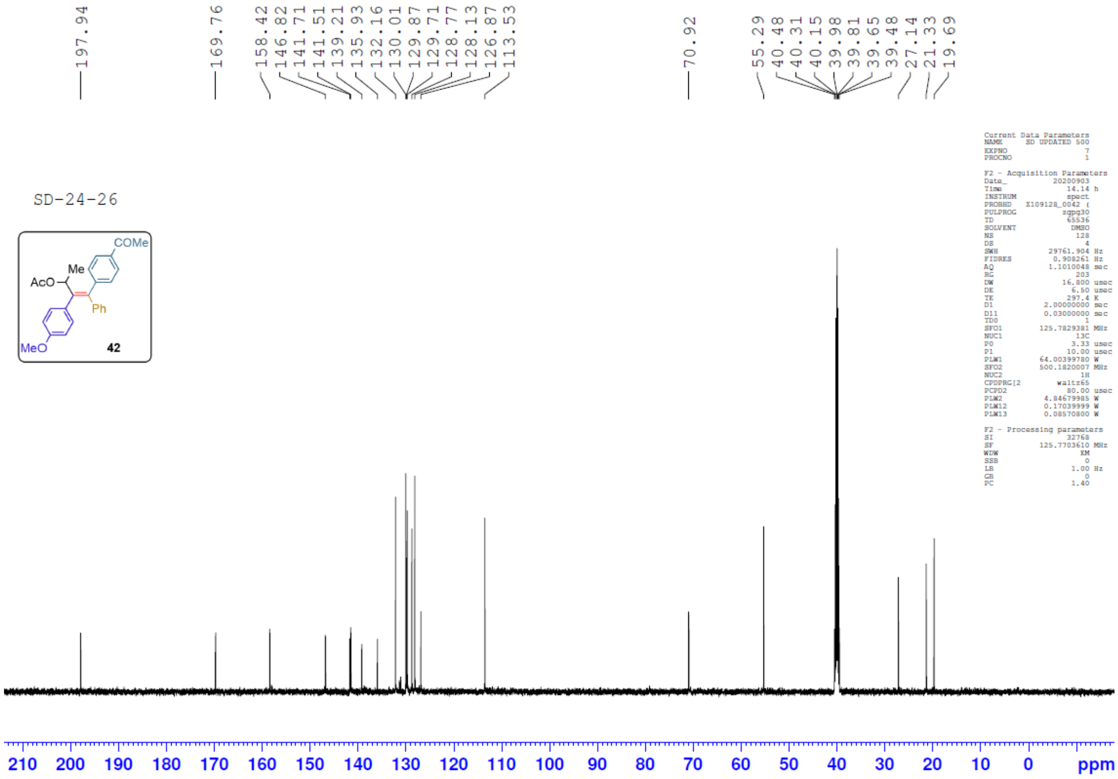

Supplementary Figure 130. ^{13}C NMR of compound **42**

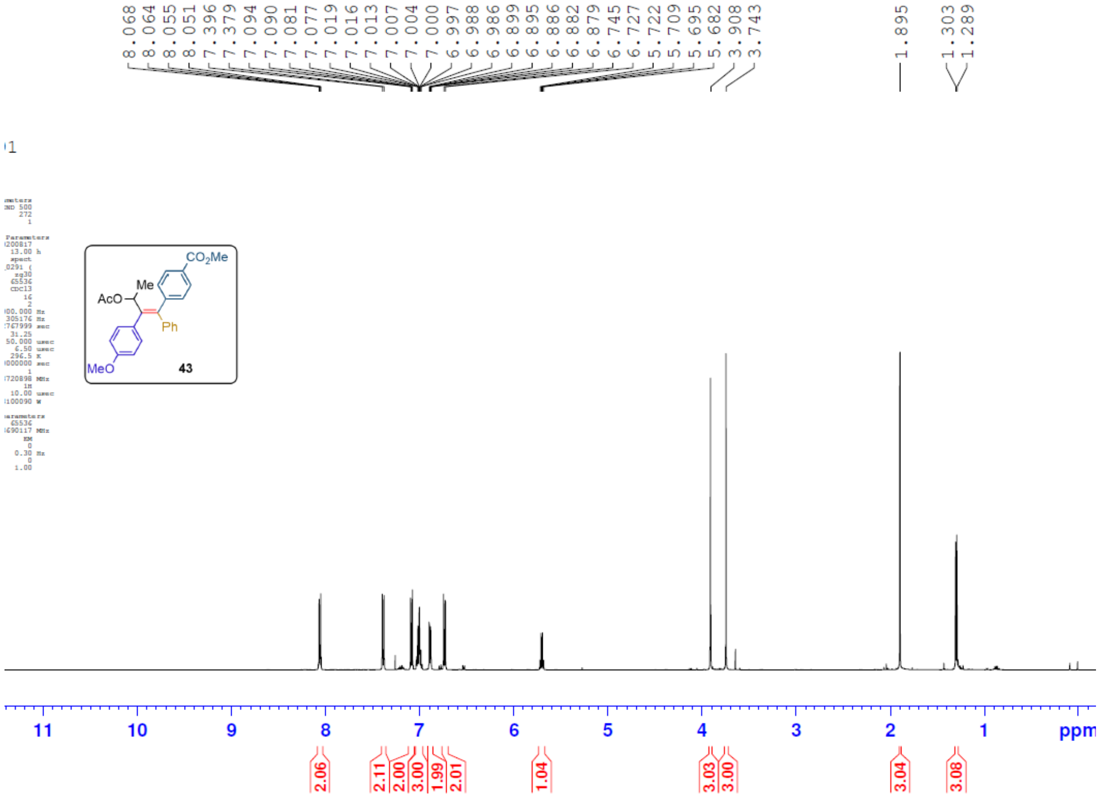

Supplementary Figure 131. ^{1}H NMR of compound **43**

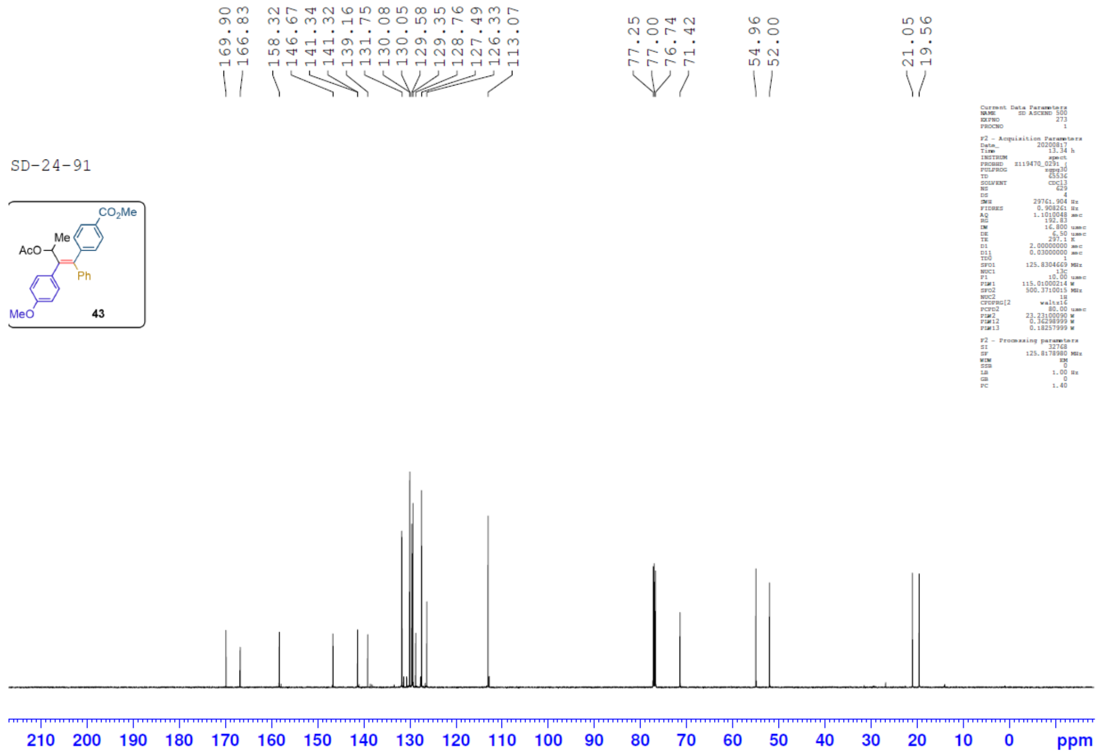

Supplementary Figure 132. ^{13}C NMR of compound **43**

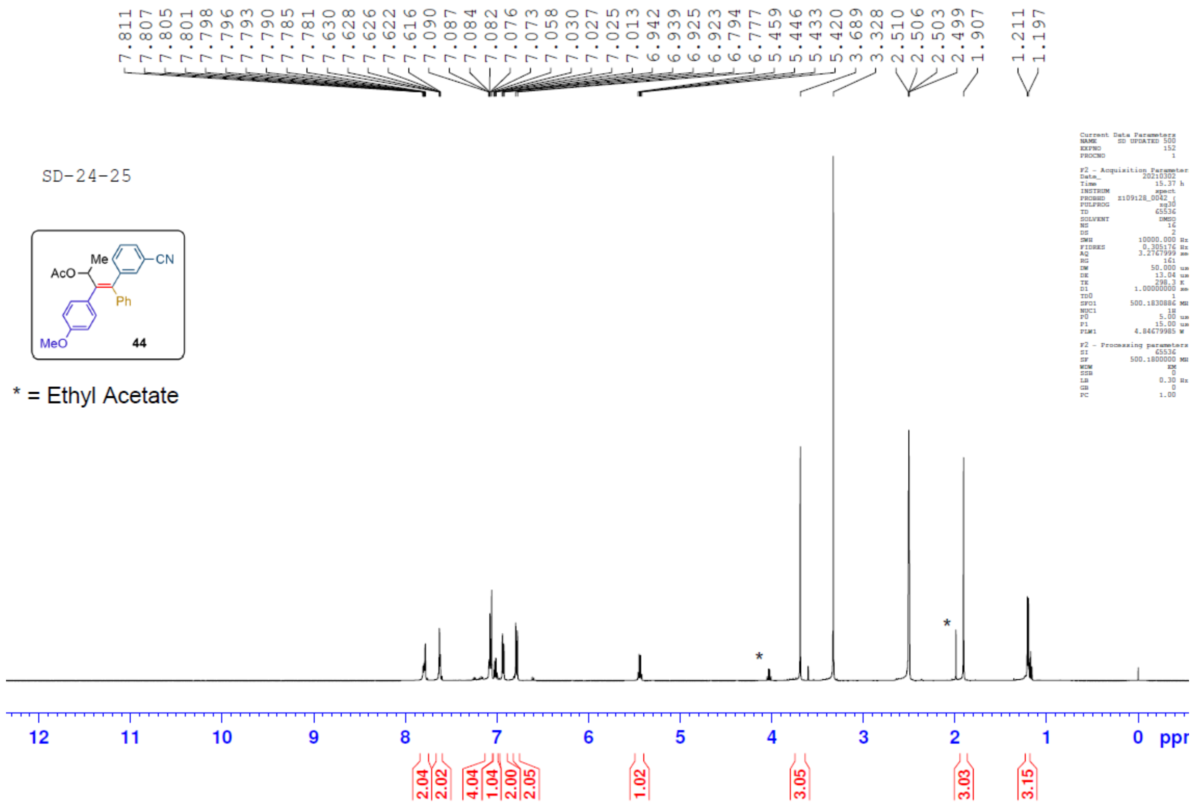

Supplementary Figure 133. ^{1}H NMR of compound **44**

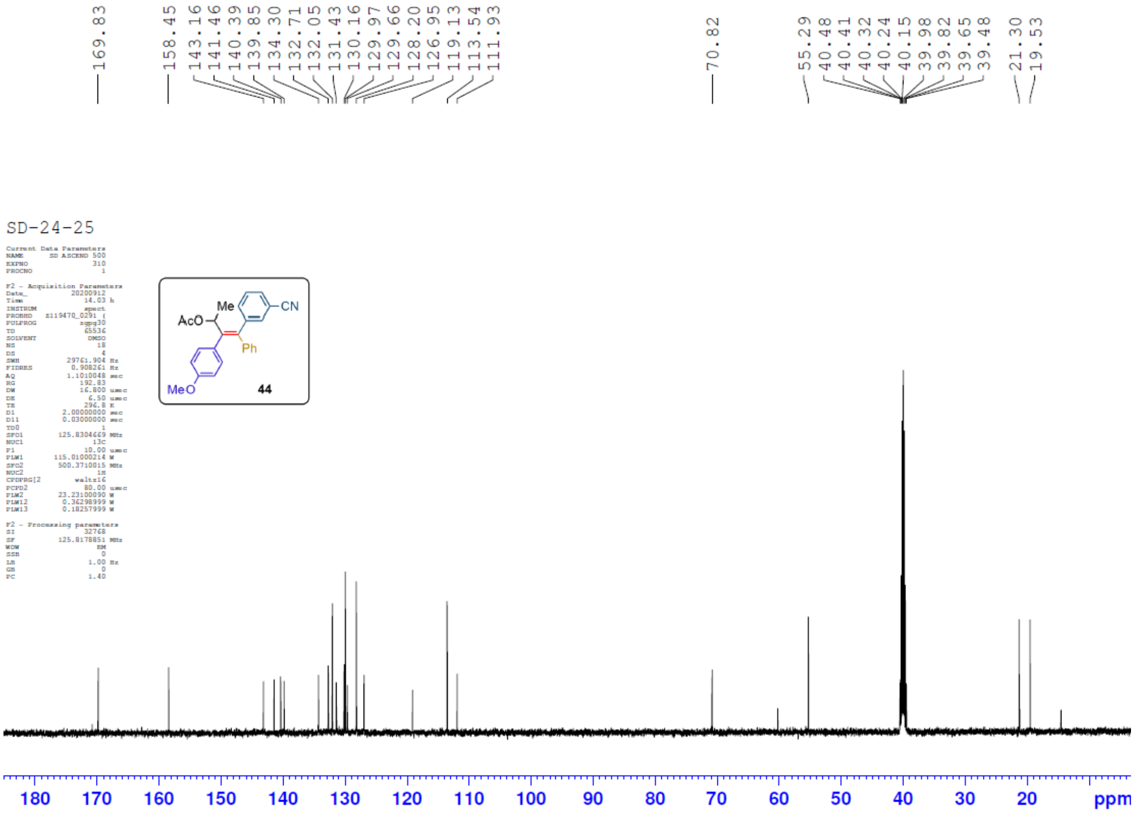

Supplementary Figure 134. ^{13}C NMR of compound **44**

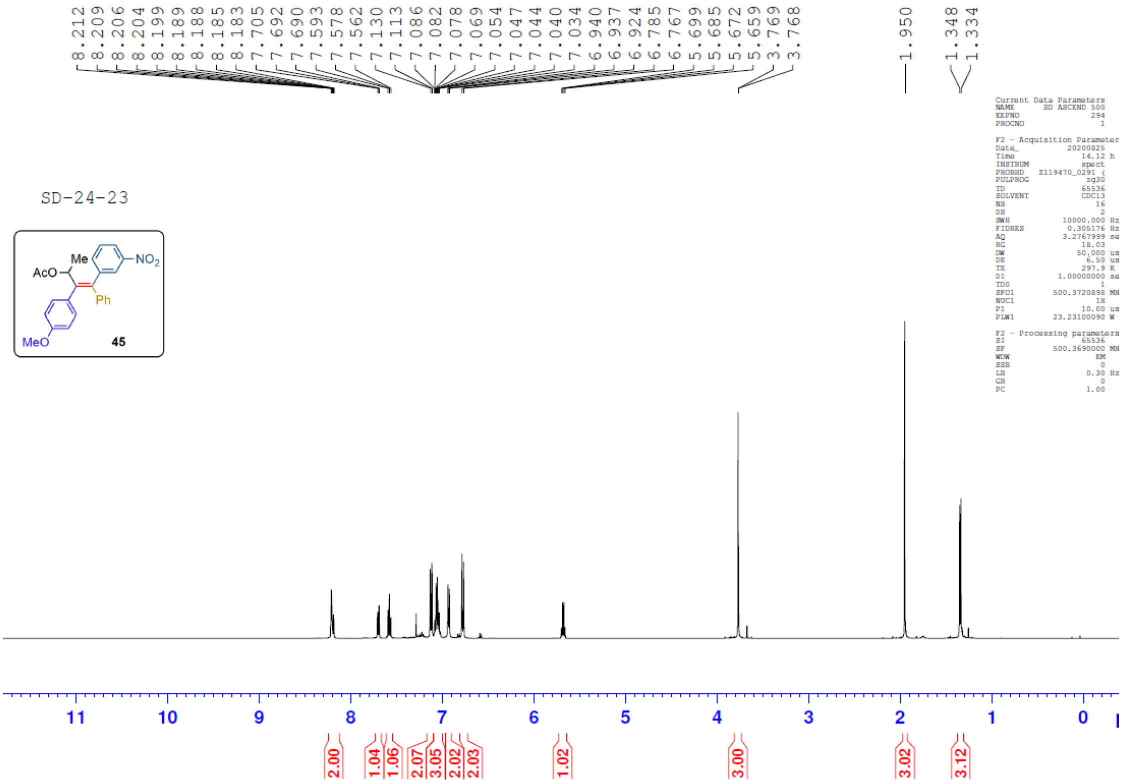

Supplementary Figure 135. ^{1}H NMR of compound **45**

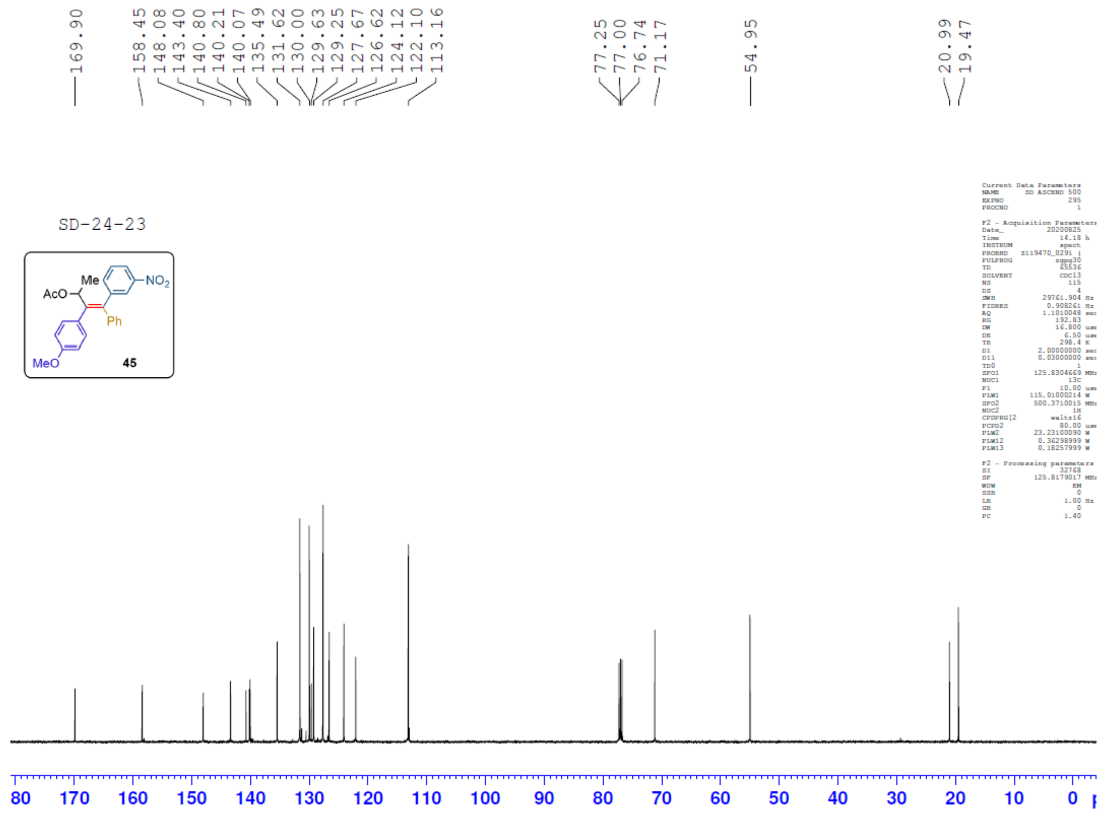

Supplementary Figure 136. ^{13}C NMR of compound **45**

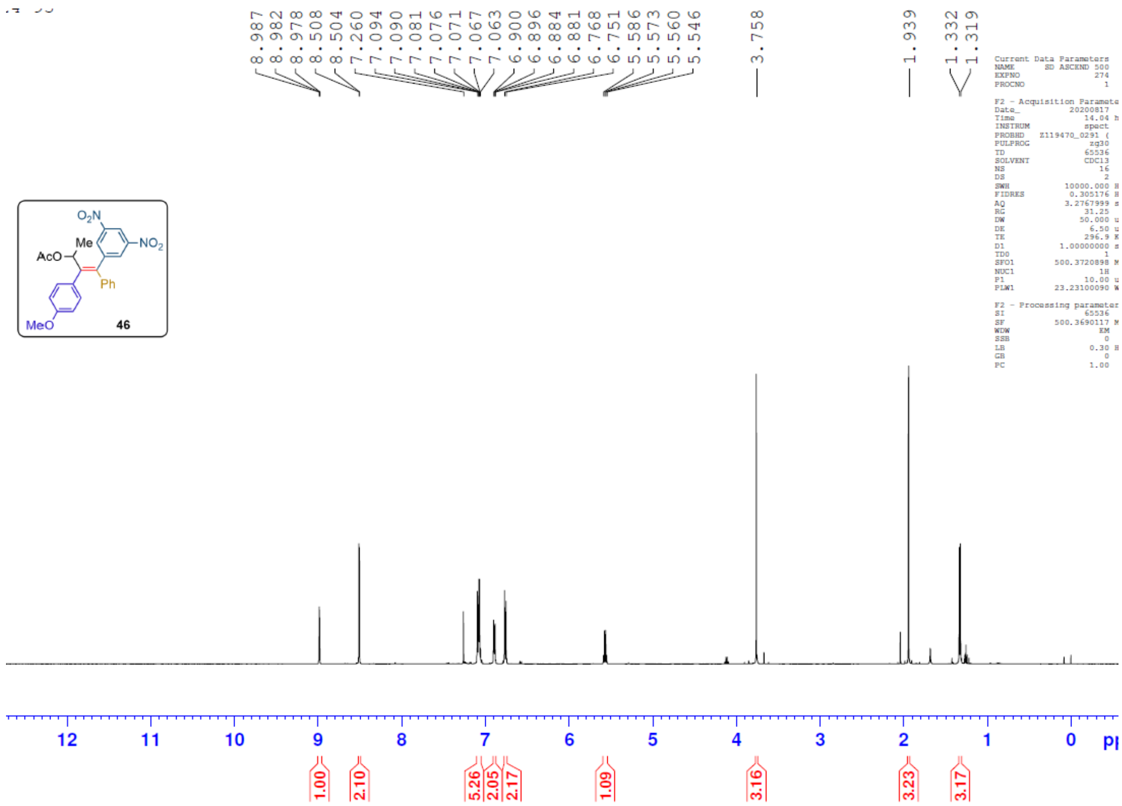

Supplementary Figure 137. ^{1}H NMR of compound **46**

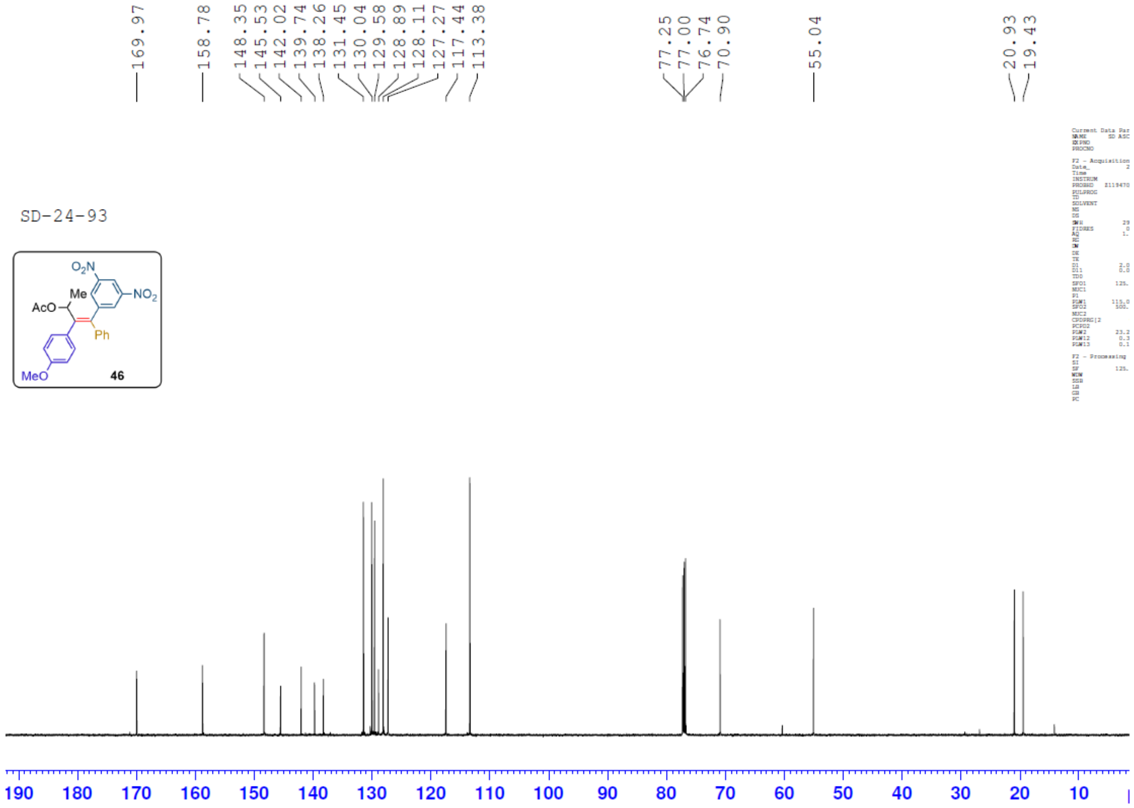

Supplementary Figure 138. ^{13}C NMR of compound **46**

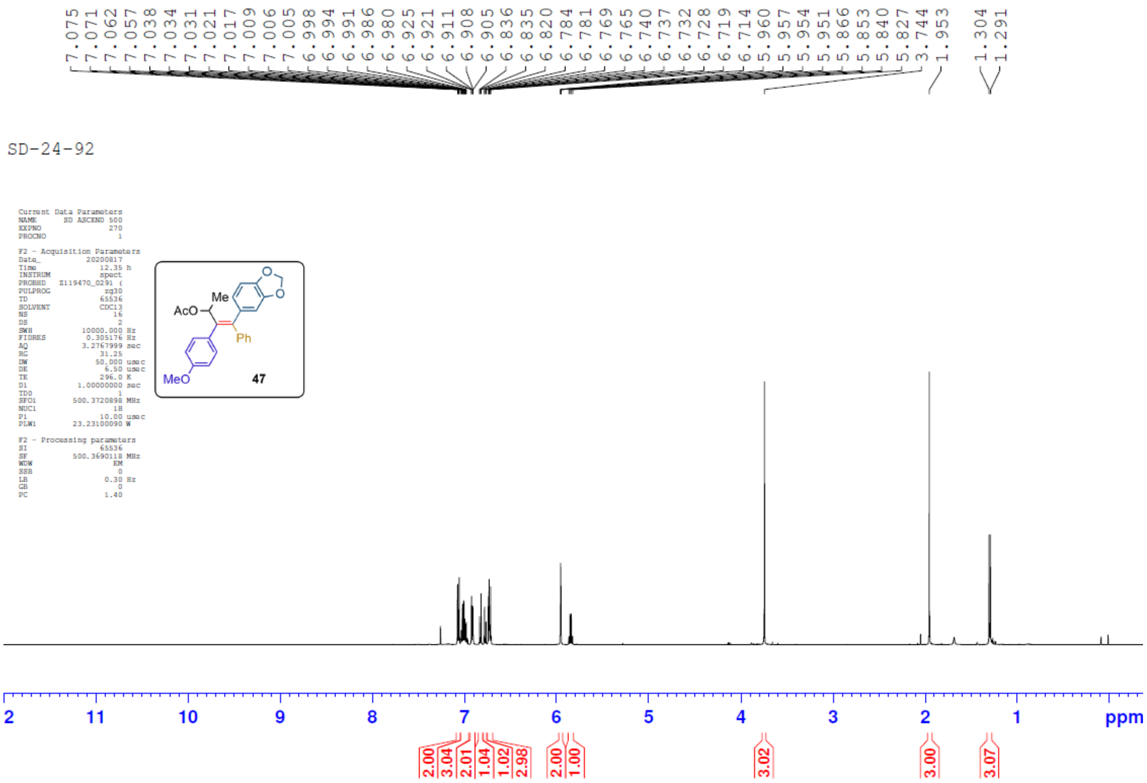

Supplementary Figure 139. ^{1}H NMR of compound **47**

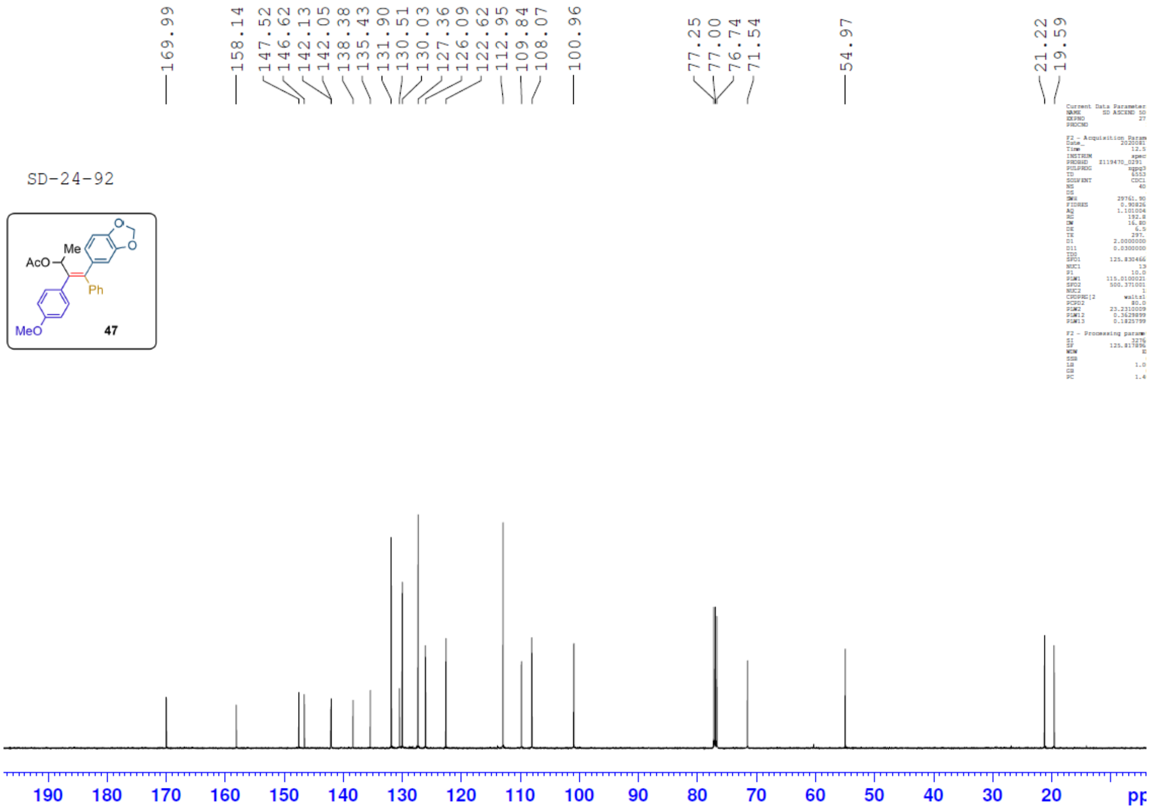

Supplementary Figure 140. ^{13}C NMR of compound **47**

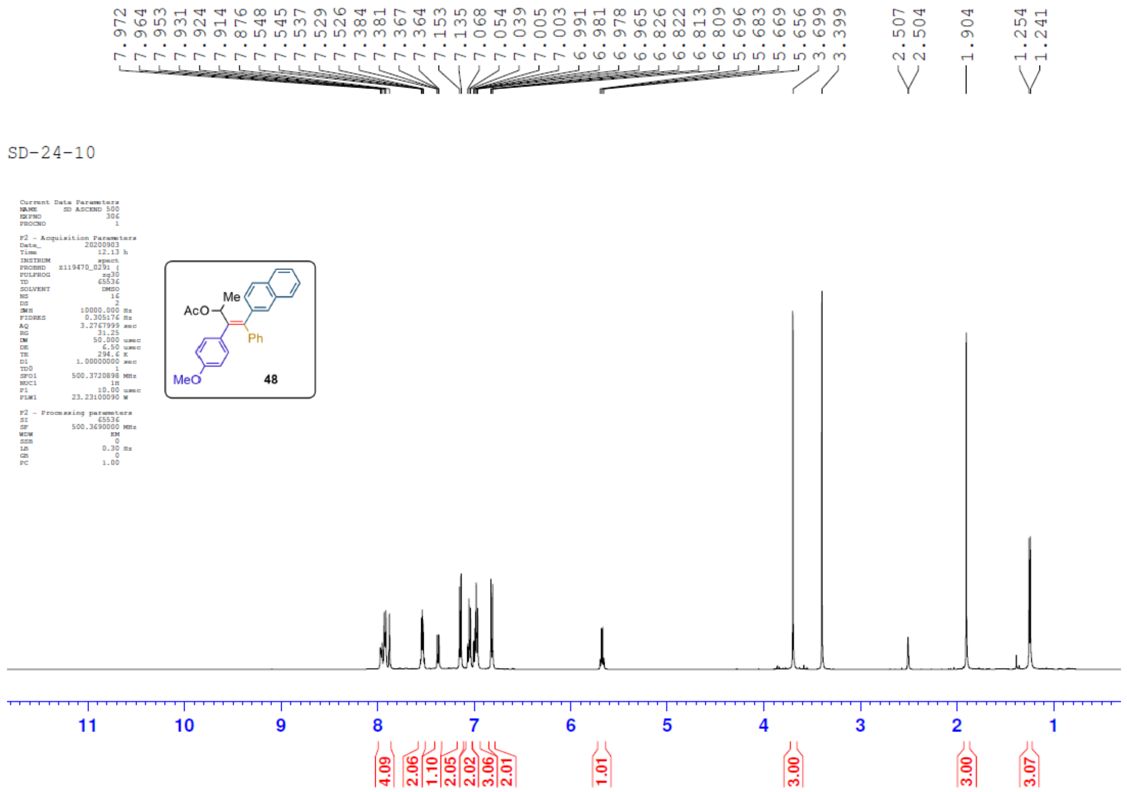

Supplementary Figure 141. ^{1}H NMR of compound **48**

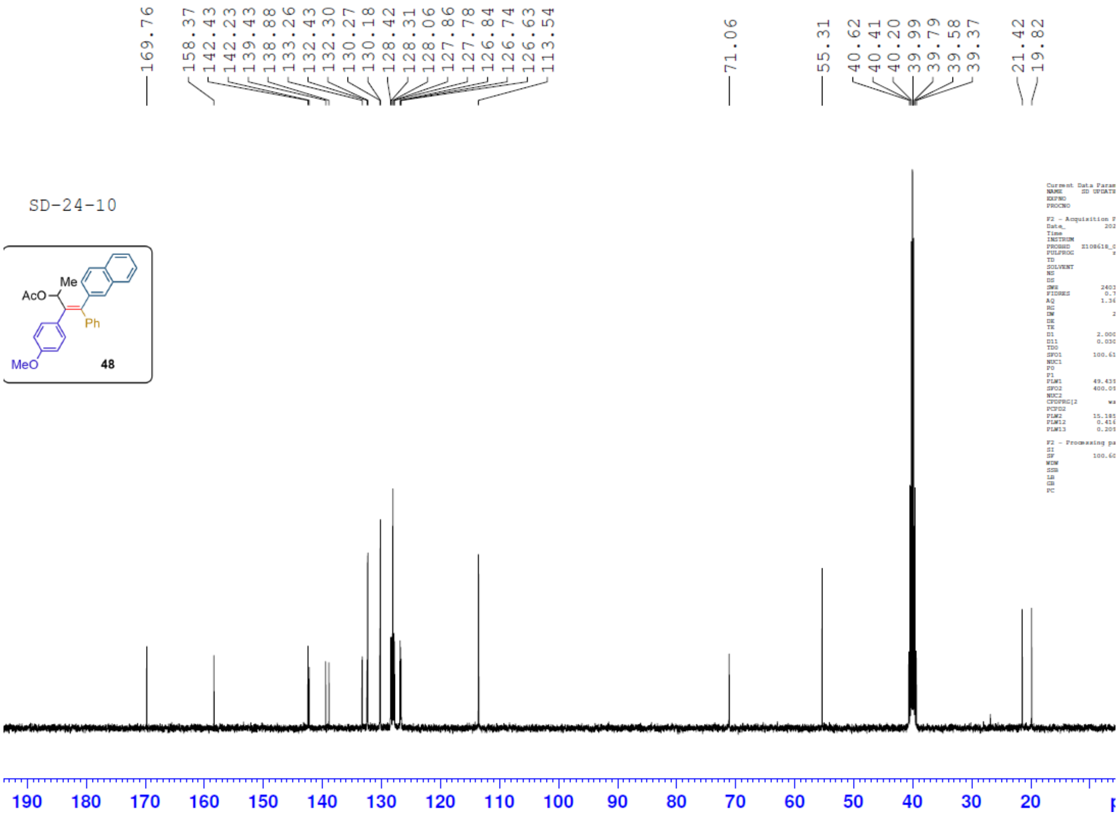

Supplementary Figure 142. ^{13}C NMR of compound **48**

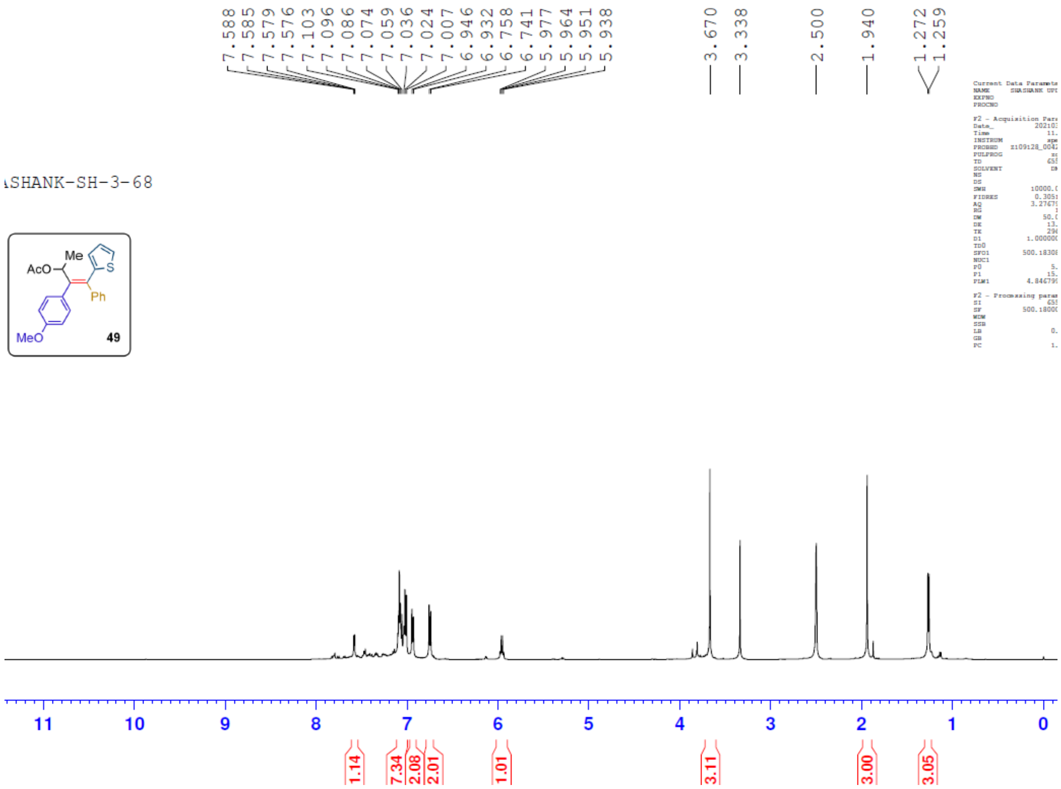

Supplementary Figure 143. ^{1}H NMR of compound **49**

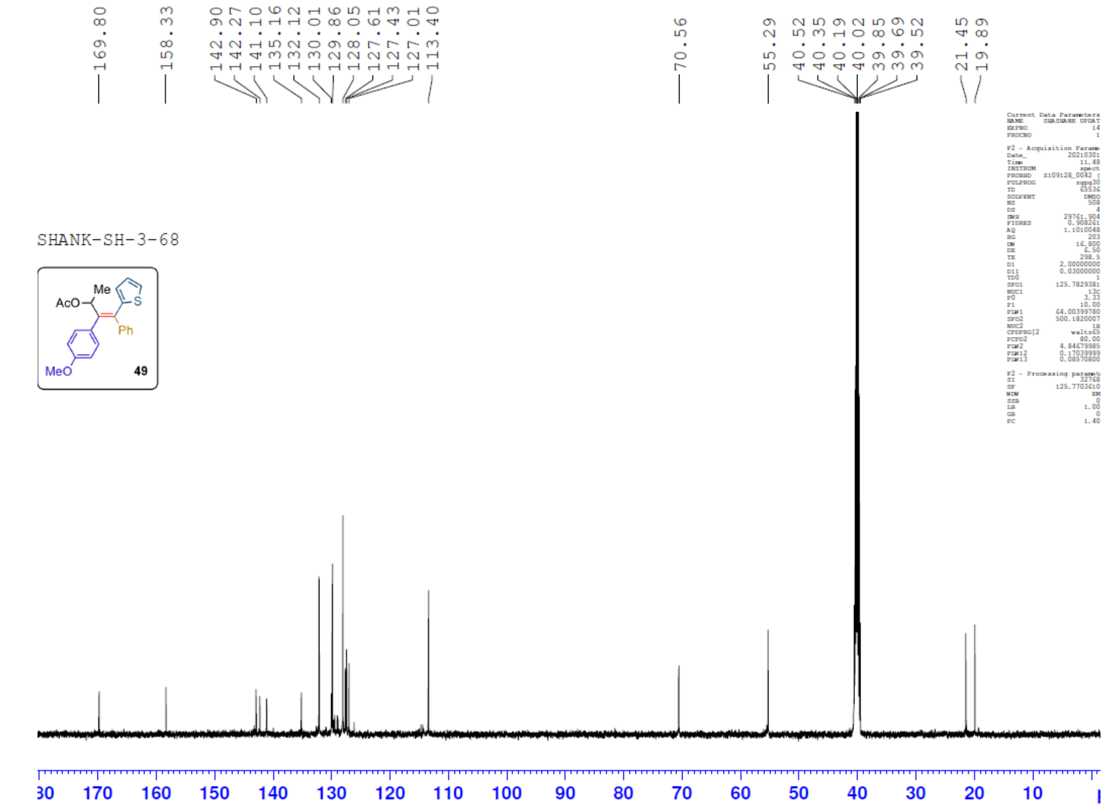

Supplementary Figure 144. ^{13}C NMR of compound **49**

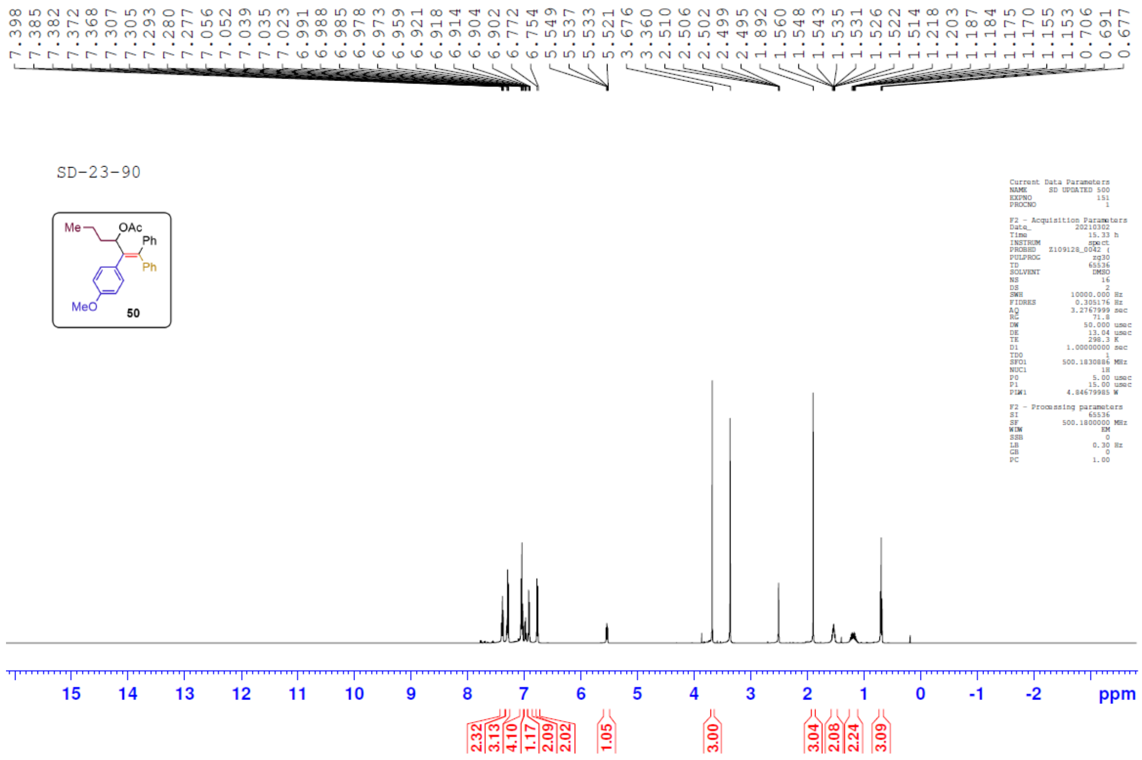

Supplementary Figure 145. ^{1}H NMR of compound **50**

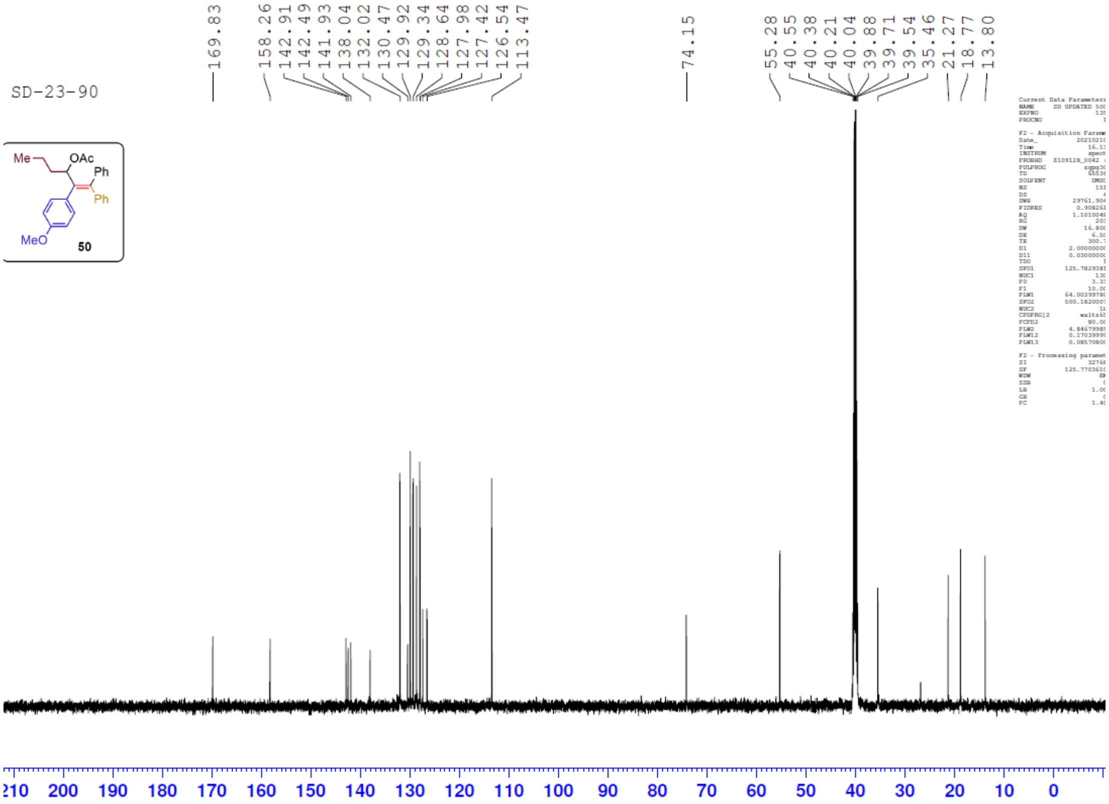

Supplementary Figure 146. ^{13}C NMR of compound **50**

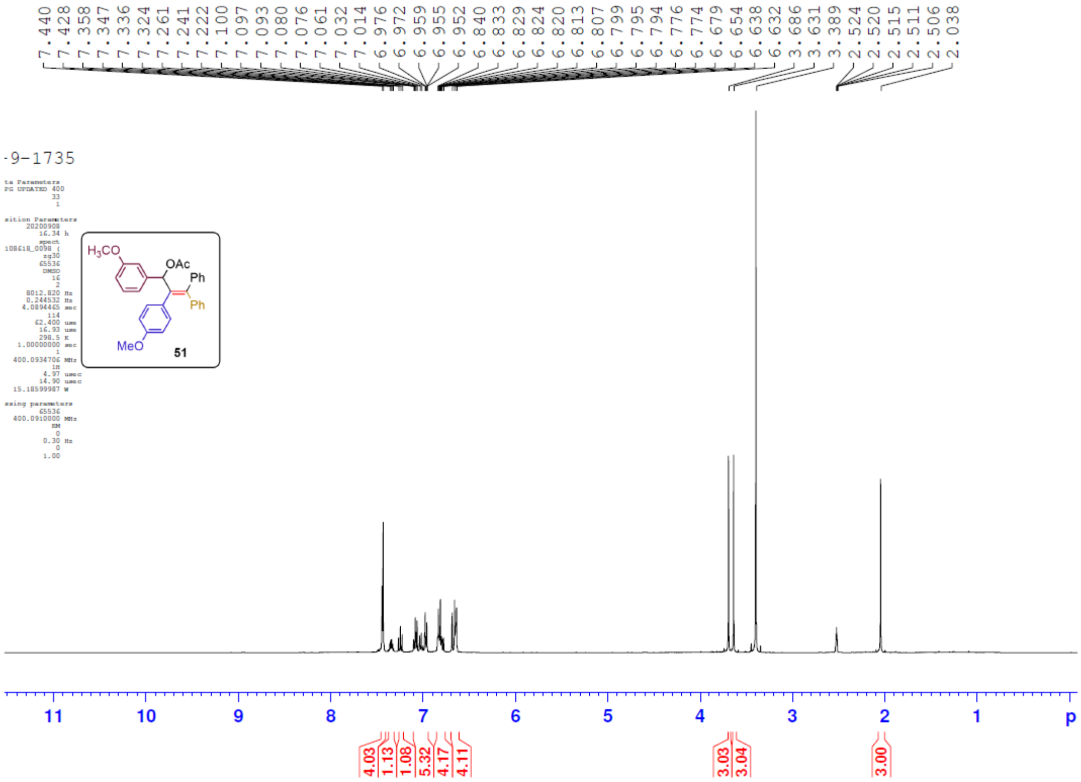

Supplementary Figure 147. ^{1}H NMR of compound **51**

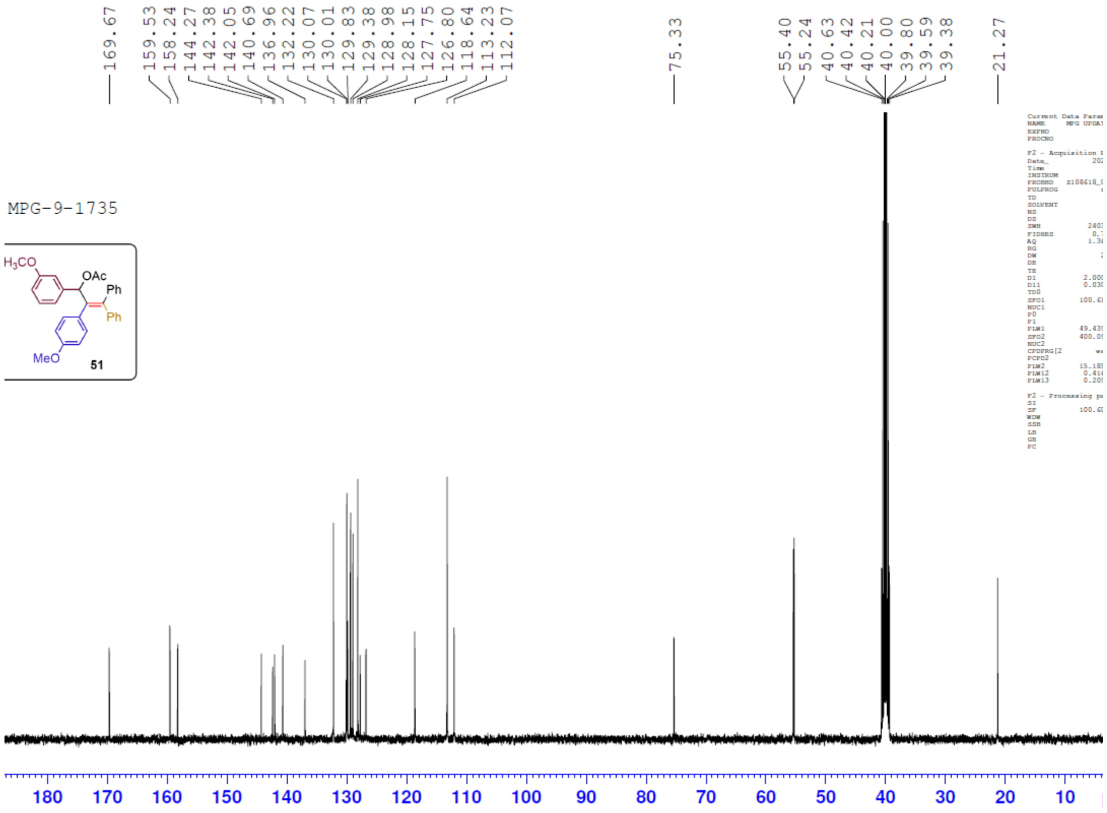

Supplementary Figure 148. ^{13}C NMR of compound **51**

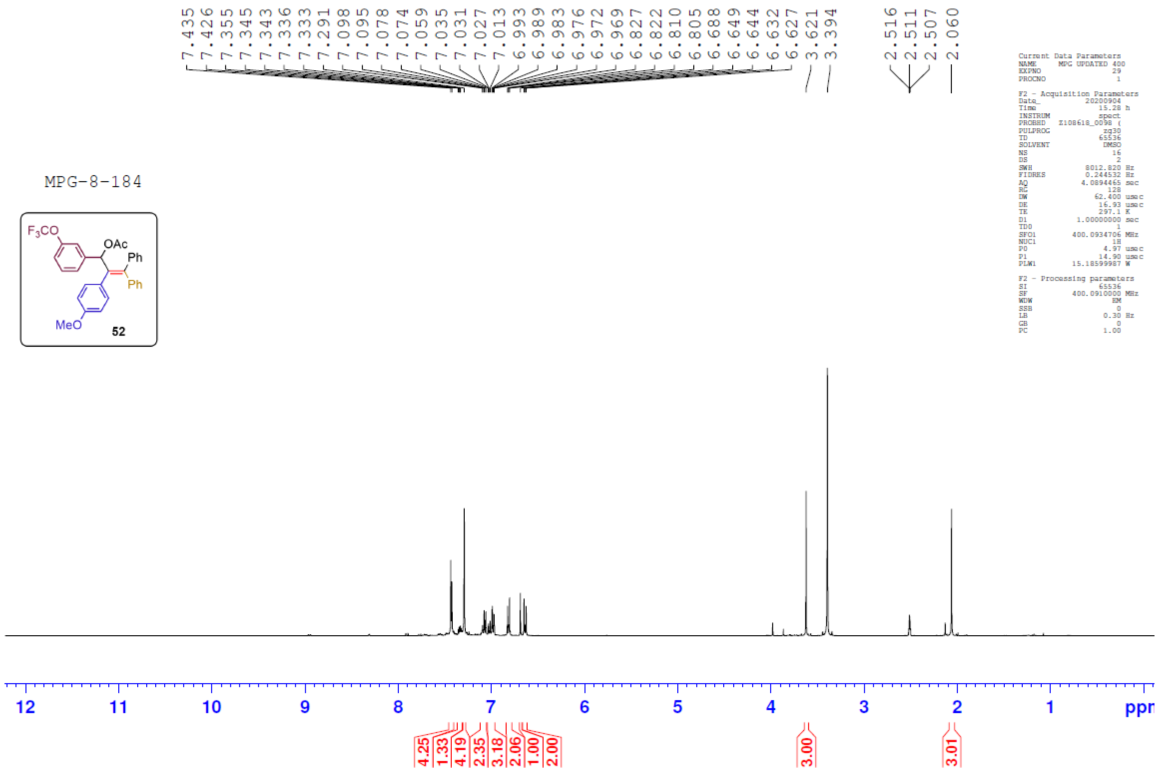

Supplementary Figure 149. ^{1}H NMR of compound **52**

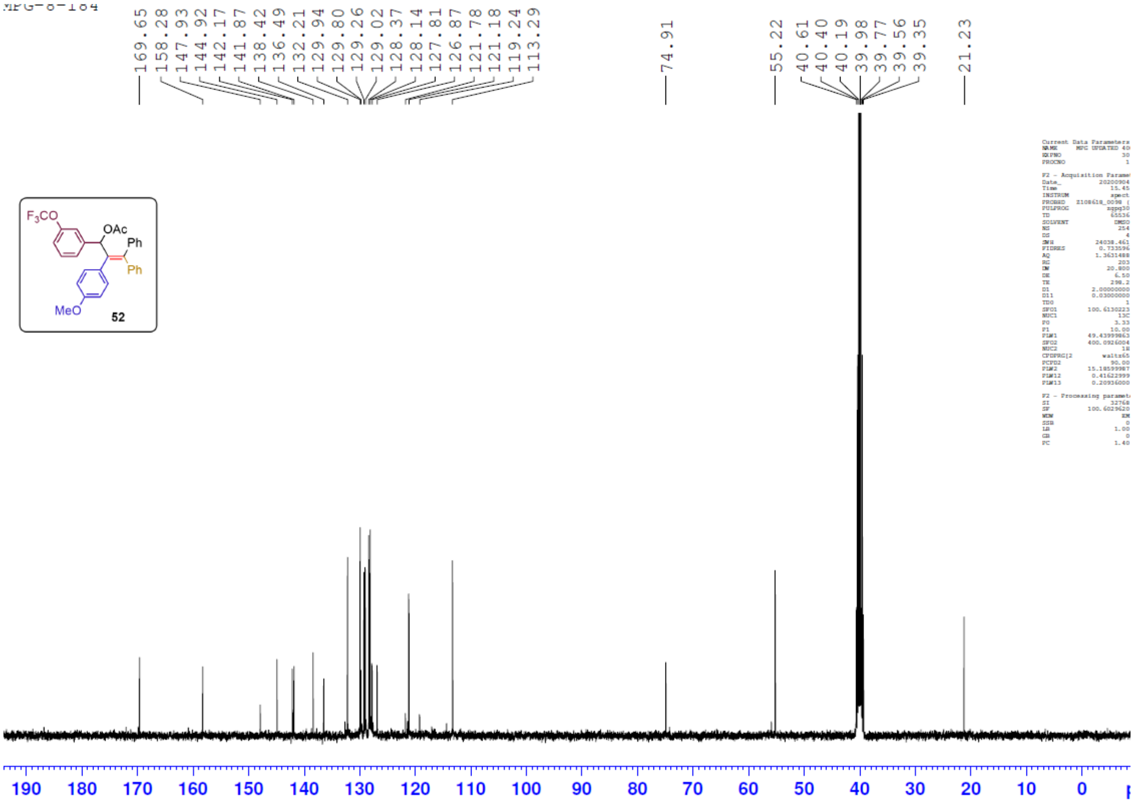

Supplementary Figure 150. ^{13}C NMR of compound **52**

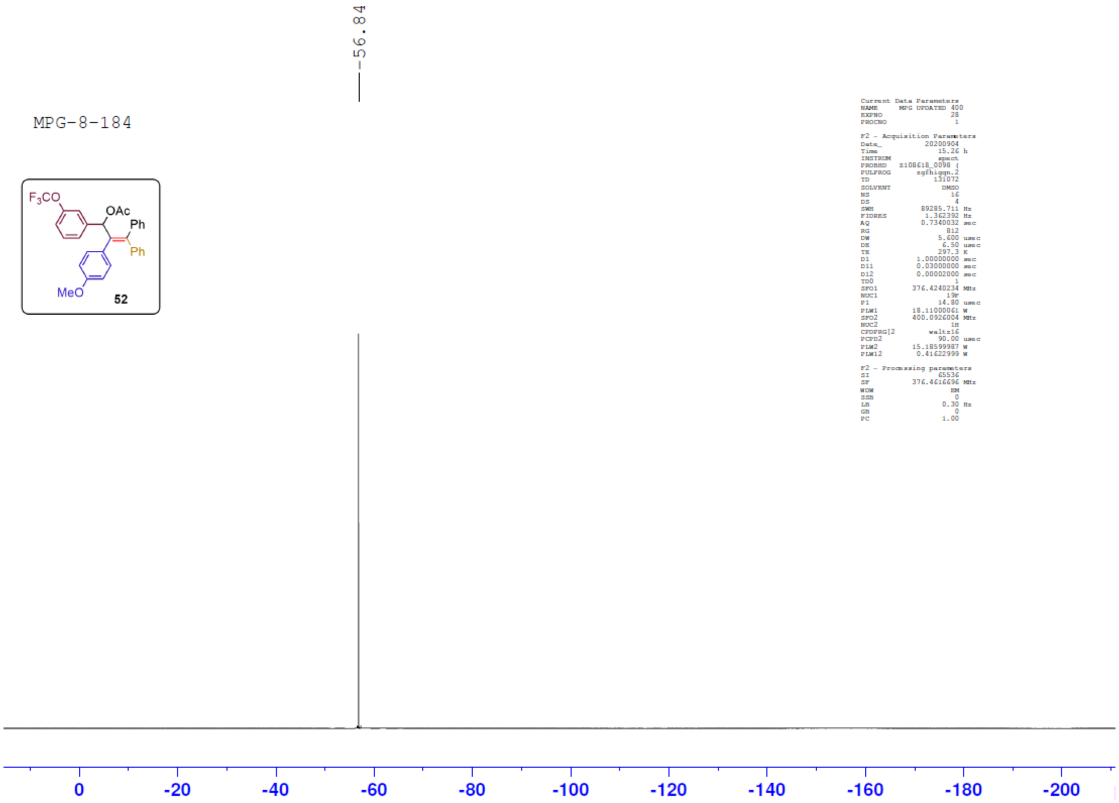

Supplementary Figure 151. ^{19}F NMR of compound **52**

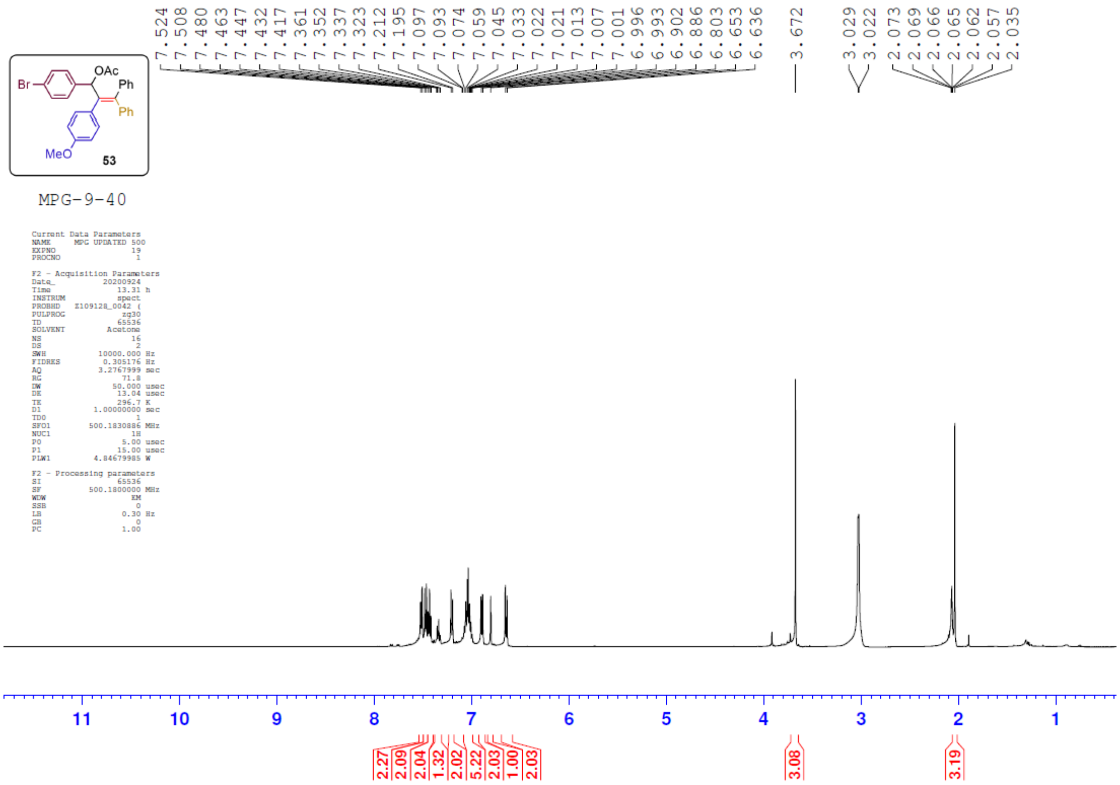

Supplementary Figure 152. ^{1}H NMR of compound **53**

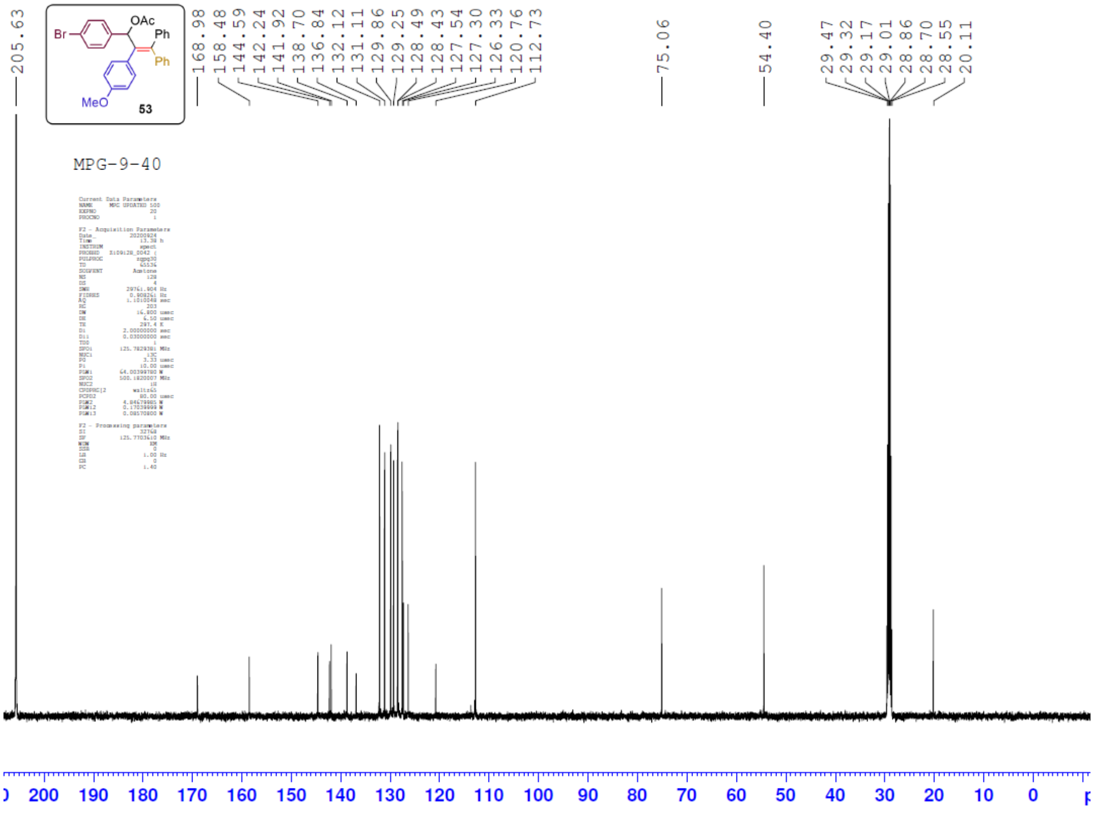

Supplementary Figure 153. ^{13}C NMR of compound **53**

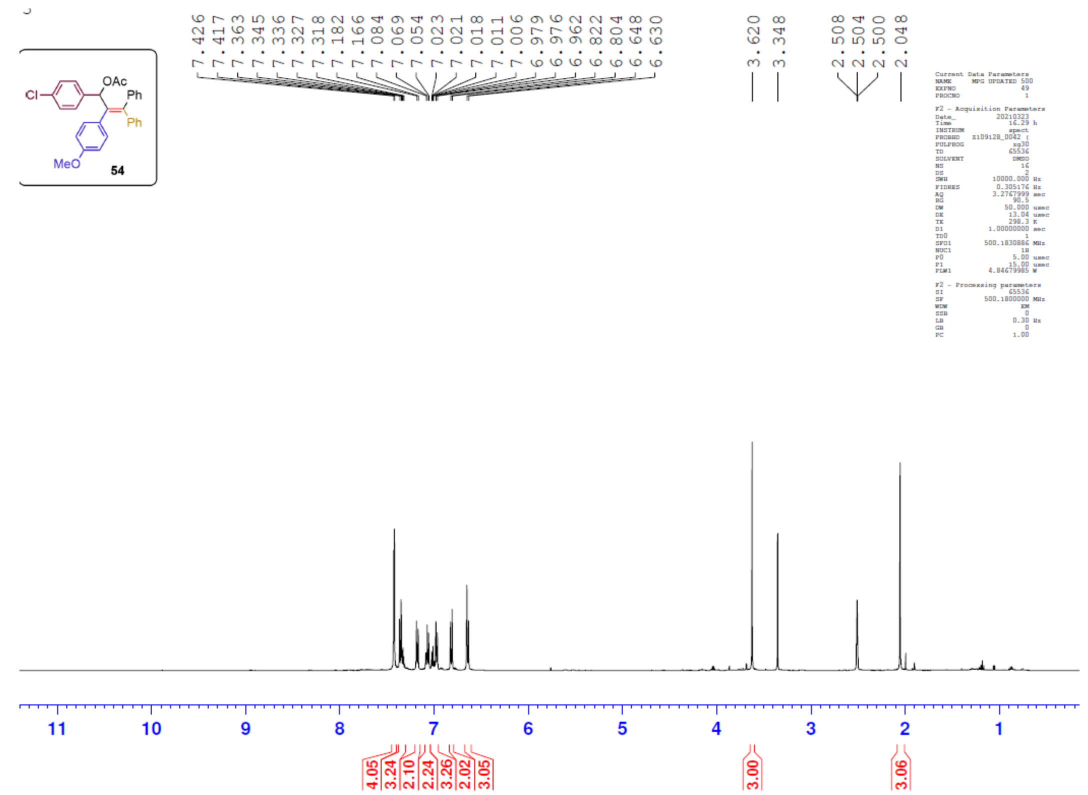

Supplementary Figure 154. ^{1}H NMR of compound **54**

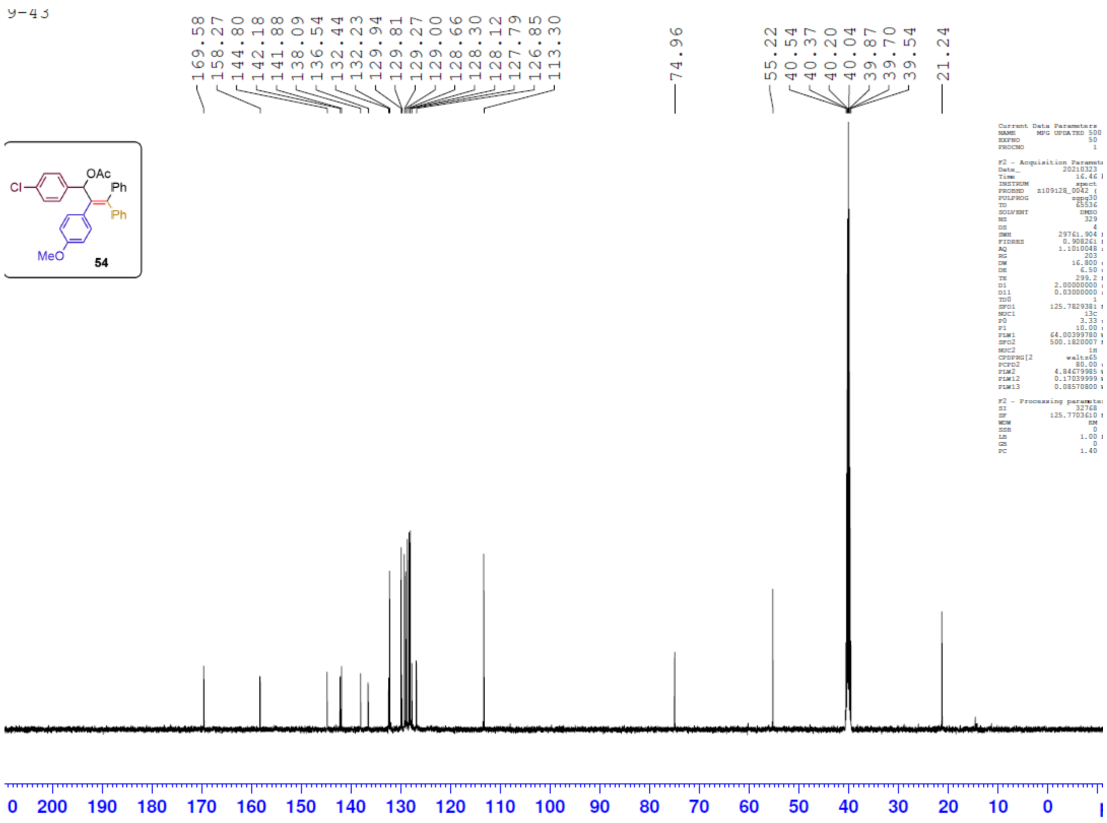

Supplementary Figure 155. ^{13}C NMR of compound **54**

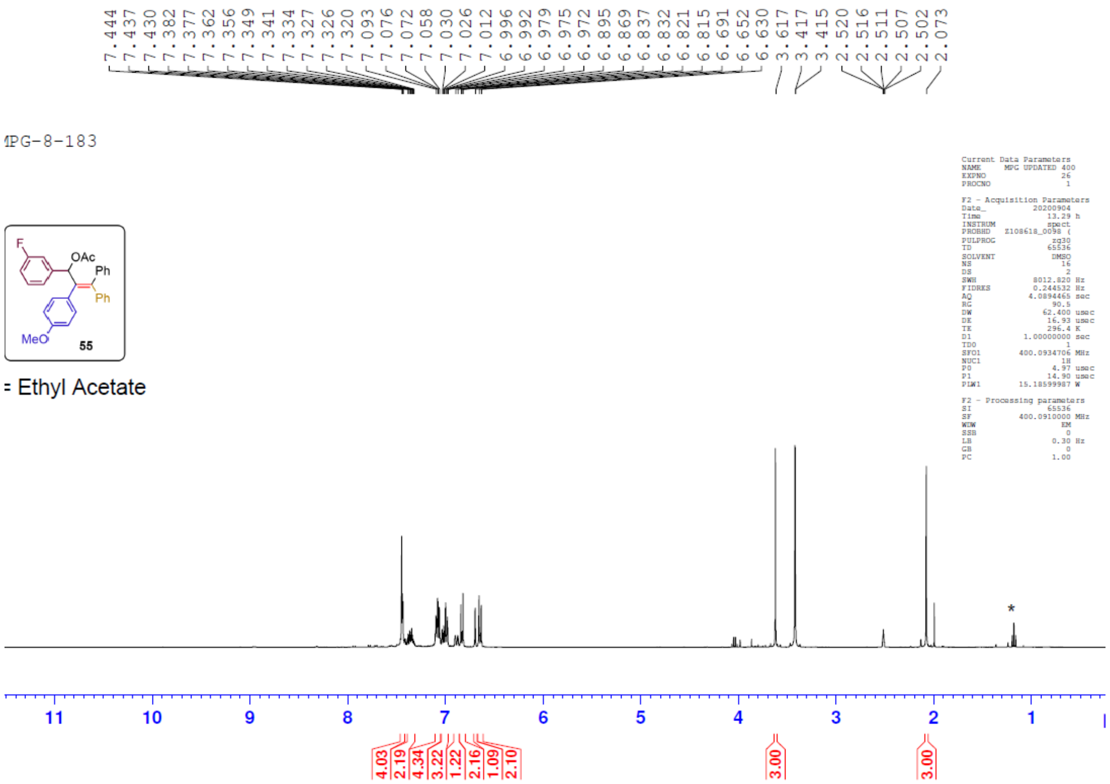

Supplementary Figure 156. ^{1}H NMR of compound **55**

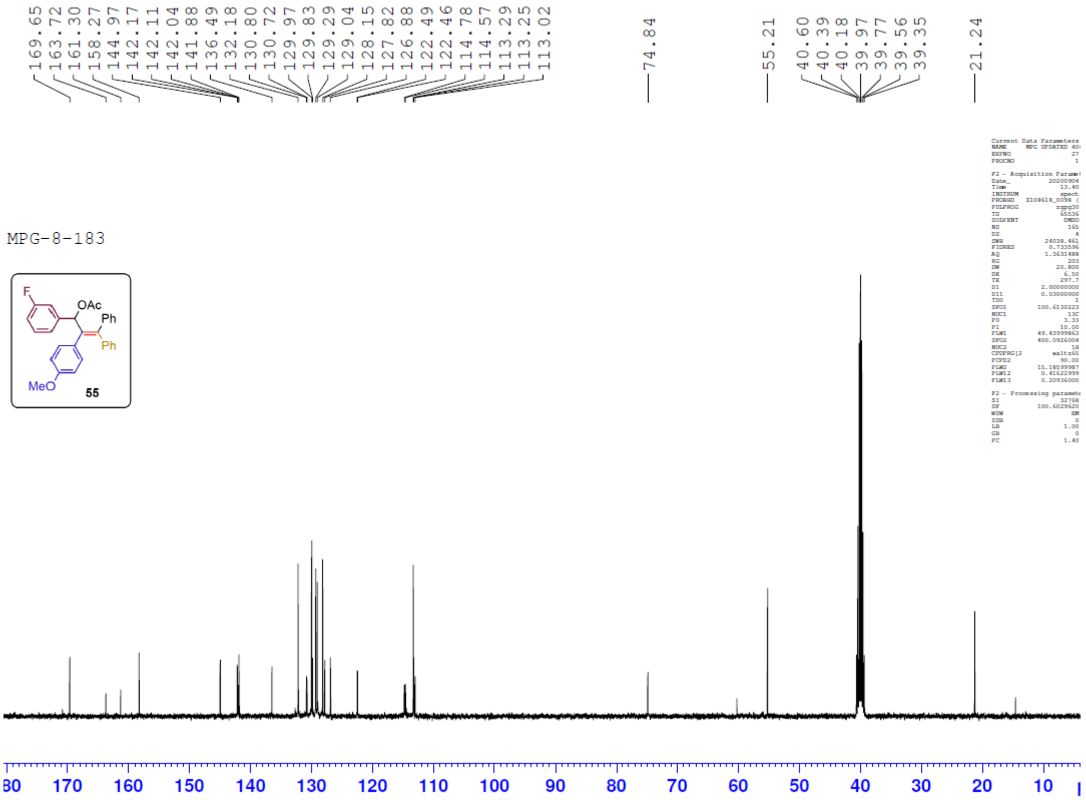

Supplementary Figure 157. ^{1}H NMR of compound **55**

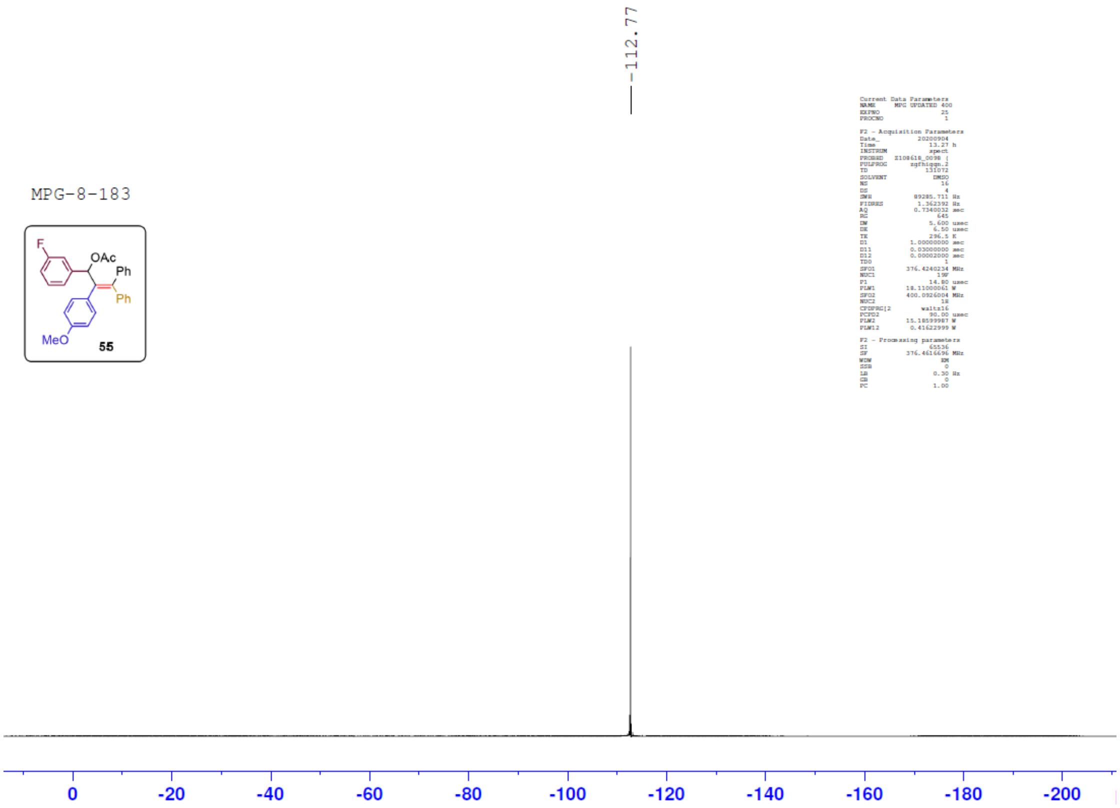

Supplementary Figure 158. ^{19}F NMR of compound **55**

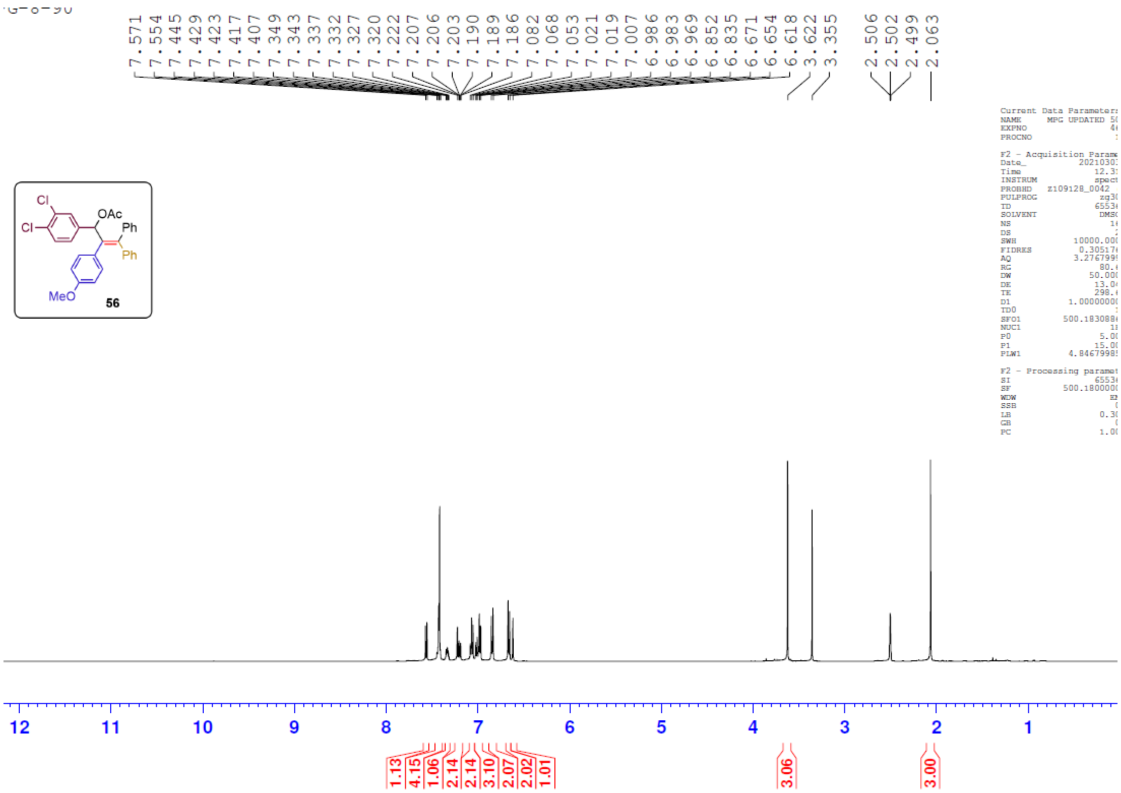

Supplementary Figure 159. ^{1}H NMR of compound **56**

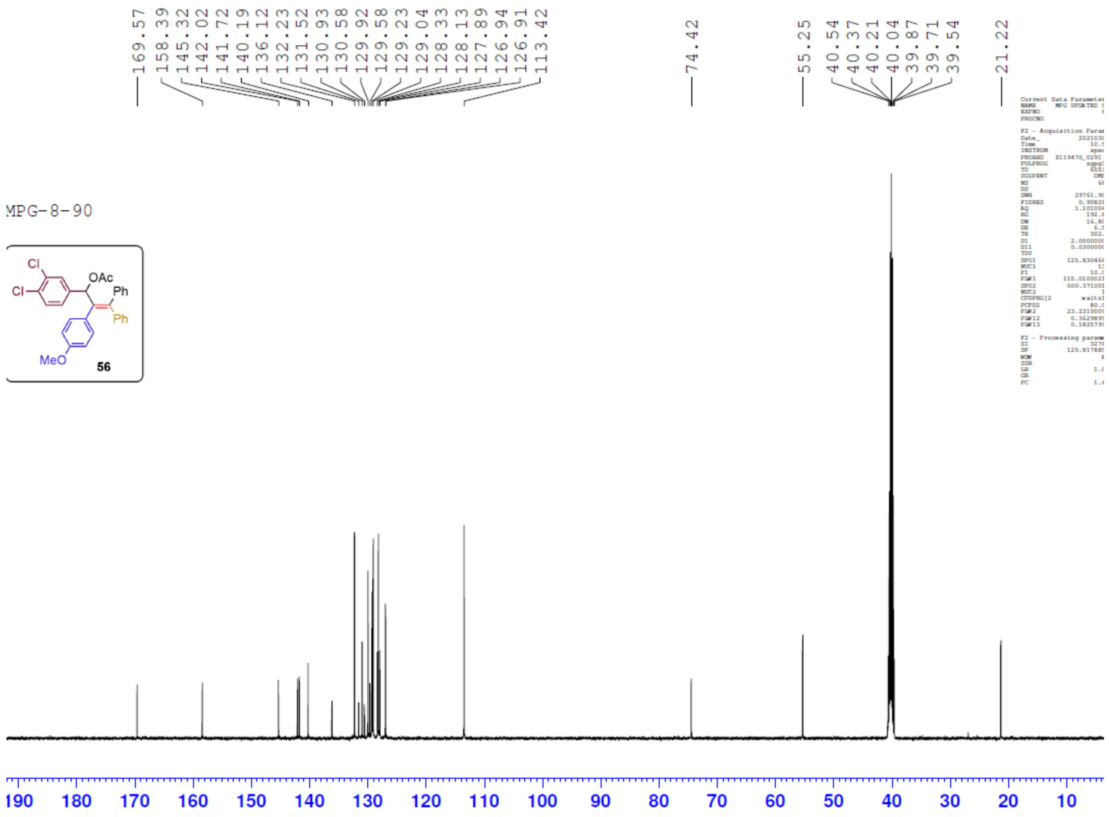

Supplementary Figure 160. ^{13}C NMR of compound **56**

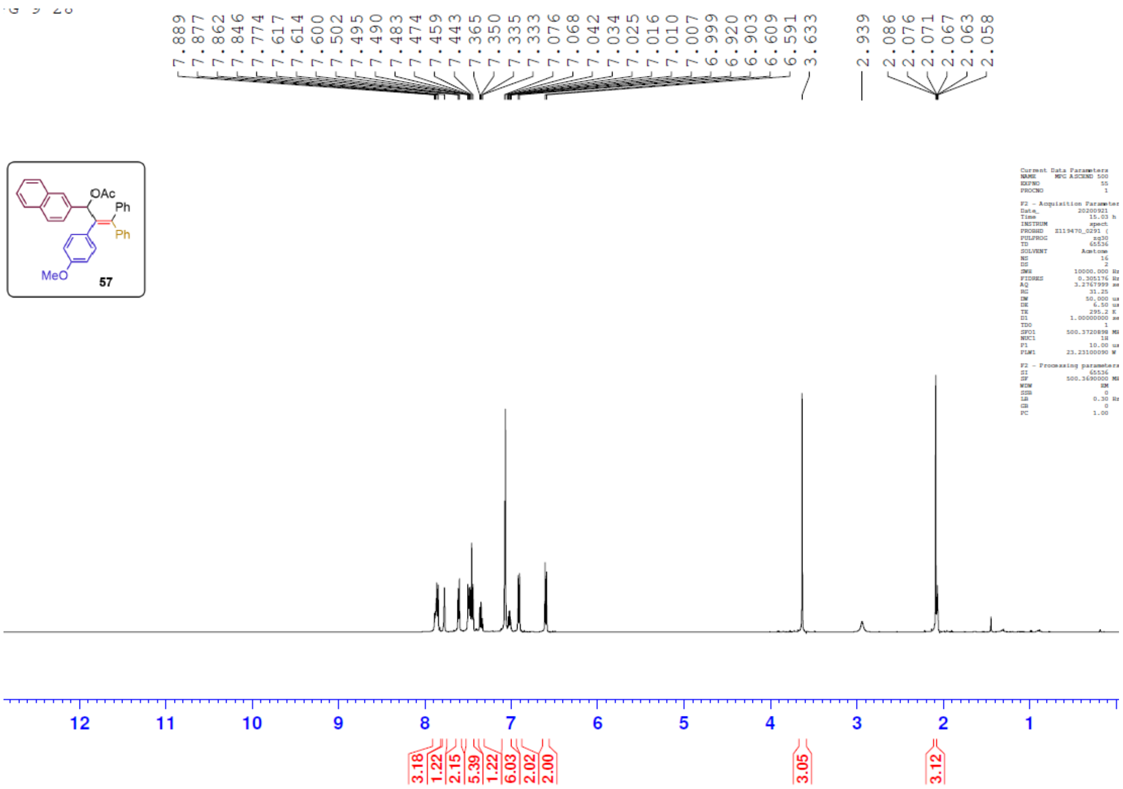

Supplementary Figure 161. ^{1}H NMR of compound **57**

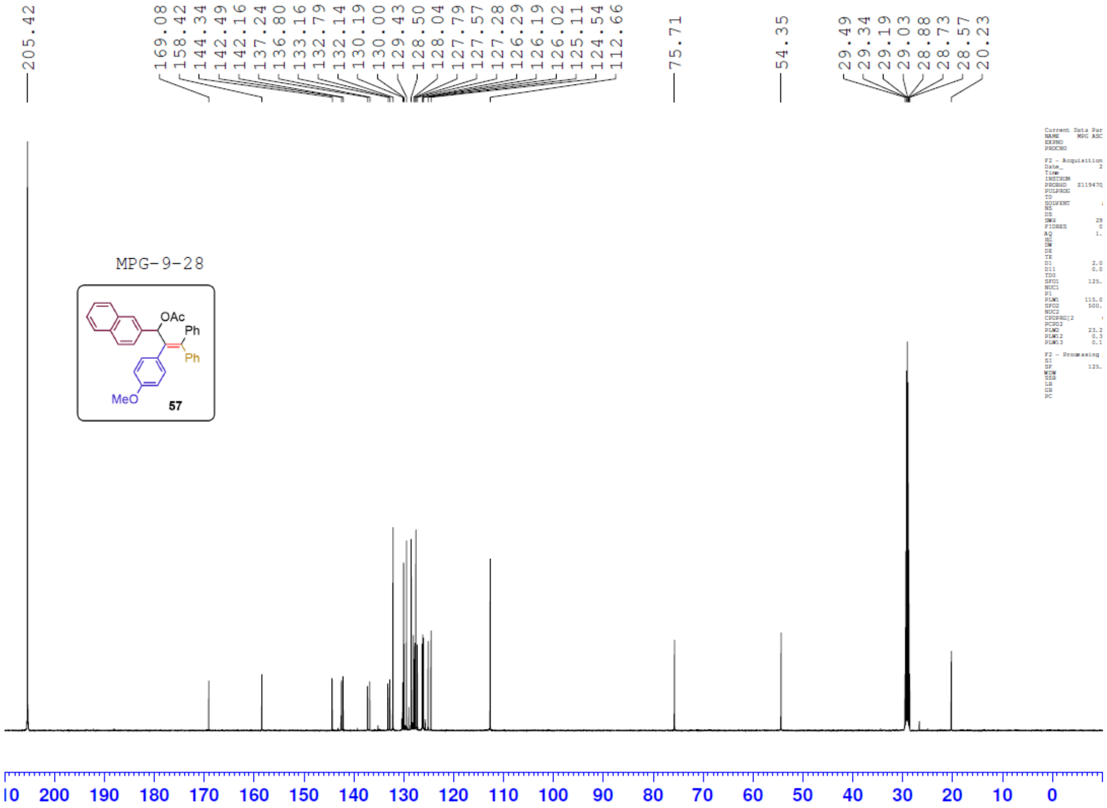

Supplementary Figure 162. ^{13}C NMR of compound **57**

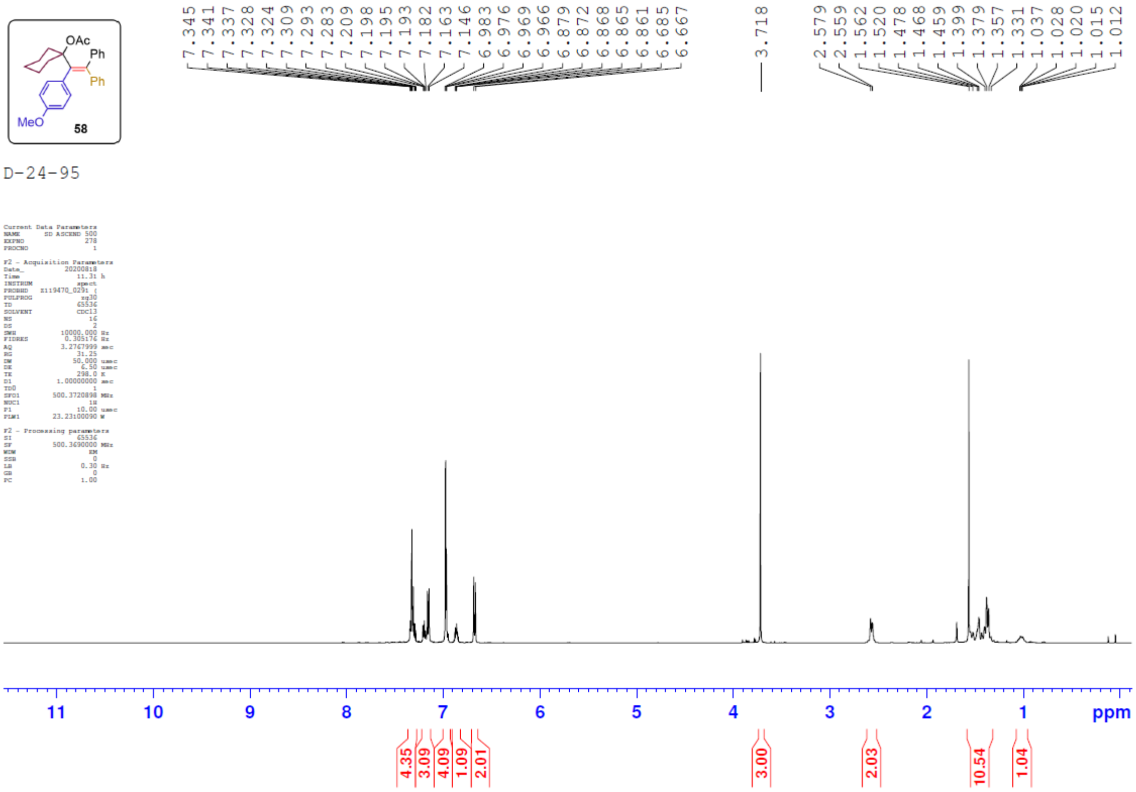

Supplementary Figure 163. ^{1}H NMR of compound **58**

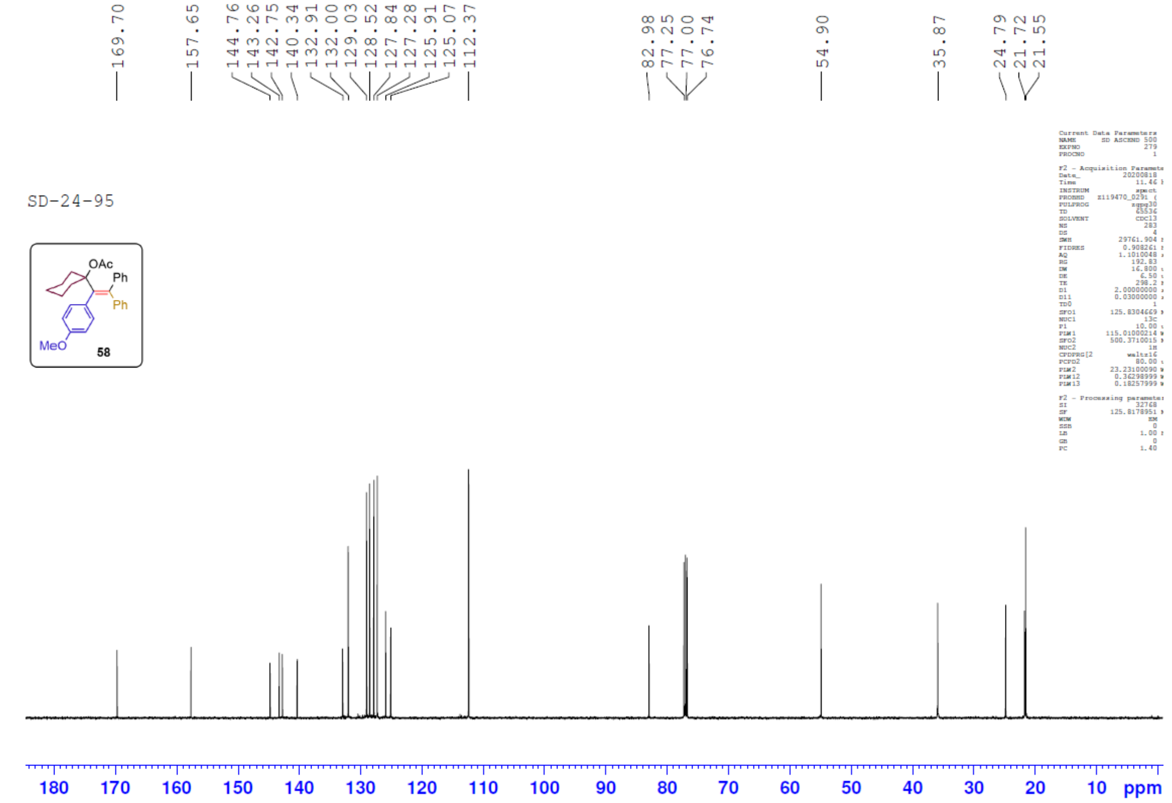

Supplementary Figure 164. ^{13}C NMR of compound **58**

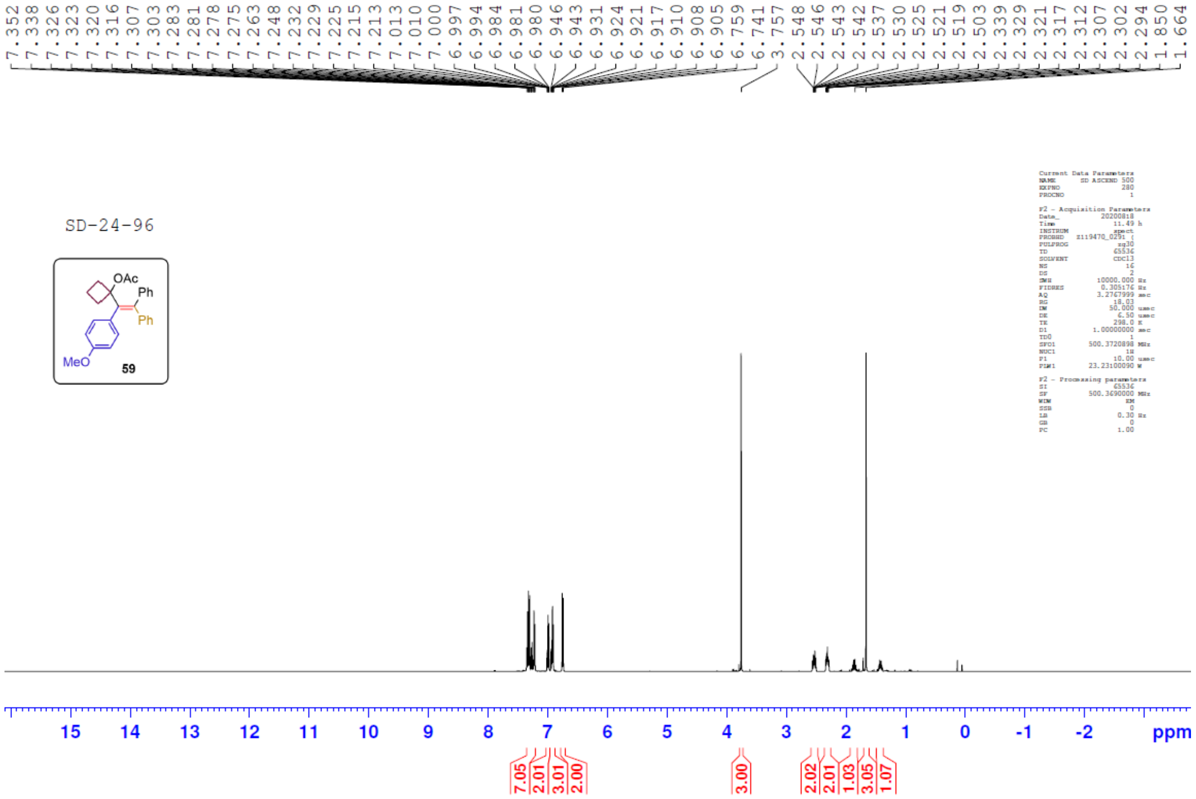

Supplementary Figure 165. ^{1}H NMR of compound **59**

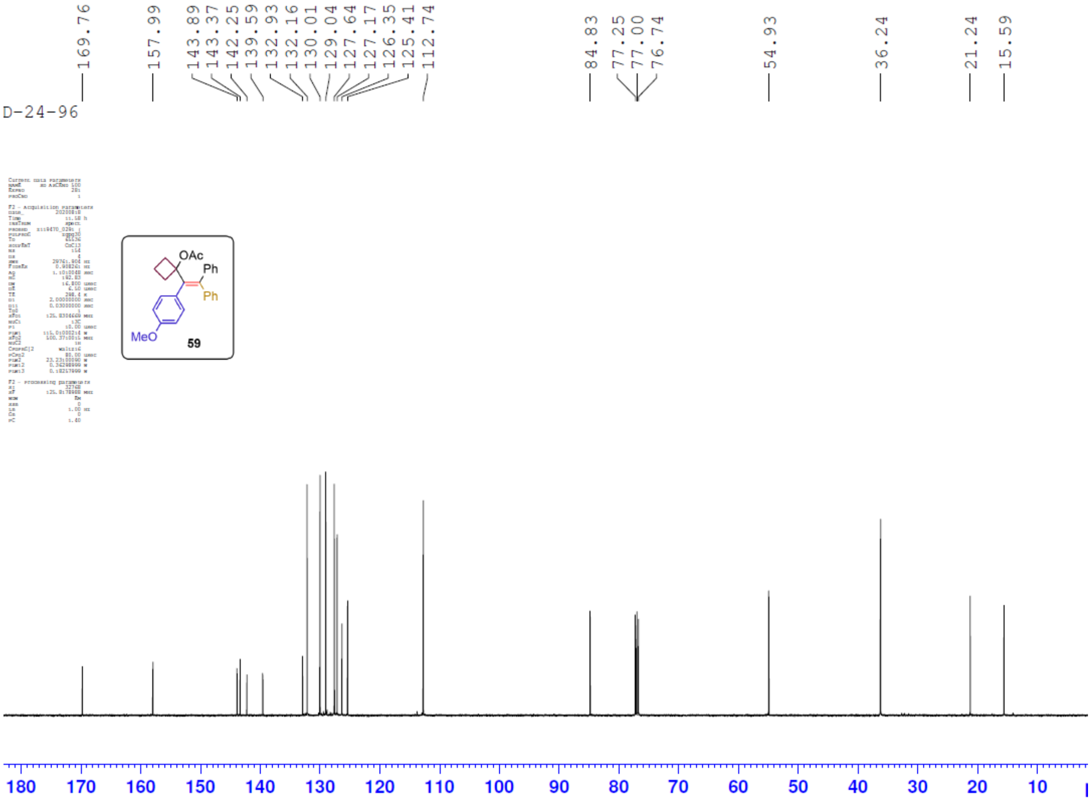

Supplementary Figure 166. ^{13}C NMR of compound **59**

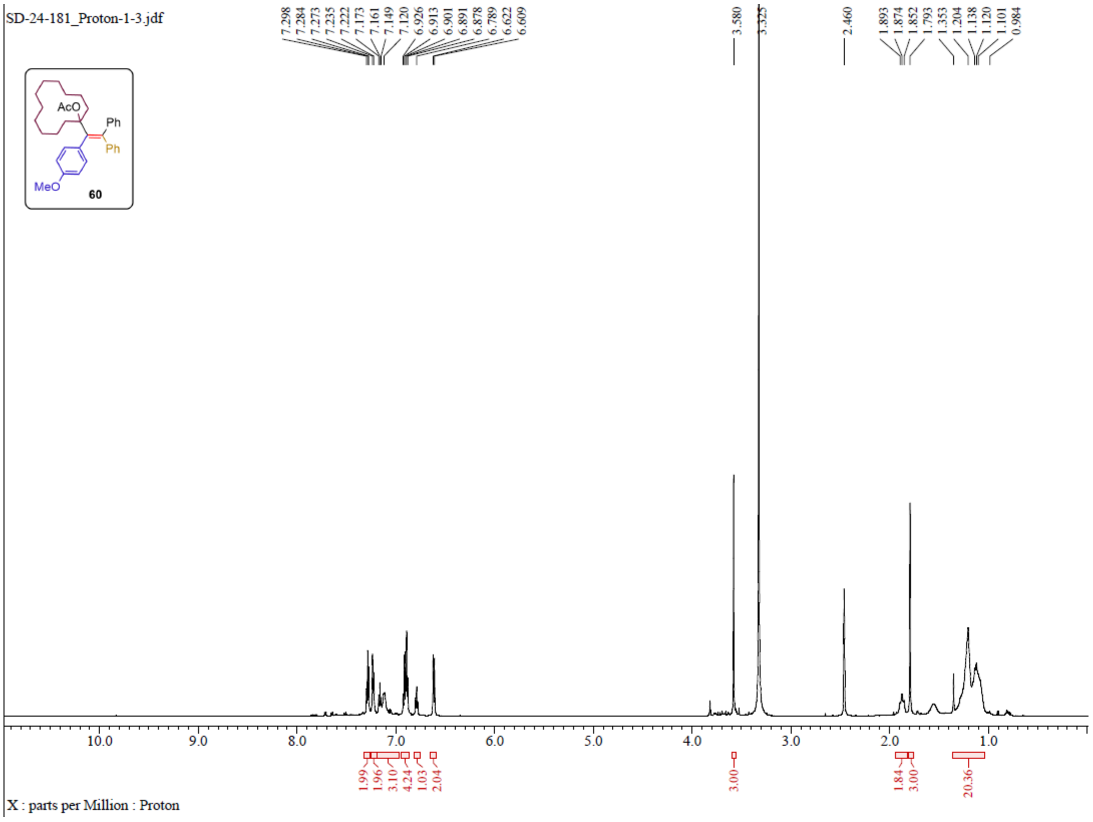

Supplementary Figure 167. ^{1}H NMR of compound **60**

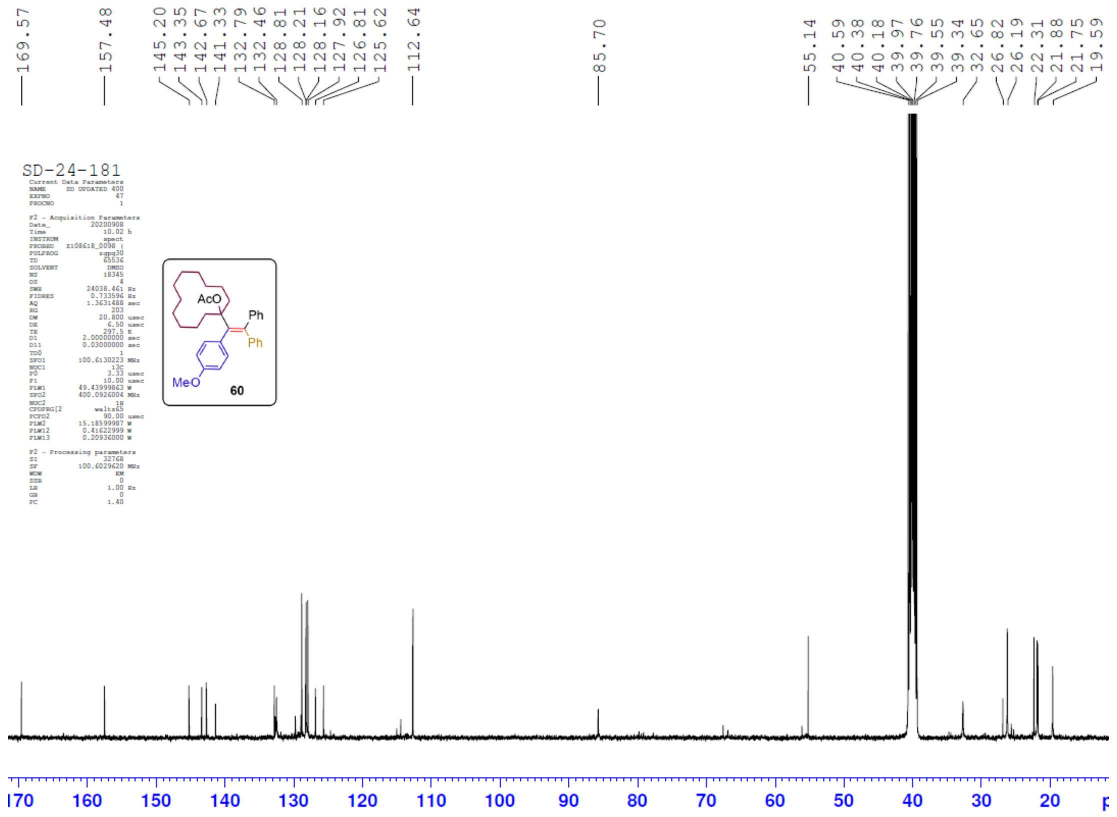

Supplementary Figure 168. ^{13}C NMR of compound **60**

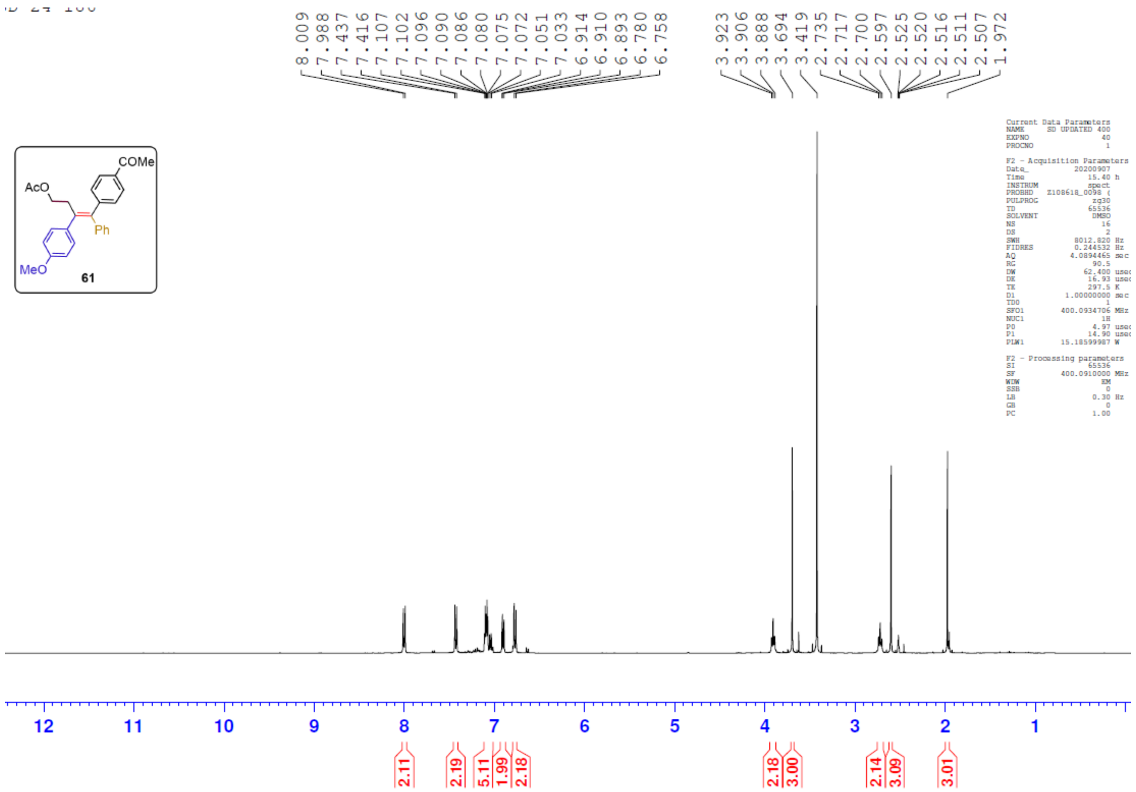

Supplementary Figure 169. ^{1}H NMR of compound **61**

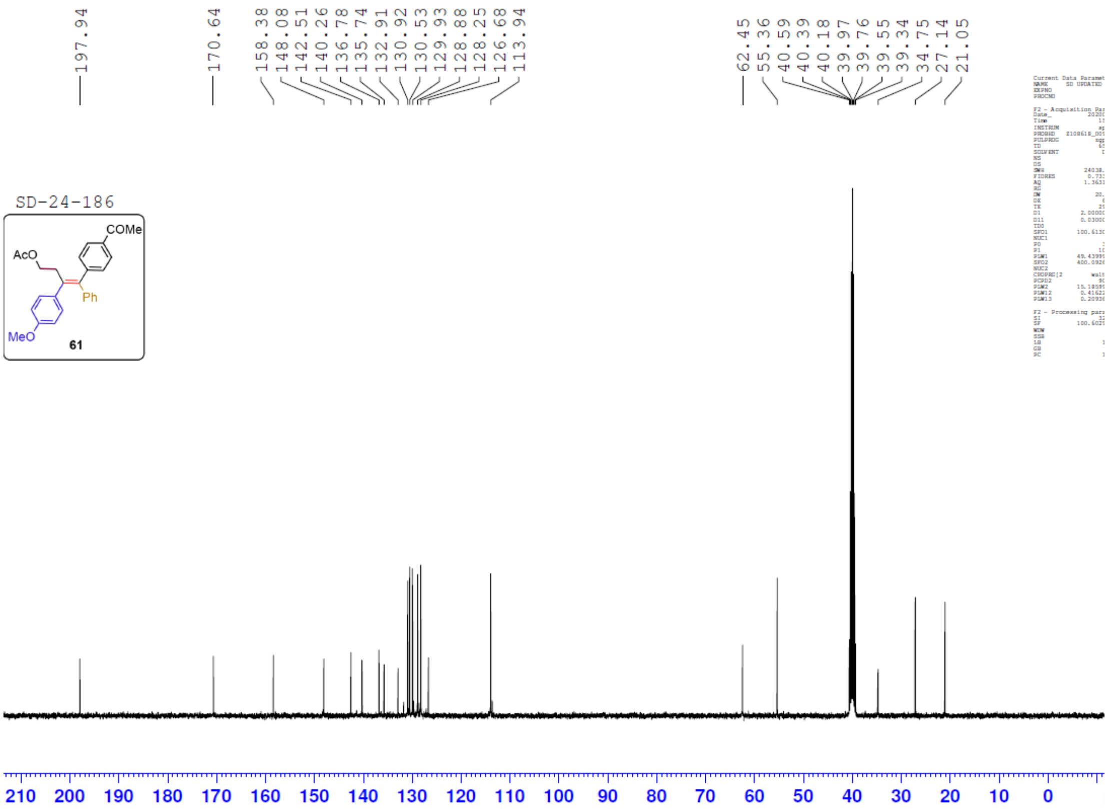

Supplementary Figure 170. ^{13}C NMR of compound **61**

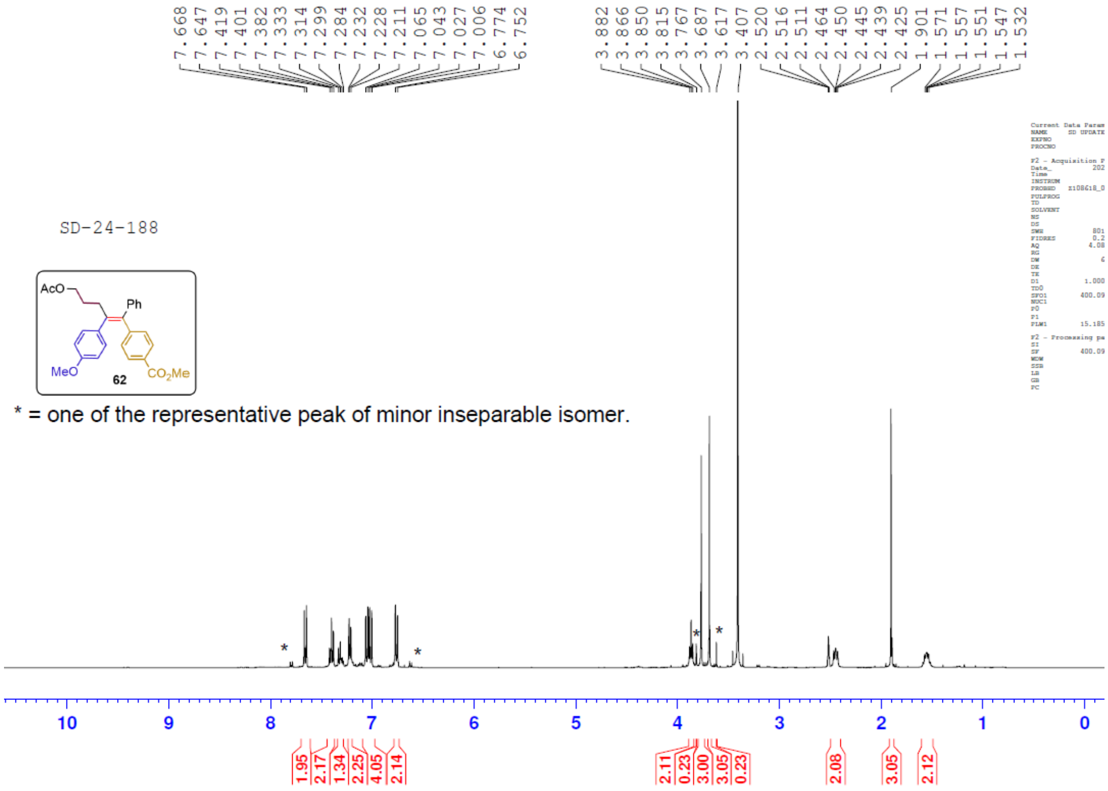

Supplementary Figure 171. ^{1}H NMR of compound **62**

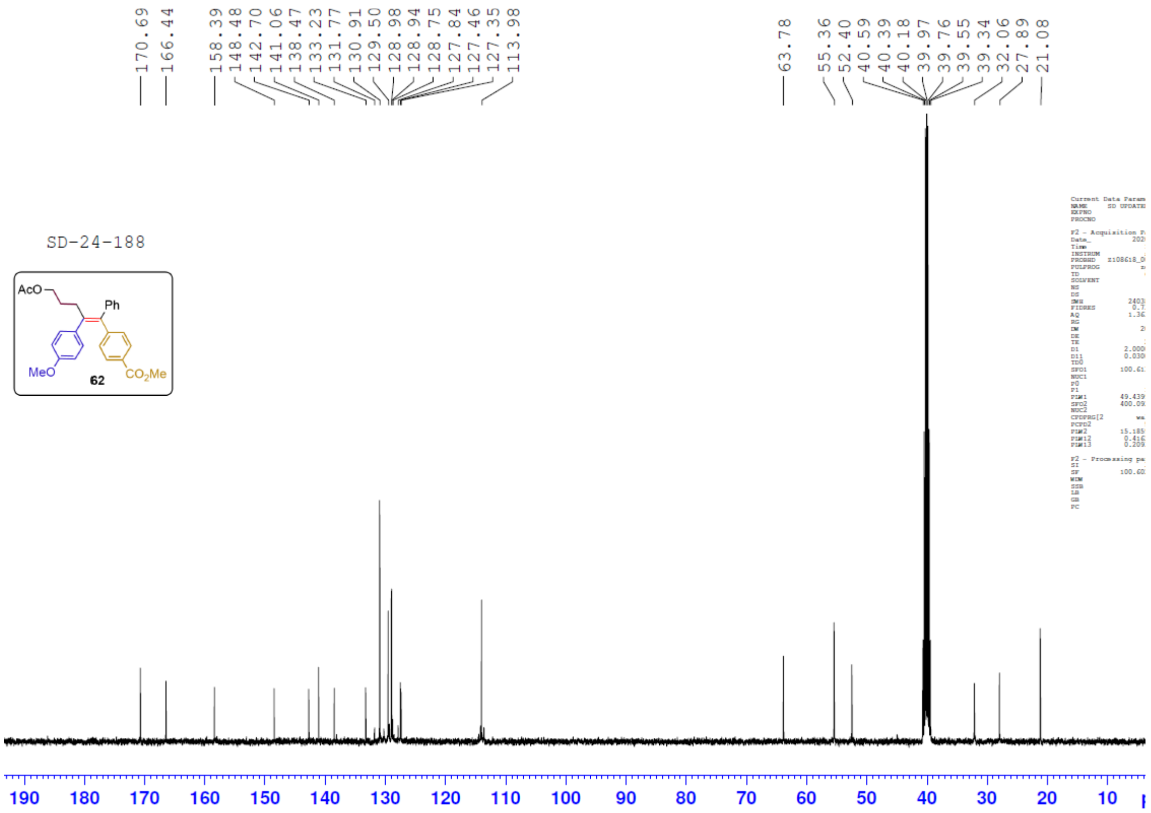

Supplementary Figure 172. ^{13}C NMR of compound **62**

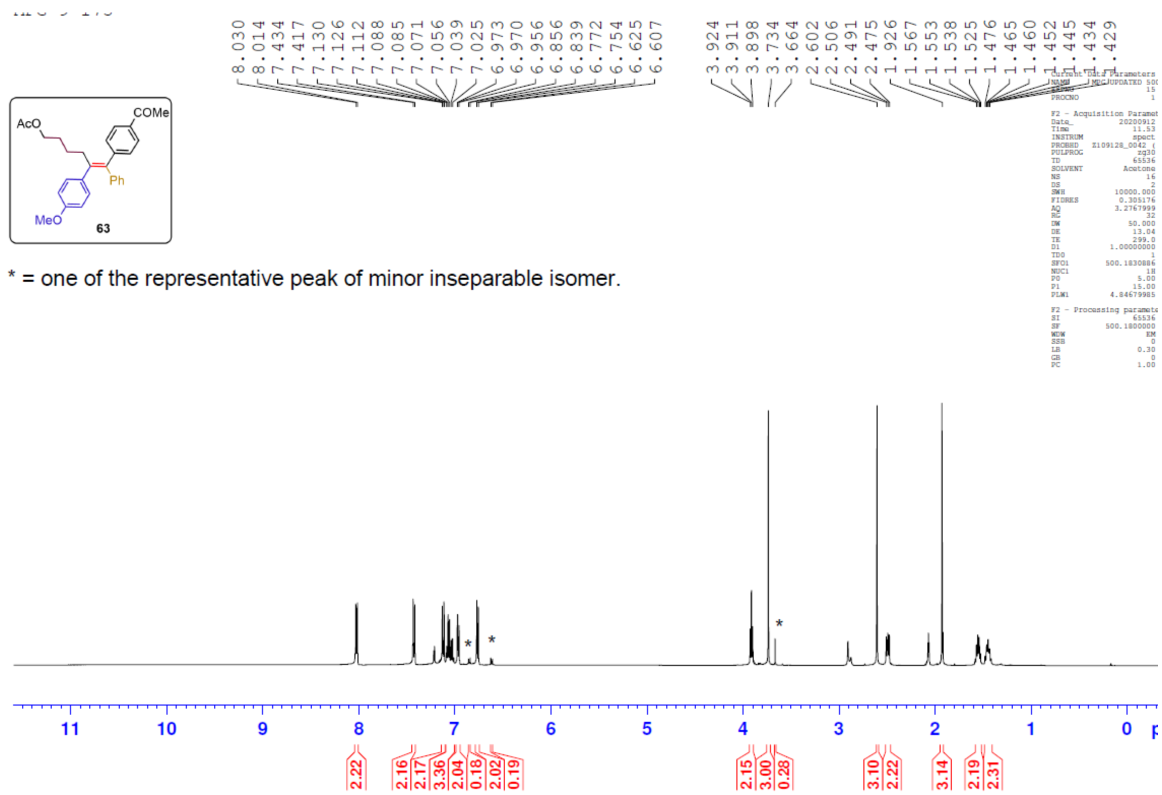

Supplementary Figure 173. ^{1}H NMR of compound **63**

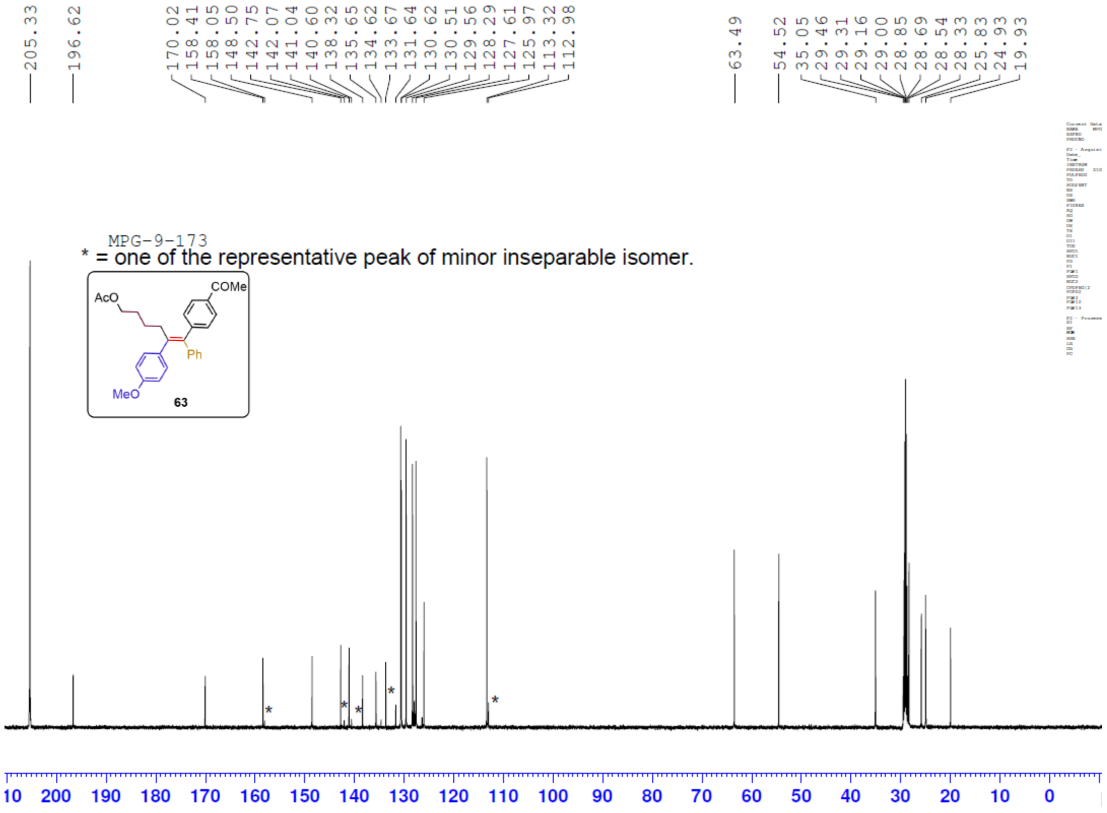

Supplementary Figure 174. ^{13}C NMR of compound **63**

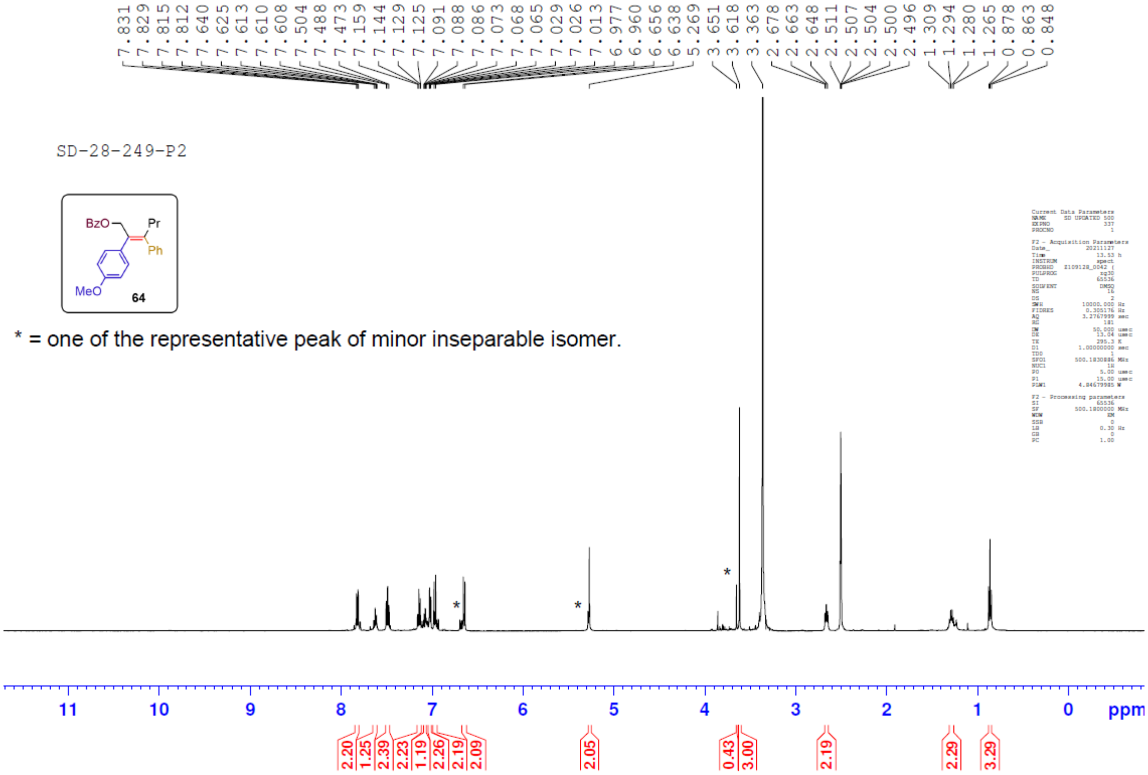

Supplementary Figure 175. ^{1}H NMR of compound **64**

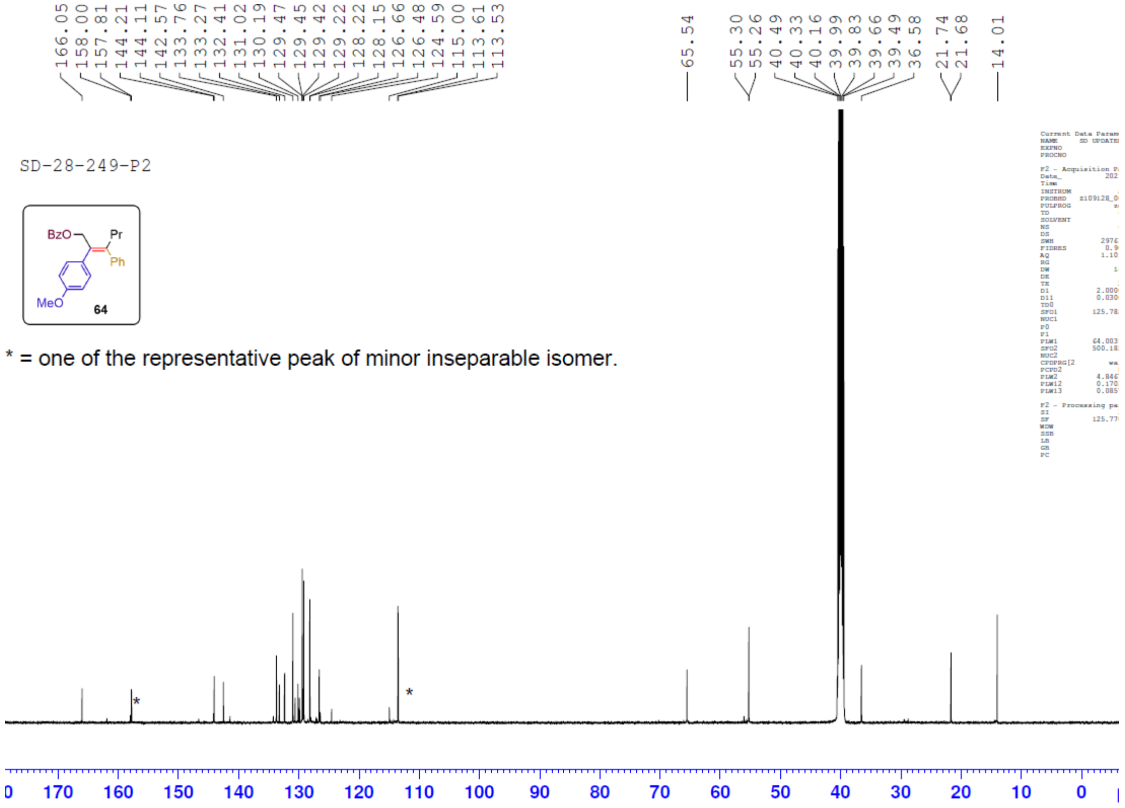

Supplementary Figure 176. ^{13}C NMR of compound **64**

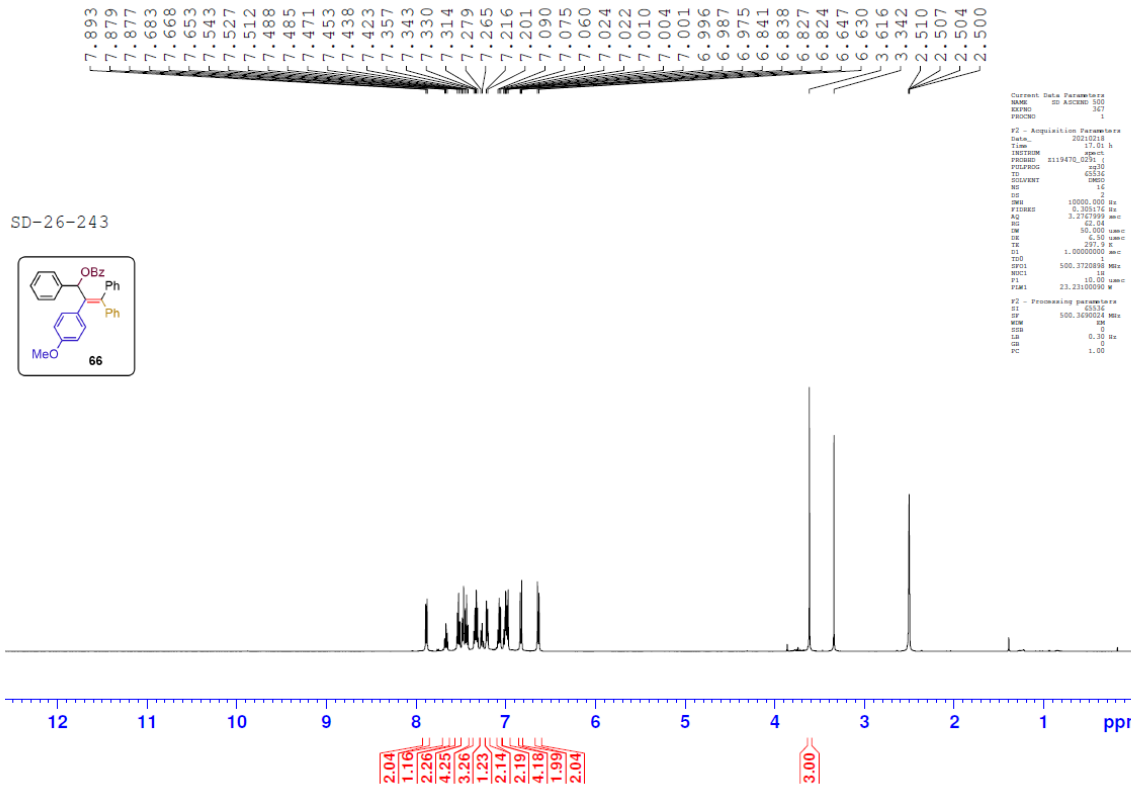

Supplementary Figure 177. ^{1}H NMR of compound **65**

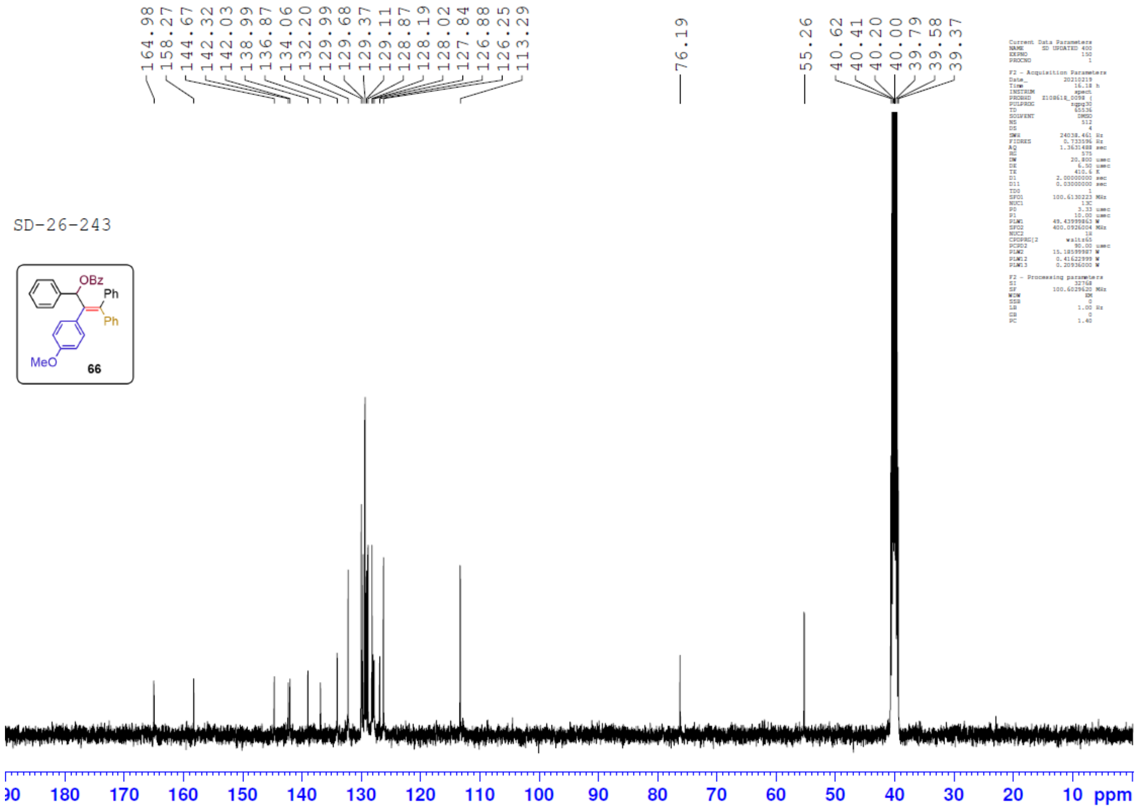

Supplementary Figure 178. ^{13}C NMR of compound **65**

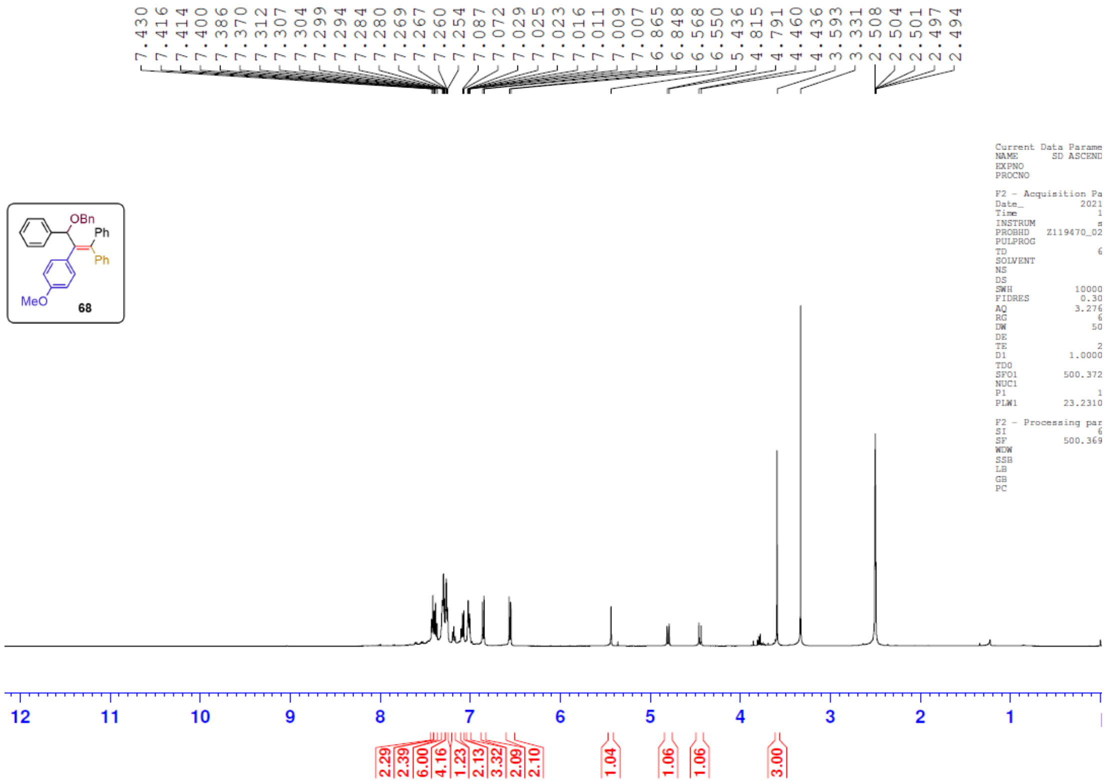

Supplementary Figure 179. ^{1}H NMR of compound **66**

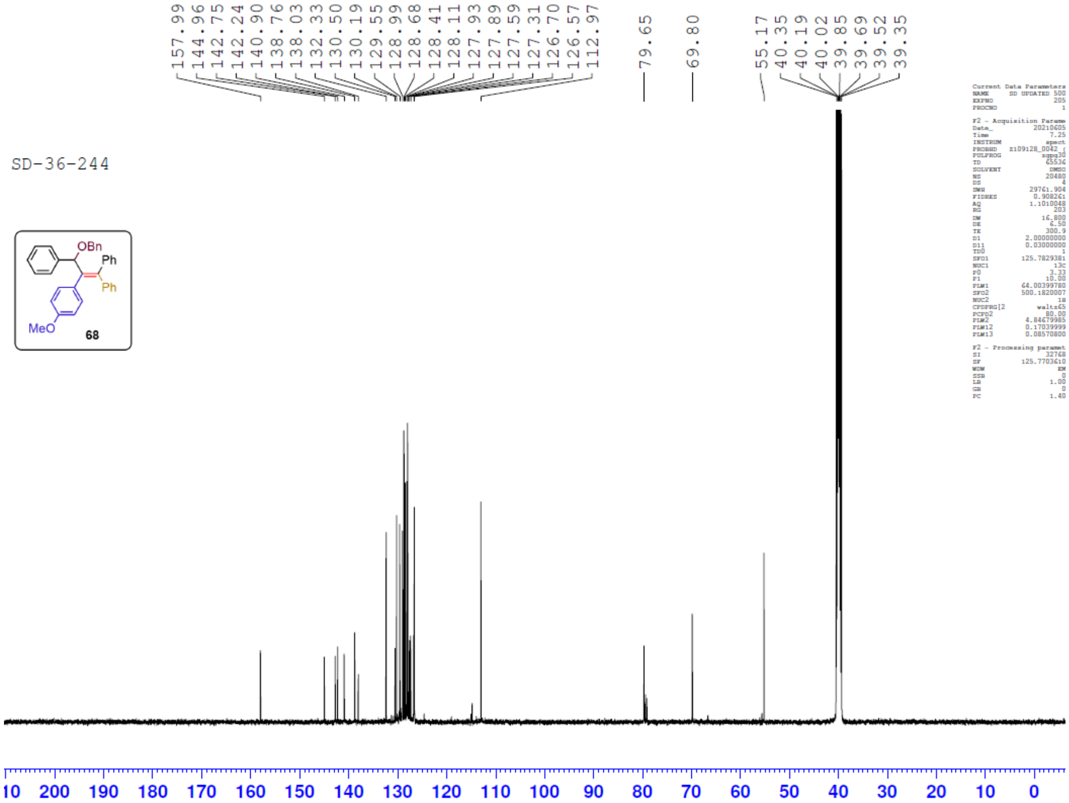

Supplementary Figure 180. ^{13}C NMR of compound **66**

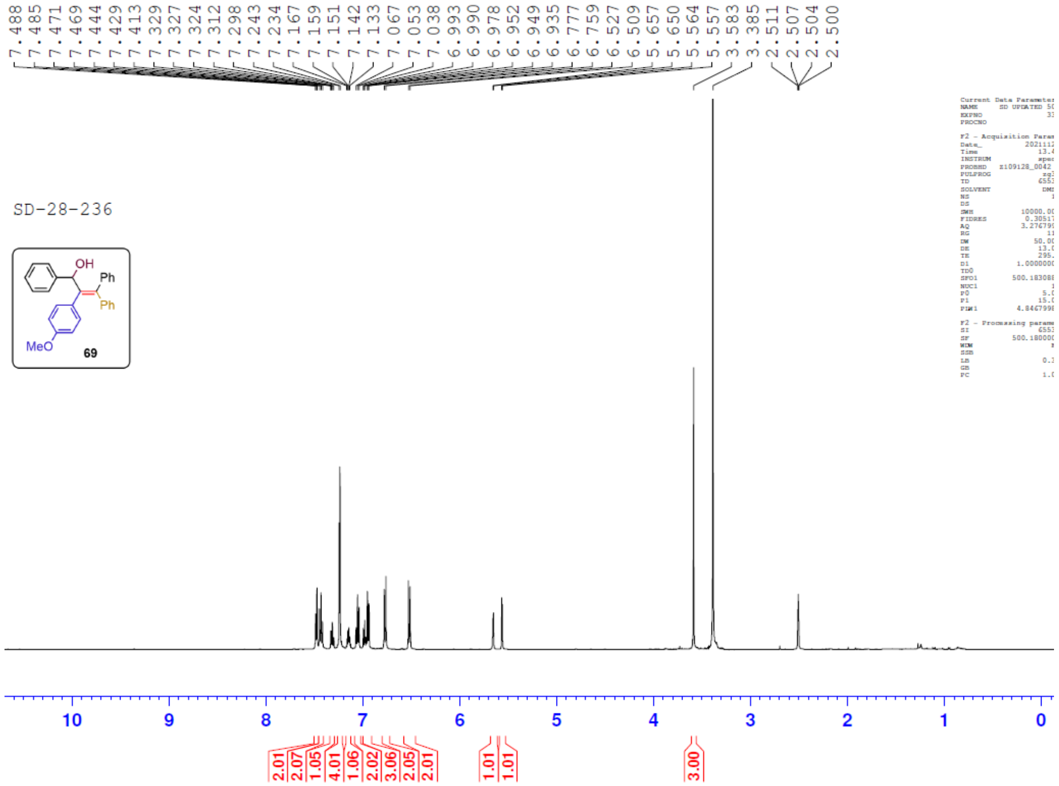

Supplementary Figure 181. ^{1}H NMR of compound **69**

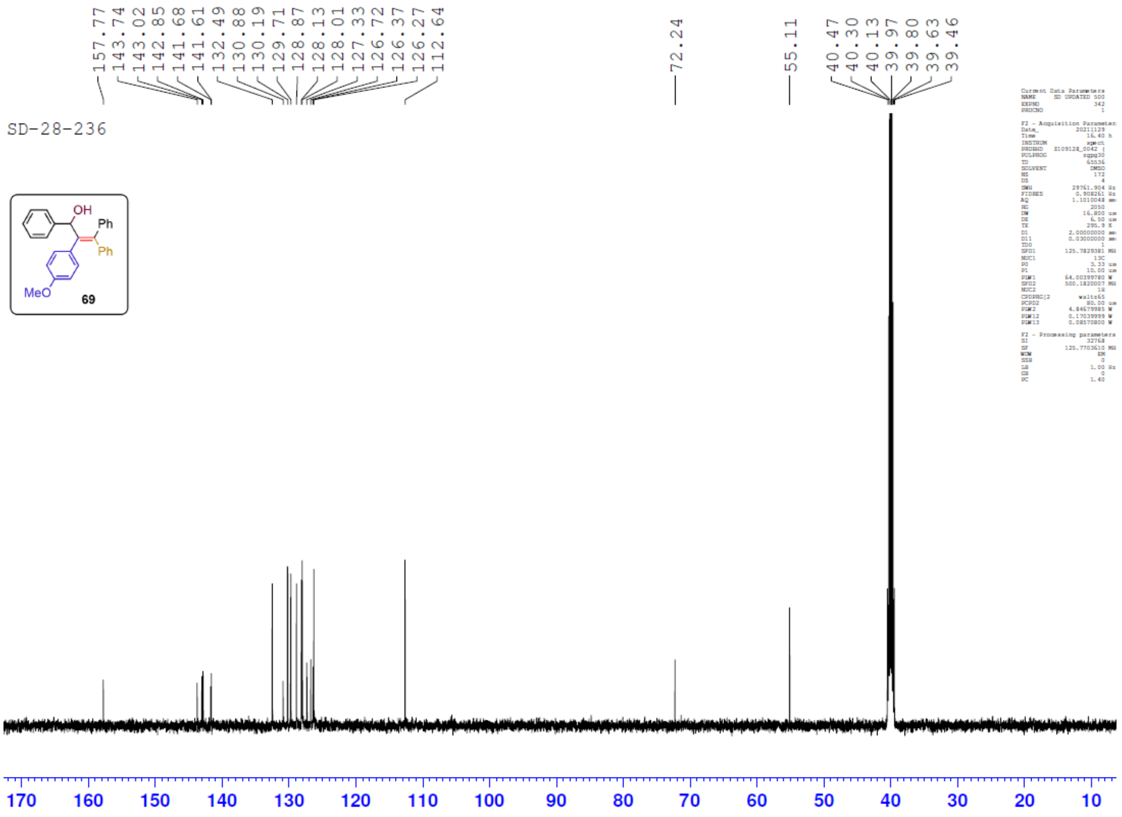

Supplementary Figure 182. ^{13}C NMR of compound **69**

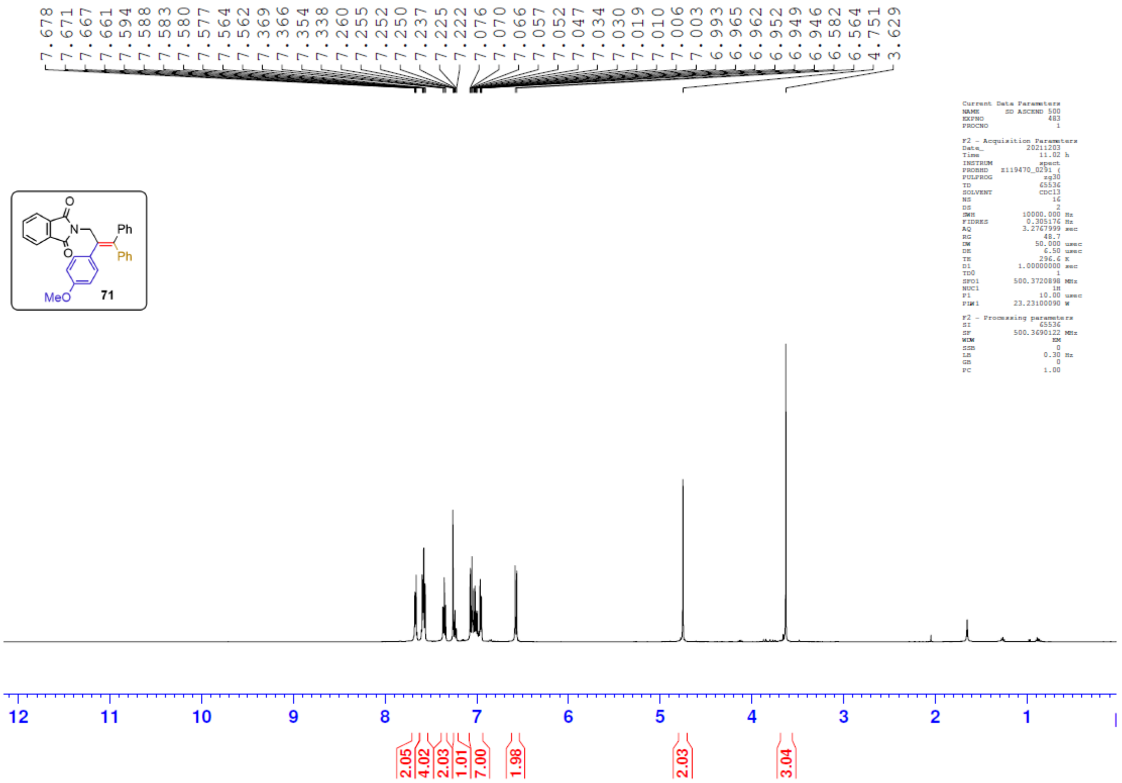

Supplementary Figure 183. ^{1}H NMR of compound **71**

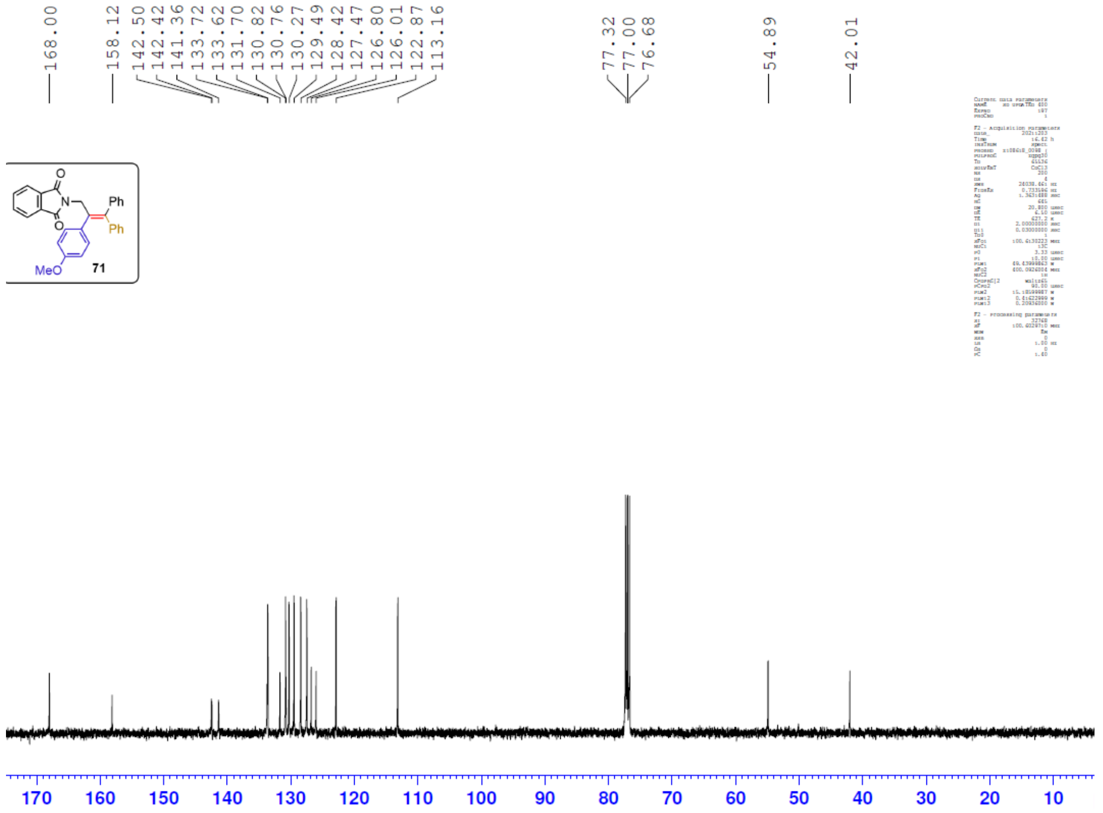

Supplementary Figure 184. ^{1}H NMR of compound **71**

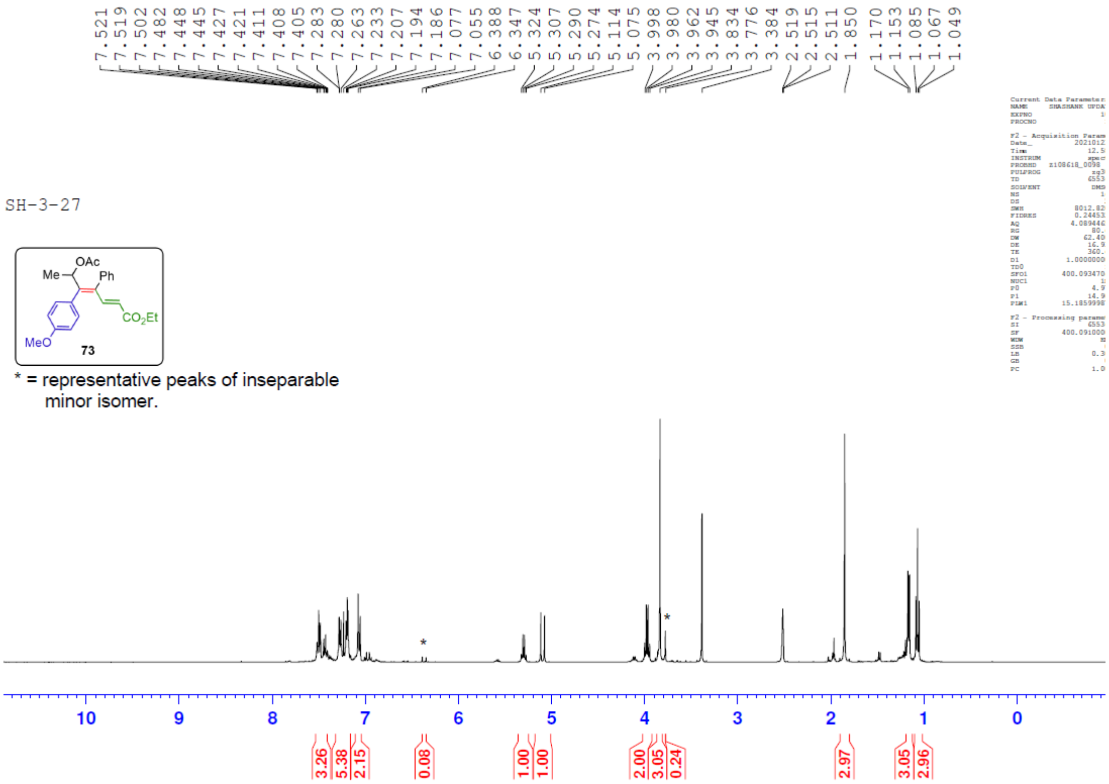

Supplementary Figure 185. ^{1}H NMR of compound **73**

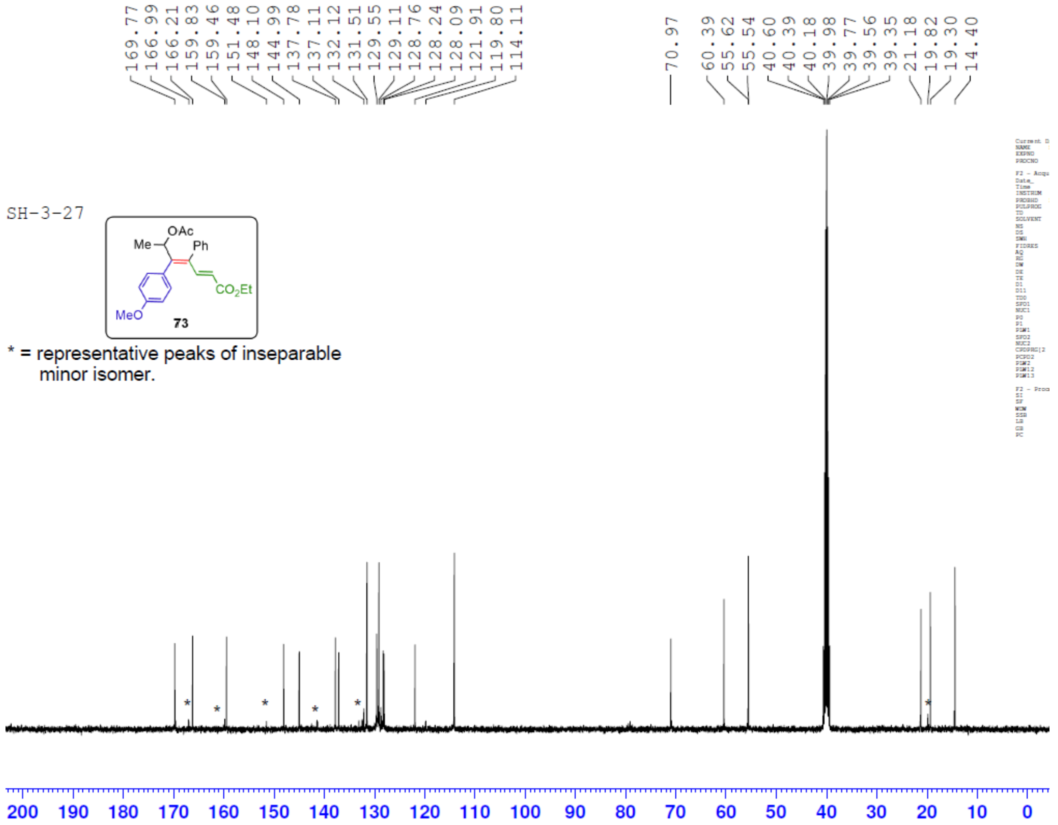

Supplementary Figure 186. ^{13}C NMR of compound **73**

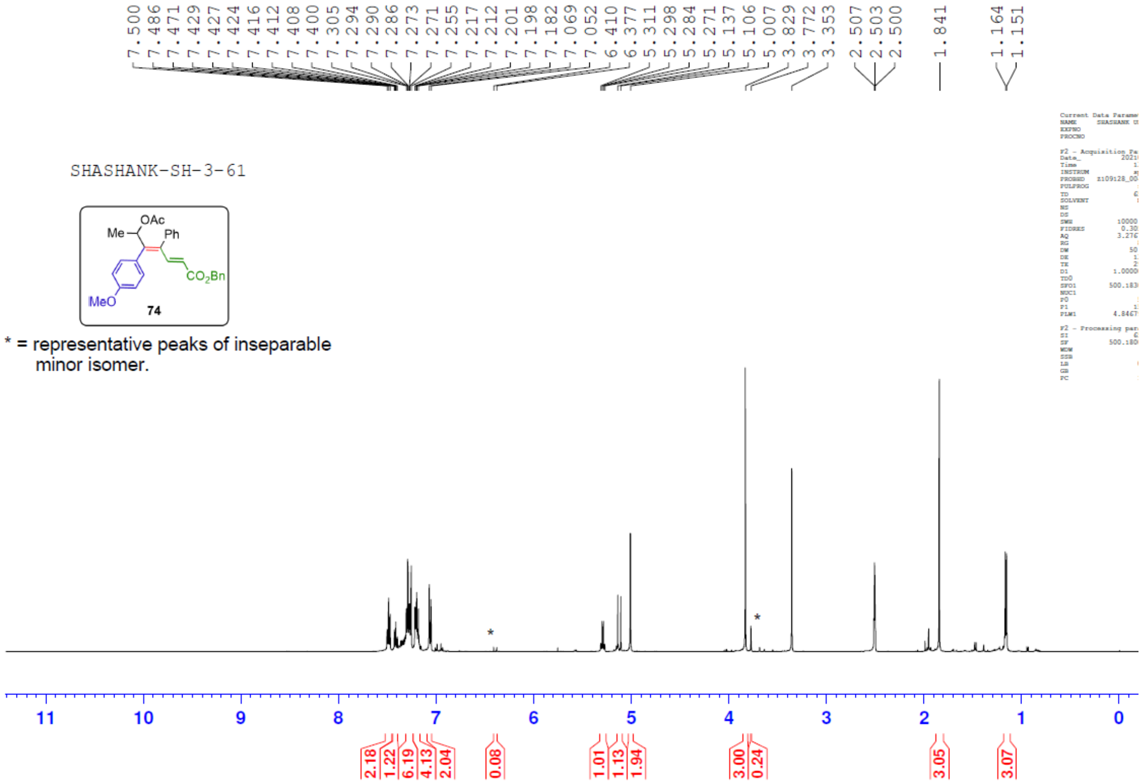

Supplementary Figure 187. ^{1}H NMR of compound **74**

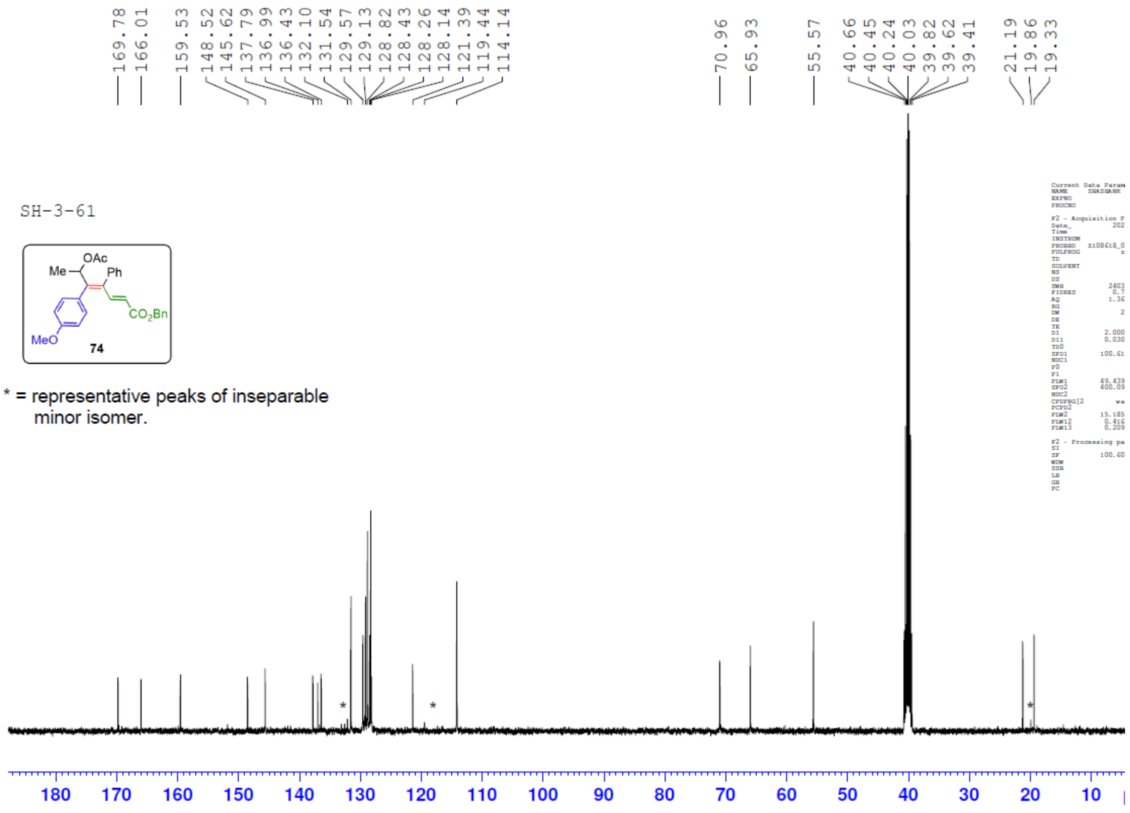

Supplementary Figure 188. ^{13}C NMR of compound **74**

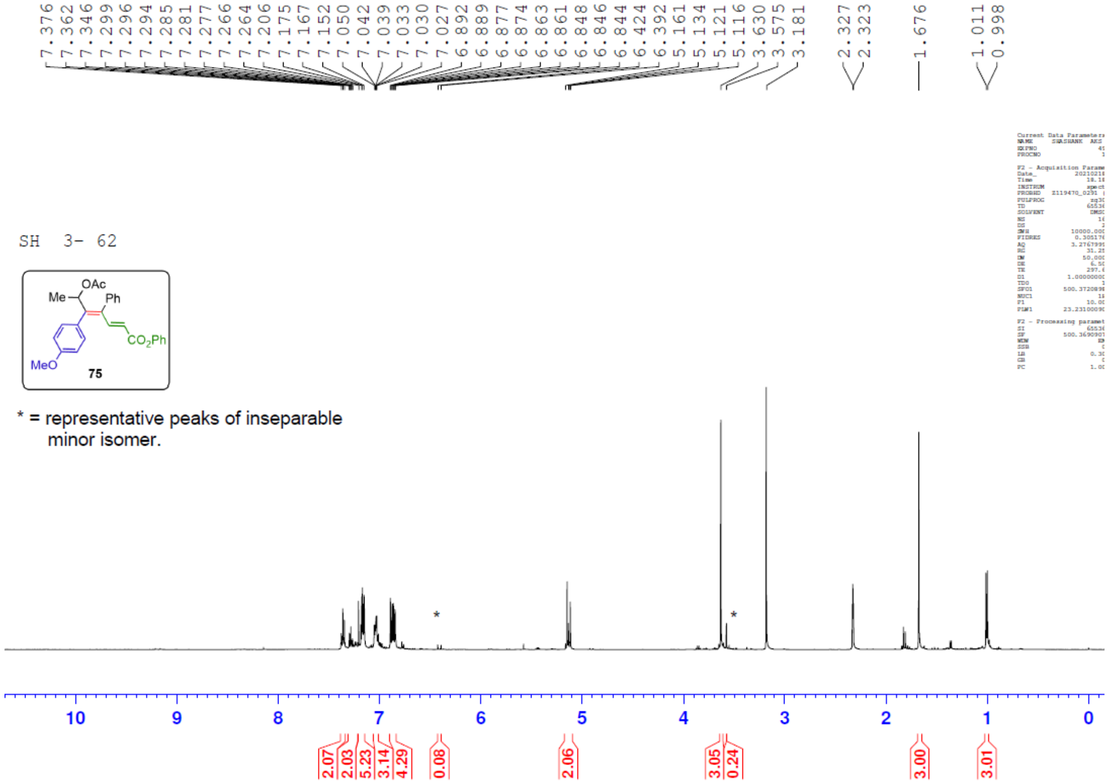

Supplementary Figure 189. ^{13}C NMR of compound **75**

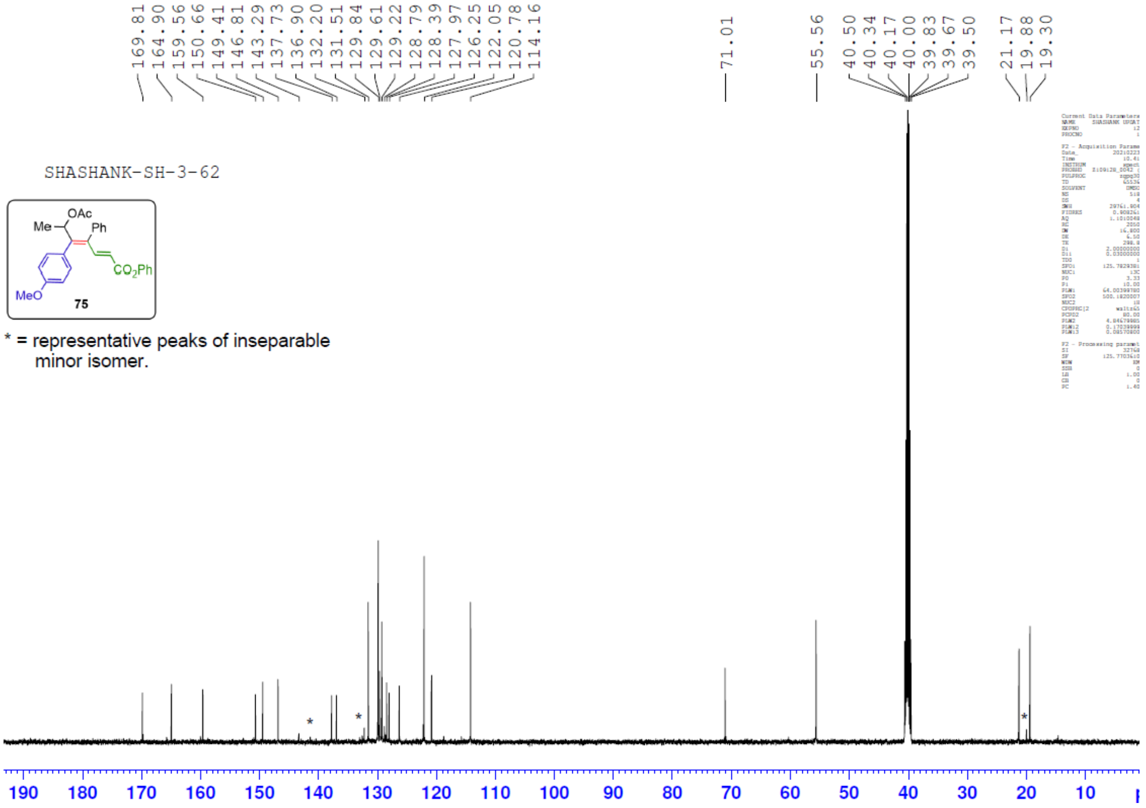

Supplementary Figure 190. ^{13}C NMR of compound **75**

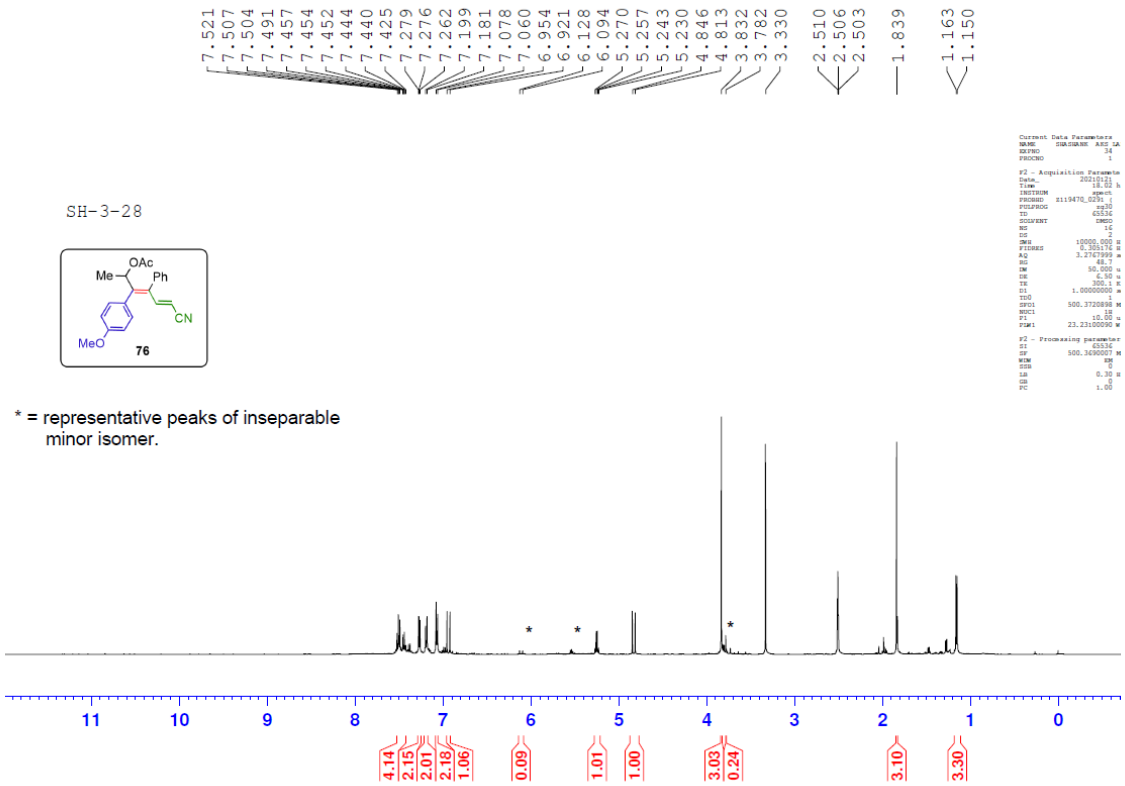

Supplementary Figure 191. ^{1}H NMR of compound **76**

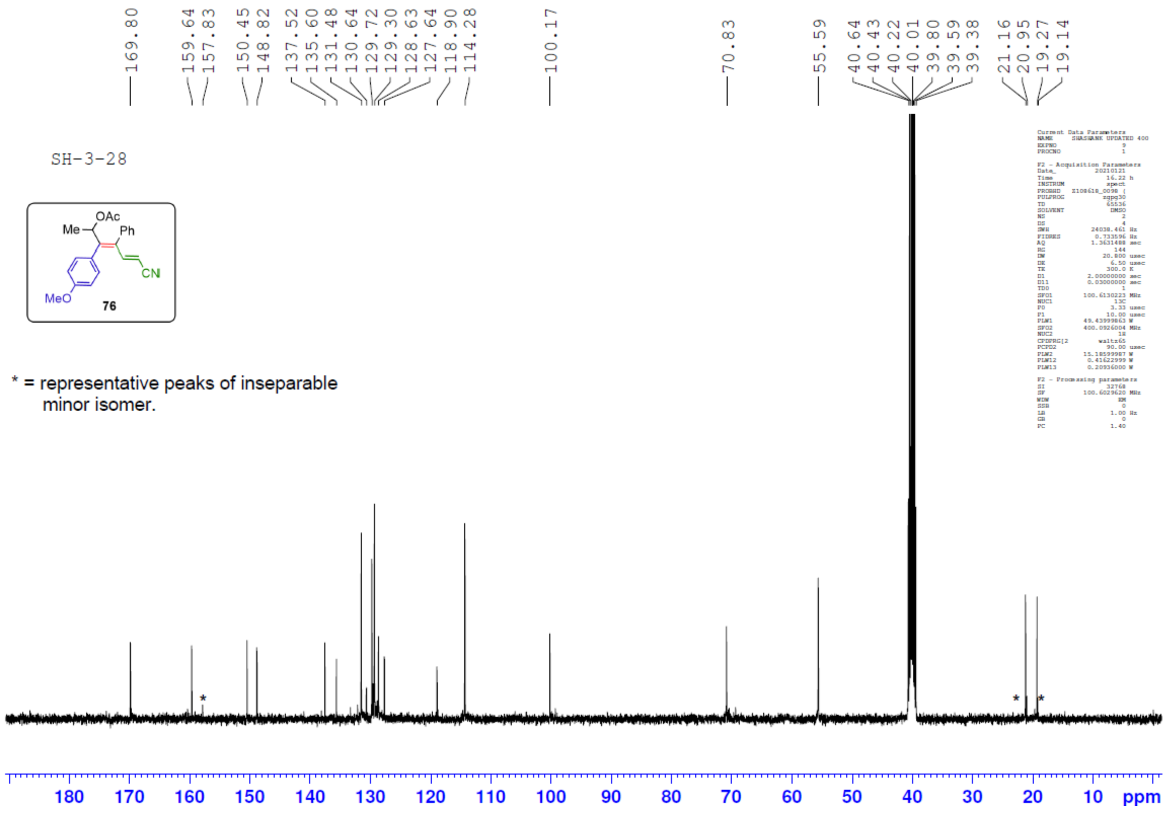

Supplementary Figure 192. ^{13}C NMR of compound **76**

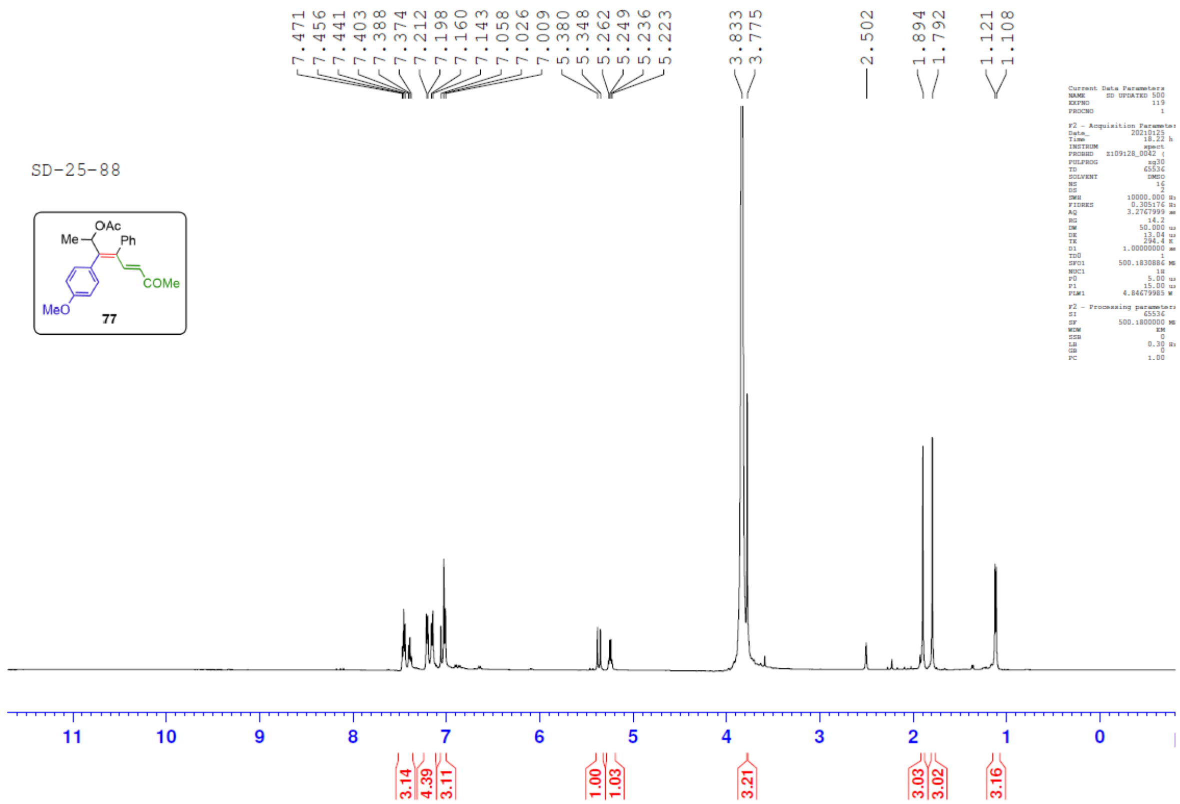

Supplementary Figure 193. ^{1}H NMR of compound **77**

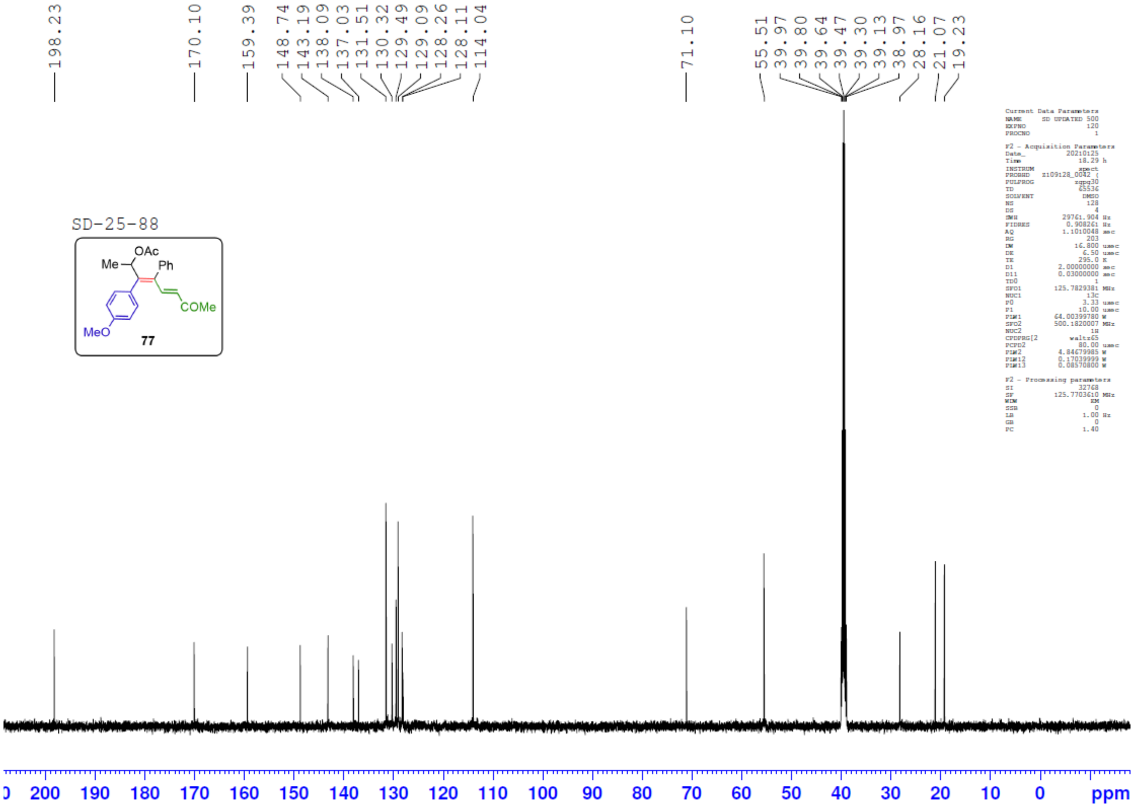

Supplementary Figure 194. ^{13}C NMR of compound **77**

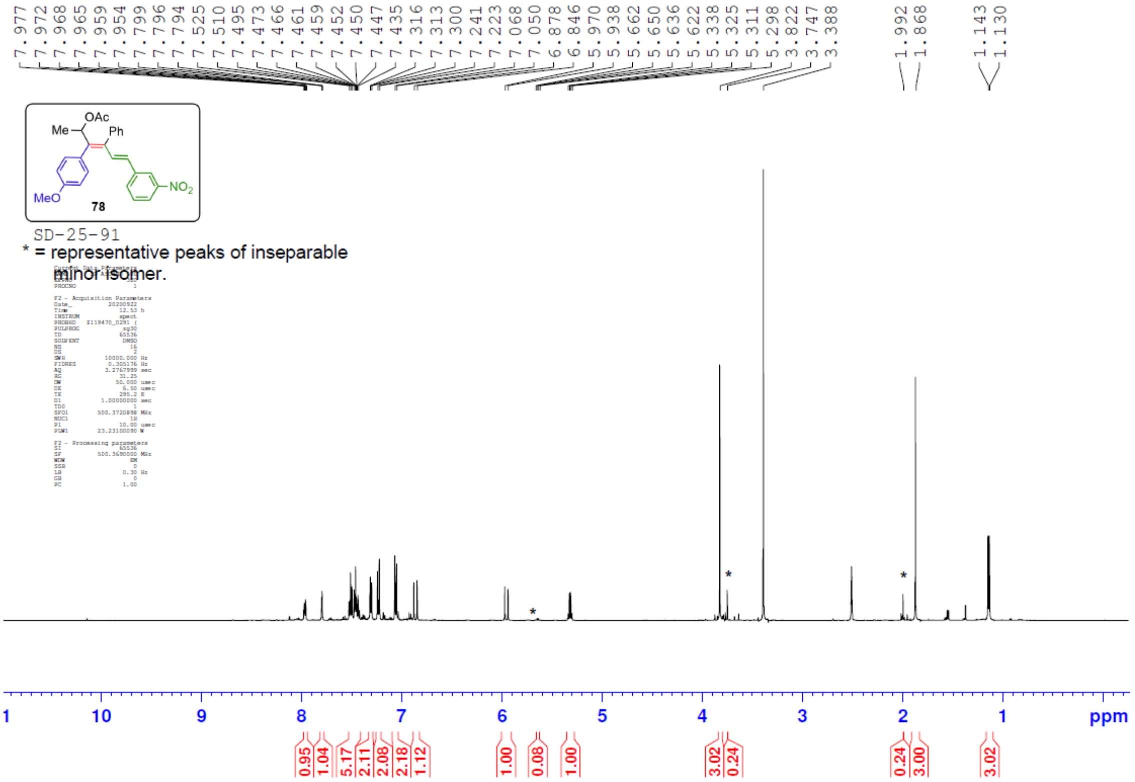

Supplementary Figure 195. ^{1}H NMR of compound **78**

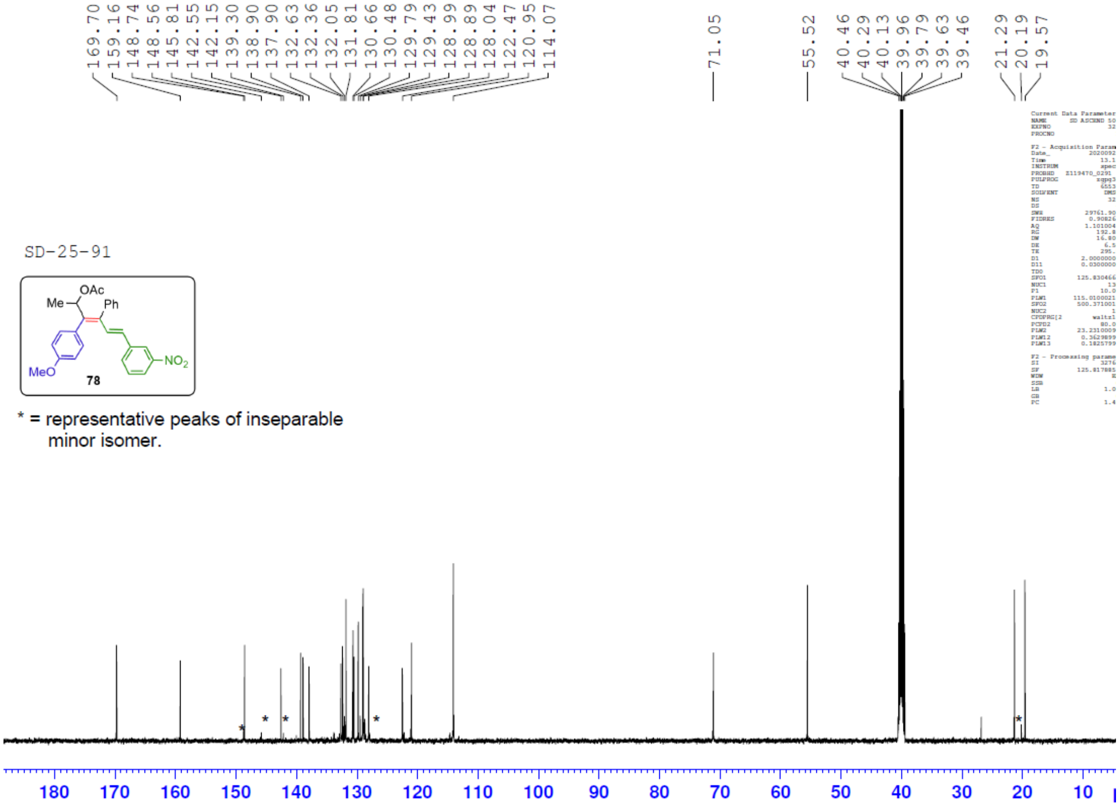

Supplementary Figure 196. ^{13}C NMR of compound **78**

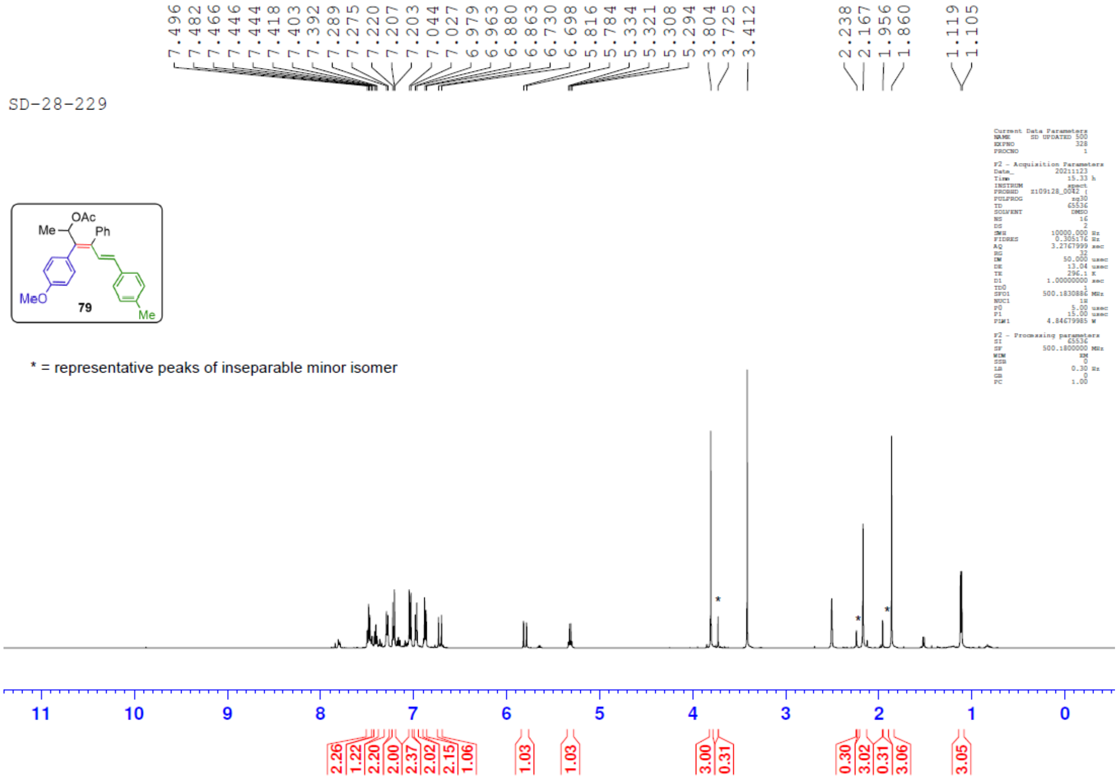

Supplementary Figure 197. ^{1}H NMR of compound **79**

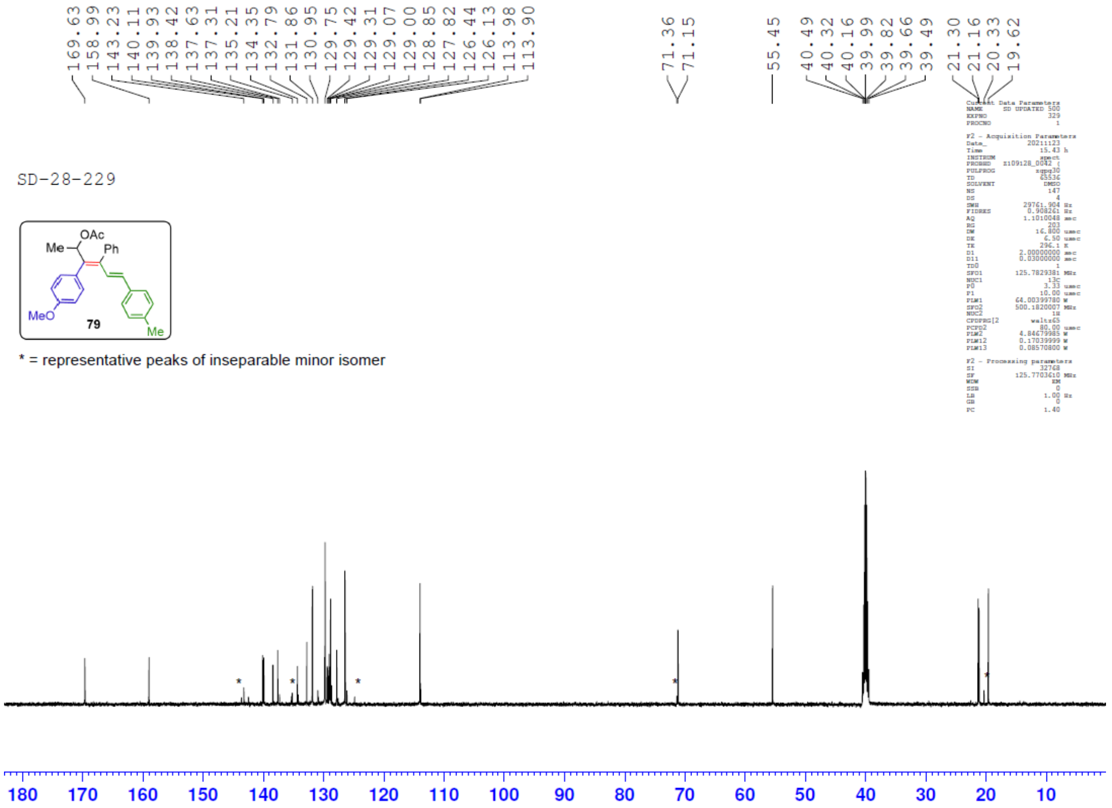

Supplementary Figure 198. ^{13}C NMR of compound **79**

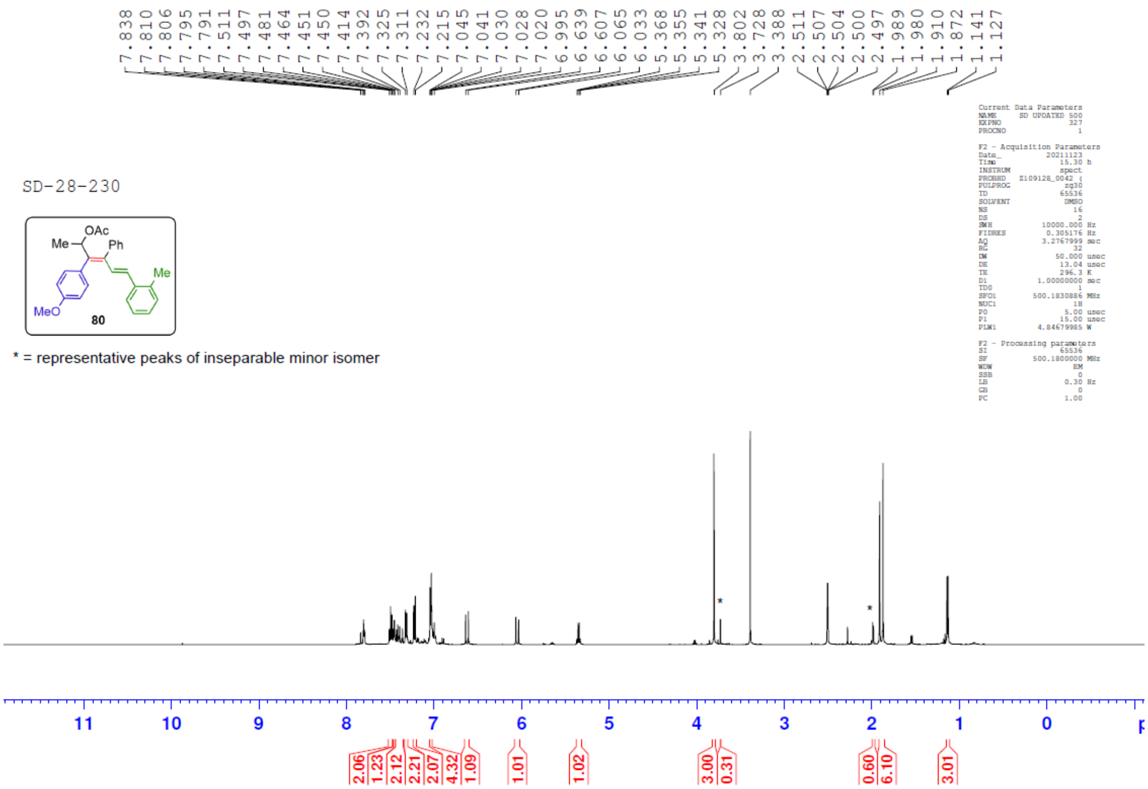

Supplementary Figure 199. ^{1}H NMR of compound **80**

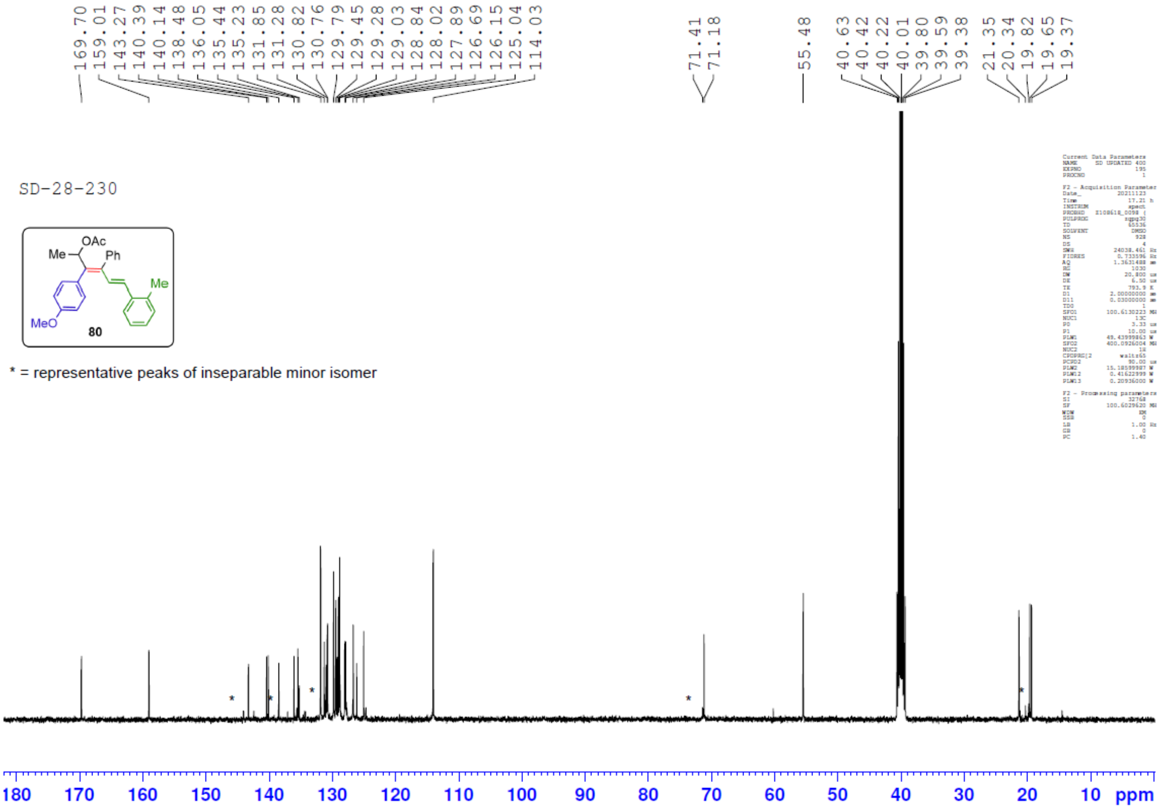

Supplementary Figure 200. ^{13}C NMR of compound **80**

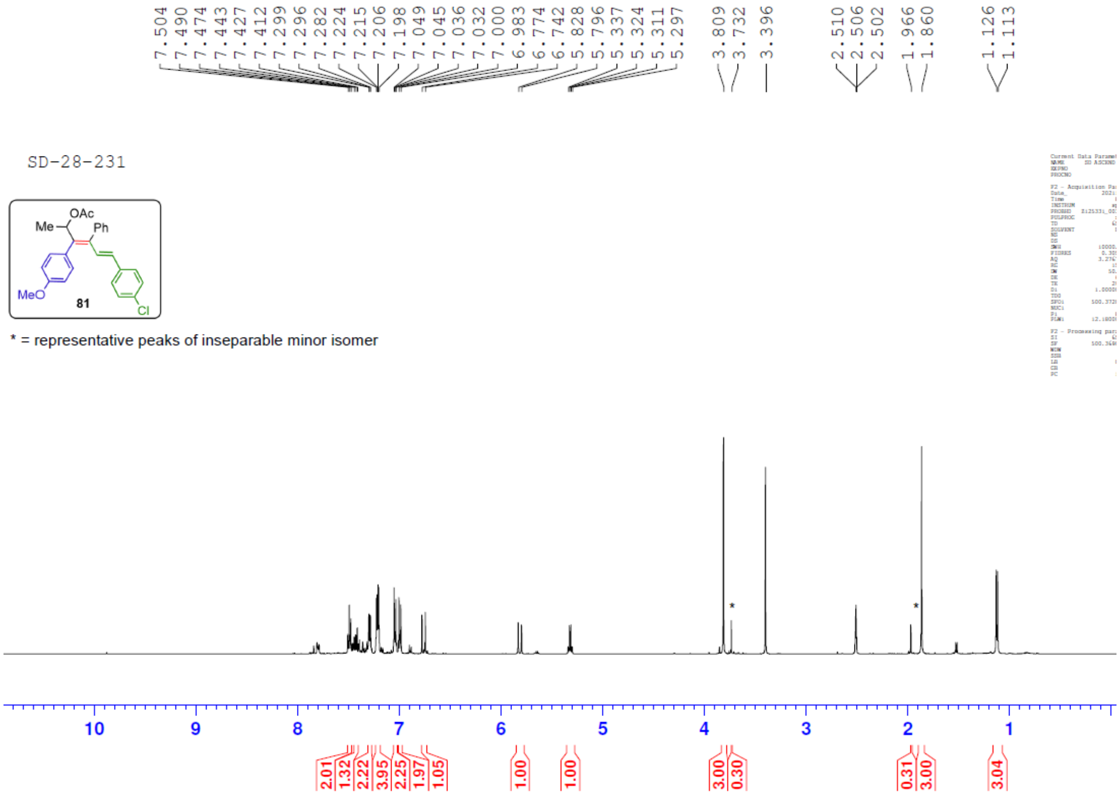

Supplementary Figure 201. ^{1}H NMR of compound **81**

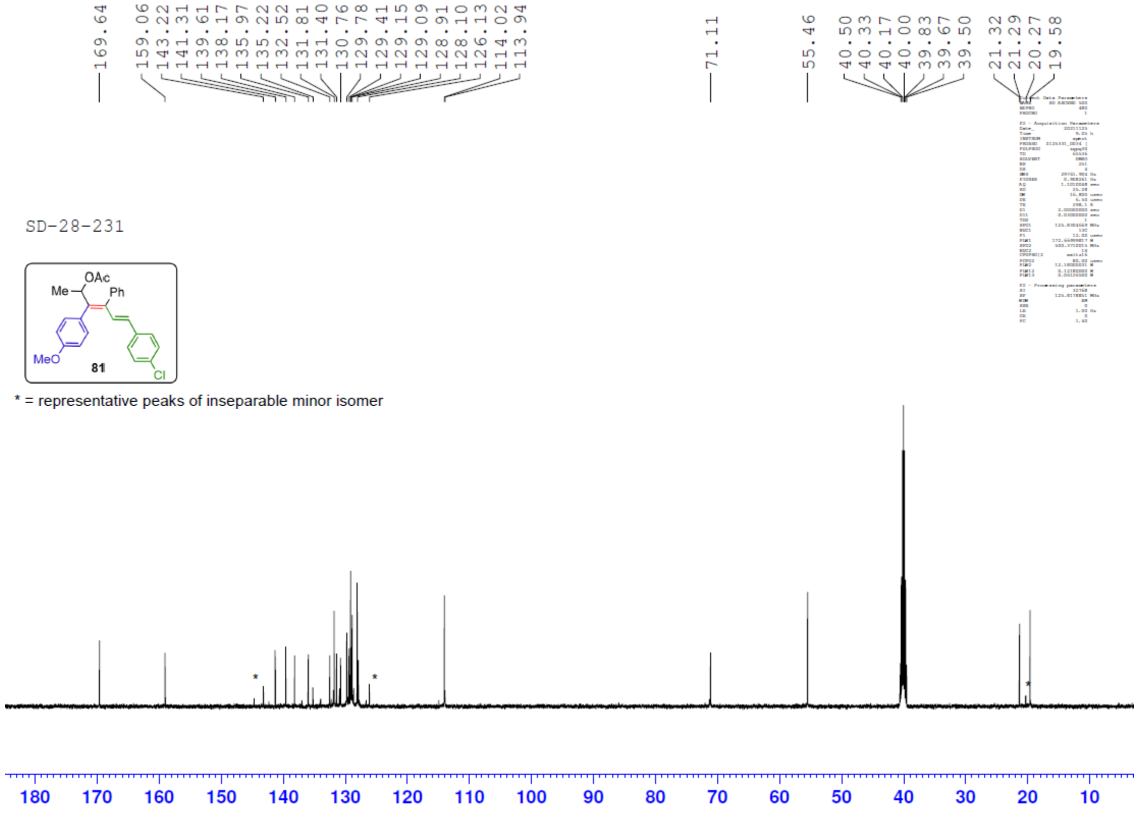

Supplementary Figure 202. ^{13}C NMR of compound **81**

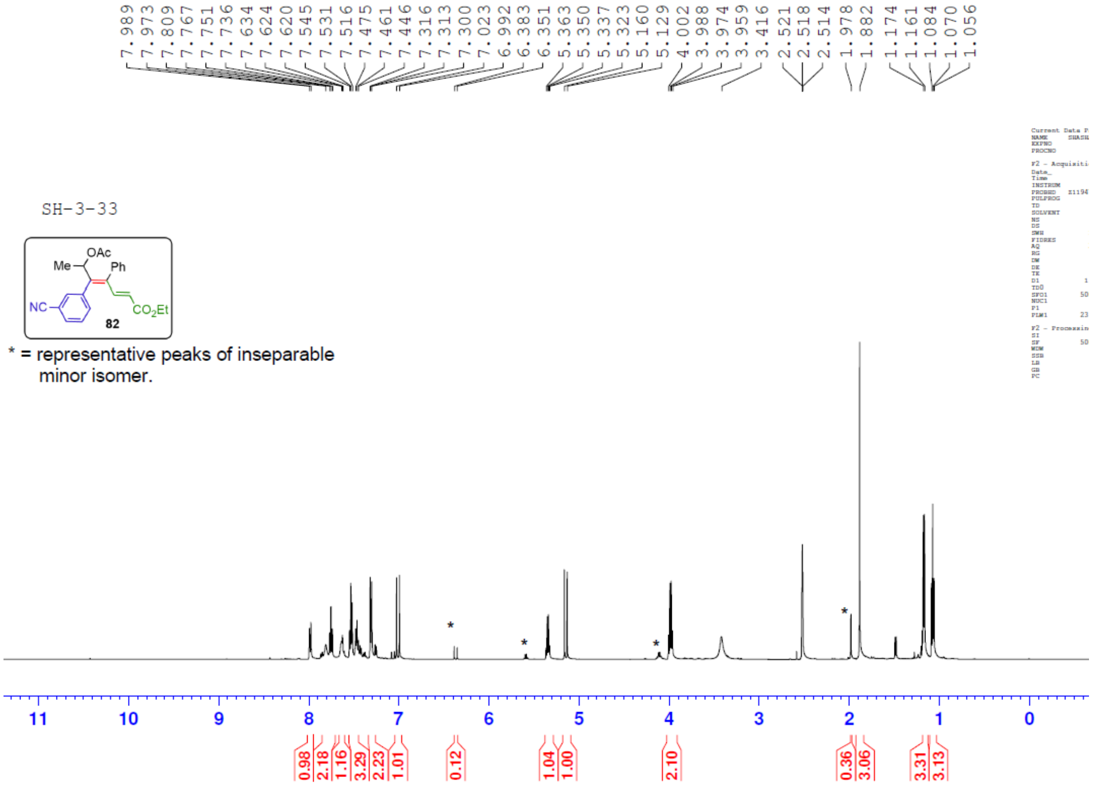

Supplementary Figure 203. ^{1}H NMR of compound **82**

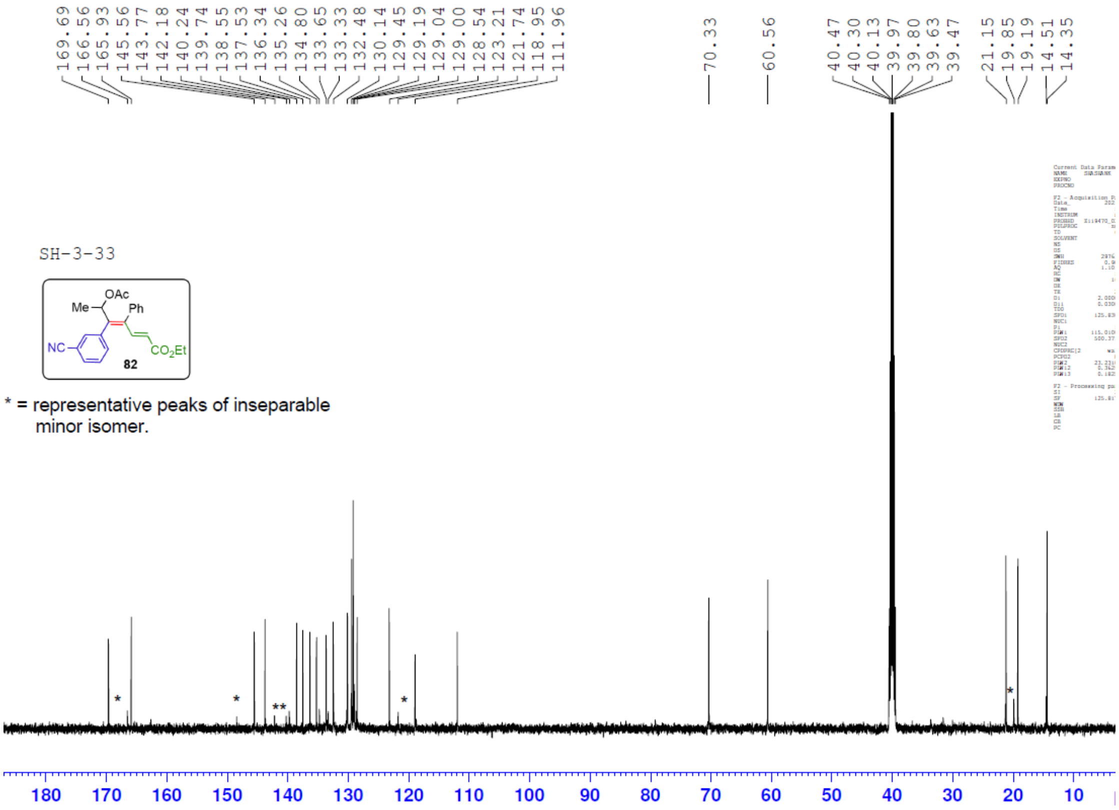

Supplementary Figure 204. ^{13}C NMR of compound **82**

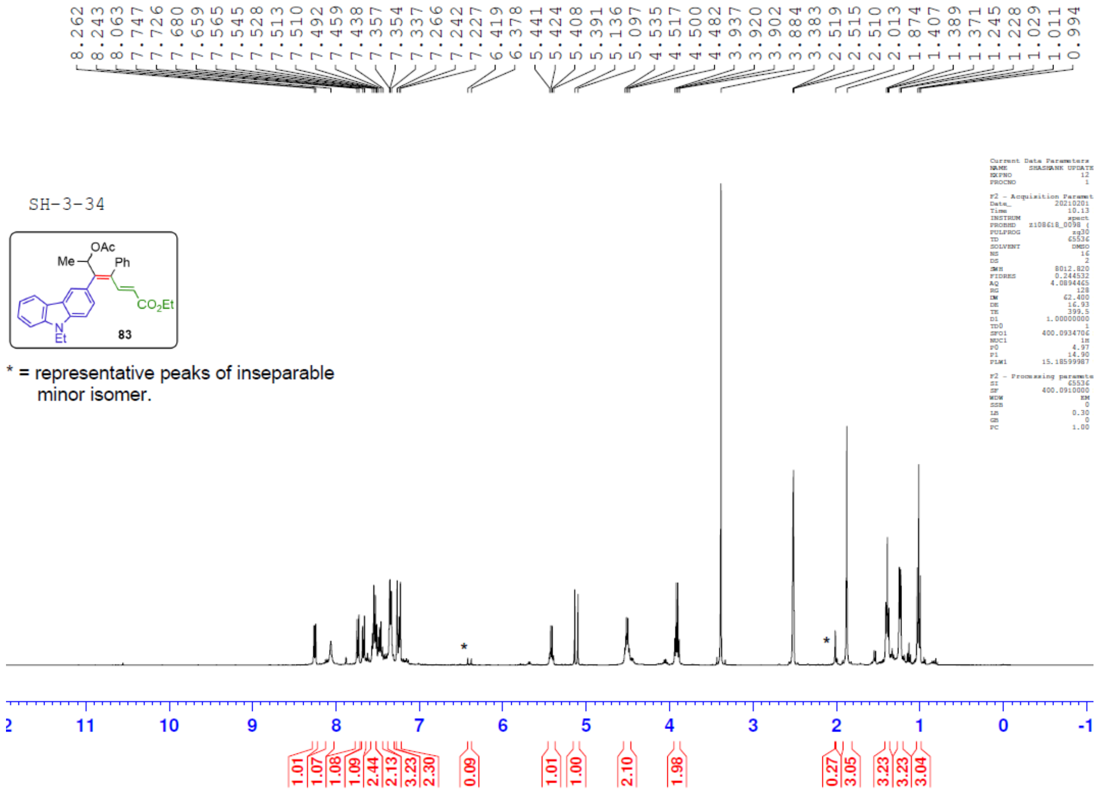

Supplementary Figure 205. ^{1}H NMR of compound **83**

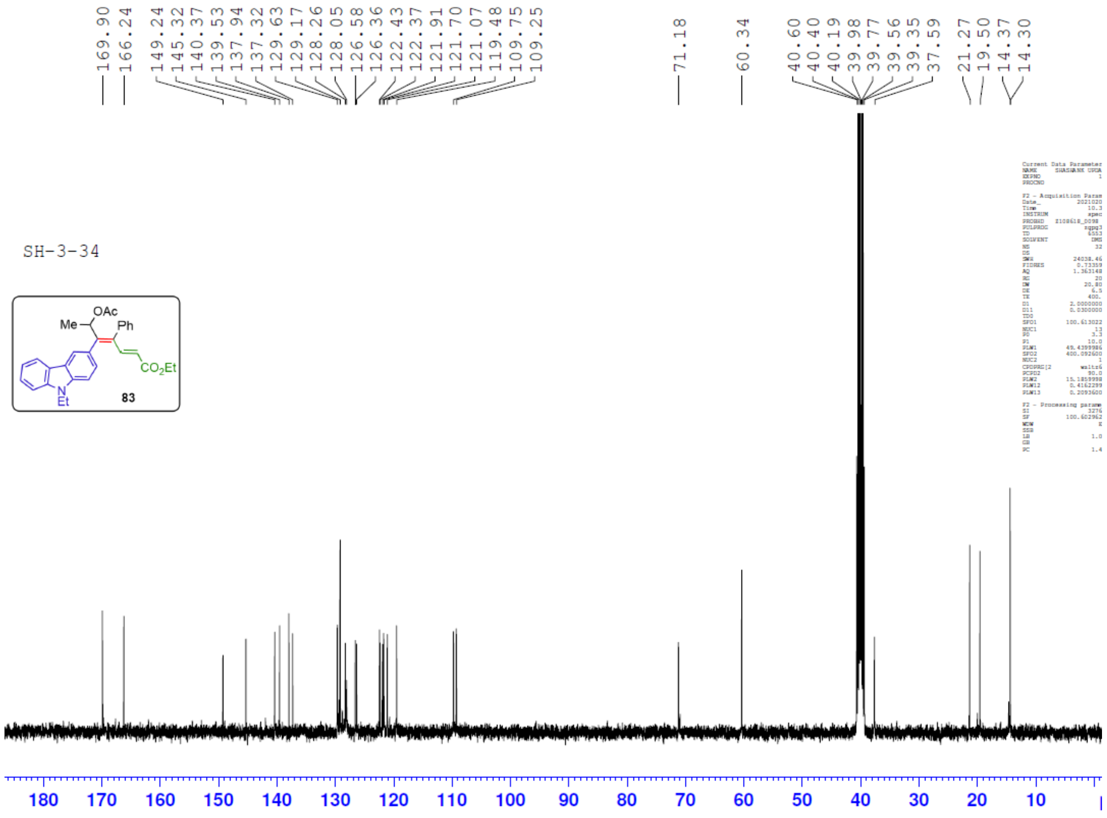

Supplementary Figure 206. ^{13}C NMR of compound **83**

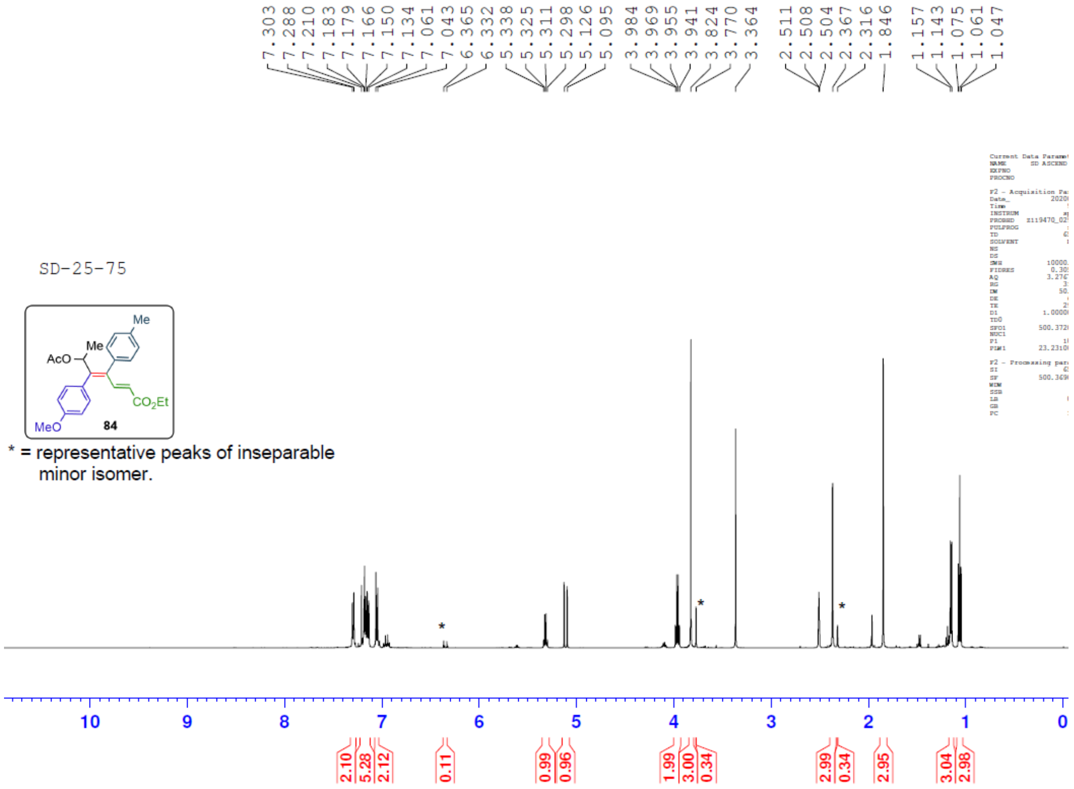

Supplementary Figure 207. ^{1}H NMR of compound **84**

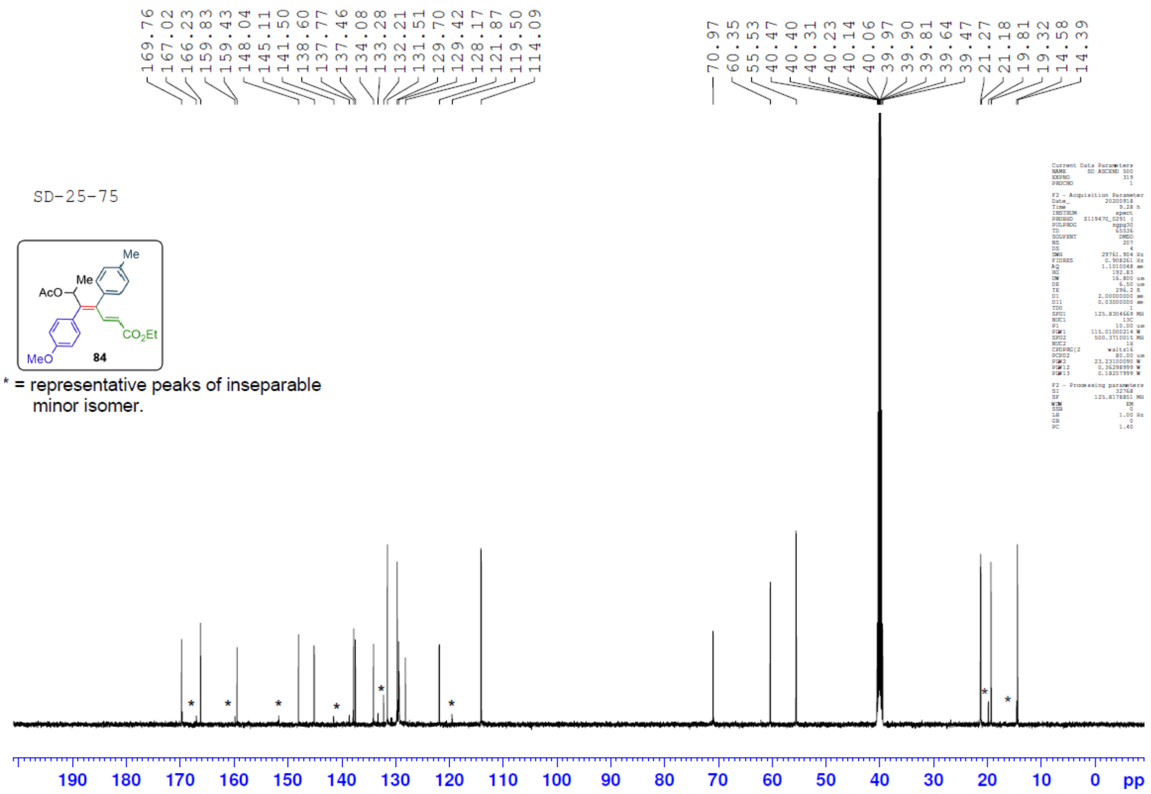

Supplementary Figure 208. ^{13}C NMR of compound **84**

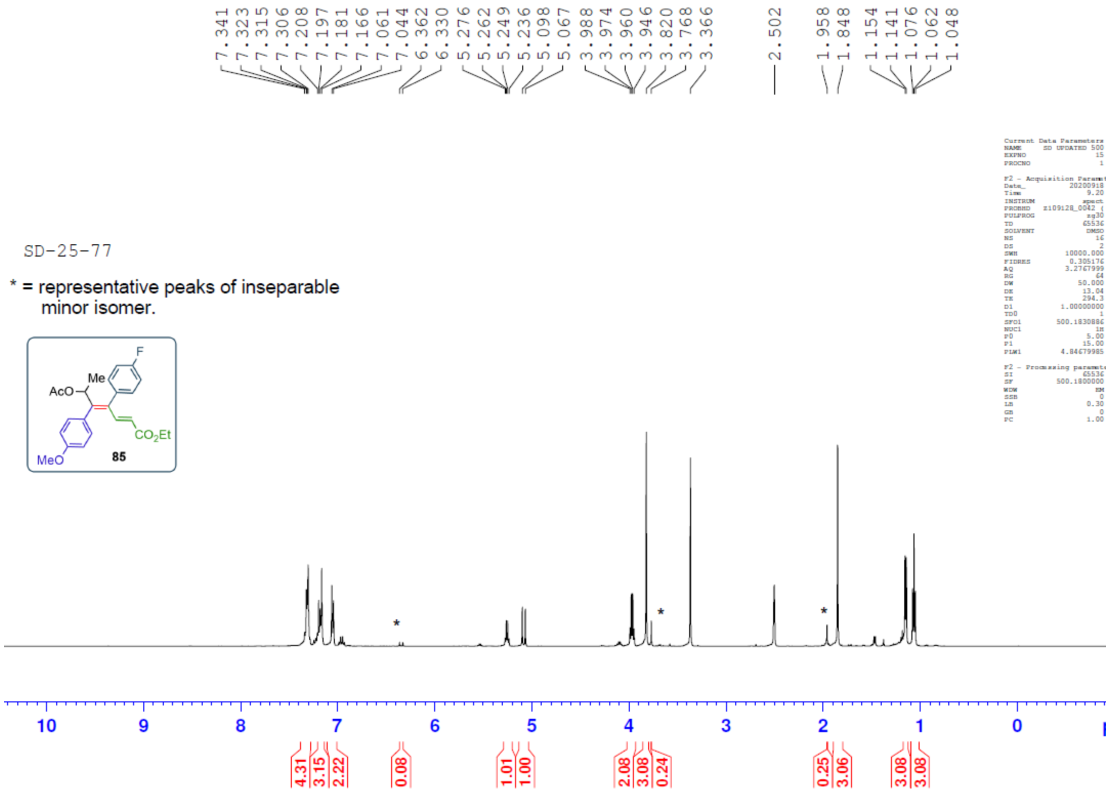

Supplementary Figure 209. ^{1}H NMR of compound **85**

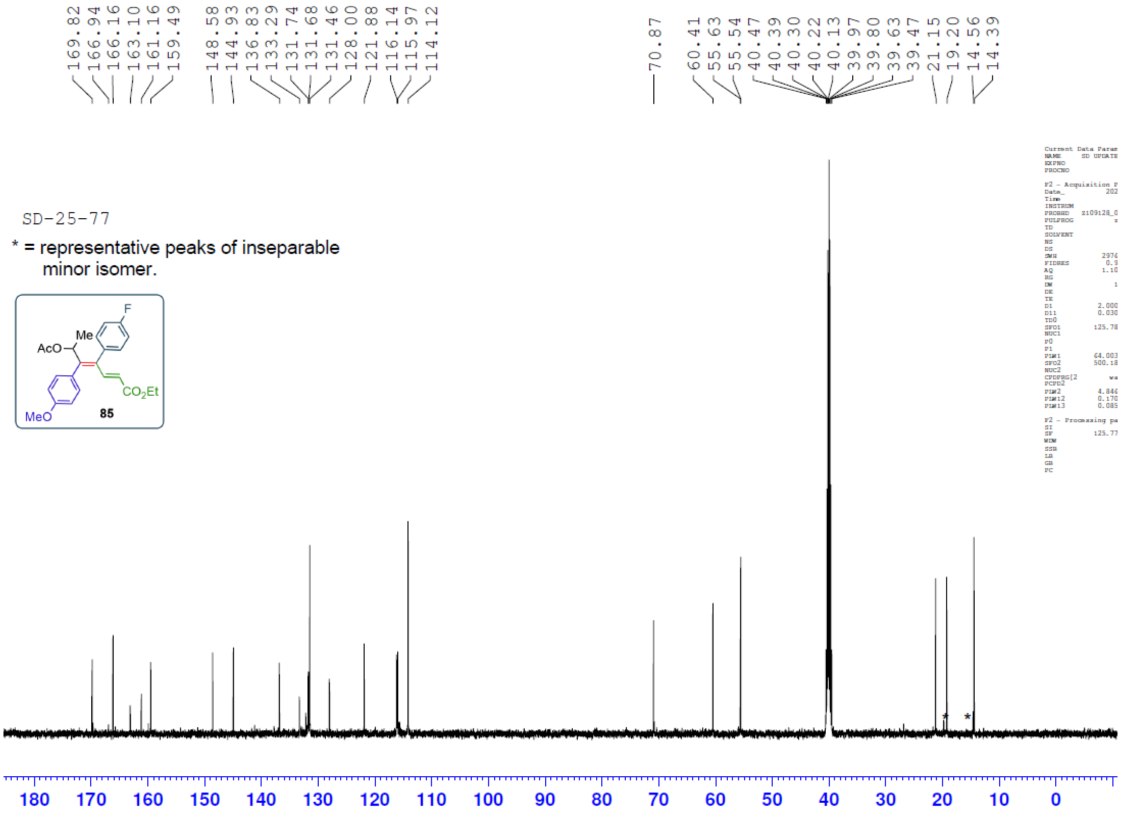

Supplementary Figure 210. ^{13}C NMR of compound **85**

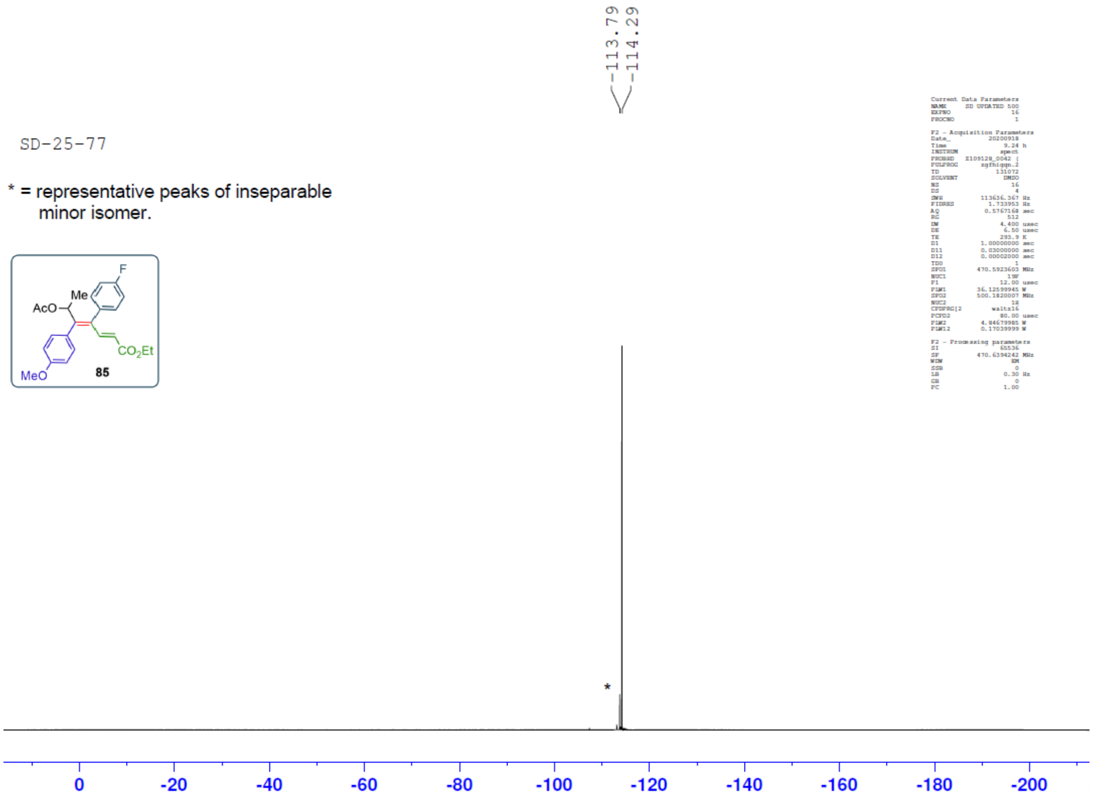

Supplementary Figure 211. ^{19}F NMR of compound **85**

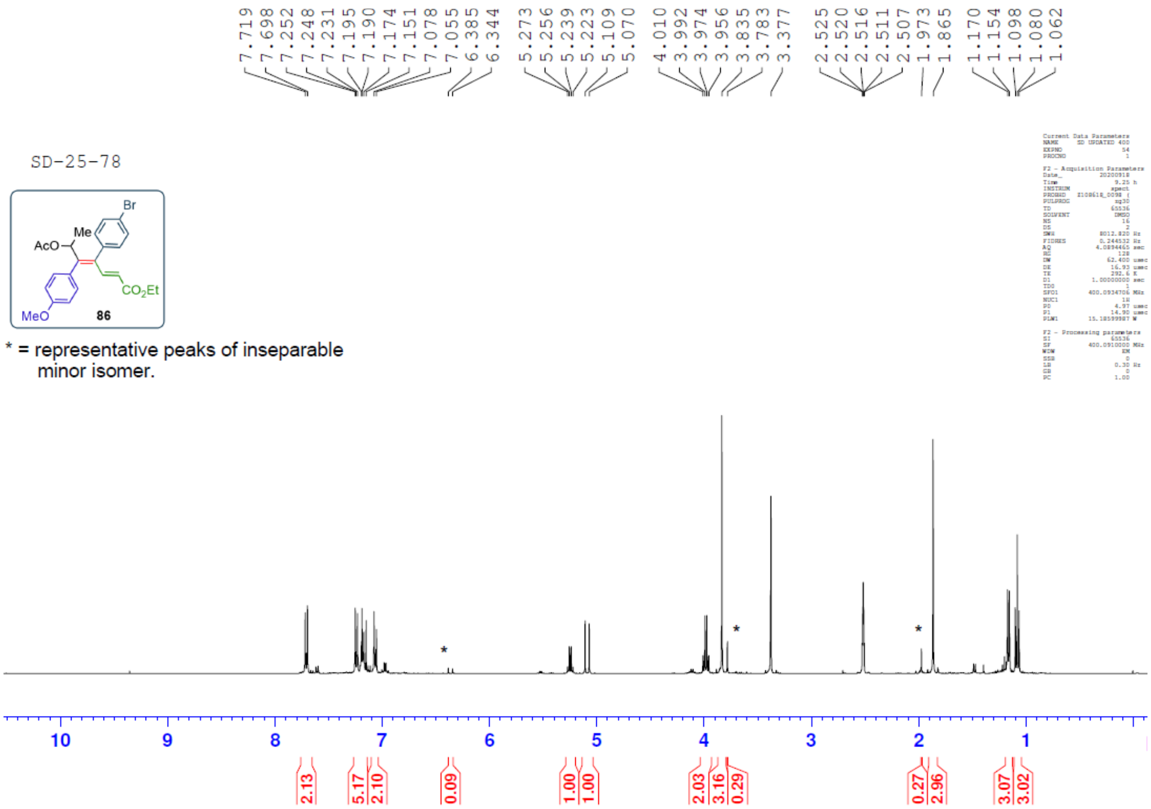

Supplementary Figure 212. ^{1}H NMR of compound **86**

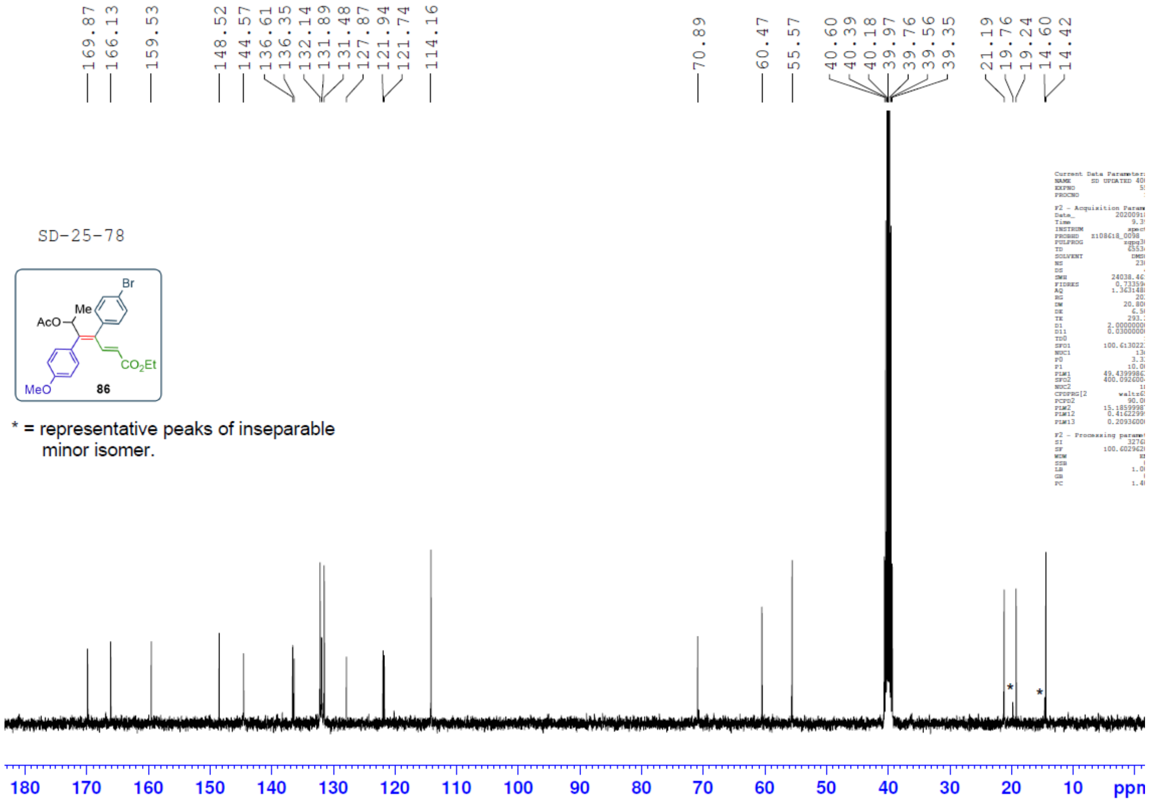

Supplementary Figure 213. ^{13}C NMR of compound **86**

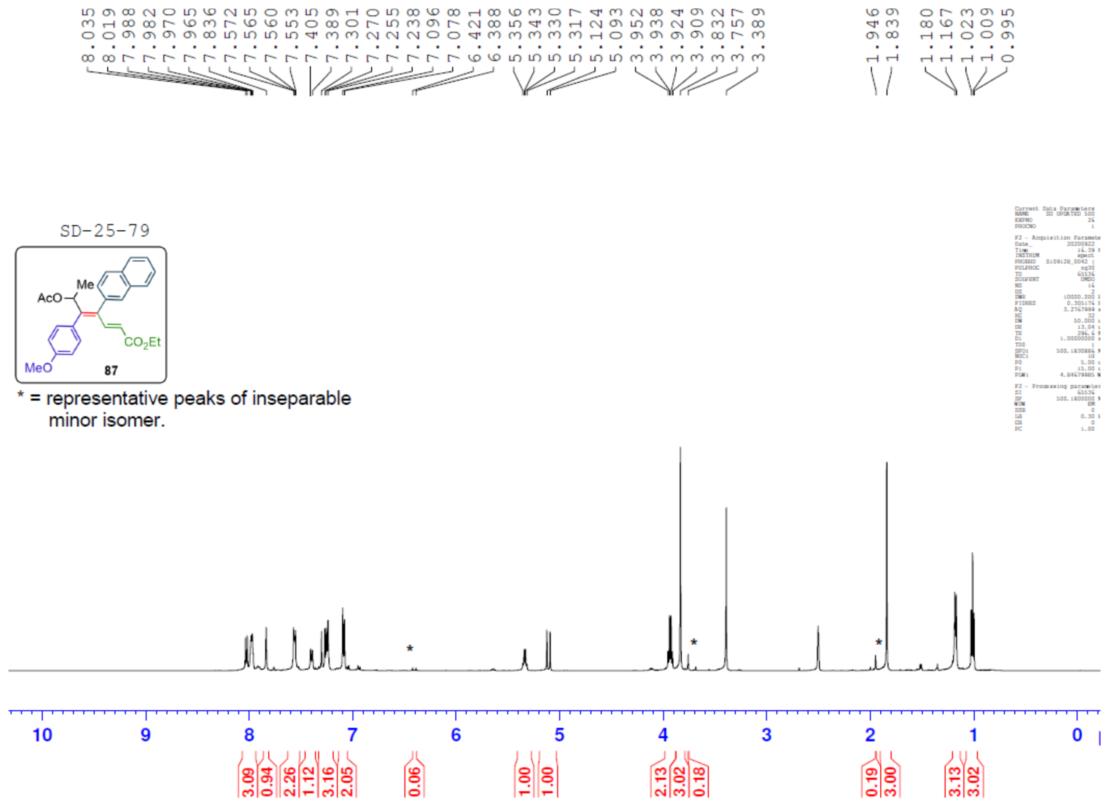

Supplementary Figure 214. ^{1}H NMR of compound **87**

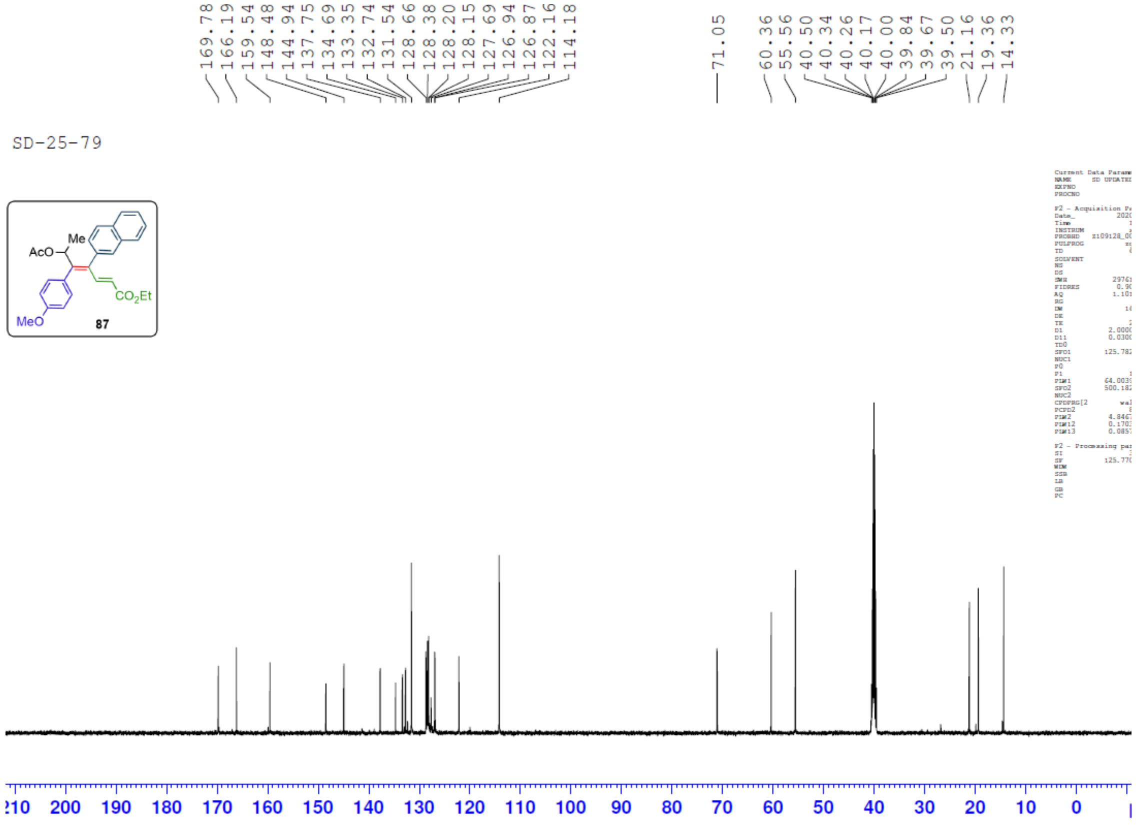

Supplementary Figure 215. ^{13}C NMR of compound **87**

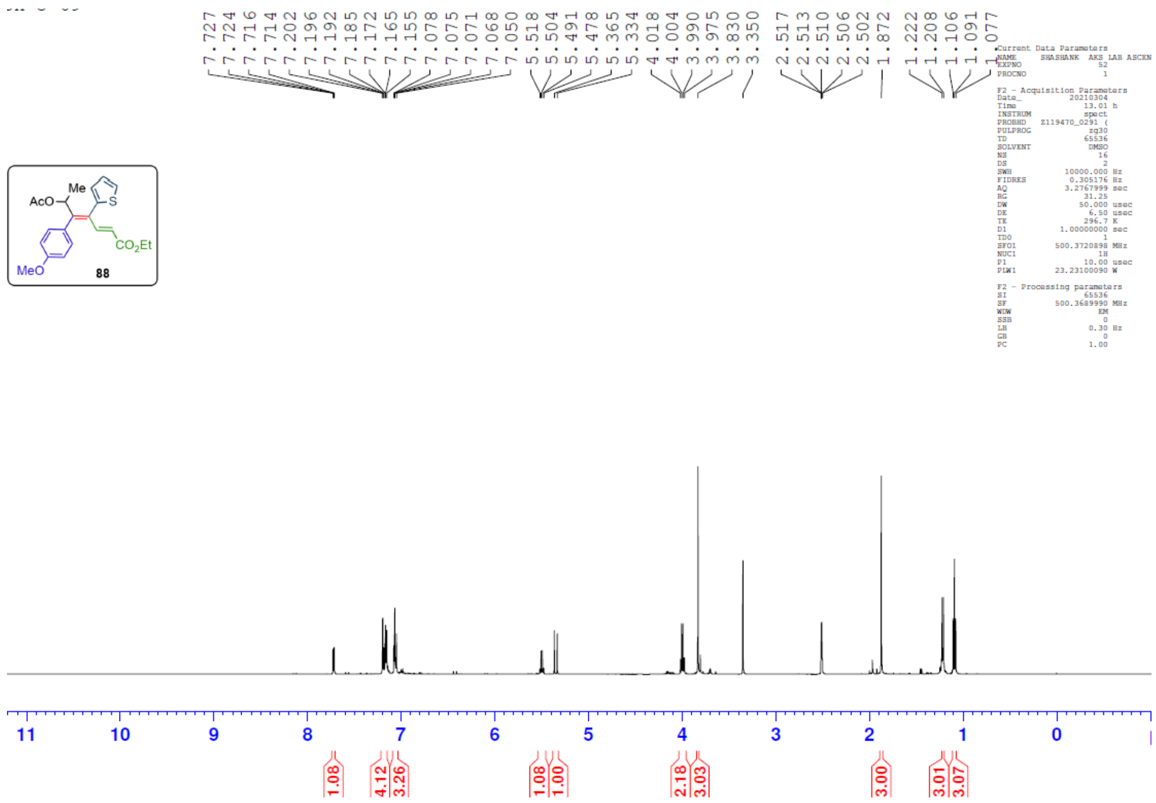

Supplementary Figure 216. ^{1}H NMR of compound **88**

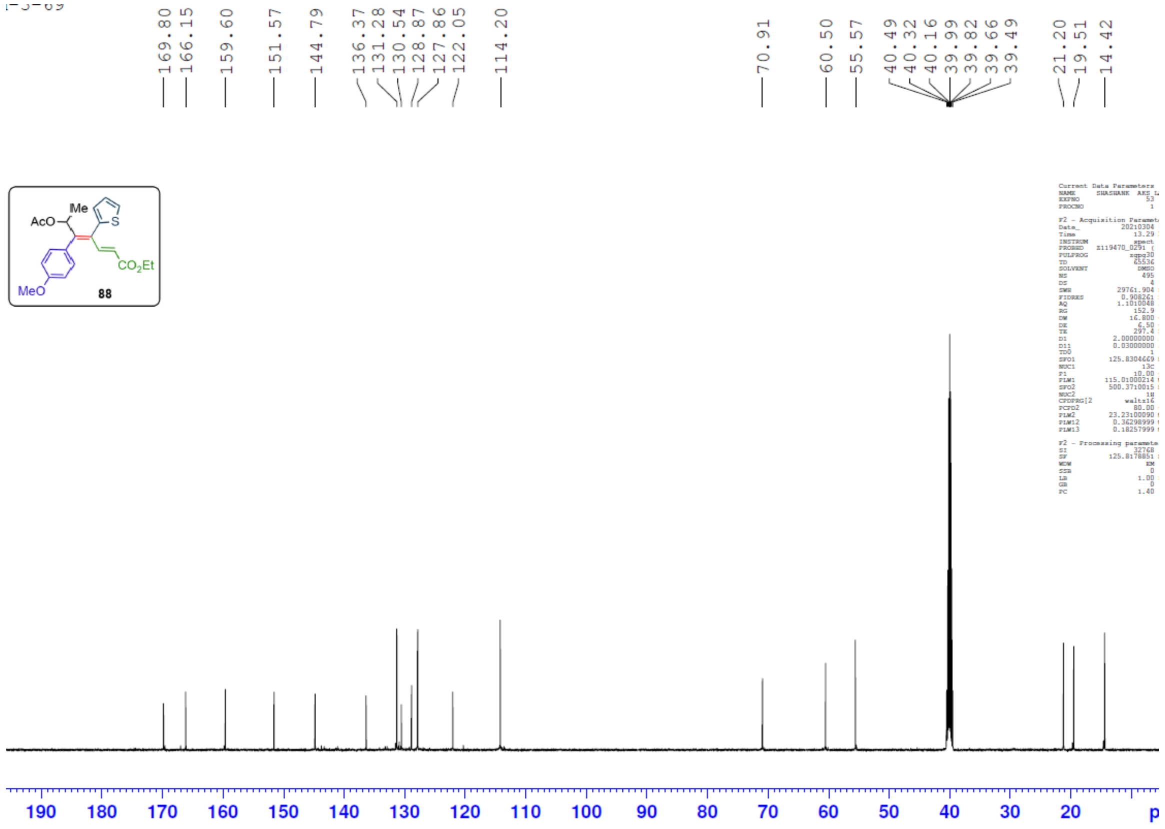

Supplementary Figure 217. ^{13}C NMR of compound **88**

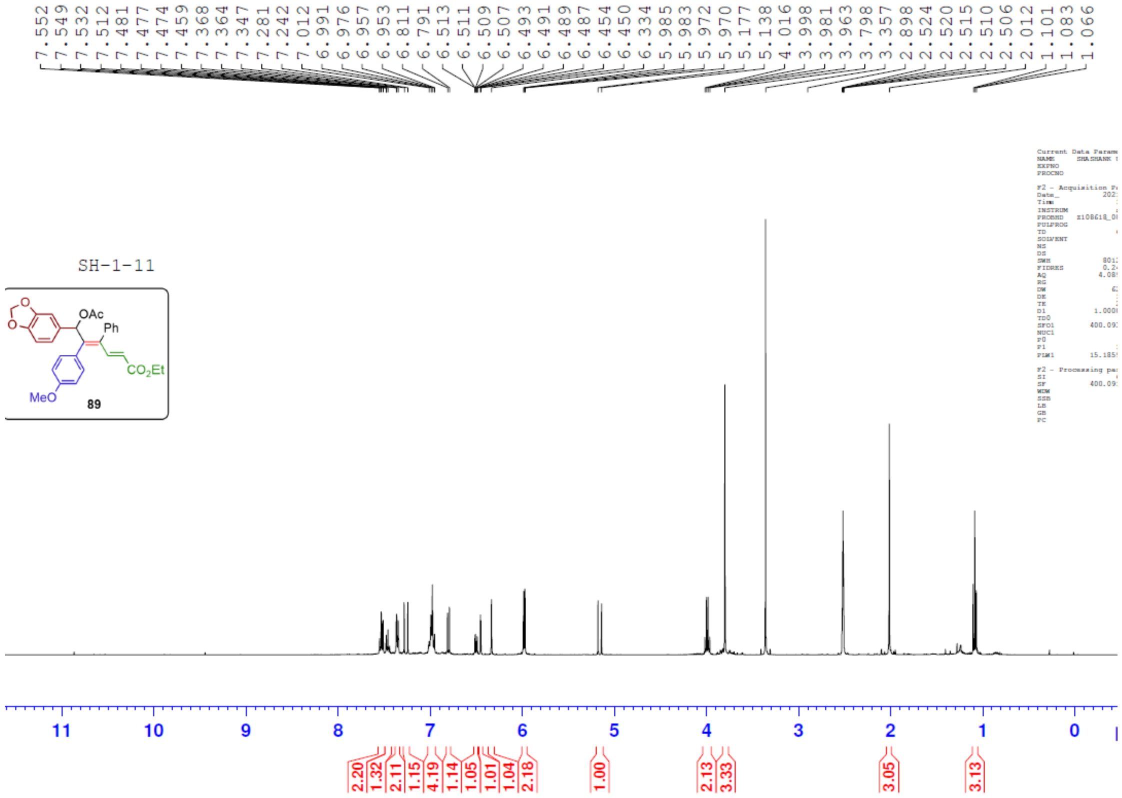

Supplementary Figure 218. ^{1}H NMR of compound **89**

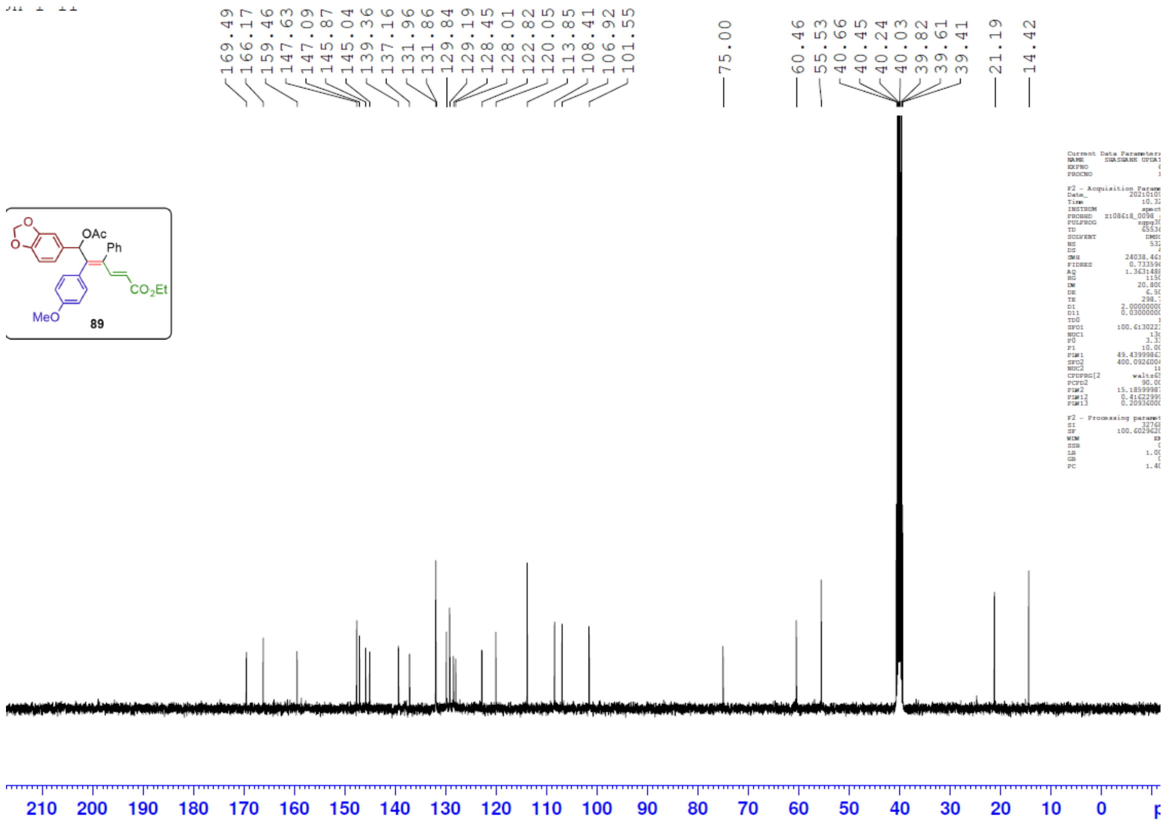

Supplementary Figure 219. ^{13}C NMR of compound **89**

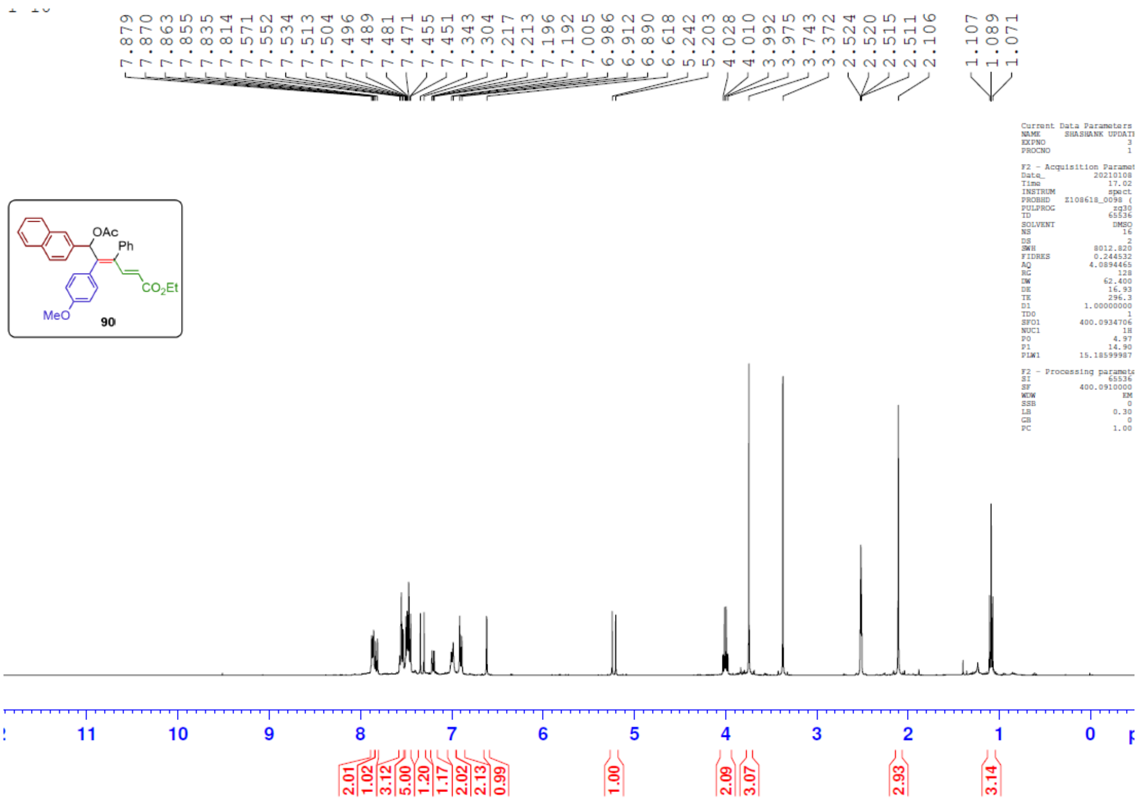

Supplementary Figure 220. ^{1}H NMR of compound **90**

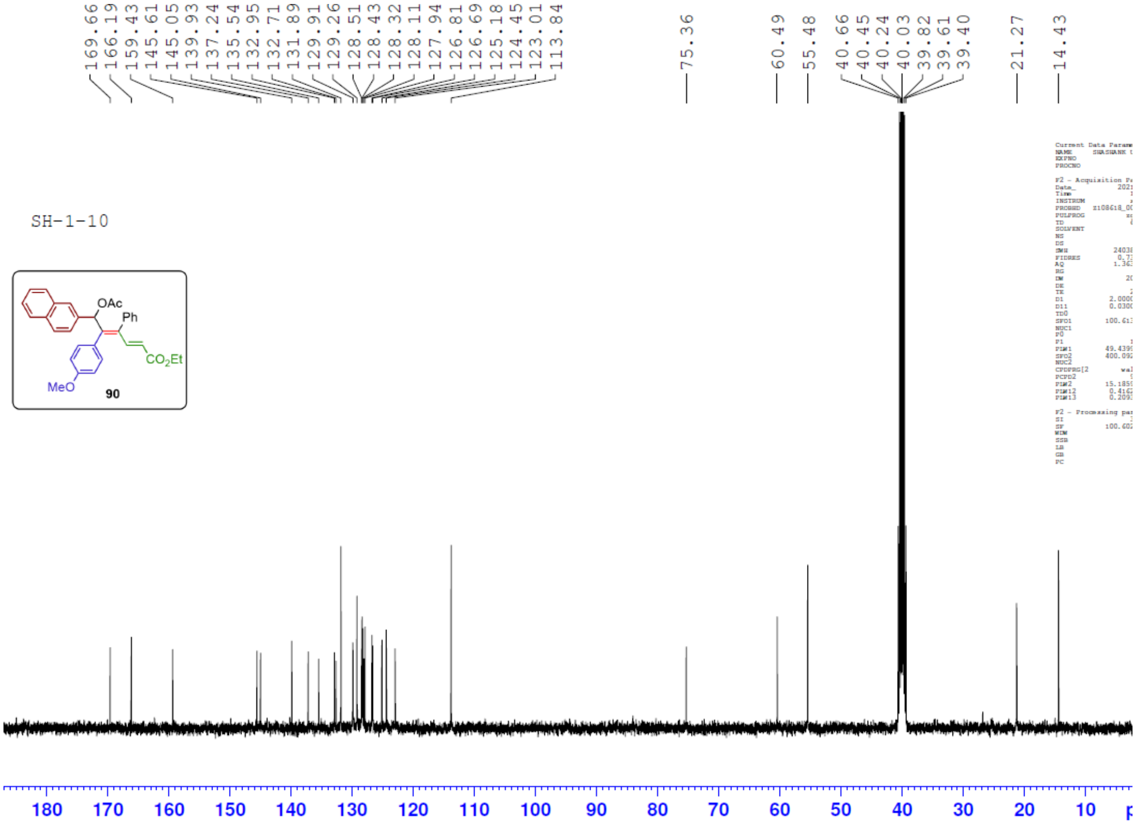

Supplementary Figure 221. ^{13}C NMR of compound **90**

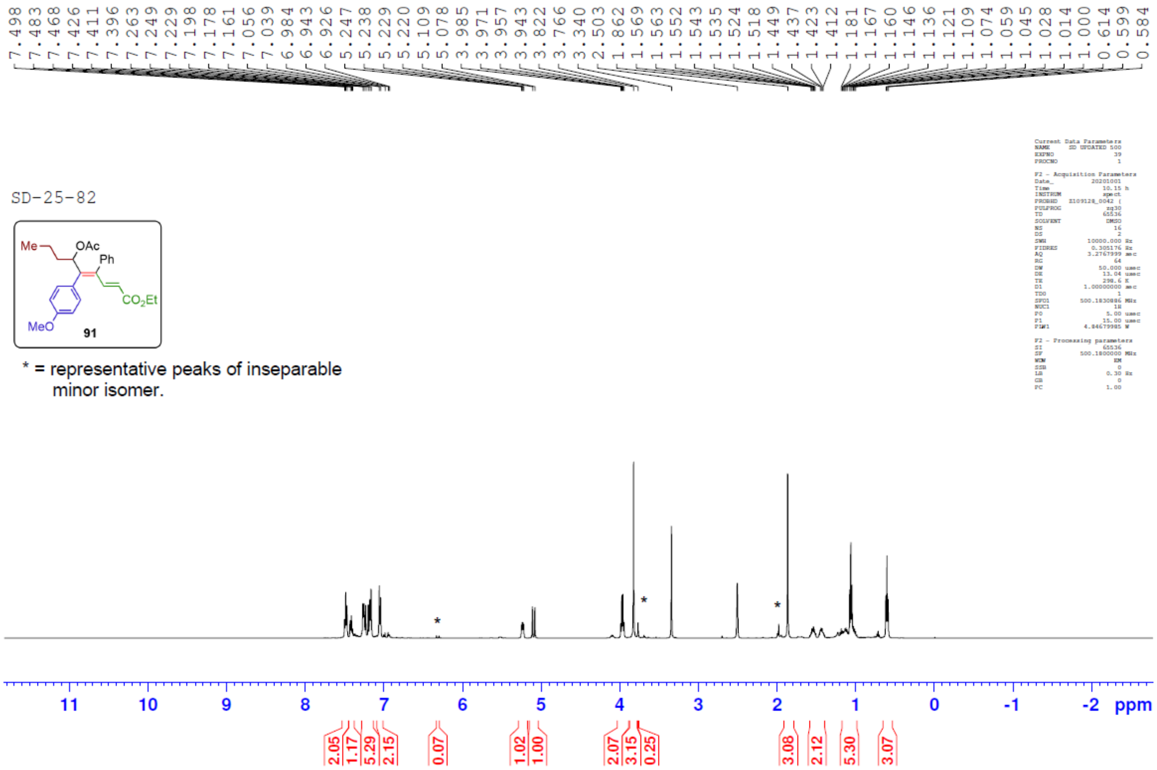

Supplementary Figure 222. ^{1}H NMR of compound **91**

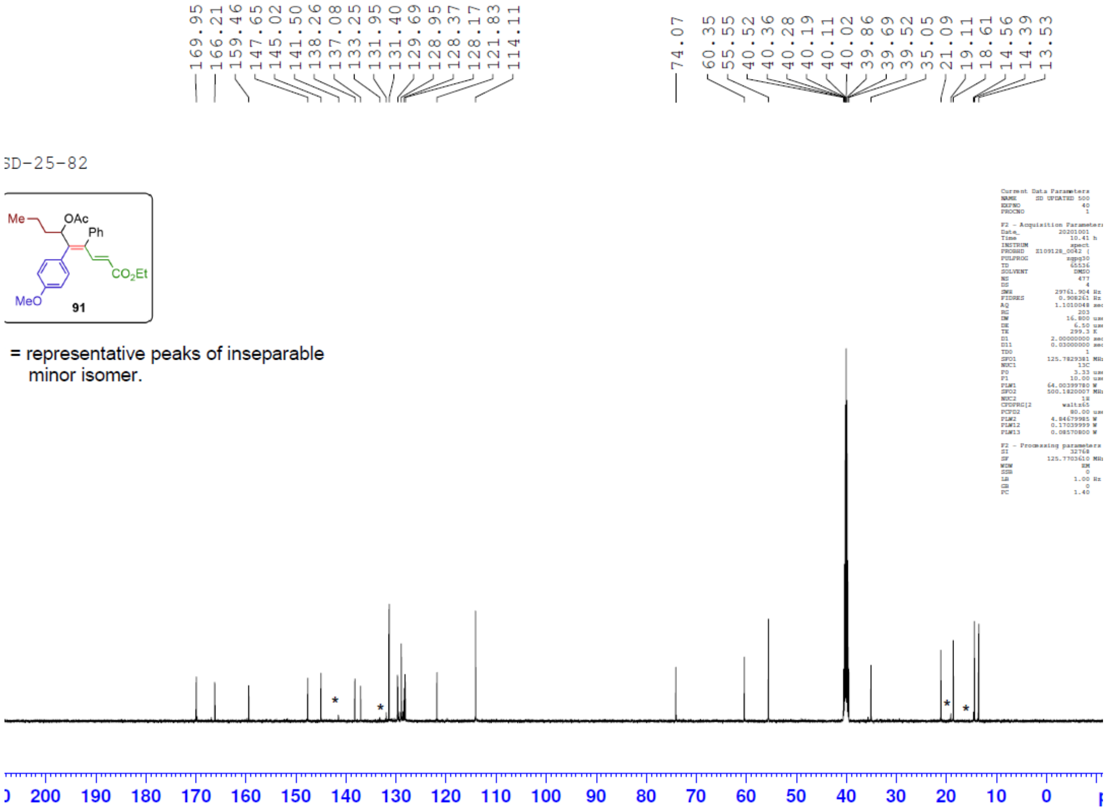

Supplementary Figure 223. ^{13}C NMR of compound **91**

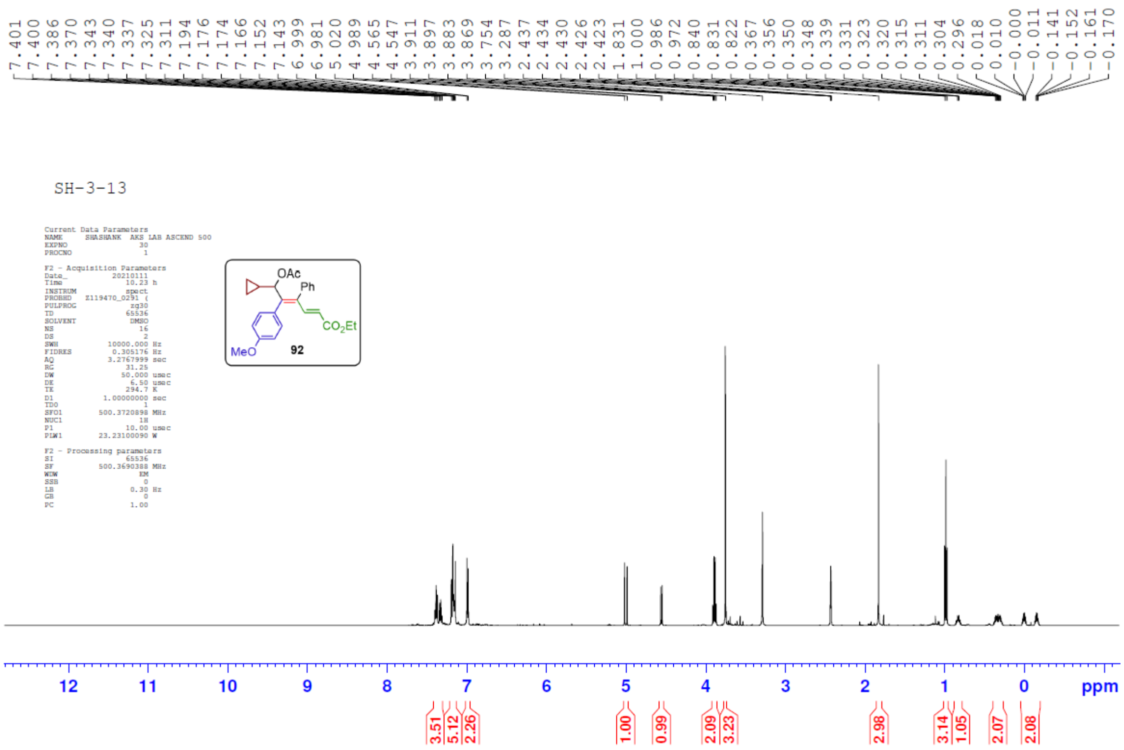

Supplementary Figure 224. ^{1}H NMR of compound **92**

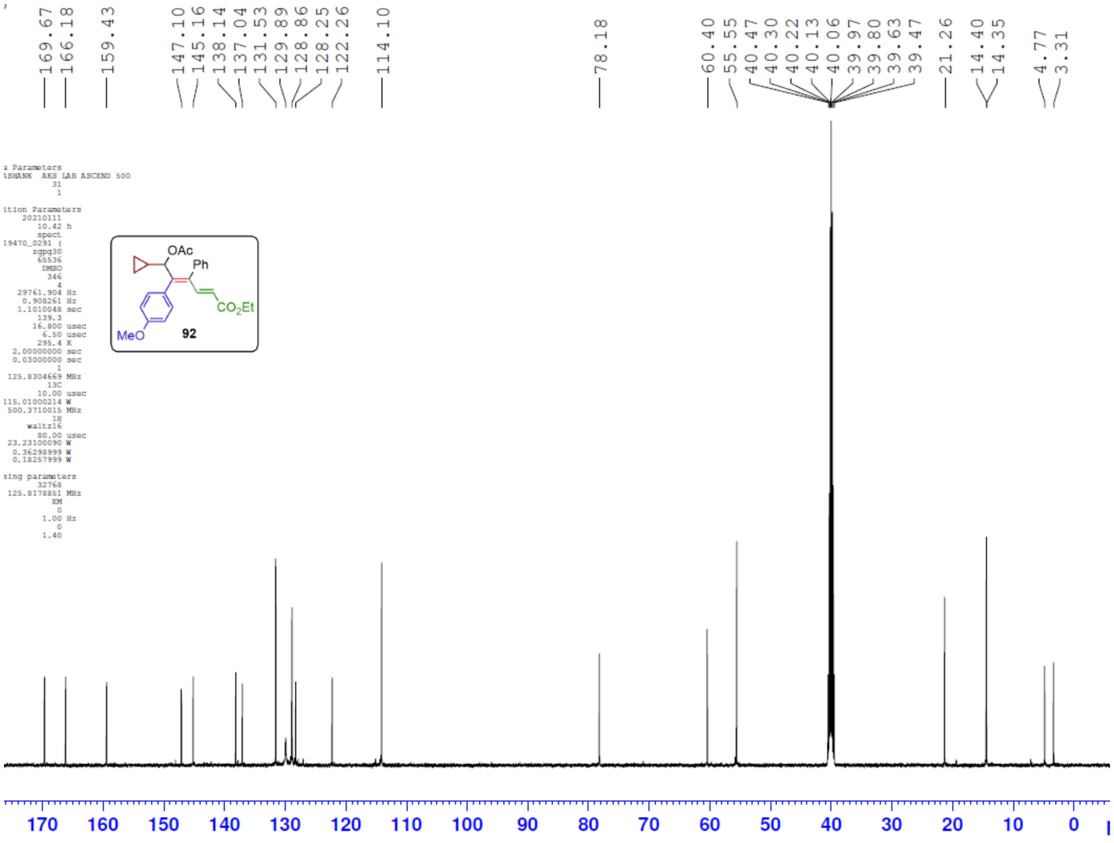

Supplementary Figure 225. ^{13}C NMR of compound **92**

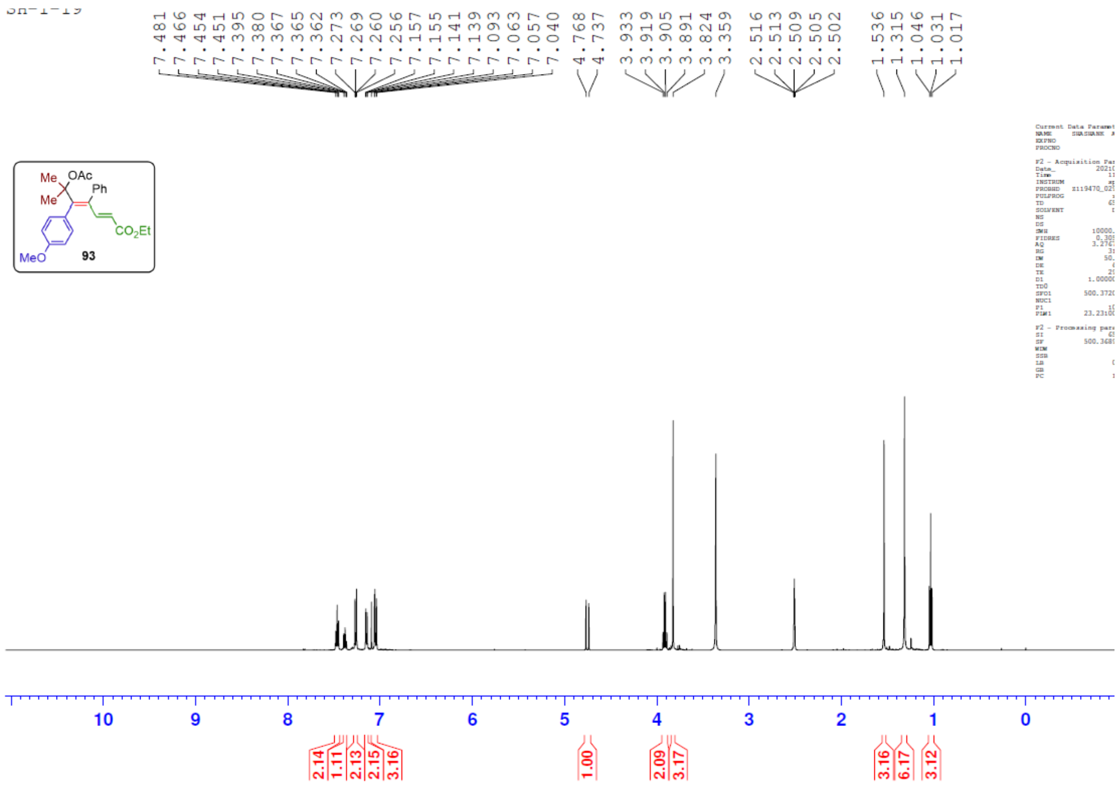

Supplementary Figure 226. ^{1}H NMR of compound **93**

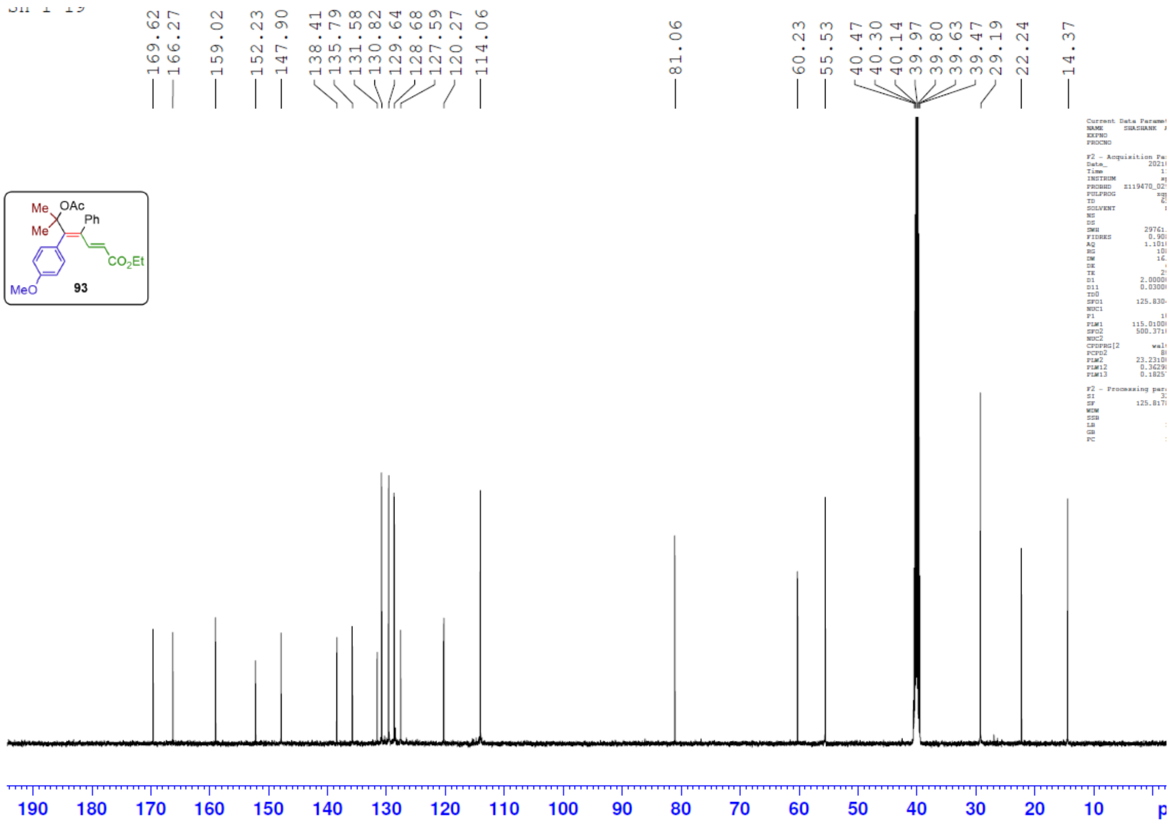

Supplementary Figure 227. ^{13}C NMR of compound **93**

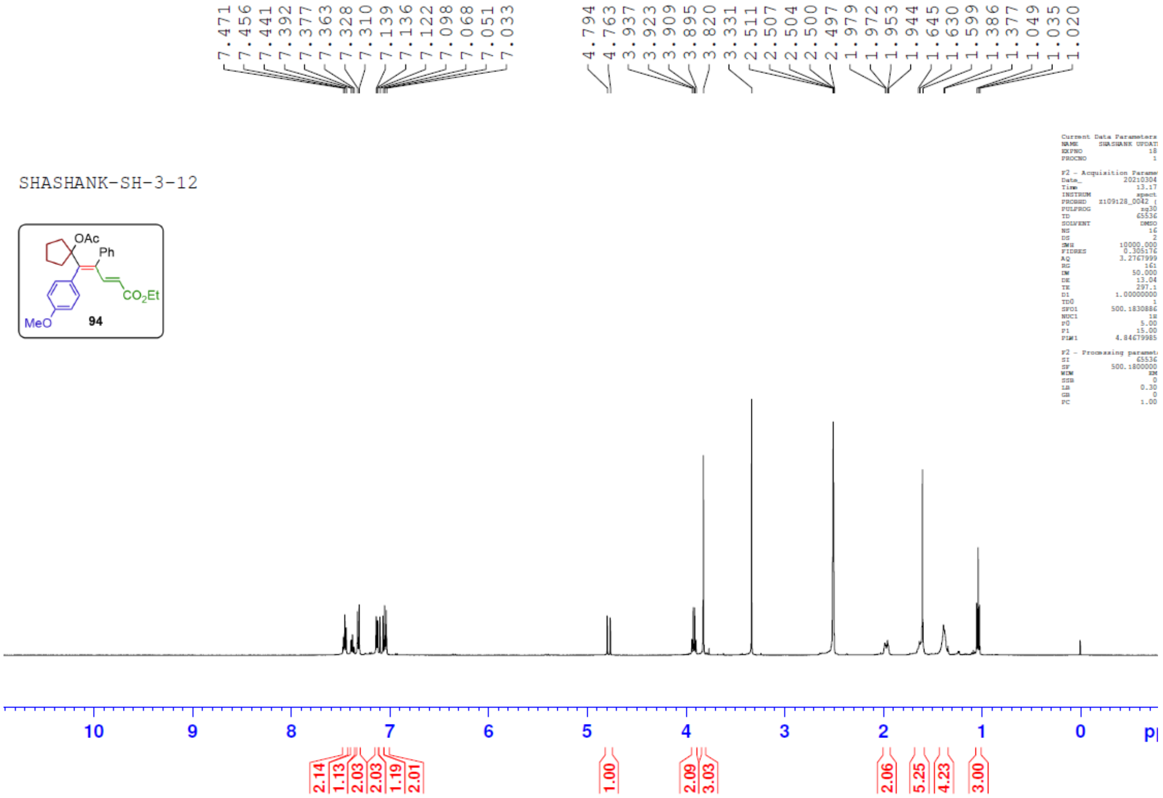

Supplementary Figure 228. ^{1}H NMR of compound **94**

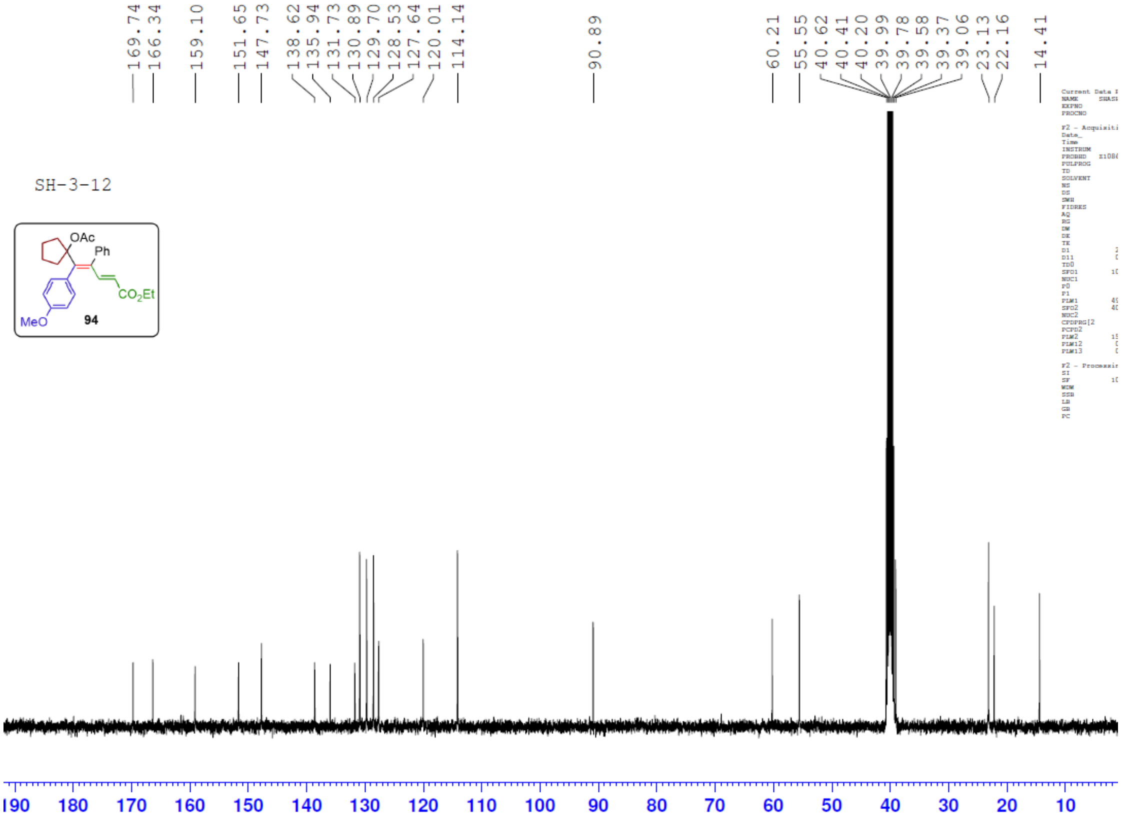

Supplementary Figure 229. ^{13}C NMR of compound **94**

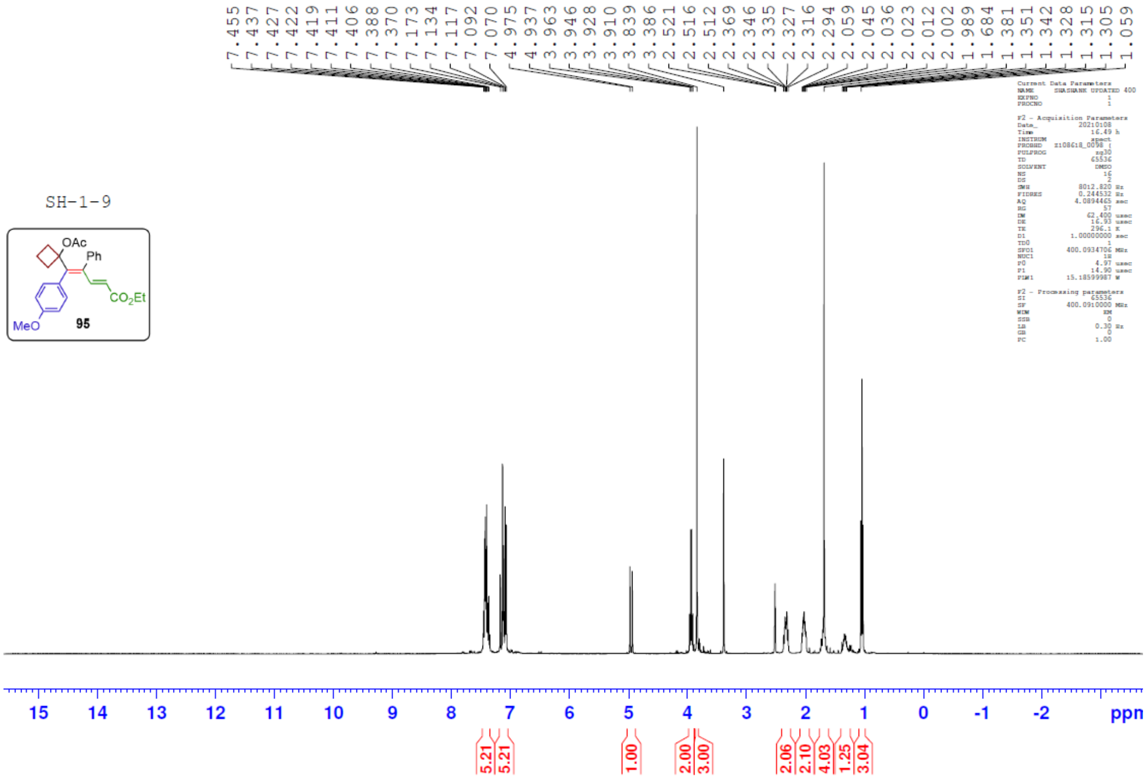

Supplementary Figure 230. ^{1}H NMR of compound **95**

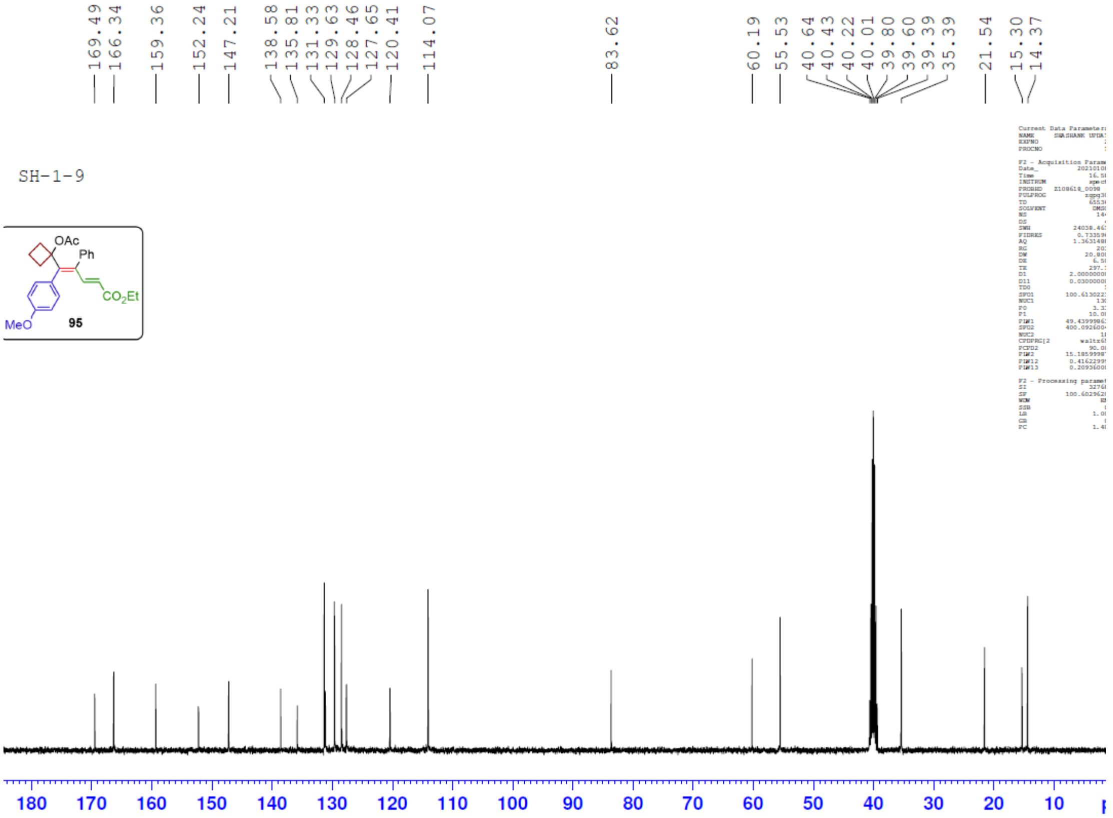

Supplementary Figure 231. ^{13}C NMR of compound **95**

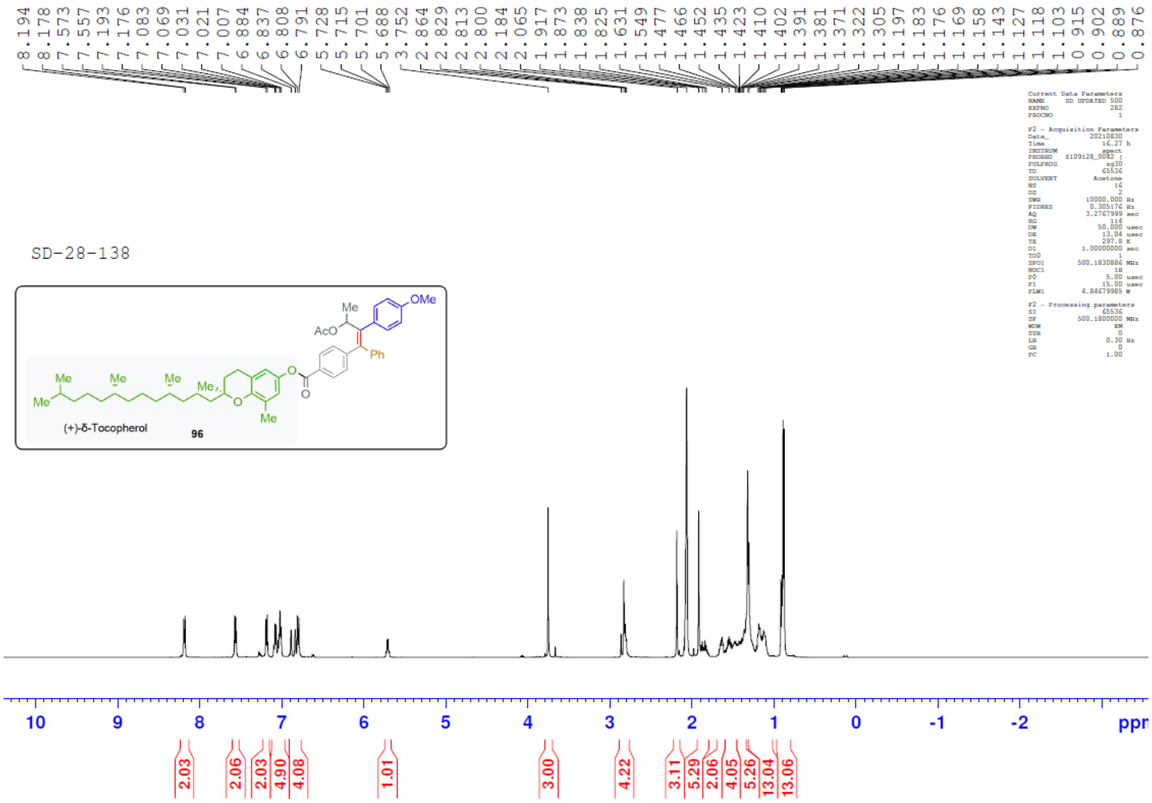

Supplementary Figure 232. ^{1}H NMR of compound **96**

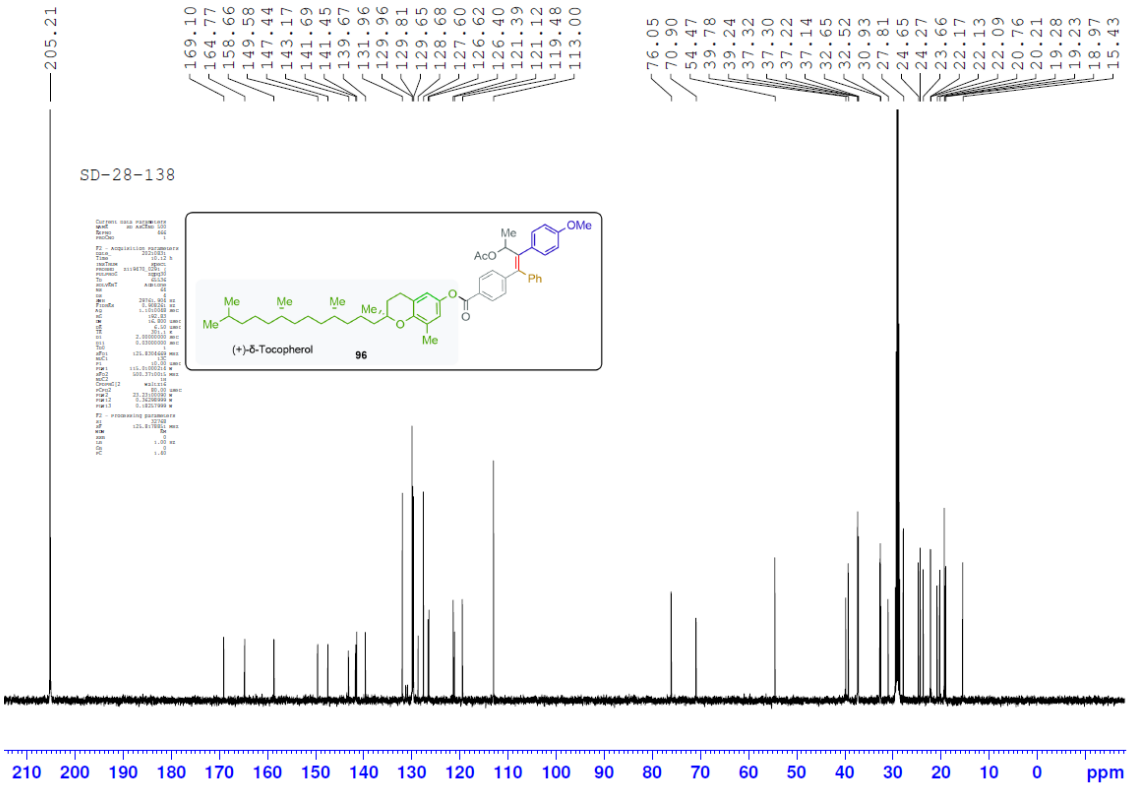

Supplementary Figure 233. ^{13}C NMR of compound **96**

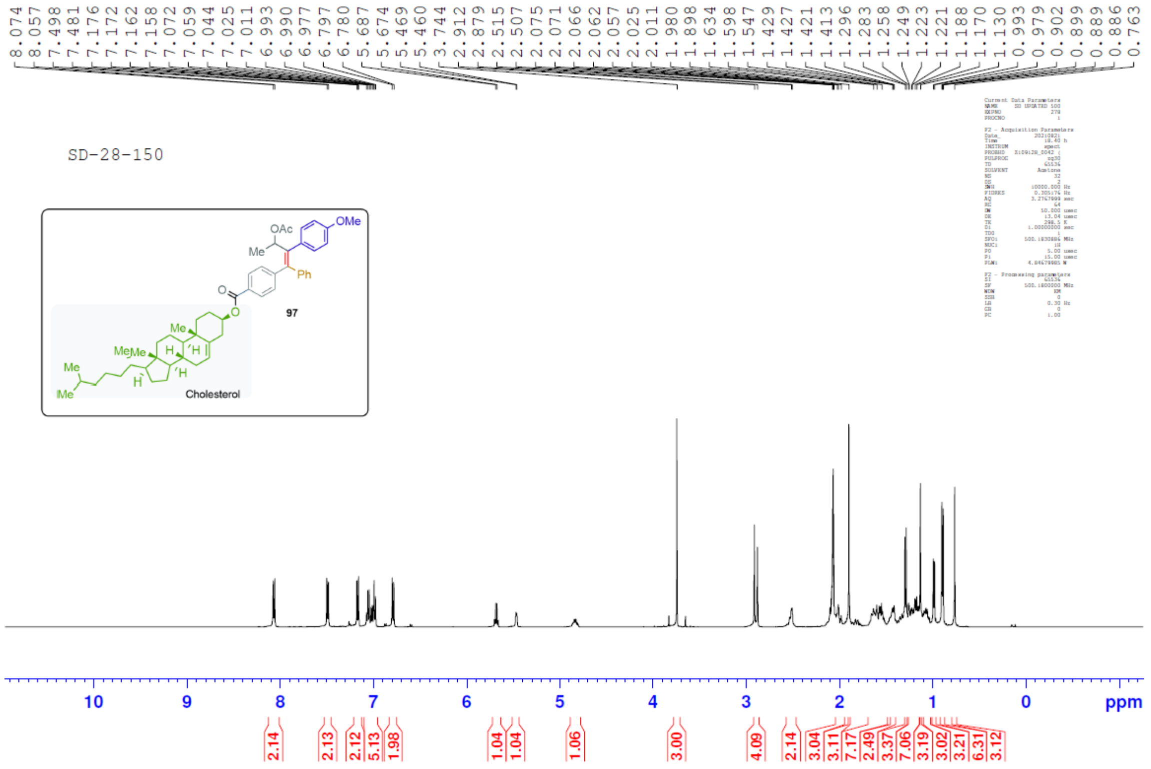

Supplementary Figure 234. ^{1}H NMR of compound **97**

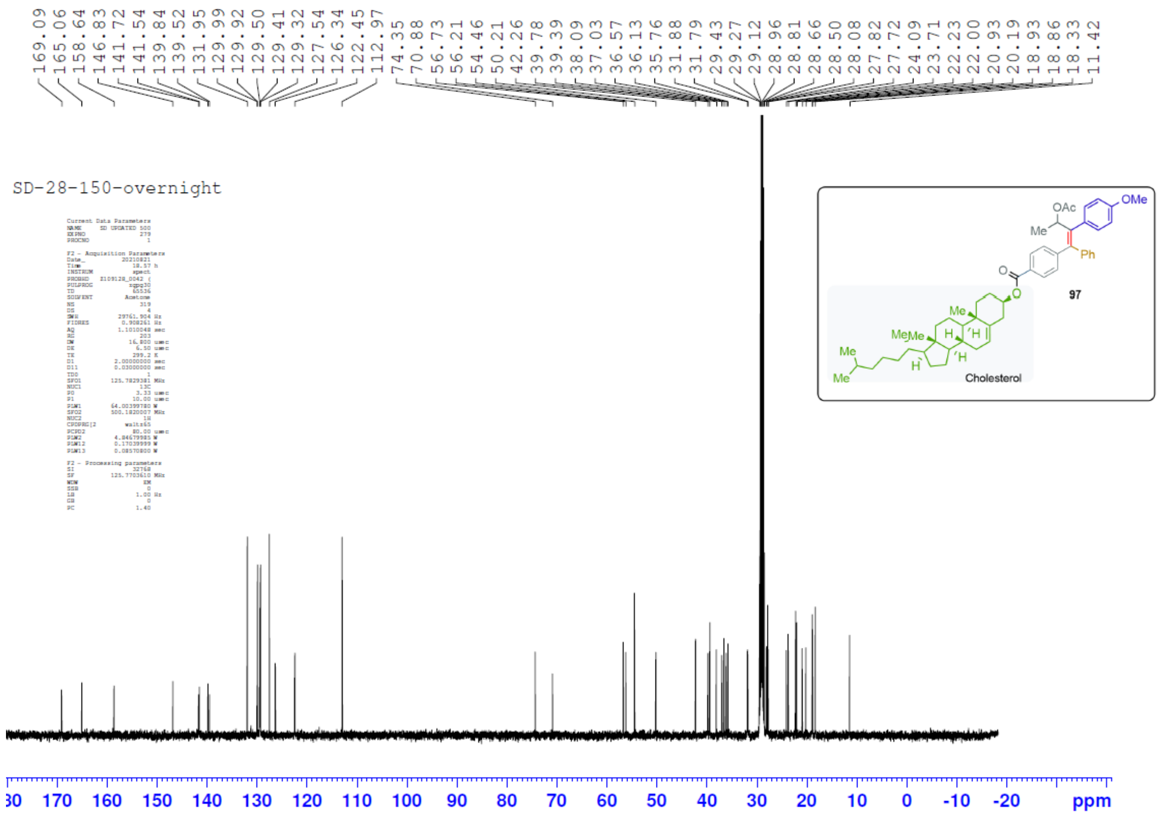

Supplementary Figure 235. ^{13}C NMR of compound **97**

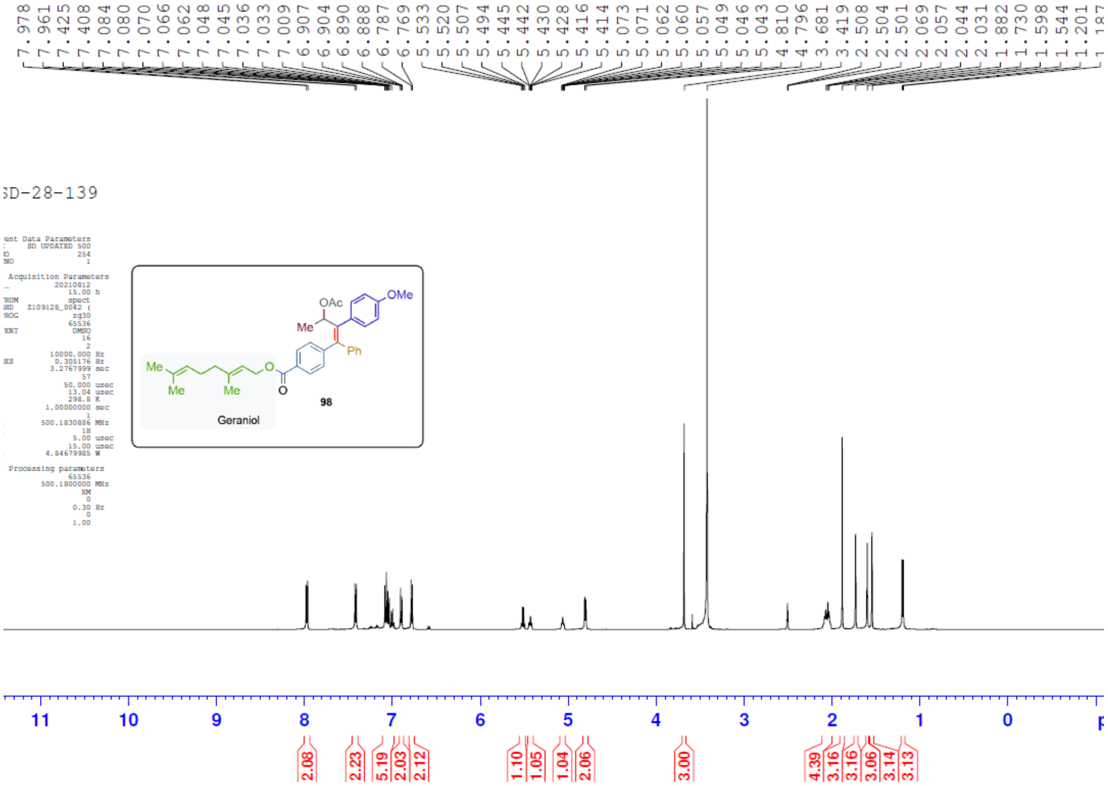

Supplementary Figure 236. ^{1}H NMR of compound **98**

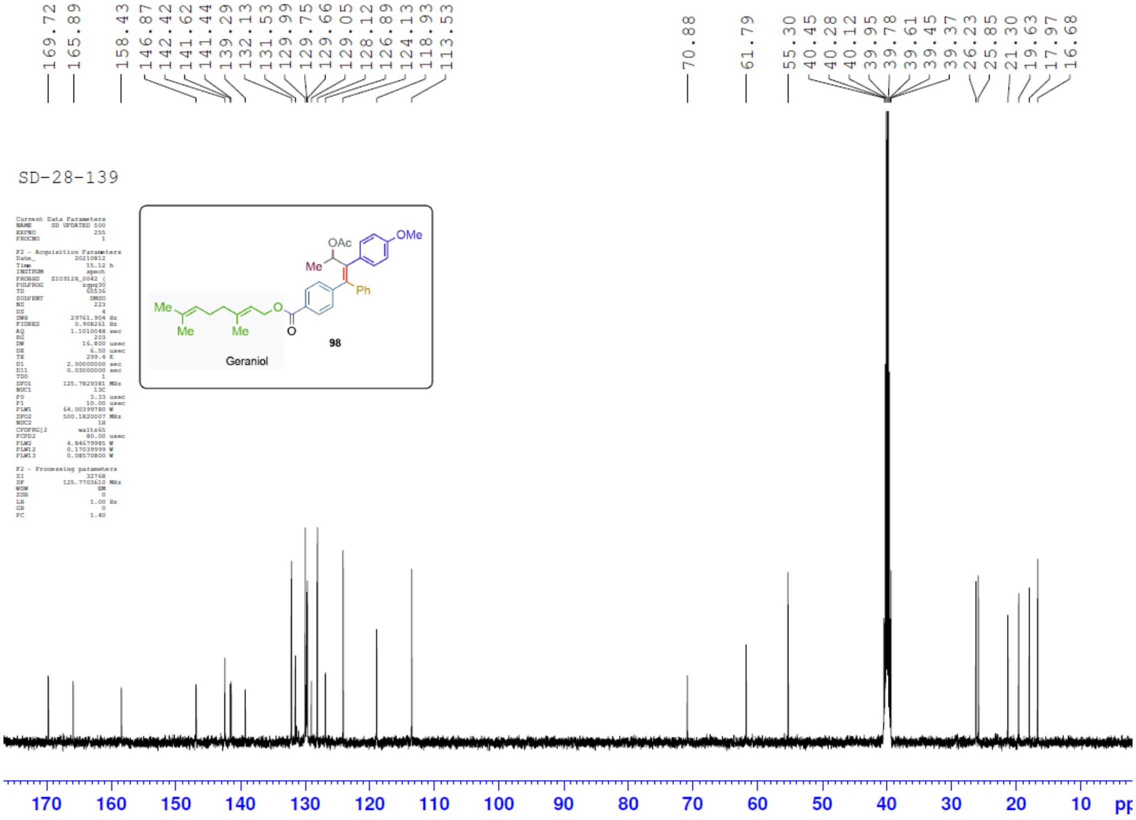

Supplementary Figure 237. ^{13}C NMR of compound **98**

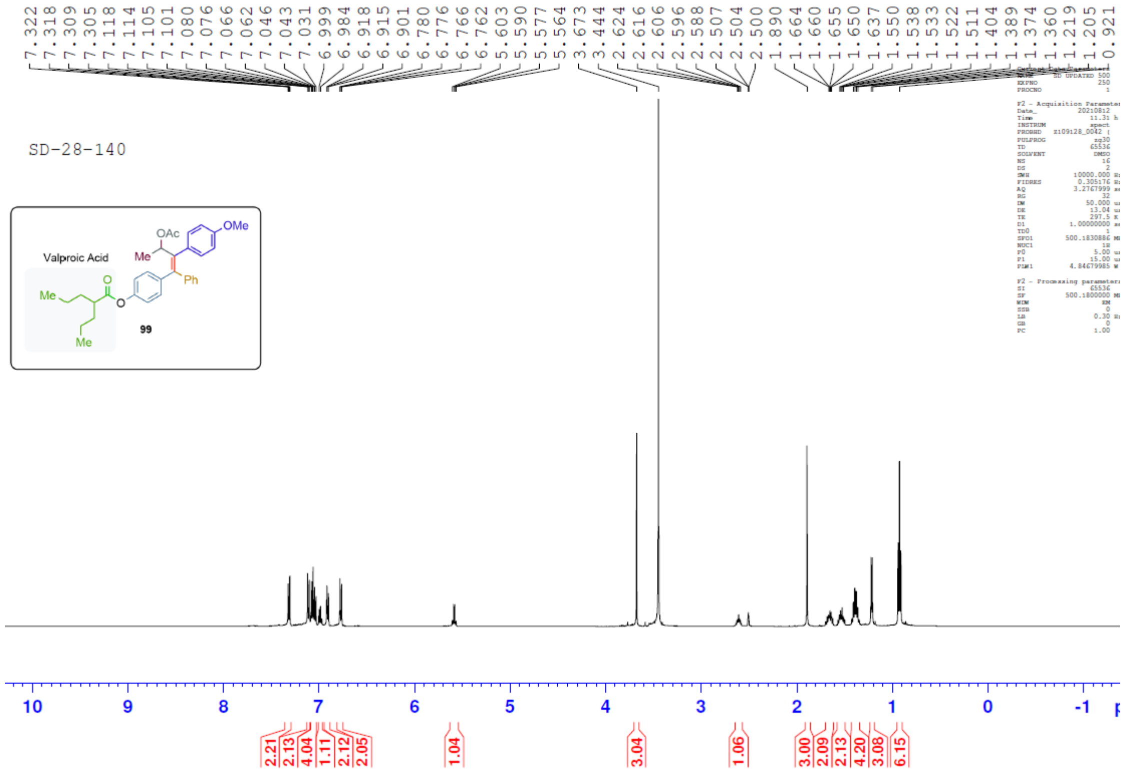

Supplementary Figure 238. ^{1}H NMR of compound **99**

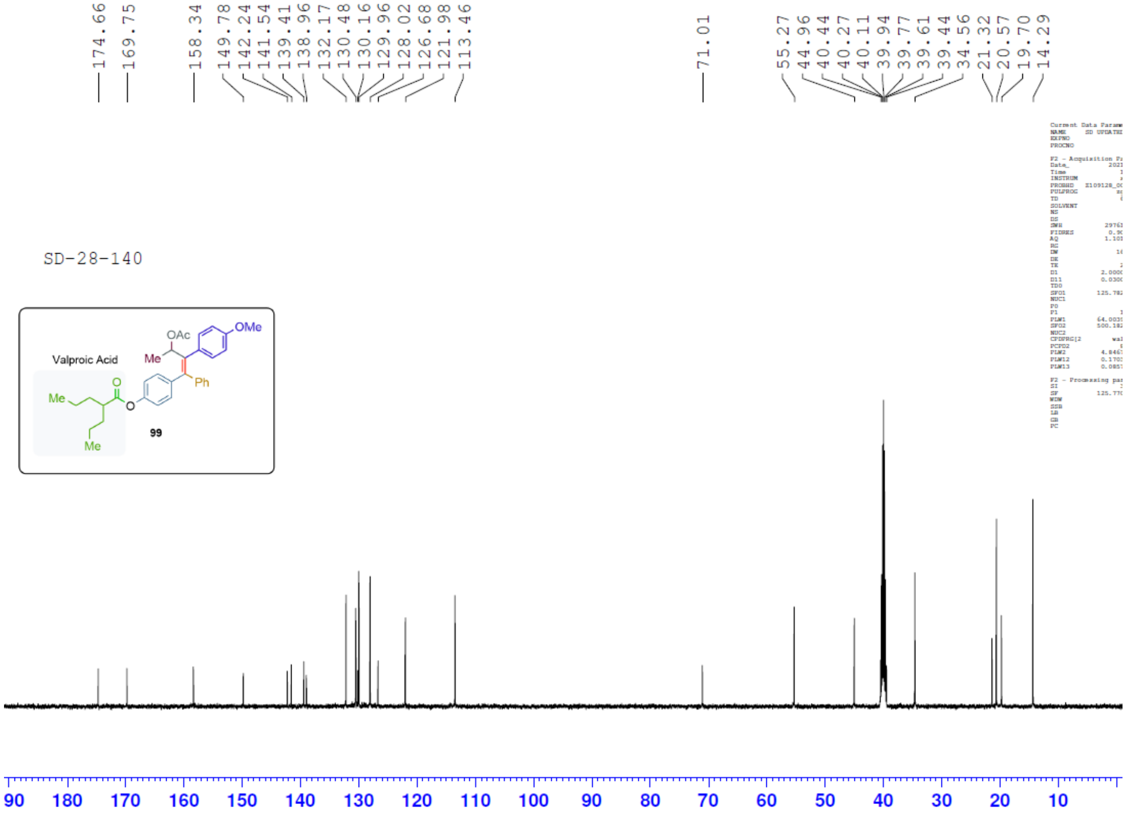

Supplementary Figure 239. ^{13}C NMR of compound **99**

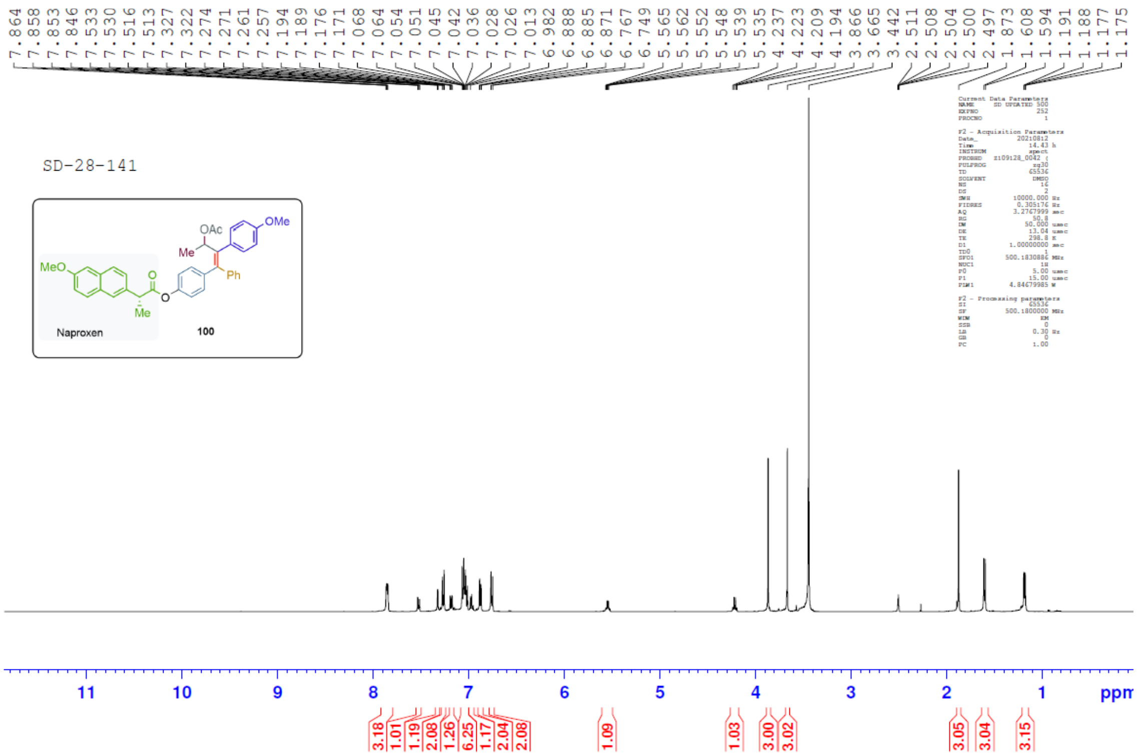

Supplementary Figure 240. ^{1}H NMR of compound **100**

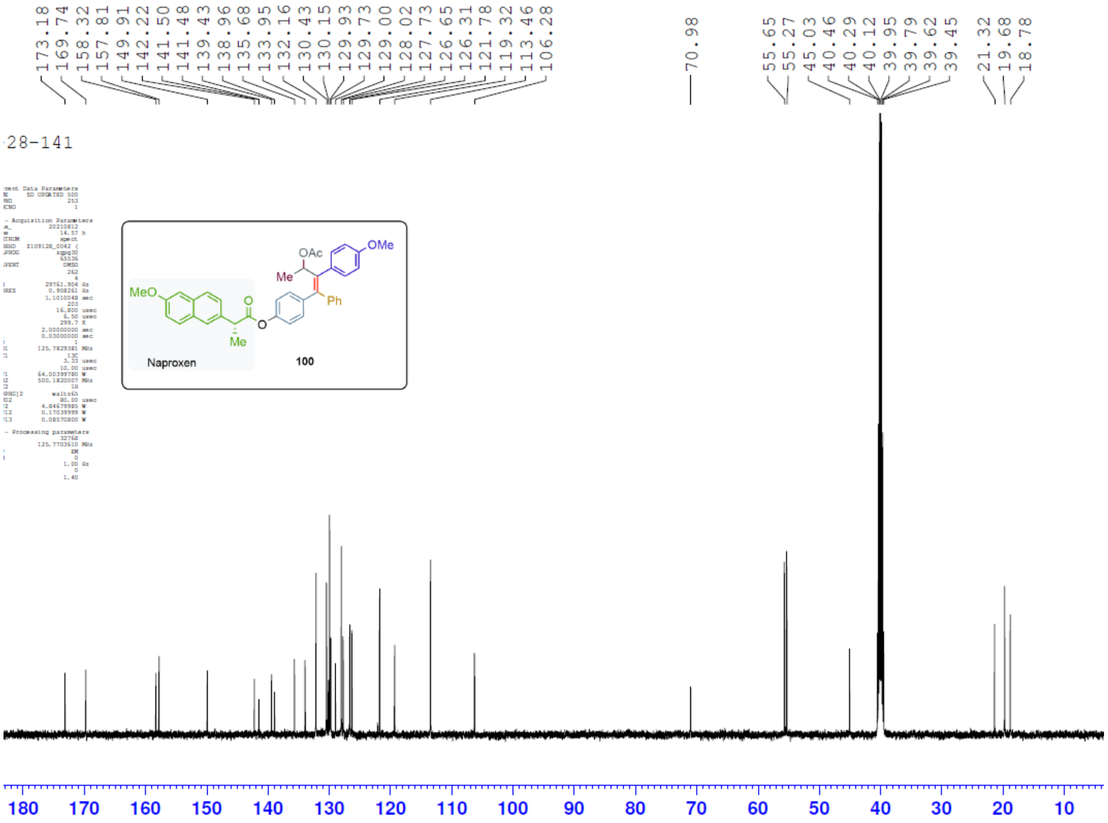

Supplementary Figure 241. ^{13}C NMR of compound **100**

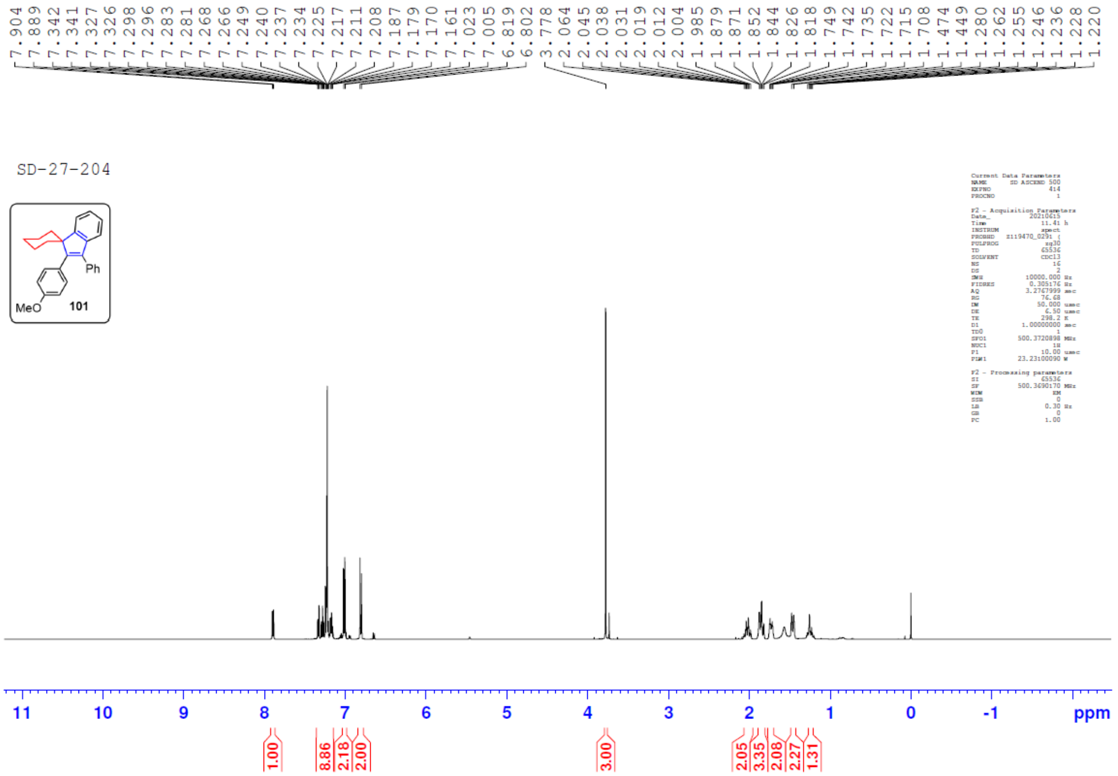

Supplementary Figure 242. ^{1}H NMR of compound **101**

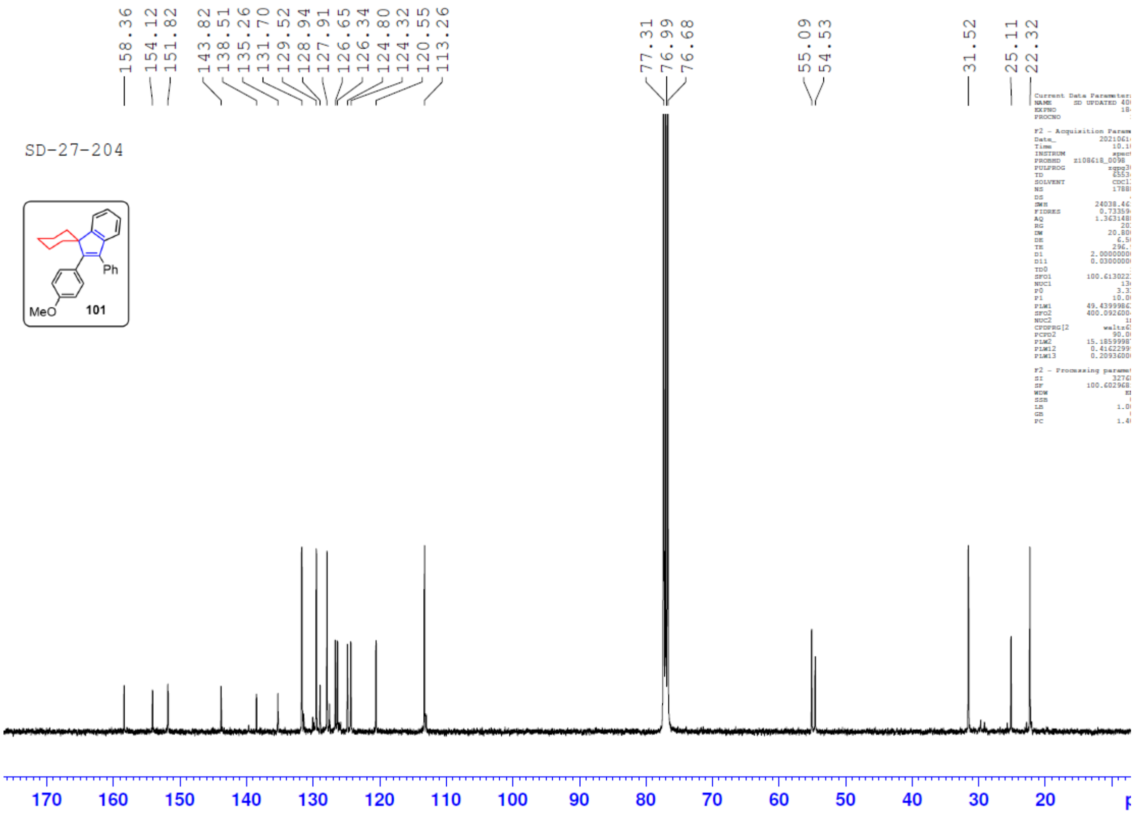

Supplementary Figure 243. ^{13}C NMR of compound **101**

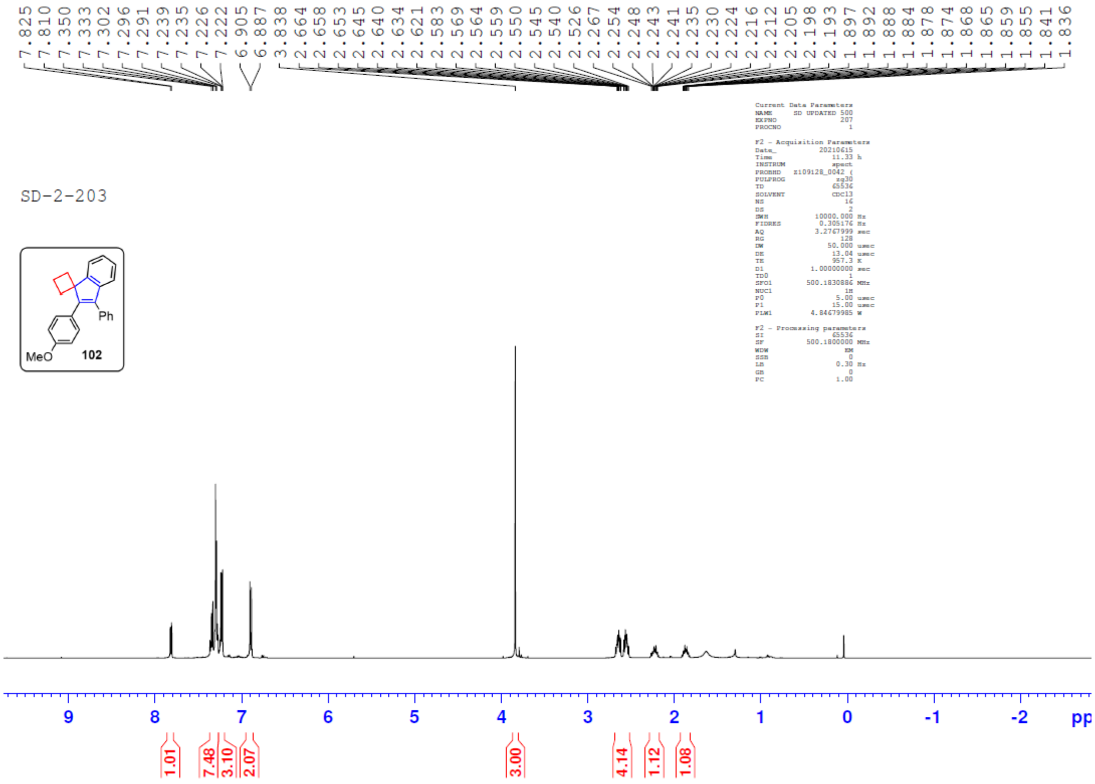

Supplementary Figure 244. ^{1}H NMR of compound **102**

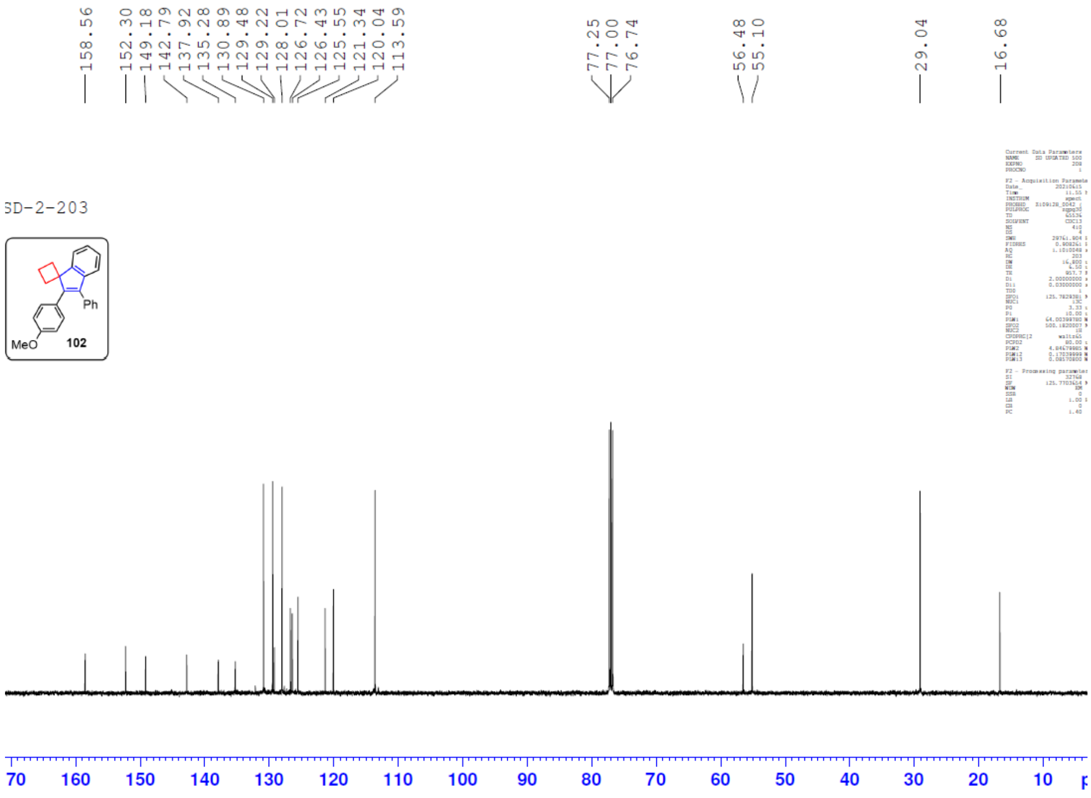

Supplementary Figure 245. ^{13}C NMR of compound **102**

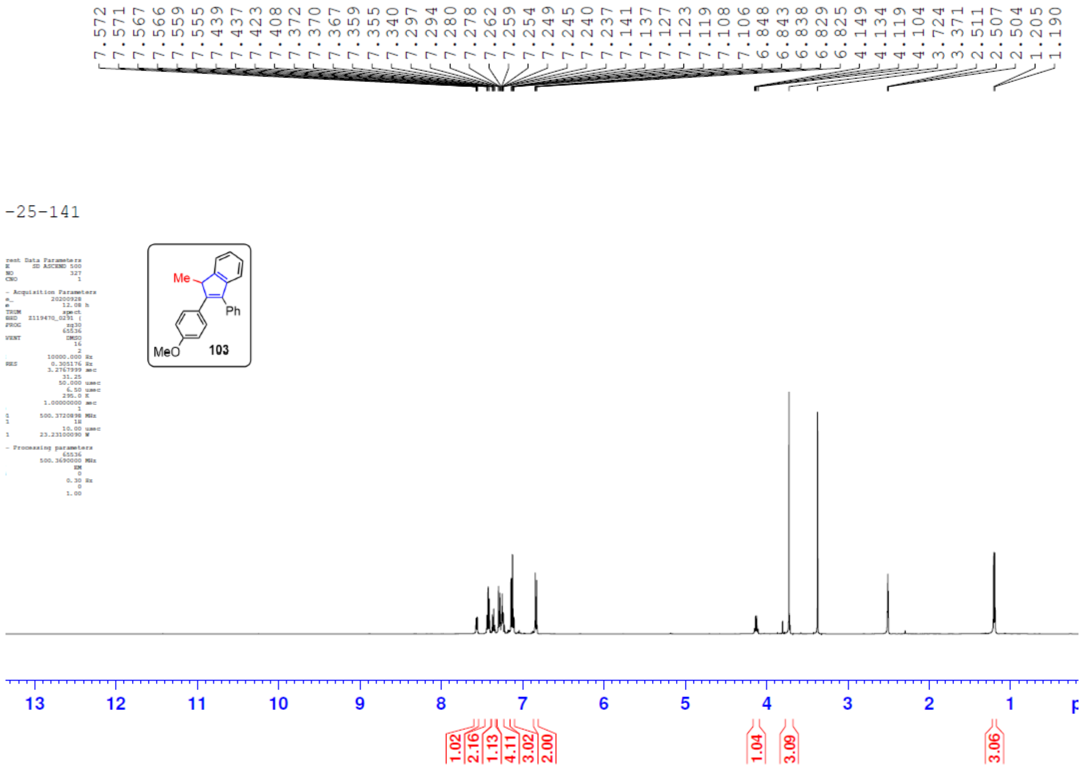

Supplementary Figure 246. ^{1}H NMR of compound **103**

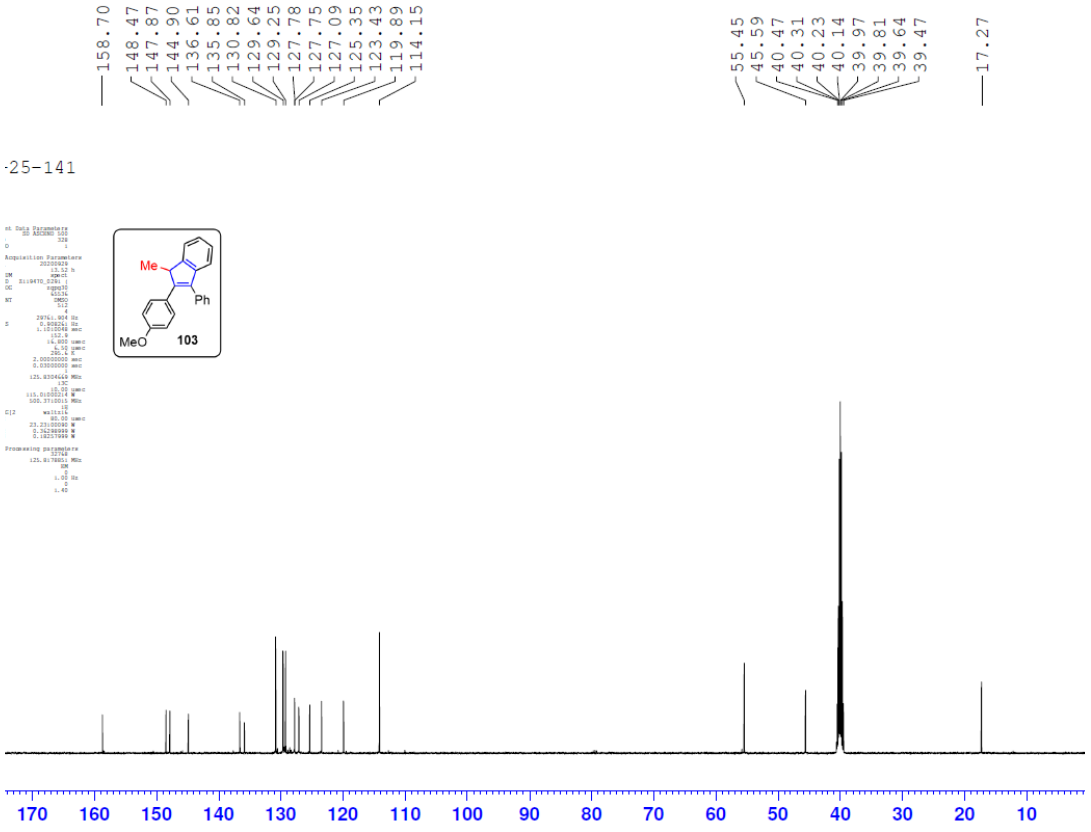

Supplementary Figure 247. ^{13}C NMR of compound **103**

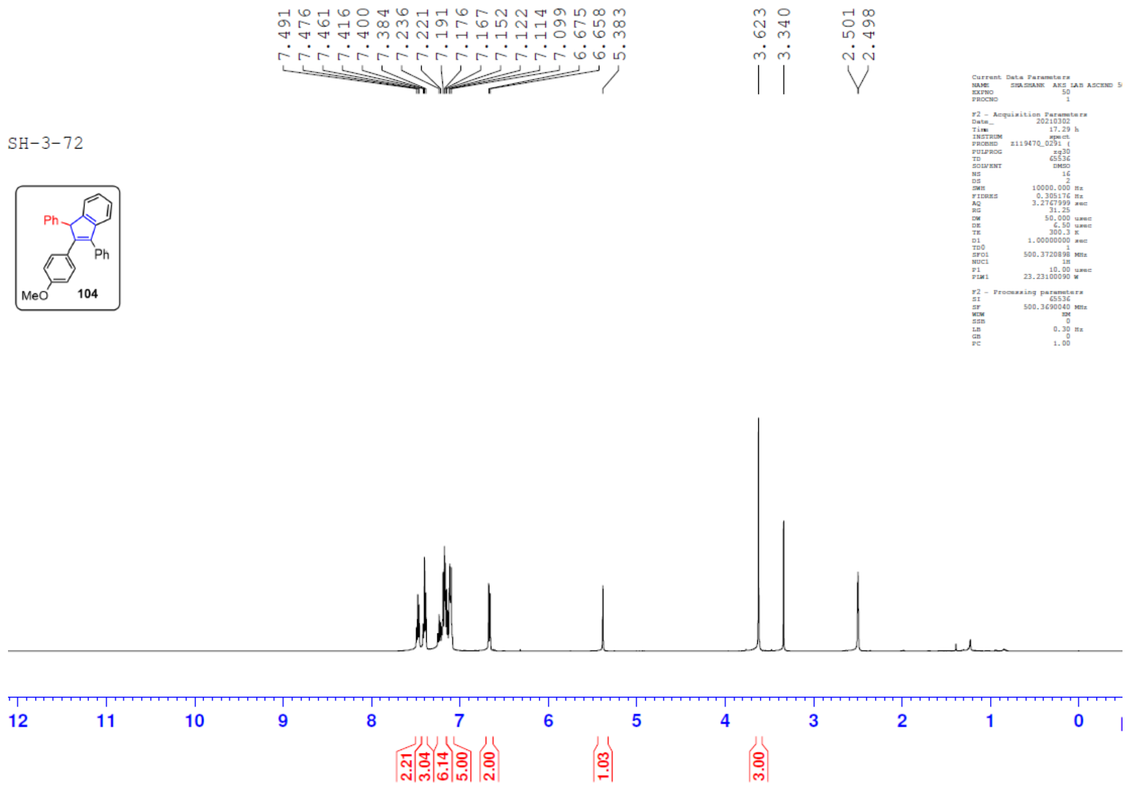

Supplementary Figure 248. ^{1}H NMR of compound **104**

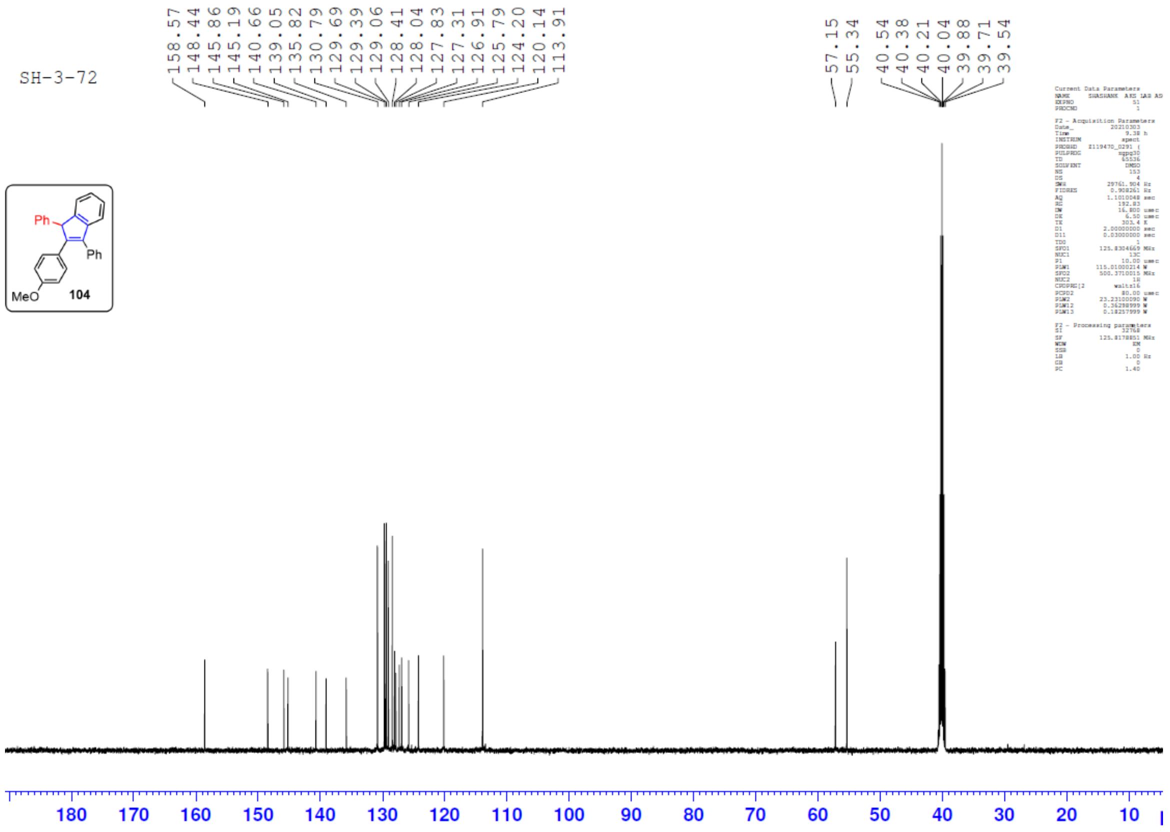

Supplementary Figure 249. ^{13}C NMR of compound **104**

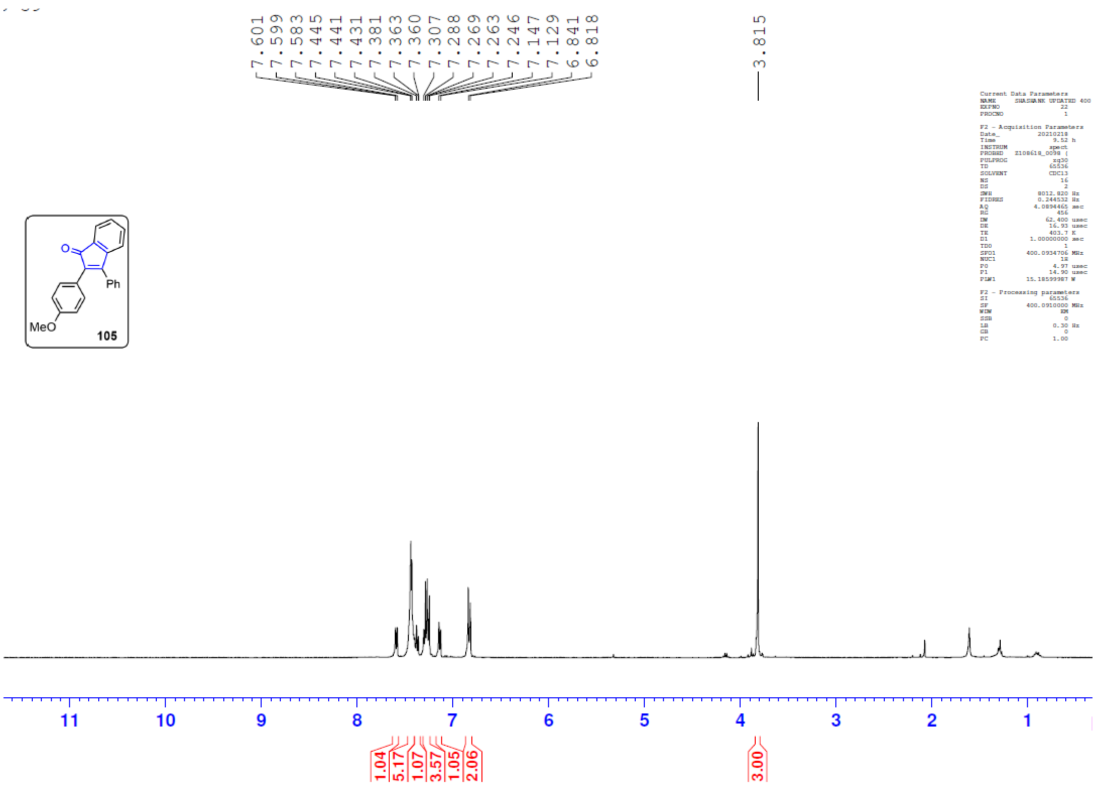

Supplementary Figure 250. ^{1}H NMR of compound **105**

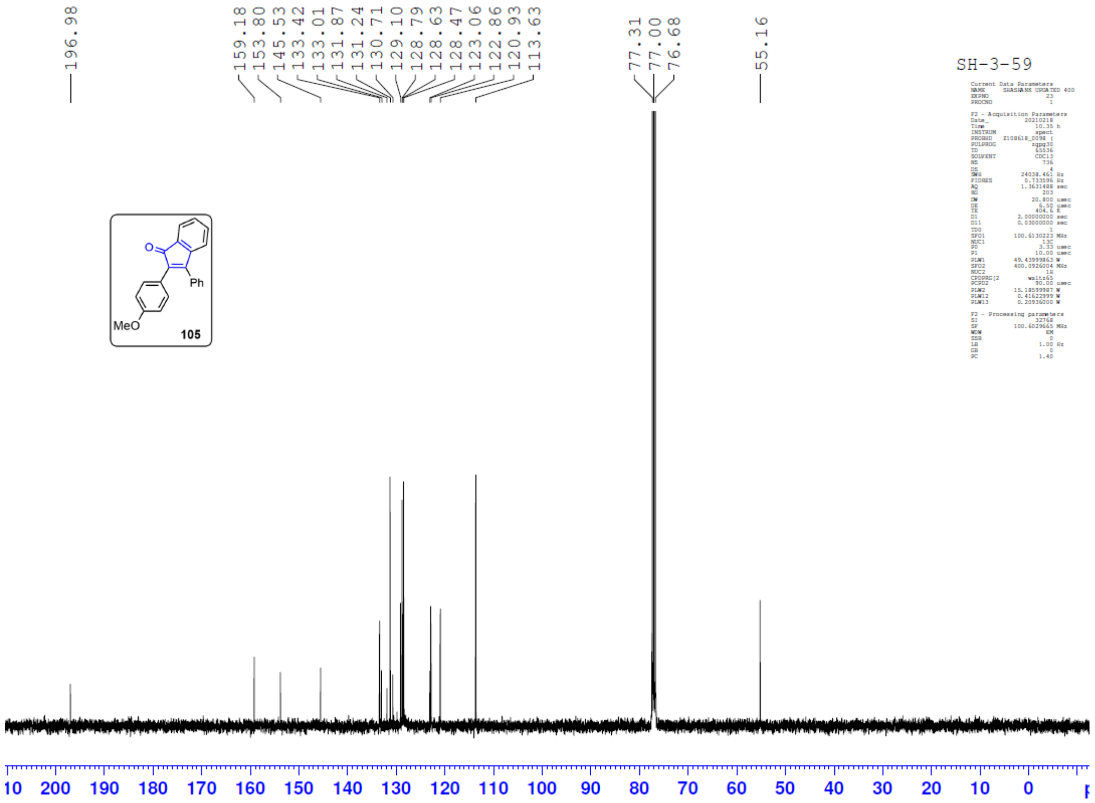

Supplementary Figure 251. ^{13}C NMR of compound **105**

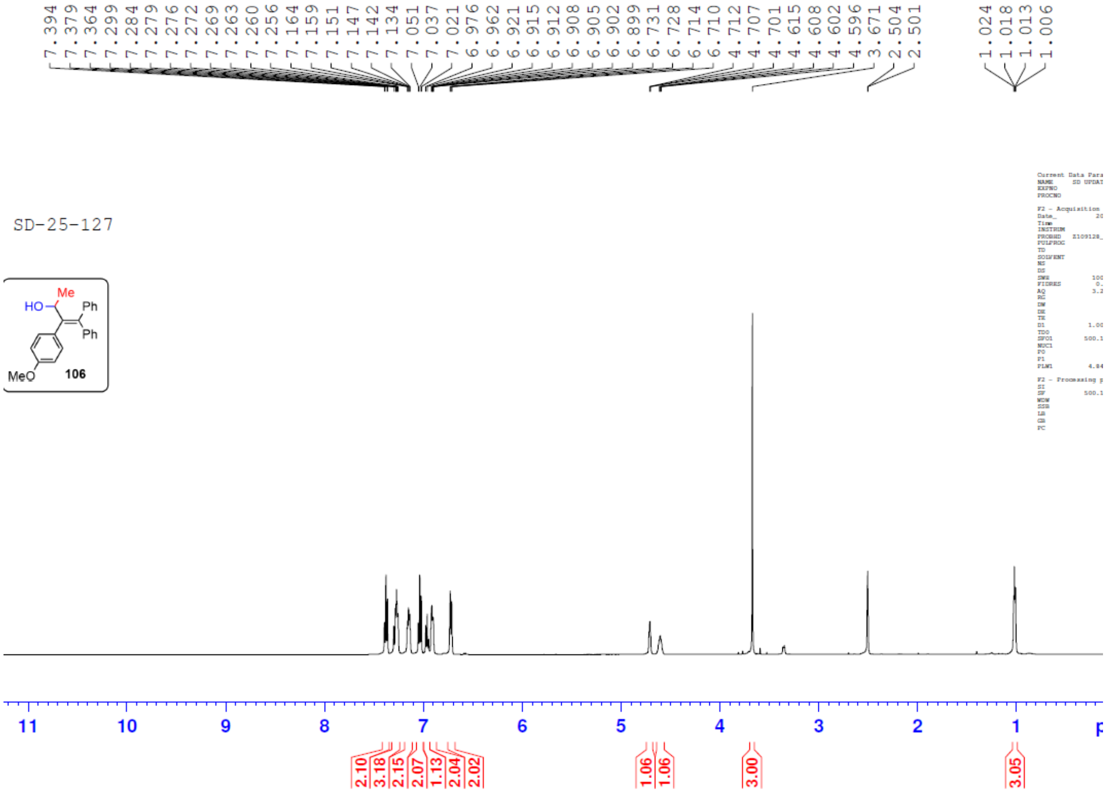

Supplementary Figure 252. ^{1}H NMR of compound **106**

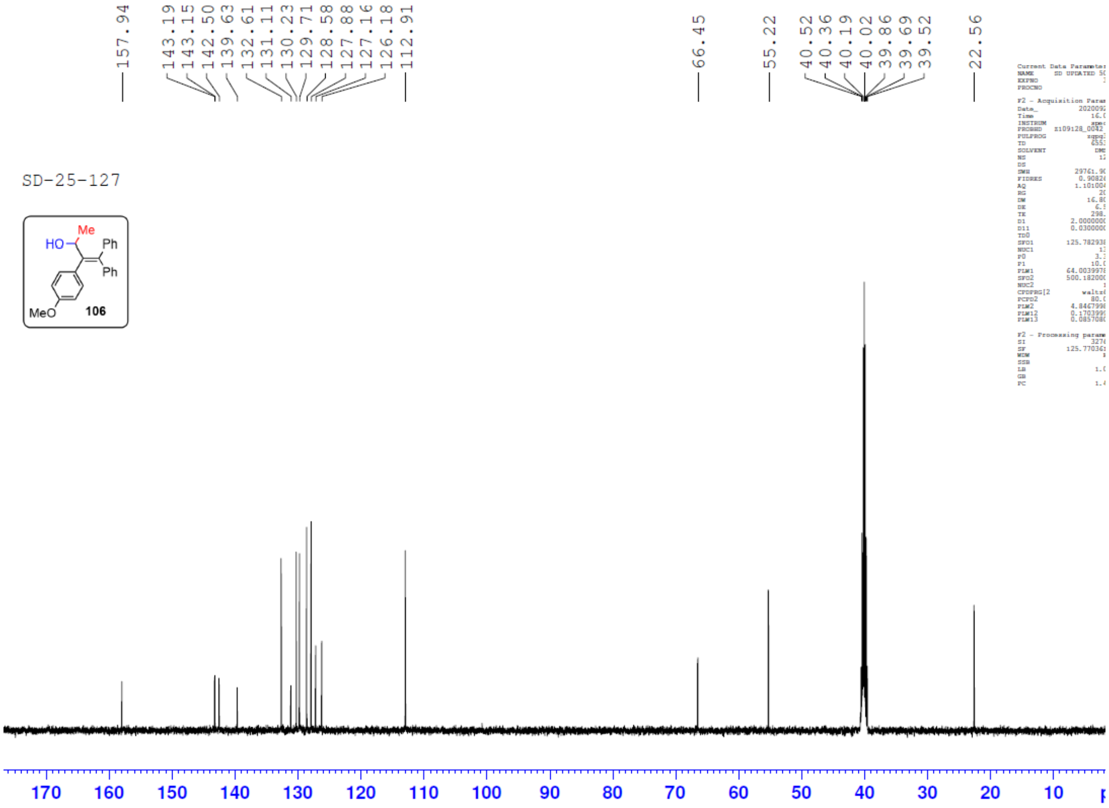

Supplementary Figure 253. ^{13}C NMR of compound **106**

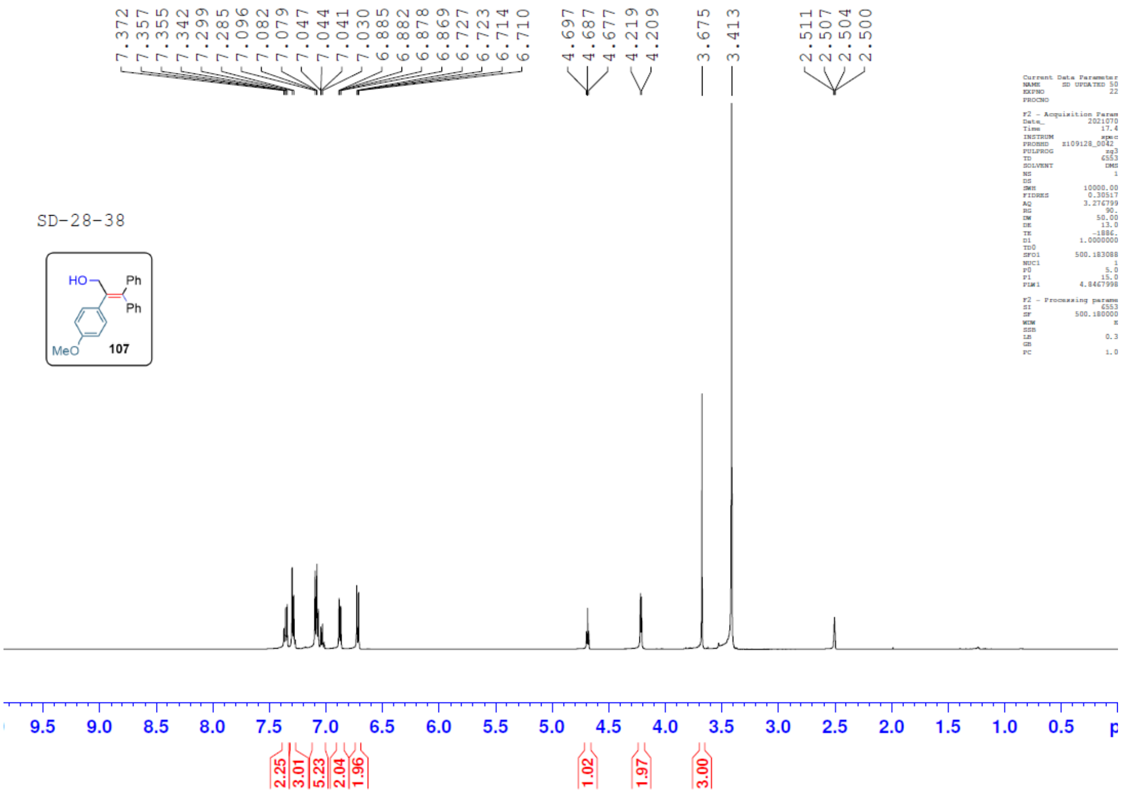

Supplementary Figure 254. ^{1}H NMR of compound **107**

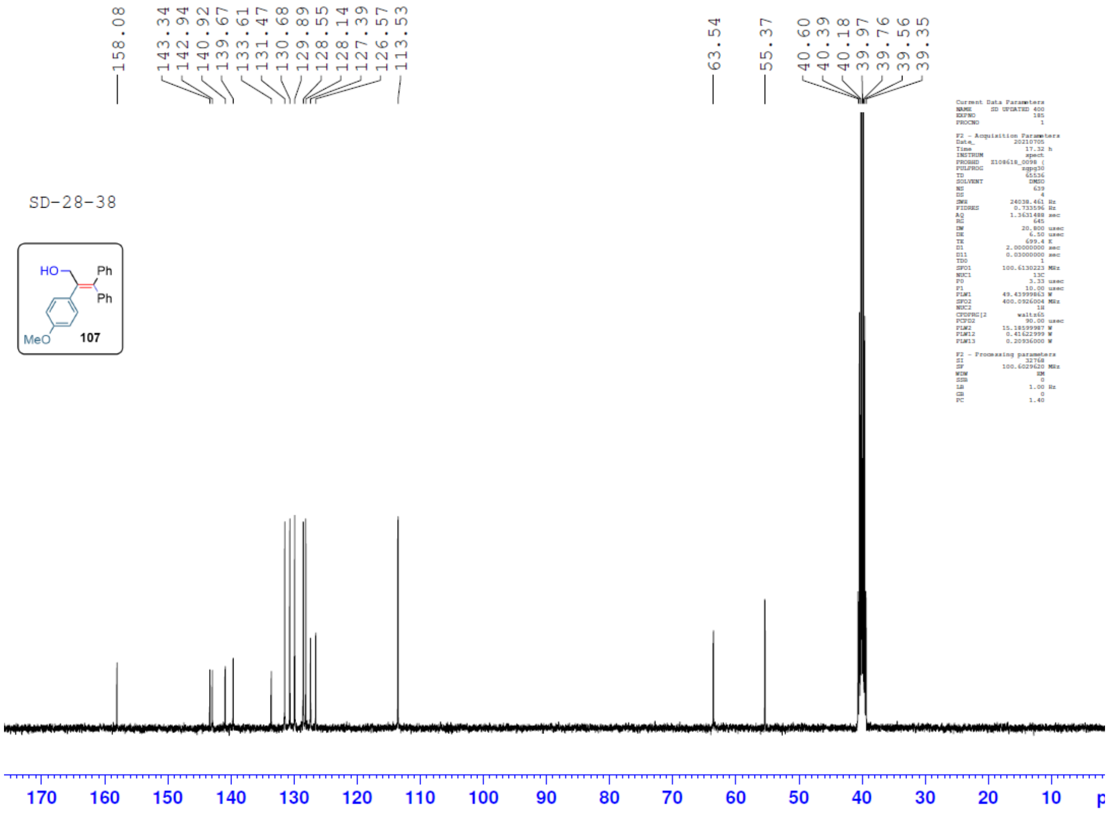

Supplementary Figure 255. ^{13}C NMR of compound **107**

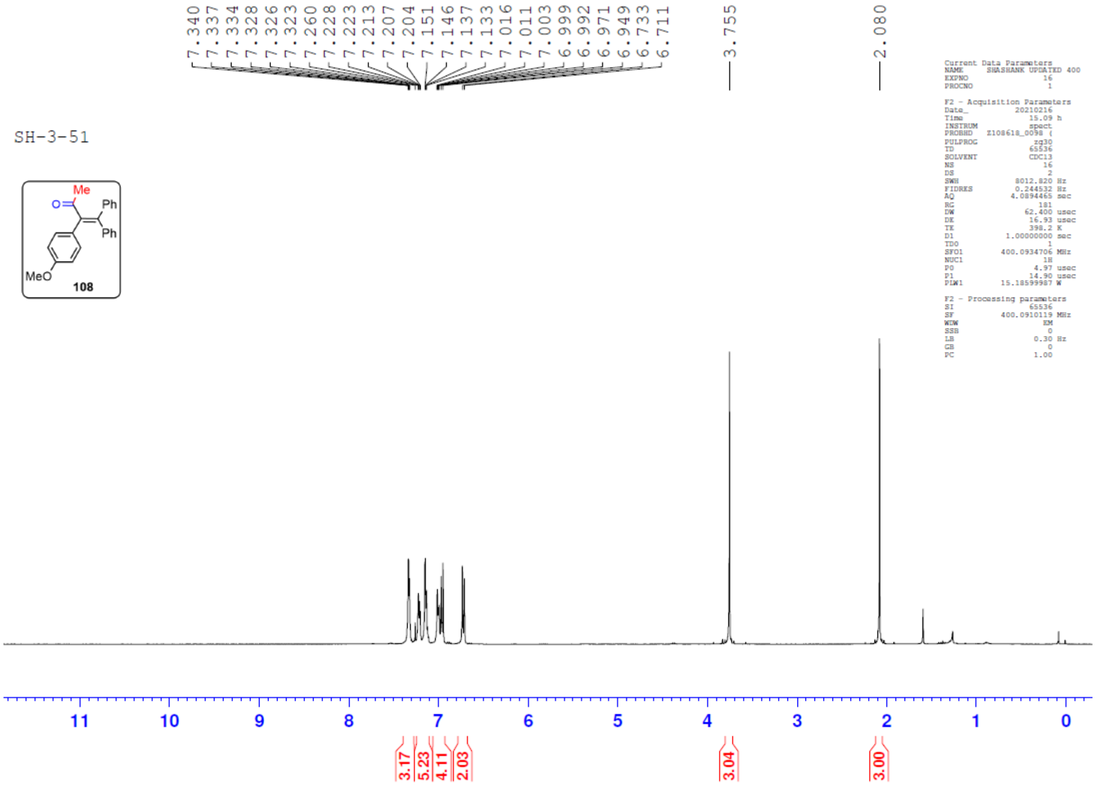

Supplementary Figure 256. ^{1}H NMR of compound **108**

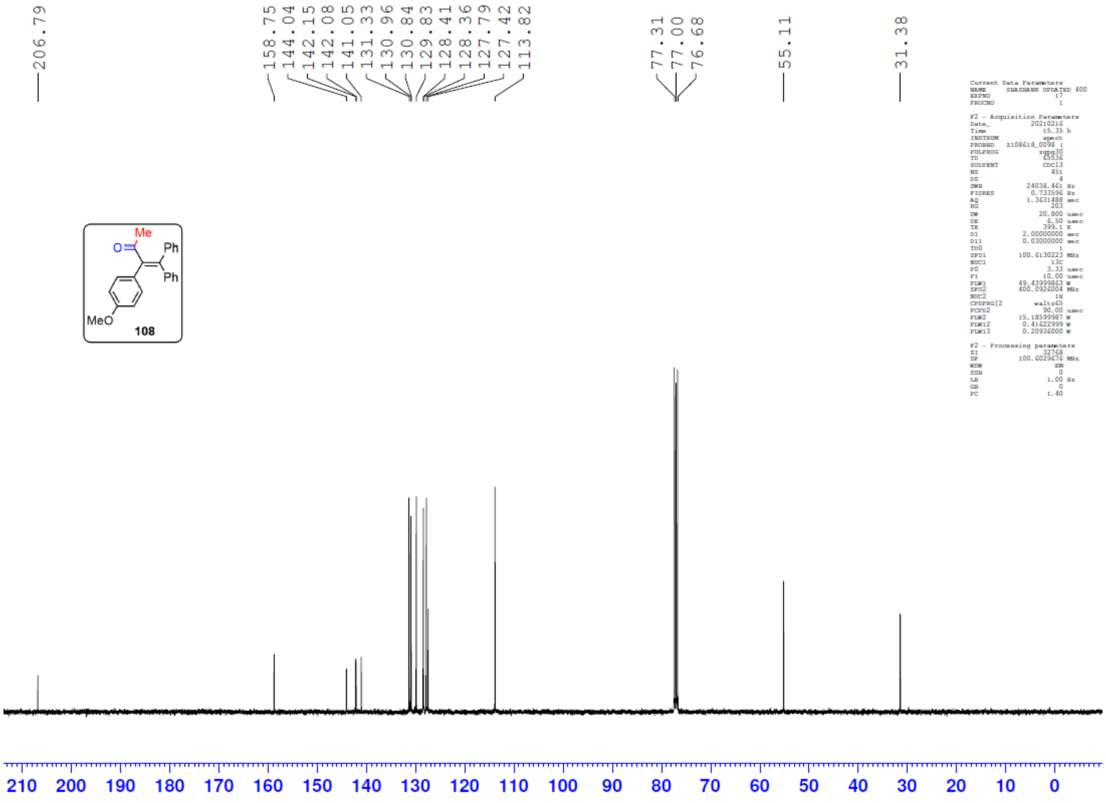

Supplementary Figure 257. ^{13}C NMR of compound **108**

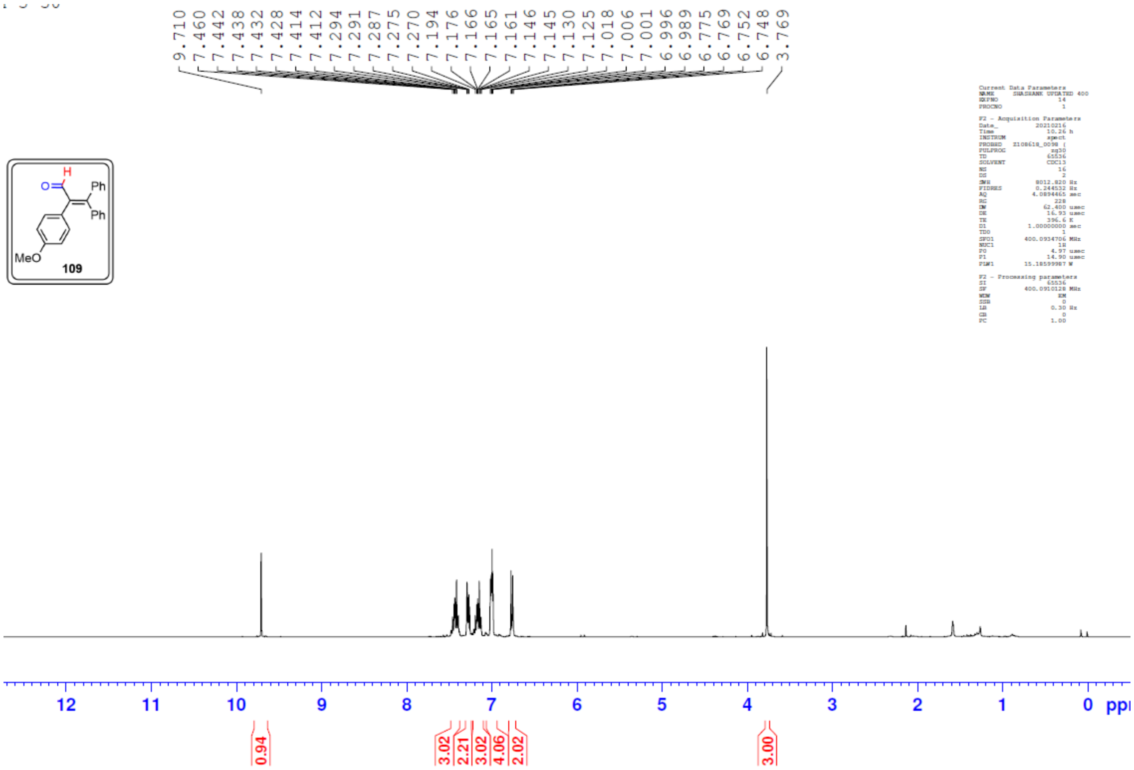

Supplementary Figure 258. ^{1}H NMR of compound **109**

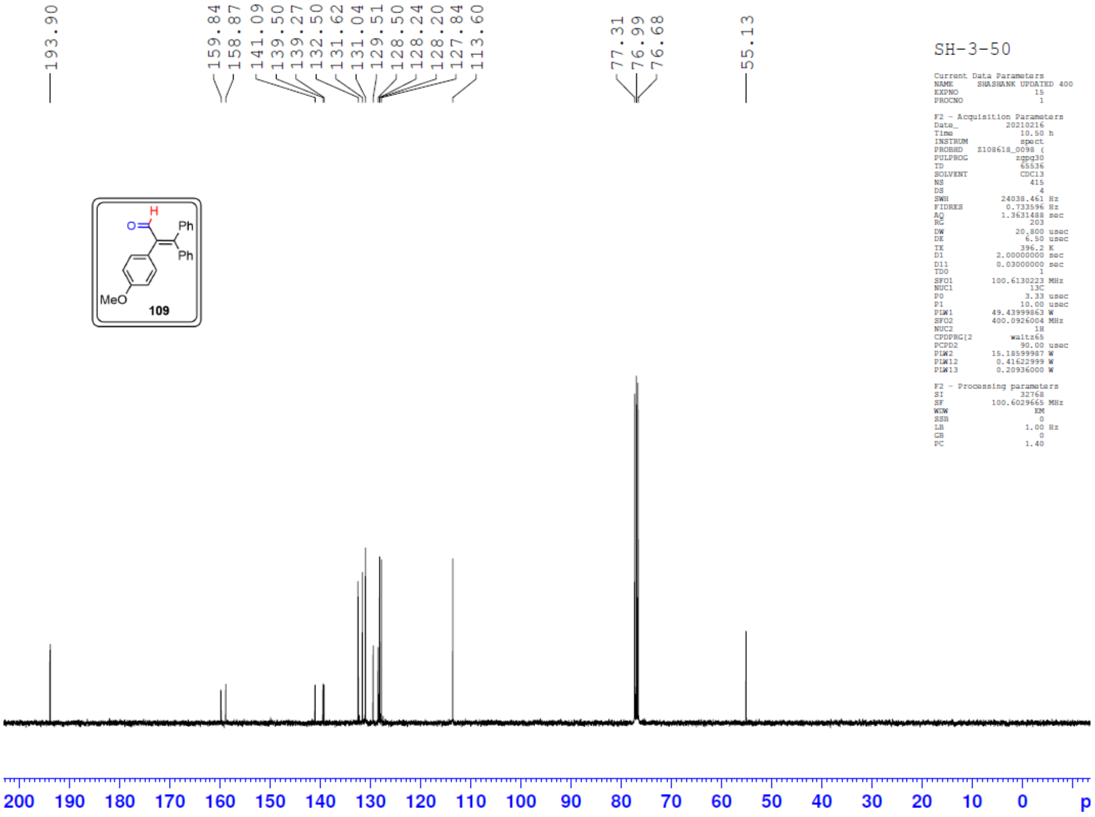

Supplementary Figure 259. ^{13}C NMR of compound **109**

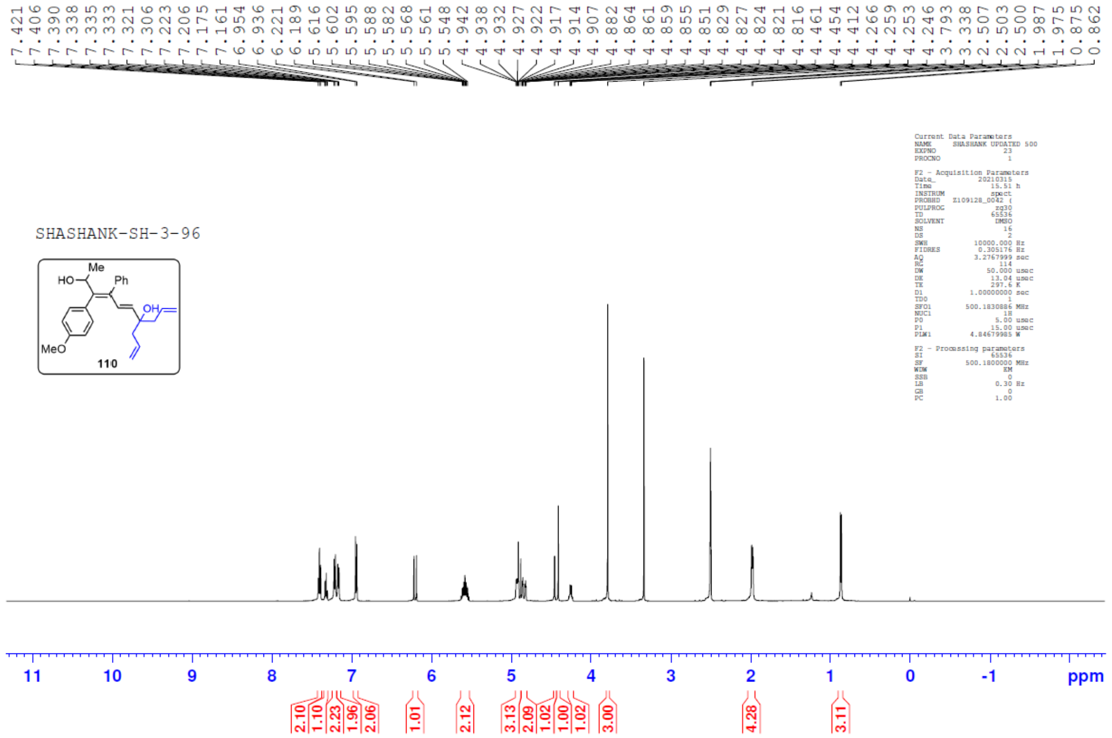

Supplementary Figure 260. ^{1}H NMR of compound **110**

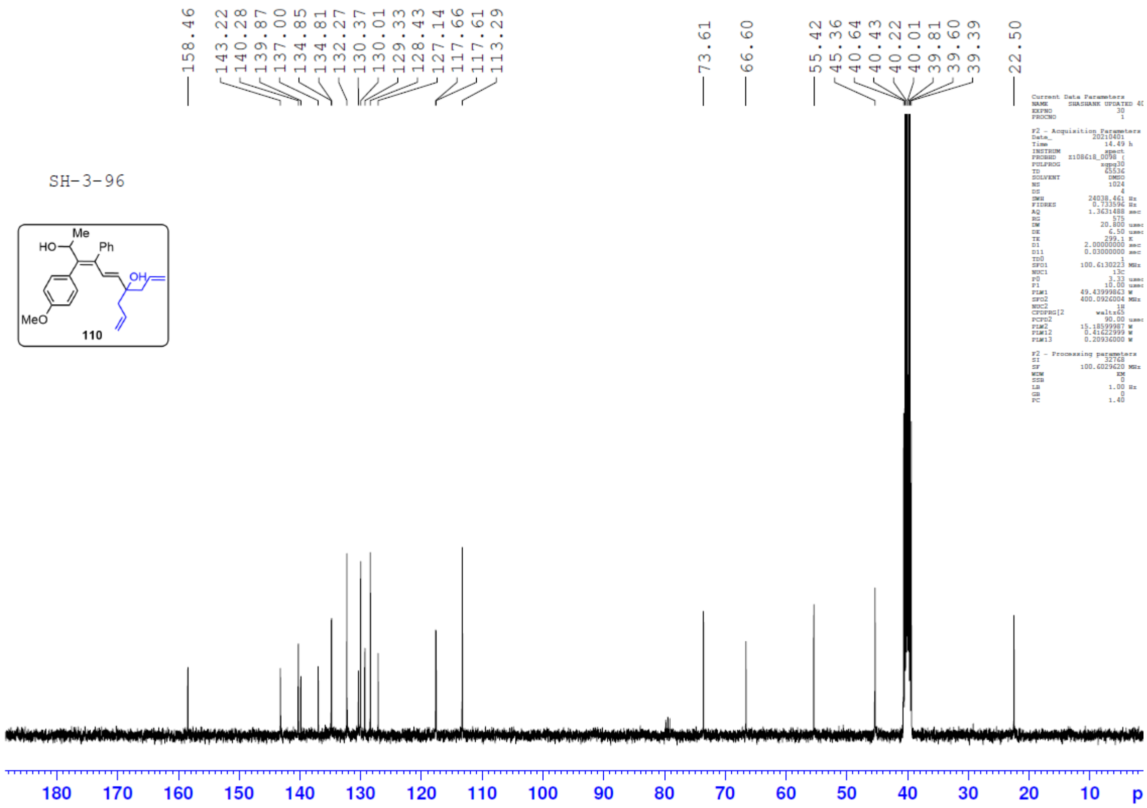

Supplementary Figure 261. ^{13}C NMR of compound **110**

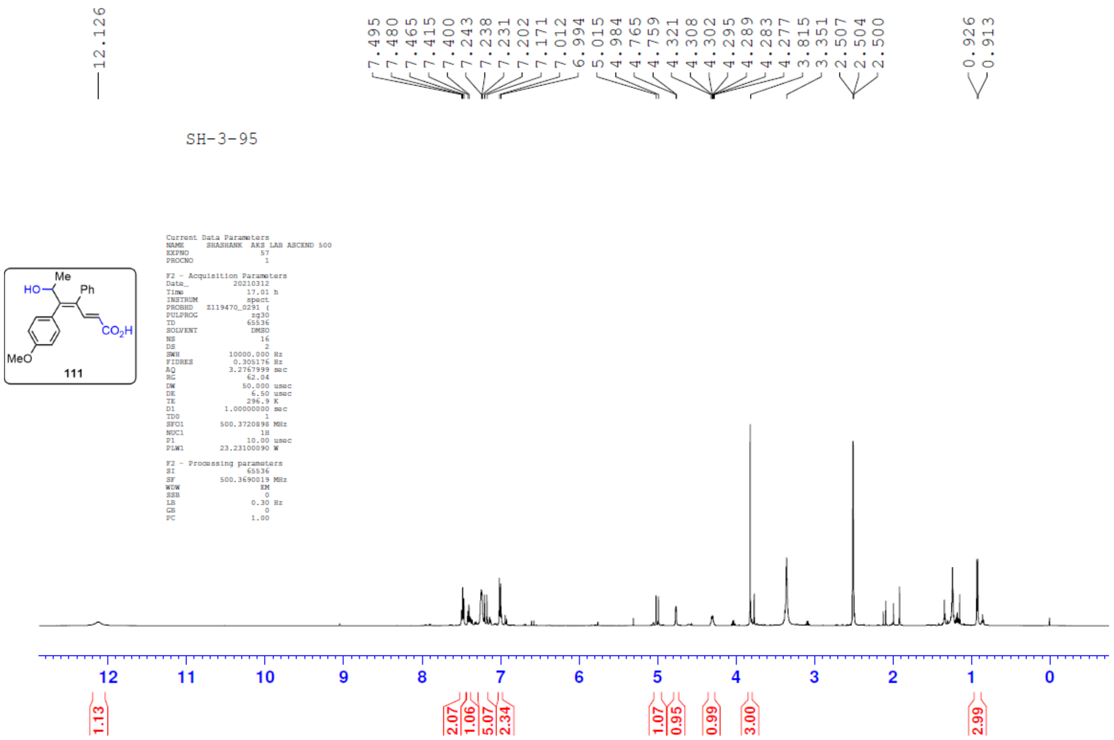

Supplementary Figure 262. ^{1}H NMR of compound **111**

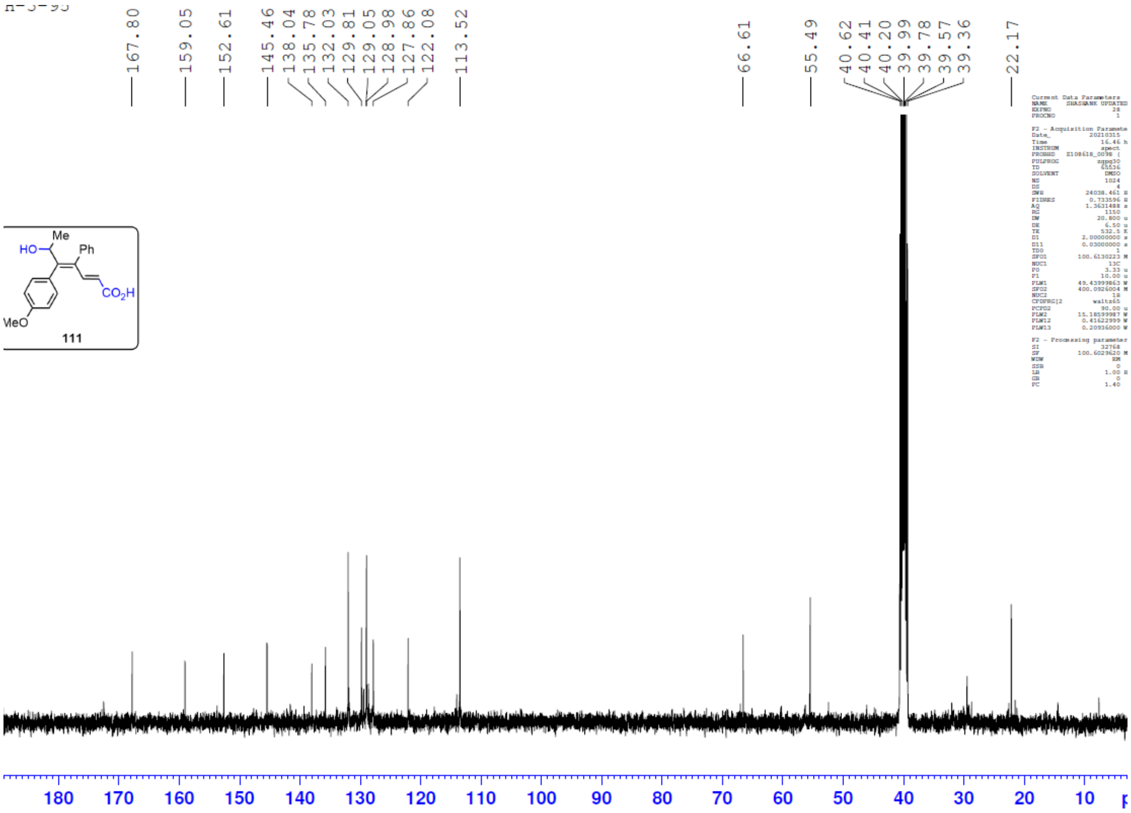

Supplementary Figure 263. ^{13}C NMR of compound **111**

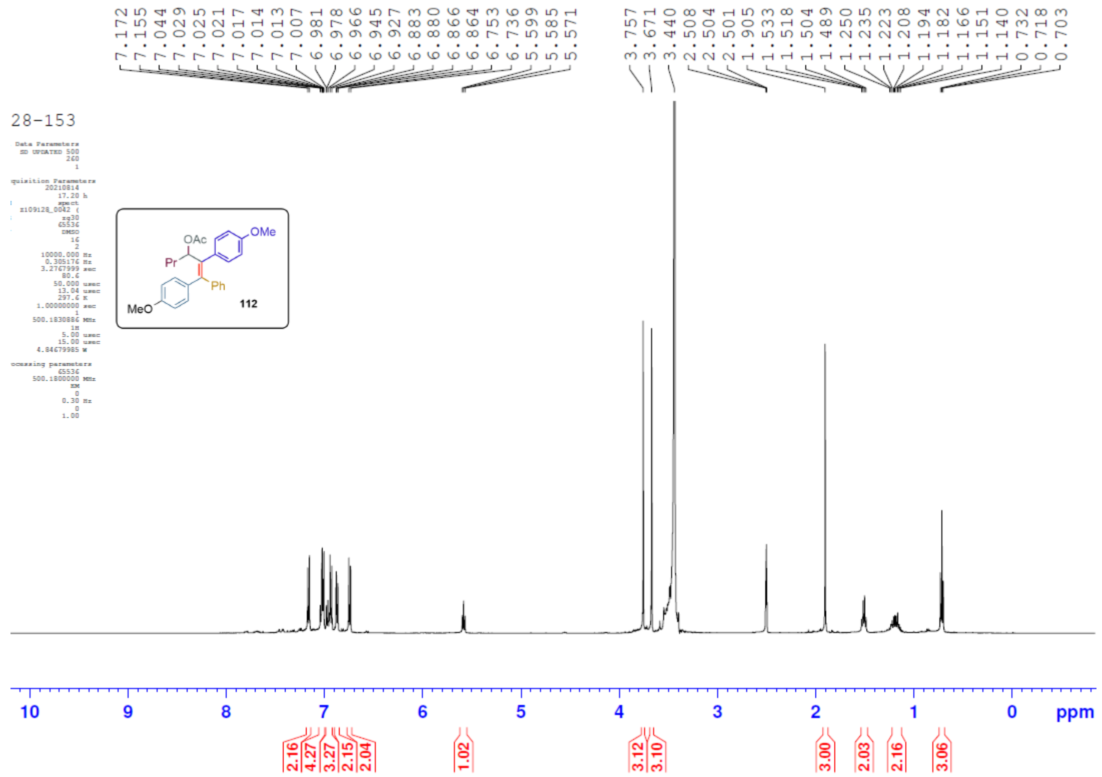

Supplementary Figure 264. ^{1}H NMR of compound **112**

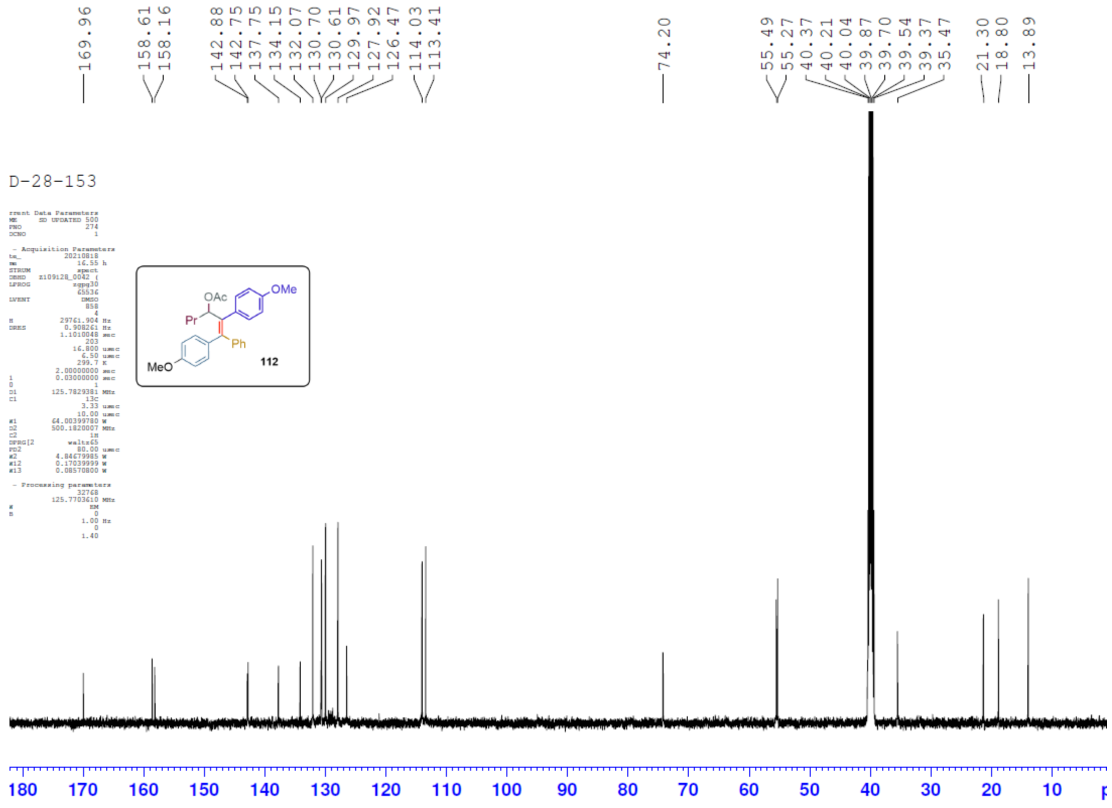

Supplementary Figure 265. ^{13}C NMR of compound **112**

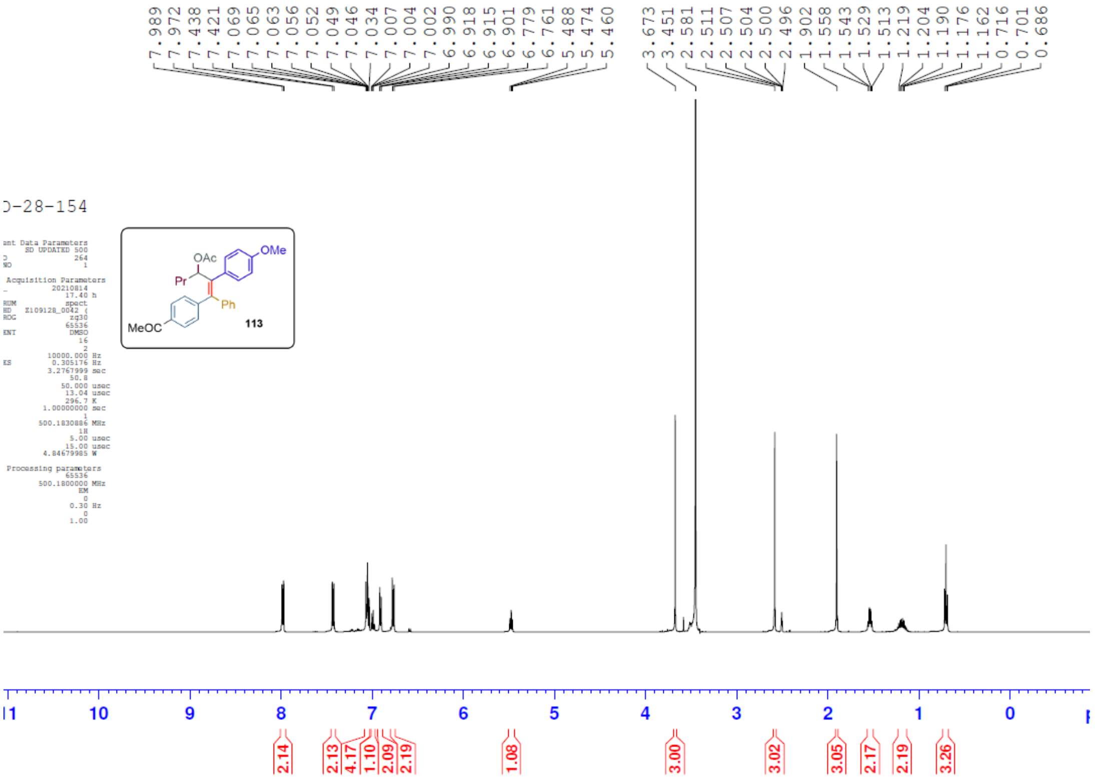

Supplementary Figure 266. ^{1}H NMR of compound **113**

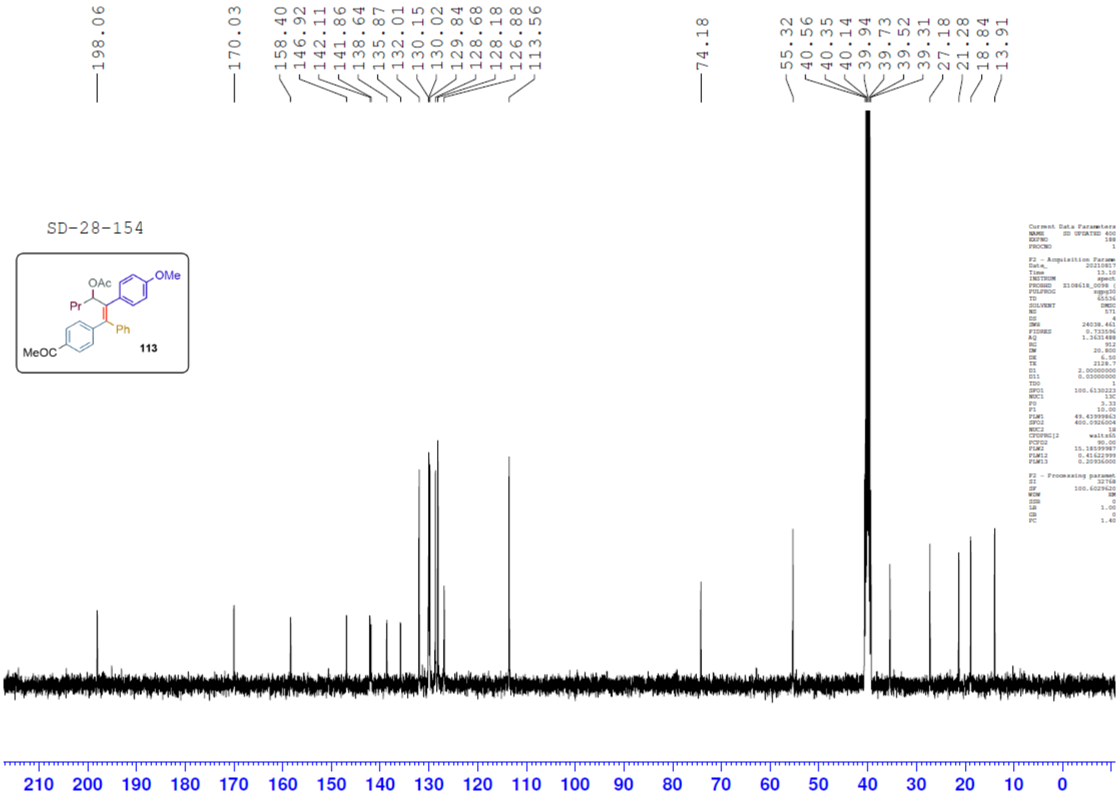

Supplementary Figure 267. ^{13}C NMR of compound **113**

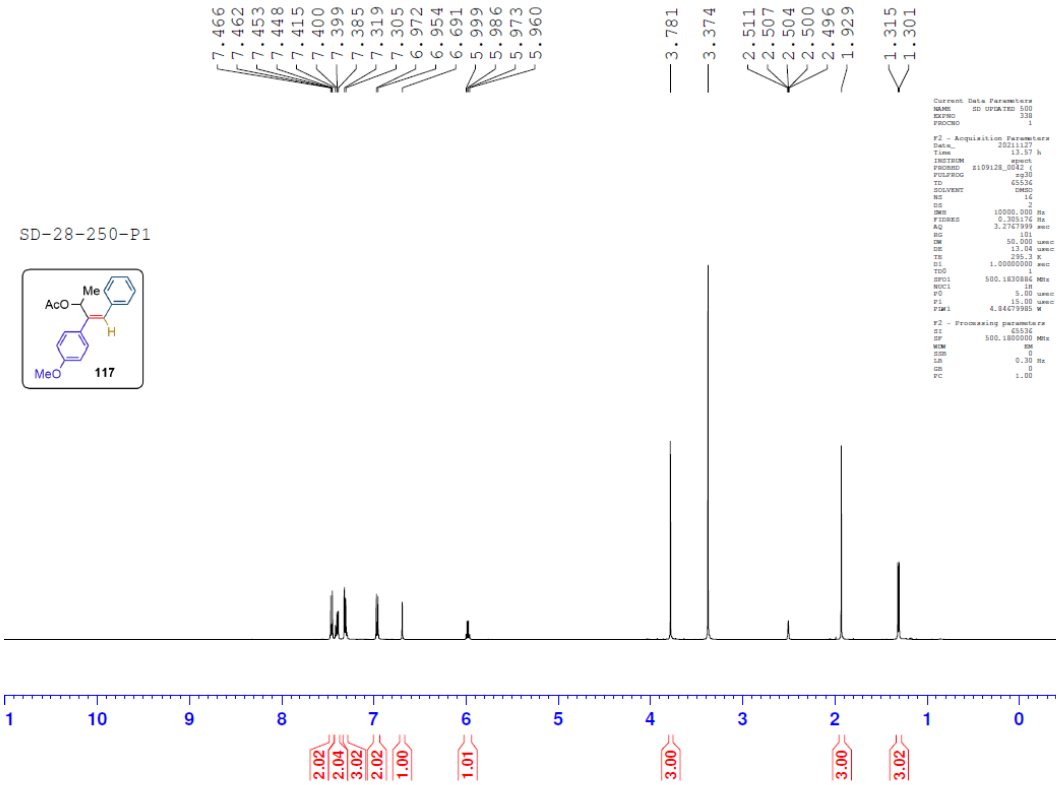

Supplementary Figure 268. ^{1}H NMR of compound **117**

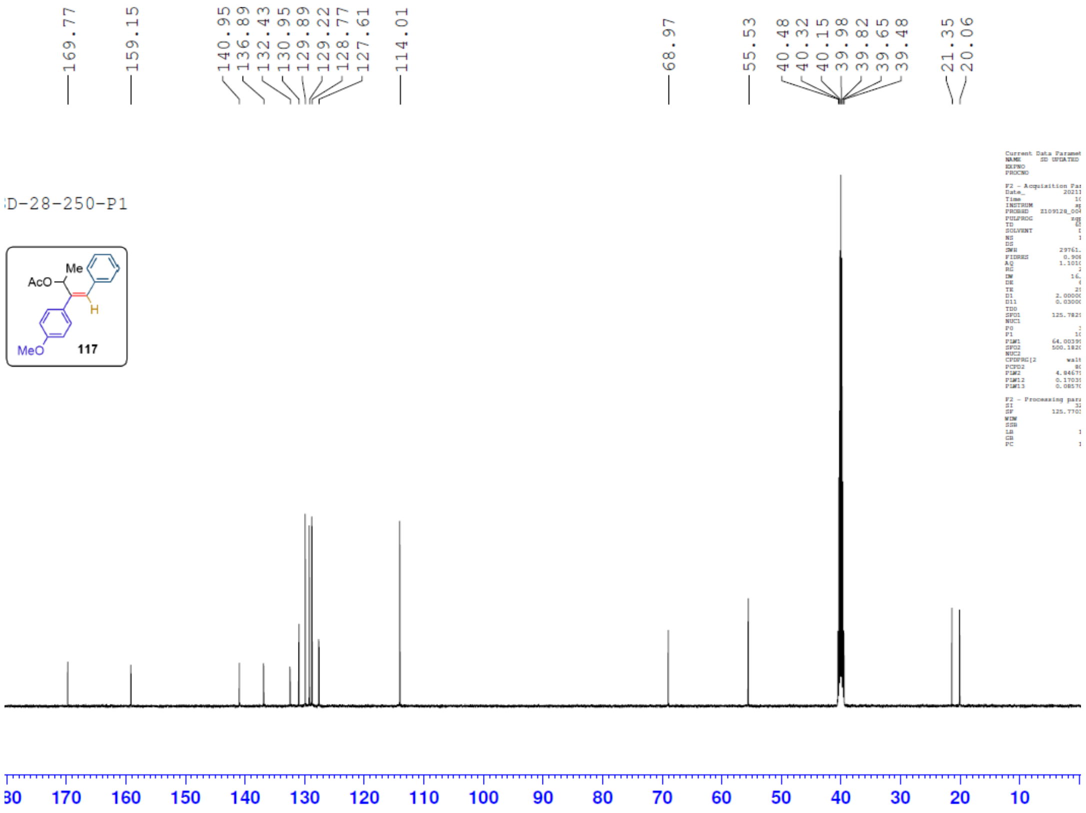

Supplementary Figure 269. ^{13}C NMR of compound **117**

Supplementary References

1. N. Ghosh, S. Nayak, A. K. Sahoo, *J. Org. Chem*. **2011**, *76*, 500–511.
2. N. Ghosh, S. Nayak, B. Prabagar, A. K. Sahoo, *J. Org. Chem*. **2014**, *79*, 2453–2462.
3. J. Panteleev, R. Y. Huang, E. K. J. Lui, M. Lautens, *Org. Lett.* **2011**, *13*, 5314–5317.
4. C. Tsukano, S. Yamamoto, Y. Takemoto, *Chem Pharm Bull (Tokyo)* **2015,** 63(9), 710-719.
5. R. Tomita, T. Koike, M. Akita. *Chem. Commun.,* **2017**, *53*, 4681-4684.
6. S. Dutta, S. Yang, R. Vanjari, R. K. Mallick, V. Gandon, A. K. Sahoo, *Angew. Chem., Int. Ed.* **2020**, *59*, 10785.
7. J. P. Perdew, W. Yue, *Phys. Rev. B*, **1986**, *33*, 8800;
8. J. P. Perdew, *Phys. Rev. B*, **1986**, *33*, 8822;
9. A. D. Becke, *Phys. Rev. A*, **1988**, *38*, 3098.
10. M. J. Frisch, G. W. Trucks, H. B. Schlegel, G. E. Scuseria, M. A. Robb, J. R. Cheeseman, G. Scalmani, V. Barone, B. Mennucci, G. A. Petersson, H. Nakatsuji, M. Caricato, X. Li, H. P. Hratchian, A. F. Izmaylov, J. Bloino, G. Zheng, J. L. Sonnenberg, M. Hada, M. Ehara, K. Toyota, R. Fukuda, J. Hasegawa, M. Ishida, T. Nakajima, Y. Honda, O. Kitao, H. Nakai, T. Vreven, J. A. Montgomery Jr, J. E. Peralta, F. Ogliaro, M. Bearpark, J. J. Heyd, E. Brothers, K. N. Kudin, V. N. Staroverov, T. Keith, R. Kobayashi, J. Normand, K. Raghavachari, A. Rendell, J. C. Burant, S. S. Iyengar, J. Tomasi, M. Cossi, N. Rega, J. M. Millam, M. Klene, J. E. Knox, J. B. Cross, V. Bakken, C. Adamo, J. Jaramillo, R. Gomperts, R. E. Stratmann, O. Yazyev, A. J. Austin, R. Cammi, C. Pomelli, J. W. Ochterski, R. L. Martin, K. Morokuma, V. G. Zakrzewski, G. A. Voth, P. Salvador, J. J. Dannenberg, S. Dapprich, A. D. Daniels, O. Farkas, J. B. Foresman, J. V. Ortiz, J. Cioslowski and D. J. Fox, Gaussian 09, revision D.01, Gaussian, Inc., Wallingford CT, 2013.
11. T. H. Dunning Jr, P. J. Hay, *Modern Theoretical Chemistry*, ed. H. F. Schaefer III, Plenum, New York, 1997, vol. 3;
12. P. J. Hay and W. R. Wadt, *J. Chem. Phys.*, **1985**, *82*, 270;
13. W. R. Wadt, P. J. Hay, *J. Chem. Phys.*, **1985**, *82*, 284;
14. P. J. Hay, W. R. Wadt, *J. Chem. Phys.*, **1985**, *82*, 299.
15. A. D. MacLean and G. S. Chandler, *J. Chem. Phys.*, **1980**, *72*, 5639;
16. R. Krishnan, J. S. Binkley, R. Seeger, J. A. Pople, *J. Chem. Phys.*, **1980**, *72*, 650.
17. Zhao, D. G. Truhlar, *J. Chem. Phys.*, **2006**, *125*, 194101.
18. F. Weigend, R. Ahlrichs, *Phys. Chem. Chem. Phys.*, **2005**, *7*, 3297;
19. F. Weigend, *Phys. Chem. Chem. Phys.*, **2006**, *8*, 1057.
20. E. Cancès, B. Mennucci, *J. Tomasi*, *J. Chem. Phys.*, **1997**, *107*, 3032;
21. M. Cossi, V. Barone, R. Cammi, J. Tomasi, *Chem. Phys. Lett.*, **1996**, *255*, 327.
22. E. R. Johnson, S. Keinan, P. Mori-Sánchez, J. Contreras-García, A. J. Cohen, W. Yang, *J. Am. Chem. Soc*. **2010**, *132*, 6498.
23. The PyMOL Molecular Graphics System, Version 2.0.4, Schrödinger, LLC.
24. C. Y. Legault, CYLview, 1.0b; Universite de Sherbrooke: Quebec, Canada, 2009; http://www.cylview.org.

25. W.-J. van Zeist, F. M. Bickelhaupt, *Org. Biomol. Chem*. **2010**, *8*, 3118-3127.
26. I. Fernández, F. P. Cossío, F. M. Bickelhaupt, *J. Org. Chem*. **2011**, *76*, 2310-2314.
27. I. Fernández, F. M. Bickelhaupt, *J. Comput. Chem*. **2012**, *33*, 509-516.
28. I. Fernández, L. P. Wolters, F. M. Bickelhaupt, *J. Comput. Chem*. **2014**, *35*, 2140-2145.
29. Some selected examples, see: a) X. Hong, Y. Liang, K. N. Houk, *J. Am. Chem. Soc*. **2014**, *136*, 2017-2025;
30. D. H. Ess, K. N. Houk, *J. Am. Chem. Soc.* **2007**, *129*, 10646-10647;
31. C. Y. Legault, Y. Garcia, C. A. Merlic, K. N. Houk, *J. Am. Chem. Soc.* **2007**, *129*, 12664-12665;
32. D. H. Ess, K. N. Houk, *J. Am. Chem. Soc*. **2008**, *130*, 10187-10198;
33. F. Liu, Y. Liang, K. N. Houk, *J. Am. Chem. Soc*. **2014**, *136*, 11483-11493;
34. A. G. Green, P. Liu, C. A. Merlic, K. N. Houk, *J. Am. Chem. Soc*. **2014**, *136*, 4575-4583.
35. S. G. Robinson, X. Wu, B. Jiang, M. S. Sigman, S. Lin, *J. Am. Chem. Soc*. **2020**, *142*, 18471−18482.
36. SAINT-Plus, *version 6.45*, Bruker AXS Inc. Madison, WI, **2003**.
37. Sheldrick, G. M. SADABS, *Program for Empirical Absorption Correction of Area Detector Data*, University of Gottingen, Germany, **1997**.
38. SMART (*version 5.625*), SHELX-TL (*version 6.12*), Bruker AXS Inc. Madison, WI, **2000**;
39. Sheldrick, G. M. SHELXS-97, SHELXL-97, University of Gottingen, Germmany, **1997**.
40. Dolomanov O. V., Blake A. J., Champness N. R., Schroder M. *J. Appl. Cryst*. **2003**, *36*, 1283.
